# Supplementary material for: The Effectiveness of Smoking Cessation, Alcohol Reduction, Diet and Physical Activity Interventions in Improving Maternal and Infant Health Outcomes: A Systematic Review of Meta-Analyses
Source: Nutrients. 2021 Mar 23;13(3):1036. doi: 10.3390/nu13031036 (PMC8005204; doi:10.3390/nu13031036)
Supplement: Supplementary file 1 [file nutrients-13-01036-s001.pdf]

S1 Table: PRISMA Checklist

Section/topic	#	Checklist item	Reported on page #
**TITLE**			
Title	1	Identify the report as a systematic review, meta-analysis, or both.	1
**ABSTRACT**			
Structured summary	2	Provide a structured summary including, as applicable: background; objectives; data sources; study eligibility criteria, participants, and interventions; study appraisal and synthesis methods; results; limitations; conclusions and implications of key findings; systematic review registration number.	3-4
**INTRODUCTION**			
Rationale	3	Describe the rationale for the review in the context of what is already known.	1
Objectives	4	Provide an explicit statement of questions being addressed with reference to participants, interventions, comparisons, outcomes, and study design (PICOS).	2
**METHODS**			
Protocol and registration	5	Indicate if a review protocol exists, if and where it can be accessed (e.g., Web address), and, if available, provide registration information including registration number.	6
Eligibility criteria	6	Specify study characteristics (e.g., PICOS, length of follow-up) and report characteristics (e.g., years considered, language, publication status) used as criteria for eligibility, giving rationale.	7
Information sources	7	Describe all information sources (e.g., databases with dates of coverage, contact with study authors to identify additional studies) in the search and date last searched.	6-7, S2 Table
Search	8	Present full electronic search strategy for at least one database, including any limits used, such that it could be repeated.	S2 Table
Study selection	9	State the process for selecting studies (i.e., screening, eligibility, included in systematic review, and, if applicable, included in the meta-analysis).	6-7, S3 Table
Data collection process	10	Describe method of data extraction from reports (e.g., piloted forms, independently, in duplicate) and any processes for obtaining and confirming data from investigators.	7-8
Data items	11	List and define all variables for which data were sought (e.g., PICOS, funding sources) and any assumptions and simplifications made.	7
Risk of bias in individual studies	12	Describe methods used for assessing risk of bias of individual studies (including specification of whether this was done at the study or outcome level), and how this information is to be used in any data synthesis.	7-8, S4 Table
Summary measures	13	State the principal summary measures (e.g., risk ratio, difference in means).	8
Synthesis of results	14	Describe the methods of handling data and combining results of studies, if done, including measures of consistency (e.g., I^2) for each meta-analysis.	8-9

Section/topic	#	Checklist item	Reported on page #
Risk of bias across studies	15	Specify any assessment of risk of bias that may affect the cumulative evidence (e.g., publication bias, selective reporting within studies).	7-8
Additional analyses	16	Describe methods of additional analyses (e.g., sensitivity or subgroup analyses, meta-regression), if done, indicating which were pre-specified.	N/A
**RESULTS**			
Study selection	17	Give numbers of studies screened, assessed for eligibility, and included in the review, with reasons for exclusions at each stage, ideally with a flow diagram.	9, Fig 1
Study characteristics	18	For each study, present characteristics for which data were extracted (e.g., study size, PICOS, follow-up period) and provide the citations.	9-12, Table 1, S5 Table
Risk of bias within studies	19	Present data on risk of bias of each study and, if available, any outcome level assessment (see item 12).	12-13, Table 2, S6 table
Results of individual studies	20	For all outcomes considered (benefits or harms), present, for each study: (a) simple summary data for each intervention group (b) effect estimates and confidence intervals, ideally with a forest plot.	S8-18 Tables<br>Figures 2-16
Synthesis of results	21	Present results of each meta-analysis done, including confidence intervals and measures of consistency.	15-31
Risk of bias across studies	22	Present results of any assessment of risk of bias across studies (see Item 15).	12-13, Table 2, S6 table
Additional analysis	23	Give results of additional analyses, if done (e.g., sensitivity or subgroup analyses, meta-regression [see Item 16]).	N/A
**DISCUSSION**			
Summary of evidence	24	Summarize the main findings including the strength of evidence for each main outcome; consider their relevance to key groups (e.g., healthcare providers, users, and policy makers).	32-34
Limitations	25	Discuss limitations at study and outcome level (e.g., risk of bias), and at review-level (e.g., incomplete retrieval of identified research, reporting bias).	35-36
Conclusions	26	Provide a general interpretation of the results in the context of other evidence, and implications for future research.	37
**FUNDING**			
Funding	27	Describe sources of funding for the systematic review and other support (e.g., supply of data); role of funders for the systematic review.	37-38

From: Moher D, Liberati A, Tetzlaff J, Altman DG, The PRISMA Group (2009). Preferred Reporting Items for Systematic Reviews and Meta-Analyses: The PRISMA Statement. PLoS Med 6(7): e1000097. doi:10.1371/journal.pmed1000097

For more information, visit: **www.prisma-statement.org**.

S2 Table: Search terms

The databases searched were: Joanna Briggs Institute Database of Systematic Reviews and Implementation Reports, JBI COnNECT+, Cochrane Database of Systematic Reviews, Cochrane Database of Abstracts of Reviews of Effectiveness (DARE), Cochrane Health Technology Assessments, Cochrane Economic Evaluations, PROSPERO, Epistemonikos, Medline, CINAHL, AMED, ASSIA, LILACS, and Social Care Online. A combination of index and free-text terms were used to search, relating to the key concepts of pregnancy and antenatal care, and behaviour change interventions. Conference abstracts were excluded. To fulfil the requirements of an umbrella review, a systematic review search filter from the Scottish Intercollegiate Guidelines Network (SIGN) was applied. SIGN search filters are validated search filters which limit the search to a particular study type, in this case, systematic reviews.

The five Google Scholar searches were limited to publications from 2011 to date, results were sorted by relevance, and the first 10 pages (100 results) of each of the five searches were screened.

**Database: Medline <1974 to September 2020>**
1 exp Obesity/
2 obes*.tw.
3 body mass.tw.
4 exp Body Composition/
5 body composition.tw.
6 exp Body Size/
7 body siz*.tw.
8 bodysiz*.tw.
9 exp Body Weight/
10 body weight.tw.
11 fat.tw.
12 fatness.tw.
13 exp Overnutrition/
14 overnutrition.tw.
15 exp Overweight/
16 overweight.tw.
17 over weight.tw.
18 weight.tw.
19 exp Weight Gain/
20 weight gain.tw.
21 weight maintenance.tw.
22 weight management.tw.
23 1 or 2 or 3 or 4 or 5 or 6 or 7 or 8 or 9 or 10 or 11 or 12 or 13 or 14 or 15 or 16 or 17 or 18 or 19 or 20 or 21 or 22
24 exp Fasting/
25 intermittent fast*.tw.
26 alternate-day fast*.tw.
27 intermittent energy restriction*.tw.
28 intermittent calori* restriction*.tw.
29 intermittent restrictive diet*.tw.
30 continuous energy restriction*.tw.
31 continuous calori* restriction*.tw.
32 continuous restrictive diet*.tw.
33 fasting calorie restriction intervention*.tw.
34 very low calorie diet*.tw.
35 periodic fasting*.tw.
36 extreme diet*.tw.
37 800* kcal.tw.
38 500 calorie*.tw.
39 sporadic fast*.tw.
40 24 or 25 or 26 or 27 or 28 or 29 or 30 or 31 or 32 or 33 or 34 or 35 or 36 or 37 or 38 or 39
41 23 and 40
42 exp Adiposity/

43	exp Adipose Tissue/
44	(adverse adj (event* or inciden*)).tw.
45	bio-impedance.tw.
46	bioimpedance.tw.
47	bioelectrical impedance analysis.tw.
48	exp Blood Glucose/
49	blood glucose.tw.
50	exp Blood Pressure/
51	blood pressure*.tw.
52	exp Body Mass Index/
53	body mass index.tw.
54	BMI.tw.
55	bodpod.tw.
56	exp Cholesterol/
57	cholesterol.tw.
58	exp Diet/
59	diet.tw.
60	exp Absorptiometry, Photon/
61	dexa scan*.tw.
62	dxa.tw.
63	exp Exercise/
64	exercise.tw.
65	hydrostatic.tw.
66	exp Magnetic Resonance Imaging/
67	magnetic resonance imag*.tw.
68	MRI.tw.
69	exp Skinfold Thickness/
70	skin-fold.tw.
71	exp Waist Circumference/
72	waist circumference.tw.
73	exp Weight Loss/
74	weight loss.tw.
75	slim.tw.
76	slimming.tw.
77	thin.tw.
78	thinness.tw.
79	42 or 43 or 44 or 45 or 46 or 47 or 48 or 49 or 50 or 51 or 52 or 53 or 54 or 55 or 56 or 57 or 58 or 59 or 60 or 61 or 62 or 63 or 64 or 65 or 66 or 67 or 68 or 69 or 70 or 71 or 72 or 73 or 74 or 75 or 76 or 77 or 78
80	23 and 40 and 79
81	limit 80 to english language
82	80 not 81
83	exp Randomized Controlled Trials as Topic/
84	exp Randomized Controlled Trial/
85	exp Random Allocation/
86	exp Double-Blind Method/
87	exp Single-Blind Method/
88	exp Clinical Trial/
89	clinical trial, phase i.pt.
90	clinical trial, phase ii.pt.
91	clinical trial, phase iii.pt.
92	clinical trial, phase iv.pt.
93	controlled clinical trial.pt.
94	randomized controlled trial.pt.
95	multicenter study.pt.
96	clinical trial.pt.
97	exp Clinical Trials as topic/
98	or/83-97
99	(clinical adj trial*).tw.
100	((singl* or doubl* or treb* or tripl*) adj (blind* or mask*)).tw.
101	exp Placebos/
102	placebo$.tw.

103	randomly allocated.tw.
104	(allocated adj2 random$).tw.
105	or/99-104
106	98 or 105
107	case report.tw.
108	letter/
109	historical article/
110	or/107-109
111	106 not 110
112	81 and 111
**Database: Embase <1974 to September 2020>**	
1	exp obesity/
2	obes*.tw.
3	exp body mass/
4	body mass.tw.
5	exp body composition/
6	body composition.tw.
7	exp body size/
8	body siz*.tw.
9	bodysiz*.tw.
10	exp body weight/
11	body weight.tw.
12	exp fat body/
13	fat.tw.
14	fatness.tw.
15	exp overnutrition/
16	overnutrition.tw.
17	overweight.tw.
18	over weight.tw.
19	weight.tw.
20	exp weight gain/
21	weight gain.tw.
22	weight maintenance.tw.
23	weight management.tw.
24	1 or 2 or 3 or 4 or 5 or 6 or 7 or 8 or 9 or 10 or 11 or 12 or 13 or 14 or 15 or 16 or 17 or 18 or 19 or 20 or 21 or 22 or 23
25	exp diet restriction/
26	fasting.tw.
27	intermittent fast*.tw.
28	alternate-day fast*.tw.
29	exp caloric restriction/
30	intermittent energy restriction*.tw.
31	intermittent calori* restriction*.tw.
32	intermittent restrictive diet*.tw.
33	continuous energy restriction*.tw.
34	continuous calori* restriction*.tw.
35	continuous restrictive diet*.tw.
36	fasting calorie restriction intervention*.tw.
37	very low calorie diet*.tw.
38	periodic fasting*.tw.
39	extreme diet*.tw.
40	800* kcal.tw.
41	500 calorie*.tw.
42	sporadic fast*.tw.
43	25 or 26 or 27 or 28 or 29 or 30 or 31 or 32 or 33 or 34 or 35 or 36 or 37 or 38 or 39 or 40 or 41 or 42
44	24 and 43
45	adiposity.tw.
46	exp adipose tissue/
47	(adverse adj (event* or inciden*)).tw.
48	bio-impedance.tw.

49	bioimpedance.tw.
50	bioelectrical impedance analysis.tw.
51	exp glucose blood level/
52	blood glucose.tw.
53	exp blood pressure/
54	blood pressure*.tw.
55	body mass index.tw.
56	BMI.tw.
57	bodpod.tw.
58	exp cholesterol/
59	cholesterol.tw.
60	exp diet/
61	diet.tw.
62	exp photon absorptiometry/
63	exp dual energy X ray absorptiometry/
64	dexa scan*.tw.
65	dxa.tw.
66	exp exercise/
67	exercise.tw.
68	hydrostatic.tw.
69	exp nuclear magnetic resonance imaging/
70	magnetic resonance imag*.tw.
71	MRI.tw.
72	exp skinfold thickness/
73	skin-fold.tw.
74	exp waist circumference/
75	waist circumference.tw.
76	exp weight reduction/
77	weight loss.tw.
78	slim.tw.
79	slimming.tw.
80	thin.tw.
81	thinness.tw.
82	45 or 46 or 47 or 48 or 49 or 50 or 51 or 52 or 53 or 54 or 55 or 56 or 57 or 58 or 59 or 60 or 61 or 62 or 63 or 64 or 65 or 66 or 67 or 68 or 69 or 70 or 71 or 72 or 73 or 74 or 75 or 76 or 77 or 78 or 79 or 80 or 81
83	24 and 43 and 82
84	limit 83 to english
85	83 not 84
86	limit 83 to (conference abstract or conference paper or conference proceeding or "conference review")
87	83 not 86
88	clinical trial/
89	randomized controlled trial/
90	exp randomization/
91	single blind procedure/
92	double blind procedure/
93	crossover procedure/
94	exp placebo/
95	randomi?ed controlled trial*.tw.
96	RCT.tw.
97	random allocation.tw.
98	randomly allocated.tw.
99	allocated randomly.tw.
100	(allocated adj2 random).tw.
101	single blind*.tw.
102	double blind*.tw.
103	(treble adj blind*).tw.
104	(triple adj blind*).tw.
105	placebo*.tw.
106	exp prospective study/
107	or/88-106

108	exp case study/
109	case report.tw.
110	abstract report/ or letter/
111	or/108-110
112	107 not 111
113	87 and 112
**Database: CINAHL (Cumulative Index of Nursing and Allied Health Literature <1981 to September 2020>**	
S1	(MH "Obesity+")
S2	TI obes* OR AB obes*
S3	TI body mass OR AB body mass
S4	(MH "Body Composition+")
S5	TI body composition OR AB body composition
S6	(MH "Body Size")
S7	TI body siz* OR AB body siz*
S8	TI bodysiz* OR AB bodysiz*
S9	(MH "Body Weight+")
S10	TI body weight OR AB body weight
S11	TI fat OR AB fat
S12	TI fatness OR AB fatness
S13	TI overnutrition OR AB overnutrition
S14	TI overweight OR AB overweight
S15	TI over weight OR AB over weight
S16	TI weight OR AB weight
S17	(MH "Weight Gain+")
S18	TI weight gain OR AB weight gain
S19	(MH "Weight Control")
S20	TI weight maintenance OR AB weight maintenance
S21	TI weight management OR AB weight management
S22	S1 OR S2 OR S3 OR S4 OR S5 OR S6 OR S7 OR S8 OR S9 OR S10 OR S11 OR S12 OR S13 OR S14 OR S15 OR S16 OR S17 OR S18 OR S19 OR S20 OR S21
S23	(MH "Fasting")
S24	TI intermittent fast* OR AB intermittent fast*
S25	TI alternate-day fast* OR AB alternate-day fast*
S26	(MH "Restricted Diet+")
S27	TI intermittent energy restriction* OR AB intermittent energy restriction*
S28	TI intermittent calori* restriction* OR AB intermittent calori* restriction*
S29	TI intermittent restrictive diet* OR AB intermittent restrictive diet*
S30	TI continuous energy restriction* OR AB continuous energy restriction*
S31	TI continuous calori* restriction* OR AB continuous calori* restriction*
S32	TI continuous restrictive diet* OR AB continuous restrictive diet*
S33	TI fasting calorie restriction intervention* OR AB fasting calorie restriction intervention*
S34	TI very low calorie diet* OR AB very low calorie diet*
S35	TI periodic fasting* OR AB periodic fasting*
S36	TI extreme diet* OR AB extreme diet*
S37	TI 800* kcal OR AB 800* kcal
S38	TI 500 calorie* OR AB 500 calorie*
S39	TI sporadic fast* OR AB sporadic fast*
S40	S23 OR S24 OR S25 OR S26 OR S27 OR S28 OR S29 OR S30 OR S31 OR S32 OR S33 OR S34 OR S35 OR S36 OR S37 OR S38 OR S39
S41	S22 AND S40
S42	TI adiposity OR AB adiposity
S43	(MH "Adipose Tissue+")
S44	TI "adverse event*" OR AB "adverse event*"
S45	TI "adverse inciden*" OR AB "adverse inciden*"
S46	TI bio-impedance OR AB bio-impedance
S47	TI bioimpedance OR AB bioimpedance
S48	TI bioelectrical impedance analysis OR AB bioelectrical impedance analysis
S49	(MH "Blood Glucose")
S50	TI blood glucose OR AB blood glucose
S51	(MH "Blood Pressure+")

S52	TI blood pressure* OR AB blood pressure*
S53	(MH "Body Mass Index")
S54	TI "body mass index" OR AB "body mass index"
S55	TI BMI OR AB BMI
S56	TI bodpod OR AB bodpod
S57	(MH "Cholesterol+")
S58	TI cholesterol OR AB cholesterol
S59	(MH "Diet+")
S60	TI diet OR AB diet
S61	(MH "Absorptiometry, Photon")
S62	TI dexa scan* OR AB dexa scan*
S63	TI dxa OR AB dxa
S64	(MH "Exercise+")
S65	TI exercise OR AB exercise
S66	TI hydrostatic OR AB hydrostatic
S67	(MH "Magnetic Resonance Imaging+")
S68	TI magnetic resonance imag* OR AB magnetic resonance imag*
S69	TI MRI OR AB MRI
S70	(MH "Skinfold Thickness")
S71	TI skin-fold OR AB skin-fold
S72	(MH "Waist Circumference")
S73	TI waist circumference OR AB waist circumference
S74	(MH "Weight Loss+")
S75	TI weight loss OR AB weight loss
S76	TI slim OR AB slim
S77	TI slimming OR AB slimming
S78	TI thin OR AB thin
S79	TI thinness OR AB thinness
S80	S42 OR S43 OR S44 OR S45 OR S46 OR S47 OR S48 OR S49 OR S50 OR S51 OR S52 OR S53 OR S54 OR S55 OR S56 OR S57 OR S58 OR S59 OR S60 OR S61 OR S62 OR S63 OR S64 OR S65 OR S66 OR S67 OR S68 OR S69 OR S70 OR S71 OR S72 OR S73 OR S74 OR S75 OR S76 OR S77 OR S78 OR S79
S81	S22 AND S40 AND S80
**Database: Cochrane Library** Date Run: September 2020	
#1	MeSH descriptor: [Obesity] explode all trees
#2	obes*:ti,ab
#3	body mass:ti,ab
#4	MeSH descriptor: [Body Composition] explode all trees
#5	body composition:ti,ab
#6	MeSH descriptor: [Body Size] explode all trees
#7	body siz*:ti,ab
#8	bodysiz*:ti,ab
#9	MeSH descriptor: [Body Weight] explode all trees
#10	body weight:ti,ab
#11	fat:ti,ab
#12	fatness:ti,ab
#13	MeSH descriptor: [Overnutrition] explode all trees
#14	overnutrition:ti,ab
#15	MeSH descriptor: [Overweight] explode all trees
#16	overweight:ti,ab
#17	over weight:ti,ab
#18	weight:ti,ab
#19	MeSH descriptor: [Weight Gain] explode all trees
#20	weight gain:ti,ab
#21	weight maintenance:ti,ab
#22	weight management:ti,ab
#23	{or #1-#22}
#24	MeSH descriptor: [Fasting] explode all trees
#25	intermittent fast*:ti,ab
#26	alternate-day fast*:ti,ab
#27	intermittent energy restriction*:ti,ab

#28	intermittent calori* restriction*:ti,ab
#29	intermittent restrictive diet*:ti,ab
#30	continuous energy restriction*:ti,ab
#31	continuous calori* restriction*:ti,ab
#32	continuous restrictive diet*:ti,ab
#33	fasting calorie restriction intervention*:ti,ab
#34	very low calorie diet*:ti,ab
#35	periodic fasting*:ti,ab
#36	extreme diet*:ti,ab
#37	800* kcal:ti,ab
#38	500 calorie*:ti,ab
#39	sporadic fast*:ti,ab
#40	{or #24-#39}
#41	#23 and #40
#42	MeSH descriptor: [Adiposity] explode all trees
#43	MeSH descriptor: [Adipose Tissue] explode all trees
#44	adverse event*:ti,ab
#45	adverse inciden*:ti,ab
#46	bio-impedance:ti,ab
#47	bioimpedance:ti,ab
#48	bioelectrical impedance analysis:ti,ab
#49	MeSH descriptor: [Blood Glucose] explode all trees
#50	blood glucose:ti,ab
#51	MeSH descriptor: [Blood Pressure] explode all trees
#52	blood pressure*:ti,ab
#53	MeSH descriptor: [Body Mass Index] explode all trees
#54	body mass index:ti,ab
#55	BMI:ti,ab
#56	bodpod:ti,ab
#57	MeSH descriptor: [Cholesterol] explode all trees
#58	cholesterol:ti,ab
#59	MeSH descriptor: [Diet] explode all trees
#60	diet:ti,ab
#61	MeSH descriptor: [Absorptiometry, Photon] explode all trees
#62	dexa scan*:ti,ab
#63	dxa:ti,ab
#64	MeSH descriptor: [Exercise] explode all trees
#65	exercise:ti,ab
#66	hydrostatic:ti,ab
#67	MeSH descriptor: [Magnetic Resonance Imaging] explode all trees
#68	magnetic resonance imag*:ti,ab
#69	MRI:ti,ab
#70	MeSH descriptor: [Skinfold Thickness] explode all trees
#71	skin-fold:ti,ab
#72	MeSH descriptor: [Waist Circumference] explode all trees
#73	waist circumference:ti,ab
#74	MeSH descriptor: [Weight Loss] explode all trees
#75	weight loss:ti,ab
#76	slim:ti,ab
#77	slimming:ti,ab
#78	thin:ti,ab
#79	thinness:ti,ab
#80	{or #42-#79}
#81	#23 and #40 and #80

**Google Scholar** Date run: November 2020
diet pregnancy intervention systematic review meta-analysis
physical activity pregnancy intervention systematic review meta-analysis
smoking pregnancy intervention systematic review meta-analysis
alcohol pregnancy intervention systematic review meta-analysis
lifestyle pregnancy intervention systematic review meta-analysis

S3 Table: Screening tool based on the inclusion criteria of this systematic review of systematic reviews

**Author and Year:**
**Journal:**
**Title:**
**Name of reviewer:**
**Design:** This article is/ contains a systematic review or meta-analysis<br>**Yes / No**
**Language/Date:**<br>The review is published in English and was published since 2008<br>**Yes / No**
**Review type:**<br>The review is an effectiveness review considering quantitative evidence<br>For mixed methods reviews: the review contains quantitative evidence focusing on effectiveness<br>**Yes / No**
**Participants:** Does the review include **any** types of pregnant women (regardless of socio demographic factors; such as age, ethnicity, parity, socioeconomic status and so forth), and NOT focus solely on women in the preconception/ postnatal phases.<br>**Yes / No**
**Interventions:**<br>Interventions of interest to the review focus on one of the following target behaviours; weight management interventions**,** smoking cessation interventions, and alcohol reduction interventions (or to behaviours that relate to these target behaviours i.e. physical activity or diet)<br>**Yes / No**
**Outcomes:**<br>Does the review report on any of the following outcomes: effectiveness on behaviour change (explicit)[1], or behaviour change (proxy), reach or usage of the intervention<br>**Yes / No**
**If you have not answered YES to all of the above questions, you should exclude the study.**<br>**If you answered yes to all, please continue.**

Footnote:

1. The screening form was developed for the wider programme of systematic reviews of systematic reviews; only systematic reviews of systematic reviews reporting on the effectiveness of interventions on proxy behaviour change (i.e. pregnancy outcomes or health-related outcomes) were included for this paper.

S4 Table: JBI Critical Appraisal Checklist for Systematic Reviews and Research Syntheses (Amended)

The JBI critical appraisal checklist comprises 11 questions relating to methodological rigor, transparency of reporting and appropriateness of conclusions and recommendations, with options of "yes" if the review clearly meets the checklist criteria, "no" and "unclear" " if the review does not clearly meet the criteria, and "not applicable". The reviews were awarded a score of 1 for each checklist criteria clearly met and 0 for those not met, with a maximum possible score of 11. The reviews were categorised as high quality if they scored 8-11, moderate quality for scores of 4-7, and low quality for scores of 0-3. No reviews were excluded based on quality score. The percentage of included reviews meeting the appraisal criteria was calculated for each of the 11 checklist questions.

Reviewer ____________________________ Date ________________________

Author ______________________________ Year _________ Record number _____

		Yes	No	Unclear	Not applicable
1.	Is the review question clearly and explicitly stated?	◻	◻	◻	◻
2.	Were the inclusion criteria appropriate for the review question? ***(Are all the PICO components clearly stated: Population, Intervention, Comparator, Outcome)***	◻	◻	◻	◻
3.	Was the search strategy appropriate? ***(Search strategy/keywords must be stated)***	◻	◻	◻	◻
4.	Were the sources and resources used to search for studies adequate? ***(At least 2 relevant databases should have been searched and at least one other data source [e.g. grey literature, reference list of included studies])***	◻	◻	◻	◻
5.	Were the criteria for appraising studies appropriate? ***(Authors must have described an appropriate method for assessing quality of included studies, e.g. CASP tool)***	◻	◻	◻	◻
6.	Was critical appraisal conducted by two or more reviewers independently?	◻	◻	◻	◻
7.	Were there methods to minimize errors in data extraction? ***(Either all data extraction was performed in duplicate or data extraction with validation methods employed)***	◻	◻	◻	◻

8.	Were the methods used to combine studies appropriate?<br>***(If meta-analysis performed did the authors justify their choice of method; if no meta-analysis is justification for this given?)***	□	□	□	□
9.	Was the likelihood of publication bias assessed?<br>***(Evidence of statistical testing for publication bias given and/or funnel plot presented)***	□	□	□	□
10.	Were recommendations for policy and/or practice supported by the reported data?	□	□	□	□
11.	Were the specific directives for new research appropriate?	□	□	□	□

Note: Adaptations to the JBI critical appraisal checklist [1] were made after piloting its use and checking for inconsistencies between reviewers in their interpretation of the critical review question. The further detail was added to aid consistency in interpretation, and used some if the descriptors from the AMSTAR critical appraisal tool [2].

References:

1. The Joanna Briggs Institute. The Joanna Briggs Institute reviewers' manual 2014: methodology for JBI umbrella reviews. Adelaide, Australia: The Joanna Briggs Institute; 2014.
2. Shea BJ, Reeves BC, Wells G, Thuku M, Hamel C, Moran J, et al. AMSTAR 2: a critical appraisal tool for systematic reviews that include randomised or non-randomised studies of healthcare interventions, or both. BMJ (Clinical research ed). 2017;358:j4008.

S5 Tables: Description of included systematic reviews according to the type of behaviour intervention

S5a Table: Systematic reviews reporting outcomes for smoking interventions

Review	Aim of the review	Search strategy	Inclusion criteria	Exclusion criteria	Included studies	Study locations
Chamberlain *et al.* 2017 [1]	To assess the effects of smoking cessation interventions during pregnancy on smoking behaviour and perinatal health outcomes.	Bibliographic databases: Cochrane Pregnancy and Childbirth's Trials Register<br>Supplementary Searches: Cited studies in trial reports and key reviews. Contacted the authors of any published protocols or 'ongoing studies'. Contacted trial authors to locate additional unpublished data.	● Individually-RCTs, cluster-RCT, and randomised cross-over trials<br>● Psychosocial interventions where a primary aim of the study was smoking cessation in pregnancy.<br>● Quasi-randomised studies if there was a very low risk of interference with the sequence generation.	● None reported	● 88 studies + 14 without outcome data =102<br>● Published: 1976 to 2015<br>● Study design: RCT n=88<br>● n>26,000 women	USA n=56<br>UK n=18<br>Norway n=3<br>Netherlands n=3<br>Sweden n=1<br>Spain n=3<br>Poland n=1<br>Australia/New Zealand n=11<br>Columbia n=1<br>Canada n=2<br>Greece n=1<br>Ireland n=1<br>Multiple countries n=1 (Argentina, Brazil, Cuba and Mexico)
Veisani *et al.* 2017 [2]	To obtain a pooled estimate the effect of prenatal smoking cessation on birth weight. (To assess the effects of smoking cessation in pregnancy period on the birth weight in RCT studies.	Bibliographic databases: Pub Med, Scopus, and Web of Science<br>Supplementary searches: cross-referring publications	● RCTs<br>● Assess the prenatal smoking cessation intervention<br>● Effects on birth weight	● Duplicate articles<br>● Poor-quality articles	● 16 studies<br>● Published: 1999 to 2016<br>● Study design: RCTs n=16<br>● n=6,192 women	UK n=4<br>USA n=7<br>France n=1<br>Poland n=1<br>Denmark n=1<br>Netherlands n=1<br>Australia n=1

S5b Table: Systematic reviews reporting outcomes for diet and/or physical activity interventions

Review		Aim of the review	Search strategy	Inclusion criteria	Exclusion criteria	Included studies	Study locations
Agha *et al.* 2014 [3]	Agha *et al.* 2014 [3]	To assess the efficacy of behavioural interventions for weight management during pregnancy in overweight, obese and morbidly obese women.	Bibliographic databases: Pub Med, Scopus, Cochrane Library, CINAHL, PsycINFO<br>Supplementary searches: reference list of all selected studies; reference list of NICE guidance on obesity; US NIH clinical Trials Registry; Google Scholar; Office for National Statistics and National Obesity Observatory contacted; personal communication with NICE	• English language.<br>• Parallel group study evaluating efficacy of behavioural interventions on weight management with standard maternity care/no intervention/ placebo.<br>• Women planning to get pregnant or those who are already pregnant.<br>• Underweight, normal weight, overweight, obese or morbidly obese.<br>• No limitations on age, ethnicity, SES, body weight.	• Studies of women with conditions such as diabetes and PCOS.<br>• Interventions based on weight reducing pharmaceutical or surgical interventions.<br>• Interventions aimed at other conditions/diseases in pregnancy or postpartum weight management.	• 14 studies<br>• Published: 2002 to 2011<br>• Study design: RCT n=12; CCT n=1; CBA n=1<br>• n=2,734 women	USA n=5<br>Australia n=2<br>Finland n=2<br>Sweden n=1<br>Norway n=1<br>Belgium n=1<br>Denmark n=1<br>Canada n=1
Bain *et al.* 2015 [4]	Bain *et al.* 2015 [4]	To assess the effects of dietary interventions in combination with physical exercise interventions for pregnant women for preventing GDM and associated adverse health consequences for the mother and her infant/child.	Bibliographic databases: MEDLINE; Embase; CINAHL<br>Supplementary searches: Cochrane Central Register of Controlled Trials (CENTRAL), reference lists of retrieved studies, hand searching journals, proceedings of major conferences; alerts for 44 journals, monthly BioMed Central alerts	• Pregnant women regardless of age, gestation, parity or plurality.	• Studies involving women with pre-existing type I or type II diabetes.	• 13 studies (39 records)<br>• Published: 2002 to 2014<br>• Study design: RCT n=11; Cluster RCT n=2<br>• n=4,983 women	USA n=3<br>Finland n=2<br>Australia n=2<br>UK n=1<br>Canada n=1<br>Italy n=1<br>Germany n=1<br>Denmark n=1<br>Egypt n=1
Allen *et al.* 2014 [5]	Allen *et al.* 2014 [5]	To evaluate the effect of dietary and lifestyle interventions with the potential to modify metabolic risk factors on the risk of PE	Bibliographic databases: MEDLINE, EMBASE and Cochrane<br>Supplementary searches: Reference lists of all primary and review articles.	• RCTs in pregnant women evaluating the effect of dietary and lifestyle interventions with the potential to modify metabolic risks such as obesity, hyperlipidemia/glycemia and hypertension on the risk of PE (results reported for diet/PA and diet only separately)	• Non-randomized<br>• Animal studies	• 18 studies<br>• Published: 1992 to 2012<br>• Study design: RCT n= 18<br>• n=8,712 women	Not reported
Campbell *et al.* 2011 [6]	Campbell *et al.* 2011 [6]	To explore the existing quantitative research evidence regarding the effectiveness of dietary interventions with or without PA in reducing the risk of excessive weight gain in pregnancy.	Bibliographic databases: ASSIA, British Nursing Index, Cinahl, Cochrane (Central, DARE, HTA, NHS EED, Database of Systematic Reviews), Econlit, Embase, Maternity and Infant Care, Medline, PyscINFO, Science Citation Index, Social Science Citation Index<br>Supplementary searches: bibliography of all included studies, experts in the field consulted.	• RCTs published in English<br>• Women >18years either planning a pregnancy or pregnant<br>• Normal weight, overweight or obese.<br>• Studies evaluating any dietary intervention with or without additional advice or support for PA.<br>• Studies reported weight related outcomes, dietary and PA outcomes or outcomes related to the pregnancy, birth or the infant.	• Studies undertaken in non-OECD countries.<br>• Women had underlying medical complications, were pregnant with twins or underweight.	• 5 studies<br>• Published: 2002 to 2010<br>• Study design: RCT n=5<br>• n=577 women	USA n=2<br>Canada n=1<br>Belgium n=1<br>Denmark n=1
Choi *et al.* 2013 [7]	Choi *et al.* 2013 [7]	To review the effectiveness of PA and PA plus diet interventions in managing weight among overweight or obese pregnant or postpartum women	Bibliographic databases: PubMed, EMBASE, CINAHL, and Cochrane Library<br>Supplementary searches: Bibliographies of relevant articles	• Published in English or Korean<br>• Published between 2000 and 2011<br>• RCT<br>• Weight related outcome measure (reports outcomes for PA and combined diet/PA separately)	• postpartum status not clearly stated.<br>• Full text not available<br>• PA interventions not related to weight management	• 7 studies (pregnant women only)<br>• Published: 2002 to 2011<br>• Study design: RCT n=7<br>• n=721 women	USA n=2<br>Australia n=1<br>Brazil n=1<br>Belgium n=1<br>Denmark n=1<br>Spain n=1
Gardner *et al.* 2011 [8]	Gardner *et al.* 2011 [8]	To meta-analyse behaviour-based interventions that have targeted diet and/or PA changes to reduce GWG, and explore moderators of intervention effectiveness	Bibliographic databases: PsycInfo, Medline, Embase, AMED, HMIC, Cochrane Health Technology Assessment<br>Supplementary searches: Cochrane Central Controlled Trials Register.	• Studies reported an evaluation, based on quantitative data<br>• Efficacy of an intervention to improve diet and/or increase PA to prevent excessive GWG<br>• Pregnant women aged 18+ years	• Interventions based on information provision only, or non-psychological interventions<br>• Studies where participants had known pre-pregnancy mental or physical health problems	• 10 papers (reporting 12 trials of 11 interventions)<br>• Published: 2000 to 2010<br>• Study design: RCT n=5; Non RCT n= 2; Time series	USA n=4<br>Canada n=3<br>Denmark n=1<br>Sweden n=1<br>Belgium n=1

Review		Aim of the review	Search strategy	Inclusion criteria	Exclusion criteria	Included studies	Study locations
			Corresponding authors were contacted to provide description of the intervention content.	• Differences between an intervention and a control group on self-reported or objective behaviour or weight gain, measured prior to delivery reported		control trial n=1; Historical cohort n=2<br>• n=1,656 women	
Hill *et al.* 2013 [9]	Hill *et al.* 2013 [9]	To evaluate the overall effectiveness of GWG interventions derived from theories of behaviour change using a generalized health psychology perspective, and assessing the behaviour change techniques reported in the interventions	<u>Bibliographic databases</u>: CINAHL, Global Health, Health Source (nursing/academic), Medline, Psychology and Behavioral Sciences Collection, and PsycINFO<br><u>Supplementary searches</u>: Eligible papers and similar reviews were perused for further relevant studies	• Papers that reported studies in which excessive GWG was the primary or secondary focus of the intervention, and they reported total GWG or adherence to GWG recommendations.<br>• No limits on pre-pregnancy BMI, socioeconomic status, ethnicity, parity, or gestational duration were set	• Studies including adolescent pregnancies<br>• Interventions designed to prevent gestational diabetes were excluded<br>• Interventions focused on smoking cessation and exercise/physical activity for any other purpose than to limit GWG	• 21 studies<br>• Published: 2002-2012<br>• Study design: RCT n=14; Case control n=1; Controlled trial n=2; Prospective intervention n=2; Cohort n=2<br>• n=3823 women	USA n=6<br>Nordic n=6<br>Canada n=3<br>Brazil n=2<br>Belgium n=2<br>Australia n=1<br>Taiwan n=1
Madhuvrata *et al.* 2015 [10]	Madhuvrata *et al.* 2015 [10]	To see if there was any intervention which could be used for primary prevention of GDM in women with risk factors for GDM.	<u>Bibliographic databases</u>: Medline, Embase, conference abstract databases<br><u>Supplementary searches</u>: Cochrane specialised trials register, ClinicalTrials.gov	• RCTs or quasi-RCTs comparing intervention with standard care in women with risk factors for GDM (including raised BMI, previous GDM, previous infant with birth weight >4500 g, family history of diabetes, high-risk ethnic groups, PCOS) (results reported for diet/PA, PA only, diet only separately)	• Trials comparing interventions in pregnant women with no risk factors for GDM and one intervention versus another	• 14 studies<br>• Published: 2002 to 2011<br>• Study design: RCT n=14<br>• n=2,422 women	USA n=3<br>Australia n=3<br>Norway n=2<br>Denmark n=2<br>Holland n=1<br>Belgium n=1<br>Finland n=2
Muktabhant *et al.* 2015 [11]	Muktabhant *et al.* 2015 [11]	To determine whether diet or exercise measures, or both, could prevent excessive GWG, and if they were safe	<u>Bibliographic databases</u>: CENTRAL, MEDLINE, Embase<br><u>Supplementary searches</u>: Cochrane Pregnancy and Childbirth Group's Trials Register. Hand searched 30 journals and conference proceedings; weekly current awareness alerts for a further 44 journals; monthly BioMed Central email alerts; contacted investigators of the previously identified ongoing studies by email to enquire about any new or imminent publications. Reference lists of retrieved studies	• Pregnant women of any BMI<br>• RCTs<br>• Interventions including diet or exercise measures, or both (results reported for diet/PA, PA only, diet only separately)	• Quasi RCT	• 63 studies (plus 2 studies which did not report number of participants)<br>• Published: 1990 to 2014<br>• Study design: RCT n=65<br>• n=>13,523 women (49 RCTs, n=11,444 women included in meta-analysis)	Australia n=10<br>Belgium n=2<br>Canada n=5<br>Denmark n=3<br>Finland n=3<br>Germany n=1<br>Ireland n=1<br>Italy n=2<br>Kosovo n=1<br>Norway n=1<br>Sweden n=2<br>Netherlands n=[illegible]<br>Spain n=3<br>UK n=1<br>USA n=20<br>Brazil n=4<br>Columbia n=1<br>Taiwan n=1
O'Brien *et al.* 2015 [12]	O'Brien *et al.* 2015 [12]	To identify the effect of providing an antenatal dietary and lifestyle intervention for women of normal BMI on maternal and infant outcomes	<u>Bibliographic databases</u>: PubMed, Medline<br><u>Supplementary searches</u>: Cochrane Controlled Trials Register, Australian and International Clinical Trials Registry	• Studies of women with a normal BMI<br>• Singleton pregnancy<br>• Provision of a dietary and/or lifestyle intervention compared with standard antenatal care or no intervention	• Quasi-randomized trials<br>• Abstract form only<br>• A specific group of women at risk of GDM or LGA infants	• 12 studies<br>• Published: 2002 to 2014<br>• Study design: RCT n=12<br>• n=2,713 women	USA n=4<br>Canada n=3<br>Finland n=1<br>Netherlands n=[illegible]<br>Sweden n=1<br>Australia n=1<br>Taiwan n=1
Oteng-Ntim *et al.* 2012 [13]	Oteng-Ntim *et al.* 2012 [13]	To determine the efficacy of combined dietary activity and behaviour support interventions in overweight and obese pregnant women (to improve pregnancy outcome)	<u>Bibliographic databases</u>: Cochrane Library, MEDLINE, EMBASE, CINAHL, Maternity and Infant care, PsycINFO, Science Citation Index, Social Science Citation Index, Global Health Popline, Medcarib, Nutrition database	• RCTs and non-RCTs that evaluated antenatal diet/lifestyle interventions<br>• Obese/overweight pregnant women<br>• Quantitative maternal and fetal health outcomes.	• Systematic reviews<br>• Trials of women with existing GDM or PCOS<br>• Trials of pre-conception or postpartum interventions	• 19 studies<br>• Published: 2000 to 2011<br>• Study design: RCT n=13; Non-RCT n= 6<br>• n=2,762 women	USA n=5<br>Canada n=3<br>Australia n=3<br>Finland n=2<br>Denmark n=1<br>Netherlands n=[illegible]

Review		Aim of the review	Search strategy	Inclusion criteria	Exclusion criteria	Included studies	Study locations
			Supplementary searches: None reported		• Trials where the pregnant women were not overweight or obese		Sweden n=1<br>Spain n=1<br>Brazil n=1<br>Belgium n=1
Rogozińska *et al.* 2015 [14]	Rogozińska *et al.* 2015 [14]	To assess the effects of nutritional manipulation in pregnancy on GDM and relevant maternal and fetal outcomes.	Bibliographic databases: Medline, Embase, Cochrane Database<br>Supplementary searches: reference lists of the included studies.	• Randomised studies<br>• Nutritional interventions in pregnancy<br>• Diet based advice, combination of diet and PA, nutritional supplements (reports outcomes for diet and combined diet/PA separately)	• Studies that evaluated only PA	• 20 studies<br>• Published: 2002 to 2014<br>• Study design: RCT n=20<br>• n=6,444 women	Not reported
Tanentsapf *et al.* 2011 [15]	Tanentsapf *et al.* 2011 [16]	To evaluate the effect of dietary interventions for reducing GWG. To examine the impact of these interventions on different child and maternal health outcomes.	Bibliographic databases: PUBMED, LILACS.<br>Supplementary searches: the Cochrane Central Register of Controlled Trials (CENTRAL), hand searching	• RCTs and QCTs with a concurrent control group<br>• Healthy normal weight, overweight, obese women with singleton pregnancy<br>• Dietary intervention for GWG or reducing pregnancy complications (some included PA elements)	• Women under the age of 18 years<br>• Medication that might interfere with weight<br>• Underweight or with increased risk of insufficient weight gain/LBW<br>• Interventions not designed to prevent excessive GWG	• 13 studies<br>• Published: 1975 to 2011<br>• Study design: RCT n= 10; QRT n= 3<br>• n=1,870 women	USA n=4<br>Egypt n=1<br>Taiwan n=1<br>Scotland n=2<br>Belgium n=1<br>Canada n=1<br>Finland n=2<br>Denmark n=1
Thangaratinam *et al.* 2012a [16]	Thangaratinam *et al.* 2012a [17]	To evaluate the effectiveness of dietary and lifestyle interventions in reducing or preventing obesity in pregnancy and to assess the beneficial and adverse effects of the interventions on obstetric, fetal and neonatal outcomes.	Bibliographic databases: MEDLINE, EMBASE,<br>BIOSIS, Latin American and Caribbean Health Sciences Literature (LILACS), Science Citation Index, Cochrane Database of Systematic Reviews, CENTRAL, DARE, HTA database, PsycINFO, Inside Conferences, Systems for Information in Grey Literature (SIGLE), Dissertation Abstracts<br>Supplementary searches: ClinicalTrials.gov. Internet specialist gateways (e.g. OMNI), general search engines (e.g. Google) and meta-search engines (e.g. Copernic)	• Normal weight, overweight or obese<br>• Any setting.<br>• Dietary, PA and behavioural change intervention<br>• Comparison: women with no intervention or routine antenatal care<br>• RCTs or non randomised studies and observational studies when the evidence from RCTs was insufficient.<br>• Maternal and fetal outcomes.	• Underweight<br>• No data to estimate effectiveness measures	• 88 studies<br>• Published: 1975 to 2010<br>• Study design: RCT n= 40; Non-RCT n=16; Cohort n=26; Case-control n=6<br>• n=182,139 women	Not reported
Thangaratinam *et al.* 2012b [17]	Thangaratinam *et al.* 2012b [18]	To evaluate the effects of dietary and lifestyle interventions in pregnancy on maternal and fetal weight and to quantify the effects of these interventions on obstetric outcomes	Bibliographic databases: Medline, Embase, BIOSIS, LILACS, Science Citation Index, Cochrane Database of Systematic Reviews, Database of Abstracts of Reviews of Effects, Health Technology Assessment Database, PsychInfo, Inside Conferences, Systems for Information in Grey Literature (SIGLE), Dissertation Abstracts, and<br>Supplementary searches: Internet searches using specialist search gateways (such as OMNI), general search engines (such as Google) and meta-search engines (such as Copernic). Cochrane Central Register of Controlled Trials, Clinical Trials.gov.	• RCTs<br>• Evaluated any dietary or lifestyle interventions with potential to influence maternal and fetal outcomes related to weight (reported results for diet/PA, diet only and PA only separately)	• Underweight	• 44 studies<br>• Published: 1976 to 2011<br>• Study design: RCT n=44<br>• n=7,278 women	Not reported

Review		Aim of the review	Search strategy	Inclusion criteria	Exclusion criteria	Included studies	Study locations
IWiP 2017 [18]	IWiP 2017 [19]	To synthesise the evidence on the overall and differential effects of interventions based on diet and PA during pregnancy, primarily on GWG and maternal and offspring composite outcomes.	Bibliographic databases: Medline, Embase, Cochrane Database of Systematic Reviews, Database of Abstracts of Reviews of Effects, Health Technology Assessment<br>Supplementary searches: Cochrane Central Register of Controlled Trials	● RCTs<br>● Assessed the effects of interventions based on diet, PA, and mixed interventions in pregnancy, on maternal and offspring outcomes (reported results for diet/PA, diet only and PA only separately)	● Studies that only included women with GDM<br>● Involved animals<br>● Reported only non-clinical outcomes<br>● Published before 1990.	● 36 studies<br>● Published: 1993 to 2017<br>● Study design: RCT n= 36<br>● n=12,526 women	Europe n=22<br>North America<br>Australia n=4<br>Brazil n=4<br>Egypt n=1<br>Iran n=1
Lau *et al.* 2017 [19]	Lau *et al.* 2017 [20]	To synthesize the best evidence to assess the effectiveness of e-based lifestyle interventions in improving maternal and neonatal outcomes among perinatal overweight or obese women.	Bibliographic databases: CINAHL, Cochrane Library, EMBASE, ProQuest Dissertations and Theses, PsycINFO, PubMed and Scopus<br>Supplementary searches: clinical trial registries, reference lists of the eligible studies and systematic reviews	● Overweight and/or obese<br>● From pregnancy to 1 year postpartum<br>● E-based lifestyle interventions comprising at least one component of dietary control, PA and weight management (website, Internet, Apps, SMS, email, computer or video)<br>● Outcomes: GWG, postnatal weight change, obstetric complications and neonatal outcomes	● Studies among the general population<br>● Pregnant women with comorbidities.<br>● Studies that had no lifestyle components or e-based elements in the intervention group.<br>● CCTs, non-experimental, abstracts, qualitative designs, protocols, reviews or conference papers.	● 14 studies<br>● Published: 2006 to 2016<br>● Study design: RCT n= 14<br>● n=3,169 women	USA n=9<br>Australia n=2<br>Sweden n=1<br>UK n=1<br>Canada n=1
Magro-Malosso *et al* 2017a [20]	Magro-Malosso *et al* 2017a [21]	To evaluate the effect of exercise during pregnancy on the risk of gestational hypertensive disorders.	Bibliographic databases: MEDLINE, Embase, Web of Sciences, Scopus, OVID and Cochrane Library<br>Supplementary searches: ClinicalTrials.gov	● RCTs of pregnant women randomized to an exercise regimen or not.<br>● Singleton pregnancies without contraindication to PA reporting data on gestational hypertensive disorders.	● Diet counselling, weight monitoring, exercise reduction<br>● Trials which included in at-risk populations (e.g. all smokers)<br>● Quasi-RCTs	● 17 studies<br>● Published: 2009 to 2016<br>● Study design: RCT n= 17<br>● n=5,075 women	Spain n=9<br>Norway n=2<br>USA n=2<br>Denmark n=2<br>Brazil n=1<br>Italy n=1
Magro-Malosso *et al* 2017b [21]	Magro-Malosso *et al* 2017b [22]	To evaluate the effect of exercise on the risk of PTB in overweight or obese pregnant women.	Bibliographic databases: MEDLINE, EMBASE, Web of Sciences, Scopus, OVID and Cochrane Library<br>Supplementary searches: ClinicalTrial.gov	● RCTs with overweight or obese pregnant women randomized to an exercise regimen or not<br>● Singleton pregnancy without contraindication to PA reporting PTB	● RCTs in women with BMI≤24.9<br>● Diet counselling, weight monitoring, exercise reduction<br>● Trials which included in at-risk populations (e.g. all smokers)<br>● Quasi-RCTs	● 9 studies<br>● Published: 2005 to 2016<br>● Study design: RCT n=17<br>● n=1,502 women	Brazil: n=2<br>Denmark n=2<br>USA n=2<br>Spain n= 2<br>Italy n=1
Shepherd *et al.* 2017 [22]	Shepherd *et al.* 2017 [23]	To assess the effects of diet interventions in combination with exercise interventions for pregnant women for preventing GDM and associated adverse health consequences for the mother and her infant/child.	Bibliographic databases: MED- LINE, Embase and CINAHL.<br>Supplementary searches: CENTRAL, Cochrane Pregnancy and Childbirth's Trials Register. Hand searched journals and conference proceedings, journals reviewed via the current awareness service, reference lists of retrieved trials.	● RCTs (incl. cluster and abstract only) assessing combined diet and exercise interventions for preventing GDM.<br>● Pregnant women regardless of age, gestation, parity or plurality.<br>● Interventions that incorporated any type of diet intervention with any type of exercise intervention.	● Quasi-RCTs.<br>● Cross-over trials<br>● Trials involving women with pre-existing GDM, type 1 or type 2 diabetes.	● 23 studies<br>● Published: 2002 to 2017<br>● Study design: cluster RCT n=2, Individually RCT n=21<br>● n=8,918 women	USA n=5<br>Finland n=3<br>Australia n=2<br>UK n=2<br>Canada n=2<br>Italy n=2<br>China n=2<br>Brazil n=1<br>Denmark n=1<br>Egypt n=1<br>Germany n=1<br>Norway n=1
Sherifali *et al.* 2017 [23]	Sherifali *et al.* 2017 [24]	To assess the effectiveness of eHealth technologies for managing weight (loss, gain, or maintenance) during pregnancy and the postpartum period. 2. To assess the effectiveness of eHealth technologies on other clinical outcomes.	Bibliographic databases: MEDLINE, EMBASE, Cochrane database of systematic reviews (CDSR), CINAHL, and PsycINFO.<br>Supplementary searches: Cochrane central, register of controlled trials (CENTRAL), reference lists and paper citations.	● RCTs, CCTs, pre-post studies, historically controlled studies, pilot studies.<br>● Women ≥18 years during pregnancy or the postpartum period.<br>● Intervention included nutrition or PA in the eHealth technology (mobile phone, Web-based, email, personal digital assistant, handheld computer, home computer, or tablet app) with a specific goal of targeting either GWG during pregnancy or weight loss during the	● All study protocols without preliminary results for data extraction.<br>● All other study designs that were not part of the inclusion criteria.	● 6 studies (only including interventions during pregnancy)<br>● Published: 2010 to 2016<br>● Study design: RCTs n=5, CCTs n=1<br>● n=363 women	UK n=1<br>Spain n=2<br>USA n= 3

Review		Aim of the review	Search strategy	Inclusion criteria	Exclusion criteria	Included studies	Study locations
				postpartum period.<br>• Min. intervention duration 3 months.			
Song *et al.* 2016 [24]	Song *et al.* 2016 [25]	To address the efficacy of lifestyle intervention during pregnancy, i.e. diet and physical activity, on the risk of GDM.	<u>Bibliographic databases</u>: PubMed, Cochrane Library, Web of Science, Springer Link, JAMA, Ovid, Sino Med, Wiley Online Library, Science Direct Embase, Clinicaltrials.gov, Lancet, Nature, Science, The New England Journal of Medicine, BioMedNet and Google Scholar) .<br><u>Supplementary searches</u>: manual searches of the reference lists of included studies and relevant reviews.	• RCTs that only evaluated lifestyle interventions during the first two trimesters of pregnancy with an outcome measure of GDM<br>• Studies published in English and Chinese	• Studies involving women with pre-existing type 1 or type 2 diabetes or having existing GDM.<br>• Studies on interventions with only nutrient supplements<br>• Systematic reviews, meta-analysis, observational studies, study protocol and pilot study	• 29 studies<br>• Published: 2002 to 2015<br>• Study design: RCTs n=29<br>• n=11,487 women	USA n=6<br>UK n=1<br>Finland n=3<br>Australia n=3<br>Ireland n=1<br>Italy n=1<br>Denmark n=3<br>Spain n=5<br>Canada n=2<br>Belgium n=1<br>Norway n=1<br>Netherlands n=[illegible]<br>India n=1
Yeo *et al.* 2017 [25]	Yeo *et al.* 2017 [26]	To (i) review randomized clinical trials targeting GWG in obese and overweight women by implementing prenatal lifestyle interventions; (ii) describe the study characteristics (demographics, types of interventions, intervention delivery settings and methodological qualities); and (iii) identify which study characteristics influence efficacy of interventions.	<u>Bibliographic databases</u>: MEDLINE (PubMed), EMBASE (Elsevier) and CINAHL(Ebsco).<br><u>Supplementary searches.</u> Authors contacted via email when necessary. No other supplementary searches noted.	• RCT's<br>• Study populations including pregnant subjects that were either over-weight (body mass index [BMI]≥25 kg m2) or obese (BMI≥30 kg m2)<br>• Interventions initiated before the third trimester<br>• GWG, either reported or easily derived from the available data.	• Studies not analysing or reporting GWG	• 32 studies<br>• Published: 2005 to 2016<br>• Study design: RCTs n=32<br>• n=5,857 women	Netherlands n=[illegible]<br>USA n=9<br>Spain n=2<br>Belgium n=2<br>Australia n=8<br>Norway n=1<br>Canada n=1<br>Finland n=1<br>Brazil n=2<br>Italy n=1<br>Denmark n=3<br>New Zealand n=[illegible]
Craemer *et al.* 2019 [26]	Craemer *et al.* 2019 [27]	To evaluate nutrition-only, exercise-only, and nutrition-plus-exercise interventions for optimizing GWG based on the 2009 IOM guidelines.	<u>Bibliographic databases</u>: PubMed, Google Scholar<br><u>Supplementary searches</u>: All studies included in 2015 Cochrane Review (Muktabhant *et al.* 2015 [11]) were examined.	• RCTs (parallel or cross sectional) (results reported separately for diet/PA, diet only and PA only)<br>• At least 20 singleton pregnant women<br>• Women aged 18 years or older<br>• Control group standard obstetric care<br>• Report means of GWG based on baseline BMI or pre-pregnancy BMI<br>• Use 2009 IOM guidelines for GWG. For studies that did not use these, additional analysis was made based on the reported mean GWG	• Studies published before 2009<br>• Mean GWG not reported<br>• Study in trial state	• 31 studies<br>• Published: 2009 to 2016<br>• Study design: RCT n=31<br>• n=8,558 women	Netherlands n=[illegible]<br>USA n=4<br>Turkey n=1<br>Belgium n=1<br>Australia and N[illegible]<br>Zealand n=3<br>Italy n=2<br>Norway n=2<br>Ireland n=3<br>Taiwan n=1<br>Canada n=3<br>Finland n=3<br>China n=1<br>Brazil n=1<br>UK n=1<br>Germany n=1<br>Denmark n=2<br>Spain n=1
Davenport *et al.* 2019a [27]	Davenport *et al.* 2019a [28]	The aim of the study is to perform a systematic review of the relationship between prenatal exercise and fetal or new-born death.	<u>Bibliographic databases</u>: MEDLINE, EMBASE, PsycINFO, Cochrane Database of Systematic Reviews, Cochrane Central Register of Controlled Trials, Scopus and Web of Science Core Collection, CINAHL Plus with Full-text, Child Development and Adolescent Studies, ERIC, Sport	• Pregnant women without contraindication to exercise<br>• Objective or subjective measures of frequency, intensity, duration, volume or type of exercise.<br>• Interventions including exercise alone or in combination with other interventions were considered.	• Women with absolute or relative contraindications to exercise.<br>• Exercise began after the initiation of labour.<br>• Case studies and not original research (narrative or systematic reviews and meta-analyses).	• 46 studies<br>• Published: 1976 to 2016<br>• Study design: RCT n=30, n-RCT n=8 (4 cohort, 1 cross-sectional, 3 case-control studies)<br>• n=266,778 women	Finland n=4<br>UK n= 3<br>USA n=10<br>Denmark n=3<br>India n=2<br>Canada n= 2<br>Japan n= 2<br>Germany n=1

Review		Aim of the review	Search strategy	Inclusion criteria	Exclusion criteria	Included studies	Study location[illegible]
			Discus, ClinicalTrials.gov and the Trip Database.<br><u>Supplementary searches</u>: None reported.	• Eligible comparators no exercise or different frequencies, intensities, durations, volumes and types of exercise or exercise in different trimesters.<br>• Outcomes were miscarriage and perinatal mortality and infant mortality.	• Studies not published in either English, Spanish or French.		China n= 3<br>Sweden n= 5<br>Australia n= 2<br>Belgium n=1<br>South Africa n=[illegible]<br>Norway n = 3<br>Benin n=1<br>New Zealand n[illegible]<br>Brazil n=1<br>Greece n=1
Davenport *et al.* 2018a [28]	Davenport *et al.* 2018a [29]	To examine the influence of prenatal exercise on depression and anxiety during pregnancy and the postpartum period.	<u>Bibliographic databases</u>: MEDLINE, EMBASE, PsycINFO, Cochrane Database of Systematic Reviews, Scopus and Web of Science Core Collection, CINAHL Plus with Full-text, Child Development & Adolescent Studies, ERIC, Sport Discus, the Trip Database<br><u>Supplementary searches</u>: Cochrane Central Register of Controlled Trials, ClinicalTrials.gov	• Pregnant women without contraindication to exercise.<br>• Objective or subjective measures of frequency, intensity, duration, volume or type of exercise.<br>• Acute (ie, a single exercise session) or habitual (ie, usual activity) prenatal exercise and interventions including exercise alone or in combination with other interventions (reports outcomes for PA and combined diet/PA separately).<br>• Outcomes were diagnosis of depression or anxiety and severity of symptoms of depression or anxiety.	• Women with absolute or relative contraindications to exercise.<br>• Exercise began after the initiation of labour.<br>• Case studies and not original research (narrative or systematic reviews and meta-analyses).<br>• Studies not published in either English, Spanish or French.	• 52 studies<br>• Published: 2008 to 2016<br>• Study design: RCTs n=26, non-RCT n=7, cohort n=10, cross-sectional n=6, case-control n=3<br>• n=131,406 women	USA n=18<br>Canada n=3<br>Sweden n=1<br>India n=2<br>Multi - Europea[illegible] countries n= 1<br>Australia n=3<br>Egypt n=1<br>Norway n= 4<br>Poland n=2<br>Iran n=2<br>Finland n=1<br>Brazil n=1<br>UK n=3<br>Spain n=3<br>Serbia n=1<br>Colombia n=1<br>South Korea n=[illegible]<br>Denmark n=1<br>Japan n=1<br>Portugal n= 1
Davenport *et al.* 2018b [29]	Davenport *et al.* 2018b [30]	To perform a systematic review and meta-analysis of the relationships between prenatal exercise and GDM, GH and PE.	<u>Bibliographic databases</u>: MEDLINE, EMBASE, PsycINFO, Cochrane Database of Systematic Reviews, Scopus and Web of Science Core Collection, CINAHL Plus with Full-text, Child Development & Adolescent Studies, ERIC, Sport Discus, the Trip Database<br><u>Supplementary searches</u>: Cochrane Central Register of Controlled Trials, ClinicalTrials.gov	• Pregnant women without contraindication to exercise.<br>• Objective or subjective measures of frequency, intensity, duration, volume or type of exercise (reports outcomes for PA and combined diet/PA separately).<br>• Relevant outcomes were GDM, GH and PE.	• Women with absolute or relative contraindications to exercise.<br>• Exercise began after the initiation of labour.<br>• Case studies and not original research (narrative or systematic reviews and meta-analyses).<br>• Studies not published in either English, Spanish or French.	• 106 studies<br>• Published: 1974 to 2017<br>• Study design: RCTs n=65, non-RCTs n=9, cohort n=13, cross-sectional n=11, case-control n=8<br>• n=273,182 women	USA n=32<br>Spain n=11<br>Canada n=11<br>Norway n=6<br>China n=6<br>UK n=3<br>Australia n=5<br>Denmark n=3<br>Finland n=4<br>Netherlands n=[illegible]<br>Italy n=1<br>Iran n=3<br>Brazil n=3<br>Belgium n=1<br>Sweden n=3<br>New Zealand n[illegible]<br>Thailand n=1<br>India n=1<br>Kosovo n=1<br>Germany n=1<br>Croatia n=1<br>Pakistan n=1

Review		Aim of the review	Search strategy	Inclusion criteria	Exclusion criteria	Included studies	Study location
							Greece n=1<br>Japan n=1<br>Multiple Europ<br>Countries n=2
Davenport *et al.* 2019b [30]	Davenport *et al.* 2019b [31]	To assess the associations between prenatal PA (in terms of frequency, intensity, type and volume of PA), with maternal harms, labour and delivery outcomes.	Bibliographic databases: MEDLINE, EMBASE, PsycINFO, Cochrane Database of Systematic Reviews, Scopus and Web of Science Core Collection, CINAHL Plus with Full-text, Child Development & Adolescent Studies, ERIC, Sport Discus, the Trip Database<br>Supplementary searches: Cochrane Central Register of Controlled Trials, ClinicalTrials.gov.	• Pregnant women without contraindication to exercise.<br>• Objective or subjective measures of frequency, intensity, duration, volume or type of exercise (reports outcomes for PA and combined diet/PA separately).<br>• Outcomes were PTB/prelabour rupture of membranes, caesarean section, instrumental delivery, induction of labour, length of labour, vaginal tears, fatigue, injury, musculoskeletal trauma, maternal harms (author defined) and diastasis recti.	• Women with absolute or relative contraindications to exercise.<br>• Exercise began after the initiation of labour.<br>• Case studies and not original research (narrative or systematic reviews and meta-analyses).<br>• Studies not published in either English, Spanish or French	• 113 studies<br>• Published: 1974 to 2017<br>• Study design: RCTs n=79, non-RCTs n=12, observational n=22<br>• n=52,858 women	USA n=32<br>Spain n=12<br>Australia n=7<br>Brazil n=7<br>Norway n=7<br>UK n=6<br>Canada n=5<br>Thailand n=4<br>Sweden n=4<br>Netherlands n=<br>Denmark n=3<br>China n=3<br>India n=1<br>Poland n=2<br>Japan n=2<br>Italy n=2<br>Benin n=1<br>Belgium n=1<br>Switzerland n=<br>Colombia n=1<br>Iran n=1<br>Germany n=1<br>Croatia n=1<br>Finland n=1<br>Mexico n=1<br>Turkey n=1<br>France n=1<br>New Zealand n=
Davenport *et al.* 2018c [31]	Davenport *et al.* 2018c [32]	To perform a systematic review and meta-analysis to explore the relationship between prenatal exercise and glycaemic control.	Bibliographic databases: MEDLINE, EMBASE, PsycINFO, Cochrane Database of Systematic Reviews, Scopus and Web of Science Core Collection, CINAHL Plus with Full-text, Child Development & Adolescent Studies, ERIC, Sport Discus, the Trip Database<br>Supplementary searches: Cochrane Central Register of Controlled Trials, ClinicalTrials.gov	• Pregnant women without contraindication to exercise.<br>• Objective or subjective measures of frequency, intensity, duration, volume or type of exercise.<br>• Original studies of any design (RCT and observational)<br>• Outcomes were indicators of glucose tolerance	• Women with absolute or relative contraindications to exercise.<br>• Exercise began after the initiation of labour.<br>• Case studies and not original research (narrative or systematic reviews and meta-analyses).<br>• Studies not published in either English, Spanish or French	• 58 studies<br>• Published: 1981 to 2017<br>• Study design: RCTs n=31, non-RCTs n=7, cohort n=8, cross-sectional n=12<br>• n=8,699 women	Not reported
Michel S *et al.* 2019 [32]	Michel S *et al.* 2019 [33]	To examine if lifestyle interventions during pregnancy have the potential to reduce weight retention at 4 months postpartum and beyond	Bibliographic databases: PubMed, EMBASE, CENTRAL, Web of Science, and the Global Health database Global Index Medicus.<br>Supplementary searches:<br>Reference lists of included studies were screened	• RCTs and cluster RCTs with a minimum of 100 participants<br>• Pregnant women of all ages, with any pre-pregnancy body mass index (BMI) and singleton pregnancies.<br>• All types of lifestyle or behavioural interventions related to weight control in pregnancy<br>• Interventions of any length as long as they commenced in pregnancy	• Studies focusing on women with severe diseases or serious conditions<br>• Studies with a follow-up period shorter than 4 months past delivery<br>• Articles not published in English or German language	• 14 studies<br>• Published: 2011 to 2017<br>• Study design: RCT n=11, cluster RCT n=3<br>• n=7,116 women	Ireland n=1<br>Taiwan n=1<br>USA n=4<br>Finland n=1<br>UK n=2<br>Germany n=1<br>Sweden n=1<br>Norway n=1<br>Denmark n=1<br>Netherlands n=

Review		Aim of the review	Search strategy	Inclusion criteria	Exclusion criteria	Included studies	Study locations
				• Required outcome measures were pre-pregnancy or early pregnancy weight and postpartum weight, or PPWR, at a minimum of 4 months postpartum.			
Morison *et al.* 2018 [33]	Morison *et al.* 2018 [34]	To assess whether an intensive diet and exercise intervention has an effect in reducing GWG and LGA newborns.	Bibliographic databases: PUBMED, COCHRANE library<br>Supplementary searches: Reference lists of the retrieved studies.	• RCTs in pregnant women<br>• Diet and exercise<br>• Intervention lasted more than 6 weeks	• Nonrandomized, quasi-RCTs.<br>• Lacked the outcomes evaluated.<br>• Pilot studies.	• 10 studies<br>• Published: 2011 to 2016<br>• Study design: RCT n=10<br>• n=6,164 women	Canada n=3<br>Brazil n=1<br>Norway n=1<br>Netherlands n=[illegible]<br>Spain n=1<br>UK n=1<br>Australia n=1<br>USA n=1
Ruchat *et al.* 2018 [34]	Ruchat *et al.* 2018 [35]	The purpose of this review was to evaluate the effect of prenatal exercise on GWG and PPWR.	Bibliographic databases: MEDLINE, EMBASE, PsycINFO, Cochrane Database of Systematic Reviews, Scopus and Web of Science Core Collection, CINAHL Plus with Full-text, Child Development & Adolescent Studies, ERIC, Sport Discus, and the Trip Database<br>Supplementary searches: Cochrane Central Register of Controlled Trials, ClinicalTrials. gov	• Primary studies of any design except case studies, narrative syntheses and systematic reviews.<br>• Exercise only and exercise + co-intervention (results reported separately for diet/PA and PA only)	• Case studies<br>• Narrative syntheses<br>• Systematic reviews<br>• Published in language other than English, Spanish or French	• 84 studies<br>• Published: 1987 to 2017<br>• Study design: RCT n=79, non-randomised intervention studies n=4, cohort study n=1<br>• n=21,530 women	USA n=19<br>Sweden n=3<br>Canada n=9<br>Netherland n=2[illegible]<br>Spain n=10<br>Italy n=2<br>Brazil n=5<br>Mexico n=3<br>Australia n=6<br>Norway n=3<br>Iran n=2<br>Belgium n=1<br>New Zealand n[illegible]<br>Taiwan n=1<br>China n=3<br>Finland n=3<br>UK n=2<br>Germany n=1<br>Denmark n=2<br>India n=1<br>Iran n=1<br>Thailand n=1<br>Japan n=1<br>Croatia n=1
Syngelaki *et al.* 2019 [35]	Syngelaki *et al.* 2019 [36]	To examine whether diet and/or exercise can prevent PE in overweight or obese pregnant women.	Bibliographic databases: PubMed, Embase, CINAHL, Web of Science<br>Supplementary searches: Cochrane CENTRAL, references, other reviews	• RCTs that evaluated the effect of diet and/or exercise on the risk of PE and hypertensive disorders of pregnancy (results reported separately for diet/PA, diet only and exercise only)<br>• Overweight/ obese women	• Not reported	• 23 studies<br>• Published: 2000 to 2017<br>• Study design: RCT n=23<br>• n=7,236 women	Not reported
Walker *et al.* 2018 [36]	Walker *et al.* 2018 [37]	To address compare the efficacy of all approaches that have been used to prevent excessive GWG (e.g., diet, PA, lifestyle, sleep, eHealth, and medical)	Bibliographic databases: MEDLINE, EMBASE, Cochrane Library, PsycInFO, CINAHL, Scopus, LILACS<br>Supplementary searches: reference lists of included trials and systematic reviews	• RCTs of interventions designed to prevent excessive GWG.<br>• Comparators were standard care, an alternate intervention, or placebo.<br>• Participants were women of all ages, ethnicities, and pre-pregnancy weight status with singleton pregnancies.	• Studies for women with pre-existing diabetes (type 1 or type 2) or GDM	• 89 studies<br>• Published: 2000 to 2018<br>• Study design: RCT n=89<br>• n=25,345 women	USA n=22<br>Australia n=11<br>Spain n=10<br>Italy n=5<br>Finland n=3<br>Egypt n=1<br>Turkey n=2<br>Sweden n=2<br>Brazil n=4<br>Ireland n=2<br>Denmark n=3

Review		Aim of the review	Search strategy	Inclusion criteria	Exclusion criteria	Included studies	Study location[illegible]
							Argentina n=1<br>Canada n=4<br>Norway n=3<br>Iran n=1<br>Netherlands n=[illegible]<br>China n=3<br>Belgium n=2<br>Taiwan n=1<br>India n=1<br>UK n=3<br>Columbia n=1<br>Germany n=1<br>Multiple Europe[illegible] countries n=1
Vincze *et al.* 2019 [37]	Vincze *et al.* 2019 [38]	To evaluate the effectiveness of weight management interventions which included a diet component aimed at limiting gestational weight gain.	<u>Bibliographic databases</u>: MEDLINE, Embase, CINAHL, Scopus, PsycINFO<br><u>Supplementary searches</u>: Cochrane Central, reference lists	• Pregnant women aged 18 years or over<br>• All pre-pregnancy BMI categories.<br>• Participants had GDM or diabetes<br>• Intervention with dietary component aimed at managing GWG, compared to usual care or ‘‘other’’<br>• Weight related primary outcome measured during pregnancy<br>• RCTs published between 1980 and January 21, 2016.	• Any other medical conditions<br>• Unpublished results	• 20 studies<br>• Published: 2002 to 2016<br>• Study design: RCT n=20<br>• n=5,895 women	USA n=6<br>Australia n=2<br>Canada n=2<br>Denmark n=3<br>Finland n=1<br>Italy n=2<br>Sweden n=1<br>Turkey n=1<br>Belgium n=1<br>Germany n=1
Shieh *et al.* 2018 [38]	Shieh *et al.* 2018 [39]	To analyse data from published studies using a RCT design on the prevention of excessive GWG specifically among pregnant overweight and obese women.	<u>Bibliographic databases</u>: PubMed, Academic Search Premier, CINAHL, MEDLINE, and PsyInfo<br><u>Supplementary searches</u>: None reported	• RCT<br>• Exercise, diet or both as the intervention<br>• Healthy overweight and/or obese pregnant adult women<br>• GWG an outcome	• Sub-study from RCTs<br>• Systematic reviews<br>• Meta-analyses<br>• Interventions to increase pregnancy weight gain<br>• Women with an identified health problem (e.g. AIDS, GDM).	• 23 studies<br>• Published: 2008 to 2016<br>• Study design: RCT n=23<br>• n=6,920 women	Belgium n=2<br>Denmark n=3<br>Norway n=1<br>UK n=1<br>Italy n=1<br>Australia n=6<br>USA n=5<br>New Zealand n[illegible]<br>Canada n=1<br>Brazil n=1<br>Multiple Europe[illegible] countries n=1
Bennett *et al.* 2017 [39]	Bennett *et al.* 2017 [40]	To comprehensively evaluate the global impact of interventions designed to prevent excessive GWG on the incidence of GDM, and whether the effects differ by maternal BMI or ethnicity.	<u>Bibliographic databases</u>: Cochrane Library, MEDLINE, EMBASE, PsycINFO, CINAHL, Scopus, LILACS, CNKI, WangFang, VIP<br><u>Supplementary searches</u>: reference lists of systematic reviews	• RCTs conducted in humans<br>• Primary or secondary aim to reduce/prevent excessive GWG<br>• Reported on the incidence of GDM in both groups separately<br>• All languages and countries	• Studies in animals<br>• Studies conducted in women with existing<br>• Studies to encourage GWG<br>• Duplication populations	• 45 studies<br>• Published: 2002 to 2016<br>• Study design: RCT n=45<br>• n=15,293 women	Australia n=8<br>China n=8<br>USA n=7<br>Spain n=6<br>Canada n=3<br>Denmark n=3<br>Finland n=2<br>UK n=2<br>Ireland n=1<br>Netherlands n=[illegible]<br>Belgium n=1<br>Italy n=1<br>Norway n=1<br>Multiple Europe[illegible] countries n=1

S5c: Systematic reviews reporting outcomes for diet only interventions

Review	Aim of the review	Search strategy	Inclusion criteria	Exclusion criteria	Included studies	Study locations
Allen *et al.* 2014 [5]	To evaluate the effect of dietary and lifestyle interventions with the potential to modify metabolic risk factors on the risk of PE.	Bibliographic databases: MEDLINE, EMBASE and Cochrane<br>Supplementary searches: Reference lists of all primary and review articles.	● RCTs in pregnant women evaluating the effect of dietary and lifestyle interventions with the potential to modify metabolic risks such as obesity, hyperlipidemia/glycemia and hypertension on the risk of PE (results reported for diet/PA and diet only separately)	● Non-randomized<br>● Animal studies	● 18 studies<br>● Published: 1992 to 2012<br>● Study design: RCT n= 18<br>● n=8,712 women	Not reported
Gresham *et al.* 2016 [40]	To investigate if dietary interventions have any effect on pregnancy outcome.	Bibliographic databases: EMBASE; Pre-Medline; MEDLINE; Proquest; Web of Science; CINAHL; Scopus; The Cochrane Library; Mosby Index; and Maternity and Infant Care.<br>Supplementary searches: reference lists of included papers.	● Studies that reported any pregnancy, neonatal or infant outcomes<br>● Any age, weight or BMI<br>● No date limits.<br>● Any healthy, human population.<br>● Randomised or pseudo-RCTs on dietary interventions.<br>● Any intensity, frequency or timing of intervention.<br>● Positive or neutral quality.	● Health conditions that may influence dietary intake.<br>● Languages other than English.<br>● Studies in animals.<br>● Case study, editorial, conference proceedings<br>● Studies on GWG or nutrient supplementation (i.e. tablet form).<br>● Multiple births.<br>● Duplicate populations<br>● Negative methodological quality.	● 14 studies<br>● Published: 1989 to 2011<br>● Study design: RCT n=14<br>● n=9,756 women	USA n=5<br>Australia n=1<br>Denmark n=2<br>Greece n=1<br>Italy n=1<br>Finland n=1<br>Netherlands n=2<br>Norway n=1
Madhuvrata *et al.* 2015 [10]	To see if there was any intervention which could be used for primary prevention of GDM in women with risk factors for GDM.	Bibliographic databases: Medline, Embase, conference abstract databases<br>Supplementary searches: Cochrane specialised trials register, ClinicalTrials.gov	● RCTs or quasi-RCTs comparing intervention with standard care in women with risk factors for GDM (including raised BMI, previous GDM, previous infant with birth weight >4500 g, family history of diabetes, high-risk ethnic groups, PCOS)<br>● (Results reported for diet/PA, PA only, diet only separately)	● Trials comparing interventions in pregnant women with no risk factors for GDM and one intervention versus another	● 14 studies<br>● Published: 2002 to 2011<br>● Study design: RCT n=14<br>● n=2,422 women	USA n=3<br>Australia n=3<br>Norway n=2<br>Denmark n=2<br>Holland n=1<br>Belgium n=1<br>Finland n=2
Muktabhant *et al.* 2015 [11]	To determine whether diet or exercise measures, or both, could prevent excessive GWG, and if they were safe.	Bibliographic databases: CENTRAL, MEDLINE, Embase<br>Supplementary searches: Cochrane Pregnancy and Childbirth Group's Trials Register. Hand searched 30 journals and conference proceedings; weekly current awareness alerts for a further 44 journals; monthly BioMed Central email alerts; contacted investigators of the previously identified ongoing studies by email to enquire about any new or imminent publications. Reference lists of retrieved studies	● Pregnant women of any BMI<br>● RCTs<br>● Interventions including diet or exercise measures, or both (results reported for diet/PA, PA only, diet only separately)	● Not reported	● 65 studies<br>● Published: 1990 to 2014<br>● Study design: RCT n=65<br>● n=>13,523 women (49 RCTs, n=11,444 women included in meta-analysis)	Australia n=11<br>Belgium n=2<br>Canada n=5<br>Denmark n=3<br>Finland n=3<br>Germany n=1<br>Ireland n=1<br>Italy n=2<br>Kosovo n=1<br>Norway n=2<br>Sweden n=2<br>Netherlands n=2<br>Spain n=3<br>UK n=1<br>USA n=20<br>Brazil n=4<br>Columbia n=1<br>Taiwan n=1
Oostdam *et al.* 2011 [41]	To systematically review literature on the effectiveness of interventions to prevent GDM.	Bibliographic databases: PubMed, EMBASE, CENTRAL<br>Supplementary searches: reference lists of included studies	● Pregnant women<br>● Languages: English, German, Danish, Dutch, Finnish, Norwegian, and Swedish.	● Women with GDM or pre-existing diabetes type I or type II.	● 19 studies<br>● Published: 1983 to 2010<br>● Study design: RCT n=19<br>● n=1,998 women	Not reported

			• (quasi) RCT of an intervention that could prevent GDM (results reported separately for diet only and PA only)<br>• Outcomes: GDM, fasting blood glucose			
Quinlivan *et al.* 2011 [42]	To estimate whether antenatal dietary interventions restrict maternal weight gain in obese pregnant women without compromising newborn birth weight.	Bibliographic databases: PubMed<br>Supplementary searches: Cochrane Controlled Trials Register, reference lists of the retrieved studies	• RCTs<br>• Antenatal dietary interventions provided to pregnant women<br>• Overweight or obese at booking.	• Information only as an abstract<br>• Trials included women of all weight categories at booking<br>• Outcomes for overweight/obese women could not be extracted.	• 4 studies<br>• Published: 2008-2011<br>• Study design: RCT n= 4<br>• n=537 women	Denmark n=1<br>USA n=1<br>Belgium n=1<br>Australia n=1
Rogozińska *et al.* 2015 [14]	To assess the effects of nutritional manipulation in pregnancy on GDM and relevant maternal and fetal outcomes.	Bibliographic databases: Medline, Embase, Cochrane Database<br>Supplementary searches: reference lists of the included studies.	• Randomised studies<br>• Nutritional interventions in pregnancy<br>• Diet based advice, combination of diet and PA, nutritional supplements (reports outcomes for diet and combined diet/PA separately)	• Studies that evaluated only PA	• 20 studies<br>• Published: 2002-2014<br>• Study design: RCT n=20<br>• n=6,444 women	Not reported
Thangaratinam *et al.* 2012a [16]	To evaluate the effectiveness of dietary and lifestyle interventions in reducing or preventing obesity in pregnancy and to assess the beneficial and adverse effects of the interventions on obstetric, fetal and neonatal outcomes.	Bibliographic databases: MEDLINE, EMBASE,<br>BIOSIS, Latin American and Caribbean Health Sciences Literature (LILACS), Science Citation Index, Cochrane Database of Systematic Reviews, CENTRAL, DARE, HTA database, PsycINFO, Inside Conferences, Systems for Information in Grey Literature (SIGLE), Dissertation Abstracts<br>Supplementary searches: ClinicalTrials.gov. Internet specialist gateways (e.g. OMNI), general search engines (e.g. Google) and meta-search engines (e.g. Copernic)	• Normal weight, overweight or obese<br>• Any setting.<br>• Dietary, PA and behavioural change intervention<br>• Comparison: women with no intervention or routine antenatal care<br>• RCTs or non-randomised studies and observational studies when the evidence from RCTs was insufficient.<br>• Maternal and fetal outcomes.	• Underweight<br>• No data to estimate effectiveness measures	• 88 studies<br>• Published: 1975 to 2010<br>• Study design: RCT n= 40; Non-RCT n=16; Cohort n=26; Case-control n=6<br>• n=182,139 women	Not reported
Thangaratinam *et al.* 2012b [17]	To evaluate the effects of dietary and lifestyle interventions in pregnancy on maternal and fetal weight and to quantify the effects of these interventions on obstetric outcomes.	Bibliographic databases: Medline, Embase, BIOSIS, LILACS, Science Citation Index, Cochrane Database of Systematic Reviews, Database of Abstracts of Reviews of Effects, Health Technology Assessment Database, PsychInfo, Inside Conferences, Systems for Information in Grey Literature (SIGLE), Dissertation Abstracts, and<br>Supplementary searches: Internet searches using specialist search gateways (such as OMNI), general search engines (such as Google) and meta-search engines (such as Copernic). Cochrane Central Register of Controlled Trials, Clinical Trials.gov.	• RCTs<br>• Evaluated any dietary or lifestyle interventions with potential to influence maternal and fetal outcomes related to weight (reported results for diet/PA, diet only and PA only separately)	• Underweight	• 44 studies<br>• Published: 1976 to 2011<br>• Study design: RCT n=44<br>• n=7,278 women	Not reported
IWiP 2017 [18]	To synthesise the evidence on the overall and differential effects of interventions based on diet and PA during pregnancy, primarily on GWG and maternal and offspring composite outcomes.	Bibliographic databases: Medline, Embase, Cochrane Database of Systematic Reviews, Database of Abstracts of Reviews of Effects, Health Technology Assessment	• RCTs<br>• Assessed the effects of interventions based on diet, PA, and mixed interventions in pregnancy, on maternal and offspring outcomes	• Studies that only included women with GDM<br>• Involved animals<br>• Reported only non-clinical outcomes<br>• Published before 1990.	• 36 studies<br>• Published: 1993 to 2017<br>• Study design: RCT n= 36<br>• n=12,526 women	Europe n=22<br>North America n=4<br>Australia n=4<br>Brazil n=4<br>Egypt n=1<br>Iran n=1

		Supplementary searches: Cochrane Central Register of Controlled Trials	(reported results for diet/PA, diet only and PA only separately)			
Song *et al.* 2016 [24]	To address the efficacy of lifestyle intervention during pregnancy, i.e. diet and physical activity, on the risk of GDM.	Bibliographic databases: PubMed, Cochrane Library, Web of Science, Springer Link, JAMA, Ovid, Sino Med, Wiley Online Library, Science Direct Embase, Clinicaltrials.gov, Lancet, Nature, Science, The New England Journal of Medicine, BioMedNet and Google Scholar) for Supplementary searches: manual searches of the reference lists of included studies and relevant reviews.	• RCTs that only evaluated lifestyle interventions during the first two trimesters of pregnancy with an outcome measure of GDM<br>• Studies published in English and Chinese	• Studies involving women with pre-existing type 1 or type 2 diabetes or having existing GDM.<br>• Studies on interventions with only nutrient supplements<br>• Systematic reviews, meta-analysis, observational studies, study protocol and pilot study	• 29 studies<br>• Published: 2002 to 2015<br>• Study design: RCTs n=29<br>• n=11,487 women	USA n=6<br>UK n=1<br>Finland n=3<br>Australia n=3<br>Ireland n=1<br>Italy n=1<br>Denmark n=3<br>Spain n=5<br>Canada n=2<br>Belgium n=1<br>Norway n=1<br>Netherlands n=1<br>India n=1
Tieu *et al.* 2017 [43]	To assess the effects of dietary advice interventions for preventing GDM and associated adverse health outcomes for women and their babies.	Bibliographic databases: MEDLINE, Embase and CINAHL<br>Supplementary searches: Cochrane Pregnancy and Childbirth's Trials Register, CENTRAL, hand searched journals, conference proceedings, journals reviewed via the current awareness service, reference lists of retrieved articles	• RCTs and quasi-RCTs involving pregnant women<br>• Interventions that assessed any type of dietary advice before testing for GDM<br>• Involving pregnant women regardless of age, gestation, parity or plurality.	• Cross-over trials<br>• Trials presented only as abstracts<br>• Studies involving women with pre-existing type 1 or type 2 diabetes<br>• Interventions assessing combined dietary advice and exercise interventions	• 11 studies<br>• Published: 1983 to 2016<br>• Study design: RCTs n=11<br>• n=2,786 women	USA n=2<br>UK n=1<br>Finland n=1<br>Australia n=4<br>Brazil n=1<br>Ireland n=1<br>Denmark n=1
Zhang *et al.* 2018 [44]	To analyse the overall effects of low-GI diets on maternal and newborn outcomes in pregnant women regardless of their health status.	Bibliographic databases: PubMed, Clinical Trials<br>Supplementary searches: Cochrane Central Register of Controlled Trials, hand searching	• RCT conducted in pregnant women<br>• ≥18 years old, singleton pregnancy<br>• With a control or comparison group<br>• Intervention was low-GI diet with dietary GI level, >4 weeks	• None reported	• 11 studies<br>• Published: 1997 to 2016<br>• Study design: RCT n=11<br>• n=1,985 women	Australia n=5<br>USA n=2<br>Ireland n=1<br>Mexico n=1<br>Canada n=1<br>China n=1
Gresham *et al.* 2014 [45]	To synthesize the best of the available evidence by conducting a systematic review and meta-analysis to determine whether dietary interventions before or during pregnancy have any effect on neonatal or infant outcomes.	Bibliographic databases: EMBASE; Pre-Medline; MEDLINE; Proquest; Web of Science; CINAHL; Scopus; The Cochrane Library; Mosby Index; and Maternity and Infant Care.<br>Supplementary searches: hand searching	• Preconception or pregnant women, any age, weight or BMI, without date limits.<br>• At least three RCTs per outcome<br>• RCTs or pseudo-RCTs on dietary interventions with any intensity, frequency or timing.<br>• Positive or neutral methodological quality	• Health conditions that may influence dietary intake.<br>• Non-English language.<br>• Studies in animals<br>• Case study, editorial, conference proceeding<br>• Studies in GWG or nutrient supplementation (i.e. tablet form).<br>• Duplicate populations.<br>• Multiple birth<br>• Negative methodological quality	• 29 studies (31 publications)<br>• Published: 1978 to 2011<br>• Study design: RCT n=29<br>• n=10,026 women	USA n=10<br>Netherlands n=3<br>Denmark n=3<br>Finland n=2<br>Australia n=1<br>Greece n=1<br>Italy n=1<br>Norway n=1<br>Chile n=1<br>Gambia n=1<br>Taiwan n=1<br>Columbia n=1<br>East Java n=1<br>Iran n=1<br>South Africa n=1
Craemer *et al.* 2019 [26]	To evaluate nutrition-only, exercise-only, and nutrition-plus-exercise interventions for optimizing GWG based on the 2009 IOM guidelines	Bibliographic databases: PubMed, Google Scholar<br>Supplementary searches: All studies included in 2015 Cochrane Review (Muktabhant *et al.* 2015 [11]) were examined	• RCTs (parallel or cross sectional) (results reported separately for diet/PA, diet only and PA only)<br>• At least 20 singleton pregnant women<br>• Women aged 18 years or older<br>• Control group standard obstetric care<br>• Report means of GWG based on baseline BMI or pre-pregnancy BMI	• Studies published before 2009<br>• Mean GWG not reported<br>• Study in trial state	• 31 studies<br>• Published: 2009 to 2016<br>• Study design: RCT n=31<br>• n=8,558 women	Netherlands n=1<br>USA n=4<br>Turkey n=1<br>Belgium n=1<br>Australia and New Zealand n=3<br>Italy n=2<br>Norway n=2<br>Ireland n=3

			● Use 2009 IOM guidelines for GWG. For studies that did not use these, additional analysis was made based on the reported mean GWG			Taiwan n=1<br>Canada n=3<br>Finland n=3<br>China n=1<br>Brazil n=1<br>UK n=1<br>Germany n=1<br>Denmark n=2<br>Spain n=1
Syngelaki *et al.* 2019 [35]	To examine whether diet and/or exercise can prevent PE in overweight or obese pregnant women.	Bibliographic databases: PubMed, Embase, CINAHL, Web of Science<br>Supplementary searches: Cochrane CENTRAL, references, other reviews	● RCTs that evaluated the effect of diet and/or exercise on the risk of PE and hypertensive disorders of pregnancy (results reported separately for diet/PA, diet only and exercise only)<br>● Overweight/ obese women	● Not reported	● 23 studies<br>● Published: 2000 to 2017<br>● Study design: RCT n=23<br>● n=7,236 women	Not reported
Walker *et al.* 2018 [36]	To address compare the efficacy of all approaches that have been used to prevent excessive GWG (e.g., diet, PA, lifestyle, sleep, eHealth, and medical).	Bibliographic databases: MEDLINE, EMBASE, Cochrane Library, PsycInFO, CINAHL, Scopus, LILACS<br>Supplementary searches: reference lists of included trials and systematic reviews	● RCTs of interventions designed to prevent excessive GWG.<br>● Comparators were standard care, an alternate intervention, or placebo.<br>● Participants were women of all ages, ethnicities, and pre-pregnancy weight status with singleton pregnancies.	● Studies for women with pre-existing diabetes (type 1 or type 2) or GDM	● 89 studies<br>● Published: 2000 to 2018<br>● Study design: RCT n=89<br>● n=25,345 women	USA n=22<br>Australia n=11<br>Spain n=10<br>Italy n=5<br>Finland n=3<br>Egypt n=1<br>Turkey n=2<br>Sweden n=2<br>Brazil n=4<br>Ireland n=2<br>Denmark n=3<br>Argentina n=1<br>Canada n=4<br>Norway n=3<br>Iran n=1<br>Netherlands n=2<br>China n=3<br>Belgium n=2<br>Taiwan n=1<br>India n=1<br>UK n=3<br>Columbia n=1<br>Germany n=1<br>Multiple European countries n=1
Shieh *et al.* 2018 [38]	To analyse data from published studies using a RCT design on the prevention of excessive GWG specifically among pregnant overweight and obese women.	Bibliographic databases: PubMed, Academic Search Premier, CINAHL, MEDLINE, and PsyInfo<br>Supplementary searches: None reported	● RCT<br>● Exercise, diet or both as the intervention<br>● Healthy overweight and/or obese pregnant adult women<br>● GWG an outcome	● Sub-study from RCTs<br>● Systematic reviews<br>● Meta-analyses<br>● Interventions to increase pregnancy weight gain<br>● Women with an identified health problem (e.g. AIDS, GDM).	● 23 studies<br>● Published: 2008 to 2016<br>● Study design: RCT n=23<br>● n=6,920 women	Belgium n=2<br>Denmark n=3<br>Norway n=1<br>UK n=1<br>Italy n=1<br>Australia n=6<br>USA n=5<br>New Zealand n=1<br>Canada n=1<br>Brazil n=1<br>Multiple European countries n=1
Bennett *et al.* 2017 [39]	To comprehensively evaluate the global impact of interventions	Bibliographic databases: Cochrane	● RCTs conducted in humans	● Studies in animals	● 45 studies<br>● Published: 2002 to 2016	Australia n=8<br>China n=8

	designed to prevent excessive GWG on the incidence of GDM, and whether the effects differ by maternal BMI or ethnicity.	Library, MEDLINE, EMBASE, PsycINFO, CINAHL, Scopus, LILACS, CNKI, WangFang, VIP<br>Supplementary searches: reference lists of systematic reviews	• Primary or secondary aim to reduce/prevent excessive GWG<br>• Reported on the incidence of GDM in both groups separately<br>• All languages and countries	• Studies conducted in women with existing<br>• Studies to encourage GWG<br>• Duplication populations	• Study design: RCT n=45<br>• n=15,293 women	USA n=7<br>Spain n=6<br>Canada n=3<br>Denmark n=3<br>Finland n=2<br>UK n=2<br>Ireland n=1<br>Netherlands n=1<br>Belgium n=1<br>Italy n=1<br>Norway n=1<br>Multiple European countries n=1

S5d: Systematic reviews reporting outcomes for physical activity only interventions

Review	Aim of the review	Search strategy	Inclusion criteria	Exclusion criteria	Included studies	Study locations
Elliot-Sale *et al.* 2015 [46]	To review the evidence from studies employing exercise-only interventions for weight management among pregnant and postpartum women.	Bibliographic databases: CINAHL, PubMed, MEDLINE, OVID, Springer Link, ScienceDirect, Oxford Journal, BioMed Central and Web of Science<br>Supplementary searches: Cochrane Central Register of Controlled Trials (CENTRAL)	● RCTs and quasi-RCTs<br>● Pregnancy or the postpartum period<br>● Comparing an exercise-based weight management intervention with routine care or another type of intervention<br>● Healthy pregnant and postpartum women free from pregnancy-related complications, medical conditions, not taking medications known to affect body weight or exercise performance	● All review papers<br>● Conference proceedings.<br>● Papers before 1990<br>● Studies on women under 18 years<br>● Underweight women, at risk of LBW or insufficient GWG.<br>● Postpartum period was not specified.	● 3 studies<br>● Published: 2011<br>● Study design: RCT n=3<br>● n=214 women	Spain n=1<br>Norway n=1<br>Brazil n=1
Han *et al.* 2012 [47]	To assess the effects of physical exercise for pregnant women for preventing glucose intolerance or GDM.	Bibliographic databases: Cochrane Central Register of Controlled Trials (CENTRAL); MEDLINE; EMBASE;<br>Supplementary searches: ClinicalTrials.gov, the WOMBAT Perinatal Trials Registry, hand searches of 30 journals; proceedings of major conferences; current awareness alerts for a further 44 journals; BioMed Central email alerts; reference lists of retrieved articles	● Published RCTs<br>● Assessing the effects of physical exercise in preventing pregnancy glucose intolerance or GDM<br>● Assessing the effects of lifestyle interventions if able to extract data for physical exercise separately.	● Quasi-RCTs and cross-over trials.<br>● Women with pre-existing type 1 and type 2 diabetes	● 5 studies<br>● Published: 2009 to 2012<br>● Study design: RCT n=5<br>● n=1,115 women	Australia n=2<br>New Zealand n=1<br>Spain n=1<br>Norway n=1
Lamina & Agbanusi 2013 [48]	To present the best available evidence on the effect of aerobic exercise training on maternal weight gain during pregnancy.	Bibliographic databases: Medline<br>Supplementary searches: reference lists of any relevant identified articles	● Published in English<br>● Study population defined<br>● RCTs of aerobic moderate intensity exercise interventions<br>● Body weight is main outcome measure<br>● Peer reviewed publications	● Not reported	● 11 studies<br>● Published: 1976 to 2012<br>● Study design: RCT n=11<br>● n=1,177 women	Not reported
Madhuvrata *et al.* 2015 [10]	To see if there was any intervention which could be used for primary prevention of GDM in women with risk factors for GDM.	Bibliographic databases: Medline, Embase, conference abstract databases<br>Supplementary searches: Cochrane specialised trials register, ClinicalTrials.gov	● RCTs or quasi-RCTs comparing intervention with standard care in women with risk factors for GDM (including raised BMI, previous GDM, previous infant with birth weight >4500 g, family history of diabetes, high-risk ethnic groups, PCOS)<br>● (Results reported for diet/PA, PA only, diet only separately)	● Trials comparing interventions in pregnant women with no risk factors for GDM and one intervention versus another	● 14 studies<br>● Published: 2002 to 2011<br>● Study design: RCT n=14<br>● n=2,422 women	USA n=3<br>Australia n=3<br>Norway n=2<br>Denmark n=2<br>Holland n=1<br>Belgium n=1<br>Finland n=2
Oostdam *et al.* 2011 [41]	To systematically review literature on the effectiveness of interventions to prevent GDM.	Bibliographic databases: PubMed, EMBASE, CENTRAL<br>Supplementary searches: reference lists of included studies	● Pregnant women<br>● (quasi) RCT of an intervention that could prevent GDM (results reported separately for diet only and PA only)<br>● Outcomes: GDM, fasting blood glucose<br>● Languages: English, German, Danish, Dutch, Finnish, Norwegian, and Swedish.	● Women with GDM or pre-existing diabetes type I or type II.	● 19 studies<br>● Published: 1983 to 2010<br>● Study design: RCT n=19<br>● n=1,998 women	Not reported
Russo *et al.* 2015 [49]	To summarize all available data from RCTs reported to date looking at the effect of PA-only interventions on the risk of GDM.	Bibliographic databases: PubMed<br>Supplementary searches: Cochrane Central Register of Controlled Trials, ClinicalTrials.gov	● RCT<br>● Pregnant women without GDM<br>● Increased PA was the only intervention and comparison with usual care<br>● GDM was documented separately for the control and intervention groups	● Any articles that did not meet the established inclusion criteria.	● 10 studies<br>● Published: 2010 to 2014<br>● Study design: RCT n=10<br>● n=3,401 women	Spain n=3<br>USA n=2<br>Australia n=1<br>Croatia n=1<br>Denmark n=1<br>Netherlands n=1<br>Norway n=1

Sanabria-Martínez *et al.* 2015 [50]	To conduct a meta-analysis of RCTs assessing the effectiveness of physical exercise interventions during pregnancy to prevent GDM and excessive maternal weight gain (MWG).	<u>Bibliographic databases</u>: Cochrane Library Plus, Science Direct, EMBASE, PubMed, Web of Science<br><u>Supplementary searches</u>: ClinicalTrials.gov	● Sedentary healthy women or those with low levels of PA (exercising <20 minutes on <3 days per week)<br>● Uncomplicated singleton pregnancies<br>● RCT in which the control group received no type of physical exercise<br>● Physical exercise programmes of low to moderate intensity.	● High risk of PTB<br>● Involved in any other trial<br>● Contraindication for exercise, as advised by an obstetrician<br>● Not planning to give birth at the same obstetrics department which the medical control of the pregnancy was performed.	● 13 studies<br>● Published: 1990 to 2014<br>● Study design: RCT n=13<br>● n=2,873 women	Spain n=7<br>Croatia n=1<br>Brazil n=1<br>Norway n=1<br>New Zealand n=1<br>USA n=2
Streuling *et al.* 2011 [51]	To perform a systematic review and meta-analysis to find out whether PA in pregnancy might help avoid high GWG.	<u>Bibliographic databases</u>: MEDLINE, EMBASE, ISI Web of Knowledge, containing Web of Science, BIOSIS Previews, Current Contents Connect and Journal Citation Reports<br><u>Supplementary searches</u>: Cochrane CENTRAL, hand searching	● RCT intervention comprised solely PA in healthy women<br>● Control group with no intervention promoting PA<br>● GWG was documented for control and intervention groups separately	● Not reported	● 12 studies<br>● Published: 1983 to 2010<br>● Study design: RCT n=12<br>● n=1,073 women	USA n=4<br>Iran n=2<br>Spain n=1<br>Australia n=1<br>New Zealand n=1<br>Brazil n=3
Sui *et al.* 2012 [52]	To systematically identify and evaluate the currently available literature relating to antenatal exercise interventions specifically targeting pregnant women who are overweight or obese.	<u>Bibliographic databases:</u> PUBMED, SCOPUS, the<br><u>Supplementary searches</u>: Cochrane Controlled Trials Register (CENTRAL) and the Australian and International Clinical Trials Registers, reference lists of included studies	● RCTs and QCTs with a monitored lifestyle intervention<br>● Comparison group: routine care<br>● Healthy overweight or obese women with a singleton pregnancy	● Intervention involved consultation/education alone<br>● Study information was available in abstract form only	● 7 studies<br>● Published: 2004 to 2011<br>● Study design: RCT n= 6; QRT n= 1<br>● n=276 women	Canada n=2<br>Australia n=2<br>Brazil n=2<br>Spain & Sweden n=1
Thangaratinam *et al.* 2012a [16]	To evaluate the effectiveness of dietary and lifestyle interventions in reducing or preventing obesity in pregnancy and to assess the beneficial and adverse effects of the interventions on obstetric, fetal and neonatal outcomes.	<u>Bibliographic databases</u>: MEDLINE, EMBASE,<br>BIOSIS, Latin American and Caribbean Health Sciences Literature (LILACS), Science Citation Index, Cochrane Database of Systematic Reviews, CENTRAL, DARE, HTA database, PsycINFO, Inside Conferences, Systems for Information in Grey Literature (SIGLE), Dissertation Abstracts<br><u>Supplementary searches</u>: ClinicalTrials.gov. Internet specialist gateways (e.g. OMNI), general search engines (e.g. Google) and meta-search engines (e.g. Copernic)	● Normal weight, overweight or obese<br>● Any setting.<br>● Dietary, PA and behavioural change intervention<br>● Comparison: women with no intervention or routine antenatal care<br>● RCTs or non-randomised studies and observational studies when the evidence from RCTs was insufficient.<br>● Maternal and fetal outcomes.	● Underweight<br>● No data to estimate effectiveness measures	● 88 studies<br>● Published: 1975 to 2010<br>● Study design: RCT n= 40; Non-RCT n=16; Cohort n=26; Case-control n=6<br>● n=182,139 women	Not reported
Thangaratinam *et al.* 2012b [17]	To evaluate the effects of dietary and lifestyle interventions in pregnancy on maternal and fetal weight and to quantify the effects of these interventions on obstetric outcomes.	<u>Bibliographic databases</u>: Medline, Embase, BIOSIS, LILACS, Science Citation Index, Cochrane Database of Systematic Reviews, Database of Abstracts of Reviews of Effects, Health Technology Assessment Database, PsychInfo, Inside Conferences, Systems for Information in Grey Literature (SIGLE), Dissertation Abstracts, and<br><u>Supplementary searches</u>: Internet searches using specialist search gateways (such as OMNI), general search engines (such as Google) and meta-search engines (such as	● RCTs<br>● Evaluated any dietary or lifestyle interventions with potential to influence maternal and fetal outcomes related to weight (reported results for diet/PA, diet only and PA only separately)	● Underweight	● 44 studies<br>● Published: 1976 to 2011<br>● Study design: RCT n=44<br>● n=7,278 women	Not reported

		Copernic). Cochrane Central Register of Controlled Trials, Clinical Trials.gov.				
da Silva *et al.* 2017 [53]	To compare the associations between leisure time PA (LTPA) in pregnancy on maternal and child health outcomes between RCTs and cohort studies.	Bibliographic databases: PubMed, Web of Science, EBSCO, Clinical Trials and Cochrane Database.<br>Supplementary Searches: Controlled Trials websites, Reference lists of included studies	• RCT with at least one PA component in a structured program during pregnancy<br>• Outcomes presented separately for intervention and the control groups.<br>• Maternal GDM, PE, GWG or child health outcomes (birth weight, PTB, birth, fetal growth).<br>• English, Spanish, and Portuguese language	• Women with comorbidities, such as diabetes, PE, or obesity.<br>• Cohort studies if the sample was selected among a specific group of women with a high risk of developing a given outcome.<br>• Studies evaluating exclusively PA before pregnancy	• 81 studies<br>• Published: 1993 to 2015<br>• Study design: RCT n=30, Cohort n=51<br>• n=598,185 women	Spain n=8<br>Sweden n=5<br>Kosovo n=1<br>Norway n=9<br>Croatia n=1<br>Brazil n=8<br>USA n=28<br>New Zealand n=3<br>Colombia n=1<br>Iran n=3<br>Canada n=1<br>UK n=2<br>China n=1<br>Holland n=1<br>Denmark n=6<br>India n=1<br>Australia n=2
Di Mascio *et al.* 2016 [54]	To evaluate the effects of exercise during pregnancy on the risk of PTB.	Bibliographic databases: MEDLINE, EMBASE, Web of Science, Scopus, OVID, and Cochrane Library<br>Supplementary Searches: ClinicalTrial.gov	• RCTs reporting PTB as an outcome.<br>• Women with uncomplicated, singleton pregnancies without any obstetric contraindication to PA.	• Only underweight or only overweight or obese women<br>• Diet counselling, weight monitoring, exercise reduction.<br>• Quasi-RCTs	• 9 studies<br>• Published: 1990 to 2016<br>• Study design: RCT n=9<br>• n=2,059 women	Spain n=5<br>Brazil n=2<br>USA n=1<br>Norway n=1
IWiP 2017 [18]	To synthesise the evidence on the overall and differential effects of interventions based on diet and PA during pregnancy, primarily on GWG and maternal and offspring composite outcomes.	Bibliographic databases: Medline, Embase, Cochrane Database of Systematic Reviews, Database of Abstracts of Reviews of Effects, Health Technology Assessment<br>Supplementary searches: Cochrane Central Register of Controlled Trials	• RCTs<br>• Assessed the effects of interventions based on diet, PA, and mixed interventions in pregnancy, on maternal and offspring outcomes (reported results for diet/PA, diet only and PA only separately)	• Studies that only included women with GDM<br>• Involved animals<br>• Reported only non-clinical outcomes<br>• Published before 1990.	• 36 studies<br>• Published: 1993 to 2017<br>• Study design: RCT n= 36<br>• n=12,526 women	Europe n=22<br>North America n=4<br>Australia n=4<br>Brazil n=4<br>Egypt n=1<br>Iran n=1
Sanabria-Martínez *et al.* 2016 [55]	To carry out a systematic review and meta-analysis of RCTs aiming to assess the effectiveness of physical exercise interventions during pregnancy on the neonate through the Apgar score, birth weight, and gestational age.	Bibliographic databases: Cochrane Library Plus, Science Direct, EMBASE, PubMed, Web of Science<br>Supplementary searches: ClinicalTrials.gov, references of published studies.	• Published January 1990 to June 2014.<br>• Papers written in Spanish or English.<br>• Sedentary healthy women or with low levels of PA (exercising <20 minutes on <3 days per week) with uncomplicated singleton pregnancies<br>• Physical exercise programs with no restrictions on frequency, duration, or type of exercise.	• Women at high risk of PTB<br>• Women involved in any other trial<br>• Women with any contraindication to practice exercise<br>• Women who were not planning to give birth at the same department in which the medical control of the pregnancy was performed.	• 14 studies<br>• Published: 2000 to 2014<br>• Study design: RCT n=14<br>• n=3,044 women	Spain n=7<br>Croatia n=1<br>Brazil n=2<br>Norway n=1<br>New Zealand n=1<br>USA n=2
Song *et al.* 2016 [24]	To address the efficacy of lifestyle intervention during pregnancy, i.e. diet and physical activity, on the risk of GDM.	Bibliographic databases: PubMed, Cochrane Library, Web of Science, Springer Link, JAMA, Ovid, Sino Med, Wiley Online Library, Science Direct Embase, Clinicaltrials.gov, Lancet, Nature, Science, The New England Journal of Medicine, BioMedNet and Google Scholar)<br>Supplementary searches: manual searches of the reference lists of included studies and relevant reviews.	• RCTs that only evaluated lifestyle interventions during the first two trimesters of pregnancy with an outcome measure of GDM<br>• Studies published in English and Chinese	• Studies involving women with pre-existing type 1 or type 2 diabetes or having existing GDM.<br>• Studies on interventions with only nutrient supplements<br>• Systematic reviews, meta-analysis, observational studies, study protocol and pilot study	• 29 studies<br>• Published: 2002 to 2015<br>• Study design: RCTs n=29<br>• n=11,487 women	USA n=6<br>UK n=1<br>Finland n=3<br>Australia n=3<br>Ireland n=1<br>Italy n=1<br>Denmark n=3<br>Spain n=5<br>Canada n=2<br>Belgium n=1<br>Norway n=1<br>Netherlands n=1<br>India n=1

Zheng *et al.* 2017 [56]	To explore the effect of exercise intervention on GDM.	Bibliographic databases: PubMed, EMbase, Web of science, EBSCO and the Cochrane library<br>Supplementary searches: reference lists of retrieved studies, relevant reviews and conference abstracts hand-searched.	● RCTs in pregnant women<br>● Exercise intervention at 10-22 weeks of pregnancy compared to standard care<br>● GDM and gestational age at birth as outcomes	● None reported	● 5 studies<br>● Published: 2012 to 2016<br>● Study design: RCT n=5<br>● n=1,691 women	Not reported
Chatzakis *et al*. 2019 [57]	To use direct and indirect data to compare interventions to each other and placebo and identify the most effective, for the prevention of GDM in overweight and obese pregnant women.	Bibliographic databases: PubMed, Scopus<br>Supplementary searches: Cochrane Central Register of Controlled Trials (Central), US Registry of clinical trials, references of the retrieved articles, automated search using PubMed's ''search for related articles" function	● RCTs comparing the administration of metformin, exercise programs, vitamin D and probiotics with placebo/no intervention<br>● Overweight and obese pregnant women<br>● No country or publication date restrictions were applied.	● Studies focused on women with a BMI<25 kg/m^2<br>● Women with pre-existing diabetes.	● 23 studies<br>● Published: 2009 to 2019<br>● Study design: RCT n=23<br>● n=4,237 women	UK n=2<br>Netherlands n=2<br>Australia n= 6<br>New Zealand n=2<br>Ireland n=2<br>Brazil n=1<br>USA n=1<br>Denmark n=1<br>Canada n=1<br>Norway n=1<br>China n=1<br>Finland n=1<br>Iran n=1<br>Spain n=1
Craemer *et al.* 2019 [26]	To evaluate nutrition-only, exercise-only, and nutrition-plus-exercise interventions for optimizing GWG based on the 2009 IOM guidelines.	Bibliographic databases: PubMed, Google Scholar<br>Supplementary searches: All studies included in 2015 Cochrane Review (Muktabhant *et al.* 2015 [11] were examined	● RCTs (parallel or cross sectional) (results reported separately for diet/PA, diet only and PA only)<br>● At least 20 singleton pregnant women<br>● Women aged 18 years or older<br>● Control group standard obstetric care<br>● Report means of GWG based on baseline BMI or pre-pregnancy BMI<br>● Use 2009 IOM guidelines for GWG. For studies that did not use these, additional analysis was made based on the reported mean GWG	● Studies published before 2009<br>● Mean GWG not reported<br>● Study in trial state	● 31 studies<br>● Published: 2009 to 2016<br>● Study design: RCT n=31<br>● n=8,558 women	Netherlands n=1<br>USA n=4<br>Turkey n=1<br>Belgium n=1<br>Australia and New Zealand n=3<br>Italy n=2<br>Norway n=2<br>Ireland n=3<br>Taiwan n=1<br>Canada n=3<br>Finland n=3<br>China n=1<br>Brazil n=1<br>UK n=1<br>Germany n=1<br>Denmark n=2<br>Spain n=1
Davenport *et al.* 2019a [27]	The aim of the study is to perform a systematic review of the relationship between prenatal exercise and fetal or new-born death.	Bibliographic databases: MEDLINE, EMBASE, PsycINFO, Cochrane Database of Systematic Reviews, Cochrane Central Register of Controlled Trials, Scopus and Web of Science Core Collection, CINAHL Plus with Full-text, Child Development and Adolescent Studies, ERIC, Sport Discus, ClinicalTrials.gov and the Trip Database<br>Supplementary searches: None reported	● Pregnant women without contraindication to exercise.<br>● Objective or subjective measures of frequency, intensity, duration, volume or type of exercise.<br>● Acute (ie, a single exercise session) or habitual (ie, usual activity) prenatal exercise and interventions including exercise alone or in combination with other interventions (reports outcomes for PA and combined diet/PA separately)<br>● Outcomes were diagnosis of depression or anxiety and severity of symptoms of depression or anxiety.	● Women with absolute or relative contraindications to exercise.<br>● Exercise began after the initiation of labour.<br>● Case studies and not original research (narrative or systematic reviews and meta-analyses).<br>● Studies not published in either English, Spanish or French	● 46 studies<br>● Published: 1976 to 2016<br>● Study design: RCT n=30, n-RCT n=8 (4 cohort, 1 cross-sectional, 3 case-control studies)<br>● n=266,778 women	Finland n=4<br>UK n= 3<br>USA n=10<br>Denmark n=3<br>India n=2<br>Canada n= 2<br>Japan n= 2<br>Germany n=1<br>China n= 3<br>Sweden n= 5<br>Australia n= 2<br>Belgium n=1<br>South Africa n=1<br>Norway n = 3<br>Benin n=1

						New Zealand n= 1<br>Brazil n=1<br>Greece n=1
Davenport *et al.* 2018a [28]	To examine the influence of prenatal exercise on depression and anxiety during pregnancy and the postpartum period.	Bibliographic databases: MEDLINE, EMBASE, PsycINFO, Cochrane Database of Systematic Reviews, Scopus and Web of Science Core Collection, CINAHL Plus with Full-text, Child Development & Adolescent Studies, ERIC, Sport Discus, the Trip Database<br>Supplementary searches: Cochrane Central Register of Controlled Trials, ClinicalTrials.gov	• Pregnant women without contraindication to exercise.<br>• Objective or subjective measures of frequency, intensity, duration, volume or type of exercise.<br>• Acute (ie, a single exercise session) or habitual (ie, usual activity) prenatal exercise and interventions including exercise alone or in combination with other interventions (reports outcomes for PA and combined diet/PA separately)<br>• Outcomes were diagnosis of depression or anxiety and severity of symptoms of depression or anxiety.	• Women with absolute or relative contraindications to exercise.<br>• Exercise began after the initiation of labour.<br>• Case studies and not original research (narrative or systematic reviews and meta-analyses).<br>• Studies not published in either English, Spanish or French	• 52 studies<br>• Published: 2008 to 2016<br>• Study design: RCTs n=26, non-RCT n=7, cohort n=10, cross-sectional n=6, case-control n=3<br>• n=131,406 women	USA n=18<br>Canada n=3<br>Sweden n=1<br>India n=2<br>Multi - European countries n= 1<br>Australia n=3<br>Egypt n=1<br>Norway n= 4<br>Poland n=2<br>Iran n=2<br>Finland n=1<br>Brazil n=1<br>UK n=3<br>Spain n=3<br>Serbia n=1<br>Colombia n=1<br>South Korea n=1<br>Denmark n=1<br>Japan n=1<br>Portugal n= 1
Davenport *et al.* 2018b [29]	To perform a systematic review and meta-analysis of the relationships between prenatal exercise and GDM, GH and PE.	Bibliographic databases: MEDLINE, EMBASE, PsycINFO, Cochrane Database of Systematic Reviews, Scopus and Web of Science Core Collection, CINAHL Plus with Full-text, Child Development & Adolescent Studies, ERIC, Sport Discus, the Trip Database<br>Supplementary searches: Cochrane Central Register of Controlled Trials, ClinicalTrials.gov	• Pregnant women without contraindication to exercise.<br>• Objective or subjective measures of frequency, intensity, duration, volume or type of exercise (reports outcomes for PA and combined diet/PA separately)<br>• Relevant outcomes were GDM, GH and PE.	• Women with absolute or relative contraindications to exercise.<br>• Exercise began after the initiation of labour.<br>• Case studies and not original research (narrative or systematic reviews and meta-analyses).<br>• Studies not published in either English, Spanish or French	• 106 studies<br>• Published: 1974 to 2017<br>• Study design: RCTs n=65, non-RCTs n=9, cohort n=13, cross-sectional n=11, case-control n=8<br>• n=273,182 women	USA n=32<br>Spain n=11<br>Canada n=11<br>Norway n=6<br>China n=6<br>UK n=3<br>Australia n=5<br>Denmark n=3<br>Finland n=4<br>Netherlands n=2<br>Italy n=1<br>Iran n=3<br>Brazil n=3<br>Belgium n=1<br>Sweden n=3<br>New Zealand n=2<br>Thailand n=1<br>India n=1<br>Kosovo n=1<br>Germany n=1<br>Croatia n=1<br>Pakistan n=1<br>Greece n=1<br>Japan n=1<br>Multiple European Countries n=2
Davenport *et al.* 2019b [30]	To assess the associations between prenatal PA (in terms of frequency, intensity, type and volume of PA),	Bibliographic databases: MEDLINE, EMBASE, PsycINFO, Cochrane Database of Systematic Reviews, Scopus and Web of Science	• Pregnant women without contraindication to exercise.<br>• Objective or subjective measures of frequency, intensity, duration, volume	• Women with absolute or relative contraindications to exercise.<br>• Exercise began after the initiation of labour.	• 113 studies<br>• Published: 1974 to 2017	USA n=32<br>Spain n=12<br>Australia n=7<br>Brazil n=7

	with maternal harms, labour and delivery outcomes.	Core Collection, CINAHL Plus with Full-text, Child Development & Adolescent Studies, ERIC, Sport Discus, the Trip Database<br>Supplementary searches: Cochrane Central Register of Controlled Trials, ClinicalTrials.gov	or type of exercise (reports outcomes for PA and combined diet/PA separately)<br>● Outcomes were PTB/prelabour rupture of membranes, caesarean section, instrumental delivery, induction of labour, length of labour, vaginal tears, fatigue, injury, musculoskeletal trauma, maternal harms (author defined) and diastasis recti.	● Case studies and not original research (narrative or systematic reviews and meta-analyses).<br>● Studies not published in either English, Spanish or French	● Study design: RCTs n=79, non-RCTs n=12, observational n=22<br>● n=52,858 women	Norway n=7<br>UK n=6<br>Canada n=5<br>Thailand n=4<br>Sweden n=4<br>Netherlands n=4<br>Denmark n=3<br>China n=3<br>India n=1<br>Poland n=2<br>Japan n=2<br>Italy n=2<br>Benin n=1<br>Belgium n=1<br>Switzerland n=1<br>Colombia n=1<br>Iran n=1<br>Germany n=1<br>Croatia n=1<br>Finland n=1<br>Mexico n=1<br>Turkey n=1<br>France n=1<br>**New Zealand n=1**
Davenport *et al.* 2018c [31]	To perform a systematic review and meta-analysis to explore the relationship between prenatal exercise and glycaemic control.	Bibliographic databases: MEDLINE, EMBASE, PsycINFO, Cochrane Database of Systematic Reviews, Scopus and Web of Science Core Collection, CINAHL Plus with Full-text, Child Development & Adolescent Studies, ERIC, Sport Discus, the Trip Database<br>Supplementary searches: Cochrane Central Register of Controlled Trials, ClinicalTrials.gov	● Pregnant women without contraindication to exercise.<br>● Objective or subjective measures of frequency, intensity, duration, volume or type of exercise.<br>● Original studies of any design (RCT and observational)<br>● Outcomes were indicators of glucose tolerance	● Women with absolute or relative contraindications to exercise.<br>● Exercise began after the initiation of labour.<br>● Case studies and not original research (narrative or systematic reviews and meta-analyses).<br>● Studies not published in either English, Spanish or French	● 58 studies<br>● Published: 1981 to 2017<br>● Study design: RCTs n=31, non-RCTs n=7, cohort n=8, cross-sectional n=12<br>● n=8,699 women	Not reported
Du *et al.* 2019 [58]	To assess the effect of physical exercise on maternal and infant outcomes in overweight and obese pregnant women.	Bibliographic databases: Cochrane Library, Embase, PubMed, Web of Science<br>Supplementary searches: ClinicalTrials.gov	● RCTs published in English<br>● Examined the effects of PA without dietary interventions during pregnancy.<br>● Overweight and obese pregnant women, singleton pregnancy, no exercise contraindications.	● Articles that were not in full text format<br>● Not published in English.	● 13 studies<br>● Published: 2009 to 2017<br>● Study design: RCT n=13<br>● n=1,439 women	Australia n=4<br>New Zealand n=1<br>Norway n=1<br>Denmark n=1<br>Canada n=1<br>Netherlands n=1<br>USA n=1<br>Ireland n=1<br>China n=1<br>Brazil n=1
Guillemette *et al.* 2018 [59]	To characterize and quantify the specific impact of prenatal exercise on children's cardiometabolic health markers, at birth and in childhood.	Bibliographic databases: Medline, EMBASE, Scopus, CINAHL, SPORTDiscus<br>Supplementary searches: CENTRAL, bibliographies of relevant narrative and systematic reviews and included studies.	● RCT or prospective cohort study<br>● Pregnant women<br>● Aerobic and/or resistance (strength) training exercises ≥4weeks in duration<br>● Birth weight (primary outcome), body composition (fat and/or lean mass), LGA; follow up (any time after birth) weight, body composition (fat and/or	● Non-human populations<br>● Intervention >60% non-aerobic/ resistance training (e.g. yoga)<br>● Acute exercise, or training <4 weeks in duration<br>● Exposures not distinguishing PA from aerobic/resistance training<br>● Not linking prenatal exercise to offspring outcomes	● 54 studies<br>● Published: 1993 to 2017<br>● Study design: RCT n=39, Cohort n=15<br>● RCT n=6,870 neonates	Finland n=1<br>USA n=12<br>Sweden n=2<br>Iran n=2<br>Brazil n=7<br>Spain n=7<br>Australia n=2<br>New Zealand n=2<br>Norway n=3

			lean mass), blood pressure, blood glucose, blood lipids	● Unequal controls, e.g., controls do not receive the same diet information<br>● Not reporting any of these offspring outcomes, or reporting in a non-extractable format and authors do not share original data		Netherlands n=1<br>Colombia n=1<br>Kosovo n=1<br>UK n=3<br>Ireland n=1<br>Canada n=2<br>Croatia n=1<br>Denmark n=5<br>China n=1
Ming *et al.* 2018 [60]	To investigate the effect of exercise during pregnancy on the occurrence of GDM among normal-weight pregnant women.	Bibliographic databases: Web of Science, Scopus (including Pubmed, MEDLINE and Embase), and the Cochrane Library<br>Supplementary searches: ClinicalTrials.gov, references for relevant articles	● RCTs of interventions with at least one type of exercise<br>● GDM was reported for both intervention and control groups<br>● Pregnant women with a pre-pregnancy BMI or a mean pre-pregnancy BMI ranging from 18.5–24.9 kg/m^2	● Interventions integrated other factors (e.g. diet)<br>● BMI <18.5 kg/m^2 or >25 kg/m^2<br>● Literature reviews, case reports or protocols, only abstract or conference contents published, or studies lacked specific data.	● 8 studies (9 trials)<br>● Published: 2012 to 2017<br>● Study design: RCT n=8<br>● n=3,256 women	Spain n=7<br>Norway n=1<br>Croatia n-=1
Nasiri-Amiri *et al.* 2019 [61]	To systematically review the articles on the effect of exercise activities on the prevention of GDM in obese and overweight pregnant women in order to achieve a regular summation in this regard.	Bibliographic databases: Medline, Cochrane Library, PubMed, Scopus, Web of Science, Embase, CINAHL<br>Supplementary searches: Hand searching, grey literature in ProQuest, Prospero. Dissertations and conference articles in Scopus and Web of science.	● RCTS<br>● Routine prenatal care compared to exercise in addition to routine care<br>● Obese and overweight pregnant women; singleton; no contraindication to exercise	● Review/descriptive studies on non-obese/overweight individuals<br>● Interventions were both exercise and other lifestyles (e.g. nutrition)<br>● Studies that did not compare control and intervention groups	● 8 studies<br>● Published: 2010 to 2018<br>● Study design: RCT n=8<br>● n=1,441 women	New Zealand n=1<br>Australia n=1<br>China n=1<br>Ireland n=1<br>Netherlands n=1<br>Norway n=1<br>Spain n=1<br>Across 9 European countries n=1
Ruchat *et al.* 2018 [34]	The purpose of this review was to evaluate the effect of prenatal exercise on GWG and PPWR	Bibliographic databases: MEDLINE, EMBASE, PsycINFO, Cochrane Database of Systematic Reviews, Scopus and Web of Science Core Collection, CINAHL Plus with Full-text, Child Development & Adolescent Studies, ERIC, Sport Discus, and the Trip Database<br>Supplementary searches: Cochrane Central Register of Controlled Trials, ClinicalTrials. gov	● Primary studies of any design except case studies, narrative syntheses and systematic reviews.<br>● Exercise only and exercise + co-intervention (results reported separately for diet/PA and PA only)	● Case studies<br>● Narrative syntheses<br>● Systematic reviews<br>● Published in language other than English, Spanish or French	● 84 studies<br>● Published: 1987 to 2017<br>● Study design: RCT n=79, non-randomised intervention studies n=4, cohort study n=1<br>● n=21,530 women	USA n=19<br>Sweden n=3<br>Canada n=9<br>Netherland n=2<br>Spain n=10<br>Italy n=2<br>Brazil n=5<br>Mexico n=3<br>Australia n=6<br>Norway n=3<br>Iran n=2<br>Belgium n=1<br>New Zealand n=2<br>Taiwan n=1<br>China n=3<br>Finland n=3<br>UK n=2<br>Germany n=1<br>Denmark n=2<br>India n=1<br>Iran n=1<br>Thailand n=1<br>Japan n=1<br>Croatia n=1
Syngelaki *et al.* 2019 [35]	To examine whether diet and/or exercise can prevent PE in overweight or obese pregnant women.	Bibliographic databases: PubMed, Embase, CINAHL, Web of Science<br>Supplementary searches: Cochrane CENTRAL, references, other reviews	● RCTs that evaluated the effect of diet and/or exercise on the risk of PE and hypertensive disorders of pregnancy (results reported separately for diet/PA, diet only and exercise only)	● Not reported	● 23 studies<br>● Published: 2000 to 2017<br>● Study design: RCT n=23<br>● n=7,236 women	Not reported

			● Overweight/ obese women			
Wang *et al.* 2019 [62]	To collect and reassess all the evidence regarding whether physical exercise and its characteristics can have an effect on maternal GWG.	Bibliographic databases: Pubmed, Embase and Cochrane Library<br>Supplementary searches: Hand search included trials bibliographies	● RCTs in pregnant women having PA such as aerobic exercises, strength training, walking, cycling, weight training compared with conventional medical care<br>● Outcome of maternal GWG during pregnant period	● Non RCT studies	● 23 studies<br>● Published: 1999 to 2017<br>● Study design: RCT n=23<br>● n=4,462 women	China n=1<br>Brazil n=2<br>Spain n=9<br>Japan n=1<br>USA n=4<br>Norway n=1<br>New Zealand n=1<br>Columbia n=1<br>Iran n=2<br>Sweden n=1
Poyatos-Leon *et al.* 2015 [63]	To determine the influence of physical exercise interventions on the mode of delivery.	Bibliographic databases: Cochrane Library Plus, Science Direct, EMBASE, PubMed, Web of Science<br>Supplementary searches: ClinicalTrials.gov, manual searches	● Published from January 1990 to December 2013.<br>● Spanish or English language<br>● population: healthy women, with low to moderate levels of PA, uncomplicated singleton pregnancies<br>● RCTs in which the control group received no type of physical exercise<br>● Supervised exercise programs<br>● Mode of delivery<br>● No restrictions on frequency, duration or type of training.	● Women at high risk of<br>● preterm delivery<br>● Women included in other exercise<br>● programs during pregnancy<br>● Women with contraindication for exercise identified by an obstetrician<br>● Women who were not planning to give birth in the same hospital obstetrics department in which the antenatal care during the pregnancy was provided	● 10 studies<br>● Published: 2009 to 2013<br>● Study design: RCT n=10<br>● n=3,160 women	Spain n=7<br>Brazil n=1<br>Norway n=1<br>USA n=1
Walker *et al.* 2018 [36]	To address compare the efficacy of all approaches that have been used to prevent excessive GWG (e.g., diet, PA, lifestyle, sleep, eHealth, and medical).	Bibliographic databases: MEDLINE, EMBASE, Cochrane Library, PsycInFO, CINAHL, Scopus, LILACS<br>Supplementary searches: reference lists of included trials and systematic reviews	● RCTs of interventions designed to prevent excessive GWG.<br>● Comparators were standard care, an alternate intervention, or placebo.<br>● Participants were women of all ages, ethnicities, and pre-pregnancy weight status with singleton pregnancies.	● Studies for women with pre-existing diabetes (type 1 or type 2) or GDM	● 89 studies<br>● Published: 2000 to 2018<br>● Study design: RCT n=89<br>● n=25,345 women	USA n=22<br>Australia n=11<br>Spain n=10<br>Italy n=5<br>Finland n=3<br>Egypt n=1<br>Turkey n=2<br>Sweden n=2<br>Brazil n=4<br>Ireland n=2<br>Denmark n=3<br>Argentina n=1<br>Canada n=4<br>Norway n=3<br>Iran n=1<br>Netherlands n=2<br>China n=3<br>Belgium n=2<br>Taiwan n=1<br>India n=1<br>UK n=3<br>Columbia n=1<br>Germany n=1<br>Multiple European countries n=1
Wiebe *et al.* 2015 [64]	To estimate the influence of structured prenatal exercise on newborn birth weight, macrosomia, and growth restriction.	Bibliographic databases: MEDLINE, EMBASE, CINAHL, Sport Discus, Ovid's All EBM Reviews database<br>Supplementary searches: ClinicalTrials.gov	● RCTs in pregnant women comparing standard prenatal care with standard care plus a supervised exercise (aerobic, resistance, or both).<br>● Any language of publication or publication format (eg, abstracts only).	● Interventions consisting solely of pelvic floor exercises, stretching, or relaxation	● 28 studies<br>● Published: 1997 to 2014<br>● Study design: RCT n=28<br>● n=5,322 women	USA n=4<br>Brazil n=4<br>Spain n=8<br>Norway n=2<br>New Zealand n=1<br>Iran n=2

						Canada n=2<br>Sweden n=1<br>Kosovo n=1<br>Netherlands n=1<br>Columbia n=1<br>Croatia n=1
Yin *et al.* 2013 [65]	To collect all the evidence available from RCTs regarding the association between physical exercise during pregnancy and the incidence of GDM.	<u>Bibliographic databases</u>: PUBMED, ISI Web of Knowledge, CBM, CNKI, VIP<br><u>Supplementary searches</u>: Cochrane CENTRAL Library, reference lists	● RCT<br>● Pregnant women without previous GDM and other complications.<br>● Increased physical activity was the only intervention.<br>● Incidence of GDM was documented separately for the control and intervention groups, with diagnosis criteria as defined in individual trials.	● Not reported	● 6 studies<br>● Published: 2009 to 2012<br>● Study design: RCT n=6<br>● n=1,278 women	USA n=1<br>Norway n=1<br>Australia n=1<br>Spain n=2<br>India n=1
Shieh *et al.* 2018 [38]	To analyse data from published studies using a RCT design on the prevention of excessive GWG specifically among pregnant overweight and obese women.	<u>Bibliographic databases</u>: PubMed, Academic Search Premier, CINAHL, MEDLINE, and PsyInfo<br><u>Supplementary searches</u>: None reported	● RCT<br>● Exercise, diet or both as the intervention<br>● Healthy overweight and/or obese pregnant adult women<br>● GWG an outcome	● Sub-study from RCTs<br>● Systematic reviews<br>● Meta-analyses<br>● Interventions to increase pregnancy weight gain<br>● Women with an identified health problem (e.g. AIDS, GDM).	● 23 studies<br>● Published: 2008 to 2016<br>● Study design: RCT n=23<br>● n=6,920 women	Belgium n=2<br>Denmark n=3<br>Norway n=1<br>UK n=1<br>Italy n=1<br>Australia n=6<br>USA n=5<br>New Zealand n=1<br>Canada n=1<br>Brazil n=1<br>Multiple European countries n=1
Bennett *et al.* 2017 [39]	To comprehensively evaluate the global impact of interventions designed to prevent excessive GWG on the incidence of GDM, and whether the effects differ by maternal BMI or ethnicity.	<u>Bibliographic databases</u>: Cochrane Library, MEDLINE, EMBASE, PsycINFO, CINAHL, Scopus, LILACS, CNKI, WangFang, VIP<br><u>Supplementary searches</u>: reference lists of systematic reviews	● RCTs conducted in humans<br>● Primary or secondary aim to reduce/prevent excessive GWG<br>● Reported on the incidence of GDM in both groups separately<br>● All languages and countries	● Studies in animals<br>● Studies conducted in women with existing<br>● Studies to encourage GWG<br>● Duplication populations	● 45 studies<br>● Published: 2002 to 2016<br>● Study design: RCT n=45<br>● n=15,293 women	Australia n=8<br>China n=8<br>USA n=7<br>Spain n=6<br>Canada n=3<br>Denmark n=3<br>Finland n=2<br>UK n=2<br>Ireland n=1<br>Netherlands n=1<br>Belgium n=1<br>Italy n=1<br>Norway n=1<br>Multiple European countries n=1

Abbreviations: PA=physical activity, RCT=randomised controlled trial, GDM=gestational diabetes mellitus, GWG=gestational weight gain, PE=preeclampsia, LGA=large for gestational age, PTB=preterm birth, PCOS=polycystic ovarian syndrome, LBW=low birth weight, BMI=body mass index, CAM=complementary and alternative medicine

S5 References:

1. Chamberlain C, O'Mara-Eves A, Porter J, Coleman T, Perlen SM, Thomas J, et al. Psychosocial interventions for supporting women to stop smoking in pregnancy. The Cochrane database of systematic reviews. 2017;2:Cd001055. Epub 2017/02/15. doi: 10.1002/14651858.CD001055.pub5. PubMed PMID: 28196405; PubMed Central PMCID: PMCPMC6472671.

2. Veisani Y, Jenabi E, Delpisheh A, Khazaei S. Effect of prenatal smoking cessation interventions on birth weight: meta-analysis. The journal of maternal-fetal & neonatal medicine : the official journal of the European Association of Perinatal Medicine, the Federation of Asia and Oceania Perinatal Societies, the International Society of Perinatal Obstet. 2019;32(2):332-8. Epub 2017/09/12. doi: 10.1080/14767058.2017.1378335. PubMed PMID: 28889768.
3. Agha M, Agha RA, Sandall J. Interventions to reduce and prevent obesity in pre-conceptual and pregnant women: a systematic review and meta-analysis. PloS one. 2014;9(5):e95132-e. doi: 10.1371/journal.pone.0095132. PubMed PMID: 24827704.
4. Bain E, Crane M, Tieu J, Han S, Crowther CA, Middleton P. Diet and exercise interventions for preventing gestational diabetes mellitus. The Cochrane database of systematic reviews. 2015;(4):Cd010443. Epub 2015/04/13. doi: 10.1002/14651858.CD010443.pub2. PubMed PMID: 25864059.
5. Allen R, Rogozinska E, Sivarajasingam P, Khan KS, Thangaratinam S. Effect of diet- and lifestyle-based metabolic risk-modifying interventions on preeclampsia: a meta-analysis. Acta Obstetricia et Gynecologica Scandinavica. 2014;93(10):973-85. doi: 10.1111/aogs.12467.
6. Campbell F, Johnson M, Messina J, Guillaume L, Goyder E. Behavioural interventions for weight management in pregnancy: a systematic review of quantitative and qualitative data. BMC Public Health. 2011;11:491. Epub 2011/06/24. doi: 10.1186/1471-2458-11-491. PubMed PMID: 21696589; PubMed Central PMCID: PMCPMC3154865.
7. Choi J, Fukuoka Y, Lee JH. The effects of physical activity and physical activity plus diet interventions on body weight in overweight or obese women who are pregnant or in postpartum: a systematic review and meta-analysis of randomized controlled trials. Prev Med. 2013;56(6):351-64. Epub 2013/02/26. doi: 10.1016/j.ypmed.2013.02.021. PubMed PMID: 23480971.
8. Gardner B, Wardle J, Poston L, Croker H. Changing diet and physical activity to reduce gestational weight gain: a meta-analysis. Obesity reviews : an official journal of the International Association for the Study of Obesity. 2011;12(7):e602-20. Epub 2011/04/28. doi: 10.1111/j.1467-789X.2011.00884.x. PubMed PMID: 21521451.
9. Hill B, Skouteris H, Fuller-Tyszkiewicz M. Interventions designed to limit gestational weight gain: a systematic review of theory and meta-analysis of intervention components. Obesity Reviews. 2013;14(6):435-50. doi: 10.1111/obr.12022.
10. Madhuvrata P, Govinden G, Bustani R, Song S, Farrell TA. Prevention of gestational diabetes in pregnant women with risk factors for gestational diabetes: a systematic review and meta-analysis of randomised trials. Obstet Med. 2015;8(2):68-85. Epub 2015/06/01. doi: 10.1177/1753495x15576673. PubMed PMID: 27512459; PubMed Central PMCID: PMCPMC4935009.
11. Muktabhant B, Lawrie TA, Lumbiganon P, Laopaiboon M. Diet or exercise, or both, for preventing excessive weight gain in pregnancy. The Cochrane database of systematic reviews. 2015;(6):Cd007145. Epub 2015/06/13. doi: 10.1002/14651858.CD007145.pub3. PubMed PMID: 26068707.
12. O'Brien CM, Grivell RM, Dodd JM. Systematic review of antenatal dietary and lifestyle interventions in women with a normal body mass index. Acta Obstet Gynecol Scand. 2016;95(3):259-69. Epub 2015/11/27. doi: 10.1111/aogs.12829. PubMed PMID: 26610169.
13. Oteng-Ntim E, Varma R, Croker H, Poston L, Doyle P. Lifestyle interventions for overweight and obese pregnant women to improve pregnancy outcome: systematic review and meta-analysis. BMC Medicine. 2012;10(1):47. doi: 10.1186/1741-7015-10-47.
14. Rogozińska E, Chamillard M, Hitman GA, Khan KS, Thangaratinam S. Nutritional manipulation for the primary prevention of gestational diabetes mellitus: a meta-analysis of randomised studies. PloS one. 2015;10(2):e0115526. Epub 2015/02/27. doi: 10.1371/journal.pone.0115526. PubMed PMID: 25719363; PubMed Central PMCID: PMCPMC4342242.
15. Tanentsapf I, Heitmann BL, Adegboye ARA. Systematic review of clinical trials on dietary interventions to prevent excessive weight gain during pregnancy among normal weight, overweight and obese women. BMC Pregnancy and Childbirth. 2011;11(1):81. doi: 10.1186/1471-2393-11-81.
16. Thangaratinam S, Rogozińska E, Jolly K, Glinkowski S, Duda W, Borowiack E, et al. Interventions to reduce or prevent obesity in pregnant women: a systematic review. 2012;16:31. doi: 10.3310/hta16310.
17. Thangaratinam S, Rogozińska E, Jolly K, Glinkowski S, Roseboom T, Tomlinson JW, et al. Effects of interventions in pregnancy on maternal weight and obstetric outcomes: meta-analysis of randomised evidence. BMJ : British Medical Journal. 2012;344:e2088. doi: 10.1136/bmj.e2088.
18. International Weight Management in Pregnancy (IWiP) collaboration. Effect of diet and physical activity based interventions in pregnancy on gestational weight gain and pregnancy outcomes: meta-analysis of individual participant data from randomised trials. BMJ (Clinical research ed). 2017;358:j3119. doi: 10.1136/bmj.j3119.
19. Lau Y, Klainin-Yobas P, Htun TP, Wong SN, Tan KL, Ho-Lim ST, et al. Electronic-based lifestyle interventions in overweight or obese perinatal women: a systematic review and meta-analysis. Obesity reviews : an official journal of the International Association for the Study of Obesity. 2017;18(9):1071-87. Epub 2017/05/26. doi: 10.1111/obr.12557. PubMed PMID: 28544551.
20. Magro-Malosso ER, Saccone G, Di Tommaso M, Roman A, Berghella V. Exercise during pregnancy and risk of gestational hypertensive disorders: a systematic review and meta-analysis. Acta Obstetricia et Gynecologica Scandinavica. 2017;96(8):921-31. doi: 10.1111/aogs.13151.
21. Magro-Malosso ER, Saccone G, Di Mascio D, Di Tommaso M, Berghella V. Exercise during pregnancy and risk of preterm birth in overweight and obese women: a systematic review and meta-analysis of randomized controlled trials. Acta Obstet Gynecol Scand. 2017;96(3):263-73. Epub 2016/12/29. doi: 10.1111/aogs.13087. PubMed PMID: 28029178.
22. Shepherd E, Gomersall JC, Tieu J, Han S, Crowther CA, Middleton P. Combined diet and exercise interventions for preventing gestational diabetes mellitus. The Cochrane database of systematic reviews. 2017;11:Cd010443. Epub 2017/11/13. doi: 10.1002/14651858.CD010443.pub3. PubMed PMID: 29129039; PubMed Central PMCID: PMCPMC6485974.

23. Sherifali D, Nerenberg KA, Wilson S, Semeniuk K, Ali MU, Redman LM, et al. The Effectiveness of eHealth Technologies on Weight Management in Pregnant and Postpartum Women: Systematic Review and Meta-Analysis. Journal of medical Internet research. 2017;19(10):e337. Epub 2017/10/17. doi: 10.2196/jmir.8006. PubMed PMID: 29030327; PubMed Central PMCID: PMCPMC5660296.
24. Song C, Li J, Leng J, Ma RC, Yang X. Lifestyle intervention can reduce the risk of gestational diabetes: a meta-analysis of randomized controlled trials. Obesity reviews : an official journal of the International Association for the Study of Obesity. 2016;17(10):960-9. Epub 2016/07/16. doi: 10.1111/obr.12442. PubMed PMID: 27417680.
25. Yeo S, Walker JS, Caughey MC, Ferraro AM, Asafu-Adjei JK. What characteristics of nutrition and physical activity interventions are key to effectively reducing weight gain in obese or overweight pregnant women? A systematic review and meta-analysis. Obesity reviews : an official journal of the International Association for the Study of Obesity. 2017;18(4):385-99. Epub 2017/02/09. doi: 10.1111/obr.12511. PubMed PMID: 28177566.
26. Craemer KA, Sampene E, Safdar N, Antony KM, Wautlet CK. Nutrition and Exercise Strategies to Prevent Excessive Pregnancy Weight Gain: A Meta-analysis. AJP Rep. 2019;9(1):e92-e120. Epub 2019/03/19. doi: 10.1055/s-0039-1683377. PubMed PMID: 31041118.
27. Davenport MH, Kathol AJ, Mottola MF, Skow RJ, Meah VL, Poitras VJ, et al. Prenatal exercise is not associated with fetal mortality: a systematic review and meta-analysis. Br J Sports Med. 2019;53(2):108-15. Epub 2018/10/20. doi: 10.1136/bjsports-2018-099773. PubMed PMID: 30337346.
28. Davenport MH, McCurdy AP, Mottola MF, Skow RJ, Meah VL, Poitras VJ, et al. Impact of prenatal exercise on both prenatal and postnatal anxiety and depressive symptoms: a systematic review and meta-analysis. British Journal of Sports Medicine. 2018;52(21):1376. doi: 10.1136/bjsports-2018-099697.
29. Davenport MH, Ruchat SM, Poitras VJ, Jaramillo Garcia A, Gray CE, Barrowman N, et al. Prenatal exercise for the prevention of gestational diabetes mellitus and hypertensive disorders of pregnancy: a systematic review and meta-analysis. Br J Sports Med. 2018;52(21):1367-75. Epub 2018/10/20. doi: 10.1136/bjsports-2018-099355. PubMed PMID: 30337463.
30. Davenport MH, Ruchat SM, Sobierajski F, Poitras VJ, Gray CE, Yoo C, et al. Impact of prenatal exercise on maternal harms, labour and delivery outcomes: a systematic review and meta-analysis. Br J Sports Med. 2019;53(2):99-107. Epub 2018/10/20. doi: 10.1136/bjsports-2018-099821. PubMed PMID: 30337349.
31. Davenport MH, Sobierajski F, Mottola MF, Skow RJ, Meah VL, Poitras VJ, et al. Glucose responses to acute and chronic exercise during pregnancy: a systematic review and meta-analysis. Br J Sports Med. 2018;52(21):1357-66. Epub 2018/10/20. doi: 10.1136/bjsports-2018-099829. PubMed PMID: 30337462.
32. Michel S, Raab R, Drabsch T, Günther J, Stecher L, Hauner H. Do lifestyle interventions during pregnancy have the potential to reduce long-term postpartum weight retention? A systematic review and meta-analysis. Obesity Reviews. 2019;20(4):527-42. doi: 10.1111/obr.12809.
33. Morison PN, Bacardi-Gascon M, Lopez-Corrales M, Jimenez-Cruz A. Combined dietary-exercise intervention for gestational weight gain and birthweight: a meta-analysis. Asia Pac J Clin Nutr. 2018;27(4):860-8. Epub 2018/07/27. doi: 10.6133/apjcn.112017.02. PubMed PMID: 30045432.
34. Ruchat S-M, Mottola MF, Skow RJ, Nagpal TS, Meah VL, James M, et al. Effectiveness of exercise interventions in the prevention of excessive gestational weight gain and postpartum weight retention: a systematic review and meta-analysis. British Journal of Sports Medicine. 2018;52(21):1347. doi: 10.1136/bjsports-2018-099399.
35. Syngelaki A, Sequeira Campos M, Roberge S, Andrade W, Nicolaides KH. Diet and exercise for preeclampsia prevention in overweight and obese pregnant women: systematic review and meta-analysis. The journal of maternal-fetal & neonatal medicine : the official journal of the European Association of Perinatal Medicine, the Federation of Asia and Oceania Perinatal Societies, the International Society of Perinatal Obstet. 2019;32(20):3495-501. Epub 2018/05/25. doi: 10.1080/14767058.2018.1481037. PubMed PMID: 29792061.
36. Walker R, Bennett C, Blumfield M, Gwini S, Ma J, Wang F, et al. Attenuating Pregnancy Weight Gain-What Works and Why: A Systematic Review and Meta-Analysis. Nutrients. 2018;10(7):944. doi: 10.3390/nu10070944. PubMed PMID: 30037126.
37. Vincze L, Rollo M, Hutchesson M, Hauck Y, MacDonald-Wicks L, Wood L, et al. Interventions including a nutrition component aimed at managing gestational weight gain or postpartum weight retention: a systematic review and meta-analysis. JBI Evidence Synthesis. 2019;17(3):297-364. doi: 10.11124/jbisrir-2017-003593. PubMed PMID: 01938924-201903000-00007.
38. Shieh C, Cullen DL, Pike C, Pressler SJ. Intervention strategies for preventing excessive gestational weight gain: systematic review and meta-analysis. Obesity Reviews. 2018;19(8):1093-109. doi: https://doi.org/10.1111/obr.12691.
39. Bennett CJ, Walker RE, Blumfield ML, Gwini S-M, Ma J, Wang F, et al. Interventions designed to reduce excessive gestational weight gain can reduce the incidence of gestational diabetes mellitus: A systematic review and meta-analysis of randomised controlled trials. Diabetes research and clinical practice. 2018;141:69-79. doi: 10.1016/j.diabres.2018.04.010. PubMed PMID: 29698713.
40. Gresham E, Bisquera A, Byles JE, Hure AJ. Effects of dietary interventions on pregnancy outcomes: a systematic review and meta-analysis. Matern Child Nutr. 2016;12(1):5-23. Epub 2014/07/23. doi: 10.1111/mcn.12142. PubMed PMID: 25048387; PubMed Central PMCID: PMCPMC6860081.
41. Oostdam N, van Poppel MN, Wouters MG, van Mechelen W. Interventions for preventing gestational diabetes mellitus: a systematic review and meta-analysis. Journal of women's health (2002). 2011;20(10):1551-63. Epub 2011/08/16. doi: 10.1089/jwh.2010.2703. PubMed PMID: 21838525.

42. Quinlivan JA, Julania S, Lam L. Antenatal dietary interventions in obese pregnant women to restrict gestational weight gain to Institute of Medicine recommendations: a meta-analysis. Obstet Gynecol. 2011;118(6):1395-401. Epub 2011/11/23. doi: 10.1097/AOG.0b013e3182396bc6. PubMed PMID: 22105270.
43. Tieu J, Shepherd E, Middleton P, Crowther CA. Dietary advice interventions in pregnancy for preventing gestational diabetes mellitus. The Cochrane database of systematic reviews. 2017;1:Cd006674. Epub 2017/01/04. doi: 10.1002/14651858.CD006674.pub3. PubMed PMID: 28046205; PubMed Central PMCID: PMCPMC6464792.
44. Zhang R, Han S, Chen GC, Li ZN, Silva-Zolezzi I, Parés GV, et al. Effects of low-glycemic-index diets in pregnancy on maternal and newborn outcomes in pregnant women: a meta-analysis of randomized controlled trials. Eur J Nutr. 2018;57(1):167-77. Epub 2016/09/11. doi: 10.1007/s00394-016-1306-x. PubMed PMID: 27612876.
45. Gresham E, Byles JE, Bisquera A, Hure AJ. Effects of dietary interventions on neonatal and infant outcomes: a systematic review and meta-analysis. Am J Clin Nutr. 2014;100(5):1298-321. Epub 2014/10/22. doi: 10.3945/ajcn.113.080655. PubMed PMID: 25332328.
46. Elliott-Sale KJ, Barnett CT, Sale C. Exercise interventions for weight management during pregnancy and up to 1 year postpartum among normal weight, overweight and obese women: a systematic review and meta-analysis. Br J Sports Med. 2015;49(20):1336-42. Epub 2014/11/20. doi: 10.1136/bjsports-2014-093875. PubMed PMID: 25406335.
47. Han S, Middleton P, Crowther CA. Exercise for pregnant women for preventing gestational diabetes mellitus. The Cochrane database of systematic reviews. 2012;(7):Cd009021. Epub 2012/07/13. doi: 10.1002/14651858.CD009021.pub2. PubMed PMID: 22786521.
48. Lamina S, Agbanusi E. Effect of aerobic exercise training on maternal weight gain in pregnancy: a meta-analysis of randomized controlled trials. Ethiop J Health Sci. 2013;23(1):59-64. PubMed PMID: 23559839.
49. Russo LM, Nobles C, Ertel KA, Chasan-Taber L, Whitcomb BW. Physical activity interventions in pregnancy and risk of gestational diabetes mellitus: a systematic review and meta-analysis. Obstet Gynecol. 2015;125(3):576-82. Epub 2015/03/03. doi: 10.1097/aog.0000000000000691. PubMed PMID: 25730218.
50. Sanabria-Martínez G, García-Hermoso A, Poyatos-León R, Álvarez-Bueno C, Sánchez-López M, Martínez-Vizcaíno V. Effectiveness of physical activity interventions on preventing gestational diabetes mellitus and excessive maternal weight gain: a meta-analysis. BJOG : an international journal of obstetrics and gynaecology. 2015;122(9):1167-74. Epub 2015/06/04. doi: 10.1111/1471-0528.13429. PubMed PMID: 26036300.
51. Streuling I, Beyerlein A, Rosenfeld E, Hofmann H, Schulz T, von Kries R. Physical activity and gestational weight gain: a meta-analysis of intervention trials. BJOG : an international journal of obstetrics and gynaecology. 2011;118(3):278-84. Epub 2010/12/08. doi: 10.1111/j.1471-0528.2010.02801.x. PubMed PMID: 21134106.
52. Sui Z, Grivell RM, Dodd JM. Antenatal exercise to improve outcomes in overweight or obese women: A systematic review. Acta Obstet Gynecol Scand. 2012;91(5):538-45. Epub 2012/01/11. doi: 10.1111/j.1600-0412.2012.01357.x. PubMed PMID: 22229625.
53. da Silva SG, Ricardo LI, Evenson KR, Hallal PC. Leisure-Time Physical Activity in Pregnancy and Maternal-Child Health: A Systematic Review and Meta-Analysis of Randomized Controlled Trials and Cohort Studies. Sports Med. 2017;47(2):295-317. Epub 2016/06/11. doi: 10.1007/s40279-016-0565-2. PubMed PMID: 27282925.
54. Di Mascio D, Magro-Malosso ER, Saccone G, Marhefka GD, Berghella V. Exercise during pregnancy in normal-weight women and risk of preterm birth: a systematic review and meta-analysis of randomized controlled trials. American journal of obstetrics and gynecology. 2016;215(5):561-71. Epub 2016/10/30. doi: 10.1016/j.ajog.2016.06.014. PubMed PMID: 27319364.
55. Sanabria-Martínez G, García-Hermoso A, Poyatos-León R, González-García A, Sánchez-López M, Martínez-Vizcaíno V. Effects of Exercise-Based Interventions on Neonatal Outcomes: A Meta-Analysis of Randomized Controlled Trials. Am J Health Promot. 2016;30(4):214-23. Epub 2016/07/13. doi: 10.1177/0890117116639569. PubMed PMID: 27404056.
56. Zheng J, Wang H, Ren M. Influence of exercise intervention on gestational diabetes mellitus: a systematic review and meta-analysis. J Endocrinol Invest. 2017;40(10):1027-33. Epub 2017/04/13. doi: 10.1007/s40618-017-0673-3. PubMed PMID: 28401529.
57. Chatzakis C, Goulis DG, Mareti E, Eleftheriades M, Zavlanos A, Dinas K, et al. Prevention of gestational diabetes mellitus in overweight or obese pregnant women: A network meta-analysis. Diabetes Res Clin Pract. 2019;158:107924. Epub 2019/11/19. doi: 10.1016/j.diabres.2019.107924. PubMed PMID: 31738997.
58. Du MC, Ouyang YQ, Nie XF, Huang Y, Redding SR. Effects of physical exercise during pregnancy on maternal and infant outcomes in overweight and obese pregnant women: A meta-analysis. Birth. 2019;46(2):211-21. Epub 2018/09/22. doi: 10.1111/birt.12396. PubMed PMID: 30240042.
59. Guillemette L, Hay JL, Kehler DS, Hamm NC, Oldfield C, McGavock JM, et al. Exercise in Pregnancy and Children's Cardiometabolic Risk Factors: a Systematic Review and Meta-Analysis. Sports Med Open. 2018;4(1):35-. doi: 10.1186/s40798-018-0148-x. PubMed PMID: 30069801.
60. Ming W-K, Ding W, Zhang CJP, Zhong L, Long Y, Li Z, et al. The effect of exercise during pregnancy on gestational diabetes mellitus in normal-weight women: a systematic review and meta-analysis. BMC Pregnancy and Childbirth. 2018;18(1):440. doi: 10.1186/s12884-018-2068-7.
61. Nasiri-Amiri F, Sepidarkish M, Shirvani MA, Habibipour P, Tabari NSM. The effect of exercise on the prevention of gestational diabetes in obese and overweight pregnant women: a systematic review and meta-analysis. Diabetology & Metabolic Syndrome. 2019;11(1):72. doi: 10.1186/s13098-019-0470-6.
62. Wang J, Wen D, Liu X, Liu Y. Impact of exercise on maternal gestational weight gain: An updated meta-analysis of randomized controlled trials. Medicine (Baltimore). 2019;98(27):e16199-e. doi: 10.1097/MD.0000000000016199. PubMed PMID: 31277127.

63. Poyatos-León R, García-Hermoso A, Sanabria-Martínez G, Álvarez-Bueno C, Sánchez-López M, Martínez-Vizcaíno V. Effects of exercise during pregnancy on mode of delivery: a meta-analysis. Acta Obstet Gynecol Scand. 2015;94(10):1039-47. Epub 2015/05/13. doi: 10.1111/aogs.12675. PubMed PMID: 25965378.
64. Wiebe HW, Boulé NG, Chari R, Davenport MH. The Effect of Supervised Prenatal Exercise on Fetal Growth: A Meta-analysis. Obstetrics & Gynecology. 2015;125(5):1185-94. doi: 10.1097/aog.0000000000000801. PubMed PMID: 00006250-201505000-00028.
65. Yin YN, Li XL, Tao TJ, Luo BR, Liao SJ. Physical activity during pregnancy and the risk of gestational diabetes mellitus: a systematic review and meta-analysis of randomised controlled trials. Br J Sports Med. 2014;48(4):290-5. Epub 2013/09/17. doi: 10.1136/bjsports-2013-092596. PubMed PMID: 24037671.

S6 Tables: Critical appraisal results for systematic reviews reporting outcomes for each behavioural domain, and overall quality for all included systematic reviews

S6a: Systematic reviews reporting effectiveness of smoking cessation interventions

Author, year	Quality assessment question[1]											Total score[2]	Quality[3]
	1	2	3	4	5	6	7	8	9	10	11		
Chamberlain *et al.* 2017 [1]	Yes	Yes	Yes	Yes	Yes	Unclear	Yes	Yes	Yes	Yes	Yes	10	High
Veisani *et al.* 2017 [2]	Yes	Yes	Yes	Yes	No	No	Yes	Yes	Yes	No	No	7	Moderate
**Subtotal**	**100% (2/2)**	**100% (2/2)**	**100% (2/2)**	**100% (2/2)**	**50% (1/2)**	**0% (0/2)**	**100% (2/2)**	**100% (2/2)**	**100% (2/2)**	**50% (1/2)**	**50% (1/2)**	**Range 7-10**	**50% moderate (1/2) 50% high (1/2)**

S6b: Systematic reviews reporting effectiveness of diet and/or physical activity interventions

Author, year	Quality assessment question[1]											Total score[2]	Quality[3]
	1	2	3	4	5	6	7	8	9	10	11		
Agha *et al.* 2014 [3]	Yes	Yes	Yes	Yes	Yes	Yes	Yes	Yes	Yes	Yes	Yes	11	High
Bain *et al.* 2015 [4]	Yes	Yes	Yes	Yes	Yes	Yes	Yes	Yes	Yes	Yes	Yes	11	High
Allen *et al.* 2014 [5]	Yes	Yes	Yes	No	Yes	Yes	No	Yes	No	Yes	Yes	7	Moderate
Campbell *et al.* 2011 [6]	Yes	Yes	Yes	Yes	Yes	Yes	Yes	Yes	No	Yes	Yes	9	High
Choi *et al.* 2013 [7]	Yes	Yes	Yes	Yes	Yes	Yes	Unclear	Yes	Yes	Yes	Yes	10	High
Gardner *et al.* 2011 [8]	Yes	Yes	No	Yes	Yes	No	Yes	Yes	Yes	Yes	Yes	9	High
Hill *et al.* 2013 [9]	Yes	Yes	Yes	Yes	Yes	Yes	Unclear	Yes	No	Yes	Yes	9	High
Madhuvrata *et al.* 2015 [10]	Yes	Yes	Yes	Yes	Yes	Yes	Yes	Yes	Yes	Yes	Yes	11	High
Muktabhant *et al.* 2015 [11]	Yes	Yes	Yes	Yes	Yes	Yes	Yes	Yes	Yes	Yes	Yes	11	High
O'Brien *et al.* 2015 [12]	Yes	Yes	Yes	No	Yes	Yes	Yes	Yes	No	Yes	Yes	9	High
Oteng-Ntim *et al.* 2012 [13]	Yes	Yes	Yes	Yes	Yes	Yes	Yes	Yes	Yes	Yes	Yes	11	High
Rogozińska *et al.* 2015 [14]	Yes	Yes	Yes	Yes	Yes	Yes	Yes	Yes	Yes	Yes	Yes	11	High
Tanentsapf *et al.* 2011 [15]	Yes	Yes	Yes	Yes	Yes	Yes	Yes	Yes	Yes	Yes	Yes	11	High
Thangaratinam *et al.* 2012a [16]	Yes	Yes	Yes	Yes	Yes	Unclear	Yes	Yes	Yes	Yes	Yes	10	High
Thangaratinam *et al.* 2012b [17]	Yes	No	No	Yes	Yes	Unclear	Yes	Yes	Yes	Yes	Yes	8	High
IWiP 2017 [18]	Yes	No	Yes	No	Yes	Yes	Yes	Yes	Yes	Yes	Yes	9	High
Lau *et al.* 2017 [19]	Yes	Yes	Yes	Yes	Yes	Yes	Yes	Yes	Yes	Yes	Yes	11	High
Magro-Malosso *et al* 2017a [20]	Yes	Yes	Yes	No	Yes	Yes	Yes	Yes	Yes	Yes	No	8	High
Magro-Malosso *et al* 2017b [21]	Yes	Yes	Yes	No	Yes	Yes	Yes	Yes	No	Yes	No	8	High
Shepherd *et al.* 2017 [22]	Yes	Yes	Yes	Yes	Yes	Yes	Yes	Yes	Yes	Yes	Yes	11	High
Sherifali *et al.* 2017 [23]	Yes	Yes	Yes	Yes	Yes	Unclear	Yes	Yes	No	Yes	Yes	9	High
Song *et al.* 2016 [24]	Yes	Yes	Yes	Yes	Yes	Yes	Yes	Yes	Yes	Yes	Yes	11	High
Yeo *et al.* 2017 [25]	Yes	Yes	Yes	Yes	Yes	Yes	Yes	Yes	Yes	Yes	Unclear	10	High
Craemer *et al.* 2019 [26]	Yes	No	Yes	No	No	No	No	Yes	Yes	Yes	Yes	6	Moderate
Davenport *et al.* 2019a [27]	Yes	Yes	Yes	Yes	Yes	No	Yes	Yes	No	No	Yes	8	High
Davenport *et al.* 2018a [28]	Yes	Yes	Yes	Yes	Yes	No	Yes	Yes	Yes	Yes	No	9	High
Davenport *et al.* 2018b [29]	Yes	Yes	Yes	No	Yes	No	Yes	Yes	Yes	Yes	Yes	9	High
Davenport *et al.* 2019b [30]	Yes	Yes	Yes	No	Yes	Unclear	Yes	Yes	No	No	Yes	8	High
Davenport *et al.* 2018c [31]	Yes	No	No	Yes	Yes	Yes	Yes	Yes	Unclear	Yes	Yes	8	High

Author, year	Quality assessment question[1]											Total score[2]	Quality[3]
	1	2	3	4	5	6	7	8	9	10	11		
Michel S *et al.* 2019 [32]	Yes	Yes	Yes	Yes	Yes	Yes	Yes	Yes	Yes	Yes	Yes	11	High
Morison *et al.* 2018 [33]	Yes	No	Yes	Yes	No	Yes	Yes	Yes	Yes	Unclear	No	7	Moderate
Ruchat *et al.* 2018 [34]	Yes	Yes	Yes	Yes	Yes	Yes	Yes	Yes	Yes	Yes	Yes	11	High
Syngelaki *et al.* 2019 [35]	Yes	Yes	Yes	Yes	Yes	Yes	Yes	Yes	Yes	No	No	9	High
Walker *et al.* 2018 [36]	Yes	Yes	Yes	Yes	Yes	Yes	Unclear	Yes	Yes	Yes	Yes	10	High
Vincze *et al.* 2019 [37]	Yes	Yes	Yes	Yes	Yes	Yes	Yes	Yes	No	Yes	Yes	10	High
Shieh *et al.* 2018 [38]	Yes	Unclear	Yes	No	Yes	Yes	Yes	Yes	No	Yes	Yes	8	High
Bennett *et al.* 2017 [39]	Yes	Yes	Yes	Yes	Yes	Yes	Yes	Yes	Yes	Unclear	Yes	10	High
**Subtotal**	**100% (37/37)**	**84% (31/37)**	**92% (34/37)**	**76% (28/37)**	**95% (35/37)**	**76% (28/37)**	**86% (32/37)**	**100% (37/37)**	**71% (26/37)**	**86% (32/37)**	**84% (31/37)**	**Range 6-11**	**8% moderate (3/37) 92% high (34/37)**

S6c: Systematic reviews reporting effectiveness of diet only interventions

Author, year	Quality assessment question[1]											Total score[2]	Quality[3]
	1	2	3	4	5	6	7	8	9	10	11		
Allen *et al.* 2014 [5]	Yes	Yes	Yes	No	Yes	Yes	No	Yes	No	Yes	Yes	7	Moderate
Gresham *et al.* 2016 [40]	Yes	Yes	Yes	Yes	Yes	Yes	Yes	Yes	Yes	Yes	Yes	11	High
Madhuvrata *et al.* 2015 [10]	Yes	Yes	Yes	Yes	Yes	Yes	Yes	Yes	Yes	Yes	Yes	11	High
Muktabhant *et al.* 2015 [11]	Yes	Yes	Yes	Yes	Yes	Yes	Yes	Yes	Yes	Yes	Yes	11	High
Oostdam *et al.* 2011 [41]	Yes	Yes	Yes	Yes	Yes	Yes	Yes	Yes	Yes	Yes	Yes	11	High
Quinlivan *et al.* 2011 [42]	Yes	Yes	Unclear	Yes	No	Unclear	Yes	Yes	Yes	Yes	No	7	Moderate
Rogozińska *et al.* 2015 [14]	Yes	Yes	Yes	Yes	Yes	Yes	Yes	Yes	Yes	Yes	Yes	11	High
Thangaratinam *et al.* 2012a [16]	Yes	Yes	Yes	Yes	Yes	Unclear	Yes	Yes	Yes	Yes	Yes	10	High
Thangaratinam *et al.* 2012b [17]	Yes	No	No	Yes	Yes	Unclear	Yes	Yes	Yes	Yes	Yes	8	High
IWiP 2017 [18]	Yes	No	Yes	No	Yes	Yes	Yes	Yes	Yes	Yes	Yes	9	High
Song *et al.* 2016 [24]	Yes	Yes	Yes	Yes	Yes	Yes	Yes	Yes	Yes	Yes	Yes	11	High
Tieu *et al.* 2017 [43]	Yes	Yes	Yes	Yes	Yes	Yes	Yes	Yes	Yes	Yes	Yes	11	High
Zhang *et al.* 2018 [44]	Yes	Unclear	Yes	Yes	Yes	Yes	Yes	Yes	Yes	Yes	Yes	10	High
Gresham *et al.* 2014 [45]	Yes	Yes	Yes	Yes	Yes	Yes	Yes	Yes	Yes	Yes	Yes	11	High
Craemer *et al.* 2019 [26]	Yes	No	Yes	No	No	No	No	Yes	Yes	Yes	Yes	6	Moderate
Syngelaki *et al.* 2019 [35]	Yes	Yes	Yes	Yes	Yes	Yes	Yes	Yes	Yes	No	No	9	High
Walker *et al.* 2018 [36]	Yes	Yes	Yes	Yes	Yes	Yes	Unclear	Yes	Yes	Yes	Yes	10	High
Shieh *et al.* 2018 [38]	Yes	Unclear	Yes	No	Yes	Yes	Yes	Yes	No	Yes	Yes	8	High
Bennett *et al.* 2017 [39]	Yes	Yes	Yes	Yes	Yes	Yes	Yes	Yes	Yes	Unclear	Yes	10	High
**Subtotal**	**100% (19/19)**	**74% (14/19)**	**89% (17/19)**	**79% (15/19)**	**89% (17/19)**	**79% (15/19)**	**84% (16/19)**	**100% (19/19)**	**89% (17/19)**	**89% (17/19)**	**89% (17/19)**	**Range 6-11**	**16% moderate (3/19) 84% high (16/19)**

S6d: Systematic reviews reporting effectiveness of physical activity only interventions

Author, year	Quality assessment question[1]											Total score[2]	Quality[3]
	1	2	3	4	5	6	7	8	9	10	11		
Elliot-Sale *et al.* 2015 [46]	Yes	Yes	Yes	Yes	No	Yes	Unclear	Yes	No	Yes	Yes	7	Moderate
Han *et al.* 2012 [47]	Yes	Yes	Yes	Yes	Yes	Yes	Yes	Yes	No	Yes	Yes	10	High
Lamina & Agbanusi 2013 [48]	Yes	Yes	Yes	Yes	Yes	Yes	No	Yes	No	Yes	Yes	9	High
Madhuvrata *et al.* 2015 [10]	Yes	Yes	Yes	Yes	Yes	Yes	Yes	Yes	Yes	Yes	Yes	11	High
Oostdam *et al.* 2011 [41]	Yes	Yes	Yes	Yes	Yes	Yes	Yes	Yes	Yes	Yes	Yes	11	High
Russo *et al.* 2015 [49]	Yes	Yes	Yes	Yes	No	Unclear	Yes	Yes	Yes	Yes	Yes	9	High
Sanabria-Martínez *et al.* 2015 [50]	Yes	Yes	Yes	Yes	Yes	Unclear	Unclear	Yes	Yes	Yes	No	8	Moderate
Streuling *et al.* 2011 [51]	Yes	Yes	Yes	Yes	Yes	Yes	No	Yes	Yes	Yes	Yes	10	High
Sui *et al.* 2012 [52]	Yes	Yes	Yes	Yes	Yes	Yes	Yes	Yes	No	Yes	Yes	10	High
Thangaratinam *et al.* 2012a [16]	Yes	Yes	Yes	Yes	Yes	Unclear	Yes	Yes	Yes	Yes	Yes	10	High
Thangaratinam *et al.* 2012b [17]	Yes	No	No	Yes	Yes	Unclear	Yes	Yes	Yes	Yes	Yes	8	High
da Silva *et al.* 2017 [53]	Yes	Yes	Yes	Yes	Yes	Yes	Yes	Yes	No	Yes	Yes	10	High
Di Mascio *et al.* 2016 [54]	Yes	Yes	Yes	No	Yes	Unclear	Unclear	Yes	Yes	Yes	No	7	Moderate
IWiP 2017 [18]	Yes	No	Yes	No	Yes	Yes	Yes	Yes	Yes	Yes	Yes	9	High
Sanabria-Martínez *et al.* 2016 [55]	Yes	Yes	Yes	Yes	Yes	Yes	Yes	Yes	Yes	Yes	No	9	High
Song *et al.* 2016 [24]	Yes	Yes	Yes	Yes	Yes	Yes	Yes	Yes	Yes	Yes	Yes	11	High
Zheng *et al.* 2017 [56]	Yes	Yes	Yes	Yes	Yes	Yes	Yes	Yes	No	Yes	Yes	10	High
Chatzakis *et al*. 2019 [57]	Yes	Yes	Yes	Yes	Yes	Unclear	Yes	Yes	Yes	Unclear	Unclear	8	High
Craemer *et al.* 2019 [26]	Yes	No	Yes	No	No	No	No	Yes	Yes	Yes	Yes	6	Moderate
Davenport *et al.* 2019a [27]	Yes	Yes	Yes	Yes	Yes	No	Yes	Yes	No	No	Yes	8	High
Davenport *et al.* 2018a [28]	Yes	Yes	Yes	Yes	Yes	No	Yes	Yes	Yes	Yes	No	9	High
Davenport *et al.* 2018b [29]	Yes	Yes	Yes	No	Yes	No	Yes	Yes	Yes	Yes	Yes	9	High
Davenport *et al.* 2019b [30]	Yes	Yes	Yes	No	Yes	Unclear	Yes	Yes	No	No	Yes	8	High
Davenport *et al.* 2018c [31]	Yes	No	No	Yes	Yes	Yes	Yes	Yes	Unclear	Yes	Yes	8	High
Du *et al*. 2019 [58]	Yes	Yes	Yes	Yes	Yes	No	Yes	Yes	Yes	Yes	Yes	10	High
Guillemette *et al.* 2018 [59]	Yes	No	Yes	Yes	No	Yes	Yes	Yes	Yes	Unclear	No	7	Moderate
Ming *et al.* 2018 [60]	Yes	Yes	Yes	Yes	Yes	No	Yes	Yes	Yes	Yes	Yes	10	High
Nasiri-Amiri *et al.* 2019 [61]	Yes	Yes	Yes	Yes	Yes	Yes	Yes	Yes	Yes	Yes	Yes	11	High
Ruchat *et al.* 2018 [34]	Yes	Yes	Yes	Yes	Yes	Yes	Yes	Yes	Yes	Yes	Yes	11	High
Syngelaki *et al.* 2019 [35]	Yes	Yes	Yes	Yes	Yes	Yes	Yes	Yes	Yes	No	No	9	High
Wang *et al.* 2019 [62]	Yes	Yes	Yes	Yes	Yes	Yes	Yes	Yes	Yes	No	Yes	10	High
Poyatos-Leon *et al.* 2015 [63]	Yes	Yes	Yes	Yes	Yes	Yes	Yes	Yes	Yes	No	No	9	High
Walker *et al.* 2018 [36]	Yes	Yes	Yes	Yes	Yes	Yes	Unclear	Yes	Yes	Yes	Yes	10	High
Wiebe *et al.* 2015 [64]	Yes	Yes	Yes	Yes	Yes	Unclear	Yes	Yes	No	Yes	Yes	9	High
Yin *et al.* 2013 [65]	Yes	Unclear	Yes	Yes	Yes	Unclear	Yes	Yes	Yes	Yes	Yes	9	High
Shieh *et al.* 2018 [38]	Yes	Unclear	Yes	No	Yes	Yes	Yes	Yes	No	Yes	Yes	8	High
Bennett *et al.* 2017 [39]	Yes	Yes	Yes	Yes	Yes	Yes	Yes	Yes	Yes	Unclear	Yes	10	High
**Subtotal**	**100% (37/37)**	**81% (30/37)**	**95% (35/37)**	**84% (31/37)**	**89% (33/37)**	**59% (22/37)**	**81% (30/37)**	**100% (37/37)**	**70% (26/37)**	**78% (29/37)**	**78% (29/37)**	**Range 6-11**	**14% moderate (5/37) 86% high (32/37)**

S6e: All included systematic reviews

Author, year	Quality assessment question[1]											Total score[2]	Quality[3]
	1	2	3	4	5	6	7	8	9	10	11		
Agha *et al.* 2014 [3]	Yes	Yes	Yes	Yes	Yes	Yes	Yes	Yes	Yes	Yes	Yes	11	High
Bain *et al.* 2015 [4]	Yes	Yes	Yes	Yes	Yes	Yes	Yes	Yes	Yes	Yes	Yes	11	High
Allen *et al.* 2014 [5]	Yes	Yes	Yes	No	Yes	Yes	No	Yes	No	Yes	Yes	7	Moderate
Campbell *et al.* 2011 [6]	Yes	Yes	Yes	Yes	Yes	Yes	Yes	Yes	No	Yes	Yes	9	High
Choi *et al.* 2013 [7]	Yes	Yes	Yes	Yes	Yes	Yes	Unclear	Yes	Yes	Yes	Yes	10	High
Elliot-Sale *et al.* 2015 [46]	Yes	Yes	Yes	Yes	No	Yes	Unclear	Yes	No	Yes	Yes	7	Moderate
Gardner *et al.* 2011 [8]	Yes	Yes	No	Yes	Yes	No	Yes	Yes	Yes	Yes	Yes	9	High
Gresham *et al.* 2016 [40]	Yes	Yes	Yes	Yes	Yes	Yes	Yes	Yes	Yes	Yes	Yes	11	High
Han *et al.* 2012 [47]	Yes	Yes	Yes	Yes	Yes	Yes	Yes	Yes	No	Yes	Yes	10	High
Hill *et al.* 2013 [9]	Yes	Yes	Yes	Yes	Yes	Yes	Unclear	Yes	No	Yes	Yes	9	High
Lamina & Agbanusi 2013 [48]	Yes	Yes	Yes	Yes	Yes	Yes	No	Yes	No	Yes	Yes	9	High
Madhuvrata *et al.* 2015 [10]	Yes	Yes	Yes	Yes	Yes	Yes	Yes	Yes	Yes	Yes	Yes	11	High
Muktabhant *et al.* 2015 [11]	Yes	Yes	Yes	Yes	Yes	Yes	Yes	Yes	Yes	Yes	Yes	11	High
O'Brien *et al.* 2015 [12]	Yes	Yes	Yes	No	Yes	Yes	Yes	Yes	No	Yes	Yes	9	High
Oostdam *et al.* 2011 [41]	Yes	Yes	Yes	Yes	Yes	Yes	Yes	Yes	Yes	Yes	Yes	11	High
Oteng-Ntim *et al.* 2012 [13]	Yes	Yes	Yes	Yes	Yes	Yes	Yes	Yes	Yes	Yes	Yes	11	High
Quinlivan *et al.* 2011 [42]	Yes	Yes	Unclear	Yes	No	Unclear	Yes	Yes	Yes	Yes	No	7	Moderate
Rogozińska *et al.* 2015 [14]	Yes	Yes	Yes	Yes	Yes	Yes	Yes	Yes	Yes	Yes	Yes	11	High
Russo *et al.* 2015 [49]	Yes	Yes	Yes	Yes	No	Unclear	Yes	Yes	Yes	Yes	Yes	9	High
Sanabria-Martínez *et al.* 2015 [50]	Yes	Yes	Yes	Yes	Yes	Unclear	Unclear	Yes	Yes	Yes	No	8	Moderate
Streuling *et al.* 2011 [51]	Yes	Yes	Yes	Yes	Yes	Yes	No	Yes	Yes	Yes	Yes	10	High
Sui *et al.* 2012 [52]	Yes	Yes	Yes	Yes	Yes	Yes	Yes	Yes	No	Yes	Yes	10	High
Tanentsapf *et al.* 2011 [15]	Yes	Yes	Yes	Yes	Yes	Yes	Yes	Yes	Yes	Yes	Yes	11	High
Thangaratinam *et al.* 2012a [16]	Yes	Yes	Yes	Yes	Yes	Unclear	Yes	Yes	Yes	Yes	Yes	10	High
Thangaratinam *et al.* 2012b [17]	Yes	No	No	Yes	Yes	Unclear	Yes	Yes	Yes	Yes	Yes	8	High
Chamberlain *et al.* 2017 [1]	Yes	Yes	Yes	Yes	Yes	Unclear	Yes	Yes	Yes	Yes	Yes	10	High
da Silva *et al.* 2017 [53]	Yes	Yes	Yes	Yes	Yes	Yes	Yes	Yes	No	Yes	Yes	10	High
Di Mascio *et al.* 2016 [54]	Yes	Yes	Yes	No	Yes	Unclear	Unclear	Yes	Yes	Yes	No	7	Moderate
IWiP 2017 [18]	Yes	No	Yes	No	Yes	Yes	Yes	Yes	Yes	Yes	Yes	9	High
Lau *et al.* 2017 [19]	Yes	Yes	Yes	Yes	Yes	Yes	Yes	Yes	Yes	Yes	Yes	11	High
Magro-Malosso *et al* 2017a [20]	Yes	Yes	Yes	No	Yes	Yes	Yes	Yes	Yes	Yes	No	8	High
Magro-Malosso *et al* 2017b [21]	Yes	Yes	Yes	No	Yes	Yes	Yes	Yes	No	Yes	No	8	High
Sanabria-Martínez *et al.* 2016 [55]	Yes	Yes	Yes	Yes	Yes	Yes	Yes	Yes	Yes	Yes	No	9	High
Shepherd *et al.* 2017 [22]	Yes	Yes	Yes	Yes	Yes	Yes	Yes	Yes	Yes	Yes	Yes	11	High
Sherifali *et al.* 2017 [23]	Yes	Yes	Yes	Yes	Yes	Unclear	Yes	Yes	No	Yes	Yes	9	High
Song *et al.* 2016 [24]	Yes	Yes	Yes	Yes	Yes	Yes	Yes	Yes	Yes	Yes	Yes	11	High
Tieu *et al.* 2017 [43]	Yes	Yes	Yes	Yes	Yes	Yes	Yes	Yes	Yes	Yes	Yes	11	High
Veisani *et al.* 2017 [2]	Yes	Yes	Yes	Yes	No	No	Yes	Yes	Yes	No	No	7	Moderate
Yeo *et al.* 2017 [25]	Yes	Yes	Yes	Yes	Yes	Yes	Yes	Yes	Yes	Yes	Unclear	10	High
Zhang *et al.* 2018 [44]	Yes	Unclear	Yes	Yes	Yes	Yes	Yes	Yes	Yes	Yes	Yes	10	High

Author, year	Quality assessment question[1]											Total score[2]	Quality[3]
	1	2	3	4	5	6	7	8	9	10	11		
Zheng *et al.* 2017 [56]	Yes	Yes	Yes	Yes	Yes	Yes	Yes	Yes	No	Yes	Yes	10	High
Gresham *et al.* 2014 [45]	Yes	Yes	Yes	Yes	Yes	Yes	Yes	Yes	Yes	Yes	Yes	11	High
Chatzakis *et al*. 2019 [57]	Yes	Yes	Yes	Yes	Yes	Unclear	Yes	Yes	Yes	Unclear	Unclear	8	High
Craemer *et al.* 2019 [26]	Yes	No	Yes	No	No	No	No	Yes	Yes	Yes	Yes	6	Moderate
Davenport *et al.* 2019a [27]	Yes	Yes	Yes	Yes	Yes	No	Yes	Yes	No	No	Yes	8	High
Davenport *et al.* 2018a [28]	Yes	Yes	Yes	Yes	Yes	No	Yes	Yes	Yes	Yes	No	9	High
Davenport *et al.* 2018b [29]	Yes	Yes	Yes	No	Yes	No	Yes	Yes	Yes	Yes	Yes	9	High
Davenport *et al.* 2019b [30]	Yes	Yes	Yes	No	Yes	Unclear	Yes	Yes	No	No	Yes	8	High
Davenport *et al.* 2018c [31]	Yes	No	No	Yes	Yes	Yes	Yes	Yes	Unclear	Yes	Yes	8	High
Du *et al*. 2019 [58]	Yes	Yes	Yes	Yes	Yes	No	Yes	Yes	Yes	Yes	Yes	10	High
Guillemette *et al.* 2018 [59]	Yes	No	Yes	Yes	No	Yes	Yes	Yes	Yes	Unclear	No	7	Moderate
Michel *et al.* 2019 [32]	Yes	Yes	Yes	Yes	Yes	Yes	Yes	Yes	Yes	Yes	Yes	11	High
Ming *et al.* 2018 [60]	Yes	Yes	Yes	Yes	Yes	No	Yes	Yes	Yes	Yes	Yes	10	High
Morison *et al.* 2018 [33]	Yes	No	Yes	Yes	No	Yes	Yes	Yes	Yes	Unclear	No	7	Moderate
Nasiri-Amiri *et al.* 2019 [61]	Yes	Yes	Yes	Yes	Yes	Yes	Yes	Yes	Yes	Yes	Yes	11	High
Ruchat *et al.* 2018 [34]	Yes	Yes	Yes	Yes	Yes	Yes	Yes	Yes	Yes	Yes	Yes	11	High
Syngelaki *et al.* 2019 [35]	Yes	Yes	Yes	Yes	Yes	Yes	Yes	Yes	Yes	No	No	9	High
Wang *et al.* 2019 [62]	Yes	Yes	Yes	Yes	Yes	Yes	Yes	Yes	Yes	No	Yes	10	High
Poyatos-Leon *et al.* 2015 [63]	Yes	Yes	Yes	Yes	Yes	Yes	Yes	Yes	Yes	No	No	9	High
Walker *et al.* 2018 [36]	Yes	Yes	Yes	Yes	Yes	Yes	Unclear	Yes	Yes	Yes	Yes	10	High
Vincze *et al.* 2019 [37]	Yes	Yes	Yes	Yes	Yes	Yes	Yes	Yes	No	Yes	Yes	10	High
Wiebe *et al.* 2015 [64]	Yes	Yes	Yes	Yes	Yes	Unclear	Yes	Yes	No	Yes	Yes	9	High
Yin *et al.* 2013 [65]	Yes	Unclear	Yes	Yes	Yes	Unclear	Yes	Yes	Yes	Yes	Yes	9	High
Shieh *et al.* 2018 [38]	Yes	Unclear	Yes	No	Yes	Yes	Yes	Yes	No	Yes	Yes	8	High
Bennett *et al.* 2017 [39]	Yes	Yes	Yes	Yes	Yes	Yes	Yes	Yes	Yes	Unclear	Yes	10	High
**Subtotal**	**100% (65/65)**	**86% (56/65)**	**94% (61/65)**	**85% (55/65)**	**89% (58/65)**	**69% (45/65)**	**85% (55/65)**	**100% (65/65)**	**72% (47/65)**	**85% (55/65)**	**78% (51/65)**	**Range 6-11**	**12% moderate (8/65) 88% high (57/65)**

Note: some of the same systematic reviews were included in diet and/or physical activity, diet only and physical activity only summaries depending on whether they reported data for all or a combination of intervention types, whereas the total quality summary for all included systematic reviews only includes each systematic review once (out of a total of 66 included reviews).

[1]Quality assessment questions using the JBI critical appraisal checklist 1) Is the review question clearly and explicitly stated?; 2) Were the inclusion criteria appropriate for the review question?; 3) Was the search strategy appropriate?; 4) Were the sources and resources used to search for studies adequate?; 5) Were the criteria for appraising studies appropriate?; 6) Was critical appraisal conducted by two or more reviewers independently?; 7) Were there methods to minimize errors in data extraction?; 8) Were the methods used to combine studies appropriate?; 9) Was the likelihood of publication bias assessed?; 10) Were recommendations for policy and/or practice supported by the reported data?; 11) Were the specific directives for new research appropriate?

[2]For total score 1 is given if yes otherwise it is zero.

[3]For quality: Low quality is 0-3. Moderate quality is 4-7. High quality is 8-11.

S6 References:

1. Chamberlain, C., et al., *Psychosocial interventions for supporting women to stop smoking in pregnancy.* Cochrane Database Syst Rev, 2017. **2**: p. Cd001055.
2. Veisani, Y., et al., *Effect of prenatal smoking cessation interventions on birth weight: meta-analysis.* J Matern Fetal Neonatal Med, 2019. **32**(2): p. 332-338.
3. Agha, M., R.A. Agha, and J. Sandall, *Interventions to reduce and prevent obesity in pre-conceptual and pregnant women: a systematic review and meta-analysis.* PloS one, 2014. **9**(5): p. e95132-e95132.
4. Bain, E., et al., *Diet and exercise interventions for preventing gestational diabetes mellitus.* Cochrane Database Syst Rev, 2015(4): p. Cd010443.
5. Allen, R., et al., *Effect of diet- and lifestyle-based metabolic risk-modifying interventions on preeclampsia: a meta-analysis.* Acta Obstetricia et Gynecologica Scandinavica, 2014. **93**(10): p. 973-985.
6. Campbell, F., et al., *Behavioural interventions for weight management in pregnancy: a systematic review of quantitative and qualitative data.* BMC Public Health, 2011. **11**: p. 491.
7. Choi, J., Y. Fukuoka, and J.H. Lee, *The effects of physical activity and physical activity plus diet interventions on body weight in overweight or obese women who are pregnant or in postpartum: a systematic review and meta-analysis of randomized controlled trials.* Preventive medicine, 2013. **56**(6): p. 351-364.
8. Gardner, B., et al., *Changing diet and physical activity to reduce gestational weight gain: a meta-analysis.* Obes Rev, 2011. **12**(7): p. e602-20.
9. Hill, B., H. Skouteris, and M. Fuller-Tyszkiewicz, *Interventions designed to limit gestational weight gain: a systematic review of theory and meta-analysis of intervention components.* Obesity Reviews, 2013. **14**(6): p. 435-450.
10. Madhuvrata, P., et al., *Prevention of gestational diabetes in pregnant women with risk factors for gestational diabetes: a systematic review and meta-analysis of randomised trials.* Obstet Med, 2015. **8**(2): p. 68-85.
11. Muktabhant, B., et al., *Diet or exercise, or both, for preventing excessive weight gain in pregnancy.* Cochrane Database Syst Rev, 2015(6): p. Cd007145.
12. O'Brien, C.M., R.M. Grivell, and J.M. Dodd, *Systematic review of antenatal dietary and lifestyle interventions in women with a normal body mass index.* Acta Obstet Gynecol Scand, 2016. **95**(3): p. 259-69.
13. Oteng-Ntim, E., et al., *Lifestyle interventions for overweight and obese pregnant women to improve pregnancy outcome: systematic review and meta-analysis.* BMC Medicine, 2012. **10**(1): p. 47.
14. Rogozińska, E., et al., *Nutritional manipulation for the primary prevention of gestational diabetes mellitus: a meta-analysis of randomised studies.* PLoS One, 2015. **10**(2): p. e0115526.
15. Tanentsapf, I., B.L. Heitmann, and A.R.A. Adegboye, *Systematic review of clinical trials on dietary interventions to prevent excessive weight gain during pregnancy among normal weight, overweight and obese women.* BMC Pregnancy and Childbirth, 2011. **11**(1): p. 81.
16. Thangaratinam, S., et al., *Interventions to reduce or prevent obesity in pregnant women: a systematic review.* 2012. **16**: p. 31.
17. Thangaratinam, S., et al., *Effects of interventions in pregnancy on maternal weight and obstetric outcomes: meta-analysis of randomised evidence.* BMJ : British Medical Journal, 2012. **344**: p. e2088.
18. International Weight Management in Pregnancy (IWiP) collaboration, *Effect of diet and physical activity based interventions in pregnancy on gestational weight gain and pregnancy outcomes: meta-analysis of individual participant data from randomised trials.* BMJ, 2017. **358**: p. j3119.
19. Lau, Y., et al., *Electronic-based lifestyle interventions in overweight or obese perinatal women: a systematic review and meta-analysis.* Obes Rev, 2017. **18**(9): p. 1071-1087.
20. Magro-Malosso, E.R., et al., *Exercise during pregnancy and risk of gestational hypertensive disorders: a systematic review and meta-analysis.* Acta Obstetricia et Gynecologica Scandinavica, 2017. **96**(8): p. 921-931.
21. Magro-Malosso, E.R., et al., *Exercise during pregnancy and risk of preterm birth in overweight and obese women: a systematic review and meta-analysis of randomized controlled trials.* Acta Obstet Gynecol Scand, 2017. **96**(3): p. 263-273.
22. Shepherd, E., et al., *Combined diet and exercise interventions for preventing gestational diabetes mellitus.* Cochrane Database Syst Rev, 2017. **11**: p. Cd010443.
23. Sherifali, D., et al., *The Effectiveness of eHealth Technologies on Weight Management in Pregnant and Postpartum Women: Systematic Review and Meta-Analysis.* J Med Internet Res, 2017. **19**(10): p. e337.

24. Song, C., et al., *Lifestyle intervention can reduce the risk of gestational diabetes: a meta-analysis of randomized controlled trials.* Obes Rev, 2016. **17**(10): p. 960-9.
25. Yeo, S., et al., *What characteristics of nutrition and physical activity interventions are key to effectively reducing weight gain in obese or overweight pregnant women? A systematic review and meta-analysis.* Obes Rev, 2017. **18**(4): p. 385-399.
26. Craemer, K.A., et al., *Nutrition and Exercise Strategies to Prevent Excessive Pregnancy Weight Gain: A Meta-analysis.* AJP reports, 2019. **9**(1): p. e92-e120.
27. Davenport, M.H., et al., *Prenatal exercise is not associated with fetal mortality: a systematic review and meta-analysis.* Br J Sports Med, 2019. **53**(2): p. 108-115.
28. Davenport, M.H., et al., *Impact of prenatal exercise on both prenatal and postnatal anxiety and depressive symptoms: a systematic review and meta-analysis.* British Journal of Sports Medicine, 2018. **52**(21): p. 1376.
29. Davenport, M.H., et al., *Prenatal exercise for the prevention of gestational diabetes mellitus and hypertensive disorders of pregnancy: a systematic review and meta-analysis.* Br J Sports Med, 2018. **52**(21): p. 1367-1375.
30. Davenport, M.H., et al., *Impact of prenatal exercise on maternal harms, labour and delivery outcomes: a systematic review and meta-analysis.* Br J Sports Med, 2019. **53**(2): p. 99-107.
31. Davenport, M.H., et al., *Glucose responses to acute and chronic exercise during pregnancy: a systematic review and meta-analysis.* Br J Sports Med, 2018. **52**(21): p. 1357-1366.
32. Michel, S., et al., *Do lifestyle interventions during pregnancy have the potential to reduce long-term postpartum weight retention? A systematic review and meta-analysis.* Obesity Reviews, 2019. **20**(4): p. 527-542.
33. Morison, P.N., et al., *Combined dietary-exercise intervention for gestational weight gain and birthweight: a meta-analysis.* Asia Pac J Clin Nutr, 2018. **27**(4): p. 860-868.
34. Ruchat, S.-M., et al., *Effectiveness of exercise interventions in the prevention of excessive gestational weight gain and postpartum weight retention: a systematic review and meta-analysis.* British Journal of Sports Medicine, 2018. **52**(21): p. 1347.
35. Syngelaki, A., et al., *Diet and exercise for preeclampsia prevention in overweight and obese pregnant women: systematic review and meta-analysis.* J Matern Fetal Neonatal Med, 2019. **32**(20): p. 3495-3501.
36. Walker, R., et al., *Attenuating Pregnancy Weight Gain-What Works and Why: A Systematic Review and Meta-Analysis.* Nutrients, 2018. **10**(7): p. 944.
37. Vincze, L., et al., *Interventions including a nutrition component aimed at managing gestational weight gain or postpartum weight retention: a systematic review and meta-analysis.* JBI Evidence Synthesis, 2019. **17**(3): p. 297-364.
38. Shieh, C., et al., *Intervention strategies for preventing excessive gestational weight gain: systematic review and meta-analysis.* Obesity Reviews, 2018. **19**(8): p. 1093-1109.
39. Bennett, C.J., et al., *Interventions designed to reduce excessive gestational weight gain can reduce the incidence of gestational diabetes mellitus: A systematic review and meta-analysis of randomised controlled trials.* Diabetes research and clinical practice, 2018. **141**: p. 69-79.
40. Gresham, E., et al., *Effects of dietary interventions on pregnancy outcomes: a systematic review and meta-analysis.* Matern Child Nutr, 2016. **12**(1): p. 5-23.
41. Oostdam, N., et al., *Interventions for preventing gestational diabetes mellitus: a systematic review and meta-analysis.* J Womens Health (Larchmt), 2011. **20**(10): p. 1551-63.
42. Quinlivan, J.A., S. Julania, and L. Lam, *Antenatal dietary interventions in obese pregnant women to restrict gestational weight gain to Institute of Medicine recommendations: a meta-analysis.* Obstet Gynecol, 2011. **118**(6): p. 1395-401.
43. Tieu, J., et al., *Dietary advice interventions in pregnancy for preventing gestational diabetes mellitus.* Cochrane Database Syst Rev, 2017. **1**: p. Cd006674.
44. Zhang, R., et al., *Effects of low-glycemic-index diets in pregnancy on maternal and newborn outcomes in pregnant women: a meta-analysis of randomized controlled trials.* Eur J Nutr, 2018. **57**(1): p. 167-177.
45. Gresham, E., et al., *Effects of dietary interventions on neonatal and infant outcomes: a systematic review and meta-analysis.* Am J Clin Nutr, 2014. **100**(5): p. 1298-321.
46. Elliott-Sale, K.J., C.T. Barnett, and C. Sale, *Exercise interventions for weight management during pregnancy and up to 1 year postpartum among normal weight, overweight and obese women: a systematic review and meta-analysis.* Br J Sports Med, 2015. **49**(20): p. 1336-42.

47. Han, S., P. Middleton, and C.A. Crowther, *Exercise for pregnant women for preventing gestational diabetes mellitus.* Cochrane Database Syst Rev, 2012(7): p. Cd009021.
48. Lamina, S. and E. Agbanusi, *Effect of aerobic exercise training on maternal weight gain in pregnancy: a meta-analysis of randomized controlled trials.* Ethiopian journal of health sciences, 2013. **23**(1): p. 59-64.
49. Russo, L.M., et al., *Physical activity interventions in pregnancy and risk of gestational diabetes mellitus: a systematic review and meta-analysis.* Obstet Gynecol, 2015. **125**(3): p. 576-82.
50. Sanabria-Martínez, G., et al., *Effectiveness of physical activity interventions on preventing gestational diabetes mellitus and excessive maternal weight gain: a meta-analysis.* Bjog, 2015. **122**(9): p. 1167-74.
51. Streuling, I., et al., *Physical activity and gestational weight gain: a meta-analysis of intervention trials.* Bjog, 2011. **118**(3): p. 278-84.
52. Sui, Z., R.M. Grivell, and J.M. Dodd, *Antenatal exercise to improve outcomes in overweight or obese women: A systematic review.* Acta Obstet Gynecol Scand, 2012. **91**(5): p. 538-45.
53. da Silva, S.G., et al., *Leisure-Time Physical Activity in Pregnancy and Maternal-Child Health: A Systematic Review and Meta-Analysis of Randomized Controlled Trials and Cohort Studies.* Sports Med, 2017. **47**(2): p. 295-317.
54. Di Mascio, D., et al., *Exercise during pregnancy in normal-weight women and risk of preterm birth: a systematic review and meta-analysis of randomized controlled trials.* Am J Obstet Gynecol, 2016. **215**(5): p. 561-571.
55. Sanabria-Martínez, G., et al., *Effects of Exercise-Based Interventions on Neonatal Outcomes: A Meta-Analysis of Randomized Controlled Trials.* Am J Health Promot, 2016. **30**(4): p. 214-23.
56. Zheng, J., H. Wang, and M. Ren, *Influence of exercise intervention on gestational diabetes mellitus: a systematic review and meta-analysis.* J Endocrinol Invest, 2017. **40**(10): p. 1027-1033.
57. Chatzakis, C., et al., *Prevention of gestational diabetes mellitus in overweight or obese pregnant women: A network meta-analysis.* Diabetes Res Clin Pract, 2019. **158**: p. 107924.
58. Du, M.C., et al., *Effects of physical exercise during pregnancy on maternal and infant outcomes in overweight and obese pregnant women: A meta-analysis.* Birth, 2019. **46**(2): p. 211-221.
59. Guillemette, L., et al., *Exercise in Pregnancy and Children's Cardiometabolic Risk Factors: a Systematic Review and Meta-Analysis.* Sports medicine - open, 2018. **4**(1): p. 35-35.
60. Ming, W.-K., et al., *The effect of exercise during pregnancy on gestational diabetes mellitus in normal-weight women: a systematic review and meta-analysis.* BMC Pregnancy and Childbirth, 2018. **18**(1): p. 440.
61. Nasiri-Amiri, F., et al., *The effect of exercise on the prevention of gestational diabetes in obese and overweight pregnant women: a systematic review and meta-analysis.* Diabetology & Metabolic Syndrome, 2019. **11**(1): p. 72.
62. Wang, J., et al., *Impact of exercise on maternal gestational weight gain: An updated meta-analysis of randomized controlled trials.* Medicine, 2019. **98**(27): p. e16199-e16199.
63. Poyatos-León, R., et al., *Effects of exercise during pregnancy on mode of delivery: a meta-analysis.* Acta Obstet Gynecol Scand, 2015. **94**(10): p. 1039-47.
64. Wiebe, H.W., et al., *The Effect of Supervised Prenatal Exercise on Fetal Growth: A Meta-analysis.* Obstetrics & Gynecology, 2015. **125**(5): p. 1185-1194.
65. Yin, Y.N., et al., *Physical activity during pregnancy and the risk of gestational diabetes mellitus: a systematic review and meta-analysis of randomised controlled trials.* Br J Sports Med, 2014. **48**(4): p. 290-5.

S7 Tables: Overlap of included studies in the systematic reviews

S7a: Included studies in smoking reviews

Review	References of included behavioural interventions delivered in pregnancy
Chamberlain *et al.* 2017 [1]	1. Albrecht S, Cassidy B, Salamie D, Reynolds M. What's happening A smoking cessation intervention for pregnant adolescents: implications for nurse practitioners. Journal of American Academy of Nurse Practitioners 199911(4):155–9<br>2. Albrecht S, Stone CA, Payne L, Reynolds MD. A preliminary study of the use of peer support in smoking cessation programs for pregnant adolescents. Journal of the American Academy of Nurse Practitioners 199810:119–25<br>3. Albrecht SA, Caruthers D, Patrick T, Reynolds M, Salamie D, Higgins LW, et al. A randomised controlled trial of a smoking cessation intervention for pregnant adolescents. Nursing Research 200655(6):402–10<br>4. Baric L, MacArthur C, Sherwood M. A study of health education aspects of smoking in pregnancy. International Journal of Health Education 197619(2 Suppl):1–17<br>5. Bauman 1983 Bauman KE, Koch GG, Dent CW, Bryan ES. The influence of observing carbon monoxide level on cigarette smoking by public prenatal patients. American Journal of Public Health 198373:1089–91<br>6. Belizan JM, Villar J, Victora C, Farnot U, Langer A, Barros F. Impact of health education during pregnancy on behavior and utilization of health resources. American Journal of Obstetrics and Gynecology 1995173:894–9<br>7. Bullock L, Everett KD, Mullen PD, Geden E, Longo DR, Madsen R. Baby BEEP: A randomized controlled trial of nurses' individualized social support for poor rural pregnant smokers. Maternal and Child Health Journal 200913(3): 395–406<br>8. Bullock LF, Everett KD, Mullen PD. Baby beep: a randomized clinical trial of smoking cessation for lowincome rural pregnant women using nurse-delivered social support. Annals of Behavioral Medicine 200835:S99<br>9. Bullock LF, Hornblow AR, Duff GB, Wells JE. Telephone support for pregnant women: outcome in late pregnancy. New Zealand Medical Journal 1995108:476–8<br>10. Burling TA, Bigelow GE, Robinson JC, Mead AM. Smoking during pregnancy: reduction via objective assessment and directive advice. Behavior Therapy 199122:31–40<br>11. Byrd JC, Meade CD. Smoking cessation among pregnant women in an urban setting. Wisconsin Medical Journal 199392:609–12<br>12. Campbell E, Walsh RA, Sanson-Fisher R, Burrows S, Stojanovski E. A group randomised trial of two methods for disseminating a smoking cessation programme to public antenatal clinics: effects on patient outcomes. Tobacco Control 200615(2):97–102<br>13. Cinciripini 2000 Blalock JA, Fouladi RT, Wetter DW, Cinciripini PM. Depression in pregnant women seeking smoking cessation treatment. Addictive Behaviours 200530(6):1195–208<br>14. Cinciripini PM, Blalock JA, Minnix JA, Robinson JD, Brown VL, Lam C, et al. Effects of an intensive depression focused intervention for smoking cessation in pregnancy. Journal of Consulting & Clinical Psychology 201078(1): 44–54<br>15. Cook C, Ward S, Myers S, Spinnato J. A prospective, randomized evaluation of intensified therapy for smoking reduction in pregnancy. American Journal of Obstetrics and Gynecology 1995172:290<br>16. Cope GF, Nayyar P, Holder R. Feedback from a point of-care test for nicotine intake to reduce smoking during pregnancy. Annals of Clinical Biochemistry 2003.40(Pt 6): 674–9<br>17. Donatelle RJ, Prows SL, Champeau D, Hudson D. Randomised controlled trial using social support and financial incentives for high risk pregnant smokers: Significant Other Supporter (SOS) program. Tobacco Control 2000.9 Suppl 3:iii67–iii69<br>18. Donovan J. Randomised controlled trial of anti-smoking advice in pregnancy. British Journal of Preventive and Social Medicine 1977.31(1):6–12<br>19. Dornelas EA, Magnavita J, Beazoglou T, Fischer EH, Oncken C, Lando H, et al. Efficacy and cost-effectiveness of a clinic-based counseling intervention tested in an ethnically diverse sample of pregnant smokers. Patient Education and Counseling 2006.64(1-3):342–9<br>20. Dunkley J. Training midwives to help pregnant women stop smoking Nursing Times 1997.93(5):64–6<br>21. Eades SJ, Sanson-Fisher RW, Wenitong M, Panaretto K, D'Este C, Gilligan C, et al. An intensive smoking intervention for pregnant Aboriginal and Torres Strait Islander women: a randomised controlled trial. Medical Journal of Australia 2012.197(1):42–6<br>22. El-Mohandes AA, El-Khorazaty MN, Kiely M, Gantz MG. Smoking cessation and relapse among pregnant African-American smokers in Washington, DC. Maternal & Child Health Journal 2011.15 Suppl 1:S96–S105<br>23. Ershoff DH, Quinn VP, Boyd NR, Stern J, Gregory M, Wirtschafter D. The Kaiser Permanente prenatal smoking cessation trial. When more isn't better, what is enough? American Journal of Preventive Medicine 1999.17(3):161–8<br>24. Ershoff DH, Quinn VP, Mullen PD. A randomized trial of a serialized self-help smoking cessation program for pregnant women in an HMO. American Journal of Public Health 1989.79(2):182–7<br>25. Gielen AC, Windsor R, Faden RR, O'Campo P, Repke J, Davis M. Evaluation of a smoking cessation intervention for pregnant women in an urban prenatal clinic. Health Education Research 1997.12(2):247–54<br>26. Glover M, Kira A, Walker N, Bauld L. Using incentives to encourage smoking abstinence among pregnant indigenous women? A feasibility study. Maternal and Child Health Journal 2014.19(6):1393–9<br>27. Graham AV, Reeb KG, Kitson GC, Zyzanski SJ, Frank SH. A clinical trial to reduce the rate of low birth weight in an inner-city black population. Family Medicine 1992.24: 439–46<br>28. Haddow JE, Wald NJ, Palomaki GE, Kloza EM, Knight GJ. Cotinine-assisted intervention in pregnancy to reduce smoking and low birthweight delivery. British Journal of Obstetrics and Gynaecology 1991.98(9):859–65<br>29. Hajek P, West R, Lee A, Foulds J, Owen L, Eiser JR, et al. Randomized controlled trial of a midwife-delivered brief smoking cessation intervention in pregnancy. Addiction 2001.96(3):485–94<br>30. Harris M, Reynolds B. A pilot study of home-based smoking cessation programs for rural, Appalachian, pregnant smokers. Journal of Obstetric, Gynecologic, and Neonatal Nursing 2015 [EPub ahead of print]<br>31. Hartmann KE, Koch MA, Pahel-Short L, Thorp JM. A randomized controlled trial of smoking cessation intervention in pregnancy in an academic clinic. Obstetrics & Gynecology 1996.87:621–6

Review	References of included behavioural interventions delivered in pregnancy
	32. Haug K, Fugelli P, Aaro LE, Foss OP. Is smoking intervention in general practice more successful among pregnant than non-pregnant women? Family Practice 1994 11:111–6
	33. Haug NA, DiClemente C, Svikis DS. Motivational enhancement therapy for nicotine dependence in methadone-maintained pregnant women. Psychology of Addictive Behaviours 2004.18(3):289–92
	34. Hegaard H, Hjaergaard H, Moller L, Wachmann H, Ottesen B. Multimodel intervention raises smoking cessation rate during pregnancy. Acta Obstetricia et Gynecologica Scandinavica 2003.82:813–9
	35. Heil SH, Higgins ST, Bernstein IM, Solomon LJ, Rogers RE, Thomas CS, et al. Effects of voucher-based incentives on abstinence from cigarette smoking and fetal growth among pregnant women. Addiction. 2008.103(6):1009–18
	36. Hennrikus D, Pirie P, Hellerstedt W, Lando HA, Steele J, Dunn C. Increasing support for smoking cessation during pregnancy and postpartum: results of a randomized controlled pilot study. Preventive Medicine 2010.50(3): 134–7
	37. Herbec A, Brown J, Tombor I, Michie S, West R. Pilot randomized controlled trial of an internet-based smoking cessation intervention for pregnant smokers ('MumsQuit'). Drug & Alcohol Dependence 2014.140:130–6
	38. Hiett A, Brazus S, Hedberg J, Brown H. Smoking cessation program effectiveness during pregnancy. American Journal of Obstetrics and Gynecology 2000.182(1 Pt 2):S150
	39. Higgins ST, Washio Y, Lopez AA, Heil SH, Solomon LJ, Lynch ME, et al. Examining two different schedules of financial incentives for smoking cessation among pregnant women. Preventive Medicine 2014 Vol 68:51–7
	40. Hjalmarson AIM, Svanberg B, Hahn L. Stopping smoking in pregnancy: effect of a self-help manual in a controlled trial. British Journal of Obstetrics and Gynaecology 1991.98: 260–4
	41. Hughes E, Lamont D, Beecroft M, Wilson D. Randomized trial of a "stage-of-change" orientated smoking cessation intervention in infertile and pregnant women. Fertility and Sterility 2000.74(3):498–503
	42. Kemp L, Harris E, McMahon C, Matthey S, Vimpani G, Anderson T, Schmied V, Aslam H, Zapart S. Child and family outcomes of a long-term nurse home visitation programme: a randomised controlled trial. Archives of Disease in Childhood 2011.96(6):533–540
	43. Kendrick JS, Metzger RW, Sexton M, Spierto FW, Floyd RL, Gargiullo PM, et al. Integrating smoking cessation into routine public prenatal care: the Smoking Cessation in Pregnancy project. American Journal of Public Health 1995 85:217–22
	44. Lawrence T, Aveyard P, Croghan E. What happens to women's self-reported cigarette consumption and urinary cotinine levels in pregnancy? Addiction 2003.98:1315–20
	45. Lee M, Miller SM, Wen KY, Hui SA, Roussi P, Hernandez E. Cognitive-behavioral intervention to promote smoking cessation for pregnant and postpartum inner city women. Journal of Behavioral Medicine 2015.38(6):923–43
	46. LeFevre ML, Ewigman B, Evans JK. Is smoking an indication for prenatal ultrasonography? RADIUS Study Group. Archives of Family Medicine 1995.4:120–3
	47. Lilley J, Forster DP. A randomised controlled trial of individual counselling of smokers in pregnancy. Public Health 1986.100:309–15
	48. Lillington L, Chlebowski R, Ruvalcaba M, Novak D, Royce J. Evaluation of a smoking cessation program for pregnant minority smokers. Cancer Practice 1995.3(3):157–63
	49. Loeb BK, Waage G, Bailey J. Smoking intervention in pregnancy. Proceedings of the Fifth World Conference on Smoking and Health 1983 July Winnipeg, Canada. 1983: 389–95
	50. Loukopoulou A, Vardavas C, Tzatzarakis M, Farmakides G, Rossolymos C, Chrelias C, et al. Urinary nicotine and cotinine as a method for assessing the effectiveness of an intervention to aid smoking cessation during pregnancy. Chest 2012.142(4 Suppl 1):1081A
	51. Lowe JB, Balanda KP, Stanton WR, Del Mar C, O'Connor V. Dissemination of an efficacious antenatal smoking cessation program in public hospitals in Australia: a randomised controlled trial. Health Education & Behavior 2002.29(5):608–19
	52. Lowe JB, Windsor R, Balanda K, Woodby L. Smoking relapse prevention methods for pregnant women: a formative evaluation. American Journal of Health Promotion 1997.11:244–6
	53. Malchodi CS, Oncken C, Dornelas EA, Caramanica L, Gregonis E. The effects of peer counselling on smoking cessation and reduction. Obstetrics & Gynecology 2003.101 (3):504–10
	54. Manfredi C, Crittenden KS, Warnecke R, Engler J, Cho YI, Shaligram C. Evaluation of a motivational smoking cessation intervention for women in public health clinics. Preventive Medicine 1999.28:51–60
	55. Mauriello L, Dyment S, Prochaska J, Gagliardi A, Weingrad-Smith J. Acceptability and feasibility of a multiple-behaviour, computer-tailored intervention for underserved pregnant women. Journal of Midwifery and Women's Health 2011.56:75–80
	56. Mayer JP, Todd R, Hawkins B. A randomised evaluation of smoking cessation interventions for pregnant women at a WIC clinic. American Journal of Public Health 1990.80: 76–7
	57. McBride CM, Baucom DH, Peterson BL, Pollak KI, Palmer C, Westman E, et al. Prenatal and postpartum smoking abstinence: a partner assisted approach. American Journal of Preventive Medicine 2004.27(3):232–8
	58. McBride CM, Curry SJ, Lando HA, Pirie PL, Grothaus LC, Nelson JC. Prevention of relapse in women who quit smoking during pregnancy. American Journal of Public Health 1999.89:706–11
	59. McLeod D, Pullon S, Benn C, Cookson T, Dowell A, Viccars A, et al. Can support and education for smoking cessation and reduction be provided effectively by midwives within primary maternity care? Midwifery 2004.20:37–50
	60. Mejdoubi J, van den Heijkant SCCM, van Leerdam FJM, Crone M, Crijnen A, HiraSing RA. Effects of nurse home visitation on cigarette smoking pregnancy outcomes: a randomized controlled trial. Midwifery 2014.30:688–95
	61. Messimer SR, Henry RC, Hickner JM. A comparison of two antismoking interventions among pregnant women in eleven primary care practices. Journal of Family Practice 1989.28(3):283–8
	62. Moore LO, Campbell R, Whelan A, Mills N, Lupton P, Misselbrook E, et al. Self help smoking cessation in pregnancy: cluster randomised controlled trial. BMJ 2002 325:1383–6
	63. Moore ML, Meis PJ, Ernest JM, Wells HB, Zaccaro DJ, Terrell T. A randomized trial of nurse intervention to reduce preterm and low birth weight births. Obstetrics & Gynecology 1998.91:656–61
	64. Naughton F, Prevost AT, Gilbert H, Sutton S. Randomized controlled trial evaluation of a tailored leaflet and SMS text message self-help intervention for pregnant smokers (MiQuit). Nicotine and Tobacco Research 2012.14(5): 569–77

Review	References of included behavioural interventions delivered in pregnancy
	65. Olds D, Robinson J, O'Brien R, Luckey D, Pettit L, Henderson C, et al. Home visiting by paraprofessionals and by nurses: a randomized, controlled trial. Pediatrics 2002 110(3):486–96
	66. Olds DL, Henderson CR, Chamberlin R, Tatelbaum R. Preventing child abuse and neglect: a randomized trial of nurse home visitation. Pediatrics 1986.78:65–78
	67. Ondersma SJ, Svikis DS, Lam PK, Connors-Burge VS, Ledgerwood DM, Hopper JA. A randomized trial of computer-delivered brief intervention and low-intensity contingency management for smoking during pregnancy. Nicotine & Tobacco Research 2012 Vol 14, issue 3: 351–60
	68. Oude Wesselink SF, Lingsma HF, Reulings PG, Wentzel HR, Erasmus V, Robben PB, et al. Does government supervision improve stop-smoking counseling in midwifery practices? Nicotine & Tobacco Research 2015 Vol 17, issue 5:572–9
	69. Panjari M, Bell R, Bishop S, Astbury J, Rice G, Doery J. A randomized controlled trial of a smoking cessation intervention during pregnancy. Australian and New Zealand Journal of Obstetrics and Gynaecology. 1999.39(3):312–7
	70. Parker DR, Windsor RA, Roberts MB, Hecht J, Hardy NV, Strolla LO, et al. Feasibility, cost, and costeffectiveness of a telephone-based motivational intervention for underserved pregnant smokers. Nicotine and Tobacco Research 2007.9(10):1043–51
	71. Patten CA, Windsor RA, Renner CC, Enoch C, Hochreiter A, Nevak C, et al. Feasibility of a tobacco cessation intervention for pregnant Alaska Native women. Nicotine & Tobacco Research 2009.12(2):79–87
	72. Pbert L, Ockene JK, Zapka J, Ma Y, Goins KV, Oncken C, et al. A community health center smoking cessation intervention for pregnant and postpartum women. American Journal of Preventive Medicine 2004.26(5): 377–85
	73. Peterson L, Rosen A, Podedworny T, Kotch J, Handel J. Smoking reduction during pregnancy by a program of selfhelp and clinical support. Obstetrics & Gynecology 1992.79: 924–30
	74. Polanska K, Hanke W, Sobala W, Lowe JB. Efficacy and effectiveness of the smoking cessation program for pregnant women. International Journal of Occupational Medicine and Environmental Health 2004.17(3):369–77
	75. Pollak KI, Lyna P, Bilheimer A, Farrell D, Gao X, Swamy GK, et al. A pilot study testing SMS text delivered scheduled gradual reduction to pregnant smokers. Nicotine & Tobacco Research 2013.15(10):1773–6
	76. Prapavessis H, De Jesus S, Harper T, Cramp A, Fitzgeorge L, Mottola MF, et al. The effects of acute exercise on tobacco cravings and withdrawal symptoms in temporary abstinent pregnant smokers. Addictive Behaviors 2014.39(3):703–8
	77. Price JH, Snyder FF, Roberts SM, Losh DP, Desmond SM, Krol RA. Comparison of three antismoking interventions among pregnant women in an urban setting: a randomized trial. Psychological Reports 1991.68:595–604
	78. Reading AE, Cox DN. The effects of ultrasound on maternal anxiety. Journal of Behavioral Medicine 1982.5(2): 237–47
	79. Rigotti N, Park ER, Regan S, Chang Y, Perry K, Loudin B, et al. Efficacy of telephone counseling for pregnant smokers. Obstetrics & Gynecology 2006.108(1):83–92
	80. Robling M, Bekkers MJ, Bell K, Butler CC, CanningsJohn R, Channon S, et al. Effectiveness of a nurse-led intensive home-visitation programme for first-time teenage mothers (Building Blocks): a pragmatic randomised controlled trial. Lancet 2016.387(10014):146–55
	81. Secker-Walker RH, Mead PB, Goodwin GD, Lepage SS, Skelly JM, Flynn BS, et al. Individualised smoking cessation counseling during prenatal and early postnatal care. American Journal of Obstetrics and Gynecology 1994 71:1347–55
	82. Secker-Walker RH, Solomon LJ, Flynn BS, Skelly JM, Mead PB. Reducing smoking during pregnancy and postpartum: physician's advice supported by individual counselling. Preventive Medicine 1998.27:422–30
	83. Secker-Walker RH, Solomon LJ, Geller BM, Flynn BS, Worden JK, Skelly JM, et al. Modeling smoking cessation: exploring the use of a videotape to help pregnant women quit smoking. Women & Health 1997.25:23–35
	84. Sexton M, Hebel JR. A clinical trial of change in maternal smoking and its effect on birth weight. JAMA 1984.251: 911–5
	85. Solomon LJ, Secker-Walker RH, Flynn BS, Skelly JM, Capeless EL. Proactive telephone peer support to help pregnant women stop smoking. Tobacco Control 2000.9 Suppl 3:iii72–iii74
	86. Stotts AL, DeLaune KA, Schmitz JM, Grabowski J. Impact of a motivational intervention on mechanisms of change in low-income pregnant women. Addictive Behaviors 2004.29 (8):1649–57
	87. Stotts AL, Groff JY, Velasquez MM, Benjamin-Garner R, Green C, Carbonari JP, et al. Ultrasound feedback and motivational interviewing targeting smoking cessation in the second and third trimesters of pregnancy. Nicotine & Tobacco Research 2009.11(8):961–8
	88. Stotts S, DiClemente CC, Dolan-Mullen P. One-to-One: a motivational intervention for resistant pregnant smokers. Addictive Behaviors 2002.27:275–92
	89. Strecher VJ, Bishop KR, Bernhardt J, Thorp JM, Cheuvrout B, Potts P. Quits for keeps: tailored smoking cessation guides for pregnancy and beyond. Tobacco Control 2000.9 (Suppl 3):iii78–iii79
	90. Tappin D, Bauld L, Purves D, Boyd K, Sinclair L, MacAskill S, et al. Financial incentives for smoking cessation in pregnancy: randomised controlled trial. BMJ (Clinical Research Ed.) 2015.350:h134
	91. Tappin DM, Lumsden MA, Gilmour WH, Crawford F, McIntyre D, Stone DH, et al. Randomised controlled trial of home based motivational interviewing by midwives to help pregnant smokers quit or cut down. BMJ 2005.331: 373–7
	92. Tappin DM, Lumsden MA, McIntyre D, McKay C, Gilmour WH, Webber R, et al. A pilot study to establish a randomized trial methodology to test the efficacy of a behavioural intervention. Health Education Research 2000 15(4):491–502
	93. Thornton L. Smoking and Pregnancy: Feasibility and Effectiveness of a Smoking Intervention Programme Among Pregnant Women [thesis]. Dublin: Dept of Public Health, 1997
	94. Tsoh JY, Kohn MA, Gerbert B. Promoting smoking cessation in pregnancy with Video Doctor plus provider cueing: a randomized trial. Acta Obstetricia et Gynecologica Scandinavica 2010.89(4):515–23

Review	References of included behavioural interventions delivered in pregnancy
	95. Tuten M, Fitzsimons H, Chisolm MS, Nuzzo PA, Jones HE. Contingent incentives reduce cigarette smoking among pregnant, methadone-maintained women: results of an initial feasibility and efficacy randomized clinical trial. Addiction 2012.107(10):1868–77<br>96. Ussher M, Lewis S, Aveyard P, Manyonda I, West R, Lewis B, et al. Physical activity for smoking cessation in pregnancy: randomised controlled trial. BMJ (Clinical Research Ed.) 2015.350:h2145<br>97. Valbo A, Eide T. Smoking cessation in pregnancy: the effect of hypnosis in a randomized study. Addictive Behaviors 1996.21:29–35<br>98. Valbo A, Nylander G. Smoking cessation in pregnancy: intervention among heavy smokers. Acta Obstetricia et Gynecologica Scandinavica 1994.73:215–9<br>99. Vilches P. Consumption of Tobacco in Pregnant Women: Proposal of a Psychological Intervention Model in the Public Health System of Andalucia [thesis] Malaga: Univesidad di Malaga, 2009<br>100. Walsh RA, Melmeth A, Byrne JM, Brinsmead MW, Redman S. A smoking cessation program at a public antenatal clinic. American Journal of Public Health 1997.87: 1201–4<br>101. Wilkinson SA, McIntyre HD. Evaluation of the 'healthy start to pregnancy' early antenatal health promotion workshop: a randomized controlled trial. BMC Pregnancy and Childbirth 2012.0.590972222222222<br>102. Windsor R, Woodby L, Miller T, Hardin M. Effectiveness of Smoking Cessation and Reduction in Pregnancy Treatment (SCRIPT) methods in Medicaid-supported prenatal care: Trial III. Health Education & Behavior 2011.38(4):412–22<br>103. Windsor RA, Lowe JB, Perkins LL, Smith-Yoder D, Artz L, Crawford M, et al. Health education for pregnant smokers: its behavioral impact and cost benefit. American Journal of Public Health 1993.83:201–6<br>104. Windsor RA, Spanos D, Samuelsson C, Bartlett EE, Manzella B, Reese Y, et al. The effectiveness of smoking cessation methods for smokers in public health maternity clinics: a randomized trial. American Journal of Public Health 1985.75:1389–92
Veisani *et al.* 2017 [2]	1. Tappin D, Bauld L, Purves D, et al. Financial incentives for smoking cessation in pregnancy: randomised controlled trial. BMJ. 2015;350:h134.<br>2. Berlin I, Grang_e G, Jacob N, et al. Nicotine patches in pregnant smokers: randomised, placebo controlled, multicentre trial of efficacy. BMJ. 2014;348:g1622.<br>3. Tappin DM, Lumsden MA, Gilmour WH, et al. Randomised controlled trial of home based motivational interviewing by midwives to help pregnant smokers quit or cut down. BMJ. 2005;331(7513):373–377.<br>4. MacArthur C, Knox EG, Lancashire RJ. Effects at age nine of maternal smoking in pregnancy: experimental and observational findings. BJOG. 01;108(1):67–73.<br>5. Oncken C, Dornelas E, Greene J, et al. Nicotine gum for pregnant smokers: a randomized controlled trial. Obstet Gynecol. 2008;112(4):859–867.<br>6. Higgins ST, Bernstein IM, Washio Y, et al. Effects of smoking cessation with voucher-based contingency management on birth outcomes. Addiction. 2010; 105(11):2023–2030.<br>7. Higgins ST, Washio Y, Lopez AA, et al. Examining two different schedules of financial incentives for smoking cessation among pregnant women. Prev Med.2014;68:51–57.<br>8. Ram A, Tuten M, Chisolm MS. Cigarette smoking reduction in pregnant women with opioid use disorder. J Addict Med. 2016;10(1):53–59.<br>9. Benjamin-Garner R, Stotts A. Impact of smoking exposure change on infant birth weight among a cohort of women in a Prenatal Smoking Cessation Study. Nicotine Tob Res. 2013;15(3):685–692.<br>10. Ussher M, Lewis S, Aveyard P, et al. The London Exercise and Pregnant smokers (LEAP) trial: a randomised controlled trial of physical activity for smoking cessation in pregnancy with an economic evaluation. Health Technol Assess. 2015;19(84):vii–xxiv, 1<br>11. Pola_nska K, Hanke W, Sobala W, et al. Efficacy and effectiveness of the smoking cessation program for pregnant women. Int J Occup Med Environ Health. 2004;17(3):369–377.<br>12. England LJ, Kendrick JS, Wilson HG, et al. Effects of smoking reduction during pregnancy on the birth weight of term infants. Am J Epidemiol. 2001;154(8): 694–701.<br>13. Wisborg K, Henriksen TB, Jespersen LB, et al. Nicotine patches for pregnant smokers: a randomized controlled study. Obstet Gynecol. 2000;96(6):967–971.<br>14. Mejdoubi J, van den Heijkant SCCM, van Leerdam FJM, et al. Effects of nurse home visitation on cigarette smoking, pregnancy outcomes and breastfeeding: a randomized controlled trial. Midwifery. 2014;30(6): 688–695.<br>15. Panjari M, Bell R, Bishop S, et al. A Randomized Controlled Trial of a Smoking Cessation intervention during pregnancy. Aust NZ J Obstet Gynaecol.1999;39(3):312–317.<br>16. Ruger JP, Weinstein MC, Hammond SK, et al. Costeffectiveness of motivational interviewing for Smoking Cessation and relapse prevention among low-ncome pregnant women: a randomized controlled trial. Value Health J Int Soc Pharmacoecon Outcomes Res. 2008;11(2):191–198.

S7b: Unique publications included in smoking reviews

Smoking unique publications	
1.	Abroms LC, Johnson PR, Heminger CL, et al. Quit4baby: results from a pilot test of a mobile smoking cessation program for pregnant women. JMIR Mhealth Uhealth. 2015;3(1):e10
2.	Albrecht S, Cassidy B, Salamie D, Reynolds M. What's happening A smoking cessation intervention for pregnant adolescents: implications for nurse practitioners. Journal of American Academy of Nurse Practitioners 199911(4):155–9
3.	Albrecht S, Stone CA, Payne L, Reynolds MD. A preliminary study of the use of peer support in smoking cessation programs for pregnant adolescents. Journal of the American Academy of Nurse Practitioners 199810:119–25
4.	Albrecht SA, Caruthers D, Patrick T, Reynolds M, Salamie D, Higgins LW, et al. A randomised controlled trial of a smoking cessation intervention for pregnant adolescents. Nursing Research 200655(6):402–10
5.	Baric L, MacArthur C, Sherwood M. A study of health education aspects of smoking in pregnancy. International Journal of Health Education 197619(2 Suppl):1–17
6.	Bauman 1983 Bauman KE, Koch GG, Dent CW, Bryan ES. The influence of observing carbon monoxide level on cigarette smoking by public prenatal patients. American Journal of Public Health 198373:1089–91
7.	Belizan JM, Villar J, Victora C, Farnot U, Langer A, Barros F. Impact of health education during pregnancy on behavior and utilization of health resources. American Journal of Obstetrics and Gynecology 1995173:894–9

Smoking unique publications	
8.	Benjamin-Garner R, Stotts A. Impact of smoking exposure change on infant birth weight among a cohort of women in a Prenatal Smoking Cessation Study. Nicotine Tob Res. 2013;15(3):685–692.
9.	Berlin I, Grang_e G, Jacob N, et al. Nicotine patches in pregnant smokers: randomised, placebo controlled, multicentre trial of efficacy. BMJ. 2014;348:g1622.
10.	Bullock L, Everett KD, Mullen PD, Geden E, Longo DR, Madsen R. Baby BEEP: A randomized controlled trial of nurses' individualized social support for poor rural pregnant smokers. Maternal and Child Health Journal 200913(3): 395–406
11.	Bullock LF, Everett KD, Mullen PD. Baby beep: a randomized clinical trial of smoking cessation for lowincome rural pregnant women using nurse-delivered social support. Annals of Behavioral Medicine 200835:S99
12.	Bullock LF, Hornblow AR, Duff GB, Wells JE. Telephone support for pregnant women: outcome in late pregnancy. New Zealand Medical Journal 1995108:476–8
13.	Burling TA, Bigelow GE, Robinson JC, Mead AM. Smoking during pregnancy: reduction via objective assessment and directive advice. Behavior Therapy 199122:31–40
14.	Byrd JC, Meade CD. Smoking cessation among pregnant women in an urban setting. Wisconsin Medical Journal 199392:609–12
15.	Campbell E, Walsh RA, Sanson-Fisher R, Burrows S, Stojanovski E. A group randomised trial of two methods for disseminating a smoking cessation programme to public antenatal clinics: effects on patient outcomes. Tobacco Control 200615(2):97–102
16.	Cinciripini 2000 Blalock JA, Fouladi RT, Wetter DW, Cinciripini PM. Depression in pregnant women seeking smoking cessation treatment. Addictive Behaviours 200530(6):1195–208
17.	Cinciripini PM, Blalock JA, Minnix JA, Robinson JD, Brown VL, Lam C, et al. Effects of an intensive depression focused intervention for smoking cessation in pregnancy. Journal of Consulting & Clinical Psychology 201078(1): 44–54
18.	Cook C, Ward S, Myers S, Spinnato J. A prospective, randomized evaluation of intensified therapy for smoking reduction in pregnancy. American Journal of Obstetrics and Gynecology 1995172:290
19.	Cope GF, Nayyar P, Holder R. Feedback from a point of-care test for nicotine intake to reduce smoking during pregnancy. Annals of Clinical Biochemistry 2003.40(Pt 6): 674–9
20.	Donatelle RJ, Prows SL, Champeau D, Hudson D. Randomised controlled trial using social support and financial incentives for high risk pregnant smokers: Significant Other Supporter (SOS) program. Tobacco Control 2000.9 Suppl 3:iii67–iii69
21.	Donovan J. Randomised controlled trial of anti-smoking advice in pregnancy. British Journal of Preventive and Social Medicine 1977.31(1):6–12
22.	Dornelas EA, Magnavita J, Beazoglou T, Fischer EH, Oncken C, Lando H, et al. Efficacy and cost-effectiveness of a clinic-based counseling intervention tested in an ethnically diverse sample of pregnant smokers. Patient Education and Counseling 2006.64(1-3):342–9
23.	Dunkley J. Training midwives to help pregnant women stop smoking Nursing Times 1997.93(5):64–6
24.	Eades SJ, Sanson-Fisher RW, Wenitong M, Panaretto K, D'Este C, Gilligan C, et al. An intensive smoking intervention for pregnant Aboriginal and Torres Strait Islander women: a randomised controlled trial. Medical Journal of Australia 2012.197(1):42–6
25.	El-Mohandes AA, El-Khorazaty MN, Kiely M, Gantz MG. Smoking cessation and relapse among pregnant African-American smokers in Washington, DC. Maternal & Child Health Journal 2011.15 Suppl 1:S96–S105
26.	England LJ, Kendrick JS, Wilson HG, et al. Effects of smoking reduction during pregnancy on the birth weight of term infants. Am J Epidemiol. 2001;154(8): 694–701.
27.	Ershoff DH, Quinn VP, Boyd NR, Stern J, Gregory M, Wirtschafter D. The Kaiser Permanente prenatal smoking cessation trial. When more isn't better, what is enough? American Journal of Preventive Medicine 1999.17(3):161–8
28.	Ershoff DH, Quinn VP, Mullen PD. A randomized trial of a serialized self-help smoking cessation program for pregnant women in an HMO. American Journal of Public Health 1989.79(2):182–7
29.	Gielen AC, Windsor R, Faden RR, O'Campo P, Repke J, Davis M. Evaluation of a smoking cessation intervention for pregnant women in an urban prenatal clinic. Health Education Research 1997.12(2):247–54
30.	Glover M, Kira A, Walker N, Bauld L. Using incentives to encourage smoking abstinence among pregnant indigenous women? A feasibility study. Maternal and Child Health Journal 2014.19(6):1393–9
31.	Graham AV, Reeb KG, Kitson GC, Zyzanski SJ, Frank SH. A clinical trial to reduce the rate of low birth weight in an inner-city black population. Family Medicine 1992.24: 439–46
32.	Haddow JE, Wald NJ, Palomaki GE, Kloza EM, Knight GJ. Cotinine-assisted intervention in pregnancy to reduce smoking and low birthweight delivery. British Journal of Obstetrics and Gynaecology 1991.98(9):859–65
33.	Hajek P, West R, Lee A, Foulds J, Owen L, Eiser JR, et al. Randomized controlled trial of a midwife-delivered brief smoking cessation intervention in pregnancy. Addiction 2001.96(3):485–94
34.	Harris M, Reynolds B. A pilot study of home-based smoking cessation programs for rural, Appalachian, pregnant smokers. Journal of Obstetric, Gynecologic, and Neonatal Nursing 2015 [EPub ahead of print]
35.	Hartmann KE, Koch MA, Pahel-Short L, Thorp JM. A randomized controlled trial of smoking cessation intervention in pregnancy in an academic clinic. Obstetrics & Gynecology 1996.87:621–6
36.	Haug K, Fugelli P, Aaro LE, Foss OP. Is smoking intervention in general practice more successful among pregnant than non-pregnant women? Family Practice 1994 11:111–6
37.	Haug NA, DiClemente C, Svikis DS. Motivational enhancement therapy for nicotine dependence in methadone-maintained pregnant women. Psychology of Addictive Behaviours 2004.18(3):289–92
38.	Hegaard H, Hjaergaard H, Moller L, Wachmann H, Ottesen B. Multimodel intervention raises smoking cessation rate during pregnancy. Acta Obstetricia et Gynecologica Scandinavica 2003.82:813–9
39.	Heil SH, Higgins ST, Bernstein IM, Solomon LJ, Rogers RE, Thomas CS, et al. Effects of voucher-based incentives on abstinence from cigarette smoking and fetal growth among pregnant women. Addiction. 2008.103(6):1009–18
40.	Hennrikus D, Pirie P, Hellerstedt W, Lando HA, Steele J, Dunn C. Increasing support for smoking cessation during pregnancy and postpartum: results of a randomized controlled pilot study. Preventive Medicine 2010.50(3): 134–7
41.	Herbec A, Brown J, Tombor I, Michie S, West R. Pilot randomized controlled trial of an internet-based smoking cessation intervention for pregnant smokers ('MumsQuit'). Drug & Alcohol Dependence 2014.140:130–6
42.	Hiett A, Brazus S, Hedberg J, Brown H. Smoking cessation program effectiveness during pregnancy. American Journal of Obstetrics and Gynecology 2000.182(1 Pt 2):S150
43.	Higgins ST, Bernstein IM, Washio Y, et al. Effects of smoking cessation with voucher-based contingency management on birth outcomes. Addiction. 2010; 105(11):2023–2030.
44.	Higgins ST, Washio Y, Lopez AA, Heil SH, Solomon LJ, Lynch ME, et al. Examining two different schedules of financial incentives for smoking cessation among pregnant women. Preventive Medicine 2014 Vol 68:51–7
45.	Hjalmarson AIM, Svanberg B, Hahn L. Stopping smoking in pregnancy: effect of a self-help manual in a controlled trial. British Journal of Obstetrics and Gynaecology 1991.98: 260–4
46.	Hughes E, Lamont D, Beecroft M, Wilson D. Randomized trial of a "stage-of-change" orientated smoking cessation intervention in infertile and pregnant women. Fertility and Sterility 2000.74(3):498–503

Smoking unique publications	
47.	Kemp L, Harris E, McMahon C, Matthey S, Vimpani G, Anderson T, Schmied V, Aslam H, Zapart S. Child and family outcomes of a long-term nurse home visitation programme: a randomised controlled trial. Archives of Disease in Childhood 2011.96(6):533–540
48.	Kendrick JS, Metzger RW, Sexton M, Spierto FW, Floyd RL, Gargiullo PM, et al. Integrating smoking cessation into routine public prenatal care: the Smoking Cessation in Pregnancy project. American Journal of Public Health 1995 85:217–22
49.	Lawrence T, Aveyard P, Croghan E. What happens to women's self-reported cigarette consumption and urinary cotinine levels in pregnancy? Addiction 2003.98:1315–20
50.	Lee M, Miller SM, Wen KY, Hui SA, Roussi P, Hernandez E. Cognitive-behavioral intervention to promote smoking cessation for pregnant and postpartum inner city women. Journal of Behavioral Medicine 2015.38(6):923–43
51.	LeFevre ML, Ewigman B, Evans JK. Is smoking an indication for prenatal ultrasonography? RADIUS Study Group. Archives of Family Medicine 1995.4:120–3
52.	Lilley J, Forster DP. A randomised controlled trial of individual counselling of smokers in pregnancy. Public Health 1986.100:309–15
53.	Lillington L, Chlebowski R, Ruvalcaba M, Novak D, Royce J. Evaluation of a smoking cessation program for pregnant minority smokers. Cancer Practice 1995.3(3):157–63
54.	Loeb BK, Waage G, Bailey J. Smoking intervention in pregnancy. Proceedings of the Fifth World Conference on Smoking and Health 1983 July Winnipeg, Canada. 1983: 389–95
55.	Loukopoulou A, Vardavas C, Tzatzarakis M, Farmakides G, Rossolymos C, Chrelias C, et al. Urinary nicotine and cotinine as a method for assessing the effectiveness of an intervention to aid smoking cessation during pregnancy. Chest 2012.142(4 Suppl 1):1081A
56.	Lowe JB, Balanda KP, Stanton WR, Del Mar C, O'Connor V. Dissemination of an efficacious antenatal smoking cessation program in public hospitals in Australia: a randomised controlled trial. Health Education & Behavior 2002.29(5):608–19
57.	Lowe JB, Windsor R, Balanda K, Woodby L. Smoking relapse prevention methods for pregnant women: a formative evaluation. American Journal of Health Promotion 1997.11:244–6
58.	MacArthur C, Knox EG, Lancashire RJ. Effects at age nine of maternal smoking in pregnancy: experimental and observational findings. BJOG. 01;108(1):67–73.
59.	Malchodi CS, Oncken C, Dornelas EA, Caramanica L, Gregonis E. The effects of peer counselling on smoking cessation and reduction. Obstetrics & Gynecology 2003.101 (3):504–10
60.	Manfredi C, Crittenden KS, Warnecke R, Engler J, Cho YI, Shaligram C. Evaluation of a motivational smoking cessation intervention for women in public health clinics. Preventive Medicine 1999.28:51–60
61.	Mauriello L, Dyment S, Prochaska J, Gagliardi A, Weingrad-Smith J. Acceptability and feasibility of a multiple-behaviour, computer-tailored intervention for underserved pregnant women. Journal of Midwifery and Women's Health 2011.56:75–80
62.	Mayer JP, Todd R, Hawkins B. A randomised evaluation of smoking cessation interventions for pregnant women at a WIC clinic. American Journal of Public Health 1990.80: 76–7
63.	McBride CM, Baucom DH, Peterson BL, Pollak KI, Palmer C, Westman E, et al. Prenatal and postpartum smoking abstinence: a partner assisted approach. American Journal of Preventive Medicine 2004.27(3):232–8
64.	McBride CM, Curry SJ, Lando HA, Pirie PL, Grothaus LC, Nelson JC. Prevention of relapse in women who quit smoking during pregnancy. American Journal of Public Health 1999.89:706–11
65.	McLeod D, Pullon S, Benn C, Cookson T, Dowell A, Viccars A, et al. Can support and education for smoking cessation and reduction be provided effectively by midwives within primary maternity care? Midwifery 2004.20:37–50
66.	Mejdoubi J, van den Heijkant SCCM, van Leerdam FJM, Crone M, Crijnen A, HiraSing RA. Effects of nurse home visitation on cigarette smoking pregnancy outcomes: a randomized controlled trial. Midwifery 2014.30:688–95
67.	Messimer SR, Henry RC, Hickner JM. A comparison of two antismoking interventions among pregnant women in eleven primary care practices. Journal of Family Practice 1989.28(3):283–8
68.	Moore LO, Campbell R, Whelan A, Mills N, Lupton P, Misselbrook E, et al. Self help smoking cessation in pregnancy: cluster randomised controlled trial. BMJ 2002 325:1383–6
69.	Moore ML, Meis PJ, Ernest JM, Wells HB, Zaccaro DJ, Terrell T. A randomized trial of nurse intervention to reduce preterm and low birth weight births. Obstetrics & Gynecology 1998.91:656–61
70.	Naughton F, Prevost AT, Gilbert H, Sutton S. Randomized controlled trial evaluation of a tailored leaflet and SMS text message self-help intervention for pregnant smokers (MiQuit). Nicotine and Tobacco Research 2012.14(5): 569–77
71.	Olds D, Robinson J, O'Brien R, Luckey D, Pettit L, Henderson C, et al. Home visiting by paraprofessionals and by nurses: a randomized, controlled trial. Pediatrics 2002 110(3):486–96
72.	Olds DL, Henderson CR, Chamberlin R, Tatelbaum R. Preventing child abuse and neglect: a randomized trial of nurse home visitation. Pediatrics 1986.78:65–78
73.	Oncken C, Dornelas E, Greene J, et al. Nicotine gum for pregnant smokers: a randomized controlled trial. Obstet Gynecol. 2008;112(4):859–867.
74.	Ondersma SJ, Svikis DS, Lam PK, Connors-Burge VS, Ledgerwood DM, Hopper JA. A randomized trial of computer-delivered brief intervention and low-intensity contingency management for smoking during pregnancy. Nicotine & Tobacco Research 2012 Vol 14, issue 3: 351–60
75.	Oude Wesselink SF, Lingsma HF, Reulings PG, Wentzel HR, Erasmus V, Robben PB, et al. Does government supervision improve stop-smoking counseling in midwifery practices? Nicotine & Tobacco Research 2015 Vol 17, issue 5:572–9
76.	Panjari M, Bell R, Bishop S, Astbury J, Rice G, Doery J. A randomized controlled trial of a smoking cessation intervention during pregnancy. Australian and New Zealand Journal of Obstetrics and Gynaecology. 1999.39(3):312–7
77.	Parker DR, Windsor RA, Roberts MB, Hecht J, Hardy NV, Strolla LO, et al. Feasibility, cost, and cost effectiveness of a telephone-based motivational intervention for underserved pregnant smokers. Nicotine and Tobacco Research 2007.9(10):1043–51
78.	Patten CA, Windsor RA, Renner CC, Enoch C, Hochreiter A, Nevak C, et al. Feasibility of a tobacco cessation intervention for pregnant Alaska Native women. Nicotine & Tobacco Research 2009.12(2):79–87
79.	Pbert L, Ockene JK, Zapka J, Ma Y, Goins KV, Oncken C, et al. A community health center smoking cessation intervention for pregnant and postpartum women. American Journal of Preventive Medicine 2004.26(5): 377–85
80.	Peterson L, Rosen A, Podedworny T, Kotch J, Handel J. Smoking reduction during pregnancy by a program of self-help and clinical support. Obstetrics & Gynecology 1992.79: 924–30
81.	Polanska K, Hanke W, Sobala W, et al. Efficacy and effectiveness of the smoking cessation program for pregnant women. Int J Occup Med Environ Health. 2004;17(3):369–377.
82.	Polanska K, Hanke W, Sobala W, Lowe JB. Efficacy and effectiveness of the smoking cessation program for pregnant women. International Journal of Occupational Medicine and Environmental Health 2004.17(3):369–77

Smoking unique publications	
83.	Pollak KI, Lyna P, Bilheimer A, Farrell D, Gao X, Swamy GK, et al. A pilot study testing SMS text delivered scheduled gradual reduction to pregnant smokers. Nicotine & Tobacco Research 2013.15(10):1773–6
84.	Prapavessis H, De Jesus S, Harper T, Cramp A, Fitzgeorge L, Mottola MF, et al. The effects of acute exercise on tobacco cravings and withdrawal symptoms in temporary abstinent pregnant smokers. Addictive Behaviors 2014.39(3):703–8
85.	Price JH, Snyder FF, Roberts SM, Losh DP, Desmond SM, Krol RA. Comparison of three antismoking interventions among pregnant women in an urban setting: a randomized trial. Psychological Reports 1991.68:595–604
86.	Ram A, Tuten M, Chisolm MS. Cigarette smoking reduction in pregnant women with opioid use disorder. J Addict Med. 2016;10(1):53–59.
87.	Reading AE, Cox DN. The effects of ultrasound on maternal anxiety. Journal of Behavioral Medicine 1982.5(2): 237–47
88.	Rigotti N, Park ER, Regan S, Chang Y, Perry K, Loudin B, et al. Efficacy of telephone counseling for pregnant smokers. Obstetrics & Gynecology 2006.108(1):83–92
89.	Robling M, Bekkers MJ, Bell K, Butler CC, CanningsJohn R, Channon S, et al. Effectiveness of a nurse-led intensive home-visitation programme for first-time teenage mothers (Building Blocks): a pragmatic randomised controlled trial. Lancet 2016.387(10014):146–55
90.	Ruger JP, Weinstein MC, Hammond SK, et al. Cost Effectiveness of motivational interviewing for Smoking Cessation and relapse prevention among low-income pregnant women: a randomized controlled trial. Value Health J Int Soc Pharmacoecon Outcomes Res. 2008;11(2):191–198.
91.	Secker-Walker RH, Mead PB, Goodwin GD, Lepage SS, Skelly JM, Flynn BS, et al. Individualised smoking cessation counseling during prenatal and early postnatal care. American Journal of Obstetrics and Gynecology 1994 71:1347–55
92.	Secker-Walker RH, Solomon LJ, Flynn BS, Skelly JM, Mead PB. Reducing smoking during pregnancy and postpartum: physician's advice supported by individual counselling. Preventive Medicine 1998.27:422–30
93.	Secker-Walker RH, Solomon LJ, Geller BM, Flynn BS, Worden JK, Skelly JM, et al. Modeling smoking cessation: exploring the use of a videotape to help pregnant women quit smoking. Women & Health 1997.25:23–35
94.	Sexton M, Hebel JR. A clinical trial of change in maternal smoking and its effect on birth weight. JAMA 1984.251: 911–5
95.	Solomon LJ, Secker-Walker RH, Flynn BS, Skelly JM, Capeless EL. Proactive telephone peer support to help pregnant women stop smoking. Tobacco Control 2000.9 Suppl 3:iii72–iii74
96.	Stotts AL, DeLaune KA, Schmitz JM, Grabowski J. Impact of a motivational intervention on mechanisms of change in low-income pregnant women. Addictive Behaviors 2004.29 (8):1649–57
97.	Stotts AL, Groff JY, Velasquez MM, Benjamin-Garner R, Green C, Carbonari JP, et al. Ultrasound feedback and motivational interviewing targeting smoking cessation in the second and third trimesters of pregnancy. Nicotine & Tobacco Research 2009.11(8):961–8
98.	Stotts S, DiClemente CC, Dolan-Mullen P. One-to-One: a motivational intervention for resistant pregnant smokers. Addictive Behaviors 2002.27:275–92
99.	Strecher VJ, Bishop KR, Bernhardt J, Thorp JM, Cheuvrout B, Potts P. Quits for keeps: tailored smoking cessation guides for pregnancy and beyond. Tobacco Control 2000.9 (Suppl 3):iii78–iii79
100.	Tappin D, Bauld L, Purves D, Boyd K, Sinclair L, MacAskill S, et al. Financial incentives for smoking cessation in pregnancy: randomised controlled trial. BMJ (Clinical Research Ed.) 2015.350:h134
101.	Tappin DM, Lumsden MA, Gilmour WH, Crawford F, McIntyre D, Stone DH, et al. Randomised controlled trial of home based motivational interviewing by midwives to help pregnant smokers quit or cut down. BMJ 2005.331: 373–7
102.	Tappin DM, Lumsden MA, McIntyre D, McKay C, Gilmour WH, Webber R, et al. A pilot study to establish a randomized trial methodology to test the efficacy of a behavioural intervention. Health Education Research 2000 15(4):491–502
103.	Thornton L. Smoking and Pregnancy: Feasibility and Effectiveness of a Smoking Intervention Programme Among Pregnant Women [thesis]. Dublin: Dept of Public Health, 1997
104.	Tsoh JY, Kohn MA, Gerbert B. Promoting smoking cessation in pregnancy with Video Doctor plus provider cueing: a randomized trial. Acta Obstetricia et Gynecologica Scandinavica 2010.89(4):515–23
105.	Tuten M, Fitzsimons H, Chisolm MS, Nuzzo PA, Jones HE. Contingent incentives reduce cigarette smoking among pregnant, methadone-maintained women: results of an initial feasibility and efficacy randomized clinical trial. Addiction 2012.107(10):1868–77
106.	Ussher M, Lewis S, Aveyard P, et al. The London Exercise and Pregnant smokers (LEAP) trial: a randomised controlled trial of physical activity for smoking cessation in pregnancy with an economic evaluation. Health Technol Assess. 2015;19(84):vii–xxiv, 1
107.	Ussher M, Lewis S, Aveyard P, Manyonda I, West R, Lewis B, et al. Physical activity for smoking cessation in pregnancy: randomised controlled trial. BMJ (Clinical Research Ed.) 2015.350:h2145
108.	Valbo A, Eide T. Smoking cessation in pregnancy: the effect of hypnosis in a randomized study. Addictive Behaviors 1996.21:29–35
109.	Valbo A, Nylander G. Smoking cessation in pregnancy: intervention among heavy smokers. Acta Obstetricia et Gynecologica Scandinavica 1994.73:215–9
110.	Vilches P. Consumption of Tobacco in Pregnant Women: Proposal of a Psychological Intervention Model in the Public Health System of Andalucia [thesis] Malaga: Univesidad di Malaga, 2009
111.	Walsh RA, Melmeth A, Byrne JM, Brinsmead MW, Redman S. A smoking cessation program at a public antenatal clinic. American Journal of Public Health 1997.87: 1201–4
112.	Wilkinson SA, McIntyre HD. Evaluation of the 'healthy start to pregnancy' early antenatal health promotion workshop: a randomized controlled trial. BMC Pregnancy and Childbirth 2012.0.590972222222222
113.	Windsor R, Woodby L, Miller T, Hardin M. Effectiveness of Smoking Cessation and Reduction in Pregnancy Treatment (SCRIPT) methods in Medicaid-supported prenatal care: Trial III. Health Education & Behavior 2011.38(4):412–22
114.	Windsor RA, Lowe JB, Perkins LL, Smith-Yoder D, Artz L, Crawford M, et al. Health education for pregnant smokers: its behavioral impact and cost benefit. American Journal of Public Health 1993.83:201–6
115.	Windsor RA, Spanos D, Samuelsson C, Bartlett EE, Manzella B, Reese Y, et al. The effectiveness of smoking cessation methods for smokers in public health maternity clinics: a randomized trial. American Journal of Public Health 11985.75:1389–92
116.	Wisborg K, Henriksen TB, Jespersen LB, et al. Nicotine patches for pregnant smokers: a randomized controlled study. Obstet Gynecol. 2000;96(6):967–971.

S7c: Included studies in diet and/or physical activity reviews

Review	References of included behavioural interventions delivered in pregnancy
Agha *et al.* 2014 [3]	1. Phelan S, Phipps MG, Abrams B, Darroch F, Schaffner A, et al. (2011) Randomized trial of a behavioural intervention to prevent excessive gestational weight gain: The Fit for delivery study. American Journal of Clinical Nutrition. 2011; 93(4): 772–779<br>2. Jackson RA, Stotland NE, Caughey AB, Gerbert B (2011) Improving diet and exercise in pregnancy with Video Doctor counseling: A randomized trial. Patient Education and Counselling. 83(2): 203–209.<br>3. Ilmonen J, Isolauri E, Poussa T, Laitinen K (2011) Impact of dietary counselling and probiotic intervention on maternal anthropometric measurements during and after pregnancy: A randomized placebo-controlled trial. Clinical Nutrition. 2011; 30(2): 156–164.<br>4. Guelinckx I, Devlieger R, Mullie P, Vansant G (2010) Effect of lifestyle intervention on dietary habits, physical activity, and gestational weight gain in obese pregnant women: a randomized controlled trial. Am J Clin Nutr. 91(2): 373–380.<br>5. Wolff S, Legarth J, Vangsgaard K, Toubro S, Astrup A (2008). A randomized trial of the effects of dietary counseling on gestational weight gain and glucose metabolism in obese pregnant women. Int J Obes (Lond); 32(3): 495–501.<br>6. Polley BA, Wing RR, Sims CJ (2002). Randomized controlled trial to prevent excessive weight gain in pregnant women. Int J Obes Relat Metab Disord; 26(11): 1494–1502.<br>7. Hui AL, Ludwig SM, Gardiner P, Sevenhuysen G, Murray R, et al (2006). Community-based exercise and dietary intervention during pregnancy: A pilot study. Canadian Journal of Diabetes;30(2): 169–175.<br>8. Haakstad LA, Bø K (2011). Effect of regular exercise on prevention of excessive weight gain in pregnancy: A randomised controlled trial. The European Journal of Contraception and Reproductive Health Care;16: 116–125.<br>9. Kinnunen TI, Pasanen M, Aittasalo M, Fogelholm M, Hilakivi-Clarke L, et al. (2007) Preventing excessive weight gain during pregnancy – a controlled trial in primary health care. Eur J Clin Nutr. 61(7): 884–891.<br>10. Claesson IM, Sydsjo¨ G, Brynhildsen J, Cedergren M, Jeppsson A, et al. (2008) Weight gain restriction for obese pregnant women: a case-control intervention study. BJOG. 115(1): 44–50.
Bain *et al.* 2015 [4]	1. Asbee SM, Jenkins TR, Butler JR, White J, Elliot M, Rutledge A. Preventing excessive weight gain during pregnancy through dietary and lifestyle counseling: a randomized controlled trial. Obstetrics and Gynecology 2009;113(2 Pt 1):305–12.<br>2. Dodd JM, Turnbull D, McPhee AJ, Deussen AR, Grivell RM, Yelland LN, et al. Antenatal lifestyle advice for women who are overweight or obese: LIMIT randomised trial. BMJ 2014;348:g1285.<br>3. El Beltagy N, El Deen SS, Mohamed R. Does physical activity and diet control reduce the risk of developing gestational diabetes mellitus in Egypt? A randomized controlled trial. Journal of Perinatal Medicine 2013;41 (Suppl 1):1176.<br>4. Harrison CL, Lombard CB, Strauss BJ, Teede HJ. Optimizing healthy gestational weight gain in women at high risk of gestational diabetes: a randomized controlled trial. Obesity 2013;21(5):904–9.<br>5. Hui A, Back L, Ludwig S, Gardiner P, Sevenhuysen G, Dean H, et al. Lifestyle intervention on diet and exercise reduced excessive gestational weight gain in pregnant women under a randomised controlled trial. BJOG: An International Journal of Obstetrics & Gynaecology 2012;119 (1):70–7.<br>6. Korpi-Hyovalti EA, Laaksonen DE, Schwab US, Vanhapiha TH, Vihla KR, Heinonen ST, et al. Feasibility of a lifestyle intervention in early pregnancy to prevent deterioration of glucose tolerance. BMC Public Health 2011;11:179.<br>7. Luoto R, Kinnunen TI, Aittasalo M, Kolu P, Raitanen J, Ojala K, et al. Primary prevention of gestational diabetes mellitus and large-for-gestational-age newborns by lifestyle counseling: a cluster-randomized controlled trial. PLoS Medicine 2011;8(5):1–11.<br>8. Petrella E, Facchinetti F, Bertarini V, Pignatti L, Neri I, Battistini NC. Occurrence of pregnancy complications in women with BMI > 25 submitted to a healthy lifestyle and eating habits program. American Journal of Obstetrics and Gynecology 2013;208(1 Suppl):S33–4.<br>9. Phelan S, Phipps MG, Abrams B, Darroch F, Schaffner A, Wing RR. Randomized trial of a behavioral intervention to prevent excessive gestational weight gain: the Fit for Delivery Study. American Journal of Clinical Nutrition 2011;93(4):772–9.<br>10. Polley BA, Wing RR, Sims CJ. Randomized controlled trial to prevent excessive weight gain in pregnant women. International Journal of Obesity & Related Metabolic Disorders: Journal of the International Association for the Study of Obesity 2002;26(11):1494–502.<br>11. Poston L, Briley AL, Barr S, Bell R, Croker H, Coxon K, et al. Developing a complex intervention for diet and activity behaviour change in obese pregnant women (the UPBEAT trial); assessment of behavioural change and process evaluation in a pilot randomised controlled trial. BMC Pregnancy and Childbirth 2013;13(1):148.<br>12. Rauh K, Gabriel E, Kerschbaum E, Schuster T, von Kries R, Amann-Gassner U, et al. Safety and efficacy of a lifestyle intervention for pregnant women to prevent excessive maternal weight gain: a cluster-randomized controlled trial. BMC Pregnancy and Childbirth 2013;13(1):151.<br>13. Vinter C, Jensen D, Ovesen P, Beck-Nielsen H, Lamont R, Jorgensen J. Postpartum weight retention and breastfeeding among obese women from the LiP (Lifestyle in Pregnancy) Study. Acta Obstetricia et Gynecologica Scandinavica 2012; 91(Suppl 159):141–2.

Review	References of included behavioural interventions delivered in pregnancy
Allen *et al.* 2014 [5]	1. Bogaerts A, Devlieger R, Nuyts E, Witters I, Gyselaers W, Van den Bergh BRH. Effects of lifestyle intervention in obese pregnant women on gestational weight gain and mental health: a randomised controlled trial. Int J Obesity. 2012;36:1–8.<br>2. Crowther C, Hiller J, Moss J, McPhee A, Jeffries W, Robinson J. Effect of treatment of gestational diabetes mellitus on pregnancy outcomes. N Engl J Med. 2005;352:2477–86.<br>3. D'Almeida A, Carter JP, Anatol A, Prost C. Effects of a combination of evening primrose oil (gamma linolenic acid) and fish oil (eicosapentaenoic + docahexaenoic acid) versus magnesium, and versus placebo in preventing preeclampsia. Women Health. 1992;19:117–31.<br>4. Guelinckx I, Devlieger R, Mullie P, Vansant G. Effect of lifestyle intervention on dietary habits, physical activity and gestational weight gain in obese pregnant women: a randomised controlled trial. Am J Clin Nutr. 2010;91: 373–80.<br>5. Jefferies K, Shub A, Walker S, Hiscock R, Permezel M. Reducing excessive weight gain in pregnancy: a randomised controlled trial. Med J Aust. 2009;191: 429–33.<br>6. Khoury J, Henriksen T, Christophersen B, Tonstad S. Effect of cholesterol-lowering diet on maternal, cord and neonatal lipids, and pregnancy outcome: a randomised clinical trial. Am J Obstet Gynecol. 2005;193:1292–301.<br>7. Landon M, Spong C, Thom E, Carpenter MW, Ramins S, Casey B, et al. A multicenter, randomised trial of treatment for mild gestational diabetes. N Engl J Med. 2009;361:1339–48.<br>8. Olsen SF, Secher NJ, Tabor A, Weber T, Walker JJ, Gluud C. Randomised clinical trials of fish oil supplementation in high risk pregnancies. BJOG. 2000;107:382–95.<br>9. Onwude JL, Lilford RJ, Hjartardottir H, Staines A, Tuffnell D. A randomised double blind placebo controlled trial of fish oil in high risk pregnancy. BJOG. 1995;102:95–100.<br>10. Phelan S, Phipps M, Abrams B, Darroch F, Schaffner A, Wing R. Randomised trial of behavioural intervention to prevent excessive gestational weight gain: the fit for delivery study. Am J Clin Nutr. 2011;93:772–9.<br>11. Polley B, Wing R, Sims C. Randomised controlled trial to prevent excessive weight gain in pregnant women. Int J Obesity. 2002;26:1494–502.<br>12. Rae A, Bond D, Evans S, North F, Roberman B, Walters B. A randomised controlled trial of dietary energy restriction in the management of obese women with gestational diabetes. Aust N Z J Obstet Gynaecol. 2000;40:416–22.<br>13. Salvig J, Olsen S, Secher N. Effects of fish oil supplementation in late pregnancy on blood pressure: a randomised controlled trial. BJOG. 1996;103:529–33.<br>14. Smuts CM, Huang M, Mundy D, Plasse T, Major S, Carlson SE. A randomised trial of docosahexaenoic acid supplementation during the third trimester of pregnancy. Obstet Gynecol. 2003;101:469–79.<br>15. Thornton Y, Smarkola C, Kopacz SM, Ishoof S. Perinatal outcomes in nutritionally monitored obese pregnant women: a randomised clinical trial. J Natl Med Assoc. 2009;101:569–77.<br>16. Vinter C, Jensen DM, Oveson P, Henning B-N, Jorgensen JS. The LiP (Lifestyle in Pregnancy) study. Diabetes Care. 2011;34:2502–7.<br>17. Wolff S, Legarth J, Vangsgaard K, Toubro S, Astrup A. A randomised trial of the effects of dieatry counseling on gestational weight gain and glucose metabolism in obese pregnant women. Int J Obesity. 2008;32:495–501<br>18. Zhou S, Yelland L, McPhee A, Quinlivan J, Gibson R, Makrides M. Fish oil supplementation in pregnancy does not reduce the risk of gestational diabetes or preeclampsia. Am J Clin Nutr. 2012;95:1378–84.
Campbell *et al.* 2011 [6]	1. Guelinckx I, Devlieger R, Mullie P, Vansant G: Effect of lifestyle intervention on dietary habits, physical activity, and gestational weight gain in obese pregnant women: a randomized controlled trial. 2010, 91:373-380.<br>2. Wolff S, Legarth J, Vangsgaard K, Toubro S, Astrup A: A randomized trial of the effects of dietary counseling on gestational weight gain and glucose metabolism in obese pregnant women. Int J Obes (Lond) 2008, 32:495-501.<br>3. Asbee SM, Jenkins TR, Butler JR, White J, Elliot M, Rutledge A: Preventing Excessive Weight Gain During Pregnancy Through Dietary and Lifestyle Counseling. A Randomized Controlled Trial. Obstetrics and Gynecology 2009, 113:305-311.<br>4. Polley BA, Wing RR, Sims CJ, Polley BA, Wing RR, Sims CJ: Randomized controlled trial to prevent excessive weight gain in pregnant women. Int J Obes Relat Metab Disord 2002, 26:1494-1502.<br>5. Hui AL, Ludwig SM, Gardiner P, Sevenhuysen G, Murray R, Morris M, et al: Community-based exercise and dietary intervention during pregnancy: a pilot study. CAN J DIABETES 2006, 30:169-175.
Choi *et al.* 2013 [7]	1. Barakat, R., Lucia, A., Ruiz, J.R., 2009. Resistance exercise training during pregnancy and newborn's birth size: a randomised controlled trial. Int. J. Obes. 33, 1048–1057.<br>2. Ong, M.J., Guelfi, K.J., Hunter, T., Wallman, K.E., Fournier, P.A., Newnham, J.P., 2009. Supervised home-based exercise may attenuate the decline of glucose tolerance in obese pregnant women. Diabetes Metab. 35, 418–421.<br>3. Nascimento, S.L., Surita, F.G., Parpinelli, M.Â., Siani, S., Pinto e Silva, J.L., 2011. The effect of an antenatal physical exercise program on maternal/perinatal outcomes and quality of life in overweight and obese pregnant women: a randomized clinical trial. BJOG: Int. J. Obstet. Gynaecol. 118, 1455–1463.<br>4. Vinter, C.A., Jensen, D.M., Ovesen, P., Beck-Nielsen, H., Jørgensen, J.S., 2011. The LiP (Lifestyle in Pregnancy) Study: a randomized controlled trial of lifestyle intervention in 360 obese pregnant women. Diabetes Care 34, 2502–2507.<br>5. Guelinckx, I., Devlieger, R., Mullie, P., Vansant, G., 2010. Effect of lifestyle intervention on dietary habits, physical activity, and gestational weight gain in obese pregnant women: a randomized controlled trial. Am. J. Clin. Nutr. 91, 373–380.<br>6. Phelan, S., Phipps, M.G., Abrams, B., Darroch, F., Schaffner, A., Wing, R.R., 2011. Ran- domized trial of a behavioral intervention to prevent excessive gestational weight gain: the Fit for Delivery Study. Am. J. Clin. Nutr. 93, 772–779.<br>7. Polley, B.A., Wing, R.R., Sims, C.J., 2002. Randomized controlled trial to prevent exces- sive weight gain in pregnant women. Int. J. Obes. 26, 1494–1502.

Review	References of included behavioural interventions delivered in pregnancy
Elliot-Sale *et al.* 2015 [8]	1. Barakat R, Pelaez M, Montejo R, et al. Exercise during pregnancy improves maternal health perception: a randomized controlled trial. Am J Obstet Gynecol 2011;204:402. e1–7.<br>2. Haakstad LAH, Bo K. Effect of regular exercise on prevention of excessive weight gain in pregnancy: a randomised controlled trial. Eur J Contracept Reprod Health Care 2011;16:116–25.<br>3. Nascimento SL, Surita FG, Parpinelli MA, et al. The effect of an antenatal physical exercise programme on maternal/perinatal outcomes and quality of life in overweight and obese pregnant women: a randomised clinical trial. BJOG 2011;118:1455–63.
Gardner *et al.* 2011 [9]	1. Claesson IM, Sydsjö G, Brynhildsen J, Cedergren M, Jeppsson A, Nyström F, Sydsjö A, Josefsson A. Weight gain restriction for obese pregnant women: a case-control intervention study. Br J Obstet Gynaecol 2007; 115: 44–50.<br>2. Hui AL, Ludwig SM, Gardiner P, Sevenhuysen G, Murray R, Morris M, Shen GX. Community-based exercise and dietary intervention during pregnancy: a pilot study. Can J Diabetes 2006; 30: 169–175.<br>3. *Polley BA, Wing RR, Cims CJ. Randomized controlled trial to prevent excessive weight gain in pregnant women. Int J Obes (Lond) 2002; 26: 1494–1502.<br>4. Gray-Donald K, Robinson E, Collier A, David K, Renaud L, Rodrigues S. Intervening to reduce weight gain in pregnancy and gestational diabetes mellitus. CMAJ 2000; 163: 1247–1251.<br>5. Kinnunen TI, Pasanen M, Aittasalo M, Fogelholm M, Hilakivi-Clarke L, Weiderpass E, Luoto R. Preventing excessive weight gain during pregnancy: a controlled trial in primary health care. Eur J Clin Nutr 2007; 61: 884–892.<br>6. Wolff S, Legarth J, Vangsgaard K, Toubro S, Astrup A. A randomized trial of the effects of dietary counselling on gestational weight gain and glucose metabolism in obese pregnant women. Int J Obes (Lond) 2008; 32: 495–501.<br>7. Asbee SM, Jenkins TR, Butler JR, White J, Elliot M, Rutledge A. Preventing excessive gestational weight during pregnancy through dietary and lifestyle counselling. Obstet Gynecol 2009; 113: 305–311.<br>8. Shirazian T, Monteith S, Friedman F, Rebarber A. Lifestyle modification program decreases pregnancy weight gain in obese women. Am J Perinat 2010; 27: 411–414<br>9. *Guelinckx I, Devlieger R, Mullie P, Vansant G. Effect of lifestyle intervention on dietary habits, physical activity, and gestational weight gain in obese pregnant women. Am J Clin Nut 2010; 91: 373–380.<br>10. Olson CM, Strawderman MS, Reed RG. Efficacy of an intervention to prevent excessive gestational weight gain. Am J Obstet Gynecol 2004; 191: 530–536.<br><br>*** Note: References 3 and 9 were included in meta-analysis twice**
Gresham *et al.* 2016 [10]	1. Asbee S.M., Jenkins T.R., Butler J.R.,White J., Elliot M. & Rutledge A. (2009) Preventing excessive weight gain during pregnancy through dietary and lifestyle counseling: a randomized controlled trial. Obstetrics and Gynecology 113, 305–312.<br>2. Bonomo M., Corica D., Mion E., Goncalves D., Motta G., Merati R. et al. (2005) Evaluating the therapeutic approach in pregnancies complicated by borderline glucose intolerance: a randomized clinical trial. Diabetic Medicine 22, 1536–1541.<br>3. Briley C., Flanagan N.L. & Lewis N. (2002) In-home prenatal nutrition intervention increased dietary iron intakes and reduced low birthweight in low-income African-American women. Journal of the American Dietetic Association 102 (7), 984–987.<br>4. Chan G.M., McElligott K., McNaught T. & Gill G. (2006) Effects of dietary calcium intervention on adolescent mothers and newborns: a randomized controlled trial. Obstetrics and Gynecology 108, 565–571.<br>5. Kafatos A., Vlachonikolis I. & Codrington C. (1989) Nutrition during pregnancy: the effects of an educational intervention program in Greece. The American Journal of Clinical Nutrition 50, 970–979.<br>6. Khoury J., Henriksen T., Christophersen B. & Tonstad S. (2005) Effect of a cholesterol-lowering diet on maternal, cord, and neonatal lipids, and pregnancy outcome: a randomized clinical trial. American Journal of Obstetrics and Gynecology 193, 1292–1301.<br>7. Knuist M., Bonsel G.J., Zondervan H.A. & Treffers P.E. (1998) Low sodium diet and pregnancy-induced hypertension: a multi-centre randomised controlled trial. British Journal of Obstetrics and Gynaecology 105, 430–434.<br>8. Luoto R., Kinnunen T.I., Aittasalo M., Kolu P., Raitanen J., Ojala K. et al. (2011) Primary prevention of gestational diabetes mellitus and large-for-gestational-age newborns by lifestyle counseling: a cluster-randomized controlled trial. PLoS (Public Library of Science) Medicine 8, e1001036'.<br>9. O'Connor M.J. & Whaley S.E. (2007) Brief intervention for alcohol use by pregnant women. American Journal of Public Health 97, 252–258.<br>10. Quinlivan J.A., Lam L.T. & Fisher J. (2011) A randomised trial of a four-step multidisciplinary approach to the antenatal care of obese pregnant women. Australian & New Zealand Journal of Obstetrics & Gynaecology 51, 141–146.<br>11. Thornton Y.S., Smarkola C., Kopacz S.M. & Ishoof S.B. (2009) Perinatal outcomes in nutritionally monitored obese pregnant women: a randomized clinical trial. Journal of the National Medical Association 101, 569– 577.<br>12. Van Buul B.J.A., Steegers E.A.P., Van Der Maten G.D., Delemarre F.M.C., Jongsma H.W., Oosterbaan H.P. et al. (1997) Dietary sodium restriction does not prevent gestational hypertension: a Dutch two-center randomized trial. Hypertension in Pregnancy 16, 335–346.<br>13. Vinter C.A., Jensen D.M., Ovesen P., Beck-Nielsen H. & Jørgensen J.S. (2011) The LiP (Lifestyle in Pregnancy) study: a randomized controlled trial of lifestyle intervention in 360 obese pregnant women. Diabetes Care 34, 2502–2507.<br>14. Wolff S., Legarth J., Vangsgaard K., Toubro S. & Astrup A. (2008) A randomized trial of the effects of dietary counselling on gestational weight gain and glucose metabolism in obese pregnant women. International Journal of Obesity 32, 495–501.
Han *et al.* 2012 [11]	1. Barakat R, Cordero Y, Coteron J, Luaces M, Montejo R. Exercise during pregnancy improves maternal glucose screen at 24-28 weeks: a randomised controlled trial. British Journal of Sports Medicine 2011 Sep 26. [Epub ahead of print].<br>2. Byrne NM, Groves AM, McIntyre HD, Callaway LK. Changes in resting and walking energy expenditure and walking speed during pregnancy in obese women. American Journal of Clinical Nutrition 2011;94(3):819–30.<br>3. Callaway L. A randomized controlled trial using exercise to reduce gestational diabetes and other adverse maternal and neonatal outcomes in obese pregnant women - the pilot study. Australian Clinical Trials Registry (www.actr.org.au) (accessed 21 June 2007).

Review	References of included behavioural interventions delivered in pregnancy
	4. Callaway L, McIntyre D, Colditz P, Byrne N, Foxcroft K, O'Connor B. Exercise in obese pregnant women: a randomized study to assess feasibility. Hypertension in Pregnancy 2008;27(4):549. ∗<br>5. Callaway LK, Colditz PB, Byrne NM, Lingwood BE, Rowlands IJ, Foxcroft K, et al. Prevention of gestational diabetes. Feasibility issues for an exercise intervention in obese pregnant women. Diabetes Care 2010;33(7):1457–9.<br>6. Hofman P, Hopkins S. Randomised controlled study of the effects of exercise during pregnancy on maternal insulin sensitivity and neonatal outcomes. Australian Clinical Trials Register (http://www.actr.org/actr) (accessed 6 December 2005).<br>7. Hopkins SA, Baldi JC, Cutfield WS, McCowan L, Hofman PL. Effects of exercise training on maternal hormonal changes in pregnancy. Clinical Endocrinology 2011;74(4): 495–500.<br>8. Hopkins SA, Baldi JC, Cutfield WS, McCowan L, Hofman PL. Exercise training in pregnancy reduces offspring size without changes in maternal insulin sensitivity. Journal of Clinical Endocrinology and Metabolism 2010;95 (5):2080–8.<br>9. Ong MJ, Guelfi KJ, Hunter T, Wallman KE, Fournier PA, Newnham JP. Supervised home-based exercise may attenuate the decline of glucose tolerance in obese pregnant women. Diabetes and Metabolism 2009;35(5):418–21.<br>10. Morkved S. Effects of regular exercise during pregnancy. ClinicalTrials.gov (http://clinicaltrials.gov/) (accessed 20 February 2008). ∗ Stafne SN, Salvesen KA, Romundstad PR, Eggebo TM, Carlsen SM, Morkved S. Regular exercise during pregnancy to prevent gestational diabetes: a randomized controlled trial. Obstetrics and Gynecology 2012;119(1):29–36.
Hill *et al.* 2013 [12]	1. Bogaerts AFL, Devlieger R, Nuyts E, Witters I, Gyselaers W, Van den Bergh BRH. Effects of lifestyle intervention in obese pregnant women on gestational weight gain and mental health: a randomized controlled trial. Int J Obes 2012; 1-8. Advance Online Publication. doi: 10.1038/ijo.2012.162<br>2. Haakstad LAH, Bø K. Effect of regular exercise on prevention of excessive weight gain in pregnancy: a randomised controlled trial. European Journal of Contraception & Reproductive Health Care 2011; 16: 116-25.<br>3. Huang T-T, Yeh C-Y, Tsai Y-C. A diet and physical activity intervention for preventing weight retention among Taiwanese childbearing women: a randomised controlled trial. Midwifery 2011; 27: 257-64.<br>4. Hui A, Back L, Ludwig S et al. Lifestyle intervention on diet and exercise reduced excessive gestational weight gain in pregnant women under a randomised controlled trial. Br J Obstet Gynaecol 2012; 119: 70-77.<br>5. Nascimento SL, Surita FG, Parpinelli MÂ, Siani S, Pinto e Silva JL. The effect of an antenatal physical exercise programme on maternal/perinatal outcomes and quality of life in overweight and obese pregnant women: a randomised clinical trial. Br J Obstet Gynaecol 2011; 118: 1455-63.<br>6. Phelan S, Phipps MG, Abrams B, Darroch F, Schaffner A, Wing RR. Randomized trial of a behavioral intervention to prevent excessive gestational weight gain: the Fit for Delivery Study. Am J Clin Nutr 2011; 93: 772-79.<br>7. Ruchat S-M, Davenport MH, Giroux I et al. Nutrition and exercise reduce excessive weight gain in normal-weight pregnant women. Med Sci Sports Exerc 2012; 44(8): 1419-26.<br>8. Vinter CA, Jensen DM, Ovesen PER, Beck-Nielsen H, Jørgensen JS. The LiP (Lifestyle in Pregnancy) Study. Diabetes Care 2011; 34: 2502-07.<br>9. Asbee SM, Jenkins TR, Butler JR, White J, Elliot M, Rutledge A. Preventing excessive weight gain during pregnancy through dietary and lifestyle counseling. Obstet Gynecol 2009; 113: 305-12.<br>10. Claesson IM, Sydsjo G, Brynhildsen J et al. Weight gain restriction for obese pregnant women: a case-control intervention study. Br J Obstet Gynaecol 2008; 115: 44-50.<br>11. Guelinckx I, Devlieger R, Mullie P, Vansant G. Effect of lifestyle intervention on dietary habits, physical activity, and gestational weight gain in obese pregnant women: a randomized controlled trial. Am J Clin Nutr 2010; 91: 373-80.<br>12. Kinnunen TI, Pasanen M, Aittasalo M et al. Preventing excessive weight gain during pregnancy - a controlled trial in primary health care. Eur J Clin Nutr 2007; 61: 884-91.<br>13. Olson CM, Strawderman MS, Reed RG. Efficacy of an intervention to prevent excessive gestational weight gain. Am J Obstet Gynecol 2004; 191: 530-36.<br>14. Polley BA, Wing RR, Sims CJ. Randomized controlled trial to prevent excessive weight gain in pregnant women. Int J Obes Relat Metab Disord 2002; 26: 1494.<br>15. Shirazian T. Lifestyle modification program decreases pregnancy weight gain in obese women. Am J Perinatol 2010; 27: 411-14.<br>16. Wolff S, Legarth J, Vangsgaard K, Toubro S, Astrup A. A randomized trial of the effects of dietary counseling on gestational weight gain and glucose metabolism in obese pregnant women. Int J Obes (Lond) 2008; 32: 495-501.<br>17. Lindholm ES, Norman M, Kilander CP, Altman D. Weight control program for obese pregnant women. Acta Obstet Gynecol Scand 2010; 89: 840-43.<br>18. Mottola MF, Giroux I, Gratton R et al. Nutrition and exercise prevent excess weight gain in overweight pregnant women. Med Sci Sports Exerc 2010; 42: 265-72.
Lamina & Agbanusi 2013 [13]	1. Barakat R, Pelaez M, Montejo R, Luaces M, Zakynthinaki M. Exercise during pregnancy improves maternal health perception: a randomized controlled trial. Am J Obstet Gynecol. 2011;25 [Epub ahead of print] [PubMed]<br>2. Claesson I, Sydsjö G, Brynhildsen J, Blomberg M, Jeppsson A, Sydsjö A, Josefsson A. Weight after childbirth: A 2year follow-up of obese women in a weight-gain restriction program. Acta Obstetricia et Gynecologica Scandinavica. 2011;90(1):103–110. [PubMed]<br>3. Huang T, Yeh C, Tsai Y. A diet and physical activity intervention for preventing weight retention among Taiwanese childbearing women: A randomised controlled trial. Midwifery. 2011;27:257–264. [PubMed]<br>4. Nascimento SL, Surita FG, Parpinelli MA, Siani, Pinto e Silva JL. The effect of an antenatal physical exercise programme on maternal/perinatal outcomes and quality of life in overweight and obese pregnant women: a randomised clinical trial. British Journal of Obstetrics and Gynecology. 2011;118(12):1455–1463. [PubMed]<br>5. Korpi-Hyövälti EAL, Laaksonen DE, Schwab US, Vanhapiha TH, Vihla KR, Heinonen ST, Niskanen LK. Feasibility of a lifestyle intervention in early pregnancy to prevent deterioration of glucose tolerance. BMC Public Health. 2011;11:179. doi: 10.1186/1471-2458-11-179. [PMC free article] [PubMed] [Cross Ref]<br>6. Phelan S, Phipps MG, Abrams B, Darroch F, Schaffner F, Wing RR. Randomized trial of a behavioral intervention to prevent excessive gestational weight gain: the Fit for Delivery Study. Am J Clin Nutr. 2011;93(4):772–779. [PMC free article] [PubMed]<br>7. Asbee SM, Jenkins TR, Butler JR, White J, Elliot M, Rutledge A. Preventing Excessive Weight Gain During Pregnancy Through Dietary and Lifestyle Counseling:A Randomized Controlled Trial. Obstet Gynecol. 2009;113:305–312. [PubMed]<br>8. Clapp JF. Long-term outcome after exercising throughout pregnancy: fitness and cardiovascular risk. Am J Obstet Gynecol. 2008;199(5):489.e1–489.e6. [PMC free article] [PubMed]

Review	References of included behavioural interventions delivered in pregnancy
	9. Wolff S, Legarth J, Vangsgaard K, Toubro S, Astrup A. A randomized trial of the effects of dietary counseling on gestational weight gain and glucose metabolism in obese pregnant women. Int J Obes. 2008;32(3):494–501. [PubMed]<br>10. Haakstad LA, Voldner N, Henriksen T, Bo K. Physical activity level and weight gain in a cohort of pregnant Norwegian women. Acta Obstetricia et Gynecologica Scandinavica. 2007;86:559–564. [PubMed]<br>11. Polley BA, Wing RR, Sims CJ. Randomized controlled trial to prevent excessive weight gain in pregnant women. International Journal of Obesity. 2002;26(11):1494–1502. [PubMed]
Madhuvrata *et al.* 2015 [14]	1. Luoto R, Kinnunen TI, Aittasalo M, et al. Primary prevention of gestational diabetes mellitus and large-for-gestational-age newborns by lifestyle counseling: a cluster-randomized controlled trial. PLoS Med 2011; 8: 1–11.<br>2. Quinlivan JA, Lam LT and Fisher J. A randomised trial of a fourstep multidisciplinary approach to the antenatal care of obese pregnant women. Aust N Z J Obstetr Gynaecol 2011; 51: 141–146.<br>3. Thornton YS, Smarkola C, Kopacz SM, et al. Perinatal outcomes in nutritionally monitored obese pregnant women: a randomized clinical trial. J National Med Assoc 2009; 101: 569–577.<br>4. Wolff S, Legarth J, Vangsgaard K, et al. A randomised trial of the effects of dietary councelling on gestational weight gain and glucose metabolism in obese pregnant women. Int J Obesity 2008; 32: 495–501.<br>5. Callaway LK, Colditz PB, Byrne NM, et al. Prevention of gestational diabetes: feasibility issues for an exercise intervention in obese pregnant women. Diabetes Care 2010; 33: 1457–1459.<br>6. Ong MJ, Guelfi KJ, Hunter T, et al. Supervised home-based exercise may attenuate the decline of glucose tolerance in obese pregnant women. Diabetes Metab 2009; 35: 418–421.<br>7. Guelinckx I, Devlieger R, Mullie P, et al. Effect of lifestyle intervention on dietary habits, physical activity, and gestational weight gain in obese pregnant women: a randomized controlled trial. Am J Clin Nutr 2010; 91: 373–80.<br>8. Vinter CA, Dorte MJ, Ovesen P, et al. The LiP (Lifestyle in Pregnancy) Study: a randomized controlled trial of lifestyle intervention in 360 obese pregnant women. Diabetes Care 2011; 34: 2502–2507.<br>9. Korpi-Hyo¨ va¨ lti EAL, Laaksonen DE, Schwab US, et al. Feasibility of a lifestyle intervention in early pregnancy to prevent deterioration of glucose tolerance. BMC Public Health 2011; 11: 179–186.<br>10. Polley BA, Wing RR and Sims CJ. Randomized controlled trial to prevent excessive weight gain in pregnant women. Int J Obesity 2002; 26: 1494–1502.<br>11. Phelan S, Phipps MG, Abrams B, et al. Randomized trial of a behavioural intervention to prevent excessive gestational weight gain: the Fit for Delivery Study. Am J Clin Nutr 2011; 93: 772–779.<br>12. Vanky E, Salvesen KA, Heimstad R, et al. Metformin reduces pregnancy complications without affecting androgen levels in pregnant polycystic ovary syndrome women: results of a randomized study. Hum Reprod 2004; 19: 1734–1740.<br>13. Vanky E, Solhild S, Hemistad R, et al. Metformin versus placebo from First Trimester to Delivery in Polycystic Ovary Syndrome: a randomized, controlled multicenter study. J Clin Endocrinol Metab 2010; 95: E448–E455.<br>14. Asbee SM, Jenkins TR, Butler JR, et al. Preventing excessive weight gain during pregnancy through dietary and lifestyle counseling a randomized controlled trial. Obstetr Gynaecol 2009; 113:305–312. (WAS Asbee Included?<br>15. Oostdam N, van Poppel M, Wouters M et al. No effect of the FitFor2 exercise programme on blood glucose, insulin sensitivity, and birthweight in pregnant women who were overweight and at risk for gestational diabetes: results of a randomised controlled trial. BJOG 2012; 119(9):1098–1107
Muktabhant *et al.* 2015 [15]	1. Althuizen E, Van Poppel MN, Seidell JC, Van der Wijden C, Van Mechelen W. Design of the new life(style) study: a randomised controlled trial to optimise maternal weight development during pregnancy. *BMC Public Health* 2006;6:168.<br>2. Althuizen E, van der Wijden C, van Mechelen W, SeidellJ, van Poppel M. The effect of a counselling intervention on weight changes during and after pregnancy: a randomised trial. *BJOG : an International Journal of Obstetrics and Gynaecology* 2013;120(1):92–9.<br>3. Broekhuizen K, Althuizen E, van Poppel MNM, Donker M, van Mechelen W. From theory to practice: intervention fidelity in a randomized controlled trial aiming to optimize weight development during pregnancy. *Health Promotion Practice* 2012;13(6):816–25.<br>4. Angel MD, De Haene J, Perez M, Hernandez G, Castaneda D, King JC. Dietary patterns associated with gestational weight gain and fat mass gain in overweight and obese pregnant women. *FASEB Journal* 2011;25:783.15.<br>5. Asbee SM, Jenkins TR, Butler JR, White J, Elliot M, Rutledge A. Dietary counseling prevents excessive weight gain during pregnancy, a randomized controlled trial. *Obstetrics & Gynecology* 2008;11(4 Suppl):6S.<br>6. Asbee SM, Jenkins TR, ButlerJR, White J, Elliot M, Rutledge A. Preventing excessive weight gain during pregnancy through dietary and lifestyle counseling: a randomized controlled trial. *Obstetrics & Gynecology* 2009;113 (2 Pt 1):305–12.<br>7. Barakat R. Effect of physical exercise program during pregnancy on excessive weight gain and its consequences. ClinicalTrials.gov(http://clinicaltrials.gov/) [accessed 21 May 2013] 2011.<br>8. Barakat R, Pelaez M, Montejo R, Luaces M, Zakynthinaki M. Exercise during pregnancy improves maternal health perception: a randomized controlled trial. *American Journal of Obstetrics and Gynecology* 2011;204(5):402.e1–402.e7.<br>9. Bisson M, Almeras N, Dufresne S, Rheaume C, Bujold E, Robitailee J, et al. Exercise improving fitness in obese women during pregnancy: a difference for the mother and child?. Pediatric Academic Societies and Asian Society for Pediatric Research Joint Meeting; 2014 May 3-6;Vancouver, Canada. 2014:Abstract no: 2946.618.<br>10. Bogaerts A. Effect of psycho-education on gestational weight gain and anxiety/depression in obese pregnant women. ClinicalTrials.gov(http://clinicaltrials.gov/) [accessed 21 May 2013] 2011.<br>11. Bogaerts A, Devlieger R, Nuyts E, Witters I, Gyselaers W, Guelinckx I. Psycho-education reduces gestational weight gain in obese pregnant women: Randomized controlled trial [abstract]. *Obesity Facts* 2012;5:53.<br>12. Byrne NM, Groves AM, McIntyre HD, Callaway LK. Changes in resting and walking energy expenditure and walking speed during pregnancy in obese women. *American Journal of Clinical Nutrition* 2011;94(3):819–30.<br>13. Callaway L. A randomized controlled trial using exercise to reduce gestational diabetes and other adverse maternal and neonatal outcomes in obese pregnant women - the pilot study. Australian Clinical Trials Registry (www.actr.org.au/)[accessed 31 October 2010].<br>14. Callaway L, McIntyre D, Colditz P, Byrne N, Foxcroft K, O'Connor B. Exercise in obese pregnant women: a randomized study to assess feasibility. *Hypertension in Pregnancy* 2008;27(4):549.

Review	References of included behavioural interventions delivered in pregnancy
	15. Callaway LK, Colditz PB, Byrne NM, Lingwood BE, Rowlands IJ, Foxcroft K, et al. Prevention of gestational diabetes. Feasibility issues for an exercise intervention in obese pregnant women. *Diabetes Care* 2010;33(7):1457–9.<br>16. Foxcroft KF, Rowlands IJ, Byrne NM, McIntyre HD, Callaway LK, for the BAMBINO group. Exercise in obese pregnant women: the role of social factors, lifestyle and pregnancy symptoms. *BMC Pregnancy and Childbirth* 2011;11:4.<br>17. Clapp III JF. Maternal carbohydrate intake and pregnancy outcome. *Proceedings of the Nutrition Society* 2002; 61(1):45–50.<br>18. Clapp IJF. Diet, exercise, and feto-placental growth. *Archives of Gynecology and Obstetrics* 1997;260:101–8.<br>19. Clapp JF 3rd, Kim H, Burciu B, Schmidt S, Petry K, Lopez B. Continuing regular exercise during pregnancy: effect of exercise volume on fetoplacental growth. *American Journal of Obstetrics and Gynecology* 2002;186(1):142–7.<br>20. Cordero Y, Mottola MF, Vargas J, Blanco M, Barakat R. Exercise is associated with a reduction in gestational diabetes mellitus. Medicine and Science in Sports and Exercise 2014;Oct 20 [Epub ahead of print].<br>21. De Oliveria Melo AS, Silva JLP, Tavares JS, Barros VO, Leite DFB, Amorim MMR. Effect of a physical exercise program during pregnancy on uteroplacental and fetal blood flow and fetal growth: A randomized controlled trial. *Obstetrics & Gynecology* 2012;120(2 Pt 1):302–10.<br>22. Melo A. Exercise and pregnancy: randomised clinical trial. Current Controlled Trials (www.controlled-trials.com/)[accessed 31 October 2010].<br>23. Di Carlo C, Iannotti G, Sparice S, Chiacchio MP, Greco E, Tommaselli GA, et al. The role of a personalized dietary intervention in managing gestational weight gain: a prospective, controlled study in a low-risk antenatal population. *Archives of Gynecology and Obstetrics* 2014;289:765–70.<br>24. Cramp CS, Moran LJ, Deussen AR, Yelland LN, Dodd JM. Evaluation of printed nutrition education material in overweight and obese women during pregnancy-findings from the limit randomised trial. *Journal of Paediatrics and Child Health* 2013;49 Suppl 2:118.<br>25. Dodd J. Limiting weight gain in overweight and obese women during pregnancy to improve health outcomes: a randomised trial. Australian Clinical Trials Registry (www.actr.org.au/) (accessed 31 October 2010).<br>26. Dodd J. Obesity in pregnancy-the limit randomised trial. *Journal of Paediatrics and Child Health* 2013;49 Suppl 2:4.<br>27. Dodd JM. Dietary and lifestyle advice for pregnant women who Are overweight or obese: the LIMIT randomized trial. *Annals of Nutrition & Metabolism* 2014;64(3-4):197–202.<br>28. Dodd JM, Cramp C, Sui Z, Yelland LN, Deussen AR, Grivell RM, et al. The effects of antenatal dietary and lifestyle advice for women who are overweight or obese onmaternal diet and physical activity: the LIMIT randomised trial. *BMC Medicine* 2014;12(1):161.<br>29. Dodd JM, McPhee AJ, Turnbull D, Yelland LN, Deussen<br>30. AR, Grivell RM, et al. The effects of antenatal dietary and lifestyle advice for women who are overweight or obese on neonatal health outcomes: the LIMIT randomised trial. *BMC Medicine* 2014;12(1):163.<br>31. Dodd JM, Turnbull D, McPhee AJ, Deussen AR, Grivell RM, Yelland LN, et al. Antenatal lifestyle advice for women who are overweight or obese: LIMIT randomised trial.*BMJ (Clinical Research Ed.)*2014;348:g1285.<br>32. Dodd JM, Turnbull DA, McPhee AJ, Wittert G, Crowther CA, Robinson JS. Limiting weight gain in overweight and obese women during pregnancy to improve health outcomes: the LIMIT randomised controlled trial. *BMC Pregnancy and Childbirth* 2011;11:79.<br>33. Eames AJ, Grivell RM, Dodd JM, Deussen A. The effect of limited gestational weight gain in overweight and obese women on maternal and infant outcomes. *Journal of Paediatrics and Child Health* 2013;49 Suppl 2:19.<br>34. Grivell R, Yelland L, Earl RA, Staehr CJ, Dodd J. The effect of antenatal dietary and lifestyle advice on fetal body composition in women who are overweight or obese: findings from the LIMIT randomised trial. *Ultrasound in Obstetrics and Gynecology* 2013;42(Suppl 1):10.<br>35. Grivell R, Yelland L, Staehr CJ, Earl RA, Dodd J. The effect of antenatal dietary and lifestyle advice on fetal growth in women who are overweight or obese: findings from the LIMIT randomised trial. *Ultrasound in Obstetrics and Gynecology* 2013;42(Suppl 1):83.<br>*36.* Kannieappan LM, Deussen AR, Moran LJ, Grivell RM, Yelland LN, Dodd JM. The effect of antenatal dietary advice on maternal body composition in women who are overweight or obese - findings from the limit randomised trial. *Journal of Paediatrics and Child Health*<br>37. 2013;49 Suppl2:94.<br>38. Newman AK, Deussen AR, Moran LJ, Grivell RM, Yelland LN, Turnbull D, et al. The effect of antenatal dietary and lifestyle advice on maternal psychological health in women who are overweight or obese-findings from the limit randomised trial. *Journal of Paediatrics and Child Health* 2013;49 Suppl 2:119.<br>39. Sui Z, Yelland LN, Turnbull D, Dodd JM. Walking to limit gestational weight gain and keep fit during pregnancy - findings from the walk randomised trial. *Journal of Paediatrics and Child Health* 2013;49 Suppl 2:120.<br>40. Ehrlich SF, Hedderson MM, Feng J, Crites Y, Quesenberry CP, Ferrara A. Lifestyle intervention improves postpartum fasting glucose levels in women with gestational diabetes. *Diabetes* 2014; 63 (Suppl 1):A95.<br>41. Ferrara A. Diet, exercise and breastfeeding intervention program for women with gestational diabetes (DEBI Trial). ClinicalTrials.gov(www.clinicaltrials.gov) [accessed 31 October 2010].<br>42. Ferrara A, Hedderson MM, Albright CL, Ehrlich SF, Quesenberry CP, Peng T, et al. A pregnancy and postpartum lifestyle intervention in women with gestational diabetes mellitus reduces diabetes risk factors: a feasibility randomized control trial. *Diabetes Care* 2011;34(7):1519–25.<br>43. Guelinckx I, Devlieger R, Mullie P, Vansant G. Effect of lifestyle intervention on dietary habits, physical activity, and gestational weight gain in obese pregnant women: a randomized controlled trial. *American Journal of Clinical Nutrition* 2010;91(2):373–80.

Review	References of included behavioural interventions delivered in pregnancy
	44. Bo K, Haakstad LAH. Is pelvic floor muscle training effective when taught in a general fitness class in pregnancy? A randomised controlled trial. *Physiotherapy* 2011;97(3):190–5.<br>45. Haakstad L. Effect of regular exercise in prevention of excessive weight gain in pregnancy. ClinicalTrials.gov (www.clinicaltrials.gov) [accessed 31 October 2010].<br>46. Haakstad L, Bo K. Effect of supervised aerobic dance exercise in prevention of excessive weight gain in pregnancy: a single blind randomized controlled trial. *International Journal of Gynecology & Obstetrics* 2009;107(Suppl 2):S198.<br>47. Haakstad L, Bo K. Exercise during pregnancy-Does it impact offspring birth weight parameters?. *Journal of Science and Medicine in Sport* 2012;15(Suppl 1):S340.<br>*48.* Haakstad LA, Bo K. Exercise in pregnant women and birth weight: a randomized controlled trial. *BMC Pregnancy and Childbirth*<br>49. 2011;11:66.<br>50. Haakstad LAH, Bo K. Effect of regular exercise on prevention of excessive weight gain in pregnancy: A randomised controlled trial.<br>51. *European Journal of Contraception and Reproductive Health Care* 2011;16(2):116–25.<br>52. Harrison CL, Lombard CB, Gibson-Helm M, Deeks A, Teede HJ. Limiting excess weight gain in high-risk pregnancies: A randomized controlled trial. *Endocrine Reviews* 2011; 32(3 Meeting Abstracts):P1–466.<br>53. Harrison CL, Lombard CB, Strauss BJ, Teede HJ. Optimizing healthy gestational weight gain in women at high risk of gestational diabetes: a randomized controlled trial. *Obesity* 2013;21(5):904–9.<br>54. Harrison CL, Teede HJ, Lombard CB. How effective is self-weighing in the setting of a lifestyle intervention to reduce gestational weight gain and postpartum weight retention?. *Australian & New Zealand Journal of Obstetrics & Gynaecology* 2014;54:382–5.<br>55. Lombard C, Harrison C, Teede H. A randomized controlled trial investigating self-weighing and the prevention of excess weight gain in early pregnancy. *Endocrine Reviews* 2011;32(3 Meeting Abstracts):P2–768.<br>56. Teede HJ, Harrison CL, Gibson-Helm M, Lombard CB. Improving physical activity in high-risk pregnancies: A randomized controlled trial. *Endocrine Reviews* 2011;32(3Meeting Abstracts):P1–467.<br>57. Hawkins M, Hosker M, Marcus BH, Rosal MC, Braun B, Stanek EJ, et al. A pregnancy lifestyle intervention to prevent gestational diabetes risk factors in overweight Hispanic women: a feasibility randomized controlled trial. *Diabetic Medicine* 2014;32:108–15.<br>58. Huang TT, Yeh CY, Tsai YC. A diet and physical activity intervention for preventing weight retention among Taiwanese childbearing women: a randomised controlled trial. *Midwifery* 2011;27(2):257–64.<br>59. Hui AL, Ludwig SM, Gardiner P, Sevenhuysen G, Murray R, Morris M, et al. Community-based exercise and dietary intervention during pregnancy: a pilot study. *Canadian Journal of Diabetes* 2006; 30(2):169–75.<br>60. Hui A, Back L, Ludwig S, Gardiner P, Sevenhuysen G, Dean H, et al. Lifestyle intervention on diet and exercise reduced excessive gestational weight gain in pregnant women undera randomised controlled trial. *BJOG: an International Journal of Obstetrics & Gynaecology* 2012;119(1):70–7.<br>61. Hui AL, Ludwig S, Gardiner P, Sevenhuysen G, Dean HJ, Sellers E, et al. Effects of lifestyle intervention on dietary intake, physical activity level, and gestational weight gain in pregnant women with different pre-pregnancy Body Mass Index in a randomized control trial. *BMC Pregnancy and Childbirth* 2014;14(1):331.<br>62. Jackson RA, Stotland NE, Caughey AB, Gerbert B. Improving diet and exercise in pregnancy with Video Doctor counseling: a randomized trial. *Patient Education and Counseling* 2011;83(2):203–9.<br>63. Jeffries K, Shub A, Walker SP, Hiscock R, Permezel M. Reducing excessive weight gain in pregnancy: a randomised controlled trial.<br>64. *Medical Journal of Australia* 2009;191(8):429–33.<br>65. Shub A. Diet and exercise in pregnancy. Australian Clinical Trials Registry (www.actr.org.au/) [accessed 21 June 2007].<br>66. Kieffer EC, Welmerink DB, Sinco BR, Welch KB, Rees Clayton EM, Schumann CY, et al. Dietary outcomes in a Spanish-language randomized controlled diabetes prevention trial with pregnant Latinas. *American Journal of Public Health* 2014;104(3):526–33.<br>*67.* Kong KL, Campbell G, Foster C, Peterson D, Lanningham-Foster L. A pilot walking program promotes moderate-intensity physical activity during pregnancy. *Medicine & Science in Sports & Exercise* 2014;46(3):462–72.<br>68. Korpi-Hyovalti E, Schwab U, Laaksonen DE, Linjama H, Heinonen S, Niskanen L. Effect of intensive counselling on and increasing physical activity during pregnancy: A feasibility study. *Diabetes* 2012;61 Suppl 1:A344.<br>69. Umpierrez G. Lifestyle intervention to limit excessive weight gain during pregnancy in minority women. ClinicalTrials.gov (accessed 21 May 2013). NCT01084941 2010.<br>70. Moses RG, Barker M, Winter M, Petocz P, Brand-Miller JC. Can a low-glycemic index diet reduce the need for insulin in gestational diabetes mellitus? A randomized trial. *Diabetes Care* 2009;32(6):996–1000.<br>71. Brand-Miller J. A pregnancy intervention to reduce postprandial glucose excursions in the primary prevention of paediatric obesity. Current Controlled Trials (www.controlled-trials.com/) [accessed 31 October 2010].<br>72. Moses RG, Casey S, Cleary J, Milosavljevic M, Quinn E, Tapsell L, et al. Effect of low glycaemic index dietary advice in normal pregnancy: The PREGGIO study. *Obesity Research and Clinical Practice* 2013;7:e34–5.<br>73. Moses RG, Casey SA, Quinn EG, Cleary JM, Tapsell LC, Milosavljevic M, et al. Pregnancy and Glycemic Index Outcomes study: effects of low glycemic index compared with conventional dietary advice on selected pregnancy outcomes. *American Journal of Clinical Nutrition* 2014;99(3):517–23.<br>74. Mujsindi W, Habash D, Childs G. Impact of nutrition education on gestational weight gain in obese pregnant women. *American Journal of Obstetrics and Gynecology* 2014;210(1 Suppl):S188.<br>75. Murtezani A, Pacarada M, Ibraimi Z, Nevzati A, Abazi N. The impact of exercise during pregnancy on neonatal outcomes: a randomised controlled trial. *Journal of Sports Medicine and Physical Fitness* 2014;54(6):802–8.<br>76. Nascimento KLK, Surita SLN, Parpinelli FGS, Kasawara MAP. Type of delivery and neonatal outcome in overweight and obese pregnant women with excessive weight gain. *Journal of Maternal-Fetal and*

Review	References of included behavioural interventions delivered in pregnancy
	Neonatal Medicine 2012;25(S2):73–4.<br>77. Nascimento SL, Surita FG, Parpinelli MA, Siani S, Pintoe Silva JL. The effect of an antenatal physical exercise programme on maternal/perinatal outcomes and quality of life in overweight and obese pregnant women: a randomised clinical trial. *BJOG: an International Journal of Obstetrics & Gynaecology* 2011;118(12):1455–63.<br>78. Surita F. Physical exercise influence among overweightand obese pregnant women. ClinicalTrials.gov (http://clinicaltrials.gov/) [accessed 21 May 2013] 2010.<br>79. Oostdam N, Bosmans J, Wouters MGAJ, Eekhoff EMW, van Mechelen W, van Poppel MNM. Cost-effectiveness of an exercise program during pregnancy to prevent gestational diabetes: Results of an economic evaluation alongside a randomised controlled trial. *BMC Pregnancy and Childbirth* 2012;12:64.<br>80. Oostdam N, Van Poppel MN, Eekhoff EM, Wouters MG, Van Mechelen W. Design of FitFor2 study: the effects of an exercise program on insulin sensitivity and plasma glucose levels in pregnant women at high risk for gestational diabetes. *BMC Pregnancy and Childbirth* 2009;9:1.<br>81. Oostdam N, Van Poppel MNM, Wouters MGAJ, Eekhoff EMW, Bekedam DJ, Kuchenbecker WKH, et al. No effect of the FitFor2 exercise programme on blood glucose, insulin sensitivity, and birthweight in pregnant women who were overweight and at risk for gestational diabetes: Results of a randomised controlled trial. *BJOG: an International Journal of Obstetrics and Gynaecology* 2012;119(9):1098–107.<br>82. Van Poppel M, Oostdam N, Wouters M, Eekhoff M, Van Mechelen W. FitFor2: Effects of an exercise training program on the incidence of gestational diabetes. *Journal of Science and Medicine in Sport* 2012;15 Suppl 1:S342–S343.<br>83. Facchinetti F. Pregnancy complications in women with BMI > 25kg/m2 enrolled in a healthy lifestule and eating habits program. ClinicalTrials.gov (accessed 21 May 2013). NCT01783210 2013.<br>84. Petrella E, Facchinetti F, Bertarini V, Pignatti L, Neri I, Battistini NC. Occurrence of pregnancy complications in women with BMI > 25 submitted to a healthy lifestyle and eating habits program. *American Journal of Obstetrics and Gynecology* 2013;208(1 Suppl):S33–4.<br>85. Petrella E, Malavolti M, Bertarini V, Pignatti L, Neri I, Battistini NC, et al. Gestational weight gain in overweight and obese women enrolled in a healthy lifestyle and eating habits program. *Journal of Maternal-Fetal and Neonatal Medicine* 2014;27(13):1348–52.<br>86. Petrov Fieril K, Glantz A, Fagevik Olsen M. The efficacy of moderate-to-vigorous resistance exercise during pregnancy: A randomized controlled trial. Acta Obstetricia et Gynecologica Scandinavica 2014 Oct 7 [Epub ahead of print].<br>87. Phelan S, Phipps MG, Abrams B, Darroch F, Grantham K, Schaffner A, et al. Does behavioral intervention in pregnancy reduce postpartum weight retention? Twelve-month outcomes of the Fit for Delivery randomized trial. *American Journal of Clinical Nutrition* 2014;99(2):302–11.<br>88. Phelan S, Phipps MG, Abrams B, Darroch F, Schaffner A, Wing RR. Factors associated with success in the “fit for delivery” intervention to reduce excessive gestational weight gain. *Obesity* 2011;19(Suppl 1):S95.<br>89. Phelan S, Phipps MG, Abrams B, Darroch F, Schaffner A, Wing RR. Randomized trial of a behavioral intervention to prevent excessive gestational weight gain: the Fit for Delivery Study. *American Journal of Clinical Nutrition* 2011;93(4):772–9.<br>90. Pinzon DC, Zamora K, Martinez JH, Florez-Lopez ME, de Plata AC, Mosquera M, et al. Type of delivery and<br>91. Ramirez-Velez R. Aerobic exercise in pregnant Latina women: Effect on metabolic and body composition outcomes. A randomized clinical trial. *FASEB Journal* 2014;28(1 Suppl 1):886.6.<br>92. Ramirez-Velez R. Combined aerobic and resistance exercise on metabolic and body composition outcomes in primigravid Latina women. *Obesity Reviews* 2014;15(Suppl2):201–2.<br>93. Ramirez-Velez R, Aguilar de Plata AC, Escudero MM, Echeverry I, Ortega JG, Salazar B, et al. Influence of regular aerobic exercise on endothelium-dependent vasodilation and cardiorespiratory fitness in pregnant women. *Journal of Obstetrics and Gynaecology Research* 2011;37(11):1601–8.<br>94. Pollak KI, Alexander SC, Bennett G, Lyna P, Coffman CJ, Bilheimer A, et al. Weight-related SMS texts promoting appropriate pregnancy weight gain: A pilot study. *Patient Education and Counselling* 2014;97:256–60.<br>95. Polley BA, Wing RR, Sims CJ. Randomized controlled trial to prevent excessive weight gain in pregnant women. *International Journal of Obesity & Related Metabolic Disorders: Journal of the International Association for the Study of Obesity* 2002;26(11):1494–502.<br>96. Briley AL, Barr S, Badger S, Bell R, Croker H, Godfrey KM, et al. A complex intervention to improve pregnancy outcome in obese women; the UPBEAT randomised controlled trial. *BMC Pregnancy and Childbirth* 2014;14(1):74.<br>97. Hayes L, Bell R, Robson S, Poston L. Association between physical activity in obese pregnant women and pregnancy outcomes: The UPBEAT Pilot Study. *Annals of Nutrition & Metabolism* 2014;64(3-4):239–46.<br>98. Maitland RA, Barr S, Briley A, Seed P, Poston L. Incidence of gestational diabetes in an obese population using the International Association of Diabetes and Pregnancy Study Groups (IADPSG) criteria in the UK Pregnancies Better Eating and Activity Trial (UPBEAT) pilot study. *Diabetic Medicine* 2012;29(Suppl 1):152.<br>99. Poston L. Improving pregnancy outcome in obese women: a feasibility study. Current Controlled Trials (http://controlled-trials.com/) (accessed 31 October 2010).<br>100. Poston L, Briley AL, Barr S, Bell R, Croker H, Coxon K, et al. Developing a complex intervention for diet andactivity behaviour change in obese pregnant women (the UPBEAT trial); assessment of behavioural change and process evaluation in a pilot randomised controlled trial. *BMC Pregnancy and Childbirth* 2013;13(1):148.<br>101. Schneeberger C, Flynn A, Barr S, Seed PT, Inskip HM, Poston L. Maternal diet patterns and glycaemic load in obese pregnant women taking part in a pilot trial of a lifestyle intervention (the upbeat trial). Diabetes 2014; Vol.63.<br>102. Price B, Amini B, Kappeler K. Exercise in pregnancy: Effect on fitness and obstetric outcomes - a randomized trial. *Medicine & Science in Sports & Exercise* 2012;44(12):2263–9.<br>103. Quinlivan JA, Lam LT, Fisher J. A randomised trial of a four-step multidisciplinary approach to the antenatal care of obese pregnant women. *Australian and New Zealand Journal of Obstetrics and Gynaecology* 2011;51:141–6.<br>104. Rae A, Bond D, Evans S, North F, Roberman B, Walters B. A randomised controlled trial of dietary energy restriction in the management of obese women with gestational diabetes. *Australian & New*

Review	References of included behavioural interventions delivered in pregnancy
	Zealand Journal of Obstetrics & Gynaecology 2000;40 (4):416–22.<br>105. Rauh K, Gabriel E, Kerschbaum E, Schuster T, von Kries R, Amann-Gassner U, et al. Safety and efficacy of a lifestyle intervention for pregnant women to prevent excessive maternal weight gain: a cluster-randomized controlled trial. *BMC Pregnancy and Childbirth* 2013;13(1):151.<br>106. Renault KM, Norgaard K, Nilas L, Carlsen EM, Cortes D, Pryds O, et al. The Treatment of Obese Pregnant Women (TOP) study: A randomized controlled trial of the effect of physical activity intervention assessed by pedometer with or without dietary intervention in obese pregnant women. *American Journal of Obstetrics and Gynecology* 2014;210(2):134.e1–9.<br>107. Pawlak DB. Glycemic load and infant birth weight in pregnant overweight/obese women. ClinicalTrials.gov (http://clinicaltrials.gov/) (accessed 31 October 2010).<br>108. Rhodes ET, Pawlak DB, Takoudes TC, Ebbeling CB, Feldman HA, Lovesky MM, et al. Effects of a low-glycemic load diet in overweight and obese pregnant women: a pilot randomized controlled trial. *American Journal of Clinical Nutrition* 2010;92(6):1306–15.<br>109. Donnelly J, Horan M, Walsh J, McGowan C, Byrne J, Molloy EJ, et al. Impact of a low GI diet on neonatal body composition (ROLO Kids). Pediatric Academic Societies Annual Meeting; 2013 May 4-7; Washington DC, USA. 2013.<br>110. Donnelly JM, Walsh JM, Byrne J, Molloy E, McAuliffe FM. Altered neonatal anthropometric measurements following maternal low GI diet in pregnancy (ROLO study). *Acta Obstetricia et Gynecologica Scandinavica* 2013;92(s160):13.<br>111. Horan M, McGowan C, Donnelly J, Gibney E, McAuliffe F. Maternal diet and weight at 3 months partum following a pregnancy intervention with a low glycaemic index diet:Results from the ROLO randomised control trial. *Archives of Disease in Childhood. Fetal and Neonatal Edition* 2014;99 (Suppl 1):A129-A130, Abstract no: PMM.20.<br>112. Horan MK, McGowan CA, Donnelly J, Gibney E, McAuliffe FM. The association of maternal characteristics and macronutrient intake in pregnancy with neonatal body composition. *Archives of Disease in Childhood. Fetal and Neonatal Edition* 2014;99(Suppl 1):A11.<br>113. Horan MK, McGowan CA, Doyle O, McAuliffe FM. Well-being in pregnancy: An examination of the effect of socioeconomic, dietary and lifestyle factors including impact of a low glycaemic index dietary intervention. *European Journal of Clinical Nutrition* 2014;68(1):19–24.<br>114. Horan MK, McGowan CA, Gibney ER, Donnelly JM, McAuliffe FM. Maternal diet and weight at 3 months postpartum following a pregnancy intervention with a low glycaemic index diet: results from the ROLO randomised control trial. *Nutrients* 2014;6(7):2946–55.<br>115. Horan MK, McGowan CA, Gibney ER, Donnelly JM, McAuliffe FM. Maternal low glycaemic index diet, fat intake and postprandial glucose influences neonatal adiposity - secondary analysis from the ROLO study. *Nutrition Journal* 2014;13(1):78.<br>116. Mahony R, Byrne J, Curran S, O'Herlihy C, McAuliffe F. A pilot study of the feasibility of a randomised trial of low glycaemic diet versus normal diet from early pregnancy in euglycaemic women. *Archives of Disease in Childhood. Fetal and Neonatal Edition* 2008;93(Suppl 1):Fa38.<br>117. McAuliffe F. A randomised controlled trial of low glycaemic index carbohydrate diet versus no dietary intervention in the prevention of recurrence of foetal macrosomia. CurrentControlled Trials (www.controlled-trials.com/) (accessed 12.05.2010).<br>118. McGowan CA, Walsh JM, Byrne J, Curran S, McAuliffe FM. The influence of a low glycemic index dietary intervention on maternal dietary intake, glycemic index and gestational weight gain during pregnancy: a randomized controlled trial. *Nutrition Journal* 2013;12(1):140.<br>119. Walsh J, Mahony R, Foley M, Mc Auliffe F. A randomised control trial of low glycaemic index carbohydrate diet versus no dietary intervention in the prevention of recurrence of macrosomia. *BMC Pregnancy and Childbirth* 2010;10:16.<br>120. Walsh J, Mahony R, Foley M, McAuliffe F. ROLO study: a randomized control trial of low glycemic index diet to prevent macrosomia in euglycemic women. *American Journal of Obstetrics and Gynecology* 2012;206,Suppl 1):S4.<br>121. Walsh J, McGowan C, Byrne J, Foley M, Mahony R, McAuliffe F. The influence of a low glycaemic index dietary intervention on maternal glycaemic index, dietary intake and gestational weight gain. *American Journal of Obstetrics and Gynecology* 2013;208(1 Suppl):S33.<br>122. Walsh JM, Mahony RM, Canty G, Foley ME, McAuliffe FM. Identification of those most likely to benefit from a low-glycaemic index dietary intervention in pregnancy. *British Journal of Nutrition* 2014;112:583–9.<br>123. Walsh JM, McGowan CA, Mahony R, Foley ME, McAuliffe FM. Low glycaemic index diet in pregnancy to prevent macrosomia (ROLO study): randomised control trial. *BMJ* 2012;345:e5605.<br>124. Nilsson K. Weight gain during pregnancy - a randomized controlled trial of intervention to prevent excessive gestational weight gain. ClinicalTrials.gov (http://clinicaltrials.gov/) [accessed 5 September 2014] 2009.<br>125. Ronnberg A, Ostlund I, Fadl H, Gottvall T, Nilsson K. Intervention during pregnancy to reduce excessive gestational weight gain-a randomised controlled trial. BJOG:an International Journal of Obstetrics and Gynaecology 2014 Nov 4 [Epub ahead of print].<br>126. Ruchat SM, Davenport MH, Giroux I, Hillier M, Batada A, Sopper MM, et al. Nutrition and exercise reduce excessive weight gain in normal-weight pregnant women. *Medicine and Science in Sports and Exercise* 2012;44(8):1419–26.<br>127. Ruiz R, Perales M, Pelaez M, Lopez C, Lucia A, Barakat R. Supervised exercise-based intervention to prevent excessive gestational weight gain: a randomized controlled trial. *Mayo Clinic Proceedings* 2013;88(12):1388–97.<br>128. Santaella MP. The role of a supervised physical exercise program as an alternative on the control of maternal estational weight gain. ClinicalTrials.gov (accessed 21 May20 13). NCT01790347 2013.<br>129. Santos IA, Stein R, Fuchs SC, Duncan BB, Ribeiro JP, Kroeff LR, et al. Aerobic exercise and submaximal functional capacity in overweight pregnant women: a randomized trial. *Obstetrics & Gynecology* 2005;106(2):243–9.

Review	References of included behavioural interventions delivered in pregnancy
	130. Morkved S. Effects of regular exercise during pregnancy.ClinicalTrials.gov(www.clinicaltrials.gov) (accessed 31 October 2010).<br>*131.* Stafne SN, Salvesen KÅ, Romundstad PR, Eggebø TM, Carlsen SM, Mørkved S. Regular exercise during pregnancy to prevent gestational diabetes: a randomized controlled trial. *Obstetrics & Gynecology* 2012;119(1):29–36.<br>132. Szmeja MA, Grivell RM, Deussen AR, Dodd JM. Evaluation of information provision to women who are overweight or obese during pregnancy. *Journal of Paediatrics and Child Health* 2011;47(Suppl 1):78.<br>133. Thornton YS, Smarkola C, Kopacz SM, Ishoof SB. Perinatal outcomes in nutritionally monitored obese pregnant women: a randomized clinical trial. *Journal of the National Medical Association* 2009;101:569–77.<br>134. Vesco K, Leo M, Gillman M, King J, McEvoy C, Karanjaa N, et al. Impact of a weight management intervention on pregnancy outcomes among obese women: The Healthy Moms Trial. *American Journal of Obstetrics and Gynecology* 2013;208(1 Suppl 1):S352.<br>135. Vesco KK, Karanja N, King JC, Gillman MW, Leo C, Perrin N, et al. Efficacy of a group-based dietary intervention for limiting gestational weight gain among obese women: A randomized trial. *Obesity (Silver Spring, Md.)*2014;2(9):1989–96.<br>136. Vesco KK, Karanja N, King JC, Gillman MW, Perrin N, McEvoy C, et al. Healthy Moms, a randomized trial to promote and evaluate weight maintenance among obese pregnant women: study design and rationale. *Contemporary Clinical Trials* 2012;33(4):777–85.<br>137. Tanvig M. Lifestyle in pregnancy and offspring (LiPO). ClinicalTrials.gov(http://clinicaltrials.gov/) [accessed 6 February 2014] 2013.<br>138. Tanvig M. Lifestyle in pregnancy and offspring - comparison between children born to obese women and children born to normal weight women (LiPO). ClinicalTrials.gov (http:// clinicaltrials.gov/) [accessed 6 February 2014] 2013.<br>139. Tanvig M. Offspring body size and metabolic profile - Effects of lifestyle intervention in obese pregnant women. *Danish Medical Journal* 2014;61(7):B4893.<br>140. Tanvig M, Vinter CA, Jorgensen JS, Wehberg S, Ovesen PG, Beck-Nielsen H, et al. Effects of lifestyle intervention in pregnancy and anthropometrics at birth on offspring metabolic profile at 2.8 years - results from the Lifestyle in Pregnancy and Offspring (LiPO) study. *Journal of Clinical Endocrinology and Metabolism* 2015;100(1):175–83.<br>141. Vinter A. Lifestyle and pregnancy: the clinical effect of lifestyle intervention during pregnancy in obese women. ClinicalTrials.gov(www.clinicaltrials.gov) [accessed 31 October 2010].<br>142. Vinter C, Jensen D, Ovesen P, Beck-Nielsen H, Lamont R, Jorgensen J. Postpartum weight retention and breastfeeding among obese women from the LiP (Lifestyle in Pregnancy) Study. *Acta Obstetricia et Gynecologica Scandinavica* 2012;91(Suppl 159):141–2.<br>143. Vinter CA, Jensen DM, Ovesen P, Beck-Nielsen H, Jorgensen JS. The LiP (Lifestyle in Pregnancy) study: a randomized controlled trial of lifestyle intervention in 360 obese pregnant women. *Diabetes Care* 2011;34(12):2502–7.<br>144. Vinter CA, Jensen DM, Ovesen P, Beck-Nielsen H, Tanvig M, Lamont RF, et al. Postpartum weight retention and breastfeeding among obese women from the randomized controlled Lifestyle in Pregnancy (LiP) trial. *Acta Obstetricia et Gynecologica Scandinavica* 2014;93:794–801.<br>145. Vitolo MR. Impact of a nutritional intervention program for weight control during pregnancy. ClinicalTrials.gov (www.clinicaltrials.gov) [accessed 31 October 2010].<br>146. Vitolo MR, Fraga Bueno MS, Mendes Gama C. Impact of a dietary counseling program on the gain weight speed of pregnant women attended in a primary care service. *Revista Brasileira de Ginecologia e Obstetricia* 2011;33(1):13–9.<br>147. Wilkinson SA, McIntyre HD. Evaluation of the ’healthy start to pregnancy’ early antenatal health promotion workshop: a randomized controlled trial. *BMC Pregnancy and Childbirth* 2012;12:131.<br>148. Wolff S, Legarth J, Vangsgaard K, Toubro S, Astrup A. A randomized trial of the effects of dietary counseling on gestational weight gain and glucose metabolism in obese pregnant women. *International Journal of Obesity* 2008;32(3):495–501<br>**Note: There are more references than studies included as several papers are reported for some studies**
O’Brien *et al.* 2015 [16]	1. Asbee SM, Jenkins TR, Butler JR, White J, Elliot M, Rutledge A. Preventing excessive weight gain during pregnancy through dietary and lifestyle counselling: a randomized controlled trial. Obstet Gynecol. 2009;113:305–12.<br>2. Phelan S, Phipps MG, Abrams B, Darroch F, Schaffner A, Wing RR. Randomized trial of a behavioral intervention to prevent excessive gestational weight gain: the Fit for Delivery Study. Am J Clin Nutr. 2011;93:772–9. ª 2015 Nordic Federation of Societies of Obstetrics and Gynecology, Acta Obstetricia et Gynecologica Scandinavica 95 (2016) 259–269 267 C.M. O’Brien et al. Antenatal dietary and lifestyle interventions<br>3. Polley BA, Wing RR, Sims CJ. Randomized controlled trial to prevent excessive weight gain in pregnant women. Int J Obes Relat Metab Disord. 2002;26:1494–502.<br>4. Hui A. Community based exercise and dietary intervention during pregnancy: a pilot study. Can J Diabetes. 2006;30:169–75.<br>5. Hui AL, Ludwig S, Gardiner P, Sevenhuysen G, Dean HJ, Sellers E, et al. Effects of lifestyle intervention on dietary intake, physical activity level, and gestational weight gain in pregnant women with different pre-pregnancy Body Mass Index in a randomized control trial. BMC Pregnancy Childbirth. 2014;14:331.<br>6. Hui A, Back L, Ludwig S, Gardiner P, Sevenhuysen G, Dean H, et al. Lifestyle intervention on diet and exercise reduced excessive gestational weight gain in pregnant women under a randomised controlled trial. BJOG. 2012;119:70–7.<br>7. Huang TT, Yeh CY, Tsai YC. A diet and physical activity intervention for preventing weight retention among Taiwanese childbearing women: a randomised controlled trial. Midwifery. 2011;27:257–64.<br>8. Jackson RA, Stotland NE, Caughey AB, Gerbert B. Improving diet and exercise in pregnancy with Video Doctor counseling: a randomized trial. Patient Educ Couns. 2011;83:203–9.<br>9. Laitinen K, Poussa T, Isolauri E, Nutrition AMI. Intestinal microbiota G. Probiotics and dietary counselling contribute to glucose regulation during and after pregnancy: a randomised controlled trial. Br J Nutr. 2009;101:1679–87.<br>10. Althuizen E, van der Wijden CL, van Mechelen W, Seidell JC, van Poppel MN. The effect of a counselling intervention on weight changes during and after pregnancy: a randomised trial. BJOG. 2013;120:92–9.<br>11. Jefferies K, Shub A, Walker SP, Hiscock R, Permezel M. Reducing excessive weight gain in pregnancy: a randomised controlled trial. Med J Aust. 2009;191:429–33.

Review	References of included behavioural interventions delivered in pregnancy
Oostdam *et al.* 2011 [17]	1. Barakat R, Lucia A, Ruiz JR. Resistance exercise training during pregnancy and newborn's birth size: A randomised controlled trial. Int J Obes 2009;33:1048–1057.<br>2. Begum MR, Khanam NN, Quadir E, et al. Prevention of gestational diabetes mellitus by continuing metformin therapy throughout pregnancy in women with polycystic ovary syndrome. J Obstet Gynaecol Res 2009;35:282–286.<br>3. Bevier WC, Fischer R, Jovanovic L. Treatment of women with an abnormal glucose challenge test (but a normal oral glucose tolerance test) decreases the prevalence of macrosomia. Am J Perinatol 1999;16:269–275.<br>4. Clapp IJF. Diet, exercise, and fetal-placental growth. Arch Gynecol Obstet 1997;260:101–108.<br>5. Fraser RB, Ford FA, Milner RD. A controlled trial of a high dietary fibre intake in pregnancy—Effects on plasma glucose and insulin levels. Diabetologia 1983;25:238–241.<br>6. Guelinckx I, Devlieger R, Mullie P, Vansant G. Effect of lifestyle intervention on dietary habits, physical activity, and gestational weight gain in obese pregnant women: A randomized controlled trial. Am J Clin Nutr 2010;91:373–380.<br>7. Hopkins SA, Baldi JC, Cutfield WS, McCowan L, Hofman PL. Exercise training in pregnancy reduces offspring size without changes in maternal insulin sensitivity. J Clin Endocrinol Metab 2010;95:2080–2088.<br>8. Hui AL, Ludwig SM, Gardiner P, et al. Community-based exercise and dietary intervention during pregnancy: A pilot study. Can J Diabetes 2006;30:169–175.<br>9. Laitinen K, Poussa T, Isolauri E. Probiotics and dietary counselling contribute to glucose regulation during and after pregnancy: A randomised controlled trial. Br J Nutr 2009; 101:1679–1687.<br>10. Ong MJ, Guelfi KJ, Hunter T, Wallman KE, Fournier PA, Newnham JP. Supervised home-based exercise may attenuate the decline of glucose tolerance in obese pregnant women. Diabetes Metab 2009;35:418–421.<br>11. Vanky E, Salvesen KA, Heimstad R, Fougner KJ, Romundstad P, Carlsen SM. Metformin reduces pregnancy complications without affecting androgen levels in pregnant polycystic ovary syndrome women: Results of a randomized study. Hum Reprod 2004;19:1734–1740.<br>12. Wolff S, Legarth J, Vangsgaard K, Toubro S, Astrup A. A randomized trial of the effects of dietary counseling on gestational weight gain and glucose metabolism in obese pregnant women. Int J Obes (Lond) 2008;32:495–501.<br>13. Asbee SM, Jenkins TR, Butler JR, White J, Elliot M, Rutledge A. Preventing excessive weight gain during pregnancy through dietary and lifestyle counseling: A randomized controlled trial. Obstet Gynecol 2009;113:305–312<br>14. Polley BA, Wing RR, Sims CJ. Randomized controlled trial to prevent excessive weight gain in pregnant women. Int J Obes Relat Metab Disord 2002;26:1494–1502.<br>15. Vanky E, Stridsklev S, Heimstad R, et al. Metformin versus placebo from first trimester to delivery in polycystic ovary syndrome: A randomized, controlled multicenter study. J Clin Endocrinol Metab 2010;95:E448–E455.<br>16. Jeffries K, Shub A, Walker SP, Hiscock R, Permezel M. Reducing excessive weight gain in pregnancy: A randomised controlled trial. Med J Aust 2009;191:429–433.<br>17. Rhodes ET, Pawlak DB, Takoudes TC, et al. Effects of a lowglycemic load diet in overweight and obese pregnant women: A pilot randomized controlled trial. Am J Clin Nutr 2010;92:1306–1315.<br>18. Thornton YS, Smarkola C, Kopacz SM, Ishoof SB. Perinatal outcomes in nutritionally monitored obese pregnant women: A randomized clinical trial. J Natl Med Assoc 2009;101: 569–577.<br>19. Moses RG, Luebcke M, Davis WS, et al. Effect of a lowglycemic-index diet during pregnancy on obstetric outcomes. Am J Clin Nutr 2006;84:807–812.
Oteng-Ntim *et al.* 2012 [18]	1. Guelinckx I, Devlieger R, Mullie P, Vansant G: Effect of lifestyle intervention on dietary habits, physical activity, and gestational weight gain in obese pregnant women: a randomized controlled trial. Am J Clin Nutr 2010, 91:373-380.<br>2. Asbee SM, Jenkins TR, Butler JR, White J, Elliot M, Rutledge A: Preventing excessive weight gain during pregnancy through dietary and lifestyle counseling: a randomized controlled trial. Obstet Gynecol 2009, 113:305-312.<br>3. Jeffries K, Shub A, Walker SP, Hiscock R, Permezel M: Reducing excessive weight gain in pregnancy: a randomised controlled trial. Med J Aust 2009, 191:429-433.<br>4. Thornton YS, Smarkola C, Kopacz SM, Ishoof SB: Perinatal outcomes in nutritionally monitored obese pregnant women: a randomized clinical trial. J Natl Med Assoc 2009, 101:569-577.<br>5. Wolff S, Legarth J, Vangsgaard K, Toubro S, Astrup A: A randomized trial of the effects of dietary counseling on gestational weight gain and glucose metabolism in obese pregnant women. Int J Obes (Lond) 2008, 32:495-501.<br>6. Yeo S: A randomized comparative trial of the efficacy and safety of exercise during pregnancy: design and methods. Contemp Clin Trials 2006, 27:531-540.<br>7. Polley BA, Wing RR, Sims CJ: Randomized controlled trial to prevent excessive weight gain in pregnant women. Int J Obes Relat Metab Disord 2002, 26:1494-1502.<br>8. Hui AL, Ludwig SM, Gardiner P, Sevenhuysen G, Murray R, Morris M, Shen GX: Community based exercise and dietary intervention during pregnancy: a pilot study. Canadian Journal of Diabetes 2006, 30:169-175.<br>9. Phelan S, Phipps MG, Abrams B, Darroch F, Schaffner A, Wing RR: Randomized trial of a behavioral intervention to prevent excessive gestational weight gain: the Fit for Delivery Study. Am J Clin Nutr 2011, 93:772-779.<br>10. Mottola MF, Giroux I, Gratton R, Hammond JA, Hanley A, Harris S, McManus R, Davenport MH, Sopper MM: Nutrition and exercise prevent excess weight gain in overweight pregnant women. Med Sci Sports Exerc 2010, 42:265-272.<br>11. Claesson IM, Sydsjo G, Brynhildsen J, Cedergren M, Jeppsson A, Nystrom F, Sydsjö A, Josefsson A: Weight gain restriction for obese pregnant women: a case-control intervention study. BJOG 2008, 115:44-50.<br>12. Gray-Donald K, Robinson E, Collier A, David K, Renaud L, Rodrigues S: Intervening to reduce weight gain in pregnancy and gestational diabetes mellitus in Cree communities: an evaluation. CMAJ 2000, 163:1247-1251.<br>13. Shirazian T, Monteith S, Friedman F, Rebarber A: Lifestyle modification program decreases pregnancy weight gain in obese women. Am J Perinatol 2010, 27:411-414.<br>14. Olson CM: A call for intervention in pregnancy to prevent maternal and child obesity. Am J Prev Med 2007, 33:435-436.<br>15. Kinnunen TI, Pasanen M, Aittasalo M, Fogelholm M, Hilakivi-Clarke L, Weiderpass E, Luoto R: Preventing excessive weight gain during pregnancy - a controlled trial in primary health care. Eur J Clin Nutr 2007, 61:884-891.

Review	References of included behavioural interventions delivered in pregnancy
Quinlivan *et al.* 2011 [19]	1. Wolff S, Legarth J, Vangsaard K, Toubro S, Astrup A. A randomized trial of effects of dietary counseling on gestational weight gain and glucose metabolism in obese pregnant women. Int J Obes (Lond) 2008;32:495–501<br>2. Thornton YS, Smarkola C, Kopacz SM, Ishoof SB. Perinatal outcomes in nutritionally monitored obese pregnant women: a randomized clinical trial. J Natl Med Assoc 2009;101:569 –77.<br>3. Guelinckx I, Devlieger R, Mullie P, Vansant G. Effect of lifestyle intervention on dietary habits, physical activity, and gestational weight gain in obese pregnant women: a randomized controlled trial. Am J Clin Nutr 2010;91:373– 80.<br>4. Quinlivan JA, Lam LT, Fisher J. A randomized trial of a four-step multidisciplinary approach to the antenatal care of obese pregnant women. AustNZJ Obstet Gynaecol 2011;51:141– 6.
Rogozińska *et al.* 2015 [20]	1. Luoto R, Laitinen K, Nermes M, Isolauri E, Luoto R, et al. (2010) Impact of maternal probiotic-supplemented dietary counselling on pregnancy outcome and prenatal and postnatal growth: a double-blind,placebo-controlled study. Br J Nutr 103(12):1792–9. doi: 10.1017/S0007114509993898 PMID: 20128938.<br>2. D'Anna R, Scilipoti A, Giordano D, Caruso C, Cannata ML, et al. (2013) Myo-inositol supplementation and onset of gestational diabetes mellitus in pregnant women with a family history of type 2 diabetes: A prospective, randomized, placebo-controlled study. Diabetes Care 36(4):854–7. doi: 10.2337/dc12-1371 PMID: 23340885.<br>3. Korpi-Hyovalti E, Schwab U, Laaksonen DE, Linjama H, Heinonen S, et al. (2012) Effect of intensive counselling on the quality of dietary fats in pregnant women at high risk of gestational diabetes mellitus. Br J Nutr 108(5):910–7. doi: 10.1017/S0007114511006118 PMID: 22093485.<br>4. Quinlivan J, Lam LT, Fisher J (2011) A randomised trial of a four-step multidisciplinary approach to the antenatal care of obese pregnant women. Australian & New Zealand Journal of Obstetrics & Gynaecology 51(2):141–6.<br>5. Thornton YS, Smarkola C, Kopacz SM, Ishoof SB (2009) Perinatal outcomes in nutritionally monitored obese pregnant women: a randomized clinical trial. J Natl Med Assoc 101(6):569–77. PMID:19585925.<br>6. Wolff S, Legarth J, Vangsgaard K, Toubro S, Astrup A (2008) A randomized trial of the effects of dietary counseling on gestational weight gain and glucose metabolism in obese pregnant women. Int J Obes (Lond) 32(3):495–501. doi: 10.1038/sj.ijo.0803710 PMID: 18227847.<br>7. Bogaerts A, Devlieger R, Nuyts E, Witters I, Gyselaers W, et al. (2013) Effects of lifestyle intervention in obese pregnant women on gestational weight gain and mental health: A randomized controlled trial. Int J Obes 37(6):814–21. doi: 10.1038/ijo.2012.162 PMID: 23032404.<br>8. Dodd JM, Turnbull D, McPhee AJ, Deussen AR, Grivell RM, et al. (2014) Antenatal lifestyle advice for women who are overweight or obese: LIMIT randomised trial. BMJ 348:doi: http://dx.doi.org/10.1136/bmj.g1285.<br>9. Guelinckx I, Devlieger R, Mullie P, Vansant G (2010) Effect of lifestyle intervention on dietary habits, physical activity, and gestational weight gain in obese pregnant women: A randomized controlled trial. Am J Clin Nutr 91(2):373–80. doi: 10.3945/ajcn.2009.28166 PMID: 19955397.<br>10. Harrison CL, Lombard CB, Gibson-Helm M, Deeks A, Teede HJ (2011) Limiting excess weight gain in high-risk pregnancies: A randomized controlled trial. Endocr Rev 32(3). doi: 10.1210/er.2011-0001 PMID: 21441345.<br>11. Hui A, Back L, Ludwig S, Gardiner P, Sevenhuysen G, et al. (2011) Lifestyle intervention on diet and exercise reduced excessive gestational weight gain in pregnant women under a randomised controlled trial. BJOG Int J Obstet Gynaecol 119(1):70–7.<br>12. Jeffries K, Shub A, Walker SP, Hiscock R, Permezel M (2009) Reducing excessive weight gain in pregnancy: a randomised controlled trial. Med J Aust 191(8):429–33. PMID: 19835535.<br>13. Petrella E, Malavolti M, Bertarini V, Pignatti L, Neri I, et al. (2013) Gestational weight gain in overweight and obese women enrolled in a healthy lifestyle and eating habits program. J Matern Fetal Neonatal Med Nov 25.<br>14. Phelan S, Phipps MG, Abrams B, Darroch F, Schaffner A, et al. (2011) Randomized trial of a behavioural intervention to prevent excessive gestational weight gain: the Fit for Delivery Study. Am J Clin Nutr 93 (4):772–9. doi: 10.3945/ajcn.110.005306 PMID: 21310836.<br>15. Polley BA, Wing RR, Sims CJ (2002) Randomized controlled trial to prevent excessive weight gain in pregnant women. Int J Obes Relat Metab Disord 26(11):1494–502. PMID: 12439652.<br>16. Poston L, Briley AL, Barr S, Bell R, Croker H, et al. (2013) Developing a complex intervention for diet and activity behaviour change in obese pregnant women (the UPBEAT trial); Assessment of behavioural change and process evaluation in a pilot randomised controlled trial. BMC Pregnancy Childbirth 13.<br>17. Renault KM, Norgaard K, Nilas L, Carlsen EM, Cortes D, et al. (2014) The Treatment of Obese Pregnant Women (TOP) study: a randomized controlled trial of the effect of physical activity intervention assessed by pedometer with or without dietary intervention in obese pregnant women. Am J Obstet Gynecol 210(2):134–9. doi: 10.1016/j.ajog.2013.09.029 PMID: 24060449.<br>18. Vesco K, Leo M, Gillman M, King J, McEvoy C, et al. (2013) Impact of a weight management intervention on pregnancy outcomes among obese women: The Healthy Moms Trial. Am J Obstet Gynecol 208(1):S352.<br>19. Vinter C, Jensen DM, Ovesen P, Beck-Nielsen H, Jorgensen JS (2011) The LiP (Lifestyle in Pregnancy) study: a randomized controlled trial of lifestyle intervention in 360 obese pregnant women. Diabetes Care 34(12):2502–7. doi: 10.2337/dc11-1150 PMID: 21972411.
Russo *et al.* 2015 [21]	1. Barakat R, Cordero Y, Coteron J, Luaces M, Montejo R. Exercise during pregnancy improves maternal glucose screen at 24–28 weeks: a randomised controlled trial. Br J Sports Med 2012;46:656–61.<br>2. Barakat R, Pelaez M, Lopez C, Lucia A, Ruiz JR. Exercise during pregnancy and gestational diabetes-related adverse effects: a randomised controlled trial. Br J Sports Med 2013;47: 630–6.<br>3. Barakat R, Perales M, Bacchi M, Coteron J, Refoyo I. A program of exercise throughout pregnancy. Is it safe to mother and newborn? Am J Health Promot 2014;29:2–8.<br>4. Callaway LK, Colditz PB, Byrne NM, Lingwood BE, Rowlands IJ, Foxcroft K, et al. Prevention of gestational diabetes: feasibility issues for an exercise intervention in obese pregnant women. Diabetes Care 2010;33:1457–9.<br>5. Ko CW, Napolitano PG, Lee SP, Schulte SD, Ciol MA, Beresford SA. Physical activity, maternal metabolic measures, and the incidence of gallbladder sludge or stones during pregnancy: a randomized trial. Am J Perinatol 2014;31:39–48.<br>6. Oostdam N, van Poppel MN, Wouters MG, Eekhoff EM, Bekedam DJ, Kuchenbecker WK, et al. No effect of the FitFor2 exercise programme on blood glucose, insulin sensitivity, and birthweight in pregnant women who were overweight and at risk for gestational diabetes: results of a randomised controlled trial. BJOG 2012;119:1098–107.

Review	References of included behavioural interventions delivered in pregnancy
	7. Price BB, Amini SB, Kappeler K. Exercise in pregnancy: effect on fitness and obstetric outcomes—a randomized trial. Med Sci Sports Exerc 2012;44:2263–9.<br>8. Renault KM, Norgaard K, Nilas L, Carlsen EM, Cortes D, Pryds O, et al. The Treatment of Obese Pregnant Women (TOP) study: a randomized controlled trial of the effect of physical activity intervention assessed by pedometer with or without dietary intervention in obese pregnant women. Am J Obstet Gynecol 2014;210:134.e1–9.<br>9. Stafne SN, Salvesen KÅ, Romundstad PR, Eggebo TM, Carlsen SM, Morkved S. Regular exercise during pregnancy to prevent gestational diabetes: a randomized controlled trial. Obstet Gynecol 2012;119:29–36.<br>10. Tomic V, Sporis G, Tomic J, Milanovic Z, ZigmundovacKlaic D, Pantelic S. The effect of maternal exercise during pregnancy on abnormal fetal growth. Croat Med J 2013;54: 362–8.
Sanabria-Martínez *et al.* 2015 [22]	1. Ruiz J, Perales M, Pelaez M, Lopez C, Lucia A, Barakat R. Supervised exercise-based intervention to prevent excessive gestational weight gain: a randomized controlled trial. May Clin Proc 2013;88:1388– 97.<br>2. Tomic V, Sporis G, Tomic J, Milanovic Z, Zigmundovac-Klaic D, Pantelic S. The effect of maternal exercise during pregnancy on abnormal fetal growth. Croat Med J 2013;54:362–8<br>3. Barakat R, Perales M, Bacchi M, Coteron J, Refoyo I. A program of exercise throughout pregnancy. Is it safe to mother and newborn?. Am J Health Promot 2014;2014:2014–8.<br>4. Barakat R, Pelaez M, Lopez C, Lucia A, Ruiz JR. Exercise during pregnancy and gestational diabetes-related adverse effects: a randomized controlled trial. Br J Sports Med 2013;47:630–6.<br>5. Barakat R, Pelaez M, Montejo R, Refoyo I, Coteron J. Exercise throughout pregnancy does not cause preterm delivery. A randomized controlled trial. J Phys Act Health 2014;11:1012–7.<br>6. Barakat R, Cordero Y, Coteron J, Luaces M, Montejo R. Exercise during pregnancy improves maternal glucose screen at 24- 28 weeks: a randomized controlled trial. Br J Sports Med 2012;46:656–61<br>7. Price BB, Saeid B, Kappeper K. Exercise in pregnancy: effect on fitness and obstetric outcomes-A randomized trial. Med Sci Sports Exerc 2012;44:2263–9.<br>8. Barakat R, Pelaez M, Montejo R, Luaces M, Zakynthinaki M. Exercise during pregnancy improves maternal health perception: a randomized controlled trial. Am J Obstet Gynecol 2011;204:402 e1–7<br>9. Haakstad LAH, Bø K. Exercise in pregnant women and birth weight: a randomized controlled trial. BMC Pregnancy Childbirth 2011;11:66.<br>10. Hopkins S, Baldi JC, Cutfield WS, McCowan L, Hofman PL. Effects of exercise training on maternal hormonal changes in pregnancy. Clin Endocrinol 2011;74:495–500.<br>11. Barakat R, Ruiz JR, Stirling JR, Zakynthinaki M, Lucia A. Type of delivery is not affected by light resistance and toning exercise training during pregnancy: a randomized controlled trial. Am J Obstet Gynecol 2009;201:590.e1–6.<br>12. Cavalcante SR, Cecatti JG, Pereira RI, Baciuk EP, Bernardo AL, Silveira C. Water aerobics II: maternal body composition and perinatal outcomes after a program for low risk pregnant women. Reprod Health 2009;6:1.<br>13. Clapp JF, Kim H, Burciu B, Lopez B. Beginning regular exercise in early pregnancy: effect on fetoplacental growth. Am J Obstet Gynecol 2000;183:1484–8.
Streuling *et al.* 2011 [23]	1. Barakat R, Lucia A, Ruiz JR. Resistance exercise training during pregnancy and newborn's birth size: a randomised controlled trial. Int J Obes (Lond) 2009;33:1048–57.<br>2. Cavalcante SR, Cecatti JG, Pereira RI, Baciuk EP, Bernardo AL, Silveira C. Water aerobics II: maternal body composition and perinatal outcomes after a program for low risk pregnant women. Reprod Health 2009;6:1.<br>3. Clapp JF III, Kim H, Burciu B, Schmidt S, Petry K, Lopez B. Continuing regular exercise during pregnancy: effect of exercise volume on fetoplacental growth. Am J Obstet Gynecol 2002;186:142–7.<br>4. Collings CA, Curet LB, Mullin JP. Maternal and fetal responses to a maternal aerobic exercise program. Am J Obstet Gynecol 1983;145: 702–7.<br>5. Garshasbi A, Faghih Zadeh S. The effect of exercise on the intensity of low back pain in pregnant women. Int J Gynaecol Obstet 2005;88:271–5.<br>6. Hopkins SA, Baldi JC, Cutfield WS, McCowan L, Hofman PL. Exercise training in pregnancy reduces offspring size without changes in maternal insulin sensitivity. J Clin Endocrinol Metab 2010;95: 2080–8.<br>7. Marquez-Sterling S, Perry AC, Kaplan TA, Halberstein RA, Signorile JF. Physical and psychological changes with vigorous exercise in sedentary primigravidae. Med Sci Sports Exerc 2000;32:58–62.<br>8. Ong MJ, Guelfi KJ, Hunter T, Wallman KE, Fournier PA, Newnham JP. Supervised home-based exercise may attenuate the decline of glucose tolerance in obese pregnant women. Diabetes Metab 2009;35:418–21<br>9. Prevedel T, Calderon I, De Conti M, Consonni E, Rudge M. Maternal and perinatal effects of hydrotherapy in pregnancy. RBGO 2003; 25:53–9.<br>10. Santos IA, Stein R, Fuchs SC, Duncan BB, Ribeiro JP, Kroeff LR, et al. Aerobic exercise and submaximal functional capacity in overweight pregnant women: a randomized trial. Obstet Gynecol 2005;106: 243–9.<br>11. Sedaghati P, Ziaee V, Ardjmand A. The effect of an ergometric training program on pregnants weight gain and low back pain. Gazz Med Ital- Arch Sci Med 2007;166:209–13<br>12. Yeo S. Adherence to walking or stretching, and risk of preeclampsia in sedentary pregnant women. Res Nurs Health 2009;32: 379–90.
Sui *et al.* 2012 [24]	1. Brankston GN, Mitchell BF, Ryan EA, Okun NB. Resistance exercise decreases the need for insulin in overweight women with gestational diabetes mellitus. Am J Obstet Gynecol. 2004;190:188–93.<br>2. Barakat R, Lucia A, Ruiz JR. Resistance exercise training during pregnancy and newborn's birth size: a randomised controlled trial. Int J Obes (Lond). 2009;33:1048–57.<br>3. Ong MJ, Guelfi KJ, Hunter T,Wallman KE, Fournier PA, Newnham JP. Supervised home-based exercise may attenuate the decline of glucose tolerance in obese pregnant women. Diabetes Metab. 2009;35:418–21.<br>4. Santos IA, Stein R, Fuchs SC, Duncan BB, Ribeiro JP, Kroeff LR, et al. Aerobic exercise and submaximal functional capacity in overweight pregnant women: a randomized trial. Obstet Gynecol. 2005;106:243–9.<br>5. Callaway LK, Colditz PB, Byrne NM, Lingwood BE, Rowlands IJ, Foxcroft K, et al. Prevention of gestational diabetes: feasibility issues for an exercise intervention in obese pregnant women. Diabetes Care. 2010;33:1457–9.<br>6. Stutzman SS, Brown CA, Hains SM, Godwin M, Smith GN, Parlow JL, et al. The effects of exercise conditioning in normal and overweight pregnant women on blood pressure and heart rate variability. Biol Res Nurs. 2010;12:137–48.<br>7. Nascimento SL, Surita FG, Parpinelli M, Siani S, Pinto e Silva JL. The effect of an antenatal physical exercise programme on maternal/perinatal outcomes and quality of life in overweight and obese pregnant women: a randomised clinical trial. BJOG. 2011;118:1455–63.

Review	References of included behavioural interventions delivered in pregnancy
Tanentsapf *et al.* 2011 [25]	1. Asbee SM, Jenkins TR, Butler JR, White J, Elliot M, Rutledge A: Preventing excessive weight gain during pregnancy through dietary and lifestyle counseling: a randomized controlled trial. Obstet Gynecol 2009, 113:305-312.<br>2. Campbell DM, MacGillivray I: The effect of a low calorie diet or a thiazide diuretic on the incidence of pre-eclampsia and on birth weight. Br J Obstet Gynaecol 1975, 82:572-577.<br>3. Guelinckx I, Devlieger R, Mullie P, Vansant G: Effect of lifestyle intervention on dietary habits, physical activity, and gestational weight gain in obese pregnant women: a randomized controlled trial. Am J Clin Nutr 2010, 91:373-380.<br>4. Huang TT, Yeh CY, Tsai YC: A diet and physical activity intervention for preventing weight retention among Taiwanese childbearing women: a randomised controlled trial. Midwifery 2011, 27:257-264.<br>5. Ilmonen J, Isolauri E, Poussa T, Laitinen K: Impact of dietary counselling and probiotic intervention on maternal anthropometric measurements during and after pregnancy: a randomized placebo-controlled trial. Clin Nutr 2011, 30:156-164.<br>6. Kinnunen TI, Pasanen M, Aittasalo M, Fogelholm M, Hilakivi-Clarke L, Weiderpass E, et al: Preventing excessive weight gain during pregnancy -a controlled trial in primary health care. Eur J Clin Nutr 2007, 61:884-891.<br>7. Phelan S, Phipps MG, Abrams B, Darroch F, Schaffner A, Wing RR: Randomized trial of a behavioral intervention to prevent excessive gestational weight gain: the Fit for Delivery Study. Am J Clin Nutr 2011, 93:772-779.<br>8. Polley BA, Wing RR, Sims CJ: Randomized controlled trial to prevent excessive weight gain in pregnant women. Int J Obes Relat Metab Disord 2002, 26:1494-1502.<br>9. Thornton YS, Smarkola C, Kopacz SM, Ishoof SB: Perinatal outcomes in nutritionally monitored obese pregnant women: a randomized clinical trial. J Natl Med Assoc 2009, 101:569-577.<br>10. Wolff S, Legarth J, Vangsgaard K, Toubro S, Astrup A: A randomized trial of the effects of dietary counseling on gestational weight gain and glucose metabolism in obese pregnant women. Int J Obes (Lond) 2008, 32:495-501.<br>11. Badrawi H, Hassanein MK, Badraoui MHH, Wafa YA, Shawky HA, Badrawi N: Pregnancy outcome in obese pregnant mothers. New Egyptian Journal of Medicine 1993, 6:1717-1726.<br>12. Campbell DM: Dietary restriction in obesity and its effect on neonatal outcome. 1982, 243-250.<br>13. Hui AL, Ludwig SM, Gardiner P, Sevenhuysen G, Murray R, Morris M: Community-based exercise and dietary intervention during pregnancy: a pilot study. Can J Diabetes 2006, 30:169-175.
Thangaratinam *et al.* 2012a [26]	1. Artal R, Catanzaro RB, Gavard JA, Mostello DJ, Friganza JC. A lifestyle intervention of weight-gain restriction: diet and exercise in obese women with gestational diabetes mellitus. Appl Physiol Nutr Metab 2007;32:596–601.<br>2. Borberg C, Gillmer MD, Brunner EJ, Gunn PJ, Oakley NW, Beard RW. Obesity in pregnancy:the effect of dietary advice. Diabetes Care 1980;3:476–81.<br>3. Campbell DM, MacGillivray I. The effect of a low calorie diet or a thiazide diuretic on the incidence of pre-eclampsia and on birth weight. Br J Obstet Gynaecol 1975;82:572–7.<br>4. Campbell DM. Dietary restriction in obesity and its effect on neonatal outcome. In Campbell DM, Gillmer MDG, editors. Nutrition in pregnancy. Proceedings of the 10th Study Group of the RCOG. London: 1983. pp. 243–50.<br>5. Casanueva E, Legarreta D, Diaz-Barriga M, Soberanis Y, Cardenas T, Iturriaga A, et al. Weight gain during pregnancy in adolescents: evaluation of a non-nutritional intervention. Rev Invest Clin 1994;46:157–61.<br>6. Claesson I-M, Sydsjo G, Brynhildsen J, Cedergren M, Jeppsson A, Nystrom F, et al. Weight gain restriction for obese pregnant women: a case–control intervention study. BJOG 2008;115:44–50.<br>7. Claesson I-M, Brynhildsen J, Cedergren M, Jeppsson A, Sydsjo A, Josefsson A. Weight gain restriction during pregnancy is safe for both the mother and neonate. Acta Obstet Gynecol Scand 2009;88:1158–62.<br>8. Clapp JF, Little KD. Effect of recreational exercise on pregnancy weight gain and subcutaneous fat deposition. Med Sci Sports Exerc 1995;27:170–7.<br>9. Collings CA, Curet LB, Mullin JP. Maternal and fetal responses to a maternal aerobic exercise programme. Am J Obstet Gynecol 1983;145:702–7.<br>10. El Hiday MM, Zumrawi FY. The effect of a nutrition education programme on pregnant women attending Khartoum model clinic. Ahfad J 1992;9:23–36.<br>11. Gray-Donald K, Robinson E, Collier A, David K, Renaud L, Rodrigues S. Intervening to reduce weight gain in pregnancy and gestational diabetes mellitus in Cree communities: an evaluation. Can Med Assoc J 2000;163:1247–51.<br>12. Hall DC, Kaufmann DA. Effects of aerobic and strength conditioning on pregnancy outcomes. Am J Obstet Gynecol 1987;157:1199–203.<br>13. Kardel KR, Kase T. Training in pregnant women: effects on fetal development and birth. Am J Obstet Gynecol 1998;178:280–6.<br>14. Kinnunen TI, Pasanen M, Aittasalo M, Fogelholm M, Hilakivi-Clarke L, Weiderpass E, et al. Preventing excessive weight gain during pregnancy – a controlled trial in primary health care. Eur J Clin Nutr 2007;61:884–91.<br>15. Moses RG, Luebcke M, Davis WS, Coleman KJ, Tapsell LC, Petocz P, et al. Effect of a low-glycemic-index diet during pregnancy on obstetric outcomes. Am J Clin Nutr 2006;84:807–12.<br>16. Narendran S, Nagarathna R, Narendran V, Gunasheela S, Nagendra HR. Efficacy of yoga on pregnancy outcome. J Altern Complement Med 2005;11:237–44.<br>17. Bell RJ, Palma SM, Lumley JM. The effect of vigorous exercise during pregnancy on birthweight. Aust N Z J Obstet Gynaecol 1995;35:46–51.<br>18. Bungum TJ, Peaslee DL, Jackson AW, Perez MA. Exercise during pregnancy and type of delivery in nulliparae. J Obstet Gynecol Neonatal Nurs 2000;29:258–64.<br>19. Clapp JF III, Dickstein S. Endurance exercise and pregnancy outcome. Med Sci Sports Exerc 1984;16:556–62.<br>20. Clapp JF III, Capeless EL. Neonatal morphometrics after endurance exercise during pregnancy. Am J Obstet Gynecol 1990;163:1805–11.<br>21. Clapp JF III. The course of labor after endurance exercise during pregnancy. Am J Obstet Gynecol 1990;163:1799–805.<br>22. Cogswell ME, Scanlon KS, Fein SB, Schieve LA. Medically advised, mother's personal target, and actual weight gain during pregnancy. Obstet Gynecol 1999;94:616–22.<br>23. Conway R, Reddy S, Davies J. Dietary restraint and weight gain during pregnancy. Eur J Clin Nutr 1999;53:849–53.<br>24. Dale E, Mullinax KM, Bryan DH. Exercise during pregnancy: effects on the fetus. Can J Appl Sport Sci 1982;7:98–103.<br>25. de Rooij SR, Painter RC, Holleman F, Bossuyt PM, Roseboom TJ. The metabolic syndrome in adults prenatally exposed to the Dutch famine. Am J Clin Nutr 2007;86:1219–24.

Review	References of included behavioural interventions delivered in pregnancy
	26. Dempsey JC, Sorensen TK, Williams MA, Lee IM, Miller RS, Dashow EE, et al. Prospective study of gestational diabetes mellitus risk in relation to maternal recreational physical activity before and during pregnancy. Am J Epidemiol 2004;159:663–70.<br>27. Hatch MC, Shu XO, McLean DE, Levin B, Begg M, Reuss L, et al. Maternal exercise during pregnancy, physical fitness, and fetal growth. Am J Epidemiol 1993;137:1105–14.<br>28. Horns PN, Ratcliffe LP, Leggett JC, Swanson MS. Pregnancy outcomes among active and sedentary primiparous women. J Obstet Gynecol Neonatal Nurs 1996;25:49–54.<br>29. Jackson MR, Gott P, Lye SJ, Ritchie JW, Clapp JF. The effects of maternal aerobic exercise on human placental development: placental volumetric composition and surface areas. Placenta 1995;16:179–91.<br>30. Knudsen VK, Orozova-Bekkevold IM, Mikkelsen TB, Wolff S, Olsen SF. Major dietary patterns in pregnancy and fetal growth. Eur J Clin Nutr 2008;62:463–70.<br>31. Lenders CM, Hediger ML, Scholl TO, Khoo CS, Slap GB, Stallings VA. Effect of high sugar intake by low-income pregnant adolescents on infant birth weight. J Adolesc Health 1994;15:596–602.<br>32. Lenders CM, Hediger ML, Scholl TO, Khoo CS, Slap GB, Stallings VA. Gestational age and infant size at birth are associated with dietary sugar intake among pregnant adolescents. J Nutr 1997;127:1113–17.<br>33. Lumey LH, Stein AD, Kahn HS, Romijn JA. Lipid profiles in middle-aged men and women after famine exposure during gestation: the Dutch Hunger Winter Families Study. Am J Clin Nutr 2009;89:1737–43.<br>34. Magann EF, Evans SF, Weitz B, Newnham J. Antepartum, intrapartum, and neonatal significance of exercise on healthy low-risk pregnant working women. Obstet Gynecol 2002;99:466–72.<br>35. Melzer K, Schutz Y, Soehnchen N, Othenin-Girard V, Martinez de TB, Irion O, et al. Effects of recommended levels of physical activity on pregnancy outcomes. Am J Obstet Gynecol 2010;202:266. e1–6. Epub 2009 Dec 22.<br>36. Mottola MF, Giroux I, Gratton R, Hammond JA, Hanley A, Harris S, et al. Nutrition and exercise prevent excess weight gain in overweight pregnant women. Med Sci Sports Exerc 2010;42:265–72.<br>37. Neugebauer R, Hoek HW, Susser E. Prenatal exposure to wartime famine and development of antisocial personality disorder in early adulthood. JAMA 1999;282:455–62.<br>38. Olson CM, Strawderman MS, Reed RG. Efficacy of an intervention to prevent excessive gestational weight gain. Am J Obstet Gynecol 2004;191:530–6.<br>39. Perichart PO, Balas NM, Parra CA, Rodriguez CA, Ramirez TA, Ortega GC, et al. A medical nutrition therapy programme improves perinatal outcomes in Mexican pregnant women with gestational diabetes and type 2 diabetes mellitus. Diabetes Educ 2009;35:1004–13.<br>40. Piravej K, Saksirinukul R. Survey of patterns, attitudes, and the general effects of exercise during pregnancy in 203 Thai pregnant women at King Chulalongkorn Memorial Hospital. J Med Assoc Thailand 2001;84(Suppl. 1):S276–82.<br>41. Shirazian T, Monteith S, Friedman F, Rebarber A. Lifestyle modification programme decreases pregnancy weight gain in obese women. Am J Perinatol 2010;27:411–14.<br>42. Stein AD, Kahn HS, Rundle A, Zybert PA, van der Pal-de Bruin, Lumey LH. Anthropometric measures in middle age after exposure to famine during gestation: evidence from the Dutch famine. Am J Clin Nutr 2007;85:869–76.<br>43. Berkowitz GS, Kelsey JL, Holford TR, Berkowitz RL. Physical activity and the risk of spontaneous preterm delivery. J Reprod Med 1983;28:581–8<br>44. Dempsey JC, Butler CL, Sorensen TK, Lee IM, Thompson ML, Miller RS, et al. A case– control study of maternal recreational physical activity and risk of gestational diabetes mellitus. Diabetes Res Clin Pract 2004;66:203–15.<br>45. Dye TD, Knox KL, Artal R, Aubry RH, Wojtowycz MA. Physical activity, obesity, and diabetes in pregnancy. Am J Epidemiol 1997;146:961–5.<br>46. Gregory PB, Rush D. Iatrogenic caloric restriction in pregnancy and birthweight. Am J Perinatol 1987;4:365–71.<br>47. Oken E, Ning Y, Rifas-Shiman SL, Radesky JS, Rich-Edwards JW, Gillman MW. Associations of physical activity and inactivity before and during pregnancy with glucose tolerance. Obstet Gynecol 2006;108:1200–7<br>48. Sorensen TK, Williams MA, Lee IM, Dashow EE, Thompson ML, Luthy DA. Recreational physical activity during pregnancy and risk of preeclampsia. Hypertension 2003;41:1273–80.<br>49. Badrawi H, Hassanein MK, Badraoui MHH, Wafa YA, Shawky HA, Badrawi N. Pregnancy outcome in obese pregnant mothers. J Perinat Med 1992;20:203.<br>50. Bechtel-Blackwell DA. Computer-assisted self-interview and nutrition education in pregnant teens. Clin Nurs Res 2002;11:450–62.<br>51. Briley C, Flanagan NL, Lewis N. In-home prenatal nutrition intervention increased dietary iron intakes and reduced low birthweight in low-income African-American women. J Am Diet Assoc 2002;102:984–7.<br>52. Clapp JF. Diet, exercise, and feto–placental growth. Arch Gynecol Obstet 1997;260:101–8.<br>53. Crowther CA, Hiller JE, Moss JR, McPhee AJ, Jeffries WS, Robinson JS. Effect of treatment of gestational diabetes mellitus on pregnancy outcomes. N Engl J Med 2005;352:2477–86.<br>54. Gomez TG, Delgado JG, Agudelo AA, Hurtado H. Diet effects on the perinatal result of obese pregnant patient. [Spanish]. Rev Colomb Obstet Ginecol 1994;45:313-6.<br>55. Khoury J, Henriksen T, Christophersen B, Tonstad S. Effect of a cholesterol-lowering diet on maternal, cord, and neonatal lipids, and pregnancy outcome: a randomised clinical trial. Am J Obstet Gynecol 2005;193:1292–301.<br>56. Landon MB, Spong CY, Thom E, Carpenter MW, Ramin SM, Casey B, et al. A multicenter, randomised trial of treatment for mild gestational diabetes. N Engl J Med 2009;361:1339–48.<br>57. Ney D, Hollingsworth DR, Cousins L. Decreased insulin requirement and improved control of diabetes in pregnant women given a high-carbohydrate, high-fiber, low-fat diet. Diabetes Care 1982;5:529–33.<br>58. Rae A, Bond D, Evans S, North F, Roberman B, Walters B. A randomised controlled trial of dietary energy restriction in the management of obese women with gestational diabetes. Aust N Z J Obstet Gynaecol 2000;40:416–22.<br>59. Thornton YS, Smarkola C, Kopacz SM, Ishoof SB. Perinatal outcomes in nutritionally monitored obese pregnant women: a randomised clinical trial. J Natl Med Assoc 2009;101:569–77.<br>60. Wolff S, Legarth J, Vangsgaard K, Toubro S, Astrup A. A randomised trial of the effects of dietary counseling on gestational weight gain and glucose metabolism in obese pregnant women. Int J Obes 2008;32:495–501.<br>61. Baciuk EP, Pereira RI, Cecatti JG, Braga AF, Cavalcante SR. Water aerobics in pregnancy: cardiovascular response, labor and neonatal outcomes. Reprod Health 2008;5:10. DOI:10.1186/1742-4765-5-10.<br>62. Barakat R, Lucia A, Ruiz JR. Resistance exercise training during pregnancy and newborn's birth size: a randomised controlled trial. Int J Obes 2009;33:1048–57.<br>63. Cavalcante SR, Cecatti JG, Pereira RI, Baciuk EP, Bernardo AL, Silveira C. Water aerobics II: maternal body composition and perinatal outcomes after a programme for low risk pregnant women. Reprod Health 2009;6:1.<br>64. Clapp JF III, Kim H, Burciu B, Lopez B. Beginning regular exercise in early pregnancy: effect on fetoplacental growth. Am J Obstet Gynecol 2000;183:1484–8.

Review	References of included behavioural interventions delivered in pregnancy
	65. Clapp JF III, Kim H, Burciu B, Schmidt S, Petry K, Lopez B. Continuing regular exercise during pregnancy: effect of exercise volume on fetoplacental growth. Am J Obstet Gynecol 2002;186:142–7.<br>66. Erkkola R. The influence of physical exercise during pregnancy upon physical work capacity and circulatory parameters. Scand J Clin Lab Invest 1976;36:747–59.<br>67. Erkkola R, Makela M. Heart volume and physical fitness of parturients. Ann Clin Res 1976;8:15–21.<br>68. Garshasbi A, Faghih ZS. The effect of exercise on the intensity of low back pain in pregnant women. Int J Gynaecol Obstet 2005;88:271–5.<br>69. Haakstad L, Bo K. Effect of supervised aerobic dance exercise in prevention of excessive weight gain in pregnancy: a single blind randomised controlled trial. Int J Gynaecol Obstet 2009;107:S198.<br>70. Hopkins SA, Baldi JC, Cutfield WS, McCowan L, Hofman PL. Exercise training in pregnancy reduces offspring size without changes in maternal insulin sensitivity. J Clin Endocrinol Metab 2010;95:2080–8.<br>71. Khaledan A, Mirdar Sh, Motahari Tabari NS, Ahmad Shirvani M. Effect of an aerobic exercise programme on fetal growth in pregnant women. HAYAT 2010;16:78.<br>72. Lee G, Challenger S, McNabb M, Sheridan M. Exercise in pregnancy. Mod Midwife 1996;6:28–33.<br>73. Marquez-Sterling S, Perry AC, Kaplan TA, Halberstein RA, Signorile JF. Physical and psychological changes with vigorous exercise in sedentary primigravidae. Med Sci Sports Exerc 2000;32:58–62.<br>74. Ong MJ, Guelfi KJ, Hunter T, Wallman KE, Fournier PA, Newnham JP. Supervised homebased exercise may attenuate the decline of glucose tolerance in obese pregnant women. Diabetes Metab 2009;35:418–21.<br>75. Prevedel T, Calderon I, De Conti M, Consonni E, Rudge M. Maternal and perinatal effects of hydrotherapy in pregnancy. Rev Bras Ginecol Obstet 2003;25:53–9.<br>76. Santos IA, Stein R, Fuchs SC, Duncan BB, Ribeiro JP, Kroeff LR, et al. Aerobic exercise and submaximal functional capacity in overweight pregnant women: a randomised trial. Obstet Gynecol 2005;106:243–9.<br>77. Sedaghati P, Ziaee V, Ardjmand A. The effect of an ergometric training programme on pregnants weight gain and low back pain. Gazz Med Ital Arch Sci Med 2007;166:209–13.<br>78. Yeo S, Steele NM, Chang MC, Leclaire SM, Ronis DL, Hayashi R. Effect of exercise on blood pressure in pregnant women with a high risk of gestational hypertensive disorders. J Reprod Med 2000;45:293–8.<br>79. Yeo S, Davidge S, Ronis DL, Antonakos CL, Hayashi R, O'Leary S. A comparison of walking versus stretching exercises to reduce the incidence of preeclampsia: a randomised clinical trial. Hypertens Pregnancy 2008;27:113–30.<br>80. Yeo S. Adherence to walking or stretching, and risk of preeclampsia in sedentary pregnant women. Res Nurs Health 2009;32:379–90.<br>81. Asbee SM, Jenkins TR, Butler JR, White J, Elliot M, Rutledge A. Preventing excessive weight gain during pregnancy through dietary and lifestyle counseling: a randomised controlled trial. Obstet Gynecol 2009;113:305–12.<br>82. Bung P, Artal R, Khodiguian N, Kjos S. Exercise in gestational diabetes. An optional therapeutic approach? Diabetes 1991;40(Suppl. 2):182–5.<br>83. Guelinckx I, Devlieger R, Mullie P, Vansant G. Effect of lifestyle intervention on dietary habits, physical activity, and gestational weight gain in obese pregnant women: a randomised controlled trial. Am J Clin Nutr 2010;91:373–80.<br>84. Hui AL, Ludwig SM, Gardiner P, Sevenhuysen G, Murray R, Morris M, et al. Communitybased exercise and dietary intervention during pregnancy: a pilot study. Can J Diabetes 2006;30:169–75.<br>85. Jeffries K, Shub A, Walker SP, Hiscock R, Permezel M. Reducing excessive weight gain in pregnancy: a randomised controlled trial. Med J Aust 2009;191:429–33.<br>86. Kulpa PJ, White BM, Visscher R. Aerobic exercise in pregnancy. Am J Obstet Gynecol 1987;156:1395–403.<br>87. Polley BA, Wing RR, Sims CJ. Randomised controlled trial to prevent excessive weight gain in pregnant women. Int J Obes 2002;26:1494-502.
Thangaratinam *et al.* 2012b [27]	1. Badrawi H, Hassanein MK, Badraoui MHH, Wafa YA, Shawky HA, Badrawi N. Pregnancy outcome in obese pregnant mothers. J Perinat Med 1992;20:203.<br>2. Bechtel-Blackwell DA. Computer-assisted self-interview and nutrition education in pregnant teens. Clin Nurs Res 2002;11:450-62.<br>3. Briley C, Flanagan NL, Lewis N. In-home prenatal nutrition intervention increased dietary iron intakes and reduced low birthweight in low-income African-American women. J Am Diet Assoc 2002;102:984-7.<br>4. Clapp IJF. Diet, exercise, and fete-placental growth. Arch Gynecol Obstet 1997;260:101-8.<br>5. Crowther CA, Hiller JE, Moss JR, McPhee AJ, Jeffries WS, Robinson JS. Effect of treatment of gestational diabetes mellitus on pregnancy outcomes. N Engl J Med 2005;352:2477-86.<br>6. Gomez TG, Delgado JG, Agudelo AA, Hurtado H. Diet effects on the perinatal result of obese pregnant patient. [Spanish]. Rev Colomb Obstet Ginecol 1994;45:313-6.<br>7. Khoury J, Henriksen T, Christophersen B, Tonstad S. Effect of a cholesterol-lowering diet on maternal, cord, and neonatal lipids, and pregnancy outcome: a randomized clinical trial. Am J Obstet Gynecol 2005;193:1292-301.<br>8. Landon MB, Spong CY, Thom E, Carpenter MW, Ramin SM, Casey B, et al. A multicenter, randomized trial of treatment for mild gestational diabetes. N Engl J Med 2009;361:1339-48.<br>9. Ney D, Hollingsworth DR, Cousins L. Decreased insulin requirement and improved control of diabetes in pregnant women given a high-carbohydrate, high-fiber, low-fat diet. Diabetes Care 1982;5:529-33.<br>10. Quinlivan JA, Lam LT, Fisher J. A randomised trial of a four-step multidisciplinary approach to the antenatal care of obese pregnant women. Aust N Z J Obstet Gynaecol 2011;51:141-6.<br>11. Rae A, Bond D, Evans S, North F, Roberman B, Walters B. A randomised controlled trial of dietary energy restriction in the management of obese women with gestational diabetes. Aust N Z J Obstet Gynaecol 2000;40:416-22.<br>12. Thornton YS, Smarkola C, Kopacz SM, Ishoof SB. Perinatal outcomes in nutritionally monitored obese pregnant women: a randomized clinical trial. J Natl Med Assoc 2009;101:569-77.<br>13. Wolff S, Legarth J, Vangsgaard K, Toubro S, Astrup A. A randomized trial of the effects of dietary counseling on gestational weight gain and glucose metabolism in obese pregnant women. Int J Obes 2008;32:495-501.<br>14. Baciuk EP, Pereira RI, Cecatti JG, Braga AF, Cavalcante SR. Water aerobics in pregnancy: cardiovascular response, labor and neonatal outcomes. Reprod Health 2008;5:10.<br>15. Barakat R, Lucia A, Ruiz JR. Resistance exercise training during pregnancy and newborn's birth size: a randomised controlled trial. Int J Obes 2009;33:1048-57.<br>16. Barakat R, Cordero Y, Coteron J, Luaces M, Montejo R. Exercise during pregnancy improves maternal glucose screen at 24-28 weeks: a randomised controlled trial. Br J Sports Med 2011 Sep 26, epub ahead of print.<br>17. Bell RJ, Palma SM. Antenatal exercise and birth-weight. Aust N Z J Obstet Gynaecol 2000;40:70-3.

Review	References of included behavioural interventions delivered in pregnancy
	18. Cavalcante SR, Cecatti JG, Pereira RI, Baciuk EP, Bernardo AL, Silveira C. Water aerobics II: maternal body composition and perinatal outcomes after a program for low risk pregnant women. Reprod Health 2009;6:1.<br>19. Clapp JF, III, Kim H, Burciu B, Lopez B. Beginning regular exercise in early pregnancy: effect on fetoplacental growth. Am J Obstet Gynecol 2000;183:1484-8.<br>20. Erkkola R. The influence of physical exercise during pregnancy upon physical work capacity and circulatory parameters. Scand J Clin Lab Invest 1976;6:747-9.<br>21. Erkkola R, Makela M. Heart volume and physical fitness of parturients. Ann Clin Res 1976;8:15-21.<br>22. Garshasbi A, Faghih ZS. The effect of exercise on the intensity of low back pain in pregnant women. Int J Gynaecol Obstet 2005;88:271-5.<br>23. Haakstad L, Bo K. Exercise in pregnant women and birth weight: a randomized controlled trial. BMC Preg Childbirth 2011;11:66.<br>24. Hopkins SA, Baldi JC, Cutfield WS, McCowan L, Hofman PL. Exercise training in pregnancy reduces offspring size without changes in maternal insulin sensitivity. J Clin Endocrinol Metab 2010;95:2080-8.<br>25. Khaledan A, Sh, Motahari Tabari NS, Ahmad Shirvani M. Effect of an aerobic exercise program on fetal growth in pregnant women. HAYAT: J Faculty Nurs Midwifery 2010;16:78.<br>26. Lee G, Challenger S, McNabb M, Sheridan M. Exercise in pregnancy. Mod Midwife 1996;6:28-33.<br>27. Marquez-Sterling S, Perry AC, Kaplan TA, Halberstein RA, Signorile JF. Physical and psychological changes with vigorous exercise in sedentary primigravidae. Med Sci Sports Exerc 2000;32:58-62.<br>28. Ong MJ, Guelfi KJ, Hunter T, Wallman KE, Fournier PA, Newnham JP. Supervised home-based exercise may attenuate the decline of glucose tolerance in obese pregnant women. Diabetes Metab 2009;35:418-21.<br>29. Prevedel T, Calderon I, DM, Adami H-O, RM. Maternal and perinatal effects of hydrotherapy in pregnancy. Rev Bras Ginecol Obstet 2003;25:53-9.<br>30. Santos IA, Stein R, Fuchs SC, Duncan BB, Ribeiro JP, Kroeff LR, et al. Aerobic exercise and submaximal functional capacity in overweight pregnant women: a randomized trial. Obstet Gynecol 2005;106:243-9.<br>31. Sedaghati P, Ziaee V, Ardjmand A. The effect of an ergometric training program on pregnants weight gain and low back pain. Gazzetta Medica Italiana Archivio per le Scienze Mediche 2007;166:209-13.<br>32. Yeo S, Steele NM, Chang MC, Leclaire SM, Ronis DL, Hayashi R. Effect of exercise on blood pressure in pregnant women with a high risk of gestational hypertensive disorders. J Reprod Med 2000;45:293-8<br>33. Asbee SM, Jenkins TR, Butler JR, White J, Elliot M, Rutledge A. Preventing excessive weight gain during pregnancy through dietary and lifestyle counseling: a randomized controlled trial. Obstet Gynecol 2009;113:305-12.<br>34. Bung P, Artal R, Khodiguian N, Kjos S. Exercise in gestational diabetes. An optional therapeutic approach? Diabetes 1991;40(suppl 2):182-5.<br>35. Ferrara A, Hedderson MM, Albright CL, Ehrlich SF, Quesenbery CP, Peng TP, et al. A pregnancy and postpartum lifestyle intervention in women with gestational diabetes mellitus reduces diabetes risk factors. A feasibility randomized control trial. Diabetes Care 2011;34:1519-25.<br>36. Guelinckx I, Devlieger R, Mullie P, Vansant G. Effect of lifestyle intervention on dietary habits, physical activity, and gestational weight gain in obese pregnant women: a randomized controlled trial. Am J Clin Nutr 2010;91:373-80.<br>37. Huang TT, Yeh CY, Tsai YC. A diet and physical activity intervention for preventing weight retention among Taiwanese childbearing women: a randomised controlled trial. Midwifery 2011;27:257-64.<br>38. Hui A, Back L, Ludwig S, Gardiner P, Sevenhuysen G, Dean H, et al. Lifestyle intervention on diet and exercise reduced excessive gestational weight gain in pregnant women under a randomised controlled trial. BJOG 2011;119:70-7.<br>39. Hui AL, Ludwig SM, Gardiner P, Sevenhuysen G, Murray R, Morris M, et al. Community-based exercise and dietary intervention during pregnancy: a pilot study. Can J Diabetes 2006;30:169-75.<br>40. Jeffries K, Shub A, Walker SP, Hiscock R, Permezel M. Reducing excessive weight gain in pregnancy: a randomised controlled trial. Med J Aust 2009;191:429-33.<br>41. Kulpa PJ, White BM, Visscher R. Aerobic exercise in pregnancy. Am J Obstet Gynecol 1987;156:1395-403.<br>42. Phelan S, Phipps MG, Abrams B, Darroch F, Schaffner A, Wing RR. Randomized trial of a behavioral intervention to prevent excessive gestational weight gain: the Fit for Delivery Study. Am J Clin Nutr 2011;93:772-9.<br>43. Polley BA, Wing RR, Sims CJ. Randomized controlled trial to prevent excessive weight gain in pregnant women. Int J Obes 2002;26:1494-502.<br>44. Vinter CA, Jensen DM, Ovesen P, Beck-Nielsen H, Jorgensen JS. The LiP (Lifestyle in Pregnancy) study: a randomized controlled trial of lifestyle intervention in 360 obese pregnant women. Diabetes Care 2011;34:2502-7.<br>45. Jackson RA, Stotland NE, Caughey AB, Gerbert B. Improving diet and exercise in pregnancy with Video Doctor counseling: a randomized trial. Patient Educ Couns 2011;83:203-9.
da Silva *et al.* 2017 [28]	1. Tomic V, Sporis G, Tomic J, et al. The effect of maternal exercise during pregnancy on abnormal fetal growth. Croat Med J. 2013;54(4):362–8.<br>2. Evenson K, Barakat R, Brown W, et al. Guidelines for physical activity during pregnancy: comparisons from around the world. Am J Lifestyle Med. 2014;8(2):102–21.<br>3. Owe KM, Nystad W, Bø K. Association between regular exercise and excessive newborn birth weight. Obstet Gynecol. 2009;114(4):770–6. doi:10.1097/AOG.0b013e3181b6c105.<br>4. Hegaard HK, Petersson K, Hedegaard M, et al. Sports and leisure- time physical activity in pregnancy and birth weight: a population-based study. Scand J Med Sci Sports. 2010;20(1): e96–102. doi:10.1111/j.1600-0838.2009.00918.x.<br>5. de Oliveria Melo AS, Silva JL, Tavares JS, et al. Effect of a physical exercise program during pregnancy on uteroplacental and fetal blood flow and fetal growth: a randomized controlled trial. Obstet Gynecol. 2012;120(2 Pt 1):302–10. doi:10.1097/AOG.0b013e31825de592.<br>6. Clapp JF, Kim H, Burciu B, et al. Beginning regular exercise in early pregnancy: effect on fetoplacental growth. Am J Obstet Gynecol. 2000;183(6):1484–8.<br>7. Salvesen KA, Stafne SN, Eggebo TM, et al. Does regular exercise in pregnancy influence duration of labor? A secondary analysis of a randomized controlled trial. Acta Obstet Gynecol Scand. 2014;93(1):73–9. doi:10.1111/aogs.12260.<br>8. Barakat R, Pelaez M, Montejo R, et al. Exercise throughout pregnancy does not cause preterm delivery: a randomized, controlled trial. J Phys Act Health. 2014;11(5):1012–7. doi:10.1123/jpah.2012-0344.<br>9. Murtezani A, Pac¸arada M, Ibraim Z, et al. The impact of exercise during pregnancy on neonatal outcomes: a randomized controlled trial. J Sports Med Phys Fitness. 2014;54(6):802–8.

Review	References of included behavioural interventions delivered in pregnancy
	10. Ruiz JR, Perales M, Pelaez M, et al. Supervised exercise-based intervention to prevent excessive gestational weight gain: a randomized controlled trial. Mayo Clin Proc. 2013;88(12): 1388–97. doi:10.1016/j.mayocp.2013.07.020.<br>11. Kihlstrand M, Stenman B, Nilsson S, et al. Water-gymnastics reduced the intensity of back/low back pain in pregnant women. Acta Obstet Gynecol Scand. 1999;78(3):180–5.<br>12. Hopkins SA, Baldi JC, Cutfield WS, et al. Effects of exercise training on maternal hormonal changes in pregnancy. Clin Endocrinol (Oxf). 2011;74(4):495–500. doi:10.1111/j.1365-2265.2010.03964.x.<br>13. Barakat R, Lucia A, Ruiz JR. Resistance exercise training during pregnancy and newborn's birth size: a randomised controlled trial. Int J Obes (Lond). 2009;33(9):1048–57. doi:10.1038/ijo.2009.150.<br>14. Barakat R, Stirling JR, Lucia A. Does exercise training during pregnancy affect gestational age? A randomised controlled trial. Br J Sports Med. 2008;42(8):674–8. doi:10.1136/bjsm.2008.047837.<br>15. Sedaghati P, Ziaee V, Ardjmand A. The effect of an ergometric training program on pregnants' weight gain and low back pain. Gazz Med Ital. 2007;166:209–13.<br>16. Stafne SN, Salvesen KA, Romundstad PR, et al. Regular exercise during pregnancy to prevent gestational diabetes: a randomized controlled trial. Obstet Gynecol. 2012;119(1):29–36.doi:10.1097/AOG.0b013e3182393f86.<br>17. Barakat R, Pelaez M, Lopez C, et al. Exercise during pregnancy and gestational diabetes-related adverse effects: a randomised controlled trial. Br J Sports Med. 2013;47(10):630–6. doi:10.1136/bjsports-2012-091788.<br>18. Barakat R, Cordero Y, Coteron J, et al. Exercise during pregnancy improves maternal glucose screen at 24–28 weeks: a randomised controlled trial. Br J Sports Med. 2012;46(9): 656–61. doi:10.1136/bjsports-2011-090009.<br>19. Barakat R, Pelaez M, Montejo R, et al. Exercise during pregnancy improves maternal health perception: a randomized controlled trial. Am J Obstet Gynecol. 2011;204(5):402.e1–7. doi:10.1016/j.ajog.2011.01.043.<br>20. Cordero Y,Mottola MF, Vargas J, et al. Exercise is associated with a reduction in gestational diabetes mellitus. Med Sci Sports Exerc. 2015;47(7):1328–33. doi:10.1249/MSS.0000000000000547.<br>21. Petrov Fieril K, Glantz A, Fagevik OM. The efficacy of moderate- to-vigorous resistance exercise during pregnancy: a randomized controlled trial. Acta Obstet Gynecol Scand. 2015;94(1):35–42. doi:10.1111/aogs.12525.<br>22. Barakat R, Perales M, Bacchi M, et al. A program of exercise throughout pregnancy. Is it safe to mother and newborn?AmJ Health Promot. 2014;29(1):2–8. doi:10.4278/ajhp.130131-QUAN-56.<br>23. Garshasbi A, Zadeh SF. The effect of exercise on the intensity of low back pain in pregnant women. Int J Gynaecol Obstet.2005;88(3):271–5.<br>24. Haakstad LA, Bo K. Effect of regular exercise on prevention of excessive weight gain in pregnancy: a randomised controlled trial. Eur J Contracept Reprod Health Care. 2011;16(2):116–25.doi:10.3109/13625187.2011.560307.<br>25. Haakstad LA, Bo K. Exercise in pregnant women and birth weight: a randomized controlled trial. BMC Pregnancy Childbirth. 2011;30(11):66. doi:10.1186/1471-2393-11-66.<br>26. Ramı´rez-Ve´lez R, Aguilar de Plata AC, Mosquera-Escudero M, et al. Efecto del ejercicio físico aeróbico sobre el consumo de oxígeno de mujeres primigestantes saludables: estudio clínico aleatorizado. [The effect of aerobic exercise on oxygen consumption in healthy first-pregnancy females: a randomized clinical trial]. Rev Colomb Obstet Ginecol. 2011;62(1):15–23.<br>27. Marquez-Sterling S, Perry AC, Kaplan TA, et al. Physical and psychological changes with vigorous exercise in sedentary primigravidae. Med Sci Sports Exerc. 2000;32(1):58–62.<br>28. Price BB, Amini SB, Kappeler K. Exercise in Pregnancy: effect on fitness and obstetric outcomes—a randomized trial. Med Sci Sports Exerc. 2012;44(12):2263–9. doi:10.1249/MSS.0b013 e318267ad67.<br>29. Rodrı´guez YC, Puente MP, Abad MDM, et al. Puede el ejercicio físico moderado durante el embarazo actuar como un factor de prevencion de la diabetes gestacional? [Can moderate physical exercise during pregnancy act as a factor in preventing gestational diabetes?]. Rev Int Cienc Deporte. 2012;8(27):3–19.<br>30. Cavalcante SR, Cecatti JG, Pereira RI, et al. Water aerobics II: maternal body composition and perinatal outcomes after a program for low risk pregnant women. Reprod Health. 2009;6(6):1. doi:10.1186/1742-4755-6-1.<br>31. Baciuk EP, Pereira RI, Cecatti JG, et al. Water aerobics in pregnancy: cardiovascular response, labor and neonatal outcomes. Reprod Health. 2008;5:10. doi:10.1186/1742-4755-5-10.<br>32. Prevedel T, Calderon I, De Conti M, et al. Maternal and perinatal effects of hydrotherapy in pregnancy. Rev Bras Ginecol Obstet. 2003;25:53–9.<br>33. Melzer K, Schutz Y, Soehnchen N, et al. Effects of recommended levels of physical activity on pregnancy outcomes. Am J Obstet Gynecol. 2010;202(3):266.e1–6. doi:10.1016/j.ajog. 2009.10.876.<br>34. Morgan KL, Rahman MA, Hill RA, et al. Physical activity and excess weight in pregnancy have independent and unique effects on delivery and perinatal outcomes. PLoS One. 2014;9(4): e94532. doi:10.1371/journal.pone.0094532.<br>35. Morkrid K, Jenum AK, Berntsen S, et al. Objectively recorded physical activity and the association with gestational diabetes. Scand J Med Sci Sports. 2014;24(5):e389–97. doi:10.1111/sms.12183.<br>36. Perkins CC, Pivarnik JM, Paneth N, et al. Physical activity and fetal growth during pregnancy. Obstet Gynecol. 2007;109(1): 81–7.<br>37. Schlaff RA, Holzman C, Maier KS, et al. Associations among gestational weight gain, physical activity, and pre-pregnancy body size with varying estimates of pre-pregnancy weight. Midwifery. 2014;30(11):1124–31. doi:10.1016/j.midw.2014.03.014.<br>38. Chasan-Taber L, Silveira M, Lynch KE, et al. Physical activity and gestational weight gain in Hispanic women. Obesity. 2014;22(3):909–18. doi:10.1002/oby.20549.<br>39. Jiang H, Qian X, Li M, et al. Can physical activity reduce excessive gestational weight gain? Findings from a Chinese urban pregnant women cohort study. Int J Behav Nutr Phys Act. 2012;9(9):12. doi:10.1186/1479-5868-9-12.<br>40. Chasan-Taber L, SchmidtMD, PekowP, et al. Physical activity and gestational diabetes mellitus among Hispanic women. J Womens Health. 2008;17(6):999–1008. doi:10.1089/jwh.2007.0560.<br>41. Vollebregt KC, Wolf H, Boer K, et al. Does physical activity in leisure time early in pregnancy reduce the incidence of preeclampsia or gestational hypertension? Acta Obstet Gynecol Scand. 2010;89(2):261–7. doi:10.3109/00016340903433982.<br>42. Sternfeld B, Quesenberry CP, Eskenazi B, et al. Exercise during pregnancy and pregnancy outcome. Med Sci Sports Exerc. 1995;27(5):634–40.<br>43. Hatch MC, Shu XO, McLean DE, et al. Maternal exercise during pregnancy, physical fitness, and fetal growth. Am J Epidemiol.1993;137(10):1105–14.<br>44. Hegaard HK, Hedegaard M, Damm P, et al. Leisure time physical activity is associated with a reduced risk of preterm delivery. Am J Obstet Gynecol. 2008;198(2):180.e1–5. doi:10.1016/j.ajog.2007.08.038.<br>45. Fleten C, Stigum H, Magnus P, et al. Exercise during pregnancy,maternal prepregnancy body mass index, and birth weight.Obstet Gynecol. 2010;115(2 Pt 1):331–7. doi:10.1097/AOG.0b013e3181ca4414.<br>46. Owe KM, Nystad W, Skjaerven R, et al. Exercise during pregnancy and the gestational age distribution: a cohort study. Med Sci Sports Exerc. 2012;44(6):1067–74. doi:10.1249/MSS.0b013 e3182442fc9.

Review	References of included behavioural interventions delivered in pregnancy
	47. Evenson KR, Siega-Riz AM, Savitz DA, et al. Vigorous leisure activity and pregnancy outcome. Epidemiology. 2002;13(6):653–9.<br>48. Harrod CS, Chasan-Taber L, Reynolds RM, et al. Physical activity in pregnancy and neonatal body composition: the healthy start study. Obstet Gynecol. 2014;124(2 Pt 1):257–64. doi:10.1097/AOG.0000000000000373.<br>49. Gollenberg AL, Pekow P, Bertone-Johnson ER, et al. Physical activity and risk of small-for-gestational-age birth among predominantly Puerto Rican women. Matern Child Health J. 2011;15(1):49–59. doi:10.1007/s10995-009-0563-1.<br>50. Misra DP, Strobino DM, Stashinko EE, et al. Effects of physical activity on preterm birth. Am J Epidemiol. 1998;147(7):628–35.<br>51. Domingues MR, Barros AJ, Matijasevich A. Leisure time physical activity during pregnancy and preterm birth in Brazil. Int J Gynaecol Obstet. 2008;103:9–15.<br>52. McCullough LE, Mendez MA, Miller EE, et al. Associations between prenatal physical activity, birth weight, and DNA methylation at genomically imprinted domains in a multi-ethnic newborn cohort. Epigenetics. 2015;10(7):597–606. doi:10.1080/15592294.2015.1045181.<br>53. Chasan-Taber L, Silveira M, Pekow P, et al. Physical activity, sedentary behavior and risk of hypertensive disorders of pregnancy in Hispanic women. Hypertens Pregnancy. 2015;34(1): 1–16. doi:10.3109/10641955.2014.946616.<br>54. Currie L, Woolcott C, Fell D, et al. The association between physical activity and maternal and neonatal outcomes: a prospective cohort. Matern Child Health J. 2014;18(8):1823–30. doi:10.1007/s10995-013-1426-3.<br>55. Portela SN, Rocha-de-Souza R, Oppermann-Lisboa K, et al. Maternal physical activity, cervical length and its relation to spontaneous vaginal birth at term. Arch Gynecol Obstet. 2014;290(2):257–62. doi:10.1007/s00404-014-3198-4.<br>56. Restall A, Taylor RS, Thompson JM, et al. Risk factors for excessive gestational weight gain in a healthy, nulliparous cohort. J Obes. 2014;2014:148391. doi:10.1155/2014/148391.<br>57. Sealy-Jefferson S, Hegner K, Misra DP. Linking non-traditional physical activity and preterm delivery in urban African-American women. Womens Health Issues. 2014;24(4):e389–95. doi:10.1016/j.whi.2014.04.007.<br>58. Tinloy J, Chuang CH, Zhu J, et al. Exercise during pregnancy and risk of late preterm birth, cesarean delivery, and hospitalizations. Womens Health Issues. 2014;24(1):e99–104. doi:10. 1016/j.whi.2013.11.003.<br>59. Kraschnewski JL, Chuang CH, Downs DS, et al. Association of prenatal physical activity and gestational weight gain: results from the first baby study. Womens Health Issues. 2013;23(4): e233–8. doi:10.1016/j.whi.2013.04.004.<br>60. Mudd LM, Pivarnik J, Holzman CB, et al. Leisure-time physical activity in pregnancy and the birth weight distribution: where is the effect? J Phys Act Health. 2012;9(8):1168–77.<br>61. Jukic AMZ, Evenson KR, Daniels JL, et al. Prospective study of the association between vigorous physical activity during pregnancy and length of gestation and birthweight. Matern Child Health J. 2012;16(5):1031–44. doi:10.1007/s10995-011-0831-8.<br>62. Doustan M, Seifourian M, Zarghami M, et al. Relationship between physical activity of mothers before and during pregnancy with the newborn health and pregnancy outcome. J Phys Educ Sport. 2012;12(2):222–9.<br>63. Fortner RT, Pekow PS, Whitcomb BW, et al. Physical activity and hypertensive disorders of pregnancy among Hispanic women. Med Sci Sports Exerc. 2011;43(4):639–46. doi:10.1249/MSS.0b013e3181f58d3e.<br>64. Juhl M, Kogevinas M, Andersen PK, et al. Is swimming during pregnancy a safe exercise? Epidemiology. 2010;21(2):253–8. doi:10.1097/EDE.0b013e3181cb6267.<br>65. Juhl M, Olsen J, Andersen PK, et al. Physical exercise during pregnancy and fetal growth measures: a study within the Danish National Birth Cohort. Am J Obstet Gynecol. 2010;202(1): e1–8. doi:10.1016/j.ajog.2009.07.033.<br>66. Stuebe AM, Oken E, Gillman MW. Associations of diet and physical activity during pregnancy with risk for excessive gestational weight gain. Am J Obstet Gynecol. 2009;201(1): 58.e1–8. doi:10.1016/j.ajog.2009.02.025.<br>67. Tavares JS, Melo AS, Amorim MM, et al. Association between maternal physical activity, gestational weight gain and birth weight in a cohort of 118 pregnant women in Campina Grande, Northeast of Brazil. Rev Assoc Med Bras. 2009;55(3):335–41.<br>68. Osterdal ML, Strom M, Klemmensen AK, et al. Does leisure time physical activity in early pregnancy protect against preeclampsia? Prospective cohort in Danish women. BJOG. 2009;116(1):98–107. doi:10.1111/j.1471-0528.2008.02001.x.<br>69. Rudra CB, Sorensen TK, Luthy DA, et al. A prospective analysis of recreational physical activity and preeclampsia risk. MedSci Sports Exerc. 2008;40(9):1581–8. doi:10.1249/MSS. 0b013e31817cab1.<br>70. Magnus P, Trogstad L, Owe KM, et al. Recreational physical activity and the risk of preeclampsia: a prospective cohort of Norwegian women. Am J Epidemiol. 2008;168(8):952–7. doi:10.1093/aje/kwn189.<br>71. Juhl M, Andersen PK, Olsen J, et al. Physical exercise during pregnancy and the risk of preterm birth: a study within the Danish national birth cohort. Am J Epidemiol. 2008;167(7): 859–66. doi:10.1093/aje/kwm364.<br>72. Dwarkanath P, Muthayya S, Vaz M, et al. The relationship between maternal physical activity during pregnancy and birth weight. Asia Pac J Clin Nutr. 2007;16(4):704–10.<br>73. Downs DS, Hausenblas HA. Pregnant women's third trimester exercise behaviors, body mass index, and pregnancy outcomes. Psychol Health. 2007;22(5):545–59.<br>74. Watson PE, McDonald BW. Activity levels in pregnant New Zealand women: relationship with socioeconomic factors, wellbeing, anthropometric measures, and birth outcome. Appl Physiol Nutr Metab. 2007;32(4):733–42.<br>75. Oken E, Ning Y, Rifas-Shiman SL, et al. Associations of physical activity and inactivity before and during pregnancy with glucose tolerance. Obstet Gynecol. 2006;108(5):1200–7. 110.<br>76. Duncombe D, Skouteris H, Wertheim EH, et al. Vigorous exercise and birth outcomes in a sample of recreational exercisers: a prospective study across pregnancy. Aust N Z J Obstet Gynaecol. 2006;46(4):288–92.<br>77. Takito MY, Benicio MH, Latorre MR. Maternal posture and its influence on birthweight. Rev Saude Publica. 2005;39(3): 325–32.<br>78. Dempsey JC, Sorensen TK, Williams MA, et al. Prospective study of gestational diabetes mellitus risk in relation to maternal recreational physical activity before and during pregnancy. Am J Epidemiol. 2004;159(7):663–70.<br>79. Magann EF, Evans SF, Weitz B, et al. Antepartum, intrapartum, and neonatal significance of exercise on healthy low-risk pregnant working women. Obstet Gynecol. 2002;99(3):466–72.<br>80. Nieuwenhuijsen MJ, Northstone K, Golding J. Swimming and birth weight. Epidemiology. 2002;13(6):725–8.<br>81. Hatch M, Levin B. Maternal leisure-time exercise and timely delivery. Am J Public Health. 1998;88(10):1528–33.

Review	References of included behavioural interventions delivered in pregnancy
Di Mascio *et al.* 2016 [29]	1. Carpenter MW, Sady SP, Sady MA, Haydon BB, Coustah DR, Thompson PD. Effects of exercise training in midpregnancy: a randomized controlled trial. Presented at the 37th annual meeting of the Society for Gynecologic Investigation, St Louis. Missouri, March 21-24, 1990;Abstr.497, p. 345.<br>2. Prevedel T, Calderon I, DeConti M, Consonni E, Rudge M. Maternal and perinatal effects of hydrotherapy in pregnancy. Revista Brasil Ginecol Obstet 2003;25:53-9.<br>3. Barakat R, Stirling JR, Lucia A. Does exercise training during pregnancy affect gestational age? A randomized controlled trial. Br J Sports Med 2008;42:674-8.<br>4. Calvalcante SR, Cecatti JG, Pereira RI, Baciuk EP, Bernardo AL, Silveira C. Water aerobics II: maternal body composition and perinatal outcomes after a program for low risk pregnant women. Reprod Health 2009;6:1.<br>5. Haakstad LAH, Bo K. Exercise in pregnant women and birth weight: a randomized controlled trial. BMC Pregnancy Childbirth 2011;30;11:66.<br>6. Ruiz JR, Perales M, Pelaez M, Lopez C, Lucia A, Barakat R. Supervised exercise-based intervention to prevent gestational weight gain: a randomized controlled trial. Mayo Clin Proc 2013;88:1388-97.<br>7. Barakat R, Perales M, Bacchi M, Coteron J, Refoyo I. A program of exercise throughout pregnancy. Is it safe to mother and newborn? Am J Health Promot 2014;29:2-8.<br>8. Barakat R, Pelaez M, Montejo R, Refoyo I, Coteron J. Exercise throughout pregnancy does not cause preterm delivery: a randomized, controlled trial. J Phys Act Health 2014;11: 1012-7.<br>9. Barakat R, Pelaez M, Cordero Y, et al. Exercise during pregnancy protects against hypertension and macrosomia: randomized clinical trial. Am J Obstet Gynecol 2016;214: 649.e1-8.
IWiP 2017 [30]	1. Althuizen E, van der Wijden CL, van Mechelen W, Seidell JC, van Poppel MN. The effect of a counselling intervention on weight changes during and after pregnancy: a randomized trial. BJOG 2013;120:92-9.<br>2. Baciuk EP, Pereira RI, Cecatti JG, Braga AF, Cavalcante SR. Water aerobics in pregnancy: Cardiovascular response, labor and neonatal outcomes. Reprod Health 2008;5:10.<br>3. Barakat R, Stirling JR, Lucia A. Does exercise training during pregnancy affect gestational age? A randomised controlled trial. Br J Sports Med 2008;42:674-8.<br>4. Barakat R, Pelaez M, Montejo R, Luaces M, Zakynthinaki M. Exercise during pregnancy improves maternal health perception: a randomized controlled trial. Am J Obstet Gynecol 2011;204:402.e1-7.<br>5. Barakat R, Pelaez M, Lopez C, Montejo R, Coteron J. Exercise during pregnancy reduces the rate of cesarean and instrumental deliveries: results of a randomized controlled trial. J Matern Fetal Neonatal Med 2012;25:2372-6.<br>6. Bogaerts AF, Devlieger R, Nuyts E, Witters I, Gyselaers W, Van den Bergh BR. Effects of lifestyle intervention in obese pregnant women on gestational weight gain and mental health: a randomized controlled trial. Int J Obes (Lond) 2013;37:814-21.<br>7. Dodd JM, Turnbull D, McPhee AJ, et al. LIMIT Randomised Trial Group. Antenatal lifestyle advice for women who are overweight or obese: LIMIT randomised trial. BMJ 2014;348:g1285.<br>8. El Beltagy N, Saad El Deen S, Mohamed R. Does physical activity and diet control reduce the risk of developing gestational diabetes mellitus in Egypt? A randomized controlled trial. J Perinat Med 2013:41.<br>9. Guelinckx I, Devlieger R, Mullie P, Vansant G. Effect of lifestyle intervention on dietary habits, physical activity, and gestational weight gain in obese pregnant women: a randomized controlled trial. Am J Clin Nutr 2010;91:373-80.<br>10. Haakstad LA, Bø K. Effect of regular exercise on prevention of excessive weight gain in pregnancy: a randomised controlled trial. Eur J Contracept Reprod Health Care 2011;16:116-25.<br>11. Harrison CL, Lombard CB, Strauss BJ, Teede HJ. Optimizing healthy gestational weight gain in women at high risk of gestational diabetes: a randomized controlled trial. Obesity (Silver Spring) 2013;21:904-9.<br>12. Hui A, Back L, Ludwig S, et al. Lifestyle intervention on diet and exercise reduced excessive gestational weight gain in pregnant women under a randomised controlled trial. BJOG 2012;119:70-7.<br>13. Jeffries K, Shub A, Walker SP, Hiscock R, Permezel M. Reducing excessive weight gain in pregnancy: a randomised controlled trial. Med J Aust 2009;191:429-33.pmid:19835535.<br>14. Khaledan A, Motahari Tabari N, Ahmad Shirvani M. Effect of an Aerobic Exercise Program on Fetal Growth in Pregnant Women. HAYAT 2010;16:55-64. http://hayat.tums.ac.ir/ browse.php?a_id=98&sid=1&slc_lang=en<br>15. Khoury J, Henriksen T, Christophersen B, Tonstad S. Effect of a cholesterol-lowering diet on maternal, cord, and neonatal lipids, and pregnancy outcome: a randomized clinical trial. Am J Obstet Gynecol 2005;193:1292-301.<br>16. Luoto R, Kinnunen TI, Aittasalo M, et al. Primary prevention of gestational diabetes mellitus and large-for-gestational-age newborns by lifestyle counseling: a cluster-randomized controlled trial. PLoS Med 2011;8:e1001036.<br>17. Nascimento SL, Surita FG, Parpinelli MÂ, Siani S, Pinto e Silva JL. The effect of an antenatal physical exercise programme on maternal/perinatal outcomes and quality of life in overweight and obese pregnant women: a randomised clinical trial. BJOG 2011;118:1455-63.<br>18. Ong MJ, Guelfi KJ, Hunter T, Wallman KE, Fournier PA, Newnham JP. Supervised home-based exercise may attenuate the decline of glucose tolerance in obese pregnant women. Diabetes Metab 2009;35:418-21.<br>19. Oostdam N, van Poppel MNM, Wouters MGAJ, et al. No effect of the FitFor2 exercise programme on blood glucose, insulin sensitivity, and birthweight in pregnant women who were overweight and at risk for gestational diabetes: results of a randomised controlled trial. BJOG 2012;119:1098-107.<br>20. Perales M, Refoyo I, Coteron J, Bacchi M, Barakat R. Exercise during pregnancy attenuates prenatal depression: a randomized controlled trial. Eval Health Prof 2015;38:59-72.<br>21. Perales M, Calabria I, Lopez C, Franco E, Coteron J, Barakat R. Regular Exercise Throughout Pregnancy Is Associated With a Shorter First Stage of Labor. Am J Health Promot 2016;30:149-54.<br>22. Petrella E, Malavolti M, Bertarini V, et al. Gestational weight gain in overweight and obese women enrolled in a healthy lifestyle and eating habits program. J Matern Fetal Neonatal Med 2014;27:1348-52.<br>23. Phelan S, Phipps MG, Abrams B, Darroch F, Schaffner A, Wing RR. Randomized trial of a behavioral intervention to prevent excessive gestational weight gain: the Fit for Delivery Study. Am J Clin Nutr 2011;93:772-9.<br>24. Prevedel TCI, DeConti M, Adami HO, et al. Maternal and perinatal effects of hydrotherapy in pregnancy. Rev Bras Ginecol Obstet 2003;25:53-9.<br>25. Poston L, Bell R, Croker H, et al. UPBEAT Trial Consortium. Effect of a behavioural intervention in obese pregnant women (the UPBEAT study): a multicentre, randomized controlled trial. Lancet Diabetes Endocrinol 2015;3:767-77.

Review	References of included behavioural interventions delivered in pregnancy
	26. Rauh K, Gabriel E, Kerschbaum E, et al. Safety and efficacy of a lifestyle intervention for pregnant women to prevent excessive maternal weight gain: a cluster-randomized controlled trial. BMC Pregnancy Childbirth 2013;13:151.<br>27. Renault KM, Nørgaard K, Nilas L, et al. The Treatment of Obese Pregnant Women (TOP) study: a randomized controlled trial of the effect of physical activity intervention assessed by pedometer with or without dietary intervention in obese pregnant women. Am J Obstet Gynecol 2014;210:134.e1-9.<br>28. Ruiz JR, Perales M, Pelaez M, Lopez C, Lucia A, Barakat R. Supervised exercise-based intervention to prevent excessive gestational weight gain: a randomized controlled trial. Mayo Clin Proc 2013;88:1388-97.<br>29. Stafne SN, Salvesen KA, Romundstad PR, Torjusen IH, Mørkved S. Does regular exercise including pelvic floor muscle training prevent urinary and anal incontinence during pregnancy? A randomised controlled trial. BJOG 2012;119:1270-80.<br>30. Sagedal LR, Øverby NC, Bere E, et al. Lifestyle intervention to limit gestational weight gain: the Norwegian Fit for Delivery randomised controlled trial. BJOG 2017;124:97-109.<br>31. Walsh JM, McGowan CA, Mahony R, Foley ME, McAuliffe FM. Low glycaemic index diet in pregnancy to prevent macrosomia (ROLO study): randomised control trial. BMJ 2012;345:e5605.<br>32. Wolff S, Legarth J, Vangsgaard K, Toubro S, Astrup A. A randomized trial of the effects of dietary counseling on gestational weight gain and glucose metabolism in obese pregnant women. Int J Obes (Lond) 2008;32:495-501.<br>33. Vinter CA, Jensen DM, Ovesen P, Beck-Nielsen H, Jørgensen JS. The LiP (Lifestyle in Pregnancy) study: a randomized controlled trial of lifestyle intervention in 360 obese pregnant women. Diabetes Care 2011;34:2502-7.<br>34. Vítolo MR, Bueno MS, Gama CM. [Impact of a dietary counseling program on the gain weight speed of pregnant women attended in a primary care service]. Rev Bras Ginecol Obstet 2011;33:13-9.pmid:21625788.<br>35. Yeo S, Neelon V, Weaver M, et al. Regular exercise from 12-22 weeks of pregnancy in women at risk for preeclampsia: A feasibility study. (unpublished).<br>36. Yeo S, Steele NM, Chang MC, Leclaire SM, Ronis
Lau *et al.* 2017 [31]	1. Albright CL, Steffen AD, Wilkens LR et al. Effectiveness of a 12-month randomized clinical trial to increase physical activity in multiethnic postpartum women: results from Hawaii's Na Mikimiki Project. Prev Med 2014; 69: 214–223.<br>2. Bertz F, Brekke HK, Ellegård L, Rasmussen KM, Wennergren M, Winkvist A. Diet and exercise weight-loss trial in lactating overweight and obese women. Am J Clin Nutr 2012; 96: 698–705<br>3. Bertz F, Winkvist A, Brekke HK. Sustainable weight loss among overweight and obese lactating women is achieved with an energy-reduced diet in line with dietary recommendations: results from the LEVA randomized controlled trial. J Acad Nutr Diet 2015; 115: 78–8<br>4. Choi J, Lee J, Vittinghoff E, Fukuoka Y. mHealth Physical Activity Intervention: a randomized pilot study in physically inactive pregnant women. Matern Child Health J 2016; 20: 1091–1101.<br>5. Colleran HL, Lovelady CA. Use of mypyramid menu planner for moms in a weight-loss intervention during lactation. J Acad Nutr Diet 2012; 112: 553–558<br>6. Fjeldsoe BS, Miller YD, Marshall AL. MobileMums: a randomized controlled trial of an SMS-based physical activity intervention. Ann Behav Med 2010; 39: 101–111.<br>7. Harrison CL, Lombard CB, Strauss BJ, Teede HJ. Optimizing healthy gestational weight gain in women at high risk of gestational diabetes: a randomized controlled trial. Obesity 2013; 21: 904–909.<br>8. Harrison CL, Lombard CB, Teede HJ. Limiting postpartum weight retention through early antenatal intervention: the HeLPher randomised controlled trial. Int J Behav Nutr Phys Act 2014; 11: 1–8.<br>9. Herring SJ, Cruice JF, Bennett GG, Davey A, Foster GD. Using technology to promote postpartum weight loss in urban, low income mothers: a pilot randomized controlled trial. J Nutr Educ Behav 2014; 46: 610–615.<br>10. Herring SJ, Cruice JF, Bennett GG, Rose MZ, Davey A, Foster GD. Preventing excessive gestational weight gain among African American women: a randomized clinical trial. Obesity 2016; 24: 30–36.<br>11. Hui AL, Ludwig SM, Gardiner P et al. Community-based exercise and dietary intervention during pregnancy: a pilot study. Can J Diabetes 2006; 30: 169–175.<br>12. Jackson RA, Stotland NE, Caughey AB, Gerbert B. Improving diet and exercise in pregnancy with video doctor counseling: a randomized trial. Patient Educ Couns 2011; 83: 203–209.<br>13. Phelan S, Phipps MG, Abrams B, Darroch F, Schaffner A, Wing RR. Randomized trial of a behavioral intervention to prevent excessive gestational weight gain: the Fit for delivery study. Am J Clin Nutr 2011; 93: 772–779.<br>14. Phelan S, Phipps MG, Abrams B et al. Does behavioural intervention in pregnancy reduce postpartum weight retention? Twelve-month outcomes of the fit for delivery randomized trial. Am J Clin Nutr 2014; 99: 302–311.<br>15. Pollak KI, Alexander SC, Bennett G et al. Weight-related SMS texts promoting appropriate pregnancy weight gain: a pilot study. Patient Educ Couns 2014; 97: 256–260<br>16. Poston L, Bell R, Croker H et al. Effect of a behavioural intervention in obese pregnant women (the UPBEAT study): a multicentre, randomised controlled trial. Lancet Diabetes Endocrinol 2015; 3: 767–777.<br>17. Smith KM. The Blossom Project Online: use of a behaviorally based website to promote physical activity and prevent excessive gestational weight gain in previously sedentary pregnant women. Graduate Theses and Dissertations 2014: 14051
Magro-Malosso *et al* 2017a [32]	1. Barakat R, Ruiz JR, Stirling JR, Zakynthinaki M, Lucia A. Type of delivery is not affected by light resistance and toning exercise training during pregnancy: a randomized controlled trial. Am J Obstet Gynecol. 2009;201:590.e1–6.<br>2. Barakat R, Pelaez M, Montejo R, Luaces M, Zakynthinaki M. Exercise during pregnancy improves maternal health perception: a randomized controlled trial. Am J Obstet Gynecol. 2011;204:402.e1–7.<br>3. Barakat R, Pelaez M, Lopez C, Montejo R, Coteron J. Exercise during pregnancy reduces the rate of cesarean and instrumental deliveries: results of a randomized controlled trial. J Matern Fetal Neonatal Med. 2012;25:2372–6.<br>4. Barakat R, Cordero Y, Coteron J, Luaces M, Montejo R. Exercise during pregnancy improves maternal glucose screen at 24–28 weeks: a randomised controlled trial. Br J Sports Med. 2012;46:656–61.<br>5. Barakat R, Perales M, Bacchi M, Coteron J, Refoyo I. A program of exercise throughout pregnancy. Is it safe to mother and newborn? Am J Health Promot. 2014;29:2–8.<br>6. Barakat R, Pelaez M, Montejo R, Refoyo I, Coteron J. Exercise throughout pregnancy does not cause preterm delivery: a randomized, controlled trial. J Phys Act Health. 2014;11:1012–7.

Review	References of included behavioural interventions delivered in pregnancy
	7. Barakat R, Pelaez M, Cordero Y, Perales M, Lopez C, Coteron J, et al. Exercise during pregnancy protects against hypertension and macrosomia: randomized clinical trial. Am J Obstet Gynecol. 2016;214:649.e1–8.<br>8. de Oliveria Melo AS, Silva JL, Tavares JS, Barros VO, Leite DF, Amorim MM. Effect of a physical exercise program during pregnancy on uteroplacental and fetal blood flow and fetal growth: a randomized controlled trial. Obstet Gynecol. 2012;120:302–10.<br>9. Haakstad LAH, Bo K. Exercise in pregnant women and birth weight: a randomized controlled trial. BMC Pregnancy Childbirth. 2011;30:66.<br>10. Kong KL, Campbell CG, Foster RC, Peterson AD, Lanningham-Foster L. A pilot walking program promotes moderate-intensity physical activity during pregnancy. Med.Sci Sports Exerc. 2014;46:462–71.<br>11. Perales M, Santos-Lozano A, Sanchis-Gomar F, Luaces M, Pareja-Galeano H, Garatachea N, et al. Maternal cardiac adaptations to a physical exercise program during pregnancy. Med Sci Sports Exerc. 2016;48:896– 906.<br>12. Petrella E, Malavolti M, Bertarini V, Pignatti L, Neri I, Battistini NC, et al. Gestational weight gain in overweight and obese women enrolled in a healthy lifestyle and eating habits program. J Matern Fetal Neonatal Med. 2014;27:1348–52.<br>13. Price B, Amini SB, Kappler K. Exercise in pregnancy: effect of fitness and obstetric outcomes – a randomized controlled trial. Med Sci Sports Exerc. 2012;44:2263–9.<br>14. Renault KM, Nørgaard K, Nilas L, Carlsen EM, Cortes D, Pryds O, et al. The Treatment of Obese Pregnant Women (TOP) study: a randomized controlled trial of the effect of physical activity intervention assessed by pedometer with or without dietary intervention in obese pregnant women. Am J Obstet Gynecol. 2014;210:134.e1–9.<br>15. Ruiz JR, Perales M, Pelaez M, Lopez C, Lucia A, Barakat R. Supervised exercise-based intervention to prevent gestational weight gain: a randomized controlled trial. Mayo Clin Proc. 2013;88:1388–97.<br>16. Stafne SN, Salvesen K A, Romundstad PR, Eggebø TM, Carlsen SM, Mørkved S. Regular exercise during pregnancy to prevent gestational diabetes: a randomized controlled trial. Obstet Gynecol. 2012;119:29–36.<br>17. Vinter CA, Jensen DM, Ovesen P, Beck-Nielsen H, Jørgensen JS. The LiP (Lifestyle in Pregnancy) study: a randomized controlled trial of lifestyle intervention in 360 obese pregnant women. Diabetes Care. 2011;34:2502–7.
Magro-Malosso *et al* 2017b [33]	1. Barakat R, Pelaez M, Cordero Y, Perales M, Lopez C, Coteron J, et al. Exercise during pregnancy protects against hypertension and macrosomia: randomized clinical trial. Am J Obstet Gynecol. 2016;214:649.e1-8.<br>2. Kong KL, Campbell CG, Foster RC, Peterson AD, Lanningham-Foster L. A pilot walking program promotes moderate-intensity physical activity during pregnancy. Med Sci Sports Exerc. 2014;46:462–71.<br>3. Nascimento SL, Surita FG, Parpinelli M^A, Siani S, Pinto e Silva JL. The effect of an antenatal physical exercise programme on maternal/perinatal outcomes and quality of life in overweight and obese pregnant women: a randomised clinical trial. BJOG. 2011;118:1455–63.<br>4. Petrella E, Malavolti M, Bertarini V, Pignatti L, Neri I, Battistini NC, et al. Gestational weight gain in overweight and obese women enrolled in a healthy lifestyle and eating habits program. J Matern Fetal Neonatal Med. 2014;27:1348–52.<br>5. Price B, Amini SB, Kappler K. Exercise in pregnancy: effect of fitness and obstetric outcomes – a randomized controlled trial. Med Sci Sports Exerc. 2012;44:2263–9.<br>6. Renault KM, Nørgaard K, Nilas L, Carlsen EM, Cortes D, Pryds O, et al. The Treatment of Obese Pregnant Women (TOP) study: a randomized controlled trial of the effect of physical activity intervention assessed by pedometer with or without dietary intervention in obese pregnant women. Am J Obstet Gynecol. 2014;210:134.e1-9.<br>7. Ruiz JR, Perales M, Pelaez M, Lopez C, Lucia A, Barakat R. Supervised exercise-based intervention to prevent gestational weight gain: a randomized controlled trial. Mayo Clin Proc. 2013;88:1388–97.<br>8. Vinter CA, Jensen DM, Ovesen P, Beck-Nielsen H, Jørgensen JS. The LiP (Lifestyle in Pregnancy) study: a randomized controlled trial of lifestyle intervention in obese pregnant women. Diabetes Care. 2011;34:2502–7.<br>9. Santos IA, Stein R, Fuchs SC, Duncan BB, Ribeiro JP, Kroeff LR, et al. Aerobic exercise and submaximal functional capacity in overweight pregnant women: a randomized trial. Obstet Gynecol. 2005;106:243–9.
Sanabria-Martínez *et al.* 2016 [34]	1. Ruiz J, Perales M, Pelaez M, et al. Supervised exercise-based intervention to prevent excessive gestational weight gain: a ran- domized controlled trial. Mayo Clin Proc. 2013;88:1388-1397.<br>2. Tomic V, Sporis G, Tomic J, et al. The effect of maternal exercise during pregnancy on abnormal fetal growth. Croat Med J. 2013; 54:362-368.<br>3. Barakat R, Perales M, Bacchi M, et al. A program of exercise throughout pregnancy. Is it safe to mother and newborn? Am J Health Promot. 2014;29:2-8.<br>4. Barakat R, Pelaez M, Lopez C, et al. Exercise during pregnancy and gestational diabetes-related adverse effects: a randomized controlled trial. Br J Sports Med. 2013;47:630-636.<br>5. Barakat R, Pelaez M, Montejo R, et al. Exercise throughout preg- nancy does not cause preterm delivery. A randomized controlled trial. J Phys Act Health. 2014;11:1012-1017.<br>6. de Oliveria AS, Silva JLP, Tavares JS, et al. Effect of a physical exercise program during pregnancy on uteroplacental and fetal blood flow and fetal growth. Obstet Gynecol. 2012;120:302-310.<br>7. Barakat R, Cordero Y, Coteron J, et al. Exercise during pregnancy improves maternal glucose screen at 24-28 weeks: a randomized controlled trial. Br J Sports Med. 2012;46:656-661.<br>8. Price BB, Saeid B, Kappeper K. Exercise in pregnancy: effect on fitness and obstetric outcomes—a randomized trial. Med Sci Sports Exerc. 2012;44:2263-2269.<br>9. Barakat R, Pelaez M, Montejo R, et al. Exercise during pregnancy improves maternal health perception: a randomized controlled trial. Am J Obstet Gynecol. 2011;204:402.e1-402.e7.<br>10. Haakstad LA, Bø K. Exercise in pregnant women and birth weight: a randomized controlled trial. BMC Pregnancy Child- birth. 2011;11:66.<br>11. Hopkins S, Baldi JC, Cutfield WS, et al. Effects of exercise train- ing on maternal hormonal changes in pregnancy. Clin Endocrinol. 2011;74:495-500.<br>12. Barakat R, Ruiz JR, Stirling JR, et al. Type of delivery is not affected by light resistance and toning exercise training during pregnancy: a randomized controlled trial. Am J Obstet Gynecol. 2009;201:590.e1-590.e6.<br>13. Cavalcante SR, Cecatti JG, Pereira RI, et al. Water aerobics II: maternal body composition and perinatal outcomes after a pro- gram for low risk pregnant women. Reprod Health. 2009;6:1.<br>14. Clapp JF, Kim H, Burciu B, Lo´pez B. Beginning regular exercise in early pregnancy: effect on fetoplacental growth. Am J Obstet Gynecol. 2000;183:1484-1488.
Shepherd *et al.* 2017 [35]	1. Asbee SM, Jenkins TR, Butler JR, White J, Elliot M, Rutledge A. Dietary counseling prevents excessive weight gain during pregnancy, a randomized controlled trial. Obstetrics and Gynecology 2008;111(4 Suppl):6S.?

Review	References of included behavioural interventions delivered in pregnancy
	2. Asbee SM, Jenkins TR, Butler JR, White J, Elliot M, Rutledge A. Preventing excessive weight gain during pregnancy through dietary and lifestyle counseling: a randomized controlled trial. Obstetrics and Gynecology 2009;113(2 Pt 1):305–12.?NCT00792480. Does dietary and lifestyle counseling prevent excessive weight gain during pregnancy? A randomized controlled trial (WIP). https://clinicaltrials.gov/ ct2/show/NCT00792480 (first received 14 November 2008).<br>3. Bruno R, Petrella E, Bertarini V, Pedrielli G, Neri I, Facchinetti F. Adherence to a lifestyle programme in overweight/obese pregnant women and effect on gestational diabetes mellitus: a randomized controlled trial. Maternal and Child Nutrition 2016 Sep 19 [Epub ahead of print]. [DOI: 10.1111/mcn.12333?NCT01783210. Pregnancy complications in women with BMI > 25kg/m2 enrolled in a healthy lifestyle and eating habits program. clinicaltrials.gov/ct2/show/NCT01783210 (first received 28 January 2013).<br>4. Dodd 2014 Limiting weight gain in overweight and obese women during pregnancy to improve health outcomes: a randomised trial. anzctr.org.au/Trial/ Registration/TrialReview.aspx?id=81642 (first received 12 October 2006).?<br>5. Cramp CS, Moran LJ, Deussen AR, Yelland LN, Dodd JM. Evaluation of printed nutrition education material in overweight and obese women during pregnancy-findings from the LIMIT randomised trial. Journal of Paediatrics and Child Health 2013;49 Suppl 2:118.?<br>6. Dodd J. Obesity in pregnancy-the LIMIT randomised trial. Journal of Paediatrics and Child Health 2013;49 Suppl 2:4. Dodd JM. Dietary and lifestyle advice for pregnant women who are overweight or obese: the LIMIT randomized trial. Annals of Nutrition and Metabolism 2014;64(3-4):197–202.<br>7. Dodd JM, Cramp C, Sui Z, Yelland LN, Deussen AR, Grivell RM, et al. The effects of antenatal dietary and lifestyle advice for women who are overweight or obese on maternal diet and physical activity: the LIMIT randomised trial. BMC Medicine 2014;12(1):161.?<br>8. Dodd JM, Deussen AR, Mohamad I, Rifas-Shiman SL, Yelland LN, Louise J, et al. The effect of antenatal lifestyle advice for women who are overweight or obese on secondary measures of neonatal body composition: the LIMIT randomised trial. BJOG: an international journal of obstetrics and gynaecology 2016;123(2):244–53.<br>9. Dodd JM, Kannieappan LM, Grivell RM, Deussen AR, Moran LJ, Yelland LN, et al. Effects of an antenatal dietary intervention on maternal anthropometric measures in pregnant women with obesity. Obesity 2015;23(8): 1555–62.<br>10. Dodd JM, McPhee AJ, Turnbull D, Yelland LN, Deussen AR, Grivell RM, et al. The effects of antenatal dietary and lifestyle advice for women who are overweight or obese on neonatal health outcomes: the LIMIT randomised trial. BMC Medicine 2014;12(1):163.<br>11. Dodd JM, Newman A, Moran LJ, Deussen AR, Grivell RM, Yelland LN, et al. The effect of antenatal dietary and lifestyle advice for women who are overweight or obese on emotional well-being: the LIMIT randomized trial. Acta Obstetricia et Gynecologica Scandinavica 2016;95(3): 309–18.<br>12. Dodd JM, O'Brien CM, Grivell RM. Modifying diet and physical activity to support pregnant women who are overweight or obese. Current Opinion in Clinical Nutrition and Metabolic Care 2015;18(3):318–23.<br>13. Dodd JM, Turnbull D, McPhee AJ, Deussen AR, Grivell RM, Yelland LN, et al. Antenatal lifestyle advice for women who are overweight or obese: LIMIT randomised trial. BMJ 2014;348:g1285.<br>14. Dodd JM, Turnbull D, McPhee AJ, Deussen AR, Grivell RM, Yelland LN, et al. Dietary and physical activity interventions for women who are overweight or obese during pregnancy - the findings of the LIMIT randomized trial. The Power of Programming 2014: International Conference on Developmental Origins of Adiposity and Long-Term Health; 2014 March 13-15; Munich, women on maternal and infant outcomes. Journal of Paediatrics and Child Health 2013;49 Suppl 2:19.?<br>15. Grivell R, Yelland L, Earl RA, Staehr CJ, Dodd J. The effect of antenatal dietary and lifestyle advice on fetal body composition in women who are overweight or obese: findings from the LIMIT randomised trial. Ultrasound in Obstetrics and Gynecology 2013;42(Suppl 1):10.<br>16. Grivell R, Yelland L, Staehr CJ, Earl RA, Dodd J. The effect of antenatal dietary and lifestyle advice on fetal growth?in women who are overweight or obese: findings from?the LIMIT randomised trial. Ultrasound in Obstetrics and Gynecology 2013;42(Suppl 1):83.<br>17. Grivell RM, Yelland L, Staehr C, Earl RA, Dodd JM. The effect of antenatal dietary and lifestyle advice on fetal body composition in women who are overweight or obese: Findings from the LIMIT randomised trial. The Power of Programming 2014: International Conference on Developmental Origins of Adiposity and Long-Term Health; 2014 March 13-15; Munich, Germany. 2014: 56–7.<br>18. Grivell RM, Yelland LN, Deussen A, Crowther CA, Dodd JM. Antenatal dietary and lifestyle advice for women who are overweight or obese and the effect on fetal growth and adiposity: the LIMIT randomised trial. BJOG: an international journal of obstetrics & gynaecology 2016;123 (2):233–43.<br>19. Kannieappan LM, Deussen AR, Moran LJ, Grivell RM, Yelland LN, Dodd JM. The effect of antenatal dietary advice on maternal body composition in women who are overweight or obese - findings from the LIMIT randomised trial. Journal of Paediatrics and Child Health 2013;49 Suppl 2:94.<br>20. Newman AK, Deussen AR, Moran LJ, Grivell RM, Yelland LN, Turnbull D, et al. The effect of antenatal dietary and lifestyle advice on maternal psychological health in women who are overweight or obese-findings from the LIMIT randomised trial. Journal of Paediatrics and Child Health 2013;49 Suppl 2:119.<br>21. Sui Z, Yelland LN, Turnbull D, Dodd JM. Walking to limit gestational weight gain and keep fit during pregnancy - findings from the walk randomised trial. Journal of Paediatrics and Child Health 2013;49 Suppl 2:120.?<br>22. Szmeja MA, Cramp C, Grivell RM, Deussen AR, Yelland LN, Dodd JM. Use of a DVD to provide dietary and lifestyle information to pregnant women who are overweight or obese: a nested randomised trial. BMC Pregnancy and Childbirth 2014;14(1):409.?<br>23. Szmeja MA, Grivell RM, Deussen AR, Dodd JM. Evaluation of information provision to women who are overweight or obese during pregnancy. Journal of Paediatrics Germany. 2014:22–3.<br>24. Dodd JM, Turnbull DA, McPhee AJ, Wittert G, Crowther CA, Robinson JS. Limiting weight gain in overweight and obese women during pregnancy to improve health outcomes: the LIMIT randomised controlled trial. BMC Pregnancy and Childbirth 2011;11:79.

Review	References of included behavioural interventions delivered in pregnancy
	25. Eames AJ, Grivell RM, Dodd JM, Deussen A. The effect of limited gestational weight gain in overweight and obese<br>26. El Beltagy N, El Deen SS, Mohamed R. Does physical activity and diet control reduce the risk of developing gestational diabetes mellitus in Egypt? A randomized controlled trial. Journal of Perinatal Medicine 2013;41 (Suppl 1):1176.<br>27. Harrison 2013 ACTRN12608000233325. Healthier lifestyles: preventing gestational diabetes in high risk pregnancies: a research and education project. anzctr.org.au/Trial/Registration/ TrialReview.aspx?ACTRN=12608000233325 (first received 5 May 2008).?<br>28. Harrison CL, Lombard CB, Gibson-Helm M, Deeks?A, Teede HJ. Limiting excess weight gain in high-risk pregnancies: A randomized controlled trial. Endocrine Reviews 2011;32(3 Meeting Abstracts):P1–466.?<br>29. Harrison CL, Lombard CB, Strauss BJ, Teede HJ. Optimizing healthy gestational weight gain in women at high risk of gestational diabetes: a randomized controlled trial. Obesity 2013;21(5):904–9.?<br>30. Harrison CL, Lombard CB, Teede HJ. Limiting postpartum weight retention through early antenatal intervention: the HeLP-her randomised controlled trial. International Journal of Behavioral Nutrition and Physical Activity 2014;11(1): 134.?<br>31. Harrison CL, Teede HJ, Lombard CB. How effective is self- weighing in the setting of a lifestyle intervention to reduce gestational weight gain and postpartum weight retention??. Australian and New Zealand Journal of Obstetrics and Gynaecology 2014;54:382–5.?<br>32. Lombard C, Harrison C, Teede H. A randomized controlled trial investigating self-weighing and the prevention of excess weight gain in early pregnancy. Endocrine Reviews 2011;32 (3 Meeting Abstracts):P2–768.?<br>33. Teede HJ, Harrison CL, Gibson-Helm M, Lombard CB. Improving physical activity in high-risk pregnancies: A randomized controlled trial. Endocrine Reviews 2011;32(3 Meeting Abstracts):P1–467.<br>34. Hawkins M, Hosker M, Marcus BH, Rosal MC, Braun B, Stanek EJ, et al. A pregnancy lifestyle intervention to prevent gestational diabetes risk factors in overweight Hispanic women: a feasibility randomized controlled trial. Diabetic Medicine 2014;32:108–15.?NCT01141582. A lifestyle intervention to prevent gestational diabetes. clinicaltrials.gov/archive/ NCT01141582/2010 06 09 (first received 2 August 2008).<br>35. Herring SJ, Cruice JF, Bennett GG, Rose MZ, Davey A, Foster GD. Preventing excessive gestational weight gain among African American women: a randomized clinical trial. Obesity 2016;24(1):30–6.?NCT01530776. Healthy4Baby: Preventing postpartum weight retention among low-income, black women. clinicaltrials.gov/ct2/show/NCT01530776 (first received 23 January 2012).<br>36. Hoirisch-Clapauch 2016 {published and unpublished data} Hoirisch-Clapauch S, Sant'Anna MCW, Moreira ECC, Frankel PP, Valle MP, D'Ippolito MM. A protocol combining daily walking and a low glycemic index diet increases the rate of take-home babies in women with consecutive first-trimester miscarriages. BJOG: an international journal of obstetrics and gynaecology 2016;123 (Suppl 2):74–5.<br>37. Hui A, Back L, Ludwig S, Gardiner P, Sevenhuysen G, Dean H, et al. Exercise and dietary intervention increases physical activity, promotes healthy diet and reduces excessive gestational weight gain in pregnant women: A randomized controlled trial in Urban community. Diabetes 2011;60: A351.?<br>38. Hui A, Back L, Ludwig S, Gardiner P, Sevenhuysen G, Dean H, et al. Lifestyle intervention on diet and exercise reduced excessive gestational weight gain in pregnant women under a randomised controlled trial. BJOG: an international journal of obstetrics and gynaecology 2012;119 (1):70–7.?<br>39. Hui AL, Ludwig S, Gardiner P, Sevenhuysen G, Dean H, Sellers E, et al. Exercise and dietary intervention during pregnancy results in reduced excessive gestational weight gain. Diabetes 2010;59(Suppl 1):A509.?<br>40. Hui AL, Ludwig SM, Gardiner P, Sevenhuysen G, Murray R, Morris M, et al. Community-based exercise and dietary intervention during pregnancy: a pilot study. Canadian Journal of Diabetes 2006;30(2):169–75.<br>41. Hui AL, Back L, Reid A, Sevenhuysen G, Ludwig S, Dean H, et al. Effects of physical activity and dietary intakes on weight gain of pregnant women with normal and above normal prepregnancy weight. Canadian Journal of Diabetes 2012;36(5 Suppl 1):S8.?<br>42. Hui AL, Ludwig S, Gardiner P, Sevenhuysen G, Dean HJ, Sellers E, et al. Effects of lifestyle intervention on dietary intake, physical activity level, and gestational weight gain in pregnant women with different pre-pregnancy Body Mass Index in a randomized control trial. BMC Pregnancy and Childbirth 2014;14(1):331.?NCT00486629. Impact of diet and exercise activity on pregnancy outcomes (IDEA). clinicaltrials.gov/ct2/show/ NCT00486629 (first received 12 June 2007).<br>43. Jing 2015 ChiCTR-IPR-15005809. The effect of a personalized intervention on weight gain and physical activity among pregnant women in China. chictr.org.cn/showprojen.aspx? proj=10240 (first received 12 January 2015).?<br>44. Jing W, Huang Y, Liu X, Luo B, Yang Y, Liao S. The effect of a personalized intervention on weight gain and physical activity among pregnant women in China. International Journal of Gynaecology and Obstetrics 2015;129(2):138–41.<br>45. Koivusalo 2016 {published data only}?Grotenfelt NE, Wasenius NS, Rono K, Laivuori H, Stach-Lempinen B, Orho-Melander M, et al. Interaction between rs10830963 polymorphism in mtnr1b and lifestyle intervention on occurrence of gestational diabetes. Diabetologia 2016;59(8):1655–8.?<br>46. Koivusalo SB, Rono K, Klemetti MM, Roine RP, Lindstrom J, Erkkola M, et al. Gestational diabetes mellitus can be prevented by lifestyle intervention: The Finnish gestational diabetes prevention study (RADIEL): A randomized controlled trial. Diabetes Care 2016;39:24–30. NCT01698385. Prevention of gestational diabetes through lifestyle modification (RADIEL) - a randomized controlled multi-centre intervention study. clinicaltrials.gov/ct2/show/ NCT01698385 (first received 11 September 2012).<br>47. Rono K, Stach-Lempinen B, Klemetti MM, Kaaja RJ, Poyhonen-Alho M, Eriksson JG, et al. Prevention of gestational diabetes through lifestyle intervention: study design and methods of a Finnish randomized controlled multicenter trial (RADIEL). BMC Pregnancy and Childbirth 2014;14:70.<br>48. Valkama A, Koivusalo S, Lindstrom J, Meinila J, Kautiainen H, Stach-Lempinen B, et al. The effect of dietary counselling on diet in pregnant women at risk for gestational diabetes. Annals of Nutrition and Metabolism 2015;67 (Suppl 1):138.<br>49. Valkama A, Koivusalo S, Lindstrom J, Meinila J, Kautiainen H, Stach-Lempinen B, et al. The effect of dietary counselling on food intakes in pregnant women at risk for gestational diabetes: a secondary analysis of a randomised controlled trial RADIEL. European Journal of Clinical Nutrition 2016;70(8):912–7.

Review	References of included behavioural interventions delivered in pregnancy
	50. Korpi-Hyovalti EA, Laaksonen DE, Schwab US, Vanhapiha TH, Vihla KR, Heinonen ST, et al. Feasibility of a lifestyle intervention in early pregnancy to prevent deterioration of glucose tolerance. BMC Public Health 2011;11:179.?NCT01130012. Prevention of gestational diabetes. clinicaltrials.gov/ct2/show/NCT01130012 (first received 11 May 2010).<br>51. Aittasalo M, Raitanen J, Kinnunen TI, Ojala K, Kolu P, Luoto R. Is intensive counseling in maternity care feasible and effective in promoting physical activity among women at risk for gestational diabetes? Secondary analysis of a cluster randomized NELLI study in Finland. International Journal of Behavioral Nutrition and Physical Activity 2012;9: 104.?ISRCTN33885819. Primary prevention of gestational diabetes among women at risk: a cluster-randomized controlled trial. isrctn.com/ISRCTN33885819 (first received 12 September 2007).?<br>52. Kinnunen TI, Puhkala J, Raitanen J, Ahonen S, Aittasalo M, Virtanen SM, et al. Effects of dietary counselling on food habits and dietary intake of Finnish pregnant women at increased risk for gestational diabetes - a secondary analysis of a cluster-randomized controlled trial. Maternal and Child Nutrition 2014;10(2):184–97.?<br>53. Kolu P, Raitanen J, Luoto R. Physical activity and health- related quality of life during pregnancy: a secondary analysis of a cluster-randomised trial. Maternal and Child Health Journal 2014;18(9):2098–105.?<br>54. Kolu P, Raitanen J, Rissanen P, Luoto R. Cost-effectiveness of lifestyle counselling as primary prevention of gestational diabetes mellitus: findings from a cluster-randomised trial. PLOS One 2013;8(2):e56392.<br>55. Kolu P, Raitanen J, Rissanen P, Luoto R. Health care costs associated with gestational diabetes mellitus among high-risk women - results from a randomised trial. BMC Pregnancy and Childbirth 2012;12:71.?<br>56. Leppanen M, Aittasalo M, Raitanen J, Kinnunen TI, Kujala UM, Luoto R. Physical activity during pregnancy: predictors of change, perceived support and barriers among women at increased risk of gestational diabetes. Maternal and Child Health Journal 2014;18(9):2158–66.?<br>57. Luoto R, Kinnunen TI, Aittasalo M, Kolu P, Raitanen J, Ojala K, et al. Primary prevention of gestational diabetes mellitus and large-for-gestational-age newborns by lifestyle counseling: a cluster-randomized controlled trial. PLOS Medicine 2011;8(5):1–11.?<br>58. Luoto RM, Kinnunen TI, Aittasalo M, Ojala K, Mansikkamaki K, Poropainen E, et al. Prevention of gestational diabetes: design of a cluster-randomized controlled trial and one-year follow-up. BMC Pregnancy and Childbirth 2010;10:39.?<br>59. Puhkala J, Luoto R, Ahotupa M, Raitanen J, Vasankari T. Postpartum weight retention is associated with elevated ratio of oxidized LDL lipids to HDL-cholesterol. Lipids 2013;48(12):1227–35.<br>60. Petrella E, Facchinetti F, Bertarini V, Pignatti L, Neri I, Battistini NC. Occurrence of pregnancy complications in women with BMI > 25 submitted to a healthy lifestyle and eating habits program. American Journal of Obstetrics and Gynecology 2013;208(1 Suppl):S33–4.?<br>61. Petrella E, Malavolti M, Bertarini V, Pignatti L, Neri I, Battistini NC, et al. Gestational weight gain in overweight and obese women enrolled in a healthy lifestyle and eating habits program. Journal of Maternal-Fetal and Neonatal Medicine 2014;27(13):1348–52.<br>62. Hagobian TA, Phelan S, Gorin AA, Phipps MG, Abrams B, Wing RR. Effects of maternal lifestyle intervention during pregnancy on untreated partner weight: results from fit for delivery study. Obesity 2016;24(1):23–5.?NCT01117961. Promoting healthy weight gain during pregnancy. clinicaltrials.gov/ct2/show/NCT01117961 (first received 15 April 2010).?<br>63. Phelan S, Phipps MG, Abrams B, Darroch F, Grantham?K, Schaffner A, et al. Does behavioral intervention in pregnancy reduce postpartum weight retention? Twelve- month outcomes of the Fit for Delivery randomized trial. American Journal of Clinical Nutrition 2014;99(2):302–12.<br>64. Phelan S, Phipps MG, Abrams B, Darroch F, Schaffner A, Wing RR. Factors associated with success in the ”fit for delivery“ intervention to reduce excessive gestational weight gain. Obesity 2011;19(Suppl 1):S95.?<br>65. Phelan S, Phipps MG, Abrams B, Darroch F, Schaffner A, Wing RR. Randomized trial of a behavioral intervention to prevent excessive gestational weight gain: the Fit for Delivery Study. American Journal of Clinical Nutrition 2011;93(4):772–9.?Phelan S, Phipps MG, Abrams B, Darroch F, Schafner A, Wing RR. Randomized trial of a behavioral intervention to prevent excessive gestational weight gain: The fit for delivery study. Obesity 2010;18(Suppl 2):S68.<br>66. Polley BA, Wing RR, Sims CJ. Randomized controlled trial to prevent excessive weight gain in pregnant women. International Journal of Obesity and Related Metabolic Disorders 2002;26(11):1494–502.<br>67. Hayes L, Bell R, Robson S, Poston L. Association between physical activity in obese pregnant women and pregnancy outcomes: The UPBEAT pilot study. Annals of Nutrition and Metabolism 2014;64(3-4):239–46.?<br>68. Hayes L, Mcparlin C, Kinnunen TI, Poston L, Robson SC, Bell R. Change in level of physical activity during pregnancy in obese women: findings from the UPBEAT pilot trial. BMC Pregnancy and Childbirth 2015;15:52.?<br>69. Maitland RA, Barr S, Briley A, Seed P, Poston L. Incidence of gestational diabetes in an obese population using the International Association of Diabetes and Pregnancy Study Groups (IADPSG) criteria in the UK Pregnancies Better Eating and Activity Trial (UPBEAT) pilot study. Diabetic Medicine 2012;29(Suppl 1):152.?<br>70. Poston L, Briley AL, Barr S, Bell R, Croker H, Coxon?K, et al. Developing a complex intervention for diet and activity behaviour change in obese pregnant women (the UPBEAT trial); assessment of behavioural change and process evaluation in a pilot randomised controlled trial. BMC Pregnancy and Childbirth 2013;13(1):148.?<br>71. Poston L, Holmes B, Kinnunen T, Croker H, Bell R, Sanders T, et al. A complex intervention to improve outcome in obese pregnancies; the upbeat study. Archives of Disease in Childhood: Fetal and Neonatal Edition 2011;96 (Suppl 1):Fa97.?<br>72. Schneeberger C, Flynn A, Barr S, Seed PT, Inskip HM, Poston L. Maternal diet patterns and glycaemic load in obese pregnant women taking part in a pilot trial of a lifestyle intervention (the upbeat trial). Diabetes 2014; Vol. 63:A342.<br>73. Briley A, Seed P, Singh C, Patel N, Poston L. Gestational weight gain in obese pregnant women, the impact of?a lifestyle intervention and implications for guidelines (UPBEAT trial). BJOG: an international journal of obstetrics and gynaecology 2016;123(Suppl 1):55–6.?<br>74. Briley A, Seed PT, Singh C, Patel N, Poston L. Gestational weight gain, BMI and pregnancy outcomes in obese pregnant women in the UPBEAT behavioural intervention RCT. Reproductive Sciences 2016;23(1 Suppl 1):277A.<br>75. Briley AL, Barr S, Badger S, Bell R, Croker H, Godfrey KM, et al. A complex intervention to improve pregnancy outcome in obese women; the UPBEAT randomised controlled trial. BMC Pregnancy and Childbirth 2014;14 (1):74.

Review	References of included behavioural interventions delivered in pregnancy
	76. Briley AL, Barr S, Badger S, Bell R, Croker H, Godfrey KM, et al. Erratum: A complex intervention to improve pregnancy outcome in obese women; the UPBEAT randomised controlled trial. BMC Pregnancy and Childbirth 2015;15:111.<br>77. Hayes L, Bell R, Robson S, Poston L. UPBEAT study: Association between physical activity in obese pregnant women and health of the offspring. The Power of Programming 2014: International Conference on Developmental Origins of Adiposity and Long-Term Health; 2014 March 13-15; Munich, Germany. 2014:48. ISRCTN89971375. UK Pregnancies Better Eating and Activity Trial. isrctn.com/ISRCTN89971375 (first received 23 July 2008).<br>78. Patel N, Godfrey KM, Pasupathy D, Levin J, Flynn AC, Hayes L, et al. Infant adiposity following a randomised controlled trial of a behavioural intervention in obese pregnancy. International Journal of Obesity 2017 Mar 21 [Epub ahead of print]. [DOI: 10.1038/ijo.2017.44<br>79. Patel NR, Pasupathy D, Flynn AC, Hayes L, Levin JG, Singh C, et al. The UPBEAT behavioural intervention in obese pregnant women - maternal and infant follow-up 6 months postpartum. Reproductive Sciences 2016;23(Suppl 1):71A.<br>80. Poston L. The UPBEAT study: A lifestyle intervention?in obese pregnant women. The Power of Programming 2014: International Conference on Developmental Origins of Adiposity and Long-Term Health; 2014 March 13-15; Munich, Germany. 2014:22.?<br>81. Poston L, Bell R, Croker H, Flynn AC, Godfrey KM, Goff L, et al. Effect of a behavioural intervention in obese pregnant women (the UPBEAT study): a multicentre, randomised controlled trial. Lancet. Diabetes and Endocrinology 2015;3(10):767–77.?<br>82. Seed PT, Briley A, Singh C, Patel N, Poston L. A novel method for devising optimal gestational weight gain in obese pregnant women. Reproductive Sciences 2016; Vol. 23, issue 1 Suppl 1:276A.?<br>83. Vieira MC, Pasupathy D, Patel NR, White SL, Briley A, Seed PT, et al. Factors associated with uncomplicated pregnancy in obese women from the UPBEAT trial. Reproductive Sciences 2016;23(Suppl 1):109A.?White L, Pasupathy D, Vieira MC, Briley AL, Seed P, Lawlor DA, et al. Prediction of gestational diabetes (GDM) in obese women. Reproductive Sciences 2016;23(Suppl 1): 124A.<br>84. Rauh 2013 DRKS00003801. Feasibility of a lifestyle intervention?in pregnancy to optimize maternal weight development. drks-neu.uniklinik-freiburg.de/drks web/navigate.do? navigationId=trial.HTML&TRIAL ID=DRKS00003801 (first received 24 April 2012).?<br>85. Rauh K, Gabriel E, Kerschbaum E, Schuster T, von Kries R, Amann-Gassner U, et al. Safety and efficacy of a lifestyle intervention for pregnant women to prevent excessive maternal weight gain: a cluster-randomized controlled trial. BMC Pregnancy and Childbirth 2013;13(1):151.<br>86. Rauh K, Gunther J, Kunath J, Stecher L, Hauner H. Lifestyle intervention to prevent excessive maternal weight gain: mother and infant follow-up at 12 months postpartum. BMC Pregnancy and Childbirth 2015;15:265.<br>87. Hillesund ER, Bere E, Sagedal LR, Vistad I, Overby NC. Effect of a diet intervention during pregnancy on dietary behavior in the randomized controlled Norwegian Fit for Delivery study. Journal of Developmental Origins of Health and Disease 2016;7(5):538–47.?NCT01001689. Fit for delivery: a study of the effect of exercise sessions and nutritional counselling on pregnancy outcome (FFF). clinicaltrials.gov/ct2/show/NCT01001689 (first received 26 October 2009).?<br>88. Sagedal LR. Who are we missing? Examining non- participants in ”Fit for Delivery“, a randomized, controlled trial of a lifestyle intervention in pregnancy. Pregnancy Hypertension 2014;4(3):237.?<br>89. Sagedal LR, Henriksen T, Overby NC, Lohne-Seiler?H, Torstveit MK, Bere E, et al. The problem of non- participation: Who declined to participate in ”Fit for Delivery“, a randomized, controlled trial of a lifestyle intervention in pregnancy?. The Power of Programming 2014: International Conference on Developmental Origins of Adiposity and Long-Term Health; 2014 March 13-15; Munich, Germany. 2014:63.?<br>90. Sagedal LR, Overby N, Lohne-Seiler H, Bere E, Torstveit M, Henriksen T, et al. Study protocol: Fit for Delivery - can a lifestyle intervention in pregnancy result in measurable health benefits for mothers and newborns? A randomized controlled trial. BMC Public Health 2013;13:132.?<br>91. Sagedal LR, Overby NC, Bere E, Torstveit MK, Lohne- Seiler H, Smastuen M, et al. Lifestyle intervention to limit gestational weight gain: the Norwegian Fit for Delivery randomised controlled trial. BJOG: an international journal of obstetrics and gynaecology 2017;124(1):97–109.?<br>92. Sagedal LR, Sanda B, Overby NC, Bere E, Torstveit MK, Lohne-Seiler H, et al. The effect of prenatal lifestyle intervention on weight retention 12 months postpartum: results of the Norwegian Fit for Delivery randomised controlled trial. BJOG: an international journal of obstetrics and gynaecology 2017;124(1):111–21.<br>93. Vinter 2011 NCT00530439. Lifestyle and pregnancy: the clinical effect of lifestyle intervention during pregnancy in obese women (LiP). clinicaltrials.gov/ct2/show/NCT00530439 (first received 13 September 2007).?NCT01918319. Lifestyle in pregnancy and offspring (LiPO). https://clinicaltrials.gov/ct2/show/NCT01918319 (first received 2 August 2013).?NCT01918423. Lifestyle in pregnancy and offspring?- comparison between children born to obese women?and children born to normal weight women (LiPO). clinicaltrials.gov/ct2/show/NCT01918423 (first received 2 August 2013).?<br>94. Tanvig M. Offspring body size and metabolic profile - Effects of lifestyle intervention in obese pregnant women. Danish Medical Journal 2014;61(7):B4893.<br>95. Tanvig M, Vinte CA, Jorgensen JS, Wehberg S, Ovesen PG, Lamont RF, et al. Anthropometrics and body composition by dual energy X-ray in children of obese women: a follow-up of a randomized controlled trial (the Lifestyle in Pregnancy and Offspring [LiPO] study). PLOS One 2014;9 (2):e89590.<br>96. Tanvig M, Vinter CA, Jorgensen JS, Wehberg S, Ovesen PG, Beck-Nielsen H, et al. Effects of lifestyle intervention in pregnancy and anthropometrics at birth on offspring metabolic profile at 2.8 years - results from the Lifestyle in Pregnancy and Offspring (LiPO) study. Journal of Clinical Endocrinology and Metabolism 2015;100(1):175–83.?<br>97. Vinter C, Jensen D, Ovesen P, Beck-Nielsen H, Lamont R, Jorgensen J. Postpartum weight retention and breastfeeding among obese women from the LiP (Lifestyle in Pregnancy) Study. Acta Obstetricia et Gynecologica Scandinavica 2012; 91(Suppl 159):141–2.<br>98. Vinter CA, Jensen DM, Ovesen P, Beck-Nielsen H, Jorgensen JS. The LiP (Lifestyle in Pregnancy) study: a randomized controlled trial of lifestyle intervention in 360 obese pregnant women. Diabetes Care 2011;34(12): 2502–7.

Review	References of included behavioural interventions delivered in pregnancy
	99. Vinter CA, Jensen DM, Ovesen P, Beck-Nielsen H, Tanvig M, Lamont RF, et al. Postpartum weight retention and breastfeeding among obese women from the randomized controlled Lifestyle in Pregnancy (LiP) trial. Acta Obstetricia et Gynecologica Scandinavica 2014;93:794–801.<br>100. Vinter CA, Jensen DM, Ovesen PG, Beck-Nielsen H, Jorgensen JS. Lifestyle and pregnancy (LIP) study: The clinical effect of lifestyle intervention during pregnancy in obese women. Diabetes 2011;60:A348–9.<br>101. Vinter CA, Jørgensen JS, Ovesen P, Beck-Nielsen H, Skytthe A, Jensen DM. Metabolic effects of lifestyle intervention in obese pregnant women. results from the randomized controlled trial 'lifestyle in pregnancy' (LiP). Diabetic Medicine 2014;31(11):1323–30.<br>102. Wang S, Ma JM, Yang HX. Lifestyle intervention for gestational diabetes mellitus prevention: A cluster- randomized controlled study. Chronic Diseases and Translational Medicine 2015;1(3):169–74.
Sherifali *et al.* 2017 [36]	1. Pollak KI, Alexander SC, Bennett G, Lyna P, Coffman CJ, Bilheimer A, et al. Weight-related SMS texts promoting appropriate pregnancy weight gain: a pilot study. Patient Educ Couns 2014 Nov;97(2):256-260 [FREE Full text] [doi: 10.1016/j.pec.2014.07.030] [Medline: 25153313]<br>2. Smith K, Lanningham-Foster L, Welch A, Campbell C. Web-based behavioral intervention increases maternal exercise but does not prevent excessive gestational weight gain in previously sedentary women. J Phys Act Health 2016 Jun;13(6):587-593. [doi: 10.1123/jpah.2015-0219] [Medline: 26594820]<br>3. Herring SJ, Cruice JF, Bennett GG, Rose MZ, Davey A, Foster GD. Preventing excessive gestational weight gain among African American women: a randomized clinical trial. Obesity (Silver Spring) 2016 Jan;24(1):30-36 [FREE Full text] [doi: 10.1002/oby.21240] [Medline: 26592857]<br>4. Pérez-Ferre N, Galindo M, Fernández MD, Velasco V, Runkle I, de la Cruz MJ, et al. The outcomes of gestational diabetes mellitus after a telecare approach are not inferior to traditional outpatient clinic visits. Int J Endocrinol 2010;2010:386941 [FREE Full text] [doi: 10.1155/2010/386941] [Medline: 20628517]<br>5. Carral F, Ayala Mdel C, Fernández JJ, González C, Piñero A, García G, et al. Web-based telemedicine system is useful for monitoring glucose control in pregnant women with diabetes. Diabetes Technol Ther 2015 May;17(5):349-354. [doi: 10.1089/dia.2014.0223] [Medline: 25629547]<br>6. Soltani H, Duxbury AM, Arden MA, Dearden A, Furness PJ, Garland C. Maternal obesity management using mobile technology: a feasibility study to evaluate a text messaging based complex intervention during pregnancy. J Obes 2015;2015:814830 [FREE Full text] [doi: 10.1155/2015/814830] [Medline: 25960889]
Song *et al.* 2016 [37]	1. Hui AL, Back L, Ludwig S et al. Effects of lifestyle intervention on dietary intake, physical activity level, and gestational weight gain in pregnant women with different pre-pregnancy body mass index in a randomized control trial. BMC Pregnancy Childbirth 2014; 14: 331 PubMed PMID: 25248797. PubMed Central PMCID: 4287470.<br>2. International Association of Diabetes and Pregnancy Study Groups Consensus Panel, Metzger BE, Gabbe SG, Persson B, Buchanan TA et al. International Association of diabetes and Pregnancy Study Groups recommendations on the diagnosis and classification of hyperglycemia in pregnancy. Diabetes Care 2010;33: 676–82 PubMed PMID: 20190296. PubMed Central PMCID:2827530.<br>3. Bogaerts AF, Devlieger R, Nuyts E,Witters I, Gyselaers W, Van den Bergh BR. Effects of lifestyle intervention in obese pregnant women on gestational weight gain and mental health: a randomized controlled trial. Int J Obes (Lond) 2013; 37: 814–21 PubMed PMID: 23032404.<br>4. Stafne SN, Salvesen KA, Romundstad PR, Eggebo TM, Carlsen SM, Morkved S. Regular exercise during pregnancy to prevent gestational diabetes: a randomized controlled trial. Obstet Gynecol 2012; 119: 29–36 PubMed PMID: 22183208.<br>5. Oostdam N, van Poppel MN, Wouters MG et al. No effect of the FitFor2 exercise programme on blood glucose, insulin sensitivity, and birthweight in pregnant women who were overweight and at risk for gestational diabetes: results of a randomised controlled trial. BJOG: Int J Obstetrics Gynaecol 2012; 119: 1098–107 PubMed PMID: 22616913.<br>6. Petrella E, Malavolti M, Bertarini V et al. Gestational weight gain in overweight and obese women enrolled in a healthy lifestyle and eating habits program. J Maternal–Fetal Neonatal Med: Official J Eur Assoc Perinatal Med, Federation Asia Oceania Perinatal Soc, Int Soc Perinatal Obstet 2014; 27: 1348–52 PubMed PMID:24175912.<br>7. Rakhshani A, Nagarathna R, Mhaskar R, Mhaskar A, Thomas A, Gunasheela S. The effects of yoga in prevention of pregnancy complications in high-risk pregnancies: a randomized controlled trial. Prev Med 2012; 55: 333–40 PubMed PMID: 22884667.<br>8. Walsh JM, McGowan CA, Mahony R, Foley ME, McAuliffe FM. Low glycaemic index diet in pregnancy to prevent macrosomia (ROLO study): randomised control trial. BMJ 2012; 345: e5605 PubMed PMID: 22936795. PubMed Central PMCID: 3431285<br>9. Poston L, Bell R, Croker H et al. Effect of a behavioural intervention in obese pregnant women (the UPBEAT study): a multicentre, randomised controlled trial. Lancet Diabetes Endocrinol 2015; 3: 767–77 PubMed PMID: 26165396.<br>10. Ko CW, Napolitano PG, Lee SP, Schulte SD, Ciol MA, Beresford SA. Physical activity, maternal metabolic measures, and the incidence of gallbladder sludge or stones during pregnancy: a randomized trial. Am J Perinatol 2014; 31: 39–48 PubMed PMID:23456902.<br>11. Phelan S, Phipps MG, Abrams B, Darroch F, Schaffner A, Wing RR. Randomized trial of a behavioral intervention to prevent excessive gestational weight gain: the Fit for Delivery Study. Am J Clin Nutr 2011; 93: 772–9 PubMed PMID: 21310836. PubMed Central PMCID: 3057546.<br>12. Polley BA, Wing RR, Sims CJ. Randomized controlled trial to prevent excessive weight gain in pregnant women. International J Obesity Related Metabolic Disorders: J Int Assoc Study Obesity 2002; 26: 1494–502 PubMed PMID: 12439652.<br>13. Price BB, Amini SB, Kappeler K. Exercise in pregnancy: effect on fitness and obstetric outcomes-a randomized trial. Med Sci Sports Exerc 2012; 44: 2263–9 PubMed PMID: 22843114.

Review	References of included behavioural interventions delivered in pregnancy
	14. Nobles C, Marcus BH, Stanek EJ 3rd et al. Effect of an exercise intervention on gestational diabetes mellitus: a randomized controlled trial. Obstet Gynecol 2015; 125: 1195–204 PubMed PMID: 25932848. PubMed Central PMCID: 4418021.<br>15. Thornton YS, Smarkola C, Kopacz SM, Ishoof SB. Perinatal outcomes in nutritionally monitored obese pregnant women: a randomized clinical trial. J Natl Med Assoc 2009; 101: 569–77 PubMed PMID: 19585925.<br>16. Barakat R, Cordero Y, Coteron J, Luaces M, Montejo R. Exercise during pregnancy improves maternal glucose screen at 24–28 weeks: a randomised controlled trial. Br J Sports Med 2012; 46: 656–61 PubMed PMID: 21948120.<br>17. Cordero Y, Mottola MF, Vargas J, Blanco M, Barakat R. Exercise is associated with a reduction in gestational diabetes mellitus. Med Sci Sports Exerc 2015; 47: 1328–33 PubMed PMID:25333246.<br>18. Ruiz JR, Perales M, Pelaez M, Lopez C, Lucia A, Barakat R. Supervised exercise-based intervention to prevent excessive gestational weight gain: a randomized controlled trial. Mayo Clin Proc 2013; 88: 1388–97 PubMed PMID: 24290112.<br>19. Barakat R, Pelaez M, Lopez C, Lucia A, Ruiz JR. Exercise during pregnancy and gestational diabetes-related adverse effects: a randomised controlled trial. Br J Sports Med 2013; 47: 630–6 PubMed PMID: 23365418.<br>20. Barakat R, Perales M, Bacchi M, Coteron J, Refoyo I. A program of exercise throughout pregnancy. Is it safe to mother and newborn? Am J Health Promotion: AJHP 2014; 29: 2–8 PubMed PMID: 24200335.<br>21. Dodd JM, Turnbull D, McPhee AJ et al. Antenatal lifestyle advice for women who are overweight or obese: LIMIT randomised trial. BMJ 2014; 348: g1285 PubMed PMID: 24513442. PubMed Central PMCID: 3919179.<br>22. Harrison CL, Lombard CB, Strauss BJ, Teede HJ. Optimizing healthy gestational weight gain in women at high risk of gestational diabetes: a randomized controlled trial. Obesity 2013; 21: 904–9 PubMed PMID: 23784892.<br>23. Quinlivan JA, Lam LT, Fisher J. A randomised trial of a fourstep multidisciplinary approach to the antenatal care of obese pregnant women. Aust N Z J Obstet Gynaecol 2011; 51: 141–6 PubMed PMID: 21466516.<br>24. Koivusalo SB, Rono K, Klemetti MM et al. Gestational diabetes mellitus can be prevented by lifestyle intervention: the Finnish gestational diabetes prevention study (RADIEL): a randomized controlled trial. Diabetes Care 2016; 39: 24–30 PubMed PMID: 26223239.<br>25. Luoto R, Laitinen K, Nermes M, Isolauri E. Impact of maternal probiotic-supplemented dietary counselling on pregnancy outcome and prenatal and postnatal growth: a double-blind, placebo controlled study. Br J Nutr 2010; 103: 1792–9 PubMed PMID: 20128938.<br>26. Luoto R, Kinnunen TI, Aittasalo M et al. Primary prevention of gestational diabetes mellitus and large-for-gestational-age newborns by lifestyle counseling: a cluster-randomized controlled trial. PLoS Med 2011; 8: e1001036 PubMed PMID: 21610860. PubMed Central PMCID: 3096610.<br>27. Wolff S, Legarth J, Vangsgaard K, Toubro S, Astrup A. A randomized trial of the effects of dietary counseling on gestational weight gain and glucose metabolism in obese pregnant women. Int J Obes (Lond) 2008; 32: 495–501 PubMed PMID:18227847<br>28. Renault KM, Norgaard K, Nilas L et al. The Treatment of Obese PregnantWomen (TOP) study: a randomized controlled trial of the effect of physical activity intervention assessed by pedometer with or without dietary intervention in obese pregnant women. Am J Obstet Gynecol 2014; 210: 134. e1–9 PubMed PMID: 24060449.<br>29. Vinter CA, Jensen DM, Ovesen P, Beck-Nielsen H, Jorgensen JS. The LiP (Lifestyle in Pregnancy) study: a randomized controlled trial of lifestyle intervention in 360 obese pregnant women. Diabetes Care 2011; 34: 2502–7 PubMed PMID: 21972411. PubMed Central PMCID: 3220844
Tieu *et al.* 2017 [38]	1. Clapp JF. Influence of endurance exercise and diet on human placental development and fetal growth. Placenta 2006;27(6-7):527–34<br>2. Clapp JF III. Diet, exercise, and feto-placental growth. Archives of Gynecology and Obstetrics 1997;260:101–8<br>3. Clapp JF III. Effect of dietary carbohydrate on the glucose and insulin response to mixed caloric intake and exercise in both nonpregnant and pregnant women. Diabetes Care 1998;21(Suppl 2):B107–B112. Clapp JF III. Maternal carbohydrate intake and pregnancy outcome. Proceedings of the Nutrition Society 2002;61: 45–50<br>4. Fraser RB. High fibre diets in pregnancy. Nutrition in Pregnancy. Proceedings of 10th Study Group of the Royal College of Obstetricians and Gynaecologists; 1982 September. London: RCOG, 1983:269–80<br>5. Fraser RB, Ford FA, Milner RDG. A controlled trial of a high dietary fibre intake in pregnancy - effects on plasma glucose and insulin levels. Diabetologia 1983;25:238–41<br>6. Hoppu U, Isolauri E, Koskinen P, Laitinen K. Diet and blood lipids in 1-4 year-old children. Nutrition Metabolism and Cardiovascular Diseases 2013;23(10):980–6<br>7. Hoppu U, Isolauri E, Laakso P, Matomaki J, Laitinen K. Probiotics and dietary counselling targeting maternal dietary fat intake modifies breast milk fatty acids and cytokines. European Journal of Nutrition 2012;51(2):211–9<br>8. Laitinen K, Ilmonen J, Isolauri E. Dietary counselling and probiotic intervention during pregnancy modify postpartum adiposity. Annals of Nutrition and Metabolism 2011;58(Suppl 3):87<br>9. Laitinen K, Poussa T, Isolauri E, Nutrition, Allergy, Mucosal Immunology and Intestinal Microbiota Group. Probiotics and dietary counselling contribute to glucose regulation during and after pregnancy: a randomised controlled trial. British Journal of Nutrition 2009;101(11): 1679–87<br>10. Luoto R, Laitinen K, Nermes M, Isolauri E. Impact of maternal probiotic-supplemented dietary counseling during pregnancy on colostrum adiponectin concentration: A prospective, randomized, placebo-controlled study. Early Human Development 2012;88(6):339–44<br>11. Luoto R, Laitinen K, Nermes M, Isolauri E. Impact of maternal probiotic-supplemented dietary counselling on pregnancy outcome and prenatal and postnatal growth: a double-blind, placebo-controlled study. British Journal of Nutrition 2010;103(12):1792–9<br>12. Luoto R, Nermes M, Laitinen K, Isolauri E. Impact of maternal probiotic-supplemented dietary counselling on pregnancy outcome and prenatal and postnatal growth: a double-blind, placebo-controlled study. Pediatric Academic Societies Annual Meeting; 2009 May 2-5; Baltimore, USA. 2009<br>13. Niinivirta K, Isolauri E, Laakso P, Linderborg K, Laitinen K. Dietary counseling to improve fat quality during pregnancy alters maternal fat intake and infant essential fatty acid status. Journal of Nutrition 2011;141(7):1281–5<br>14. Niinivirta K, Laakso P, Linderborg K, Poussa T, Isolauri E, Laitinen K. Maternal dietary counseling during pregnancy and infant fatty acid profiles. International Journal of Food Sciences and Nutrition 2014;65(3):268–72

Review	References of included behavioural interventions delivered in pregnancy
	15. Piirainen T, Isolauri E, Lagstrom H, Laitinen K. Impact of dietary counselling on nutrient intake during pregnancy: a prospective cohort study. British Journal of Nutrition 2006; 96(6):1095–104<br>16. Vahamiko S, Isolauri E, Laitinen K. Weight status and dietary intake determine serum leptin concentrations in pregnant and lactating women and their infants. British Journal of Nutrition 2013;110(6):1098–106<br>17. Markovic 2016 ACTRN12610000681055. A randomized, two-arm parallel dietary intervention study to compare the effects of consuming a low glycemic diet or wholegrain high fibre diet on infant birth weight and body composition, complications related to Gestational Diabetes Mellitus (GDM) and progression to GDM diagnosis in women at high-risk of GDM. anzctr.org.au/Trial/Registration/ TrialReview.aspx?id=335632 Date first received: 18 August 2010<br>18. Kizirian N, Garnett S, Markovic T, Ross G, Louie J, Muirhead R, et al. Effects of a low-glycaemic index diet during pregnancy on offspring body composition: a pilot study. Obesity Research and Clinical Practice 2013;7(Suppl 2):e103<br>19. Kizirian N, Garnett S, Markovic T, Ross G, Muirhead R, Brodie S, et al. Maternal diet and infant body composition in women at risk of gestational diabetes mellitus. Obesity Research and Clinical Practice 2014;8(Suppl 1):55<br>20. Markovic TP, Muirhead R, Overs S, Kizirian N, Louie J, Sweeting A, et al. Predictors of birthweight in women at high risk of gestational diabetes mellitus. Obesity Research and Clinical Practice 2013;7(2):e3–4<br>21. Markovic TP, Muirhead R, Overs S, Ross GP, Louie JC, Kizirian N, et al. Randomized controlled trial investigating the effects of a low-glycemic index diet on pregnancy outcomes in women at high risk of gestational diabetes mellitus: the GI Baby 3 Study. Diabetes Care 2016;39(1): 31–8<br>22. Moses RG, Luebcke M, Davis WS, Coleman KJ, Tapsell LC, Petocz P, et al. Effect of a low-glycemic-index diet during pregnancy on obstetric outcomes. American Journal of Clinical Nutrition 2006;84(4):807–12<br>23. Moses RG, Luebke M, Petocz P, Brand-Miller JC. Maternal diet and infant size 2 y after the completion of a study of a low-glycemic-index diet in pregnancy. American Journal of Clinical Nutrition 2007;86(6):1806<br>24. Goletzke J, Buyken AE, Louie JC, Moses RG, BrandMiller JC. Dietary micronutrient intake during pregnancy is a function of carbohydrate quality. American Journal of Clinical Nutrition 2015;102(3):626–32<br>25. Moses RG, Casey S, Cleary J, Milosavljevic M, Quinn E, Tapsell L, et al. Effect of low glycaemic index dietary advice in normal pregnancy: The PREGGIO study. Obesity Research and Clinical Practice 2013;7:e34–5<br>26. Moses RG, Casey SA, Quinn EG, Cleary JM, Tapsell LC, Milosavljevic M, et al. Pregnancy and Glycemic Index Outcomes study: effects of low glycemic index compared with conventional dietary advice on selected pregnancy outcomes. American Journal of Clinical Nutrition 2014;99 (3):517–23<br>27. Quinlivan J. A randomised trial of a multidisciplinary teamcare approach involving obstetric, dietary and clinical psychological input in obese pregnant women to reduce the incidence of gestational diabetes. https:// www.anzctr.org.au/Trial/Registration/TrialReview.aspx?id= 821 (accessed 2 November 2015)<br>28. Quinlivan JA, Lam LT, Fisher J. A randomised trial of a four-step multidisciplinary approach to the antenatal care of obese pregnant women. Australian and New Zealand Journal of Obstetrics and Gynaecology 2011;51(2):141–6<br>29. Thornton YS, Smarkola C, Kopacz SM, Ishoof SB. Perinatal outcomes in nutritionally monitored obese pregnant women: a randomized clinical trial. Journal of the National Medical Association 2009;101(6):569–77<br>30. Vitolo MR, Fraga Bueno MS, Mendes Gama C. Impact of a dietary counseling program on the gain weight speed of pregnant women attended in a primary care service. Revista Brasileira de Ginecologia e Obstetricia 2011;33(1):13–9<br>31. Donnelly J, Horan M, Walsh J, McGowan C, Byrne J, Molloy EJ, et al. Impact of a low GI diet on neonatal body composition (ROLO Kids). Pediatric Academic Societies Annual Meeting; 2013 May 4-7; Washington DC, USA. 2013<br>32. Donnelly JM, Lindsay KL, Walsh JM, Horan M, Molloy EJ, McAuliffe FM. Fetal metabolic influences of neonatal anthropometry and adiposity. BMC Pediatrics 2015;15(1): 175<br>33. Donnelly JM, Walsh JM, Byrne J, Molloy E, McAuliffe FM. Altered neonatal anthropometric measurements following maternal low GI diet in pregnancy (ROLO study). Acta Obstetricia et Gynecologica Scandinavica 2013;92(s160):13<br>34. Donnelly JM, Walsh JM, Byrne J, Molloy EJ, McAuliffe FM. Impact of maternal diet on neonatal anthropometry: A randomized controlled trial. Pediatric Obesity 2015;10(1): 52–6<br>35. Donnelly JMT, Lindsay KL, Walsh JM, Horan M, Molloy EJ, McAuliffe F. Impact of maternal and fetal inflammatory markers on neonatal and infant adiposity. Archives of Disease in Childhood 2015;100:A133–4<br>36. Horan M, Donnelly J, Gibney E, McGowan C, McAuliffe F. The association of maternal characteristics and macronutrient intake in pregnancy with neonatal body composition. The Power of Programming 2014: International Conference on Developmental Origins of Adiposity and Long-Term Health; 2014 March 13-15; Munich, Germany. 2014<br>37. Horan M, McGowan C, Donnelly J, Gibney E, McAuliffe F. Maternal diet and weight at 3 months partum following a pregnancy intervention with a low glycaemic index diet: Results from the ROLO randomised control trial. Archives of Disease in Childhood. Fetal and Neonatal Edition 2014;99 (Suppl 1):A129-A130, Abstract no: PMM.20<br>38. Horan MK, McGowan CA, Donnelly J, Gibney E, McAuliffe FM. The association of maternal characteristics and macronutrient intake in pregnancy with neonatal body composition. Archives of Disease in Childhood. Fetal and Neonatal Edition 2014;99(Suppl 1):A11<br>39. Horan MK, McGowan CA, Doyle O, McAuliffe FM. Well-being in pregnancy: An examination of the effect of socioeconomic, dietary and lifestyle factors including impact of a low glycaemic index dietary intervention. European Journal of Clinical Nutrition 2014;68(1):19–24<br>40. Horan MK, McGowan CA, Gibney ER, Donnelly JM, McAuliffe FM. Maternal diet and weight at 3 months postpartum following a pregnancy intervention with a low glycaemic index diet: results from the ROLO randomised control trial. Nutrients 2014;6(7):2946–55<br>41. Horan MK, McGowan CA, Gibney ER, Donnelly JM, McAuliffe FM. Maternal low glycaemic index diet, fat intake and postprandial glucose influences neonatal adiposity - secondary analysis from the ROLO study. Nutrition Journal 2014;13(1):78. ISRCTN54392969. A randomised controlled trial of low glycaemic index carbohydrate diet versus no dietary intervention in the prevention of recurrence of foetal macrosomia. controlled-trials.com/ISRCTN54392969 Date first received: 10 August 2009

Review	References of included behavioural interventions delivered in pregnancy
	42. Mahony R, Byrne J, Curran S, O'Herlihy C, McAuliffe F. A pilot study of the feasibility of a randomised trial of low glycaemic diet versus normal diet from early pregnancy in euglycaemic women. Archives of Disease in Childhood. Fetal and Neonatal Edition 2008;93(Suppl 1):Fa38<br>43. McAuliffe F. Maternal nutrition and fetal health. The Power of Programming 2014: International Conference on Developmental Origins of Adiposity and Long-Term Health; 2014 March 13-15; Munich, Germany. 2014<br>44. McGowan CA, Walsh JM, Byrne J, Curran S, McAuliffe FM. The influence of a low glycemic index dietary intervention on maternal dietary intake, glycemic index and gestational weight gain during pregnancy: a randomized controlled trial. Nutrition Journal 2013;12(1):140<br>45. Walsh J, Mahony R, Foley M, Mc Auliffe F. A randomised control trial of low glycaemic index carbohydrate diet versus no dietary intervention in the prevention of recurrence of macrosomia. BMC Pregnancy and Childbirth 2010;10:16<br>46. Walsh J, Mahony R, Foley M, McAuliffe F. ROLO study: a randomized control trial of low glycemic index diet to prevent macrosomia in euglycemic women. American Journal of Obstetrics and Gynecology 2012;206(Suppl 1):S4<br>47. Walsh J, McGowan C, Byrne J, Foley M, Mahony R, McAuliffe F. The influence of a low glycaemic index dietary intervention on maternal glycaemic index, dietary intake and gestational weight gain. American Journal of Obstetrics and Gynecology 2013;208(1 Suppl):S33<br>48. Walsh JM, Mahony RM, Canty G, Foley ME, McAuliffe FM. Identification of those most likely to benefit from a low-glycaemic index dietary intervention in pregnancy. British Journal of Nutrition 2014;112:583–9<br>49. Walsh JM, Mahony RM, Culliton M, Foley ME, McAuliffe FM. Impact of a low glycemic index diet in pregnancy on markers of maternal and fetal metabolism and inflammation. Reproductive Sciences 2014;21(11):1378–81<br>50. Walsh JM, McAuliffe FM. Impact of maternal nutrition on pregnancy outcome - Does it matter what pregnant women eat? Best Practice and Research. Clinical Obstetrics and Gynaecology 2015;29(1):63–78<br>51. Walsh JM, McGowan CA, Mahony R, Foley ME, McAuliffe FM. Low glycaemic index diet in pregnancy to prevent macrosomia (ROLO study): randomised control trial. BMJ 2012;345:e5605.<br>52. Wolff S, Legarth J, Vangsgaard K, Toubro S, Astrup A. A randomized trial of the effects of dietary counseling on gestational weight gain and glucose metabolism in obese pregnant women. International Journal of Obesity 2008;32 (3):495–501
Yeo *et al.* 2017 [39]	1. Renault KM, Norgaard K, Nilas Let al. The Treatment of Obese Pregnant Women (TOP) study: a randomized controlled trial of the effect of physical activity intervention assessed by pedometer with or without dietary intervention in obese pregnant women. AmJ Obstet Gynecol 2014;210(2): 134.e131–134.e139.18.<br>2. Vinter CA, Jensen DM, Ovesen P, Beck-Nielsen H, Jorgensen JS. The LiP (Lifestyle in Pregnancy) study: a randomized controlled trial of lifestyle intervention in 360 obese pregnant women. Diabetes Care 2011;34(12): 2502–2507<br>3. Asbee SM, Jenkins TR, Butler JR, White J, Elliot M, Rutledge A. Preventing excessive weight gain during pregnancy through dietary and lifestyle counselling: a randomized controlled trial. Obstet Gynecol 2009;113(2 Pt 1): 305–312.26.<br>4. Bogaerts AF, Devlieger R, Nuyts E, Witters I, Gyselaers W, Vanden Bergh BR. Effects of lifestyle intervention in obese pregnant women on gestational weight gain and mental health: a randomized controlled trial. Int J Obes (Lond)2013;37(6): 814–821.27.<br>5. Harrison CL, Lombard CB, Strauss BJ, Teede HJ. Optimizing healthy gestational weight gain in women at high risk of gestational diabetes: a randomized controlled trial. Obesity (Silver Spring)2013;21(5): 904–909.28.<br>6. Quinlivan JA, Lam LT, Fisher JA. randomised trial of a four-step multidisciplinary approach to the antenatal care of obese pregnant women. Aust N Z J Obstet Gynaecol 2011;51(2): 141–146.29.<br>7. Thornton YS, Smarkola C, Kopacz SM, Ishoof SB. Perinatal outcomes in nutritionally monitored obese pregnant women: a randomized clinical trial. J Natl Med Assoc 2009;101(6): 569–577.<br>8. Vesco KK, Karanja N, King JCet al. Efficacy of a group-based dietary intervention for limiting gestational weight gain among obese women: a randomized trial. Obesity (Silver Spring)2014;22(9): 1989–1996.31.<br>9. Wolff S, Legarth J, Vangsgaard K, Toubro S, Astrup A. A randomized trial of the effects of dietary counselling on gestational weight gain and glucose metabolism in obese pregnant women.2008;32(3):495–501.http://www.nature.com/ijo/journal/v32/n3/pdf/0803710a.pdf.32.<br>10. Herring SJ, Cruice JF, Bennett GG, Rose MZ, Davey A, Foster GD. Preventing excessive gestational weight gain among African American women: a randomized clinical trial. Obesity (SilverSpring)2016;24(1): 30–36.33.<br>11. Petrella E, Malavolti M, Bertarini Vet al. Gestational weight gain in overweight and obese women enrolled in a healthy lifestyle and eating habits program .J Matern Fetal Neonatal Med2014;27(13): 1348–1352.34.<br>12. Jeffries K, Shub A, Walker SP, Hiscock R, Permezel M. Reducing excessive weight gain in pregnancy: a randomised controlled trial.Med J Aust2009;191(8): 429–433.35.<br>13. Nascimento SL, Surita FG, Parpinelli MA, Siani S, Pinto e Silva JL. The effect of an antenatal physical exercise programme on maternal/perinatal outcomes and quality of life in overweight and obese pregnant women: a randomised clinical trial.BJOG2011;118(12): 1455–1463.36.<br>14. McCarthy EA, Walker SP, Ugoni A, Lappas M, Leong O, Shub A. Self-weighing and simple dietary advice for overweight and obese pregnant women to reduce obstetric complications without impact on quality of life: a randomised controlled trial.BJOG2016;123(6): 965–973.37.<br>15. Barakat R, Lucia A, Ruiz JR. Resistance exercise training during pregnancy and new-born's birth size: a randomised controlled trial. Int J Obes (2005)2009;33(9): 1048–1057.38.<br>16. Harden SM, Beauchamp MR, Pitts BH et al. Group-based life-style sessions for gestational weight gain management: a mixed method approach. Am J Health Behav 2014;38(4): 560–569.39.<br>17. Hui AL, Back L, Ludwig Set al. Effects of lifestyle intervention on dietary intake, physical activity level, and gestational weight gain in pregnant women with different pre-pregnancy body mass index in a randomized control trial. BMC Pregnancy Childbirth 2014;14: 331.40.<br>18. Koivusalo SB, Rono K, Klemetti MM et al. Gestational diabetes mellitus can be prevented by lifestyle intervention: the Finnish Gestational Diabetes Prevention study (RADIEL): a randomized controlled trial. Diabetes Care 2016;39(1): 24–30.41.

Review	References of included behavioural interventions delivered in pregnancy
	19. Phelan S, Phipps MG, Abrams B, Darroch F, Schaffner A, Wing RR. Randomized trial of a behavioral intervention to prevent excessive gestational weight gain: the fit for delivery study. Am JClin Nutr 2011;93(4): 772–779.42.<br>20. Santos IA, Stein R, Fuchs SC et al. Aerobic exercise and sub-maximal functional capacity in overweight pregnant women: a randomized trial. Obstet Gynecol 2005;106(2): 243–249.43.<br>21. Seneviratne SN, Jiang Y, Derraik Jet al. Effects of antenatal exercise in overweight and obese pregnant women on maternal and perinatal outcomes: a randomised controlled trial. BJOG 2016;123(4): 588–597.44.<br>22. Althuizen E, van der Wijden CL, van Mechelen W, Seidell JC, van Poppel MN. The effect of a counselling intervention on weight changes during and after pregnancy: a randomised trial. BJOG 2013;120(1): 92–99.45.<br>23. Byrne NM, Groves AM, McIntyre HD, Callaway LK. Changes in resting and walking energy expenditure and walking speed during pregnancy in obese women. Am J Clin Nutr 2011;94(3):819–830<br>24. Dodd JM, Turnbull D, McPhee AJ et al. Antenatal lifestyle ad-vice for women who are overweight or obese: LIMIT randomised trial. BMJ 2014;348.47.<br>25. Garnaes KK, Morkved S, Salvesen O, Moholdt T. Exercise Med 2016;13(7): e1002079.48.<br>26. Guelinckx I, Devlieger R, Mullie P, Vansant G. Effect of life-style intervention on dietary habits, physical activity, and gestational weight gain in obese pregnant women: a randomized controlled trial. Am J Clin Nutr 2010;91(2): 373–380.49.<br>27. Kong KL, Campbell CG, Foster RC, Peterson AD, Lanningham-Foster L. A pilot walking program promotes moderate-intensity physical activity during pregnancy. Med SciSports Exerc 2014;46(3): 462–471.50.<br>28. Rhodes ET, Pawlak DB, Takoudes TC et al. Effects of a low-glycemic load diet in overweight and obese pregnant women a pilot randomized controlled trial. Am J Clin Nutr 2010;92(6):1306–1315<br>29. Dekker Nitert M, Barrett HL, Denny KJ, McIntyre HD, Callaway LK. Exercise in pregnancy does not alter gestational weight gain, MCP-1 or leptin in obese women. Aust N Z J ObstetGynaecol2015;55(1): 27–33.55.<br>30. Ruiz JR, Perales M, Pelaez M, Lopez C, Lucia A, Barakat R. Supervised exercise-based intervention to prevent excessive gestational weight gain: a randomized controlled trial. Mayo Clin Proc 2013;88(12): 1388–1397.56.<br>31. Hawkins M, Hosker M, Marcus BH et al.A pregnancy life-style intervention to prevent gestational diabetes risk factors in overweight Hispanic women: a feasibility randomized controlled trial. Diabet Med 2015;32(1): 108–115<br>32. Ong MJ, Guelfi KJ, Hunter T, Wallman KE, Fournier PA, Newnham JP. Supervised home-based exercise may attenuate the decline of glucose tolerance in obese pregnant women. Diabetes & Metabolism 2009;35(5): 418–421
Zhang *et al.* 2018 [40]	1. Markovic TP, Muirhead R, Overs S, Ross GP, Louie JCY, Kizirian N, Denyer G, Petocz P, Hyett J, Brand-Miller JC (2016) Randomized controlled trial investigating the effects of a low glycemic index diet on pregnancy outcomes in women at high risk of gestational diabetes mellitus: the GI Baby 3 study. Diabetes Care 39:31–38<br>2. Ma WJ, Huang ZH, Huang BX, Qi BH, Zhang YJ, Xiao BX, Li YH, Chen L, Zhu HL (2015) Intensive low-glycaemic-load dietary intervention for the management of glycaemia and serum lipids among women with gestational diabetes: a randomized control trial. Public Health Nutr 18:1506–1513<br>3. Moses RG, Casey SA, Quinn EG, Cleary JM, Tapsell LC, Milosavljevic M, Petocz P, Brand-Miller JC (2014) Pregnancy and glycemic index outcomes study: effects of low glycaemic index compared with conventional dietary advice on selected pregnancy outcomes(1–3). Am J Clin Nutr 99:517–523<br>4. Perichart-Perera O, Balas-Nakash M, Rodriguez-Cano A, Legorreta-Legorreta J, Parra-Covarrubias A, Vadillo-Ortega F (2012) Low glycemic index carbohydrates versus all types of carbohydrates for treating diabetes in pregnancy: a randomized clinical trial to evaluate the effect of glycemic control. Int J Endocrinol 2012:296017<br>5. Walsh JM, McGowan CA, Mahony R, Foley ME, McAuliffe FM (2012) Low glycaemic index diet in pregnancy to prevent macrosomia (ROLO study): randomised control trial. BMJ 345:e5605–e5613<br>6. Grant SM, Wolever TMS, O'Connor DL, Nisenbaum R, Josse RG (2011) Effect of a low glycaemic index diet on blood glucose in women with gestational hyperglycaemia. Diabetes Res Clin Pract 91:15–22<br>7. Louie JCY, Markovic TP, Perera N, Foote D, Petocz P, Ross GP, Brand-Miller JC (2011) A randomized controlled trial investigating the effects of a low-glycemic index diet on pregnancy outcomes in gestational diabetes mellitus. Diabetes Care 34:2341–2346<br>8. Rhodes ET, Pawlak DB, Takoudes TC, Ebbeling CB, Feldman HA, Lovesky MM, Cooke EA, Leidig MM, Ludwig DS (2010) Effects of a low-glycemic load diet in overweight and obese pregnant women a pilot randomized controlled trial. Am J Clin Nutr 92:1306–1315<br>9. Moses RG, Barker M, Winter M, Petocz P, Brand-Miller JC (2009) Can a low-glycemic index diet reduce the need for insulin in gestational diabetes mellitus? A randomized trial. Diabetes Care 32:996–1000<br>10. Moses RG, Luebcke M, Davis WS, Coleman KJ, Tapsell LC, Petocz P, Brand-Miller JC (2006) Effect of a low-glycemic-index diet during pregnancy on obstetric outcomes. Am J Clin Nutr 84:807–812<br>11. Clapp JF (1997) Diet, exercise, and feto-placental growth. Arch Gynecol Obstet 260:101–108
Zheng *et al.* 2017 [41]	1. Barakat R, Pelaez M, Lopez C, Lucia A, Ruiz JR (2013) Exercise during pregnancy and gestational diabetes-related adverse effects: a randomised controlled trial. Br J Sports Med 47:630–636<br>2. Guelfi KJ, Ong MJ, Crisp NA, Fournier PA, Wallman KE, Grove JR et al (2016) Regular exercise to prevent the recurrence of gestational diabetes mellitus: a randomized controlled trial. Obstet Gynecol 128:819–827<br>3. Stafne SN, Salvesen KA, Romundstad PR, Eggebo TM, Carlsen SM, Morkved S (2012) Regular exercise during pregnancy to prevent gestational diabetes: a randomized controlled trial. Obstet Gynecol 119:29–36<br>4. Nobles C, Marcus BH, Stanek EJ 3rd, Braun B, Whitcomb BW, Solomon CG et al (2015) Effect of an exercise intervention on gestational diabetes mellitus: a randomized controlled trial. Obstet Gynecol 125:1195–1204<br>5. Wang C, Wei Y, Zhang X, Zhang Y, Xu Q, Su S et al (2016) Effect of regular exercise commenced in early pregnancy on the incidence of gestational diabetes mellitus in overweight and obese pregnant women: a randomized controlled trial. Diabetes Care 39:e163–e164

Review	References of included behavioural interventions delivered in pregnancy
Gresham *et al.* 2014 [42]	1. Smuts CM, Borod E, Peeples JM, Carlson SE. High-DHA eggs: feasibility as a means to enhance circulating DHA in mother and infant. Lipids 2003;38:407–14.<br>2. Chan GM, McElligott K, McNaught T, Gill G. Effects of dietary calcium intervention on adolescent mothers and newborns: a randomized controlled trial. Obstet Gynecol 2006;108:565–71.<br>3. Mardones-Santander F, Rosso P, Stekel A, Ahumada E, Llaguno S, Pizarro F, Salinas J, Vial I, Walter T. Effect of a milk-based food supplement on maternal nutritional status and fetal growth in underweight Chilean women. Am J Clin Nutr 1988;47:413–9.<br>4. Ross SM, Nel E, Naeye RL. Differing effects of low and high bulk maternal dietary supplements during pregnancy. Early Hum Dev 1985;10:295–302.<br>5. Rush D, Stein Z, Susser M. A randomized controlled trial of prenatal nutritional supplementation in New York City. Pediatrics 1980;65:683–97.<br>6. Mora JO, Clement J, Christiansen N, Suescun J, Wagner M, Herrera MG. Nutritonal supplementation and the outcome of pregnancy. III. Perinatal and neonatal mortality. Nutr Rep Int 1978;18:167–75.<br>7. Courville AB, Harel O, Lammi-Keefe C. Consumption of a DHAcontaining functional food during pregnancy is associated with lower infant ponderal index and cord plasma insulin concentration. Br J Nutr 2011;106:208–12.<br>8. Luoto R, Kinnunen T, Aittasalo M, Kolu P, Raitanen J, Ojala K, Mansikkama¨ki K, Lamberg S, Vasankari T, Komulainen T, et al. Primary prevention of gestational diabetes mellitus and large-for-gestationalage newborns by lifestyle counseling: a cluster-randomized controlled trial. PLoS Med 2011;8:e1001036.<br>9. Quinlivan JA, Lam LT, Fisher J. A randomised trial of a four-step multidisciplinary approach to the antenatal care of obese pregnant women. Aust N Z J Obstet Gynaecol 2011;51:141–6.<br>10. Vinter CA, Jensen DM, Ovesen P, Beck-Nielsen H, Jørgensen JS. The LiP (Lifestyle in Pregnancy) study: a randomized controlled trial of lifestyle intervention in 360 obese pregnant women. Diabetes Care 2011;34:2502–7.<br>11. Asbee SM, Jenkins TR, Butler JR, White J, Elliot M, Rutledge A. Preventing excessive weight gain during pregnancy through dietary and lifestyle counseling: a randomized controlled trial. Obstet Gynecol 2009;113:305–12.<br>12. Bonomo M, Corica D, Mion E, Goncalves D, Motta G, Merati R, Ragusa A, Morabito A. Evaluating the therapeutic approach in pregnancies complicated by borderline glucose intolerance: a randomized clinical trial. Diabet Med 2005;22:1536–41.<br>13. Briley C, Flanagan NL, Lewis N. In-home prenatal nutrition intervention increased dietary iron intakes and reduced low birthweight in low-income African-American women. J Am Diet Assoc 2002;102:984–7.<br>14. Fard NM, Mehrabian F, Sarraf-Zadegan N, Sajadi F. Fat-modified diets during pregnancy and lactation and serum lipids after birth. Indian J Pediatr 2004;71:683–7.<br>15. Kafatos AG, Vlachonikolis I, Codrington C. Nutrition during pregnancy: the effects of an educational intervention program in Greece. Am J Clin Nutr 1989;50:970–9.<br>16. Khoury J, Henriksen T, Christophersen B, Tonstad S. Effect of a cholesterol-lowering diet on maternal, cord, and neonatal lipids, and pregnancy outcome: a randomized clinical trial. Am J Obstet Gynecol 2005;193:1292–301.<br>17. Knuist M, Bonsel GJ, Zondervan HA, Treffers PE. Low sodium diet and pregnancy-induced hypertension: a multi-centre randomised controlled trial. Br J Obstet Gynaecol 1998;105:430–4.<br>18. O'Connor MJ, Whaley SE. Brief intervention for alcohol use by pregnant women. Am J Public Health 2007;97:252–8.<br>19. Thornton YS, Smarkola C, Kopacz SM, Ishoof SB. Perinatal outcomes in nutritionally monitored obese pregnant women: a randomized clinical trial. J Natl Med Assoc 2009;101:569–77.<br>20. Van Buul BJA, Steegers EAP, Van Der Maten GD, Delemarre FMC, Jongsma HW, Oosterbaan HP, De Jong PA. Dietary sodium restriction does not prevent gestational hypertension: a Dutch two-center randomized trial. Hypertens Pregnancy 1997;16:335–46<br>21. Wolff S, Legarth J, Vangsgaard K, Toubro S, Astrup A. A randomized trial of the effects of dietary counseling on gestational weight gain and glucose metabolism in obese pregnant women. Int J Obes (Lond) 2008;32:495–501.<br>22. Bech BH, Obel C, Henriksen TB, Olsen J. Effect of reducing caffeine intake on birth weight and length of gestation: randomised controlled trial. BMJ 2007;334:409–12.<br>23. Ceesay SM, Prentice AM, Cole TJ, Foord F, Weaver LT, Poskitt EM, Whitehead RG. Effects on birth weight and perinatal mortality of maternal dietary supplements in rural Gambia: 5 year randomised controlled trial. BMJ 1997;315:786–90.<br>24. de Groot RHM, Hornstra G, van Houwelingen AC, Roumen F. Effect of alpha-linolenic acid supplementation during pregnancy on maternal and neonatal polyunsaturated fatty acid status and pregnancy outcome. Am J Clin Nutr 2004;79:251–60.<br>25. Kusin JA, Kardjati S, Houtkooper JM, Renqvist UH. Energy supplementation during pregnancy and postnatal growth. Lancet 1992;340:623–6.<br>26. McDonald EC, Pollitt E, Mueller W. The bacon chow study: maternal nutritional supplementation and birth weight of offspring. Am J Clin Nutr 1981;34:2133–44.<br>27. Metcoff J, Costiloe P, Crosby WM, Dutta S, Sandstead HH, Milne D, Bodwell CE, Majors SH. Effect of food supplementation (WIC) during pregnancy on birth weight. Am J Clin Nutr 1985;41:933–47.<br>28. Smuts CM, Huang M, Mundy D, Plasse T, Major S, Carlson SE. A randomized trial of docosahexaenoic acid supplementation during the third trimester of pregnancy. Obstet Gynecol 2003;101:469–79.<br>29. Aaltonen J, Ojala T, Laitinen K, Piirainen TJ, Poussa TA, Isolauri E. Evidence of infant blood pressure programming by maternal nutrition during pregnancy: a prospective randomized controlled intervention study. J Pediatr 2008;152:79–84.
Chatzakis *et al*. 2019 [43]	1. Ong MJ, Guelfi KJ, Hunter T, Wallman KE, Fournier PA, Newnham JP. Supervised home-based exercise may attenuate the decline of glucose tolerance in obese pregnant women. Diabetes Metab 2009;35:418–21.<br>2. Callaway LK, Colditz PB, Byrne NM, Lingwood BE, Rowlands IJ, Foxcroft K, et al. Prevention of gestational diabetes: Feasibility issues for an exercise intervention in obese pregnant women. Diabetes Care 2010;33:1457–9<br>3. Nascimento SL, Surita FG, Parpinelli M, Siani S, Pinto e Silva JL. The effect of an antenatal physical exercise programme on maternal/perinatal outcomes and quality of life in overweight and obese pregnant women: A randomised clinical trial. BJOG An Int J Obstet Gynaecol 2011;118:1455–63.<br>4. Oostdam N, van Poppel MNM, Wouters MGAJ, Eekhoff EMW, Bekedam DJ, Kuchenbecker WKH, et al. No effect of the FitFor2 exercise programme on blood glucose, insulin sensitivity, and birthweight in pregnant women who were overweight and at risk for gestational diabetes: results of a randomised controlled trial. BJOG 2012;119:1098–107

Review	References of included behavioural interventions delivered in pregnancy
	5. Kong KL, Campbell CG, Foster RC, Peterson AD, Lanningham- Foster L. A pilot walking program promotes moderate intensity physical activity during pregnancy. Med Sci Sports Exerc 2014;46:462–71.<br>6. Renault KM, Nørgaard K, Nilas L, Carlsen EM, Cortes D, Pryds O, et al. The Treatment of Obese Pregnant Women (TOP) study: A randomized controlled trial of the effect of physical activity intervention assessed by pedometer with or without dietary intervention in obese pregnant women. Am J Obstet Gynecol 2014;210:134.e1–9.<br>7. Bisson M, Alme´ras N, Dufresne SS, Robitaille J, Rhe´aume C, Bujold E, et al. A 12-week exercise program for pregnant women with obesity to improve physical activity levels: An open randomised preliminary study. PLoS ONE 2015;10:1–17. Dekker [45] Some concerns<br>8. Seneviratne SN, Jiang Y, Derraik JGB, McCowan LME, Parry GK, Biggs JB, et al. Effects of antenatal exercise in overweight and obese pregnant women on maternal and perinatal outcomes: A randomised controlled trial. BJOG An Int J Obstet Gynaecol 2016;123:588–97.<br>9. Garnæs KK, Mørkved S, Salvesen Ø, Moholdt T. Exercise training and weight gain in obese pregnant women: a randomized controlled trial (ETIP Trial). PLoS Med 2016;13:1–18.<br>10. Simmons D, Devlieger R, Van Assche A, Jans G, Galjaard S, Corcoy R, et al. Effect of physical activity and/or healthy eating on gdm risk: The dali lifestyle study. J Clin Endocrinol Metab 2017;102:903–13.<br>11. Wang C, Wei Y, Zhang X, Zhang Y, Xu Q, Sun Y, et al. A randomized clinical trial of exercise during pregnancy to prevent gestational diabetes mellitus and improve pregnancy outcome in overweight and obese pregnant women. Am J Obstet Gynecol 2017;216:340–51.<br>12. Daly N, Farren M, McKeating A, O'Kelly R, Stapleton M, Turner MJ. A medically supervised pregnancy exercise intervention in obese women: A randomized controlled trial. Obstet Gynecol 2017;130:1001–10.<br>13. Dekker Nitert M, Barrett HL, Denny KJ, McIntyre HD, Callaway LK. Exercise in pregnancy does not alter gestational weight gain, MCP-1 or leptin in obese women. Aust New Zeal J Obstet Gynaecol 2015;55:27–33. https://doi.org/10.1111/ajo.12300.<br>14. Chiswick C, Reynolds RM, Denison F, Drake AJ, Forbes S, Newby DE, et al. Effect of metformin on maternal and fetal outcomes in obese pregnant women (EMPOWaR): A randomised, double-blind, placebo-controlled trial. Lancet Diabetes Endocrinol 2015;3:778–86.<br>15. Syngelaki A, Nicolaides KH, Balani J, Hyer S, Akolekar R, Kotecha R, et al. Metformin versus placebo in obese pregnant women without diabetes mellitus. Obstet Gynecol Surv 2016;71:324–6.<br>16. Brink HSM, Alkemade AJ, van der Lely J, van der L. Metformin in women at high risk of gestational diabetes mellitus Introduction. Diabetes Metab 2018: 8–10.<br>17. Dodd JM, Louise J, Deussen AR, Grivell RM, Dekker G, McPhee AJ, et al. Effect of metformin in addition to dietary and lifestyle advice for pregnant women who are overweight or obese: the GRoW randomised, double-blind, placebo controlled trial. Lancet Diabetes Endocrinol 2019;7:15–24.<br>18. Lindsay K, Kennelly M, Smith T, Maguire O, Shanahan F, Brennan L, et al. PPO.19 Probiotics in obese pregnancy to reduce maternal fasting glucose: A randomised controlled trial. Arch Dis Child - Fetal Neonatal Ed 2014; 99: A156.1-A156.<br>19. Pellonpera¨ O, Mokkala K, Houttu N, Vahlberg T, Koivuniemi E, Tertti K, et al. Efficacy of fish oil and/or probiotic intervention on the incidence of gestational diabetes mellitus in an at-risk group of overweight and obese women: a randomized, placebo-controlled, double-blind clinical trial. Diabetes Care 2019;42:1009–17.<br>20. Okesene-Gafa KAM, Li M, McKinlay CJD, Taylor RS, Rush EC, Wall CR, et al. Effect of antenatal dietary interventions in maternal obesity on pregnancy weight-gain and birthweight: Healthy Mums and Babies (HUMBA) randomized trial. Am JObstet Gynecol 2019.<br>21. Asgharian H, Homayouni-Rad A, Mirghafourvand M, Mohammad-Alizadeh-Charandabi S. Effect of probiotic yoghurt on plasma glucose in overweight and obese pregnant women: a randomized controlled clinical trial. Eur J Nutr 2019.<br>22. Callaway LK, McIntyre HD, Barrett HL, Foxcroft K, Tremellen A, Lingwood BE, et al. Probiotics for the prevention of gestational diabetes mellitus in overweight and obese women: Findings from the SPRING double-blind randomized controlled trial. Diabetes Care 2019;42:364–71.<br>23. Corcoy R, Mendoza LC, Simmons D, Desoye G, Adelantado JM, Chico A, et al. The DALI vitamin D randomized controlled trial for gestational diabetes mellitus prevention: No major benefit shown besides vitamin D sufficiency. Clin Nutr 2019:1–9.
Craemer *et al.* 2019 [44]	1. Di Carlo C, Iannotti G, Sparice S et al.The role of a personalized dietary intervention in managing gestational weight gain: a prospective, controlled study in a low-risk antenatal population. Arch Gynecol Obstet. 2014;289(04):765–770. [PubMed] [Google Scholar]<br>2. Horan M K, McGowan C A, Gibney E R, Byrne J, Donnelly J M, McAuliffe F M. Maternal nutrition and glycaemic index during pregnancy impacts on offspring adiposity at 6 months of age analysis from the ROLO randomised controlled trial. Nutrients. 2016;8(01):13–15. [PMC free article] [PubMed] [Google Scholar]<br>3. Luo X-D, Dong X, Zhou J. Effects of nutritional management intervention on gestational weight gain and perinatal outcome. Saudi Med J. 2014;35(10):1267–1270. [PMC free article] [PubMed] [Google Scholar]<br>4. McGowan C A, Walsh J M, Byrne J, Curran S, McAuliffe F M. The influence of a low glycemic index dietary intervention on maternal dietary intake, glycemic index and gestational weight gain during pregnancy: a randomized controlled trial. Nutr J. 2013;12(01):140. [PMC free article] [PubMed] [Google Scholar]<br>5. Quinlivan J A, Lam L T, Fisher J. A randomised trial of a four-step multidisciplinary approach to the antenatal care of obese pregnant women. Aust N Z J Obstet Gynaecol. 2011;51(02):141–146. [PubMed] [Google Scholar]<br>6. Walsh J M, McAuliffe F M. Impact of maternal nutrition on pregnancy outcome--does it matter what pregnant women eat? Best Pract Res Clin Obstet Gynaecol. 2015;29(01):63–78. [PubMed] [Google Scholar]<br>7. Althuizen E, van der Wijden C L, van Mechelen W, Seidell J C, van Poppel M NM. The effect of a counselling intervention on weight changes during and after pregnancy: a randomised trial. BJOG. 2013;120(01):92–99. [PubMed] [Google Scholar]

Review	References of included behavioural interventions delivered in pregnancy
	8. Asbee S M, Jenkins T R, Butler J R, White J, Elliot M, Rutledge A.Preventing excessive weight gain during pregnancy through dietary and lifestyle counseling: a randomized controlled trial Obstet Gynecol 2009113(2 Pt 1):305–312. [PubMed] [Google Scholar]<br>9. Aşcı Ö, Rathfisch G. Effect of lifestyle interventions of pregnant women on their dietary habits, lifestyle behaviors, and weight gain: a randomized controlled trial. J Health Popul Nutr. 2016;35:7. [PMC free article] [PubMed] [Google Scholar]<br>10. Bogaerts A F, Devlieger R, Nuyts E, Witters I, Gyselaers W, Van den Bergh B RH. Effects of lifestyle intervention in obese pregnant women on gestational weight gain and mental health: a randomized controlled trial. Int J Obes. 2013;37(06):814–821. [PubMed] [Google Scholar]<br>11. Huang T T, Yeh C Y, Tsai Y C. A diet and physical activity intervention for preventing weight retention among Taiwanese childbearing women: a randomised controlled trial. Midwifery. 2011;27(02):257–264. [PubMed] [Google Scholar]<br>12. Hui A, Back L, Ludwig S et al.Lifestyle intervention on diet and exercise reduced excessive gestational weight gain in pregnant women under a randomised controlled trial. BJOG. 2012;119(01):70–77. [PubMed] [Google Scholar]<br>13. Hui A L, Back L, Ludwig S et al.Effects of lifestyle intervention on dietary intake, physical activity level, and gestational weight gain in pregnant women with different pre-pregnancy body mass index in a randomized control trial. BMC Pregnancy Childbirth. 2014;14:331. [PMC free article] [PubMed] [Google Scholar]<br>14. Korpi-Hyövälti E A, Laaksonen D E, Schwab U S et al.Feasibility of a lifestyle intervention in early pregnancy to prevent deterioration of glucose tolerance. BMC Public Health. 2011;11:179. [PMC free article] [PubMed] [Google Scholar]<br>15. Luoto R, Kinnunen T I, Aittasalo M et al.Primary prevention of gestational diabetes mellitus and large-for-gestational-age newborns by lifestyle counseling: a cluster-randomized controlled trial. PLoS Med. 2011;8(05):e1001036. [PMC free article] [PubMed] [Google Scholar]<br>16. Mustila T, Raitanen J, Keskinen P, Saari A, Luoto R. Lifestyle counseling during pregnancy and offspring weight development until four years of age: follow-up study of a controlled trial. J Negat Results Biomed. 2012;11:11. [PMC free article] [PubMed] [Google Scholar]<br>17. Petrella E, Malavolti M, Bertarini V et al.Gestational weight gain in overweight and obese women enrolled in a healthy lifestyle and eating habits program. J Matern Fetal Neonatal Med. 2014;27(13):1348–1352. [PubMed] [Google Scholar]<br>18. Phelan S, Phipps M G, Abrams B, Darroch F, Schaffner A, Wing R R. Randomized trial of a behavioral intervention to prevent excessive gestational weight gain: the Fit for Delivery Study. Am J Clin Nutr. 2011;93(04):772–779. [PMC free article] [PubMed] [Google Scholar]<br>19. Poston L, Bell R, Croker H et al.Effect of a behavioural intervention in obese pregnant women (the UPBEAT study): a multicentre, randomised controlled trial. Lancet Diabetes Endocrinol. 2015;3(10):767–777. [PubMed] [Google Scholar] Rauh K, Gabriel E, Kerschbaum E et al.Safety and efficacy of a lifestyle intervention for pregnant women to prevent excessive maternal weight gain: a cluster-randomized controlled trial. BMC Pregnancy Childbirth. 2013;13:151. [PMC free article] [PubMed] [Google Scholar]<br>20. Renault K M, Nørgaard K, Nilas L et al.The Treatment of Obese Pregnant Women (TOP) study: a randomized controlled trial of the effect of physical activity intervention assessed by pedometer with or without dietary intervention in obese pregnant women. Am J Obstet Gynecol. 2014;210(02):1340–1.34E11. [PubMed] [Google Scholar]<br>21. Ruchat S M, Davenport M H, Giroux I et al.Nutrition and exercise reduce excessive weight gain in normal-weight pregnant women. Med Sci Sports Exerc. 2012;44(08):1419–1426. [PubMed] [Google Scholar]<br>22. Szmeja M A, Cramp C, Grivell R M, Deussen A R, Yelland L N, Dodd J M. Use of a DVD to provide dietary and lifestyle information to pregnant women who are overweight or obese: a nested randomised trial. BMC Pregnancy Childbirth. 2014;14:409. [PMC free article] [PubMed] [Google Scholar]<br>23. Tanvig M, Vinter C A, Jørgensen J S et al.Effects of lifestyle intervention in pregnancy and anthropometrics at birth on offspring metabolic profile at 2.8 years: results from the Lifestyle in Pregnancy and Offspring (LiPO) study. J Clin Endocrinol Metab. 2015;100(01):175–183. [PubMed] [Google Scholar]<br>24. Vesco K K, Karanja N, King J C et al.Efficacy of a group-based dietary intervention for limiting gestational weight gain among obese women: a randomized trial. Obesity (Silver Spring) 2014;22(09):1989–1996. [PMC free article] [PubMed] [Google Scholar]<br>25. Byrne N M, Groves A M, McIntyre H D, Callaway L K; BAMBINO group.Changes in resting and walking energy expenditure and walking speed during pregnancy in obese women Am J Clin Nutr 20119403819–830. [PubMed] [Google Scholar]<br>26. Garnæs K K, Mørkved S, Salvesen Ø, Moholdt T. Exercise training and weight gain in obese pregnant women: a randomized controlled trial (ETIP trial) PLoS Med. 2016;13(07):e1002079. [PMC free article] [PubMed] [Google Scholar]<br>27. Haakstad L A, Bø K. Effect of regular exercise on prevention of excessive weight gain in pregnancy: a randomised controlled trial. Eur J Contracept Reprod Health Care. 2011;16(02):116–125. [PubMed] [Google Scholar]<br>28. Kong K L, Campbell C G, Foster R C, Peterson A D, Lanningham-Foster L. A pilot walking program promotes moderate-intensity physical activity during pregnancy. Med Sci Sports Exerc. 2014;46(03):462–471. [PubMed] [Google Scholar]<br>29. Nascimento S L, Surita F G, Parpinelli MÂ, Siani S, Pinto e Silva J L. The effect of an antenatal physical exercise programme on maternal/perinatal outcomes and quality of life in overweight and obese pregnant women: a randomised clinical trial. BJOG. 2011;118(12):1455–1463. [PubMed] [Google Scholar]

Review	References of included behavioural interventions delivered in pregnancy
	30. Ruiz J R, Perales M, Pelaez M, Lopez C, Lucia A, Barakat R. Supervised exercise-based intervention to prevent excessive gestational weight gain: a randomized controlled trial. Mayo Clin Proc. 2013;88(12):1388–1397. [PubMed] [Google Scholar]
Davenport *et al.* 2019a [45]	1. Guelinckx I, Devlieger R, Mullie P, et al. Effect of lifestyle intervention on dietary habits, physical activity, and gestational weight gain in obese pregnant women: a randomized controlled trial. Am J Clin Nutr 2010;91:373–80.<br>2. Luoto R, Kinnunen TI, Aittasalo M, et al. Primary prevention of gestational diabetes mellitus and large-for-gestational-age newborns by lifestyle counseling: a cluster randomized controlled trial. PLoS Med 2011;8:e1001036.<br>3. Vinter CA, Jensen DM, Ovesen P, et al. The LiP (Lifestyle in Pregnancy) study: a randomized controlled trial of lifestyle intervention in 360 obese pregnant women. Diabetes Care 2011;34:2502–7.<br>4. Renault KM, Nørgaard K, Nilas L, et al. The Treatment of Obese Pregnant Women (TOP) study: a randomized controlled trial of the effect of physical activity intervention assessed by pedometer with or without dietary intervention in obese pregnant women. Am J Obstet Gynecol 2014;210:134.e1–e9.<br>5. Koivusalo SB, Rönö K, Klemetti MM, et al. Gestational diabetes mellitus can be prevented by lifestyle intervention: the finnish gestational diabetes prevention study (RADIEL): a randomized controlled trial. Diabetes Care 2016;39:24–30.<br>6. Poston L, Bell R, Croker H, et al. Effect of a behavioural intervention in obese pregnant women (the UPBEAT study): a multicentre, randomised controlled trial. Lancet Diabetes Endocrinol 2015;3:767–77.<br>7. Jing W, Huang Y, Liu X, et al. The effect of a personalized intervention on weight gain and physical activity among pregnant women in China. Int J Gynaecol Obstet 2015;129:138–41.<br>8. Korpi-Hyövälti E, Heinonen S, Schwab U, et al. Effect of intensive counselling on physical activity in pregnant women at high risk for gestational diabetes mellitus. A clinical study in primary care. Prim Care Diabetes 2012;6:261–8.<br>9. Jackson RA, Stotland NE, Caughey AB, et al. Improving diet and exercise in pregnancy with video doctor counseling: a randomized trial. Patient Educ Couns 2011;83:203–9.<br>10. Hawkins M, Hosker M, Marcus BH, et al. A pregnancy lifestyle intervention to prevent gestational diabetes risk factors in overweight Hispanic women: a feasibility randomized controlled trial. Diabet Med 2015;32:108–15.<br>11. Polley BA, Wing RR, Sims CJ. Randomized controlled trial to prevent excessive weight gain in pregnant women. Int J Obes Relat Metab Disord 2002;26:1494–502.<br>12. Phelan S, Phipps MG, Abrams B, et al. Randomized trial of a behavioral intervention to prevent excessive gestational weight gain: the Fit for Delivery Study. Am J Clin Nutr 2011;93:772–9.<br>13. Kluge J, Hall D, Louw Q, et al. Specific exercises to treat pregnancy-related low back pain in a South African population. Int J Gynaecol Obstet 2011;113:187–91.<br>14. Sagedal LR, Vistad I, Øverby NC, et al. The effect of a prenatal lifestyle intervention on glucose metabolism: results of the Norwegian fit for delivery randomized controlled trial. BMC Pregnancy Childbirth 2017;17:167.<br>15. Bisson M, Alméras N, Dufresne SS, et al. A 12-week exercise program for pregnant women with obesity to improve physical activity levels: an open randomised preliminary study. PLoS One 2015;10:e0137742.<br>16. Petrov Fieril K, Glantz A, Fagevik Olsen M. The efficacy of moderate-to-vigorous resistance exercise during pregnancy: a randomized controlled trial. Acta Obstet Gynecol Scand 2015;94:35–42.<br>17. Prabhu N. Effect of a exercise by pregnant women and birth weight: a randomized controlled trial. Journal of Evolution of Medical and Dental Sciences 2015;04:1509–<br>18. Ronnberg AK, Ostlund I, Fadl H, et al. Intervention during pregnancy to reduce excessive gestational weight gain—a randomised controlled trial. BJOG 2015;122:537–44.<br>19. Kihlstrand M, Stenman B, Nilsson S, et al. Water-gymnastics reduced the intensity of back/low back pain in pregnant women. Acta Obstet Gynecol Scand 1999;78:180–5.<br>20. Ussher M, Lewis S, Aveyard P, et al. Physical activity for smoking cessation in pregnancy: randomised controlled trial. BMJ 2015;350:h2145.<br>21. Guelfi KJ, Ong MJ, Crisp NA, et al. Regular exercise to prevent the recurrence of gestational diabetes mellitus: a randomized controlled trial. Obstet Gynecol 2016;128:819–27.<br>22. Dumas GA, Reid JG, Wolfe LA, et al. Exercise, posture, and back pain during pregnancy. Clin Biomech 1995;10:98–103.<br>23. Haby K, Glantz A, Hanas R, et al. Mighty mums - an antenatal health care intervention can reduce gestational weight gain in women with obesity. Midwifery 2015;31:685–92.<br>24. Sun Y, Zhao H. The effectiveness of lifestyle intervention in early pregnancy to prevent gestational diabetes mellitus in Chinese overweight and obese women: a quasiexperimental study. Appl Nurs Res 2016;30:125–30.<br>25. Magann EF, Evans SF, Weitz B, et al. Antepartum, intrapartum, and neonatal significance of exercise on healthy low-risk pregnant working women. Obstet Gynecol 2002;99:466–72.<br>26. Madsen M, Jørgensen T, Jensen ML, et al. Leisure time physical exercise during pregnancy and the risk of miscarriage: a study within the Danish National Birth Cohort. BJOG 2007;114:1419–26.<br>27. Feodor Nilsson S, Andersen PK, Strandberg-Larsen K, et al. Risk factors for miscarriage from a prevention perspective: a nationwide follow-up study. BJOG 2014;121:1375–85.<br>28. Zhang X, Li J, Gu Y, et al. A pilot study on environmental and behavioral factors related to missed abortion. Environ Health Prev Med 2011;16:273–8.<br>29. Maconochie N, Doyle P, Prior S, et al. Risk factors for first trimester miscarriage-- results from a UK-population-based case-control study. BJOG 2007;114:170–86.<br>30. Rauh K, Gabriel E, Kerschbaum E, et al. Safety and efficacy of a lifestyle intervention for pregnant women to prevent excessive maternal weight gain: a cluster-randomized controlled trial. BMC Pregnancy Childbirth 2013;13:151.<br>31. Hollingsworth DR, Moore TR. Postprandial walking exercise in pregnant insulin dependent (type I) diabetic women: reduction of plasma lipid levels but absence of a significant effect on glycemic control. Am J Obstet Gynecol 1987;157:1359–63.<br>32. Dodd JM, Cramp C, Sui Z, et al. The effects of antenatal dietary and lifestyle advice for women who are overweight or obese on maternal diet and physical activity: the LIMIT randomised trial. BMC Med 2014;12:161.<br>33. Elden H, Ostgaard HC, Fagevik-Olsen M, et al. Treatments of pelvic girdle pain in pregnant women: adverse effects of standard treatment, acupuncture and stabilising exercises on the pregnancy, mother, delivery and the fetus/neonate. BMC Complement Altern Med 2008;8:34.<br>34. Seneviratne SN, Jiang Y, Derraik J, et al. Effects of antenatal exercise in overweight and obese pregnant women on maternal and perinatal outcomes: a randomised controlled trial. BJOG 2016;123:588–97.

Review	References of included behavioural interventions delivered in pregnancy
	35. Taniguchi C, Sato C. Home-based walking during pregnancy affects mood and birth outcomes among sedentary women: A randomized controlled trial. Int J Nurs Pract 2016;22:420–6.<br>36. Jovanovic-Peterson L, Durak EP, Peterson CM. Randomized trial of diet versus diet plus cardiovascular conditioning on glucose levels in gestational diabetes. Am J Obstet Gynecol 1989;161:415–9<br>37. Lawani MM, Alihonou E, Akplogan B, et al. [Effect of antenatal gymnastics on childbirth: a study on 50 sedentary women in the Republic of Benin during the second and third quarters of pregnancy]. Sante 2003;13:235–41.<br>38. Collings CA, Curet LB, Mullin JP. Maternal and fetal responses to a maternal aerobic exercise program. Am J Obstet Gynecol 1983;145:702–7.<br>39. Denda OS, Dalkiranis AX, Denda MS, et al. The effect of some dietary components on body weight and fat deposition in pregnant women in combination with exercise during pregnancy and postpartum period. Studies in Physical Culture and Tourism 2005;12.<br>40. Abirami P, Judie A. Integrated approach of yoga therapy on maternal and fetal outcome in gestational diabetes mellitus. International Journal of Pharmaceutical and Clinical Research 2015;7:377–82.<br>41. Lombardi W, Wilson S, Peniston PB. Wellness intervention with pregnant soldiers. Mil Med 1999;164:22–9.<br>42. Magnus P, Trogstad L, Owe KM, et al. Recreational physical activity and the risk of preeclampsia: a prospective cohort of Norwegian women. Am J Epidemiol 2008;168:952–7.<br>43. Dumith SC, Domingues MR, Mendoza-Sassi RA, et al. Physical activity during pregnancy and its association with maternal and child health indicators. Rev Saude Publica 2012;46:327–33.<br>44. Xu G, Wu Y, Yang L, et al. Risk factors for early miscarriage among Chinese: a hospitalbased case-control study. Fertil Steril 2014;101:1663–70<br>45. Erkkola, R. "The influence of physical training during pregnancy on physical work capacity and circulatory parameters." Scandinavian journal of clinical and laboratory investigation 36.8 (1976): 747-754.<br>46. Garnæs, K. K., Mørkved, S., Salvesen, Ø., & Moholdt, T. (2016). Exercise training and weight gain in obese pregnant women: a randomized controlled trial (ETIP trial). PLoS medicine, 13(7).
Davenport *et al.* 2018a [46]	1. Barakat R, Perales M, Bacchi M, Coteron J, Refoyo I. A program of exercise throughout pregnancy. Is it safe to mother and newborn? American Journal of Health Promotion. 2014;29(1):2-8.<br>2. Davis K, Goodman SH, Leiferman J, Taylor M, Dimidjian S. A randomized controlled trial of yoga for pregnant women with symptoms of depression and anxiety. Complement Ther Clin Pract. 2015;21(3):166-72.<br>3. Field T, Diego M, Delgado J, Medina L. Tai chi/yoga reduces prenatal depression, anxiety and sleep disturbances. Complement Ther Clin Pract. 2013;19(1):6-10.<br>4. Field T, Diego M, Hernandez-Reif M, Medina L, Delgado J, Hernandez A. NIHMS326920 PMC3319349; Yoga and massage therapy reduce prenatal depression and prematurity. Journal of Bodywork & Movement Therapies. 2012;16(2):204.<br>5. Guelfi KJ, Ong MJ, Crisp NA, Fournier PA, Wallman KE, Grove JR, et al. Regular Exercise to Prevent the Recurrence of Gestational Diabetes Mellitus: A Randomized Controlled Trial. Obstet Gynecol. 2016;128(4):819-27.<br>6. Gustafsson MK, Stafne SN, Romundstad PR, Morkved S, Salvesen K, Helvik AS. The effects of an exercise programme during pregnancy on health-related quality of life in pregnant women: a Norwegian randomised controlled trial. BJOG. 2016;123(7):1152-60.<br>7. Jennifer M, Tiffany F, Miguel D, Debra B, Rae N, Martha P. Yoga reduces prenatal depression symptoms. Complement Ther Clin Pract. 2012;19(1):6.<br>8. Lisa AU, Cynthia LB, Kaeli AS, Susanna RM, Ivan WM. A pilot randomized controlled trial comparing prenatal yoga to perinatal health education for antenatal depression. Archives of women's mental health. 2015;19(3):543.<br>9. Newham JJ, Wittkowski A, Hurley J, Aplin JD, Westwood M. Effects of antenatal yoga on maternal anxiety and depression: a randomized controlled trial. Depress Anxiety. 2014;31(8):631-40.<br>10. Perales M, Cordero Y, Vargas M, Lucia A, Barakat R. Exercise and depression in overweight and obese pregnant women: A randomised controlled trial. Archivos de Medicina del Deporte. 2015;32:70-7.<br>11. Perales M, Mateos S, Vargas M, Sanz I, Lucia A, Barakat R. Fetal and maternal heart rate responses to exercise in pregnant women. A randomized controlled trial. Archivos de Medicina del Deporte. 2015;32(6):361-7.<br>12. Robledo-Colonia AF, Sandoval-Restrepo N, Mosquera-Valderrama YF, Escobar-Hurtado C, Ramirez-Velez R. Aerobic exercise training during pregnancy reduces depressive symptoms in nulliparous women: a randomised trial. J Physiother. 2012;58(1):9-15.<br>13. Satyapriya M, Nagarathna R, Padmalatha V, Nagendra HR. Effect of integrated yoga on anxiety, depression & well being in normal pregnancy. 2013.<br>14. Taniguchi C, Sato C. Home-based walking during pregnancy affects mood and birth outcomes among sedentary women: A randomized controlled trial. Int J Nurs Pract. 2016;22(5):420-6.<br>15. Choi J, Lee JH, Vittinghoff E, Fukuoka Y. NIHMS743852 Available on 05/01/17] PMC4826820 Available on 05/01/17]; mHealth Physical Activity Intervention: A Randomized Pilot Study in Physically Inactive Pregnant Women. Maternal & Child Health Journal. 2016;20(5):1091-101.<br>16. Dodd JM, Newman A, Moran LJ, et al. The effect of antenatal dietary and lifestyle advice for women who are overweight or obese on emotional well-being: the LIMIT randomized trial. Acta ObstetGynecolScand. 2016;95(3):309.<br>17. Phelan S, Phipps MG, Abrams B, Darroch F, Grantham K, Schaffner A, et al. Does behavioral intervention in pregnancy reduce postpartum weight retention? Twelve-month outcomes of the Fit for Delivery randomized trial. Am J Clin Nutr. 2014;99(2):302-11.<br>18. Poston L, Briley AL, Barr S, Bell R, Croker H, Coxon K, et al. Developing a complex intervention for diet and activity behaviour change in obese pregnant women (the UPBEAT trial); assessment of behavioural change and process evaluation in a pilot randomised controlled trial. BMC Pregnancy Childbirth. 2013;13:148.<br>19. Ussher M, Lewis S, Aveyard P, et al. Physical activity for smoking cessation in pregnancy: randomised controlled trial. British Medical Journal. 2015;350:h2145.<br>20. Vieten C, Astin J. Effects of a mindfulness-based intervention during pregnancy on prenatal stress and mood: results of a pilot study. Arch Womens Ment Health. 2008;11(1):67-74.<br>21. Field T, Diego M, Delgado J, Medina L. Yoga and social support reduce prenatal depression, anxiety and cortisol. J Bodyw Mov Ther. 2013;17(4):397-403.<br>22. Satyapriya M, Nagarathna R, Padmalatha V, Nagendra HR. Effect of integrated yoga on anxiety, depression & well being in normal pregnancy. Complement Ther Clin Pract. 2013;19(4):230-6.<br>23. Haakstad LA, Torset B, Bo K. What is the effect of regular group exercise on maternal psychological outcomes and common pregnancy complaints? An assessor blinded RCT. Midwifery. 2016;32:81-6.

Review	References of included behavioural interventions delivered in pregnancy
	24. Mohammadi F, Malakooti J, Babapoor J, Mohammad-Alizadeh-Charandabi S. The effect of a home-based exercise intervention on postnatal depression and fatigue: A randomized controlled trial. Int J Nurs Pract. 2015;21(5):478-85.<br>25. Savitha Pramilda C, Raj G, Rani GS. Effect of Antenatal Yoga on Postnatal Depressive Symptoms among the Primipara Women of a Selected Hospital. International Journal of Nursing Education. 2016;8(1):182.<br>26. Songoygard KM, Stafne SN, Evensen KA, Salvesen KA, Vik T, Morkved S. Does exercise during pregnancy prevent postnatal depression? A randomized controlled trial. Acta Obstet Gynecol Scand. 2012;91(1):62-7.<br>27. Miquelutti MA, Cecatti JG, Makuch MY. Evaluation of a birth preparation program on lumbopelvic pain, urinary incontinence, anxiety and exercise: a randomized controlled trial. BMC Pregnancy Childbirth. 2013;13:154.<br>28. El-Rafie M, Khafagy GM, Gamal MG. Effect of aerobic exercise during pregnancy on antenatal depression. International Journal of Women's Health. 2016;8:53.<br>29. Guszkowska M, Langwald M, Sempolska K. Does physical exercise help maintain mental health during pregnancy? A comparison of changes in mental health in participants of physical exercise classes and childbirth classes. Journal of Physical Activity & Health. 2015;12(1):30.<br>30. Kalayeh SMO, Nikbakht R, Seddigi S. The effect of exercise during pregnancy on mother's depression in Zahedan. International Journal of Pharmacy and Technology. 2016.<br>31. Claesson IM, Klein S, Sydsjo G, Josefsson A. Physical activity and psychological well-being in obese pregnant and postpartum women attending a weight-gain restriction programme. Midwifery. 2014;30(1):11.<br>32. Battle CL, Uebelacker LA, Magee SR, Sutton KA, Miller IW. Potential for prenatal yoga to serve as an intervention to treat depression during pregnancy. Womens Health Issues. 2015;25(2):134.<br>33. Beddoe AE, Yang CPP, Kennedy HP, Weiss SJ, Lee KA. The effects of mindfulness-based yoga during pregnancy on maternal psychological and physical distress. Journal of obstetric, gynecologic, and neonatal nursing : JOGNN. 2009;38(3):310.<br>34. Beddoe AE, Yang CPP, Kennedy HP, Weiss SJ, Lee KA. The effects of mindfulness-based yoga during pregnancy on maternal psychological and physical distress. Journal of obstetric, gynecologic, and neonatal nursing : JOGNN. 2009;38(3):310.<br>35. Shim MJ, Lee YS, Oh HE, Kim JS. Effects of a back-pain-reducing program during pregnancy for Korean women: a non-equivalent control-group pretest-posttest study. Int J Nurs Stud. 2007;44(1):19-28.<br>36. Gjestland K, Bo K, Owe KM, Eberhard-Gran M. Do pregnant women follow exercise guidelines? Prevalence data among 3482 women, and prediction of low-back pain, pelvic girdle pain and depression. Brit J Sport Med. 2013;47(8):515.<br>37. Demissie Z, Siega-Riz AM, Evenson KR, Herring AH, Dole N, Gaynes BN. Physical activity and depressive symptoms among pregnant women: the PIN3 study. Arch Womens Ment Health 2011;14(12):145-57.<br>38. Orr ST, James SA, Garry J, Prince CB, Newton ER. Exercise and pregnancy outcome among urban, low-income, black women. EthnDis. 2006;16(4):933-7.<br>39. Downs DS, DiNallo JM, Kirner TL. Determinants of pregnancy and postpartum depression: prospective influences of depressive symptoms, body image satisfaction, and exercise behavior. Ann Behav Med. 2008;36(1):54-63.<br>40. Tendais I, Figueiredo B, Mota J, Conde A. Physical activity, health-related quality of life and depression during pregnancy. Cad Saude Publica. 2011;27(2):219-28.<br>41. Gaston A, Prapavessis H. Tired, moody and pregnant? Exercise may be the answer. Psychol Health. 2013;28(12):1353-69.<br>42. Kolu P, Raitanen J, Luoto R. Physical activity and health-related quality of life during pregnancy: a secondary analysis of a cluster-randomised trial. Matern Child Health J. 2014;18(9):2098-105.<br>43. Loprinzi PD, Loprinzi KL, Cardinal BJ. The relationship between physical activity and sleep among pregnant women. 2012.<br>44. Monika G. PHYSICAL ACTIVITY IN RELATION TO AFFECTIVE STATES AND LABOR ANXIETY IN PREGNANT WOMEN. Medicina Sportiva 2011;15(3):114-8.<br>45. Petrovic D, Perovic M, Lazovic B, Pantic I. Association between walking, dysphoric mood and anxiety in late pregnancy: A cross-sectional study. Psychiatry Res. 2016;246:360-3.<br>46. de Wit L, Jelsma JG, van Poppel MN, Bogaerts A, Simmons D, Desoye G, et al. Physical activity, depressed mood and pregnancy worries in European obese pregnant women: results from the DALI study. BMC Pregnancy Childbirth. 2015;15:158.<br>47. Bershadsky S, Trumpfheller L, Kimble HB, Pipaloff D, Yim IS. The effect of prenatal Hatha yoga on affect, cortisol and depressive symptoms. Complement Ther Clin Pract. 2014;20(2):106.<br>48. Costa DD, Rippen N, Dritsa M, Ring A. Self-reported leisure-time physical activity during pregnancy and relationship to psychological well-being. J Psychosom Obstet Gynaecol. 2003;24(2):111-9.<br>49. Goodwin A, Astbury J, McMeeken J. Body image and psychological well-being in pregnancy. A comparison of exercisers and non-exercisers. Aust N Z J Obstet Gynaecol. 2000;40(4):442-7.<br>50. Bowen A, Stewart N, Baetz M, Muhajarine N. Antenatal depression in socially high-risk women in Canada. Journal of Epidemiology & Community Health 2009;63(5):414-6.<br>51. Ersek JL, Brunner Huber LR. Physical activity prior to and during pregnancy and risk of postpartum depressive symptoms. J Obstet Gynecol Neonatal Nurs. 2009;38(5):556-66.<br>52. Strom M, Mortensen EL, Halldorson TI, Osterdal ML, Olsen SF. Leisure-time physical activity in pregnancy and risk of postpartum depression: a prospective study in a large national birth cohort. J Clin Psychiatry. 2009;70(12):1707-14.<br>53. Sexton MB, Flynn HA, Lancaster C, et al. Predictors of recovery from prenatal depressive symptoms from pregnancy through postpartum. 2012.<br>54. Jennifer G, Swathy S, Jenn L. Antenatal physical activity: Investigating the effects on postpartum depression. 2012.<br>55. Perales M, Santos-Lozano A, Sanchis-Gomar F, Luaces M, Pareja-Galeano H, Garatachea N, et al. Maternal Cardiac Adaptations to a Physical Exercise Program during Pregnancy. Med Sci Sports Exerc. 2016;48(5):896-906.
Davenport *et al.* 2018b [47]	1. Barakat R, Ruiz JR, Stirling JR, Zakynthinaki M, Lucia A. Type of delivery is not affected by light resistance and toning exercise training during pregnancy: a randomized controlled trial. Am J Obstet Gynecol. 2009;201(6):590 e1-6.<br>2. Elden H, Ostgaard HC, Fagevik-Olsen M, Ladfors L, Hagberg H. Treatments of pelvic girdle pain in pregnant women: adverse effects of standard treatment, acupuncture and stabilising exercises on the pregnancy, mother, delivery and the fetus/neonate. BMC Complement Altern Med. 2008;8:34.

Review	References of included behavioural interventions delivered in pregnancy
	3. Barakat R, Cordero Y, Coteron J, Luaces M, Montejo R. Exercise during pregnancy improves maternal glucose screen at 24-28 weeks: a randomised controlled trial. Br J Sports Med. 2012;46(9):656-61.<br>4. Price BB, Amini SB, Kappeler K. Exercise in pregnancy: effect on fitness and obstetric outcomes-a randomized trial. Med Sci Sports Exerc. 2012;44(12):2263-9.<br>5. Cordero Y, Peláez M, De Miguel M, Perales M, Barakat R. Can moderate physical exercise during pregnancy act as a factor in preventing Gestational Diabetes? Revista Internacional de Ciencias del Deporte. 2012;27(8):3-19.<br>6. Barakat R, Pelaez M, Lopez C, Lucia A, Ruiz JR. Exercise during pregnancy and gestational diabetes-related adverse effects: a randomised controlled trial. Br J Sports Med. 2013;47(10):630-6.<br>7. Barakat R, Perales M, Bacchi M, Coteron J, Refoyo I. A program of exercise throughout pregnancy. Is it safe to mother and newborn? Am J Health Promot. 2014;29(1):2-8.<br>8. Bisson M, Almeras N, Dufresne SS, Robitaille J, Rheaume C, Bujold E, et al. A 12-Week Exercise Program for Pregnant Women with Obesity to Improve Physical Activity Levels: An Open Randomised Preliminary Study. PLoS One. 2015;10(9):e0137742.<br>9. Cordero Y, Mottola MF, Vargas J, Blanco M, Barakat R. Exercise Is Associated with a Reduction in Gestational Diabetes Mellitus. Med Sci Sports Exerc. 2015;47(7):1328-33.<br>10. Kong KL, Campbell CG, Foster RC, Peterson AD, Lanningham-Foster L. A pilot walking program promotes moderate-intensity physical activity during pregnancy. Med Sci Sports Exerc. 2014;46(3):462-71.<br>11. Nobles C, Marcus BH, Stanek EJ, 3rd, Braun B, Whitcomb BW, Solomon CG, et al. Effect of an exercise intervention on gestational diabetes mellitus: a randomized controlled trial. Obstet Gynecol. 2015;125(5):1195-204.<br>12. Okido MM, Valeri FL, Martins WP, Ferreira CH, Duarte G, Cavalli RC. Assessment of foetal wellbeing in pregnant women subjected to pelvic floor muscle training: a controlled randomised study. Int Urogynecol J. 2015;26(10):1475-81.<br>13. Ruiz JR, Perales M, Pelaez M, Lopez C, Lucia A, Barakat R. Supervised exercise-based intervention to prevent excessive gestational weight gain: a randomized controlled trial. Mayo Clin Proc. 2013;88(12):1388-97.<br>14. Renault KM, Norgaard K, Nilas L, Carlsen EM, Cortes D, Pryds O, et al. The Treatment of Obese Pregnant Women (TOP) study: a randomized controlled trial of the effect of physical activity intervention assessed by pedometer with or without dietary intervention in obese pregnant women. Am J Obstet Gynecol. 2014;210(2):134 e1-9.<br>15. Tomic V, Sporis G, Tomic J, Milanovic Z, Zigmundovac-Klaic D, Pantelic S. The effect of maternal exercise during pregnancy on abnormal fetal growth. Croat Med J. 2013;54(4):362-8.<br>16. Oostdam N, van Poppel MN, Wouters MG, Eekhoff EM, Bekedam DJ, Kuchenbecker WK, et al. No effect of the FitFor2 exercise programme on blood glucose, insulin sensitivity, and birthweight in pregnant women who were overweight and at risk for gestational diabetes: results of a randomised controlled trial. BJOG. 2012;119(9):1098-107.<br>17. Seneviratne SN, Jiang Y, Derraik J, McCowan L, Parry GK, Biggs JB, et al. Effects of antenatal exercise in overweight and obese pregnant women on maternal and perinatal outcomes: a randomised controlled trial. BJOG. 2016;123(4):588-97.<br>18. Ussher M, Lewis S, Aveyard P, Manyonda I, West R, Lewis B, et al. The London Exercise And Pregnant smokers (LEAP) trial: a randomised controlled trial of physical activity for smoking cessation in pregnancy with an economic evaluation. Health Technol Assess. 2015;19(84):vii-xxiv, 1-135.<br>19. Ko CW, Napolitano PG, Lee SP, Schulte SD, Ciol MA, Beresford SA. Physical activity, maternal metabolic measures, and the incidence of gallbladder sludge or stones during pregnancy: a randomized trial. Am J Perinatol. 2014;31(1):39-48.<br>20. Callaway LK, Colditz PB, Byrne NM, Lingwood BE, Rowlands IJ, Foxcroft K, et al. Prevention of gestational diabetes: feasibility issues for an exercise intervention in obese pregnant women. Diabetes Care. 2010;33(7):1457-9.<br>21. Simmons D, Jelsma JG, Galjaard S, Devlieger R, van Assche A, Jans G, et al. Results From a European Multicenter Randomized Trial of Physical Activity and/or Healthy Eating to Reduce the Risk of Gestational Diabetes Mellitus: The DALI Lifestyle Pilot. Diabetes Care. 2015;38(9):1650-6.<br>22. Barakat R, Pelaez M, Cordero Y, Perales M, Lopez C, Coteron J, et al. Exercise during pregnancy protects against hypertension and macrosomia: randomized clinical trial. Am J Obstet Gynecol. 2016;214(5):649 e1-8.<br>23. Garnaes KK, Morkved S, Salvesen O, Moholdt T. Exercise Training and Weight Gain in Obese Pregnant Women: A Randomized Controlled Trial (ETIP Trial). PLoS Med. 2016;13(7):e1002079.<br>24. Guelfi KJ, Ong MJ, Crisp NA, Fournier PA, Wallman KE, Grove JR, et al. Regular Exercise to Prevent the Recurrence of Gestational Diabetes Mellitus: A Randomized Controlled Trial. Obstet Gynecol. 2016;128(4):819-27.<br>25. Wang C, Wei Y, Zhang X, Zhang Y, Xu Q, Su S, et al. Effect of Regular Exercise Commenced in Early Pregnancy on the Incidence of Gestational Diabetes Mellitus in Overweight and Obese Pregnant Women: A Randomized Controlled Trial. Diabetes Care. 2016;39(10):e163-4.<br>26. Simmons D, Devlieger R, van Assche A, Jans G, Galjaard S, Corcoy R, et al. Effect of Physical Activity and/or Healthy Eating on GDM Risk: The DALI Lifestyle Study. J Clin Endocrinol Metab. 2017;102(3):903-13.<br>27. Hui AL, Ludwig SM, Gardiner P, Sevenhuysen G, Murray R, Morris M, et al. Community-based exercise and dietary intervention during pregnancy: a pilot study. Canadian journal of diabetes. 2006;30(2):169-75.<br>28. Guelinckx I, Devlieger R, Mullie P, Vansant G. Effect of lifestyle intervention on dietary habits, physical activity, and gestational weight gain in obese pregnant women: a randomized controlled trial. Am J Clin Nutr. 2010;91(2):373-80.<br>29. Hui A, Back L, Ludwig S, Gardiner P, Sevenhuysen G, Dean H, et al. Lifestyle intervention on diet and exercise reduced excessive gestational weight gain in pregnant women under a randomised controlled trial. BJOG. 2012;119(1):70-7.<br>30. Luoto R, Kinnunen TI, Aittasalo M, Kolu P, Raitanen J, Ojala K, et al. Primary prevention of gestational diabetes mellitus and large-for-gestational-age newborns by lifestyle counseling: a cluster-randomized controlled trial. PLoS Med. 2011;8(5):e1001036.<br>31. Stafne SN, Salvesen KA, Romundstad PR, Eggebo TM, Carlsen SM, Morkved S. Regular exercise during pregnancy to prevent gestational diabetes: a randomized controlled trial. Obstet Gynecol. 2012;119(1):29-36.<br>32. Vinter CA, Jensen DM, Ovesen P, Beck-Nielsen H, Jorgensen JS. The LiP (Lifestyle in Pregnancy) study: a randomized controlled trial of lifestyle intervention in 360 obese pregnant women. Diabetes Care. 2011;34(12):2502-7.

Review	References of included behavioural interventions delivered in pregnancy
	33. Hui AL, Back L, Ludwig S, Gardiner P, Sevenhuysen G, Dean HJ, et al. Effects of lifestyle intervention on dietary intake, physical activity level, and gestational weight gain in pregnant women with different pre-pregnancy Body Mass Index in a randomized control trial. BMC Pregnancy Childbirth. 2014;14:331.<br>34. Rauh K, Gabriel E, Kerschbaum E, Schuster T, von Kries R, Amann-Gassner U, et al. Safety and efficacy of a lifestyle intervention for pregnant women to prevent excessive maternal weight gain: a cluster-randomized controlled trial. BMC Pregnancy Childbirth. 2013;13:151.<br>35. Koivusalo SB, Rono K, Klemetti MM, Roine RP, Lindstrom J, Erkkola M, et al. Gestational Diabetes Mellitus Can Be Prevented by Lifestyle Intervention: The Finnish Gestational Diabetes Prevention Study (RADIEL): A Randomized Controlled Trial. Diabetes Care. 2016;39(1):24-30.<br>36. Poston L, Bell R, Croker H, Flynn AC, Godfrey KM, Goff L, et al. Effect of a behavioural intervention in obese pregnant women (the UPBEAT study): a multicentre, randomised controlled trial. Lancet Diabetes Endocrinol. 2015;3(10):767-77.<br>37. Jing W, Huang Y, Liu X, Luo B, Yang Y, Liao S. The effect of a personalized intervention on weight gain and physical activity among pregnant women in China. Int J Gynaecol Obstet. 2015;129(2):138-41.<br>38. Petrella E, Malavolti M, Bertarini V, Pignatti L, Neri I, Battistini NC, et al. Gestational weight gain in overweight and obese women enrolled in a healthy lifestyle and eating habits program. J Matern Fetal Neonatal Med. 2014;27(13):1348-52.<br>39. Harrison CL, Lombard CB, Strauss BJ, Teede HJ. Optimizing healthy gestational weight gain in women at high risk of gestational diabetes: a randomized controlled trial. Obesity (Silver Spring). 2013;21(5):904-9.<br>40. Polley BA, Wing RR, Sims CJ. Randomized controlled trial to prevent excessive weight gain in pregnant women. Int J Obes Relat Metab Disord. 2002;26(11):1494-502.<br>41. Phelan S, Phipps MG, Abrams B, Darroch F, Schaffner A, Wing RR. Randomized trial of a behavioral intervention to prevent excessive gestational weight gain: the Fit for Delivery Study. Am J Clin Nutr. 2011;93(4):772-9.<br>42. Dodd JM, Newman A, Moran LJ, Deussen AR, Grivell RM, Yelland LN, et al. The effect of antenatal dietary and lifestyle advice for women who are overweight or obese on emotional well-being: the LIMIT randomized trial. Acta Obstet Gynecol Scand. 2016;95(3):309-18.<br>43. Sagedal LR, Overby NC, Bere E, Torstveit MK, Lohne-Seiler H, Smastuen M, et al. Lifestyle intervention to limit gestational weight gain: the Norwegian Fit for Delivery randomised controlled trial. BJOG. 2017;124(1):97-109.<br>44. Smith K, Lanningham-Foster L, Welch A, Campbell C. Web-Based Behavioral Intervention Increases Maternal Exercise but Does Not Prevent Excessive Gestational Weight Gain in Previously Sedentary Women. J Phys Act Health. 2016;13(6):587-93.<br>45. Vesco KK, Karanja N, King JC, Gillman MW, Leo MC, Perrin N, et al. Efficacy of a group-based dietary intervention for limiting gestational weight gain among obese women: a randomized trial. Obesity (Silver Spring). 2014;22(9):1989-96.<br>46. Asbee SM, Jenkins TR, Butler JR, White J, Elliot M, Rutledge A. Preventing excessive weight gain during pregnancy through dietary and lifestyle counseling: a randomized controlled trial. Obstet Gynecol. 2009;113(2 Pt 1):305-12.<br>47. Barakat R, Pelaez M, Montejo R, Luaces M, Zakynthinaki M. Exercise during pregnancy improves maternal health perception: a randomized controlled trial. Am J Obstet Gynecol. 2011;204(5):402 e1-7.<br>48. Hopkins SA, Baldi JC, Cutfield WS, McCowan L, Hofman PL. Exercise training in pregnancy reduces offspring size without changes in maternal insulin sensitivity. J Clin Endocrinol Metab. 2010;95(5):2080-8.<br>49. Murtezani A, Pacarada M, Ibraimi Z, Nevzati A, Abazi N. The impact of exercise during pregnancy on neonatal outcomes: a randomized controlled trial. J Sports Med Phys Fitness. 2014;54(6):802-8.<br>50. Perales M, Mateos S, Vargas M, Sanz I, Lucia A, Barakat R. Fetal and maternal heart rate responses to exercise in pregnant women. A randomized Controlled Trial. Archivos de medicina del deporte 2015;32(6):361-7.<br>51. Haakstad LA, Bo K. Effect of regular exercise on prevention of excessive weight gain in pregnancy: a randomised controlled trial. Eur J Contracept Reprod Health Care. 2011;16(2):116-25.<br>52. Pelaez M, Gonzalez-Cerron S, Montejo R, Barakat R. Pelvic floor muscle training included in a pregnancy exercise program is effective in primary prevention of urinary incontinence: a randomized controlled trial. Neurourol Urodyn. 2014;33(1):67-71.<br>53. Yeo S, Davidge S, Ronis DL, Antonakos CL, Hayashi R, O'Leary S. A comparison of walking versus stretching exercises to reduce the incidence of preeclampsia: a randomized clinical trial. Hypertens Pregnancy. 2008;27(2):113-30.<br>54. McAuley SE, Jensen D, McGrath MJ, Wolfe LA. Effects of human pregnancy and aerobic conditioning on alveolar gas exchange during exercise. Can J Physiol Pharmacol. 2005;83(7):625-33.<br>55. Hollingsworth DR, Moore TR. Postprandial walking exercise in pregnant insulin-dependent (type I) diabetic women: reduction of plasma lipid levels but absence of a significant effect on glycemic control. Am J Obstet Gynecol. 1987;157(6):1359-63.<br>56. Avery MD, Leon AS, Kopher RA. Effects of a partially home-based exercise program for women with gestational diabetes. Obstet Gynecol. 1997;89(1):10-5.<br>57. de Oliveria Melo AS, Silva JL, Tavares JS, Barros VO, Leite DF, Amorim MM. Effect of a physical exercise program during pregnancy on uteroplacental and fetal blood flow and fetal growth: a randomized controlled trial. Obstet Gynecol. 2012;120(2 Pt 1):302-10.<br>58. Kasawara KT, Burgos CS, do Nascimento SL, Ferreira NO, Surita FG, Pinto ESJL. Maternal and Perinatal Outcomes of Exercise in Pregnant Women with Chronic Hypertension and/or Previous Preeclampsia: A Randomized Controlled Trial. ISRN Obstet Gynecol. 2013;2013:857047.<br>59. Kordi R, Abolhasani M, Rostami M, Hantoushzadeh S, Mansournia MA, Vasheghani-Farahani F. Comparison between the effect of lumbopelvic belt and home based pelvic stabilizing exercise on pregnant women with pelvic girdle pain; a randomized controlled trial. J Back Musculoskelet Rehabil. 2013;26(2):133-9.<br>60. Taniguchi C, Sato C. Home-based walking during pregnancy affects mood and birth outcomes among sedentary women: A randomized controlled trial. Int J Nurs Pract. 2016;22(5):420-6.<br>61. Korpi-Hyovalti E, Heinonen S, Schwab U, Laaksonen DE, Niskanen L. Effect of intensive counselling on physical activity in pregnant women at high risk for gestational diabetes mellitus. A clinical study in primary care. Prim Care Diabetes. 2012;6(4):261-8.

Review	References of included behavioural interventions delivered in pregnancy
	62. Dyck RF, Sheppard SM, Klomp H, Tan L, Chad K, van Vliet SH. Using exercise to prevent gestational diabetes among aboriginal women -- hypothesis and results of a pilot/feasibility project in Saskatchewan. Canadian Journal of Diabetes Care. 1999;23(3):32-8.<br>63. Sun Y, Zhao H. The effectiveness of lifestyle intervention in early pregnancy to prevent gestational diabetes mellitus in Chinese overweight and obese women: A quasi-experimental study. Appl Nurs Res. 2016;30:125-30.<br>64. Liu J, Wilcox S, Whitaker K, Blake C, Addy C. Preventing excessive weight gain during pregnancy and promoting postpartum weight loss: a pilot lifestyle intervention for overweight and obese African American women. Matern Child Health J. 2015;19(4):840-9.<br>65. Mustila T, Raitanen J, Keskinen P, Saari A, Luoto R. Pragmatic controlled trial to prevent childhood obesity in maternity and child health care clinics: pregnancy and infant weight outcomes (the VACOPP Study). BMC Pediatr. 2013;13:80.<br>66. Shirazian T, Monteith S, Friedman F, Rebarber A. Lifestyle modification program decreases pregnancy weight gain in obese women. Am J Perinatol. 2010;27(5):411-4.<br>67. McGiveron A, Foster S, Pearce J, Taylor MA, McMullen S, Langley-Evans SC. Limiting antenatal weight gain improves maternal health outcomes in severely obese pregnant women: findings of a pragmatic evaluation of a midwife-led intervention. J Hum Nutr Diet. 2015;28 Suppl 1:29-37.<br>68. Narendran S, Nagarathna R, Narendran V, Gunasheela S, Nagendra HR. Efficacy of yoga on pregnancy outcome. J Altern Complement Med. 2005;11(2):237-44.<br>69. O'Connor PJ, Poudevigne MS, Cress ME, Motl RW, Clapp JF, 3rd. Safety and efficacy of supervised strength training adopted in pregnancy. J Phys Act Health. 2011;8(3):309-20.<br>70. Lombardi W, Wilson S, Peniston PB. Wellness intervention with pregnant soldiers. Mil Med. 1999;164(1):22-9.<br>71. Bell RJ, Palma SM, Lumley JM. The effect of vigorous exercise during pregnancy on birth-weight. Aust N Z J Obstet Gynaecol. 1995;35(1):46-51.<br>72. Hatch MC, Shu XO, McLean DE, Levin B, Begg M, Reuss L, et al. Maternal exercise during pregnancy, physical fitness, and fetal growth. Am J Epidemiol. 1993;137(10):1105-14.<br>73. Magann EF, Evans SF, Weitz B, Newnham J. Antepartum, intrapartum, and neonatal significance of exercise on healthy low-risk pregnant working women. Obstet Gynecol. 2002;99(3):466-72.<br>74. Piravej K, Saksirinukul R. Survey of patterns, attitudes, and the general effects of exercise during pregnancy in 203 Thai pregnant women at King Chulalongkorn Memorial Hospital. J Med Assoc Thai. 2001;84 Suppl 1:S276-82.<br>75. To WW, Wong MW. Bone mineral density changes during pregnancy in actively exercising women as measured by quantitative ultrasound. Arch Gynecol Obstet. 2012;286(2):357-63.<br>76. Putnam KF, Mueller LA, Magann EF, Thagard A, Johnson AM, Ounpraseuth ST, et al. Evaluating effects of self-reported domestic physical activity on pregnancy and neonatal outcomes in "stay at home" military wives. Mil Med. 2013;178(8):893-8.<br>77. Badon SE, Wander PL, Qiu C, Miller RS, Williams MA, Enquobahrie DA. Maternal Leisure Time Physical Activity and Infant Birth Size. Epidemiology. 2016;27(1):74-81.<br>78. Vollebregt KC, Wolf H, Boer K, van der Wal MF, Vrijkotte TG, Bonsel GJ. Does physical activity in leisure time early in pregnancy reduce the incidence of preeclampsia or gestational hypertension? Acta Obstet Gynecol Scand. 2010;89(2):261-7.<br>79. Harris ST, Liu J, Wilcox S, Moran R, Gallagher A. Exercise during pregnancy and its association with gestational weight gain. Matern Child Health J. 2015;19(3):528-37.<br>80. Chasan-Taber L, Schmidt MD, Pekow P, Sternfeld B, Manson JE, Solomon CG, et al. Physical activity and gestational diabetes mellitus among Hispanic women. J Womens Health (Larchmt). 2008;17(6):999-1008.<br>81. Chasan-Taber L, Silveira M, Pekow P, Braun B, Manson JE, Solomon CG, et al. Physical activity, sedentary behavior and risk of hypertensive disorders of pregnancy in Hispanic women. Hypertens Pregnancy. 2015;34(1):1-16.<br>82. Currie LM, Woolcott CG, Fell DB, Armson BA, Dodds L. The association between physical activity and maternal and neonatal outcomes: a prospective cohort. Matern Child Health J. 2014;18(8):1823-30.<br>83. Iqbal R, Rafique G, Badruddin S, Qureshi R, Cue R, Gray-Donald K. Increased body fat percentage and physical inactivity are independent predictors of gestational diabetes mellitus in South Asian women. Eur J Clin Nutr. 2007;61(6):736-42.<br>84. Morkrid K, Jenum AK, Berntsen S, Sletner L, Richardsen KR, Vangen S, et al. Objectively recorded physical activity and the association with gestational diabetes. Scand J Med Sci Sports. 2014;24(5):e389-97.<br>85. Leng J, Liu G, Zhang C, Xin S, Chen F, Li B, et al. Physical activity, sedentary behaviors and risk of gestational diabetes mellitus: a population-based cross-sectional study in Tianjin, China. Eur J Endocrinol. 2016;174(6):763-73.<br>86. Li Q, Xiong R, Wang L, Cui J, Shi L, Liu Y, et al. Associations of dietary habits, physical activity and cognitive views with gestational diabetes mellitus among Chinese women. Public Health Nutrition. 2014;17(8):1850-7.<br>87. Momeni Javid F, Simbar M, Dolatian M, Alavi Majd H. Comparison of lifestyles of women with gestational diabetes and healthy pregnant women. Glob J Health Sci. 2015;7(2):162-9.<br>88. Oken E, Ning Y, Rifas-Shiman SL, Radesky JS, Rich-Edwards JW, Gillman MW. Associations of physical activity and inactivity before and during pregnancy with glucose tolerance. Obstet Gynecol. 2006;108(5):1200-7.<br>89. Lindqvist M, Lindkvist M, Eurenius E, Persson M, Ivarsson A, Mogren I. Leisure time physical activity among pregnant women and its associations with maternal characteristics and pregnancy outcomes. Sex Reprod Healthc. 2016;9:14-20.<br>90. Dale E, Mullinax KM, Bryan DH. Exercise during pregnancy: effects on the fetus. Can J Appl Sport Sci. 1982;7(2):98-103.<br>91. White E, Pivarnik J, Pfeiffer K. Resistance training during pregnancy and perinatal outcomes. J Phys Act Health. 2014;11(6):1141-8.<br>92. Dempsey JC, Butler CL, Sorensen TK, Lee IM, Thompson ML, Miller RS, et al. A case-control study of maternal recreational physical activity and risk of gestational diabetes mellitus. Diabetes Res Clin Pract. 2004;66(2):203-15.<br>93. Harizopoulou VC, Kritikos A, Papanikolaou Z, Saranti E, Vavilis D, Klonos E, et al. Maternal physical activity before and during early pregnancy as a risk factor for gestational diabetes mellitus. Acta Diabetol. 2010;47 Suppl 1:83-9.

Review	References of included behavioural interventions delivered in pregnancy
	94. Nasiri-Amiri F, Bakhtiari A, Faramarzi M, Adib Rad H, Pasha H. The Association Between Physical Activity During Pregnancy and Gestational Diabetes Mellitus: A Case-Control Study. Int J Endocrinol Metab. 2016;14(3):e37123.<br>95. Juhl M, Kogevinas M, Andersen PK, Andersen AM, Olsen J. Is swimming during pregnancy a safe exercise? Epidemiology. 2010;21(2):253-8.<br>96. Weissgerber TL, Davies GA, Roberts JM. Modification of angiogenic factors by regular and acute exercise during pregnancy. J Appl Physiol (1985). 2010;108(5):1217-23.<br>97. Ferland S, Bujold E, Giguere Y, Girard M, Demers S, Forest JC. Association between physical activity in early pregnancy and markers of placental growth and function. J Obstet Gynaecol Can. 2013;35(9):787-92.<br>98. Saftlas AF, Logsden-Sackett N, Wang W, Woolson R, Bracken MB. Work, leisure-time physical activity, and risk of preeclampsia and gestational hypertension. Am J Epidemiol. 2004;160(8):758-65.<br>99. Martin CL, Brunner Huber LR. Physical activity and hypertensive complications during pregnancy: findings from 2004 to 2006 North Carolina Pregnancy Risk Assessment Monitoring System. Birth. 2010;37(3):202-10.<br>100. Marcoux S, Brisson J, Fabia J. The effect of leisure time physical activity on the risk of pre-eclampsia and gestational hypertension. J Epidemiol Community Health. 1989;43(2):147-52.<br>101. Snapp CA, Donaldson SK. Gestational diabetes mellitus: physical exercise and health outcomes. Biol Res Nurs. 2008;10(2):145-55.<br>102. Haelterman E, Marcoux S, Croteau A, Dramaix M. Population-based study on occupational risk factors for preeclampsia and gestational hypertension. Scand J Work Environ Health. 2007;33(4):304-17.<br>103. Spracklen CN, Ryckman KK, Triche EW, Saftlas AF. Physical Activity During Pregnancy and Subsequent Risk of Preeclampsia and Gestational Hypertension: A Case Control Study. Matern Child Health J. 2016;20(6):1193-202.<br>104. Downs DS, Hausenblass HA. Pregnant women's third trimester exercise behaviors, body mass index, and pregnancy outcomes. Psychology and Health. 2007;22(5):545-59.<br>105. Magnus P, Trogstad L, Owe KM, Olsen SF, Nystad W. Recreational physical activity and the risk of preeclampsia: a prospective cohort of Norwegian women. Am J Epidemiol. 2008;168(8):952-7.<br>106. Sorensen TK, Williams MA, Lee IM, Dashow EE, Thompson ML, Luthy DA. Recreational physical activity during pregnancy and risk of preeclampsia. Hypertension. 2003;41(6):1273-80.
Davenport *et al.* 2019b [48]	1. Avery MD, Leon AS, Kopher RA. Effects of a partially home-based exercise program for women with gestational diabetes. Obstet Gynecol. 1997;89(1):10-5.<br>2. Guelfi KJ, Ong MJ, Crisp NA, Fournier PA, Wallman KE, Grove JR, et al. Regular Exercise to Prevent the Recurrence of Gestational Diabetes Mellitus: A Randomized Controlled Trial. Obstetrics & Gynecology. 2016;128(4):819-27.<br>3. Kulpa PJ, White BM, Visscher R. Aerobic exercise in pregnancy. Am J Obstet Gynecol. 1987;156(6):1395-403.<br>4. Hollingsworth DR, Moore TR. Postprandial walking exercise in pregnant insulin-dependent (type I) diabetic women: reduction of plasma lipid levels but absence of a significant effect on glycemic control. Am J Obstet Gynecol. 1987;157(6):1359-63.<br>5. Beyaz EA, Ozcan E, Ketenci A, Beyaz MM. The effectiveness of pregnancy rehabilitation: Effects on low back pain and calf cramps during pregnancy and pregnancy outcome. Nobel Medicus. 2011;7(2):67-74.<br>6. Lombardi W, Wilson S, Peniston PB. Wellness intervention with pregnant soldiers. Mil Med. 1999;164(1):22-9.<br>7. Clapp JF, 3rd. The course of labor after endurance exercise during pregnancy. Am J Obstet Gynecol. 1990;163(6 Pt 1):1799-805.<br>8. Magann EF, Evans SF, Weitz B, Newnham J. Antepartum, intrapartum, and neonatal significance of exercise on healthy low-risk pregnant working women. Obstet Gynecol. 2002;99(3):466-72.<br>9. Piravej K, Saksirinukul R. Survey of patterns, attitudes, and the general effects of exercise during pregnancy in 203 Thai pregnant women at King Chulalongkorn Memorial Hospital. Journal of the Medical Association of Thailand. 2001;84(Suppl 1):S276-82.<br>10. Downs DS, Hausenblas HA. Pregnant women's third trimester exercise behaviors, body mass index, and pregnancy outcomes. PsycholHealth. 2007;22(5):545-59.<br>11. Baciuk EP, Pereira RI, Cecatti JG, Braga AF, Cavalcante SR. Water aerobics in pregnancy: Cardiovascular response, labor and neonatal outcomes. Reprod Health. 2008;5:10.<br>12. Barakat R, Ruiz JR, Stirling JR, Zakynthinaki M, Lucia A. Type of delivery is not affected by light resistance and toning exercise training during pregnancy: a randomized controlled trial. Am J Obstet Gynecol. 2009;201(6):590 e1-6.<br>13. Elden H, Ostgaard HC, Fagevik-Olsen M, Ladfors L, Hagberg H. Treatments of pelvic girdle pain in pregnant women: adverse effects of standard treatment, acupuncture and stabilising exercises on the pregnancy, mother, delivery and the fetus/neonate. BMC Complement Altern Med. 2008;8:34.<br>14. Barakat R, Cordero Y, Coteron J, Luaces M, Montejo R. Exercise during pregnancy improves maternal glucose screen at 24-28 weeks: a randomised controlled trial. British Journal of Sports Medicine. 2012;46(9):656-61.<br>15. Barakat R, Pelaez M, Montejo R, Luaces M, Zakynthinaki M. Exercise during pregnancy improves maternal health perception: a randomized controlled trial. Am J Obstet Gynecol. 2011;204(5):402 e17.<br>16. Dias LA, Driusso P, Aita DL, Quintana SM, Bo K, Ferreira CH. Effect of pelvic floor muscle training on labour and newborn outcomes: a randomized controlled trial. Rev Bras Fisioter. 2011;15(6):487-93.<br>17. Nascimento SL, Surita FG, Parpinelli MA, Siani S, Pinto e Silva JL. The effect of an antenatal physical exercise programme on maternal/perinatal outcomes and quality of life in overweight and obese pregnant women: a randomised clinical trial. BJOG. 2011;118(12):1455-63.<br>18. Pinzon DC, Zamora K, Martinez JH, et al. Type of delivery and gestational age is not affected by pregnant Latin-American women engaging in vigorous exercise: a secondary analysis of data from a controlled randomized trial. Revista de Salud Publica. 2012;14(5):731-43.<br>19. Price BB, Amini SB, Kappeler K. Exercise in pregnancy: effect on fitness and obstetric outcomes a randomized trial. Med Sci Sports Exerc. 2012;44(12):2263-9.<br>20. Cordero Rodriguez Y, Peláez Puente M, Miguel Abad Md, Perales Santaella M, Barakat Carballo R. Puede el ejercicio físico moderado durante el embarazo actuar como un factor de prevención de la Diabetes Gestacional? / Can moderate physical exercise during pregnancy act as a factor in preventing Gestational Diabetes? RICYDE Rev int cienc deporte. 2012;27(8):3-19.<br>21. Barakat R, Pelaez M, Lopez C, Lucia A, Ruiz JR. Exercise during pregnancy and gestational diabetes-related adverse effects: a randomised controlled trial. Br J Sports Med. 2013;47(10):630-6.<br>22. Barakat R, Perales M, Bacchi M, Coteron J, Refoyo I. A program of exercise throughout pregnancy. Is it safe to mother and newborn? American Journal of Health Promotion. 2014;29(1):2-8.

Review	References of included behavioural interventions delivered in pregnancy
	23. Bisson M, Almeras N, Dufresne SS, Robitaille J, Rheaume C, Bujold E, et al. A 12-Week Exercise Program for Pregnant Women with Obesity to Improve Physical Activity Levels: An Open Randomised Preliminary Study. PLoS One. 2015;10(9):e0137742.<br>24. Cordero Y, Mottola MF, Vargas J, Blanco M, Barakat R. Exercise Is Associated with a Reduction in Gestational Diabetes Mellitus. Medicine and Science in Sports and Exercise. 2015;47(7):1328-33.<br>25. Ghodsi Z, Asltoghiri M. Effects of aerobic exercise training on maternal and neonatal outcome: a randomized controlled trial on pregnant women in Iran. J Pak Med Assoc. 2014;64(9):1053-6.<br>26. Halse RE, Wallman KE, Dimmock JA, Newnham JP, Guelfi KJ. Home-Based Exercise Improves Fitness and Exercise Attitude and Intention in Women with GDM. Med Sci Sports Exerc. 2015;47(8):1698-704.<br>27. Kasawara KT, Burgos CS, do Nascimento SL, Ferreira NO, Surita FG, Pinto ESJL. Maternal and Perinatal Outcomes of Exercise in Pregnant Women with Chronic Hypertension and/or Previous Preeclampsia: A Randomized Controlled Trial. ISRN Obstet Gynecol. 2013;2013:857047.<br>28. Kong KAI, Campbell C, Foster R, Peterson A, Lanningham-Foster L. A Pilot Walking Program Promotes Moderate-Intensity Physical Activity during Pregnancy. Medicine & Science in Sports & Exercise. 2014;46(3):462-71.<br>29. Nobles C, Marcus BH, Stanek rEJ, Braun B, Whitcomb BW, Solomon CG, et al. Effect of an exercise intervention on gestational diabetes mellitus: a randomized controlled trial. Obstetrics and gynecology. 2015;125(5):1195.<br>30. Okido M, Valeri F, Martins W, Ferreira C, Duarte G, Cavalli R. Assessment of foetal wellbeing in pregnant women subjected to pelvic floor muscle training: a controlled randomised study. International Urogynecology Journal. 2015;26(10):1475-81.<br>31. Orawan L, Preecha W. Effect of pelvic floor muscle training (PFMT) during pregnancy on bladder neck descend and delivery. Journal of the Medical Association of Thailand. 2014;97(Suppl 8):S156-63.<br>32. Petrov Fieril K, Glantz A, Fagevik Olsen M. The efficacy of moderate-to-vigorous resistance exercise during pregnancy: a randomized controlled trial. Acta Obstetricia et Gynecologica Scandinavica. 2015;94(1):35-42.<br>33. Ruiz JR, Perales M, Pelaez M, Lopez C, Lucia A, Barakat R. Supervised exercise-based intervention to prevent excessive gestational weight gain: a randomized controlled trial. Mayo Clin Proc. 2013;88(12):1388-97.<br>34. Renault KM, Norgaard K, Nilas L, Carlsen EM, Cortes D, Pryds O, et al. The Treatment of Obese Pregnant Women (TOP) study: a randomized controlled trial of the effect of physical activity intervention assessed by pedometer with or without dietary intervention in obese pregnant women. Am J Obstet Gynecol. 2014;210(2):134 e1-9.<br>35. Tomic V, Sporis G, Tomic J, Milanovic Z, Zigmundovac-Klaic D, Pantelic S. The effect of maternal exercise during pregnancy on abnormal fetal growth. Croat Med J. 2013;54(4):362-8.<br>36. Wang X, Li GY, Deng ML. Pelvic floor muscle training as a persistent nursing intervention: Effect on delivery outcome and pelvic floor myodynamia. International Journal of Nursing Sciences. 2014;1(1):48-52.<br>37. Collings CA, Curet LB, Mullin JP. Maternal and fetal responses to a maternal aerobic exercise program. Am J Obstet Gynecol. 1983;145(6):702-7.<br>38. Oostdam N, van Poppel MNM, Wouters MGAJ, Eekhoff EMW, Bekedam DJ, Kuchenbecker WKH, et al. No effect of the FitFor2 exercise programme on blood glucose, insulin sensitivity, and birthweight in pregnant women who were overweight and at risk for gestational diabetes: results of a randomised controlled trial. Bjog-an International Journal of Obstetrics and Gynaecology. 2012;119(9):1098-107.<br>39. Perales M, Mateos S, Vargas M, Sanz I, Lucia A, Barakat R. Fetal and maternal heart rate responses to exercise in pregnant women. A randomized controlled trial. Archivos de Medicina del Deporte. 2015;32(6):361-7.<br>40. Seneviratne SN, Jiang Y, Derraik J, McCowan L, Parry GK, Biggs JB, et al. Effects of antenatal exercise in overweight and obese pregnant women on maternal and perinatal outcomes: a randomised controlled trial. BJOG. 2016;123(4):588-97.<br>41. Chávez VG, Sánchez, M. D. P V, Rasch JRK. Effect of pelvic floor exercise during pregnancy and puerperium on prevention of urinary stress incontinence. Ginecol Obstet Mex. 2004;72(628-636).<br>42. Morkved S, Bo K, Schei B, Salvesen KA. Pelvic floor muscle training during pregnancy to prevent urinary incontinence: a single-blind randomized controlled trial. Obstet Gynecol. 2003;101(2):313-9.<br>43. Marquez-Sterling S, Perry AC, Kaplan TA, Halberstein RA, Signorile JF. Physical and psychological changes with vigorous exercise in sedentary primigravidae. Med Sci Sports Exerc. 2000;32(1):58-62.<br>44. Barakat R, Alonso G, Rojo JJ. Physical exercise during pregnancy and its relationship with the duration of stages of labor. Prog Obstet Ginecol. 2005;48(2):61-8.<br>45. Fritel X, de Tayrac R, Bader G, Savary D, Gueye A, Deffieux X, et al. Preventing Urinary Incontinence With Supervised Prenatal Pelvic Floor Exercises: A Randomized Controlled Trial. Obstet Gynecol. 2015;126(2):370-7.<br>46. Callaway LK, Colditz PB, Byrne NM, Lingwood BE, Rowlands IJ, Foxcroft K, et al. Prevention of gestational diabetes: feasibility issues for an exercise intervention in obese pregnant women. Diabetes Care. 2010;33(7):1457-9.<br>47. Reilly ET, Freeman RM, Waterfield MR, Waterfield AE, Steggles P, Pedlar F. Prevention of postpartum stress incontinence in primigravidae with increased bladder neck mobility: a randomised controlled trial of antenatal pelvic floor exercises. Bjog. 2002;109(1):68-76.<br>48. Ko PC, Liang CC, Chang SD, Lee JT, Chao AS, Cheng PJ. A randomized controlled trial of antenatal pelvic floor exercises to prevent and treat urinary incontinence. Int Urogynecol J. 2011;22(1):17-22.<br>49. Kihlstrand M, Stenman B, Nilsson S, Axelsson O. Water-gymnastics reduced the intensity of back/low back pain in pregnant women. Acta Obstet Gynecol Scand. 1999;78(3):180-5.<br>50. Ussher M, Lewis S, Aveyard P, et al. Physical activity for smoking cessation in pregnancy: randomised controlled trial. British Medical Journal. 2015;350:h2145.<br>51. Nielsen CA, Sigsgaard I, Olsen M, Tolstrup M, Danneskiold-Samsoee B, Bock JE. Trainability of the pelvic floor. A prospective study during pregnancy and after delivery. Acta Obstet Gynecol Scand. 1988;67(5):437-40.<br>52. Barakat R, Pelaez M, Cordero Y, Perales M, Lopez C, Coteron J, et al. Exercise during pregnancy protects against hypertension and macrosomia: randomized clinical trial. Am J Obstet Gynecol. 2016;214(5):649 e1-8.<br>53. Taniguchi C, Sato C. Home-based walking during pregnancy affects mood and birth outcomes among sedentary women: A randomized controlled trial. International Journal of Nursing Practice. 2016;22(5):420-6.<br>54. Carpenter RE, Emery SJ, Uzun O, Rassi D, Lewis MJ. Influence of antenatal physical exercise on heart rate variability and QT variability. Journal of Maternal-Fetal & Neonatal Medicine. 2017;30(1):79.<br>55. de Barros MC, Lopes MA, Francisco RP, Sapienza AD, Zugaib M. Resistance exercise and glycemic control in women with gestational diabetes mellitus. Am J Obstet Gynecol. 2010;203(6):556 e1-6.<br>56. Guelinckx I, Devlieger R, Mullie P, Vansant G. Effect of lifestyle intervention on dietary habits, physical activity, and gestational weight gain in obese pregnant women: a randomized controlled trial. Am J Clin Nutr. 2010;91(2):373-80.<br>57. Hui A, Back L, Ludwig S, Gardiner P, Sevenhuysen G, Dean H, et al. Lifestyle intervention on diet and exercise reduced excessive gestational weight gain in pregnant women under a randomised controlled trial. BJOG. 2012;119(1):70-7.

Review	References of included behavioural interventions delivered in pregnancy
	58. Stafne SN, Salvesen KA, Romundstad PR, Eggebo TM, Carlsen SM, Morkved S. Regular exercise during pregnancy to prevent gestational diabetes: a randomized controlled trial. Obstet Gynecol. 2012;119(1):29-36.<br>59. Vinter CA, Jensen DM, Ovesen P, Beck-Nielsen H, Jorgensen JS. The LiP (Lifestyle in Pregnancy) study: a randomized controlled trial of lifestyle intervention in 360 obese pregnant women. Diabetes Care. 2011;34(12):2502-7.<br>60. Bo S, Rosato R, Ciccone G, Canil S, Gambino R, Poala CB, et al. Simple lifestyle recommendations and the outcomes of gestational diabetes. A 2 x 2 factorial randomized trial. Diabetes Obes Metab. 2014;16(10):1032-5.<br>61. Hui AL, Back L, Ludwig S, Gardiner P, Sevenhuysen G, Dean HJ, et al. Effects of lifestyle intervention on dietary intake, physical activity level, and gestational weight gain in pregnant women with different pre-pregnancy Body Mass Index in a randomized control trial. BMC Pregnancy Childbirth. 2014;14:331.<br>62. Miquelutti MA, Cecatti JG, Makuch MY. Evaluation of a birth preparation program on lumbopelvic pain, urinary incontinence, anxiety and exercise: a randomized controlled trial. BMC Pregnancy Childbirth. 2013;13:154.<br>63. Rauh K, Gabriel E, Kerschbaum E, Schuster T, von Kries R, Amann-Gassner U, et al. Safety and efficacy of a lifestyle intervention for pregnant women to prevent excessive maternal weight gain: a cluster-randomized controlled trial. BMC Pregnancy Childbirth. 2013;13:151.<br>64. Koivusalo SB, Rono K, Klemetti MM, et al. Gestational Diabetes Mellitus Can Be Prevented by Lifestyle Intervention: The Finnish Gestational Diabetes Prevention Study (RADIEL): A Randomized Controlled Trial. Diabetes Care. 2016;39(1):24-30.<br>65. Poston L, Bell R, Croker H, Flynn AC, Godfrey KM, Goff L, et al. Effect of a behavioural intervention in obese pregnant women (the UPBEAT study): a multicentre, randomised controlled trial. Lancet Diabetes Endocrinol. 2015;3(10):767-77.<br>66. Althuizen E vdWC, van Mechelen W, Seidell JC, van Poppel MNM. The effect of a counselling intervention on weight changes during and after pregnancy: a randomised trial. British Journal of Obstetrics and Gynecology. 2013;120:92-9.<br>67. Petrella E, Malavolti M, Bertarini V, Pignatti L, Neri I, Battistini NC, et al. Gestational weight gain in overweight and obese women enrolled in a healthy lifestyle and eating habits program. J Matern Fetal Neonatal Med. 2014;27(13):1348-52.<br>68. Polley BA, Wing RR, Sims CJ. Randomized controlled trial to prevent excessive weight gain in pregnant women. Int J Obes Relat Metab Disord. 2002;26(11):1494-502.<br>69. Phelan S, Phipps MG, Abrams B, Darroch F, Schaffner A, Wing RR. Randomized trial of a behavioral intervention to prevent excessive gestational weight gain: the Fit for Delivery Study. Am J Clin Nutr. 2011;93(4):772-9. 7<br>70. Sagedal LR, Overby NC, Bere E, Torstveit MK, Lohne-Seiler H, Smastuen M, et al. Lifestyle intervention to limit gestational weight gain: the Norwegian Fit for Delivery randomised controlled trial. BJOG. 2017;124(1):97-109.<br>71. Asbee, S M, Jenkins, T R, Butler, J R, et al. Preventing excessive weight gain during pregnancy through dietary and lifestyle counseling: a randomized controlled trial. Obstetrics and gynecology. 2009;113(2 Pt 1):305.<br>72. Dodd J, Grivell R, Owens J. Antenatal Dietary and Lifestyle Interventions for Women Who are Overweight or Obese: Outcomes from the LIMIT Randomized Trial. Current Nutrition Reports. 2014;3(4):392-9.<br>73. Vesco KK, Karanja N, King JC, Gillman MW, Leo MC, Perrin N, et al. Efficacy of a group-based dietary intervention for limiting gestational weight gain among obese women: a randomized trial. Obesity (Silver Spring, Md). 2014;22(9):1989-96.<br>74. Kariminia A, Chamberlain ME, Keogh J, Shea A. Randomised controlled trial of effect of hands and knees posturing on incidence of occiput posterior position at birth. BMJ. 2004;328(7438):490.<br>75. Beckmann CR, Beckmann CA. Effect of a structured antepartum exercise program on pregnancy and labor outcome in primiparas. J Reprod Med. 1990;35(7):704-9.<br>76. Melzer K, Schutz Y, Soehnchen N, et al. Effects of recommended levels of physical activity on pregnancy outcomes. American Journal of Obstetrics & Gynecology. 2010;202(3):266.e1-6.<br>77. Lindqvist M, Lindkvist M, Eurenius E, Persson M, Ivarsson A, Mogren I. Leisure time physical activity among pregnant women and its associations with maternal characteristics and pregnancy outcomes. Sexual and Reproductive Healthcare. 2016;9:14-20.<br>78. Perales M, Cordero Y, Vargas M, Lucia A, Barakat R. Exercise and depression in overweight and obese pregnant women: A randomised controlled trial. Archivos de Medicina del Deporte. 2015;32:707.<br>79. Chuntharapat S, Petpichetchian W, Hatthakit U. Yoga during pregnancy: effects on maternal comfort, labor pain and birth outcomes. Complement Ther Clin Pract. 2008;14(2):105-15.<br>80. Perales M, Santos-Lozano A, Sanchis-Gomar F, Luaces M, Pareja-Galeano H, Garatachea N, et al. Maternal Cardiac Adaptations to a Physical Exercise Program during Pregnancy. Med Sci Sports Exerc. 2016;48(5):896-906.<br>81. Woldringh C, Wijngaart Mvd, Albers-Heitner P, Lycklama a N, A A, Lagro-Janssen T. Pelvic floor muscle training is not effective in women with UI in pregnancy: a randomised controlled trial. Int Urogynecol J Pelvic Floor Dysfunct. 2007;18(4):383-90.<br>82. Bo K, Hilde G, Staer-Jensen J, Siafarikas F, Tennfjord MK, Engh ME. Does general exercise training before and during pregnancy influence the pelvic floor "opening" and delivery outcome? A 3D/4D ultrasound study following nulliparous pregnant women from mid-pregnancy to childbirth. Br J Sports Med. 2015;49:196-9.<br>83. Carpenter RE, Emery SJ, Uzun O, Silva LAD, Lewis MJ. Influence of antenatal physical exercise on haemodynamics in pregnant women: A flexible randomisation approach. BMC Pregnancy and Childbirth. 2015;15:156.<br>84. Botkin C, Driscoll CE. Maternal aerobic exercise: newborn effects. Fam Pract Res J. 1991;11(4):387-93.<br>85. Salvesen KA, Stafne SN, Eggebo TM, Morkved S. Does regular exercise in pregnancy influence duration of labor? A secondary analysis of a randomized controlled trial. Acta Obstet Gynecol Scand. 2014;93(1):73-9.<br>86. Brenner IK, Wolfe LA, Monga M, McGrath MJ. Physical conditioning effects on fetal heart rate responses to graded maternal exercise. Medicine & Science in Sports & Exercise. 1999;31(6):792-9.<br>87. Horns PN, Ratcliffe LP, Leggett JC, Swanson MS. Pregnancy outcomes among active and sedentary primiparous women. Journal of Obstetric, Gynecologic, & Neonatal Nursing. 1996;25(1):4954.<br>88. Rice PL, Fort IL. The relationship of maternal exercise on labor, delivery and health of the newborn. J Sports Med Phys Fitness. 1991;31(1):95-9.<br>89. Kobe H, Nakai A, Koshino T, Araki T. Effect of regular maternal exercise on lipid peroxidation levels and antioxidant enzymatic activities before and after delivery. J Nippon Med Sch. 2002;69(6):5428.<br>90. Dale E, Mullinax KM, Bryan DH. Exercise during pregnancy: effects on the fetus. Canadian Journal of Applied Sport Sciences. 1982;7(2):98-103.<br>91. Pomerance JJ, Gluck L, Lynch VA. Physical fitness in pregnancy: its effect on pregnancy outcome. American Journal of Obstetrics & Gynecology. 1974;44(3):383-7.

Review	References of included behavioural interventions delivered in pregnancy
	92. Lawani MM, Alihonou E, Akplogan B, Poumarat G, Okou L, Adjadi N. [Effect of antenatal gymnastics on childbirth: a study on 50 sedentary women in the Republic of Benin during the second and third quarters of pregnancy]. Sante. 2003;13(4):235-41.<br>93. Abirami P, Judie A. Integrated approach of yoga therapy on maternal and fetal outcome in gestational diabetes mellitus. International Journal of Pharmaceutical and Clinical Research. 2015;7(6):377-82.<br>94. Bo K, Fleten C, Nystad W. Effect of antenatal pelvic floor muscle training on labor and birth. Obstet Gynecol. 2009;113(6):1279-84.<br>95. Mason L, Roe B, Wong H, Davies J, Bamber J. The role of antenatal pelvic floor muscle exercises in prevention of postpartum stress incontinence: a randomised controlled trial. J Clin Nurs. 2010;19(1920):2777-86.<br>96. To WWK, Wong MWN. Bone mineral density changes during pregnancy in actively exercising women as measured by quantitative ultrasound. Archives of Gynecology and Obstetrics. 2012;286(2):357-63.<br>97. Vollebregt KC, Wolf H, Boer K, van der Wal MF, Vrijkotte TGM, Bonsel GJ. Does physical activity in leisure time early in pregnancy reduce the incidence of preeclampsia or gestational hypertension? Acta Obstetricia Et Gynecologica Scandinavica. 2010;89(2):261-7.<br>98. Haakstad LA, Bo K. Effect of regular exercise on prevention of excessive weight gain in pregnancy: a randomised controlled trial. Eur J Contracept Reprod Health Care. 2011;16(2):116-25.<br>99. Gaston A, Prapavessis H. Tired, moody and pregnant? Exercise may be the answer. Psychol Health. 2013;28(12):1353-69.<br>100. Choi J, Lee JH, Vittinghoff E, Fukuoka Y. mHealth Physical Activity Intervention: A Randomized Pilot Study in Physically Inactive Pregnant Women. Maternal & Child Health Journal. 2016;20(5):1091101.<br>101. Guszkowska M, Sempolska K, Zaremba A, Langwald M. Exercise or relaxation? Which is more effective in improving the emotional state of pregnant women? 2013.<br>102. Guszkowska M. PHYSICAL ACTIVITY IN RELATION TO AFFECTIVE STATES AND LABOR ANXIETY IN PREGNANT WOMEN. Med Sport. 2011;15(3):114-8.<br>103. Wallace AM, Boyer DB, Dan A, Holm K. Aerobic exercise, maternal self-esteem, and physical discomforts during pregnancy. J Nurse Midwifery. 1986;31(6):255-62.<br>104. Ferrara A, Hedderson MM, Brown SD, Albright CL, Ehrlich SF, Tsai AL, et al. The Comparative Effectiveness of Diabetes Prevention Strategies to Reduce Postpartum Weight Retention in Women With Gestational Diabetes Mellitus: The Gestational Diabetes' Effects on Moms (GEM) Cluster Randomized Controlled Trial. Diabetes Care. 2016;39(1):65-74.<br>105. Eggen MH, Stuge B, Mowinckel P, Jensen KS, Hagen KB. Can supervised group exercises including ergonomic advice reduce the prevalence and severity of low back pain and pelvic girdle pain in pregnancy? A randomized controlled trial. Phys Ther. 2012;92(6):781-90.<br>106. Uebelacker LA, Battle CL, Sutton KA, Magee SR, Miller IW. A pilot randomized controlled trial comparing prenatal yoga to perinatal health education for antenatal depression. Arch Womens Ment Health. 2016;19(3):543-7.<br>107. Peterson CD, Haas M, Gregory WT. A pilot randomized controlled trial comparing the efficacy of exercise, spinal manipulation, and neuro emotional technique for the treatment of pregnancy-related low back pain. Chiropr Man Therap. 2012;20(1):18.<br>108. Youngwanichsetha S, Phumdoung S, Ingkathawornwong T. The effects of mindfulness eating and yoga exercise on blood sugar levels of pregnant women with gestational diabetes mellitus. Appl Nurs Res. 2014;27(4):227-30.<br>109. Ong MJ, Guelfi KJ, Hunter T, Wallman KE, Fournier PA, Newnham JP. Supervised home-based exercise may attenuate the decline of glucose tolerance in obese pregnant women. Diabetes Metab. 2009;35(5):418-21.<br>110. O'Connor PJ, Poudevigne MS, Cress ME, Motl RW, Clapp JF, 3rd. Safety and efficacy of supervised strength training adopted in pregnancy. J Phys Act Health. 2011;8(3):309-20.<br>111. Lynch AM, Goodman C, Choy PL, Dawson B, Newnham JP, McDonald S, et al. Maternal physiological responses to swimming training during the second trimester of pregnancy. Res Sports Med. 2007;15(1):33-45.<br>112. Battle CL, Uebelacker LA, Magee SR, Sutton KA, Miller IW. Potential for prenatal yoga to serve as an intervention to treat depression during pregnancy. Womens Health Issues. 2015;25(2):134-41.<br>113. Banerjee A, V. Mahalakshmi V, R. Baranitharan R. Effect of Antenatal Exercise Program with and without Abdominal Strengthening Exercises on Diastasis Rectus Abdominis - A Post Partum Follow Up. Indian Journal of Physiotherapy & Occupational Therapy. 2013;7(4):123-6.<br>114. Chiarello CM, Falzone LA, McCaslin KE, Patel MN, Ulery KR. The effects of an exercise program on diastasis recti abdominis in pregnant women [corrected] [published erratum appears in J WOMENS HEALTH PHYS THER 2005;29(3):76]. 2005.<br>115. Putnam KF, Mueller LA, Magann EF, et al. Evaluating effects of self-reported domestic physical activity on pregnancy and neonatal outcomes in "stay at home" military wives. Mil Med. 2013;178(8):893-8.
Davenport *et al.* 2018c [49]	1. Mottola MF, Inglis S, Brun CR, Hammond JA. Physiological and metabolic responses of late pregnant women to 40 min of steady-state exercise followed by an oral glucose tolerance perturbation. J Appl Physiol (1985). 2013;115(5):597-604.<br>2. Lotgering FK, Spinnewijn WE, Struijk PC, Boomsma F, Wallenburg HC. Respiratory and metabolic responses to endurance cycle exercise in pregnant and postpartum women. Int J Sports Med. 1998;19(3):193-8.<br>3. Artal R, Wiswell R, Romem Y. Hormonal responses to exercise in diabetic and nondiabetic pregnant patients. 1985.<br>4. Avery MD, Walker AJ. Acute effect of exercise on blood glucose and insulin levels in women with gestational diabetes. 2001.<br>5. Cowett RM, Carpenter MW, Carr S, et al. Glucose and lactate kinetics during a short exercise bout in pregnancy. 1996.<br>6. Soultanakis HN, Artal R, Wiswell RA. Prolonged exercise in pregnancy: glucose homeostasis, ventilatory and cardiovascular responses. Semin Perinatol. 1996;20(4):315-27.<br>7. Halse RE, Wallman KE, Newnham JP, Guelfi KJ. Home-based exercise training improves capillary glucose profile in women with gestational diabetes. Med Sci Sports Exerc. 2014;46(9):1702-9.<br>8. Jovanovic-Peterson L, Durak EP, Peterson CM. Randomized trial of diet versus diet plus cardiovascular conditioning on glucose levels in gestational diabetes. Am J Obstet Gynecol. 1989;161(2):415-9.<br>9. Ruchat SM, Davenport MH, Giroux I, Hillier M, Batada A, Sopper MM, et al. Effect of exercise intensity and duration on capillary glucose responses in pregnant women at low and high risk for gestational diabetes. Diabetes Metab Res Rev. 2012;28(8):669-78.<br>10. Davenport MH, Mottola MF, McManus R, Gratton R. A walking intervention improves capillary glucose control in women with gestational diabetes mellitus: a pilot study. Appl Physiol Nutr Metab. 2008;33(3):511-7.

Review	References of included behavioural interventions delivered in pregnancy
	11. Giroux I, Inglis SD, Lander S, Gerrie S, Mottola MF. Dietary intake, weight gain, and birth outcomes of physically active pregnant women: a pilot study. Appl Physiol Nutr Metab. 2006;31(5):483-9.<br>12. Wolfe LA, Heenan AP, Bonen A. Aerobic conditioning effects on substrate responses during graded cycling in pregnancy. Can J Physiol Pharmacol. 2003;81(7):696-703.<br>13. Bessinger RC, McMurray RG, Hackney AC. Substrate utilization and hormonal responses to moderate intensity exercise during pregnancy and after delivery. 2002.<br>14. Clapp JF, Wesley M, Sleamaker RH. Thermoregulatory and metabolic responses to jogging prior to and during pregnancy. Medicine & Science in Sports & Exercise. 1987;19(2):124???30.<br>15. Clapp JF, 3rd. Effect of dietary carbohydrate on the glucose and insulin response to mixed caloric intake and exercise in both nonpregnant and pregnant women. Diabetes Care. 1998;21 Suppl 2:B107-12.<br>16. Artal R, Platt LD, Sperling M, Kammula RK, Jilek J, Nakamura R. I. Maternal cardiovascular and metabolic responses in normal pregnancy. 1981.<br>17. Artal R, Rutherford S, Romem Y, Kammula RK, Dorey FJ, Wiswell RA. Fetal heart rate responses to maternal exercise. American Journal of Obstetrics & Gynecology. 1986.<br>18. Bonen A, Campagna P, Gilchrist L, Young DC, Beresford P. Substrate and endocrine responses during exercise at selected stages of pregnancy. 1992.<br>19. Halse RE, Wallman KE, Newnham JP, Guelfi KJ. Pregnant women exercise at a higher intensity during 30 min of self-paced cycling compared with walking during late gestation: implications for 2 h postprandial glucose levels. 2013.<br>20. Hauth JC, Gilstrap LC, Widmer K. Fetal heart rate reactivity before and after maternal jogging during the third trimester. American Journal of Obstetrics & Gynecology. 1982.<br>21. Jovanovic L, Kessler A, Peterson CM. Human maternal and fetal response to graded exercise. J Appl Physiol (1985). 1985;58(5):1719-22.<br>22. Lesser KB, Gruppuso PA, Terry RB, Carpenter MW. Exercise fails to improve postprandial glycemic excursion in women with gestational diabetes. J Matern Fetal Med. 1996;5(4):211-7.<br>23. McMurray RG, Hackney AC, Guion WK, Katz VL. Metabolic and hormonal responses to low-impact aerobic dance during pregnancy. Med Sci Sports Exerc. 1996;28(1):41-6.<br>24. Young JC, Treadway JL. The effect of prior exercise on oral glucose tolerance in late gestational women. Eur J Appl Physiol Occup Physiol. 1992;64(5):430-3.<br>25. Bung P, Artal R, Khodiguian N, Kjos S. Exercise in gestational diabetes. An optional therapeutic approach? Diabetes. 1991;40 Suppl 2:182.<br>26. de Barros MC, Lopes MA, Francisco RP, Sapienza AD, Zugaib M. Resistance exercise and glycemic control in women with gestational diabetes mellitus. Am J Obstet Gynecol. 2010;203(6):556 e1-6.<br>27. Callaway LK, Colditz PB, Byrne NM, Lingwood BE, Rowlands IJ, Foxcroft K, et al. Prevention of gestational diabetes: feasibility issues for an exercise intervention in obese pregnant women. Diabetes Care. 2010;33(7):1457-9.<br>28. Cordero Y, Mottola MF, Vargas J, Blanco M, Barakat R. Exercise Is Associated with a Reduction in Gestational Diabetes Mellitus. Med Sci Sports Exerc. 2015;47(7):1328-33.<br>29. Daniel JA, Dikki CE, Ibrahim W. Aerobic dance exercise improves blood glucose level in pregnant women with gestational diabetes mellitus. 2014.<br>30. Garnaes KK, Morkved S, Salvesen O, Moholdt T. Exercise Training and Weight Gain in Obese Pregnant Women: A Randomized Controlled Trial (ETIP Trial). PLoS Med. 2016;13(7):e1002079.<br>31. Guelfi KJ, Ong MJ, Crisp NA, Fournier PA, Wallman KE, Grove JR, et al. Regular Exercise to Prevent the Recurrence of Gestational Diabetes Mellitus: A Randomized Controlled Trial. Obstet Gynecol. 2016;128(4):819-27.<br>32. Hanan EM, Adly Ali S, Mohamed Mostafa R. Effect of Antenatal Exercises on Umbilical Blood Flow and Neonate Wellbeing in Diabetic Pregnant Women. Indian Journal of Physiotherapy & Occupational Therapy. 2012.<br>33. Ko CW, Napolitano PG, Lee SP, Schulte SD, Ciol MA, Beresford SA. Physical activity, maternal metabolic measures, and the incidence of gallbladder sludge or stones during pregnancy: a randomized trial. Am J Perinatol. 2014;31(1):39-48.<br>34. Oostdam N, van Poppel MN, Wouters MG, Eekhoff EM, Bekedam DJ, Kuchenbecker WK, et al. No effect of the FitFor2 exercise programme on blood glucose, insulin sensitivity, and birthweight in pregnant women who were overweight and at risk for gestational diabetes: results of a randomised controlled trial. BJOG. 2012;119(9):1098-107.<br>35. Ong MJ, Guelfi KJ, Hunter T, Wallman KE, Fournier PA, Newnham JP. Supervised home-based exercise may attenuate the decline of glucose tolerance in obese pregnant women. 2009.<br>36. Wang C, Wei Y, Zhang X, Zhang Y, Xu Q, Su S, et al. Effect of Regular Exercise Commenced in Early Pregnancy on the Incidence of Gestational Diabetes Mellitus in Overweight and Obese Pregnant Women: A Randomized Controlled Trial. Diabetes Care. 2016;39(10):e163-4.<br>37. Abirami P, Judie A. Reduction of risk on newly detected gestational diabetes mellitus by multi model intervention - A hospital based study. 2014.<br>38. Bo S, Rosato R, Ciccone G, Canil S, Gambino R, Poala CB, et al. Simple lifestyle recommendations and the outcomes of gestational diabetes. A 2 x 2 factorial randomized trial. Diabetes Obes Metab. 2014;16(10):1032-5.<br>39. Hawkins M, Hosker M, Marcus BH, Rosal MC, Braun B, Stanek EJ, 3rd, et al. A pregnancy lifestyle intervention to prevent gestational diabetes risk factors in overweight Hispanic women: a feasibility randomized controlled trial. Diabet Med. 2015;32(1):108-15.<br>40. Koivusalo SB, Rono K, Klemetti MM, et al. Gestational Diabetes Mellitus Can Be Prevented by Lifestyle Intervention: The Finnish Gestational Diabetes Prevention Study (RADIEL): A Randomized Controlled Trial. Diabetes Care. 2016;39(1):24-30.<br>41. Luoto R, Kinnunen TI, Aittasalo M, Kolu P, Raitanen J, Ojala K, et al. Primary prevention of gestational diabetes mellitus and large-for-gestational-age newborns by lifestyle counseling: a cluster-randomized controlled trial. PLoS Med. 2011;8(5):e1001036.<br>42. Poston L, Bell R, Croker H, Flynn AC, Godfrey KM, Goff L, et al. Effect of a behavioural intervention in obese pregnant women (the UPBEAT study): a multicentre, randomised controlled trial. Lancet Diabetes Endocrinol. 2015;3(10):767-77.<br>43. Stafne SN, Salvesen KA, Romundstad PR, Eggebo TM, Carlsen SM, Morkved S. Regular exercise during pregnancy to prevent gestational diabetes: a randomized controlled trial. Obstet Gynecol. 2012;119(1):29-36.<br>44. Vinter CA, Jorgensen JS, Ovesen P, Beck-Nielsen H, Skytthe A, Jensen DM. Metabolic effects of lifestyle intervention in obese pregnant women. Results from the randomized controlled trial 'Lifestyle in Pregnancy' (LiP). Diabet Med. 2014;31(11):1323-30.

Review	References of included behavioural interventions delivered in pregnancy
	45. Youngwanichsetha S, Phumdoung S, Ingkathawornwong T. The effects of mindfulness eating and yoga exercise on blood sugar levels of pregnant women with gestational diabetes mellitus. Appl Nurs Res. 2014;27(4):227-30.<br>46. Simmons D, Jelsma JG, Galjaard S, Devlieger R, van Assche A, Jans G, et al. Results From a European Multicenter Randomized Trial of Physical Activity and/or Healthy Eating to Reduce the Risk of Gestational Diabetes Mellitus: The DALI Lifestyle Pilot. Diabetes Care. 2015;38(9):1650-6.<br>47. Simmons D, Devlieger R, Assche Av, Jans G, Galjaard S, Corcoy R, et al. Effect of physical activity and/or healthy eating on GDM risk: The DALI Lifestyle Study. Journal of Clinical Endocrinology & Metabolism. 2016.<br>48. Avery MD, Leon AS, Kopher RA. Effects of a partially home-based exercise program for women with gestational diabetes. Obstet Gynecol. 1997;89(1):10-5.<br>49. Ferrara A, Hedderson MM, Albright CL, Ehrlich SF, Quesenberry CP, Jr., Peng T, et al. A pregnancy and postpartum lifestyle intervention in women with gestational diabetes mellitus reduces diabetes risk factors: a feasibility randomized control trial. Diabetes Care. 2011;34(7):1519-25.<br>50. Brankston GN, Mitchell BF, Ryan EA, Okun NB. Resistance exercise decreases the need for insulin in overweight women with gestational diabetes mellitus. 2004.<br>51. Sagedal LR, Overby NC, Bere E, Torstveit MK, Lohne-Seiler H, Smastuen M, et al. Lifestyle intervention to limit gestational weight gain: the Norwegian Fit for Delivery randomised controlled trial. BJOG. 2017;124(1):97-109.<br>52. Artal R, Catanzaro RB, Gavard JA, Mostello DJ, Friganza JC. A lifestyle intervention of weight-gain restriction: diet and exercise in obese women with gestational diabetes mellitus. Appl Physiol Nutr Metab. 2007;32(3):596-601.<br>53. Mustila T, Raitanen J, Keskinen P, Saari A, Luoto R. Pragmatic controlled trial to prevent childhood obesity in maternity and child health care clinics: pregnancy and infant weight outcomes (the VACOPP Study). BMC Pediatr. 2013;13:80.<br>54. Anjana RM, Sudha V, Lakshmipriya N, et al. Physical activity patterns and gestational diabetes outcomes - The wings project. Diabetes Research & Clinical Practice. 2016.<br>55. Clapp JF. The changing thermal response to endurance exercise during pregnancy. American Journal of Obstetrics and Gynecology. 1991;165(6):1684-9.<br>56. Clapp JF, 3rd. Long-term outcome after exercising throughout pregnancy: fitness and cardiovascular risk. Am J Obstet Gynecol. 2008;199(5):489 e1-6.<br>57. Makela J, Lagstrom H, Kaljonen A, Simell O, Niinikoski H. Hyperglycemia and lower diet quality in pregnant overweight women and increased infant size at birth and at 13 months of age--STEPS study. 2013.<br>58. Medek H, Halldorsson T, Gunnarsdottir I, Geirsson RT. Physical activity of relatively high intensity in mid-pregnancy predicts lower glucose tolerance levels. Acta Obstet Gynecol Scand. 2016;95(9):1055-62.
Du *et al.* 2019 [50]	1. Ong MJ, Guelfi KJ, Hunter T, Wallman KE, Fournier PA, Newnham JP. Supervised home-based exercise may attenuate the decline of glucose tolerance in obese pregnant women. Diabetes Metab. 2009;35:418-421.<br>2. Callaway LK, Colditz PB, Byrne NM, et al. Prevention of gestational diabetes: feasibility issues for an exercise intervention in obese pregnant women. Diabetes Care. 2010;33:1457-1459.<br>3. Renault KM, Norgaard K, Nilas L, et al. The Treatment of Obese Pregnant Women (TOP) study: a randomized controlled trial of the effect of physical activity intervention assessed by pedometer with or without dietary intervention in obese pregnant women. Am J Obstet Gynecol. 2014;210:134.e131-134.e139.<br>4. Bisson M, Almeras N, Dufresne SS, et al. A 12-week exercise program for pregnant women with obesity to improve physical activity levels: an open randomised preliminary study. PloS One. 2015;10:e0137742.<br>5. Oostdam N, Poppel MN, Wouters MG, et al. No effect of the FitFor2 exercise programme on blood glucose, insulin sensitivity, and birthweight in pregnant women who were overweight and at risk for gestational diabetes: results of a randomised controlled trial. BJOG. 2012;119:1098-1107.<br>6. Kong KL, Campbell CG, Foster RC, Peterson AD, LanninghamFoster L. A pilot walking program promotes moderate-intensity physical activity during pregnancy. Med Sci Sports Exerc. 2014;46:462-471.<br>7. Daly N, Farren M, McKeating A, O'Kelly R, Stapleton M, Turner MJ. A medically supervised pregnancy exercise intervention in obese women: a randomized controlled trial. Obstet Gynecol. 2017;130:1001-1010.<br>8. Wang C, Wei Y, Zhang X, et al. A randomized clinical trial of exercise during pregnancy to prevent gestational diabetes mellitus and improve pregnancy outcome in overweight and obese pregnant women. Am J Obstet Gynecol. 2017;216:340-351.<br>9. Nascimento SL, Surita FG, Parpinelli M, Siani S, Pinto e Silva JL. The effect of an antenatal physical exercise programme on maternal/perinatal outcomes and quality of life in overweight and obese pregnant women: a randomised clinical trial. BJOG. 2011;118:1455-1463.<br>10. Dekker Nitert M, Barrett HL, Denny KJ, McIntyre HD, Callaway LK. Exercise in pregnancy does not alter gestational weight gain, MCP-1 or leptin in obese women. Aust N Z J Obstet Gynaecol. 2015;55:27-33.<br>11. Seneviratne SN, Jiang Y, Derraik J, et al. Effects of antenatal exercise in overweight and obese pregnant women on maternal and perinatal outcomes: a randomised controlled trial. BJOG. 2016;123:588-597.<br>12. Garnaes KK, Nyrnes SA, Salvesen KA, Salvesen O, Morkved S, Moholdt T. Effect of supervised exercise training during pregnancy on neonatal and maternal outcomes among overweight and obese women. Secondary analyses of the ETIP trial: a randomised controlled trial. PloS One. 2017;12:e0173937.<br>13. Simmons D, Devlieger R, van Assche A, et al. Effect of physical activity and/or healthy eating on GDM risk: the DALI lifestyle study. J Clin Endocrinol Metab. 2017;102:903-913.
Guillemette *et al.* 2018 [51]	1. Baciu k EP, Pereira RI, Cecatti JG, Braga AF, Cavalcante SR. Water aerobics in pregnancy: cardiovascular response, labor and neonatal outcomes. Reprod Health. 2008; https://doi.org/10.1186/1742-4755-5-10.<br>2. Barakat R, Lucia A, Ruiz JR. Resistance exercise training during pregnancy and newborn's birth size: a randomised controlled trial. International journal of obesity. 2005) 2009; https://doi.org/10.1038/ijo.2009.150.<br>3. Barakat R, Pelaez M, Cordero Y, Perales M, Lopez C, Coteron J, et al. Exercise during pregnancy protects against hypertension and macrosomia: randomized clinical trial. Am J Obstet Gynecol. 2016; https://doi.org/10. 1016/j.ajog.2015.11.039.<br>4. Barakat R, Pelaez M, Lopez C, Montejo R, Coteron J. Exercise during pregnancy reduces the rate of cesarean and instrumental deliveries: results of a randomized controlled trial. Journal of maternal-fetal & neonatal medicine. 2013; https://doi.org/10.3109/14767058.2012.696165.

Review	References of included behavioural interventions delivered in pregnancy
	5. Avery MD, Leon AS, Kopher RA. Effects of a partially home-based exercise program for women with gestational diabetes. Obstet Gynecol. 1997;89(1):10-5.<br>6. Barakat R, Perales M, Bacchi M, Coteron J, Refoyo I. A program of exercise throughout pregnancy. Is it safe to mother and newborn? American journal of health promotion: AJHP. 2014;29(1):2–8.<br>7. Clapp JF, Kim H, Burciu B, Lopez B. Beginning regular exercise in early pregnancy: effect on fetoplacental growth. Am J Obstet Gynecol. 2000; https://doi.org/10.1067/mob.2000.107096.<br>8. Cordero FY, Mottola FM, Vargas FJ, Blanco FM, Barakat FR. Exercise is associated with a reduction in gestational diabetes mellitus. Med Sci Sports Exerc. 2015;47(7):1328–33. https://doi.org/10.1249/MSS.0000000000000547.<br>9. Daly N, Farren M, McKeating A, O'Higgins A, Mullaney L, Turner MJ. Effect of a randomized controlled trial of an intensive medically supervised exercise program designed to improve maternal glucose control on gestational weight gain. Am J Obstet Gynecol. 2017;216(1 Supplement 1):S24.<br>10. de Barros MC, Lopes MA, Francisco RP, Sapienza AD, Zugaib M. Resistance exercise and glycemic control in women with gestational diabetes mellitus. Am J Obstet Gynecol. 2010;203:556–6. e1–6<br>11. De Oliveria Melo AS, Silva JLP, Tavares JS, Barros VO, Leite DFB, Amorim MMR. Effect of a physical exercise program during pregnancy on uteroplacental and fetal blood flow and fetal growth: a randomized controlled trial. Obstet Gynecol. 2012;120(2 PART 1):302–10.<br>12. Erkkola R, Mäkelä M. Heart volume and physical fitness of parturients. Ann Clin Res. 1976;8(1):15–21.<br>13. Garnaes KK, Nyrnes SA, Salvesen KA, Salvesen O, Morkved S, Moholdt T. Effect of supervised exercise training during pregnancy on neonatal and maternal outcomes among overweight and obese women. Secondary analyses of the ETIP trial: a randomised controlled trial. PLoS One. 2017;12(3) (no pagination):e0173937.<br>14. Garshasbi A, Faghih Zadeh S. The effect of exercise on the intensity of low back pain in pregnant women. Int J Gynecol Obstet. 2005;88(3):271–5.<br>15. Ghodsi Z, Asltoghiri M. Effects of aerobic exercise training on maternal and neonatal outcome: a randomized controlled trial on pregnant women in Iran. JPMA The Journal of the Pakistan Medical Association. 2014;64(9):1053–6.<br>16. Guelfi KJ, Ong MJ, Crisp NA, Fournier PA, Wallman KE, Grove JR, et al. Regular exercise to prevent the recurrence of gestational diabetes mellitus: a randomized controlled trial. Obstet Gynecol. 2016;128(4):819–27. https:// doi.org/10.1097/aog.0000000000001632.<br>17. Haakstad LA, Bo K. Exercise in pregnant women and birth weight: a randomized controlled trial. BMC Pregnancy & Childbirth. 2011;11:66.<br>18. Hellenes OM, Vik T, Løhaugen GC, Salvesen K, Stafne SN, Mørkved S, et al. Regular moderate exercise during pregnancy does not have an adverse effect on the neurodevelopment of the child. Acta paediatrica (Oslo, Norway : 1992). 2015; https://doi.org/10.1111/apa.12890.<br>19. Hollingsworth DR, Moore TR. Postprandial walking exercise in pregnant insulin-dependent (type I) diabetic women: reduction of plasma lipid levels but absence of a significant effect on glycemic control. American Journal of Obstetrics & Gynecology. 1987;157(6):1359–63.<br>20. Hopkins SA, Baldi JC, Cutfield WS, McCowan L, Hofman PL. Exercise training in pregnancy reduces offspring size without changes in maternal insulin sensitivity. J Clin Endocrinol Metab. 2010; https://doi.org/10.1210/jc.2009-2255.<br>21. Kasawara KT, Burgos CS, do Nascimento SL, Ferreira NO, Surita FG, Pinto ESJL. Maternal and perinatal outcomes of exercise in pregnant women with chronic hypertension and/or previous preeclampsia: a randomized controlled trial. ISRN obstetrics and gynecology. 2013;2013:857047. https:// doi.org/10.1155/2013/857047.<br>22. Kong KL, Campbell CG, Foster RC, Peterson AD, Lanningham-Foster L. A pilot walking program promotes moderate-intensity physical activity during pregnancy. Med Sci Sports Exerc. 2014;46(3):462–71.<br>23. Labonte-Lemoyne E, Curnier D, Ellemberg D. Exercise during pregnancy enhances cerebral maturation in the newborn: a randomized controlled trial. Journal of Clinical & Experimental Neuropsychology: official. J Int Neuropsychol Soc. 2017;39(4):347–54.<br>24. Marquez-Sterling S, Perry AC, Kaplan TA, Halberstein RA, Signorile JF. Physical and psychological changes with vigorous exercise in sedentary primigravidae. Med Sci Sports Exerc. 2000;<br>25. Murtezani A, Paçarada M, Ibraimi Z, Nevzati A, Abazi N. The impact of exercise during pregnancy on neonatal outcomes: a randomized controlled trial. J Sports Med Phys Fitness. 2014;54(6):802–8.<br>26. Nascimento SL, Surita FG, Parpinelli M, Siani S, Pinto e Silva JL. The effect of an antenatal physical exercise programme on maternal/perinatal outcomes and quality of life in overweight and obese pregnant women: a randomised clinical trial. BJOG: An International Journal of Obstetrics and Gynaecology. 2011;118(12):1455–63.<br>27. Oostdam N, Poppel MN, Wouters MG, Eekhoff EM, Bekedam DJ, Kuchenbecker WK, et al. No effect of the FitFor2 exercise programme on blood glucose, insulin sensitivity, and birthweight in pregnant women who were overweight and at risk for gestational diabetes: results of a randomised controlled trial. BJOG : an international journal of obstetrics and gynaecology. 2012; https://doi.org/10.1111/j.1471-0528.2012.03366.x.<br>28. Perales M, Calabria I, Lopez C, Franco E, Coteron J, Barakat R. Regular exercise throughout pregnancy is associated with a shorter first stage of labor. Am J Health Promot. 2017; https://doi.org/10.4278/ajhp.140221-QUAN-79.<br>29. Petrov Fieril K, Glantz A, Fagevik Olsen M. The efficacy of moderate-tovigorous resistance exercise during pregnancy: a randomized controlled trial. Acta Obstet Gynecol Scand. 2015;94(1):35–42.<br>30. Pinzon DC, Zamora K, Martinez JH, Florez-Lopez ME, de Plata AC, Mosquera M, et al. Type of delivery and gestational age is not affected by pregnant Latin-American women engaging in vigorous exercise: a secondary analysis of data from a controlled randomized trial. Revista de salud publica (Bogota, Colombia). 2012;14(5):731–43.<br>31. Price BB, Amini SB, Kappeler K. Exercise in pregnancy: effect on fitness and obstetric outcomes—a randomized trial. Medicine and science in sports and exercise. 2012; https://doi.org/10.1249/MSS.0b013e318267ad67.<br>32. Ramos JG, Bgeginski R, Opperman ML, Martins-Costa S, Delevatti R, Schuch R, et al. Effect of aerobic training in pregnant women diagnosed with gestational diabetes: a preliminary report. Pregnancy Hypertension. 2015;5(1):105.

Review	References of included behavioural interventions delivered in pregnancy
	33. Seneviratne SN, Jiang Y, Derraik J, McCowan L, Parry GK, Biggs JB, et al. Effects of antenatal exercise in overweight and obese pregnant women on maternal and perinatal outcomes: a randomised controlled trial. BJOG : an international journal of obstetrics and gynaecology. 2016; https://doi.org/10. 1111/1471-0528.13738.<br>34. Ussher M, Lewis S, Aveyard P, Manyonda I, West R, Lewis B, et al. Physical activity for smoking cessation in pregnancy: randomised controlled trial. BMJ (Clinical research ed). 2015;350:h2145.<br>35. Wang C, Wei Y, Zhang X, Zhang Y, Xu Q, Sun Y, et al. A randomized clinical trial of exercise during pregnancy to prevent gestational diabetes mellitus and improve pregnancy outcome in overweight and obese pregnant women. Am J Obstet Gynecol. 2017;216(4):340–51.<br>36. Kihlstrand M, Stenman B, Nilsson S, Axelsson O. Water-gymnastics reduced the intensity of back/low back pain in pregnant women. Acta Obstet Gynecol Scand. 1999;78(3):180. https://doi.org/10.1080/j. 1600-0412.1999.780302.x.<br>37. Ruiz JR, Perales M, Pelaez M, Lopez C, Lucia A, Barakat R. Supervised exercise-based intervention to prevent excessive gestational weight gain: a randomized controlled trial. Mayo Clin Proc. 2013;88(12):1388–97. https:// doi.org/10.1016/j.mayocp.2013.07.020.<br>38. Sklempe Kokic I, Ivanisevic M, Biolo G, Simunic B, Kokic T, Pisot R. Combination of a structured aerobic and resistance exercise improves glycaemic control in pregnant women diagnosed with gestational diabetes mellitus. A randomised controlled trial. Women and birth. 2017; https://doi. org/10.1016/j.wombi.2017.10.004.<br>39. Santos IA, Stein R, Fuchs SC, Duncan BB, Ribeiro JP, Kroeff LR, et al. Aerobic exercise and submaximal functional capacity in overweight pregnant women: a randomized trial. Obstet Gynecol. 2005;106(2):243–9. https://doi. org/10.1097/01.aog.0000171113.36624.86.
Michel *et al.* 2019 [52]	1. Althuizen E, Van Der Wijden CL, Van Mechelen W, Seidell JC, Van Poppel MNM. The effect of a counselling intervention on weight changes during and after pregnancy: a randomised trial. BJOG. 2013;120(1):92-99.<br>2. Horan MK, McGowan CA, Gibney ER, Byrne J, Donnelly JM, McAuliffe FM. Maternal nutrition and glycaemic index during pregnancy impacts on offspring adiposity at 6 months of age-analysis from the ROLO randomised controlled trial. Nutrients. 2016;8(1):7.<br>3. Horan M, Donnelly J, McGowan C, Gibney E, McAuliffe F. The association between maternal nutrition and lifestyle during pregnancy and 2-year-old offspring adiposity: analysis from the ROLO study. J Public Health. 2016;24(5):427-436.<br>4. Huang TT, Yeh CY, Tsai YC. A diet and physical activity intervention for preventing weight retention among Taiwanese childbearing women: a randomised controlled trial. Midwifery. 2011;27(2):257-264.<br>5. Ickovics JR, Earnshaw V, Lewis JB, et al. Cluster randomized controlled trial of group prenatal care: perinatal outcomes among adolescents in New York City health centers. Am J Public Health. 2016;106(2):359-365.<br>6. Luoto R, Kinnunen TI, Aittasalo M, et al. Primary prevention of gestational diabetes mellitus and large-for-gestational-age newborns by lifestyle counseling: a cluster-randomized controlled trial. PLoS Med. 2011;8(5):e1001036.<br>7. Puhkala J, Raitanen J, Kolu P, Tuominen P, Husu P, Luoto R. Metabolic syndrome in Finnish women 7 years after a gestational diabetes prevention trial. BMJ Open. 2017;7(3):e014565.<br>8. McEachan RRC, Santorelli G, Bryant M, et al. The HAPPY (Healthy and Active Parenting Programmme for early Years) feasibility randomised control trial: acceptability and feasibility of an intervention to reduce infant obesity. BMC Public Health. 2016;16(1):211-211.<br>9. Patel N, Godfrey KM, Pasupathy D, et al. Infant adiposity following a randomised controlled trial of a behavioural intervention in obese pregnancy. Int J Obes (Lond). 2017;41(7):1018-1026.<br>10. Peccei A, Blake-Lamb T, Rahilly D, Hatoum I, Bryant A. Intensive prenatal nutrition counseling in a community health setting: a randomized controlled trial. Obstet Gynecol. 2017;130(2):423-432.<br>11. Phelan S, Phipps MG, Abrams B, Darroch F, Schaffner A, Wing RR. Randomized trial of a behavioral intervention to prevent excessive gestational weight gain: the Fit for Delivery Study. Am J Clin Nutr. 2011;93(4):772-779.<br>12. Phelan S, Phipps MG, Abrams B, et al. Does behavioral intervention in pregnancy reduce postpartum weight retention? Twelve-month outcomes of the Fit for Delivery randomized trial. Am J Clin Nutr. 2014; 99(2):302-311.<br>13. Rauh K, Gabriel E, Kerschbaum E, et al. Safety and efficacy of a lifestyle intervention for pregnant women to prevent excessive maternal weight gain: a cluster-randomized controlled trial. BMC Pregnancy Childbirth. 2013;13:1-11.<br>14. Rauh K, Günther J, Kunath J, Stecher L, Hauner H. Lifestyle intervention to prevent excessive maternal weight gain: mother and infant follow-up at 12 months postpartum. BMC Pregnancy Childbirth. 2015; 15(1):265-265.<br>15. Ronnberg A, Hanson U, Ostlund I, Nilsson K. Effects on postpartum weight retention after antenatal lifestyle intervention—a secondary analysis of a randomized controlled trial. Acta Obstet Gynecol Scand. 2016;95(9):999-1007.<br>16. Sagedal LR, Sanda B, Overby NC, et al. The effect of prenatal lifestyle intervention on weight retention 12 months postpartum: results of the Norwegian Fit for Delivery randomised controlled trial. BJOG. 2017;124(1):111-121.<br>17. Vesco K, Leo M, Karanja N, et al. One-year postpartum outcomes following a weight management intervention in pregnant women with obesity. Obesity. 2016;24(10):2042-2049.
Ming *et al.* 2018 [53]	1. Cordero Y, Mottola MF, Vargas J, Blamco M, Barakat R. Exercise is associated with a reduction in gestational diabetes mellitus. Med Sci Sports Exerc. 2015;47(7):1328–33.<br>2. Barakat R, Perales M, Bacchi M, Coteron J, Refoyo I. A program of exercise throughout pregnancy. Is it safe to mother and newborn? Am J Health Promot. 2014;29(1):2–8.<br>3. Barakat R, Palaez M, Montejo R, Refoyo I, Coteron J. Exercise throughout pregnancy does not cause preterm delivery: a randomized, controlled trial. J Phys Act Health. 2014;11(5):1012.<br>4. Ruiz JR, Perales M, Pelaez M, Lopez C, Lucia A, Barakat R. Supervised exercise-based intervention to prevent excessive gestational weight gain: a randomized controlled trial. Mayo Clin Proc. 2013;88(12):1388–97.<br>5. Tomic V, Sporis G, Tomic J, Milanovic Z, Zigmundovac-Klaic D, Pantelic C. The effect of maternal exercise during pregnancy on abnormal fetal growth. Croat Med J. 2013;54(4):362–8.

Review	References of included behavioural interventions delivered in pregnancy
	6. Barakat R, Cordero Y, Coteron J, Luaces M, Montejo R. Exercise during pregnancy improves maternal glucose screen at 24–28 weeks: a randomised controlled trial. Br J Sports Med. 2012;46(9):656–61.<br>7. Stafne SN, Salvesen KA, Romundstad PR, Eggebo TM, Caelsen SM, Morkved S. Regular exercise during pregnancy to prevent gestational diabetes: a randomized controlled trial. Obstet Gynecol. 2012;119(1):29–36.<br>8. Barakat R, Pelaez M, Lopez C, Lucia A, Ruiz JR. Exercise during pregnancy and gestational diabetes-related adverse effects: a randomised controlled trial. Br J Sports Med. 2013;47(10):630–6
Morison *et al.* 2018 [54]	1. Poston L, Bell R, Croker H, Flynn AC, Godfrey KM, Goff L et al. Effect of a behavioural intervention in obese pregnant women (the UPBEAT study): a multicentre , randomised controlled trial. Lancet Diabetes Endocrinol. 2015;3:767-77.<br>2. Dodd JM, Turnbull D, McPhee AJ, Deussen AR, Grivell RM, Yelland LN et al. Antenatal lifestyle advice for women who are overweight or obese: LIMIT randomised trial. BMJ. 2014;348:g1285.<br>3. Vesco KK, Karanja N, King JC, Matthew W, Leo MC, Perrin N et al. Efficacy of a group-based intervention for limiting gestational weight gain among obese women: a randomized trial. Obesity. 2015;22:1989-96. doi: 10.1002/ oby.20831.<br>4. Hui A, Back L, Ludwig S, Gardiner P, Sevenhuysen G, Dean H et al. Lifestyle intervention on diet and exercise reduced excessive gestational weight gain in pregnant women under a randomised controlled trial. BJOG. 2012; 119:70-7. doi: 10.1111/j.1471-0528.2011.03184.x.<br>5. Hui AL, Back L, Ludwig S, Gardiner P, Sevenhuysen G, Dean HJ et al. Effects of lifestyle intervention on dietary intake, physical activity level, and gestational weight gain in pregnant women with different pre-pregnancy body mass index in a randomized control trial. BMC Pregnancy Childbirth. 2014;14:331.<br>6. Nascimento SL, Surita FG, Parpinelli M, Siani S, Pinto e Silva JL. The effect of an antenatal physical exercise programme on maternal/perinatal outcomes and quality of life in overweight and obese pregnant women: a randomised clinical trial. BJOG. 2011;118:1455-63.<br>7. Althuizen E, van der Wijden CL, van Mechelen W, Seidell JC, van Poppel MN. The effect of a counselling intervention on weight changes during and after pregnancy: a randomised trial. BJOG. 2013;120:92-9. doi: 10.1111/147 1-0528.12014.<br>8. Ruiz JR, Perales M, Pelaez M, Lopez C, Lucia A, Barakat R. Supervised exercise-based intervention to prevent excessive gestational weight gain: a randomized controlled trial. Mayo Clin Proc. 2013;88:1388-97. doi: 10.1016/j.mayocp.2013.07. 020.<br>9. Sagedal LR, Øverby NC, Bere E, Torstveit MK, LohneSeiler H, Småstuen M et al. Lifestyle intervention to limit gestational weight gain: the Norwegian Fit for Delivery randomised controlled trial. BJOG. 2017;124:97-109.<br>10. Ruchat S, Davenport MH, Giroux I, Hillier M, Batada A, Sopper MM et al. Nutrition and exercise reduce excessive weight gain in normal-weight pregnant women. Med Sci Sports Exerc. 2012;44:1419-26.
Nasiri-Amiri *et al.* 2019 [55]	1. Garnæs KK, Mørkved S, Salvesen Ø, Moholdt T. Exercise training and weight gain in obese pregnant women: a randomized controlled trial (ETIP trial). PLoS Med. 2016;13(7):e1002079.<br>2. Barakat R, Pelaez M, Lopez C, Lucia A, Ruiz JR. Exercise during pregnancy and gestational diabetes-related adverse effects: a randomised controlled trial. Br J Sports Med. 2013;47:630–6.<br>3. Daly N, Farren M, McKeating A, O'kelly R, Stapleton M, Turner MJ. A medically supervised pregnancy exercise intervention in obese women: a randomized controlled trial. Obstet Gynecol. 2017;130(5):1001–10.<br>4. Oostdam N, Van Poppel M, Wouters M, Eekhoff E, Bekedam D, Kuchenbecker W, et al. No effect of the FitFor2 exercise programme on blood glucose, insulin sensitivity, and birthweight in pregnant women who were overweight and at risk for gestational diabetes: results of a randomised controlled trial. BJOG Int J Obstet Gynaecol. 2012;119(9):1098–107.<br>5. Wang C, Wei Y, Zhang X, Zhang Y, Xu Q, Su S, et al. Effect of regular exercise commenced in early pregnancy on the incidence of gestational diabetes mellitus in overweight and obese pregnant women: a randomized controlled trial. Diabetes Care. 2016;39(10):e163–4.<br>6. Simmons D, Devlieger R, Van Assche A, Jans G, Galjaard S, Corcoy R, et al. Effect of physical activity and/or healthy eating on GDM risk: the DALI lifestyle study. J Clin Endocrinol Metab. 2016;102(3):903–13.<br>7. Seneviratne S, Jiang Y, Derraik J, McCowan L, Parry G, Biggs J, et al. Effects of antenatal exercise in overweight and obese pregnant women on maternal and perinatal outcomes: a randomised controlled trial. BJOG Int J Obstet Gynaecol. 2016;123(4):588–97.<br>8. Callaway LK, Colditz PB, Byrne NM, Lingwood BE, Rowlands IJ, Foxcroft K, et al. Prevention of gestational diabetes: feasibility issues for an exercise intervention in obese pregnant women. Diabetes Care. 2010;33(7):1457–9.
Ruchat *et al.* 2018 [56]	1. Saila B. Koivusalo, Kristiina Rönö, Miira M. Klemetti, Risto P. Roine, Jaana Lindström, et al. Gestational Diabetes Mellitus Can Be Prevented by Lifestyle Intervention: The Finnish Gestational Diabetes Prevention Study (RADIEL) Diabetes Care Jan 2016, 39 (1) 24-30; DOI: 10.2337/dc15-0511<br>2. Hopkins SA, Baldi JC, Cutfield WS, McCowan L, Hofman PL. Exercise training in pregnancy reduces offspring size without changes in maternal insulin sensitivity. J Clin Endocrinol Metab. 2010 May;95(5):2080-8. doi: 10.1210/jc.2009-2255. Epub 2010 Mar 24. PubMed PMID: 20335449.<br>3. Guelfi KJ, Ong MJ, Crisp NA, Fournier PA, Wallman KE, Grove JR, Doherty DA, Newnham JP. Regular Exercise to Prevent the Recurrence of Gestational Diabetes Mellitus: A Randomized Controlled Trial. Obstet Gynecol. 2016 Oct;128(4):819-27. doi: 10.1097/AOG.0000000000001632. PubMed PMID: 27607876.<br>4. Asbee SM, Jenkins TR, Butler JR, White J, Elliot M, Rutledge A. Preventing excessive weight gain during pregnancy through dietary and lifestyle counseling: a randomized controlled trial. Obstet Gynecol. 2009 Feb;113(2 Pt 1):305-12. doi: 10.1097/AOG.0b013e318195baef. PubMed PMID: 19155899.<br>5. Bruno, R., Petrella, E., Bertarini, V., Pedrielli, G., Neri, I., and Facchinetti, F. (2017) Adherence to a lifestyle programme in overweight/obese pregnant women and effect on gestational diabetes mellitus: a randomized controlled trial. Maternal & Child Nutrition, 13: e12333. doi: 10.1111/mcn.12333.

Review	References of included behavioural interventions delivered in pregnancy
	6. Dekker Nitert, M., Barrett, H.L., Denny, K.J., McIntyre, H.D., Callaway, L.K. and (2015), Exercise in pregnancy does not alter gestational weight gain, MCP-1 or leptin in obese women. Aust N Z J Obstet Gynaecol, 55: 27-33. doi:10.1111/ajo.12300<br>7. Korpi-Hyövälti E, Heinonen S, Schwab U, Laaksonen DE, Niskanen L. Effect of intensive counselling on physical activity in pregnant women at high risk for gestational diabetes mellitus. A clinical study in primary care. Prim Care Diabetes. 2012 Dec;6(4):261-8. doi: 10.1016/j.pcd.2012.07.004. Epub 2012 Aug 13. PubMed PMID: 22898328.<br>8. Miquelutti MA, Cecatti JG, Makuch MY. Evaluation of a birth preparation program on lumbopelvic pain, urinary incontinence, anxiety and exercise: a randomized controlled trial. BMC Pregnancy Childbirth. 2013 Jul 29;13:154. doi: 10.1186/1471-2393-13-154. PubMed PMID: 23895188; PubMed Central PMCID: PMC3750492<br>9. Oostdam N, van Poppel MN, Wouters MG, Eekhoff EM, Bekedam DJ, Kuchenbecker WK, Quartero HW, Heres MH, van Mechelen W. No effect of the FitFor2 exercise programme on blood glucose, insulin sensitivity, and birthweight in pregnant women who were overweight and at risk for gestational diabetes: results of a randomised controlled trial. BJOG. 2012 Aug;119(9):1098-107. doi: 10.1111/j.1471-0528.2012.03366.x. Epub 2012 May 23. PubMed PMID: 22616913.<br>10. Poston, L., Bell R, Croker H, Flynn AC, Godfrey KM, Goff L, Hayes L, Khazaezadeh N, Nelson SM, Oteng-Ntim E, Pasupathy D, Patel N, Robson SC, Sandall J, Sanders TAB, Sattar N, Seed PT, Wardle J, Whitworth MK, Briley AL on behalf of The UPBEAT Trial Consortium, Effect of a behavioural intervention in obese pregnant women (the UPBEAT study): a multicentre, randomised controlled trial, Lancet Diabetes Endocrinol, 2015, doi: 10.1016/S2213-8587(15)00227-2.<br>11. Nascimento SL, Surita FG, Parpinelli MÂ, et al. The effect of an antenatal physical exercise programme on maternal/perinatal outcomes and quality of life in overweight and obese pregnant women: a randomised clinical trial. BJOG 2011;118:1455–63.<br>12. Bisson M, Alméras N, Dufresne SS, et al. A 12-week exercise program for pregnant women with obesity to improve physical activity levels: an open randomised preliminary study. PLoS One 2015;10:e0137742.<br>13. Cordero Y, Mottola MF, Vargas J, et al. Exercise is associated with a reduction ingestational diabetes mellitus. Med Sci Sports Exerc 2015;47:1328–33.<br>14. Kong KL, Campbell CG, Foster RC, et al. A pilot walking program promotes moderate-intensity physical activity during pregnancy. Med Sci Sports Exerc 2014;46:462–71.<br>15. Perales M, Cordero Y, Vargas M, et al. Exercise and depression in overweight and obese pregnant women: a randomised controlled trial. Arch Medicina Deporte 2015;32(3):156–63.<br>16. Ruiz JR, Perales M, Pelaez M, et al. Supervised exercise-based intervention to prevent excessive gestational weight gain: a randomized controlled trial. Mayo Clin Proc 2013;88:1388–97.<br>17. Renault KM, Nørgaard K, Nilas L, et al. The Treatment of Obese Pregnant Women (TOP) study: a randomized controlled trial of the effect of physical activity intervention assessed by pedometer with or without dietary intervention in obese pregnant women. Am J Obstet Gynecol 2014;210:134.e1–134.e9.<br>18. Perales M, Mateos S, Vargas M, et al. Fetal and maternal heart rate responses to exercise in pregnant women. A randomized controlled trial. Arch Medicina Deporte 2015;32:361–7.<br>19. Haakstad LA, Bo K. Effect of regular exercise on prevention of excessive weight gain in pregnancy: a randomised controlled trial. Eur J Contracept Reprod Health Care 2011;16:116–25.<br>20. Ronnberg AK, Ostlund I, Fadl H, et al. Intervention during pregnancy to reduce excessive gestational weight gain—a randomised controlled trial. BJOG 2015;122:537–44.<br>21. Ussher M, Lewis S, Aveyard P, et al. The London Exercise And Pregnant smokers (LEAP) trial: a randomised controlled trial of physical activity for smoking cessation in pregnancy with an economic evaluation. Health Technol Assess 2015;19:1–136.<br>22. Garnæs KK, Mørkved S, Salvesen Ø, et al. Exercise training and weight gain in obese pregnant women: a randomized controlled trial (ETIP trial). PLoS Med 2016;13:e1002079.<br>23. Simmons D, Devlieger R, van Assche A, et al. Effect of physical activity and/ or healthy eating on GDM risk: the DALI lifestyle study. J Clin Endocrinol Metab 2017;102:903–13.<br>24. Hui AL, Ludwig S, Gardiner P, et al. Community-based exercise and dietary intervention during pregnancy: a pilot study. Can J Diabetes 2006;30:1–7.<br>25. Hui A, Back L, Ludwig S, et al. Lifestyle intervention on diet and exercise reduced excessive gestational weight gain in pregnant women under a randomised controlled trial. BJOG 2012;119:70–7.<br>26. Vinter CA, Jensen DM, Ovesen P, et al. The LiP (Lifestyle in Pregnancy) study: a randomized controlled trial of lifestyle intervention in 360 obese pregnant women. Diabetes Care 2011;34:2502–7.<br>27. Hui AL, Back L, Ludwig S, et al. Effects of lifestyle intervention on dietary intake, physical activity level, and gestational weight gain in pregnant women with different pre-pregnancy body mass index in a randomized control trial. BMC Pregnancy Childbirth 2014;14:331.<br>28. Rauh K, Gabriel E, Kerschbaum E, et al. Safety and efficacy of a lifestyle intervention for pregnant women to prevent excessive maternal weight gain: a cluster randomized controlled trial. BMC Pregnancy Childbirth 2013;13:151.<br>29. Althuizen E, van der Wijden CL, van Mechelen W, et al. The effect of a counselling intervention on weight changes during and after pregnancy: a randomised trial. BJOG 2013;120:92–9.<br>30. Jing W, Huang Y, Liu X, et al. The effect of a personalized intervention on weight gain and physical activity among pregnant women in China. Int J Gynaecol Obstet 2015;129:138–41.<br>31. Petrella E, Malavolti M, Bertarini V, et al. Gestational weight gain in overweight and obese women enrolled in a healthy lifestyle and eating habits program. J maternal, Fetal Neonatal Med 2014;27:1348–52.<br>32. Ferrara A, Hedderson MM, Albright CL, et al. A pregnancy and postpartum lifestyle intervention in women with gestational diabetes mellitus reduces diabetes risk factors: a feasibility randomized control trial. Diabetes Care 2011;34:1519–25.<br>33. Kinnunen TI, Raitanen J, Aittasalo M, et al. Preventing excessive gestational weight gain: a secondary analysis of a cluster-randomised controlled trial. Eur J Clin Nutr 2012;66:1344–50.<br>34. Polley BA, Wing RR, Sims CJ. Randomized controlled trial to prevent excessive weight gain in pregnant women. Int J Obes Relat Metab Disord 2002;26:1494–502.<br>35. Fit for Delivery Study. Am J Clin Nutr 2011;93:772–9.<br>36. Sagedal LR, Øverby NC, Bere E, et al. Lifestyle intervention to limit gestational weight gain: the Norwegian Fit for Delivery randomised controlled trial. BJOG 2017;124:97–109.

Review	References of included behavioural interventions delivered in pregnancy
	37. Smith K, Lanningham-Foster L, Welch A, et al. Web-based behavioral intervention increases maternal exercise but does not prevent excessive gestational weight gain in previously sedentary women. J Phys Act Health 2016;13:587–93.<br>38. Dodd JM, Turnbull D, McPhee AJ, et al. Antenatal lifestyle advice for women who are overweight or obese: LIMIT randomised trial. BMJ 2014;348:g1285.<br>39. Vesco KK, Karanja N, King JC, et al. Efficacy of a group-based dietary intervention for limiting gestational weight gain among obese women: a randomized trial. Obesity 2014;22:1989–96.<br>40. Jackson RA, Stotland NE, Caughey AB, et al. Improving diet and exercise in pregnancy with Video Doctor counseling: a randomized trial. Patient Educ Couns 2011;83:203–9.<br>41. Renault KM, Carlsen EM, Nørgaard K, et al. Intake of sweets, snacks and soft drinks predicts weight gain in obese pregnant women: detailed analysis of the results of a randomised controlled trial. PLoS One 2015;10:e0133041.<br>42. Simmons D, Jelsma JG, Galjaard S, et al. Results from a European multicentre randomized trial of physical activity and/or healthy eating to reduce the risk of gestational diabetes mellitus: the DALI lifestyle pilot. Diabetes Care 2015;38:1650–6.<br>43. Seneviratne SN, Jiang Y, Derraik J, et al. Effects of antenatal exercise in overweight and obese pregnant women on maternal and perinatal outcomes: a randomised controlled trial. BJOG 2016;123:588–97.<br>44. Huang TT, Yeh CY, Tsai YC. A diet and physical activity intervention for preventing weight retention among Taiwanese childbearing women: a randomised controlled trial. Midwifery 2011;27:257–64.<br>45. Phelan S, Phipps MG, Abrams B, et al. Does behavioral intervention in pregnancy reduce postpartum weight retention? Twelve-month outcomes of the Fit for Delivery randomized trial. Am J Clin Nutr 2014;99:302–11.<br>46. Harrison CL, Lombard CB, Teede HJ. Limiting postpartum weight retention through early antenatal intervention: the HeLP-her randomised controlled trial. Int J Behav Nutr Phys Act 2014;11:134.<br>47. Ferrara A, Hedderson MM, Brown SD, et al. The comparative effectiveness of diabetes prevention strategies to reduce postpartum weight retention in women with gestational diabetes mellitus: the Gestational Diabetes' Effects on Moms (GEM) cluster randomized controlled trial. Diabetes Care 2016;39:65–74.<br>48. Ruchat SM, Davenport MH, Giroux I, et al. Nutrition and exercise reduce excessive weight gain in normal-weight pregnant women. Med Sci Sports Exerc 2012;44:1419–26.<br>49. Vinter CA, Jensen DM, Ovesen P, et al. Postpartum weight retention and breastfeeding among obese women from the randomized controlled Lifestyle in Pregnancy (LiP) trial. Acta Obstet Gynecol Scand 2014;93:794–801.<br>50. Rauh K, Günther J, Kunath J, et al. Lifestyle intervention to prevent excessive maternal weight gain: mother and infant follow-up at 12 months postpartum. BMC Pregnancy Childbirth 2015;15:265.<br>51. Clapp JF, Kim H, Burciu B, et al. Beginning regular exercise in early pregnancy: effect on fetoplacental growth. Am J Obstet Gynecol 2000;183:1484–8.<br>52. Barakat R, Ruiz JR, Stirling JR, et al. Type of delivery is not affected by light resistance and toning exercise training during pregnancy: a randomized controlled trial. Am J Obstet Gynecol 2009;201:590.e1–590.e6.<br>53. Cavalcante SR, Cecatti JG, Pereira RI, et al. Water aerobics II: maternal body composition and perinatal outcomes after a program for low risk pregnant women. Reprod Health 2009;6:1.<br>54. Garshasbi A, Faghih Zadeh S. The effect of exercise on the intensity of low back pain in pregnant women. Int J Gynaecol Obstet 2005;88:271–5.<br>55. Sedaghati P, Ziaee V, Ardjmand A. The effect of an ergometric training program on pregnants weight gain and low back pain. Gazzetta Medica Italiana Archivio Per Le Scienze Mediche 2007:166.<br>56. Barakat R, Cordero Y, Coteron J, et al. Exercise during pregnancy improves maternal glucose screen at 24-28 weeks: a randomised controlled trial. Br J Sports Med 2012;46:656–61.<br>57. Barakat R, Pelaez M, Montejo R, et al. Exercise during pregnancy improves maternal health perception: a randomized controlled trial. Am J Obstet Gynecol 2011;204:402.e1–402.e7.<br>58. Price BB, Amini SB, Kappeler K. Exercise in pregnancy: effect on fitness and obstetric outcomes-a randomized trial. Med Sci Sports Exerc 2012;44:2263–9.<br>59. Cordero Y, Peláez M, De Miguel M, et al. Can moderate physical exercise during pregnancy act as a factor in preventing gestational diabetes? Revista Internacional de Ciencias del Deporte 2012;27:3–19.<br>60. Barakat R, Pelaez M, Lopez C, et al. Exercise during pregnancy and gestational diabetes-related adverse effects: a randomised controlled trial. Br J Sports Med 2013;47:630–6.<br>61. Ghodsi Z, Asltoghiri M. Effects of aerobic exercise training on maternal and neonatal outcome: a randomized controlled trial on pregnant women in Iran. J Pak Med Assoc 2014;64:1053.<br>62. Ko CW, Napolitano PG, Lee SP, et al. Physical activity, maternal metabolic measures, and the incidence of gallbladder sludge or stones during pregnancy: a randomized trial. Am J Perinatol 2014;31:39–48.<br>63. Marquez-Sterling S, Perry AC, Kaplan TA, et al. Physical and psychological changes with vigorous exercise in sedentary primigravidae. Med Sci Sports Exerc 2000;32:58–62.<br>64. Ong MJ, Guelfi KJ, Hunter T, et al. Supervised home-based exercise may attenuate the decline of glucose tolerance in obese pregnant women. Diabetes Metab 2009;35:418–21.<br>65. Kihlstrand M, Stenman B, Nilsson S, et al. Water-gymnastics reduced the intensity of back/low back pain in pregnant women. Acta Obstet Gynecol Scand 1999;78:180–5.<br>66. Ussher M, Lewis S, Aveyard P, et al. Physical activity for smoking cessation in pregnancy: randomised controlled trial. BMJ 2015;350:h2145.<br>67. Taniguchi C, Sato C. Home-based walking during pregnancy affects mood and birth outcomes among sedentary women: a randomized controlled trial. Int J Nurs Pract 2016;22:420–6.<br>68. Labonte-Lemoyne E, Curnier D, Ellemberg D. Exercise during pregnancy enhances cerebral maturation in the newborn: a randomized controlled trial. J Clin Exp Neuropsychol 2017;39:347–54.
Syngelaki *et al.* 2019 [57]	1. Rae A, Bond D, Evans S, et al. A randomised controlled trial of dietary energy restriction in the management of obese women with gestational diabetes. Aust N Z J Obstet Gynaecol. 2000;40(4):416–422.<br>2. Polley BA, Wing RR, Sims CJ. Randomized controlled trial to prevent excessive weight gain in pregnant women. Int J Obes Relat Metab Disord. 2002;26(11): 1494–1502.<br>3. Wolff S, Legarth J, Vangsgaard K, et al. A randomized trial of the effects of dietary counseling on gestational weight gain and glucose metabolism in obese pregnant women. Int J Obes. 2008;32(3):495–501.<br>4. Thornton YS, Smarkola C, Kopacz SM, et al. Perinatal outcomes in nutritionally monitored obese pregnant women: a randomized clinical trial. J Natl Med Assoc. 2009;101(6):569–577.<br>5. Guelinckx I, Devlieger R, Mullie P, et al. Effect of lifestyle intervention on dietary habits, physical activity, and gestational weight gain in obese pregnant women: a randomized controlled trial. Am J Clin Nutr. 2010;91(2):373–380.<br>6. Phelan S, Phipps MG, Abrams B, et al. Randomized trial of a behavioral intervention to prevent excessive gestational weight gain: the Fit for Delivery Study. AMJ Clin Nutr. 2011;93(4):772–779.<br>7. Bogaerts AF, Devlieger R, Nuyts E, et al. Effects of lifestyle intervention in obese pregnant women on gestational weight gain and mental health: a randomized controlled trial. Int J Obes (Lond). 2013;37(6):814–821.

Review	References of included behavioural interventions delivered in pregnancy
	8. Dodd JM, Newman A, Moran LJ, et al. The effect of antenatal dietary and lifestyle advice for women who are overweight or obese on emotional well-being: the LIMIT randomized trial. Acta Obstet Gynecol Scand. 2016;95(3):309–318.<br>9. Petrella E, Malavolti M, Bertarini V, et al. Gestational weight gain in overweight and obese women enrolledin a healthy lifestyle and eating habits program. J Matern Fetal Neonatal Med. 2014;27(13):1348–1352.<br>10. Renault KM, Nørgaard K, Nilas L, et al. The Treatment of Obese Pregnant Women (TOP) study: a randomized controlled trial of the effect of physical activity intervention assessed by pedometer with or without dietary intervention in obese pregnant women. Am J Obstet Gynecol. 2014;210(2):134.e1–134.e9.<br>11. Vesco KK, Karanja N, King JC, et al. Efficacy of a group-based dietary intervention for limiting gestational weight gain among obese women: a randomized trial. Obesity (Silver Spring). 2014;22(9): 1989–1996.<br>12. Poston L, Bell R, Croker H, et al. Effect of a behavioural intervention in obese pregnant women (the UPBEAT study): a multicentre, randomised controlled trial. Lancet Diabetes Endocrinol. 2015;3(10):767–777.<br>13. McCarthy EA, Walker SP, Ugoni A, et al. Selfweighing and simple dietary advice for overweight and obese pregnant women to reduce obstetric complicationswithout impact on quality of life: a randomised controlled trial. BJOG. 2016;123(6):965–973.<br>14. Bruno R, Petrella E, Bertarini V, et al. Adherence to a lifestyle programme in overweight/obese pregnant women and effect on gestational diabetes mellitus: a randomized controlled trial. Matern Child Nutr. 2017;13(3). doi:10.1111/mcn.12333<br>15. Peaceman AM, Kwasny MJ, Gernhofer N, et al. MOMFIT: A randomized clinical trial of an intervention to prevent excess gestational weight gain in overweight and obese women. Am J Obstet Gynecol. 2017;216(1):S2–SS3.<br>16. Peccei A, Blake-Lamb T, Rahilly D, et al. Intensive prenatal nutrition counseling in a community health setting: A randomized controlled trial. Obstet Gynecol. 2017;130(2):423–432.<br>17. Ruiz JR, Perales M, Pelaez M, et al. Supervised exercise- based intervention to prevent excessive gestational weight gain: a randomized controlled trial. Mayo Clin Proc. 2013;88(12):1388–1397.<br>18. Bisson M, Almeras N, Dufresne SS, et al. A 12-week exercise program for pregnant women with obesity to improve physical activity levels: an open randomised Preliminary Study. PLoS One. 2015;10(9):e0137742.<br>19. Seneviratne SN, Jiang Y, Derraik J, et al. Effects of antenatal exercise in overweight and obese pregnant women on maternal and perinatal outcomes: a randomised controlled trial. BJOG. 2016;123(4):588–597.<br>20. Garnaes KK, Mørkved S, Salvesen Ø, et al. Exercise training and weight gain in obese pregnant women: a randomized controlled trial (ETIP trial). PLOS Med. 2016;13(7):e1002079.<br>21. Barakat R, Pelaez M, Cordero Y, et al. Exercise during pregnancy protects against hypertension and macrosomia: randomized clinical trial. Am J Obstet Gynecol 2016;214(5):649.e1–649.e8.<br>22. Wang C, Wei Y, Zhang X, et al. A randomized clinical trial of exercise during pregnancy to prevent gestational diabetes mellitus and improve pregnancy outcome in overweight and obese pregnant women. Am J Obstet Gynecol. 2017;216(4):340–351.<br>23. Vinter CA, Jensen DM, Ovesen P, et al. The LiP (Lifestyle in Pregnancy) study: a randomized controlled trial of lifestyle intervention in 360 obese pregnant women. Diabetes Care. 2011;34(12):2502–2507.<br>24. Wilson, S. M., et al. (2018). "Contingency Management Versus Psychotherapy for Prenatal Smoking Cessation: A Meta-Analysis of Randomized Controlled Trials." Women's Health Issues: Official Publication Of The Jacobs Institute Of Women's Health 28(6): 514-523.
Wang *et al.* 2019 [58]	1. Wang C, Wei Y, Zhang X, et al. A randomized clinical trial of exercise during pregnancy to prevent gestational diabetes mellitus and improve pregnancy outcome in overweight and obese pregnant women. Am J Obstet Gynecol 2017;216:340–51.<br>2. Da Silva S, Hallal P, Domingues M, et al. A randomized controlled trial of exercise during pregnancy on maternal and neonatal outcomes: results from the PAMELA study. Int J Behav Nutr Phys Act 2017;14:175.<br>3. Perales M, Calabria I, Lopez C, et al. Regular exercise throughout pregnancy is associated with a shorter first stage of labor. Am J Health Promot 2016;30:149–54.<br>4. Barakat R, Cordero Y, Coteron J, et al. Exercise during pregnancy improves maternal glucose screen at 24–28 weeks: a randomised controlled trial. Br J Sports Med 2012;46:656–61.<br>5. Barakat R, Perales M, Bacchi M, et al. A program of exercise throughout pregnancy. Is it safe to mother and newborn? Am J Health Promot 2014;29:2–8.<br>6. Barakat R, Pelaez M, Montejo R, et al. Exercise throughout pregnancy does not cause preterm delivery: a randomized, controlled trial. J Phys Act Health 2014;11:1012–7<br>7. Ruiz J, Perales M, Pelaez M, et al. Supervised exercise-based intervention to prevent excessive gestational weight gain: a randomized controlled trial. Mayo Clin Proc 2013;88:1388–97.<br>8. Barakat R, Pelaez M, Lopez C, et al. Exercise during pregnancy and gestational diabetes-related adverse effects: a randomised controlled trial. Br J Sports Med 2013;47:630–6.<br>9. Price B, Amini S, Kappeler K. Exercise in pregnancy: effect on fitness and obstetric outcomes-a randomized trial. Med Sci Sports Exerc 2012;44:2263–9.<br>10. Rodríguez YC, Puente MP, Abad MDM, RBC, et al. Can moderate physical exercise during pregnancy act as a factor in preventing Gestational Diabetes? Rev Int Cienc Deporte 2012;8:3–19.<br>11. Barakat R, Pelaez M, Montejo R, et al. Exercise during pregnancy improves maternal health perception: a randomized controlled trial. Am J Obstet Gynecol 2011;204:402e1-7.<br>12. Haakstad L, Bø K. Exercise in pregnant women and birth weight: a randomized controlled trial. BMC Pregnancy Childbirth 2011;11:66.<br>13. Hopkins S, Baldi J, Cutfield W, et al. Effects of exercise training on maternal hormonal changes in pregnancy. Clin Endocrinol (Oxf) 2011;74:495–500.<br>14. Ramírez-Vélez R, Aguilar de Plata AC, Mosquera-Escudero M, BS, et al. The effect of aerobic exercise on oxygen consumption in healthy first pregnancy females: a randomized clinical trial. Rev Colomb Obstet Ginecol 2011;62:15–23.<br>15. Cavalcante S, Cecatti J, Pereira R, et al. Water aerobics II: maternal body composition and perinatal outcomes after a program for low risk pregnant women. Reprod Health 2009;6:1.<br>16. Barakat R, Lucia A, Ruiz J. Resistance exercise training during pregnancy and newborn's birth size: a randomised controlled trial. Int J Obes V 33 20052009;1048–57.<br>17. Sedaghati P, Ziaee V, Ardjmand AA. The effect of an ergometric training program on pregnants' weight gain and low back pain. Gazz Med Ital 2007;166:209–13.<br>18. Garshasbi A, Faghih Zadeh S. The effect of exercise on the intensity of low back pain in pregnant women. Int J Gynaecol Obstet 2005;88:271–5.<br>19. Kihlstrand M, Stenman B, Nilsson S, et al. Water-gymnastics reduced the intensity of back/low back pain in pregnant women. Acta Obstet GynecolScand 1999;78:180–5.

Review	References of included behavioural interventions delivered in pregnancy
	20. Taniguchi et al.,2016<br>21. Polley BA, Wing RR, Sims CJ (2002). Randomized controlled trial to prevent excessive weight gain in pregnant women. Int J Obes Relat Metab Disord; 26(11): 1494–1502.
Poyatos-Leon *et al.* 2015 [59]	1. Cavalcante SR, Cecatti JG, Pereira RI, Baciuk EP, Bernardo AL, Silveira C. Water aerobics II: maternal body composition and perinatal outcomes after a program for low risk pregnant women. Reprod Health. 2009; 6: 1.<br>2. Barakat R, Perales M, Bacchi M, Coteron J, Refoyo I. A program of exercise throughout pregnancy. Is it safe to mother and newborn? Am J Health Promot. 2013; 29: 2– 8.<br>3. Stafne SN, Salvesen KA, Romundstad PR, Eggebo TM, Carlsen SM, Morkved S. Regular exercise during pregnancy to prevent gestational diabetes: a randomized controlled trial. Obstet Gynecol. 2012; 119: 29– 36.<br>4. Barakat R, Pelaez M, López C, Montejo R, Coteron J. Exercise during pregnancy reduces the rate of cesarean and instrumental deliveries: results of a randomized controlled trial. J Matern Fetal Neonatal Med. 2012; 25: 2372– 6.<br>5. Price BB, Amini SB, Kappeler K. Exercise in pregnancy: effect on fitness and obstetric outcomes a randomized trial. Med Sci Sports Exerc. 2012; 44: 2263– 9.<br>6. Barakat R, Pelaez M, Montejo R, Luaces M, Zakynthinaki M. Exercise during pregnancy improves maternal health perception: a randomized controlled trial. Am J Obstet Gynecol. 2011; 204: 402.e1– 7.<br>7. Barakat R, Ruiz JR, Stirling JR, Zakynthinaki M, Lucia A. Type of delivery is not affected by light resistance and toning exercise training during pregnancy: a randomized controlled trial. Am J Obstet Gynecol. 2009; 201: 590.e1– 6.
Walker *et al.* 2018 [60]	1. Barakat R, Cordero Y, Coteron J, Luaces M, Montejo R. Exercise during pregnancy improves maternal glucose screen at 24–28 weeks: a randomised controlled trial. Br J Sports Med. 2012; 46: 656– 61<br>2. Haby K., Berg M., Gyllensten H., Hanas R., Premberg A. Mighty Mums—A lifestyle intervention at primary care level reduces gestational weight gain in women with obesity. BMC Obes. 2018;5:16. doi: 10.1186/s40608-018-0194-4.<br>3. Syngelaki A., Nicolaides K., Balani J., Hyer S., Akolekar R., Kotecha R., Pastides A., Shehata H. Metformin versus Placebo in Obese Pregnant Women without Diabetes Mellitus. N. Engl. J. Med. 2016;374:434–443. doi: 10.1056/NEJMoa1509819.<br>4. Brownfoot F., Davey M., Kornman L., Brownfoot F. Routine weighing to reduce excessive antenatal weight gain: A randomised controlled trial. BJOG. 2016;123:254–261. doi: 10.1111/1471-0528.13735.<br>5. Clapp J., Kim H., Burciu B., Schmidt S., Petry K., Lopez B. Continuing regular exercise during pregnancy: Effect of exercise volume on fetoplacental growth. Am. J. Obstet. Gynecol. 2002;186:142–147. doi: 10.1067/mob.2002.119109.<br>6. Nobles C., Marcus B., Stanek E., Braun B., Whitcomb B., Manson J., et al. The Effect of an Exercise Intervention on Gestational Weight Gain: The Behaviors Affecting Baby and You (B.A.B.Y.) Study: A Randomized Controlled Trial. Am. J. Health Promot. 2018;32:736–744. doi: 10.1177/0890117117732409.<br>7. Markovic T., Muirhead R., Overs S., Ross G., Louie J., Kizirian N., et al. Randomized Controlled Trial Investigating the Effects of a Low-Glycemic Index Diet on Pregnancy Outcomes in Women at High Risk of Gestational Diabetes Mellitus: The GI Baby 3 Study. Diabetes Care. 2016;39:31–38. doi: 10.2337/dc15-0572.<br>8. Peccei A., Blake-Lamb T., Rahilly D., Hatoum I., Bryant A. Intensive prenatal nutrition counseling in a community health setting: A randomized controlled trial. Obstet. Gynecol. 2017;130:423–432. doi: 10.1097/AOG.0000000000002134.<br>9. Rhodes E., Pawlak D., Takoudes T., Ebbeling C., Feldman H., Lovesky M., Cooke E.A., Leidig M.M., Ludwig D.S. Effects of a low-glycemic load diet in overweight and obese pregnant women: A pilot randomized controlled trial. Am. J. Clin. Nutr. 2010;92:1306–1315. doi: 10.3945/ajcn.2010.30130.<br>10. Cahill A., Haire-Joshu D., Cade W., Stein R., Woolfolk C., Moley K., Mathur A., Schechtman K., Klein S. Weight control program and gestational weight gain in disadvantaged women with overweight or obesity: A randomized clinical trial. Obesity. 2018;26:485–491. doi: 10.1002/oby.22070.<br>11. Pawalia A., Kulandaivelan S., Savant S., Yadav V. Exercise in pregnancy: Effect on obesity parameters in indian women—A randomized controlled trial. Rom. J. Diabetes Nutr. Metab. Dis. 2017;24:315–323. doi: 10.1515/rjdnmd-2017-0037.<br>12. Phelan S., Wing R., Brannen A., McHugh A., Hagobian T., Schaffner A., Jelalian E., Hart C.N., Scholl T.O., Munoz-Christian K., et al. Randomized controlled clinical trial of behavioral lifestyle intervention with partial meal replacement to reduce excessive gestational weight gain. Am. J. Clin. Nutr. 2018;107:183–194. doi: 10.1093/ajcn/nqx043. [PMC free article]<br>13. Simmons D., Jelsma J., Galjaard S., Devlieger R., van Assche A., Jans G., Corcoy R., Adelantado J.M., Dunne F., Desoye G., et al. Results from a European multicenter randomized trial of physical activity and/or healthy eating to reduce the risk of gestational diabetes mellitus: The DALI lifestyle pilot. Diabetes Care. 2015;38:1650–1656. doi: 10.2337/dc15-0360.<br>14. Thomson J., Tussing-Humphreys L., Goodman M., Olender S. Gestational weight gain: Results from the delta healthy sprouts comparative impact trial. J. Pregnancy. 2016;2016:12. doi: 10.1155/2016/5703607.<br>15. Moses R., Casey S., Quinn E., Cleary J., Tapsell L., Milosavljevic M., Petocz P., Brand-Miller J.C. Pregnancy and Glycemic Index Outcomes study: Effects of low glycemic index compared with conventional dietary advice on selected pregnancy outcomes. Am. J. Clin. Nutr. 2014;99:517–523. doi: 10.3945/ajcn.113.074138.<br>16. Perales M., Cordero Y., Vargas M., Lucia A., Barakat R. Exercise and depression in overweight and obese pregnant women: A randomised controlled trial. Arch. Med. Deporte. 2015;32:156–163.<br>17. Abdel-Aziz S., Hegazy I., Mohamed D., Abu El Kasem M., Hagag S. Effect of dietary counseling on preventing excessive weight gain during pregnancy. Public Health. 2018;154:172–181. doi: 10.1016/j.puhe.2017.10.014.<br>18. Vítolo M., Bueno M., Gama C. Impact of a dietary counseling program on the gain weight speed of pregnant women attended in a primary care service. Rev. Bras. Ginecol. Obstet. 2011;33:58–59. doi: 10.1590/S0100-72032011000100009.

Review	References of included behavioural interventions delivered in pregnancy
	19. Renault K., Nørgaard K., Nilas L., Carlsen E., Cortes D., Pryds O., Secher N.J. The Treatment of Obese Pregnant Women (TOP) study: A randomized controlled trial of the effect of physical activity intervention assessed by pedometer with or without dietary intervention in obese pregnant women. Am. J. Obstet. Gynecol. 2014;210:134. doi: 10.1016/j.ajog.2013.09.029.<br>20. Wang S., Ma J., Yang H. Lifestyle intervention for gestational diabetes mellitus prevention: A cluster-randomized controlled study. Chronic Dis. Transl. Med. 2015;1:169–174. doi: 10.1016/j.cdtm.2015.09.001<br>21. Bosaeus M., Hussain A., Karlsson T., Andersson L., Hulthen L., Svelander C., Sandberg A.S., Larsson I., Ellegard L., Holmang A. A randomized longitudinal dietary intervention study during pregnancy: Effects on fish intake, phospholipids, and body composition. Nutrition. 2015;14:1. doi: 10.1186/1475-2891-14-1.<br>22. Vinter C., Jensen D., Ovesen P., Beck-Nielsen H.J.O. Lifestyle and pregnancy (LIP) study: The clinical effect of lifestyle intervention during pregnancy in obese women. Diabetes. 2011;60:348–349. doi: 10.2337/dc11-1150.<br>23. Korpi-Hyovalti E., Schwab U., Laaksonen D.E., Linjama H., Heinonen S., Niskanen L. Effect of intensive counselling on the quality of dietary fats in pregnant women at high risk of gestational diabetes mellitus. Br. J. Nutr. 2012;108:910–917. doi: 10.1017/S0007114511006118.<br>24. McCarthy E., Walker S., Ugoni A., Lappas M., Leong O., Shub A. Self-weighing and simple dietary advice for overweight and obese pregnant women to reduce obstetric complications without impact on quality of life: A randomised controlled trial. BJOG. 2016;123:965–973. doi: 10.1111/1471-0528.13919.<br>25. Walsh J., McGowan C., Mahony R., Foley M., McAuliffe F. Low glycaemic index diet in pregnancy to prevent macrosomia (ROLO study): Randomised control trial. BMJ. 2012;345:e5605. doi: 10.1136/bmj.e5605.<br>26. Thornton Y., Smarkola C., Kopacz S., Ishoof S. Perinatal outcomes in nutritionally monitored obese pregnant women: A randomized clinical trial. J. Natl. Med. Assoc. 2009;101:569–577. doi: 10.1016/S0027-9684(15)30942-1.<br>27. Deveer R., Deveer M., Akbaba E., Engin-Üstün Y., Aydoğan P., Çelikkaya H., Danisman N., Mollamahmutoglu L. The effect of diet on pregnancy outcomes among pregnants with abnormal glucose challenge test. Eur. Rev. Med. Pharmacol. Sci. 2013;17:1258–1261.<br>28. Di Carlo C., Iannotti G., Sparice S., Chiacchio M., Greco E., Tommaselli G., Nappi C. The role of a personalized dietary intervention in managing gestational weight gain: A prospective, controlled study in a low-risk antenatal population. Arch. Gynecol. Obstet. 2014;289:765–770. doi: 10.1007/s00404-013-3054-y.<br>29. Bonomo M., Corica D., Mion E., Goncalves D., Motta G., Merati R., Ragusa A., Morabito A. Evaluating the therapeutic approach in pregnancies complicated by borderline glucose intolerance: A randomized clinical trial. Diabet. Med. 2005;22:1536–1541. doi: 10.1111/j.1464-5491.2005.01690.x.<br>30. Wolff S., Legarth J., Vangsgaard K., Toubro S., Astrup A. A randomized trial of the effects of dietary counseling on gestational weight gain and glucose metabolism in obese pregnant women. Int. J. Obes. 2008;32:495–501. doi: 10.1038/sj.ijo.0803710.<br>31. Ilmonen J., Isolauri E., Poussa T., Laitinen K. Impact of dietary counselling and probiotic intervention on maternal anthropometric measurements during and after pregnancy: A randomized placebo-controlled trial. Clin. Nutr. 2011;30:156–164. doi: 10.1016/j.clnu.2010.09.009.<br>32. Barakat R., Cordero Y., Coteron J., Luaces M., Montejo R. Exercise during pregnancy improves maternal glucose screen at 24–28 weeks: A randomised controlled trial. Br. J. Sports Med. 2012;46:656–661. doi: 10.1136/bjsports-2011-090009.<br>33. Barakat R., Lucia A., Ruiz J. Resistance exercise training during pregnancy and newborn's birth size: A randomised controlled trial. Int. J. Obes. 2009;33:9. doi: 10.1038/ijo.2009.150.<br>34. Barakat R., Pelaez M., Cordero Y., Perales M., Lopez C., Coteron J., Mottola M.F. Exercise during pregnancy protects against hypertension and macrosomia: Randomized clinical trial. Am. J. Obstet. Gynecol. 2016;214:649-e1. doi: 10.1016/j.ajog.2015.11.039.<br>35. Barakat R., Pelaez M., Lopez C., Lucia A., Ruiz J.R. Exercise during pregnancy and gestational diabetes-related adverse effects: A randomised controlled trial. Br. J. Sports Med. 2013;47:630–636. doi: 10.1136/bjsports-2012-091788.<br>36. Barakat R., Pelaez M., Montejo R., Luaces M., Zakynthinaki M. Exercise during pregnancy improves maternal health perception: A randomized controlled trial. Am. J. Obstet. Gynecol. 2011;204:402. doi: 10.1016/j.ajog.2011.01.043<br>37. Barakat R., Pelaez M., Montejo R., Refoyo I., Coteron J. Exercise throughout pregnancy does not cause preterm delivery: A randomized, controlled trial. J. Phys. Act. Health. 2014;11:1012–1017. doi: 10.1123/jpah.2012-0344.<br>38. Ruiz J., Perales M., Pelaez M., Lopez C., Lucia A., Barakat R. Supervised exercise-based intervention to prevent excessive gestational weight gain: A randomized controlled trial. Mayo Clin. Proc. 2013;88:1388–1397. doi: 10.1016/j.mayocp.2013.07.020.<br>39. Clapp J., Kim H., Burciu B., Lopez B. Beginning regular exercise in early pregnancy: Effect on fetoplacental growth. Am. J. Obstet. Gynecol. 2000;183:1484–1488. doi: 10.1067/mob.2000.107096.<br>40. Garshasbi A., Faghih Zadeh S. The effect of exercise on the intensity of low back pain in pregnant women. Int. J. Gynecol. Obstet. 2005;88:271–275. doi: 10.1016/j.ijgo.2004.12.001.<br>41. Cavalcante S., Cecatti J., Pereira R., Baciuk E., Bernardo A., Silveira C. Water aerobics II: Maternal body composition and perinatal outcomes after a program for low risk pregnant women. Reprod. Health. 2009;6:1. doi: 10.1186/1742-4755-6-1.<br>42. Haakstad L., Bø K. Effect of regular exercise on prevention of excessive weight gain in pregnancy: A randomised controlled trial. Eur. J. Contracept. Reprod. Health Care. 2011;16:116–125. doi: 10.3109/13625187.2011.560307.<br>43. Nascimento S., Surita F., Parpinelli M., Siani S., Pinto e Silva J. The effect of an antenatal physical exercise programme on maternal/perinatal outcomes and quality of life in overweight and obese pregnant women: A randomised clinical trial. BJOG. 2011;118:1455–1463. doi: 10.1111/j.1471-0528.2011.03084.x.

Review	References of included behavioural interventions delivered in pregnancy
	44. Kong K., Campbell C., Foster R., Peterson A., Lanningham-Foster L. A pilot walking program promotes moderate-intensity physical activity during pregnancy. Med. Sci. Sports Exerc. 2014;46:462–471. doi: 10.1249/MSS.0000000000000141.<br>45. Bisson M., Almeras N., Dufresne S., Robitaille J., Rheaume C., Bujold E., Frenette J., Tremblay A., Marc I. A 12-week exercise program for pregnant women with obesity to improve physical activity levels: An open randomised preliminary study. PLoS ONE. 2015;10:e0137742. doi: 10.1371/journal.pone.0137742.<br>46. Ronnberg A., Ostlund I., Fadl H., Gottvall T., Nilsson K. Intervention during pregnancy to reduce excessive gestational weight gain-a randomised controlled trial. BJOG. 2015;122:537–544. doi: 10.1111/1471-0528.13131.<br>47. Dekker Nitert M., Barrett H., Denny K., McIntyre H., Callaway L. Exercise in pregnancy does not alter gestational weight gain, MCP-1 or leptin in obese women. Aust. N. Z. J. Obstet. Gynaecol. 2015;55:27–33. doi: 10.1111/ajo.12300.<br>48. Oostdam N., Van Poppel M., Wouters M., Eekhoff E., Bekedam D., Kuchenbecker W., Quartero H., Heres M., van Mechelen W. No effect of the FitFor2 exercise programme on blood glucose, insulin sensitivity, and birthweight in pregnant women who were overweight and at risk for gestational diabetes: Results of a randomised controlled trial. BJOG. 2012;119:1098–1107. doi: 10.1111/j.1471-0528.2012.03366.x.<br>49. Bacchi M., Mottola M., Perales M., Refoyo I., Barakat R. Aquatic activities during pregnancy prevent excessive maternal weight gain and preserve birth weight: A randomized clinical trial. Am. J. Health Promot. 2018;32:729–735. doi: 10.1177/0890117117697520.<br>50. Barakat R., Franco E., Perales M., Lopez C., Mottola M. Exercise during pregnancy is associated with shorter duration of labor: A randomized clinical trial. Eur. J. Obstet. Gynecol. Reprod. Biol. 2018;224:33–40. doi: 10.1016/j.ejogrb.2018.03.009<br>51. da Silva S., Hallal P., Domingues M., Bertoldi A., Silveira M., Bassani D., da Silva I.C., da Sliva B.C., Coll C.D., Evenson K. A randomized controlled trial of exercise during pregnancy on maternal and neonatal outcomes: Results from the PAMELA study. Int. J. Behav. Nutr. Phys. Act. 2017;14 doi: 10.1186/s12966-017-0632-6.<br>52. Daly N., Farren M., McKeating A., O'Kelly R., Stapleton M., Turner M., et al. A medically supervised pregnancy exercise intervention in obese women: A randomized controlled trial. Obstet. Gynecol. 2017;130:1001–1010. doi: 10.1097/AOG.0000000000002267.<br>53. Garnæs K., Mørkved S., Salvesen Ø., Moholdt T. Exercise training and weight gain in obese pregnant women: A randomized controlled trial (ETIP Trial) PLoS Med. 2016;13:1–18. doi: 10.1371/journal.pmed.1002079.<br>54. Wang C., Wei Y., Zhang X., Zhang Y., Xu Q., Sun Y., Su S., Zhang L., Liu C., Feng Y., et al. A randomized clinical trial of exercise during pregnancy to prevent gestational diabetes mellitus and improve pregnancy outcome in overweight and obese pregnant women. Am. J. Obstet. Gynecol. 2017;216:340–351. doi: 10.1016/j.ajog.2017.01.037.<br>55. Rodríguez-Blanque R., Carlos Sánchez-García J., Manuel Sánchez-López A., Mur-Villar N., Fernández-Castillo R., José Aguilar-Cordero M. Influence of physical exercise during pregnancy on birthweight: A randomized clinical trial. Nutr. Hosp. 2017;34:834–840. doi: 10.20960/nh.1095.<br>56. Petrella E., Malavolti M., Bertarini V., Pignatti L., Neri I., Battistini N.C., Facchinetti F. Gestational weight gain in overweight and obese women enrolled in a healthy lifestyle and eating habits program. J. Matern. Neonatal Med. 2014;27:1348–1352. doi: 10.3109/14767058.2013.858318.<br>57. Phelan S., Phipps M., Abrams B., Darroch F., Schaffner A., Wing R. Randomized trial of a behavioral intervention to prevent excessive gestational weight gain: The Fit for Delivery Study. Am. J. Clin. Nutr. 2011;93:772–779. doi: 10.3945/ajcn.110.005306.<br>58. Polley B., Wing R., Sims C. Randomized controlled trial to prevent excessive weight gain in pregnant women. Int. J. Obes. 2002;26:1494–1502. doi: 10.1038/sj.ijo.0802130.<br>59. Poston L., Bell R., Croker H., Flynn A., Godfrey K., Goff L., Hayes L., Khazaezadeh N., Nelson S.M., Oteng-Ntim E., et al. Effect of a behavioural intervention in obese pregnant women (the UPBEAT study): A multicentre, randomised controlled trial. Lancet Diabetes Endocrinol. 2015;3:767–777. doi: 10.1016/S2213-8587(15)00227-2.<br>60. Asbee S., Jenkins T., Butler J., White J., Elliot M., Rutledge A. Preventing excessive weight gain during pregnancy through dietary and lifestyle counseling: A randomized controlled trial. Obstet. Gynecol. 2009;113:305–312. doi: 10.1097/AOG.0b013e318195baef.<br>61. Aşcı Ö., Rathfisch G. Effect of lifestyle interventions of pregnant women on their dietary habits, lifestyle behaviors, and weight gain: A randomized controlled trial. J. Health Popul. Nutr. 2016;35:1–9. doi: 10.1186/s41043-016-0044-2.<br>62. Guelinckx I., Devlieger R., Mullie P., Vansant G. Effect of lifestyle intervention on dietary habits, physical activity, and gestational weight gain in obese pregnant women: A randomized controlled trial. Am. J. Clin. Nutr. 2010;91:373–380. doi: 10.3945/ajcn.2009.28166.<br>63. Huang T., Yeh C., Tsai Y. A diet and physical activity intervention for preventing weight retention among Taiwanese childbearing women: A randomised controlled trial. Midwifery. 2011;27:257–264. doi: 10.1016/j.midw.2009.06.009.<br>64. Hui A., Back L., Ludwig S., Gardiner P., Sevenhuysen G., Dean H., Sellers E., McGavock J., Morris M., Bruce S., et al. Lifestyle intervention on diet and exercise reduced excessive gestational weight gain in pregnant women under a randomized controlled trial. Obstet. Gynecol. Surv. 2012;67:263–264. doi: 10.1097/OGX.0b013e3182561861<br>65. Hui A., Back L., Ludwig S., Gardiner P., Sevenhuysen G., Dean H., Sellers E., McGavock J., Morris M., Jiang D., et al. Effects of lifestyle intervention on dietary intake, physical activity level, and gestational weight gain in pregnant women with different pre-pregnancy Body Mass Index in a randomized control trial. BMC Pregnancy Childbirth. 2014;14:331. doi: 10.1186/1471-2393-14-331.<br>66. Ruchat S., Davenport M., Giroux I., Hillier M., Batada A., Sopper M., Hammond J.M., Mottola M.F. Nutrition and exercise reduce excessive weight gain in normal-weight pregnant women. Med. Sci. Sports Exerc. 2012;44:1419–1426. doi: 10.1249/MSS.0b013e31825365f1.<br>67. Bogaerts A., Devlieger R., Nuyts E., Witters I., Gyselaers W., Van den Bergh B. Effects of lifestyle intervention in obese pregnant women on gestational weight gain and mental health: A randomized controlled trial. Int. J. Obes. 2013;37:814–821. doi: 10.1038/ijo.2012.162.

Review	References of included behavioural interventions delivered in pregnancy
	68. Harrison C., Lombard C., Strauss B., Teede H. Optimizing healthy gestational weight gain in women at high risk of gestational diabetes: A randomized controlled trial. Obesity. 2013;21:904–909. doi: 10.1002/oby.20163.<br>69. Hawkins M., Hosker M., Marcus B., Rosal M., Braun B., Stanek E., Markenson G., Chasan-Taber L. A pregnancy lifestyle intervention to prevent gestational diabetes risk factors in overweight Hispanic women: A feasibility randomized controlled trial. Diabet. Med. 2015;32:108–115. doi: 10.1111/dme.12601.<br>70. Rauh K., Gabriel E., Kerschbaum E., Schuster T., von Kries R., Amann-Gassner U., Hauner H. Safety and efficacy of a lifestyle intervention for pregnant women to prevent excessive maternal weight gain: A cluster-randomized controlled trial. BMC Pregnancy Childbirth. 2013;13:151. doi: 10.1186/1471-2393-13-151.<br>71. Gesell S., Katula J., Strickland C., Vitolins M. Feasibility and initial efficacy evaluation of a community-based cognitive-behavioral lifestyle intervention to prevent excessive weight gain during pregnancy in Latina women. Matern. Child Health J. 2015;19:1842–1852. doi: 10.1007/s10995-015-1698-x.<br>72. Skouteris H., McPhie S., Hill B., McCabe M., Milgrom J., Kent B., Bruce L., Herring S., Gale J., Mihalopoulos C., et al. Health coaching to prevent excessive gestational weight gain: A randomized-controlled trial. Br. J. Health Psychol. 2016;21:31–51. doi: 10.1111/bjhp.12154.<br>73. Sagedal L., Øverby N., Bere E., Torstveit M., Lohne-Seiler H., Småstuen M., Hillesund E.R., Henriksen T., Vistad I. Lifestyle intervention to limit gestational weight gain: The Norwegian Fit for Delivery randomised controlled trial. BJOG. 2016;124:97–109. doi: 10.1111/1471-0528.13862.<br>74. Althuizen E., van der Wijden C., van Mechelen W., Seidell J., van Poppel M. The effect of a counselling intervention on weight changes during and after pregnancy: A randomised trial. BJOG. 2013;120:92–99. doi: 10.1111/1471-0528.12014.<br>75. Dodd J., Turnbull D., McPhee A., Deussen A., Grivell R., Yelland L., Crowther C.A., Wittert G., Owens J.A., Robinson J.S. Antenatal lifestyle advice for women who are overweight or obese: LIMIT randomised trial. BMJ. 2014;348:1285. doi: 10.1136/bmj.g1285.<br>76. Vesco K., Karanja N., King J., Gillman M., Leo M., Perrin N., McEvoy C.T., Eckhardt C.L., Smith K.S., Stevens C.L. Efficacy of a group-based dietary intervention for limiting gestational weight gain among obese women: A randomized trial. Obesity. 2014;22:1989–1996. doi: 10.1002/oby.20831<br>77. Jing W., Huang Y., Liu X., Luo B., Yang Y., Liao S. The effect of a personalized intervention on weight gain and physical activity among pregnant women in China. Int. J. Gynaecol. Obstet. 2015;129:138–141. doi: 10.1016/j.ijgo.2014.11.014.<br>78. Luoto R., Kinnunen T., Aittasalo M., Ojala K., Mansikkamaki K., Toropainen E., Kolu P., Vasankari T. Prevention of gestational diabetes: Design of a cluster-randomized controlled trial and one-year follow-up. BMC Pregnancy Childbirth. 2010;10:39. doi: 10.1186/1471-2393-10-39.<br>79. Bruno R., Petrella E., Bertarini V., Pedrielli G., Neri I., Facchinetti F. Adherence to a lifestyle programme in overweight/obese pregnant women and effect on gestational diabetes mellitus: A randomized controlled trial. Matern. Child Nutr. 2017;13 doi: 10.1111/mcn.12333.<br>80. Herring Cruice J., Bennett G., Rose M., Davey A., Foster G. Preventing excessive gestational weight gain among African American women: A randomized clinical trial. Obesity. 2016;24:30–36. doi: 10.1002/oby.21240.<br>81. Willcox J., Wilkinson S., Lappas M., Ball K., Crawford D., McCarthy E., Fjeldsoe B., Whittaker R., Maddison R., Campbell K. A mobile health intervention promoting healthy gestational weight gain for women entering pregnancy at a high body mass index: The txt4two pilot randomised controlled trial. BJOG. 2017;124:1718–1728. doi: 10.1111/1471-0528.14552<br>82. Olsen C., Groth S., Graham M., Reschke J., Strawderman M., Fernandez D. The effectiveness of an online intervention in preventing excessive gestational weight gain: The e-moms roc randomized controlled trial. BMC Pregnancy Childbirth. 2018;18:148. doi: 10.1186/s12884-018-1767-4.<br>83. Smith K., Lanningham-Foster L., Welch A., Campbell C. Web-Based Behavioral Intervention Increases Maternal Exercise but Does Not Prevent Excessive Gestational Weight Gain in Previously Sedentary Women. J. Phys. Act. Health. 2016;13:587–593. doi: 10.1123/jpah.2015-0219.<br>84. Jackson R., Stotland N., Caughey A., Gerbert B. Improving diet and exercise in pregnancy with Video Doctor counseling: A randomized trial. Patient Educ. Couns. 2011;83:203–209. doi: 10.1016/j.pec.2010.05.019.<br>85. Daley A., Jolly K., Jebb S., Lewis A., Clifford S., Roalfe A., Kenyon S., Aveyard P. Feasibility and acceptability of regular weighing, setting weight gain limits and providing feedback by community midwives to prevent excess weight gain during pregnancy: Randomised controlled trial and qualitative study. BMC Obes. 2015;2:35. doi: 10.1186/s40608-015-0061-5.<br>86. Jeffries K., Shub A., Walker S., Hiscock R., Permezel M. Reducing excessive weight gain in pregnancy: A randomised controlled trial. Med. J. Aust. 2009;191:429–433.<br>87. Quinlivan J., Lam L., Fisher J. A randomised trial of a four-step multidisciplinary approach to the antenatal care of obese pregnant women. Aust. N. Z. J. Obstet. Gynaecol. 2011;51:141–146. doi: 10.1111/j.1479-828X.2010.01268.x.<br>88. Santamaria A., Di Benedetto A., Petrella E., Pintaudi B., Corrado F., D'Anna R., Neri I., Facchinetti F. Myo-inositol may prevent gestational diabetes onset in overweight women: A randomized, controlled trial. J. Matern. Fetal Neonatal Med. 2016;29:3234–3237. doi: 10.3109/14767058.2015.1121478.<br>89. Herrera-Perdigon J., Hopkins E., Marcalle M., Brooten D., Youngblut J., Lizardo M. Weight gain in high-risk pregnant women: Comparison by primary diagnosis and type of care. Clin. Excell. Nurse Pract. 2005;9:195–201.<br>90. Bechtel-Blackwell D. Computer-assisted self-interview and nutrition education in pregnant teens. Clin. Nurs. Res. 2002;11:450–462. doi: 10.1177/105477302237456.
Vincze *et al.* 2019 [61]	1. Pollak KI, Alexander SC, Bennett G, Lyna P, Coffman CJ, Bilheimer A, et al. Weight-related SMS texts promoting appropriate pregnancy weight gain: A pilot study. Patient Educ Couns 2014;97(2):256–60.<br>2. Shyam S, Arshad F, Abdul Ghani R, Wahab NA, Safii NS, Nisak MYB, et al. Low glycaemic index diets improve glucose tolerance and body weight in women with previous history of gestational diabetes: a six months randomized trial. Nutr J 2013;12:68-.

Review	References of included behavioural interventions delivered in pregnancy
	3. Asbee SM, Jenkins TR, Butler JR, White J, Elliot M, Rutledge A. Preventing excessive weight gain during pregnancy through dietary and lifestyle counseling: a randomized controlled trial. Obstet Gynecol 2009;113(2 Pt 1):305–12.<br>4. Bosaeus M, Hussain A, Karlsson T, Andersson L, Hulthen L, Svelander C, et al. A randomized longitudinal dietary intervention study during pregnancy: effects on fish intake, phospholipids, and body composition. Nutr J 2015;14:1.<br>5. Renault KM, Norgaard K, Nilas L, Carlsen EM, Cortes D, Pryds O, et al. The Treatment of Obese Pregnant Women (TOP) study: A randomized controlled trial of the effect of physical activity intervention assessed by pedometer with or without dietary intervention in obese pregnant women. Am Journal Obstet Gynecol 2014;210(2). 134.e1-.e9.<br>6. Vinter CA, Jensen DM, Ovesen P, Beck-Nielsen H, Jørgensen JS. The LiP (Lifestyle in Pregnancy) study: a randomized controlled trial of lifestyle intervention in 360 obese pregnant women. Diabetes Care 2011;34(12):2502–7.<br>7. Guelinckx I, Devlieger R, Mullie P, Vansant G. Effect of lifestyle intervention on dietary habits, physical activity, and gestational weight gain in obese pregnant women: a randomized controlled trial. Am J Clin Nutr 2010;91(2):373–80.<br>8. Hui AL, Back L, Ludwig S, Gardiner P, Sevenhuysen G, Dean HJ, et al. Effects of lifestyle intervention on dietary intake, physical activity level, and gestational weight gain in pregnant women with different pre-pregnancy Body Mass Index in a randomized control trial. BMC Pregnancy Childbirth 2014;14:331.<br>9. Phelan S, Phipps MG, Abrams B, Darroch F, Schaffner A, Wing RR. Randomized trial of a behavioral intervention to prevent excessive gestational weight gain: the Fit for Delivery Study. Am J Clin Nutr 2011;93(4):772–9.<br>10. Polley B, Wing R, Sims C. Randomized controlled trial to prevent excessive weight gain in pregnant women. Int J Obesity 2002;26(11):1494–502<br>11. Bertz F, Brekke HK, Ellega˚rd L, Rasmussen KM, Wennergren M, Winkvist A. Diet and exercise weight-loss trial in lactating overweight and obese women. Am J Clin Nutr 2012;96(4): 698–705.<br>12. Davenport MH, Girouox I, Sopper MM, Mottola MF. Postpartum Exercise Regardless of Intensity Improves Chronic Disease Risk Factors. Med Sci Sports Exerc 2011;43(6):951–8.<br>13. Harrison CL, Lombard CB, Strauss BJ, Teede HJ. Optimizing healthy gestational weight gain in women at high risk of gestational diabetes: a randomized controlled trial. Obesity 2013;21(5):904–9.<br>14. Carlo C, Iannotti G, Sparice S, Chiacchio MP, Greco E, Tommaselli GA, et al. The role of a personalized dietary intervention in managing gestational weight gain: A prospective, controlled study in a low-risk antenatal population. Arch Gynecol Obstet 2014;289(4):765–70.<br>15. Deveer R, Deveer M, Akbaba E, Engin-U¨stu¨ n Y, Aydo?an P, Celikkaya H, et al. The effect of diet on pregnancy outcomes among pregnant with abnormal glucose challenge test. Eur Rev Med Pharmacol Sci 2013;17(9):1258–61.<br>16. Dodd JM, Kannieappan LM, Grivell RM, Deussen AR, Moran LJ, Yelland LN, et al. Effects of an antenatal dietary intervention on maternal anthropometric measures in pregnant women with obesity. Obesity 2015;23(8):1555–62.<br>17. Dodd JM, Turnbull D, McPhee AJ, Deussen AR, Grivell RM, Yelland LN, et al. Antenatal lifestyle advice for women who are overweight or obese: LIMIT randomised trial. BMJ 2014;348:g1285.<br>18. Harrison CL, Lombard CB, Teede HJ. Limiting postpartum weight retention through early antenatal intervention: the HeLP-her randomised controlled trial. Int J Behav Nutr Phys Act 2014;11:134.<br>19. Herring SJ, Cruice JF, Bennett GG, Rose MZ, Davey A, Foster GD. Preventing excessive gestational weight gain among African American women: A randomized clinical trial. Obesity 2016;24(1):30–6.<br>20. Hui A, Back L, Ludwig S, Gardiner P, Sevenhuysen G, Dean H, et al. Lifestyle intervention on diet and exercise reduced excessive gestational weight gain in pregnant women under a randomised controlled trial. BJOG 2012;119(1): 70–7.<br>21. Kinnunen TI, Raitanen J, Aittasalo M, Luoto R. Preventing excessive gestational weight gain-a secondary analysis of a cluster-randomised controlled trial. Eur J Clin Nut 2012;66(12): 1344–50.<br>22. Petrella E, Malavolti M, Bertarini V, Pignatti L, Neri I, Battistini NC, et al. Gestational weight gain in overweight and obese women enrolled in a healthy lifestyle and eating habits program. J Matern Fetal Neonatal Med 2014;27(13): 1348–52.<br>23. Phelan S, Phipps MG, Abrams B, Darroch F, Grantham K, Schaffner A, et al. Does behavioral intervention in pregnancy reduce postpartum weight retention? Twelve-month outcomes of the Fit for Delivery randomized trial. Am J Clin Nut 2014;99(2):302–11.<br>24. Rauh K, Gabriel E, Kerschbaum E, Schuster T, Kries R, AmannGassner U, et al. Safety and efficacy of a lifestyle intervention for pregnant women to prevent excessive maternal weight gain: A cluster-randomized controlled trial. BMC Pregnancy Childbirth 2013;13:151.<br>25. Rauh K, Gunther J, Kunath J, Stecher L, Hauner H. Lifestyle intervention to prevent excessive maternal weight gain: mother and infant follow-up at 12 months postpartum. BMC Pregnancy Childbirth 2015;15:265.<br>26. Vesco KK, Karanja N, King JC, Gillman MW, Leo MC, Perrin N, et al. Efficacy of a group-based dietary intervention for limiting gestational weight gain among obese women: A randomized trial. Obesity 2014;22(9):1989–96.<br>27. Vinter CA, Jensen DM, Ovesen P, Beck-Nielsen H, Tanvig M, Lamont RF, et al. Postpartum weight retention and breastfeeding among obese women from the randomized controlled Lifestyle in Pregnancy (LiP) trial. Acta Obstet Gynecol Scand 2014;93(8):794–801.<br>28. Wolff S, Legarth J, Vangsgaard K, Toubro S, Astrup A. A randomized trial of the effects of dietary counseling on gestational weight gain and glucose metabolism in obese pregnant women. Int J Obesity 2008;32(3):495–501.<br>29. Gray-Donald K, Robinson E, Collier A, David K, Renaud L, Rodrigues S. Intervening to reduce weight gain in pregnancy and gestational diabetes mellitus in Cree communities: an evaluation. Can Med Assoc J 2000;163(10):1247–51.<br>30. Huang T-t, Yeh C-Y, Tsai Y-C. A diet and physical activity intervention for preventing weight retention among Taiwanese childbearing women: a randomised controlled trial. Midwifery 2011;27(2):257–64.

Review	References of included behavioural interventions delivered in pregnancy
	31. Ilmonen J, Isolauri E, Poussa T, Laitinen K. Impact of dietary counselling and probiotic intervention on maternal anthropometric measurements during and after pregnancy: A randomized placebo-controlled trial. Clinical Nutrition 2011;30(2):156–64.<br>32. Jaakkola J, Isolauri E, Poussa T, Laitinen K. Benefits of repeated individual dietary counselling in long-term weight control in women after delivery. Maternal & Child Nutrition 2015;11:1041–8.<br>33. Martin J, MacDonald-Wicks L, Hure A, Smith R, Collins CE. Reducing postpartum weight retention and improving breastfeeding outcomes in overweight women: A pilot randomised controlled trial. Nutrients 2015;7(3):1465–79<br>34. Wilkinson SA, Pligt P, Gibbons KS, McIntyre HD. Trial for Reducing Weight Retention in New Mums: a randomised controlled trial evaluating a low intensity, postpartum weight management programme. J Hum Nutr Diet 2015;28(Suppl 1):15–28.<br>35. Brekke HK, Bertz F, Rasmussen KM, Bosaeus I, Ellegard L, Winkvist A. Diet and exercise interventions among overweight and obese lactating women: Randomized trial of effects on cardiovascular risk factors. PloS One 2014;9(2):e88250.<br>36. Colleran HL, Lovelady CA. Use of MyPyramid Menu Planner for Moms in a weight-loss intervention during lactation. J Acad Nutr Diet 2012;112(4):553–8. 66. Colleran HL, Wideman L, Lovelady CA. Effects of energy restriction and exercise on bone mineral density during lactation. Med Sci Sport Ex 2012;44(8):1570–9<br>37. Craigie AM, Macleod M, Barton KL, Treweek S, Anderson AS. Supporting postpartum weight loss in women living in deprived communities: design implications for a randomised control trial. Eur J Clin Nutr 2011;65(8):952–8.<br>38. Herring SJ, Cruice JF, Bennett GG, Davey A, Foster GD. Using Technology to Promote Postpartum Weight Loss in Urban, Low-Income Mothers: A Pilot Randomized Controlled Trial. J Nutr Educ Behav 2014;46(6):610–5.<br>39. Huseinovic E, Winkvist A, Bertz F, Berte´us Forslund H, Brekke HK. Eating frequency, energy intake and body weight during a successful weight loss trial in overweight and obese postpartum women. Eur J Clin Nut 2014;68(1):71–6.<br>40. Leermakers EA, Anglin K, Wing RR. Reducing postpartum weight retention through a correspondence intervention. Int J Obes Relat Metab Disord 1998;22(11):1103–9<br>41. Lovelady CA, Garner KE, Moreno KL, Williams JP. The effect of weight loss in overweight, lactating women on the growth of their infants. New Engl J Med 2000;342(7):449–53.<br>42. Ostbye T, Krause KM, Lovelady CA, Morey MC, Bastian LA, Peterson BL, et al. Active Mothers Postpartum. A Randomized Controlled Weight-Loss Intervention Trial. Am J Prev Med 2009;37(3):173–80.<br>43. O'Toole ML, Sawicki MA, Artal R. Structured diet and physical activity prevent postpartum weight retention. J Womens Health 2003;12(10):991–8.<br>44. Stendell-Hollis NR, Thompson PA, West JL, Wertheim BC, Thomson CA. A comparison of Mediterranean-style and MyPyramid diets on weight loss and inflammatory biomarkers in postpartum breastfeeding women. J Womens Health 2013;22(1):48–57.<br>45. Wiltheiss GA, Lovelady CA, West DG, Brouwer RJ, Krause KM, Østbye T. Diet quality and weight change among overweight and obese postpartum women enrolled in a behavioral intervention program. J Acad Nutr Diet 2013;113(1): 54–62
Wiebe *et al.* 2015 [62]	1. Price BB, Amini SB, Kappeler K. Exercise in pregnancy: effect on fitness and obstetric outcomes-a randomized trial. Med SciSports Exerc 2012;44:2263–9<br>2. Tomic V, Sporis G, Tomic J, Milanovic Z, Zigmundovac-Klaic D,Pantelic S. The effect of maternal exercise during pregnancy on abnormal fetal growth. Croat Med J 2013;54:362–8.<br>3. Avery MD, Leon AS, Kopher RA. Effects of a partially home-based exercise program for women with gestational diabetes. Obstet Gynecol 1997;89:10–5.21.<br>4. Baciuk EP, Pereira RI, Cecatti JG, Braga AF, Cavalcante SR. Water aerobics in pregnancy: cardiovascular response, labor and neonatal outcomes. Reprod Health 2008;5:10.<br>5. Barakat R, Pelaez M, Lopez C, Montejo R, Coteron J. Exercise during pregnancy reduces the rate of cesarean and instrumental deliveries: results of a randomized controlled trial. J MaternFetal Neonatal Med 2012;25:2372–6.<br>6. Clapp JF III, Kim H, Burciu B, Lopez B. Beginning regular exercise in early pregnancy: effect on fetoplacental growth.Am J Obstet Gynecol 2000;183:1484–8.<br>7. de Oliveria Melo AS, Silva JL, Tavares JS, Barros VO, Leite DF, Amorim MM. Effect of a physical exercise program during pregnancy on uteroplacental and fetal blood flow and fetal growth: a randomized controlled trial. Obstet Gynecol 2012;120:302–10.<br>8. Garshasbi A, Faghih Zadeh S. The effect of exercise on the intensity of low back pain in pregnant women. Int J GynaecolObstet 2005;88:271–5.<br>9. Hopkins SA, Baldi JC, Cutfield WS, McCowan L, Hofman PL. Exercise training in pregnancy reduces offspring size without changes in maternal insulin sensitivity. J Clin Endocrinol Metab 2010;95:2080–8.<br>10. Kasawara KT, Burgos CS, do Nascimento SL, Ferreira NO, Surita FG, Pinto E Silva JL. Maternal and perinatal outcomes of exercise in pregnant women with chronic hypertension and/or previous preeclampsia: a randomized controlled trial. ISRN Obstet Gynecol 2013;2013:857047.<br>11. Khaledan A, Mirdar S, Motahari Tabari N, Ahmad SM. Effect of an aerobic exercise program on fetal growth in pregnant women. J HAYAT 2010;16:55–64.<br>12. Kihlstrand M, Stenman B, Nilsson S, Axelsson O. Water-gymnastics reduced the intensity of back/low back pain in pregnant women. Acta Obstet Gynecol Scand 1999;78:180–5.<br>13. Marquez-Sterling S, Perry AC, Kaplan TA, Halberstein RA, Signorile JF. Physical and psychological changes with vigorous exercise in sedentary primigravidae. Med Sci Sports Exerc2000;32:58–62.<br>14. Pinzón DC, Zamora K, Martínez JH, Floréz-López ME, dePlata AC, Mosquera M, et al. Type of delivery and gestational age is not affected by pregnant Latin-American women engaging in vigorous exercise: a secondary analysis of data from a controlled randomized trial. Rev Salud Publica (Bogota)2012;14:731–43.<br>15. Barakat R, Pelaez M, Montejo R, Luaces M, Zakynthinaki M. Exercise during pregnancy improves maternal health perception: a randomized controlled trial. Am J Obstet Gynecol 2011;204:402.e1–7.<br>16. Barakat R, Cordero Y, Coteron J, Luaces M, Montejo R. Exercise during pregnancy improves maternal glucose screen at 24–28 weeks: a randomised controlled trial. Br J Sports Med 2012;46:656–61.

Review	References of included behavioural interventions delivered in pregnancy
	17. Barakat R, Pelaez M, Lopez C, Lucia A, Ruiz JR. Exercise during pregnancy and gestational diabetes-related adverse effects: a randomised controlled trial. Br J Sports Med 2013;47:630–6.<br>18. Haakstad LA, Bø K. Exercise in pregnant women and birthweight: a randomized controlled trial. BMC Pregnancy Child-birth 2011;11:66.<br>19. Hui A, Back L, Ludwig S, Gardiner P, Sevenhuysen G, Dean H, et al. Lifestyle intervention on diet and exercise reduced excessive gestational weight gain in pregnant women under a randomised controlled trial. BJOG 2012;119:70–7.<br>20. Hui AL, Ludwig S, Gardiner P, Sevenhuysen G, Murray R, Morris M, et al. Community-based exercise and dietary intervention during pregnancy: a pilot study. Can J Diabetes 2004;28:1–7.<br>21. Nascimento SL, Surita FG, Parpinelli MÂ, Siani S, Pinto eSilva JL. The effect of an antenatal physical exercise programme on maternal/perinatal outcomes and quality of life in overweight and obese pregnant women: a randomised clinical trial. BJOG 2011;118:1455–63.<br>22. Oostdam N, van Poppel MN, Wouters MG, Eekhoff EM, Bekedam DJ, Kuchenbecker WK, et al. No effect of the FitFor2exercise programme on blood glucose, insulin sensitivity, and birthweight in pregnant women who were overweight and at risk for gestational diabetes: results of a randomised controlled trial. BJOG 2012;119:1098–107.<br>23. Stafne SN, Salvesen KÂ, Romundstad PR, Eggebø TM,Carlsen SM, Mørkved S. Regular exercise during pregnancy to prevent gestational diabetes: a randomized controlled trial.Obstet Gynecol 2012;119:29–36.<br>24. Barakat R, Perales M, Bacchi M, Coteron J, Refoyo I. A program of exercise throughout pregnancy. Is it safe to mother and newborn? Am J Health Promot 2014;29:2–8.<br>25. Cordero Y, Mottola MF, Vargas J, Blanco M, Barakat R. Exercise is associated with a reduction in gestational diabetes melli-tus. Med Sci Sports Exerc 2014 Oct 20 [Epub ahead of print].<br>26. Ruiz JR, Perales M, Pelaez M, Lopez C, Lucia A, Barakat R. Supervised exercise-based intervention to prevent excessive gestational weight gain: a randomized controlled trial. MayoClin Proc 2013;88:1388–97.<br>27. Murtezani A, Pacarada M, Ibraimi Z, Nevzati A, Abazi N. The impact of exercise during pregnancy on neonatal outcomes: a randomized controlled trial. J Sports Med Phys Fitness 2014;54:802–8.<br>28. Barakat R, Lucia A, Ruiz JR. Resistance exercise training dur-ing pregnancy and new-born's birth size: a randomised con-trolled trial. Int J Obes (Lond) 2009;33:1048–57.
Yin *et al.* 2013 [63]	1. Price BB, Amini SB, Kappeler K. Exercise in pregnancy: effect on fitness and obstetric outcomes—a randomized trial. Med Sci Sports Exerc 2012;44:2263–9.<br>2. Stafne SN, Salvesen KA, Romundstad PR, et al. Regular exercise during pregnancy to prevent gestational diabetes: a randomized controlled trial. Obstet Gynecol 2012;119:29–36.<br>3. Callaway LK, Colditz PB, Byrne NM,et al. Prevention of gestational diabetes: feasibility issues for an exercise intervention in obese pregnant women.DiabetesCare2010;33:1457–9.<br>4. Barakat R, Cordero Y, Coteron J, et al. Exercise during pregnancy improves maternal glucose screen at 24–28 weeks: a randomised controlled trial.Br J Sports Med2011;46:656–61.<br>5. Barakat R, Ruiz JR, Stirling JR,et al. Type of delivery is not affected by light resistance and toning exercise training during pregnancy: a randomized controlled trial. Am J Obstet Gynecol 2009;201:509.e1–6.<br>6. Rakhshani A, Nagarathna R, Mhaskar R, et al. The effects of yoga in prevention of pregnancy complications in high-risk pregnancies: a randomized controlled trial. Prev Med 2012;55:333–40
Shieh *et al.* 2018 [64]	1. Bisson M, Almeras N, Dufresne SS, Frenette J, Tremblay A,Marc I. A 12-week exercise program for pregnant women with obesity to improve physical activity levels: an open randomised preliminary study. PLoS One 2015;10: e0137742.https://doi.org/10.1371/journal.pone.0137742.<br>2. Bogaerts AF, Devlieger R, Nuyts E, Witters I, Gyselaers W, Vanden Bergh BR. Effects of lifestyle intervention in obese pregnant women on gestational weight gain and mental health: a randomized controlled trial. Int J Obes (Lond)2013;37: 814–821.https://doi.org/10.1038/ijo.2012.162.<br>3. Dekker Nitert D, Barrett HL, Denny KJ, McIntyre HD, Callaway LK. Exercise in pregnancy does not alter gestational weight gain, MCP-1 or leptin in obese women. Aust N Z JObstet Gynaecol 2015;55(1): 27–33.https://doi.org/10.1111/ajo.12300.<br>4. Dodd JM, Turnbull D, McPhee AJet al. Antenatal lifestyle advice for women who are overweight or obese: LIMIT randomised trial.BMJ2014;348: g1285.https://doi.org/10.1136/bmj.g1285.<br>5. Garnæs KK, Morkved S, Salvesen O, Moholdt T. Exercise training and weight gain in obese pregnant women: a randomized controlled trial (ETIP Trial).PLoS Med2016;13: e1002079.https://doi.org/10.1371/journal.pmed.1002079.<br>6. Guelinckx I, Devlieger R, Mullie P, Vansant G. Effect of lifestyle intervention on dietary habits, physical activity, and gestational weight gain in obese pregnant women: a randomized controlled trial. Am J Clin Nutr 2010;91: 373–380.https://doi.org/10.3945/ajcn.2009.28166.<br>7. Harrison CL, Lombard CB, Strauss BJ, Teede HJ. Optimizing healthy gestational weight gain in women at high risk of gestational diabetes: a randomized controlled trial. Obesity (Silver Spring)2013;21: 904–909.https://doi.org/10.1002/oby.20163.<br>8. Hawkins M, Hosker M, Marcus BH et al. A pregnancy lifestyle intervention to prevent gestational diabetes risk factors in overweight Hispanic women: a feasibility randomized controlled trial. Diabet Med 2015;32: 108–115.https://doi.org/10.1111/dme.12601.<br>9. Kong KL, Campbell CG, Foster RC, Peterson AD, Lanningham-Foster L. A pilot walking program promotes moderate-intensity physical activity during pregnancy. Med Sci Sports Exerc2014;46:462–471.https://doi.org/10.1249/MSS.0000000000000141.<br>10. McCarthy E, Walker S, Ugoni A, Lappas M, Leong O, Shub A. Self-weighing and simple dietary advice for overweight and obese pregnant women to reduce obstetric complications without impact on quality of life: a randomised controlled trial. BJOG 2016;123:965–973.<br>11. Nascimento SL, Surita FG, Parpinelli MA, Siani S, Pinto e SilvaJL. The effect of an antenatal physical exercise programme on maternal/perinatal outcomes and quality of life in overweight and obese pregnant women: a randomised clinical trial.BJOG2011;118: 1455–1463.https://doi.org/10.1111/j.1471-0528.2011.03084.x.

Review	References of included behavioural interventions delivered in pregnancy
	12. Petrella E, Malavolti M, Bertarini Vet al. Gestational weight gain in overweight and obese women enrolled in a healthy lifestyle and eating habits program. J Matern Fetal Neonatal Med2014;27:1348–1352.https://doi.org/10.3109/14767058.2013.858318.<br>13. Poston L, Bell R, Croker Het al. Effect of a behavioural intervention in obese pregnant women (the UPBEAT study): a multicentre, randomised controlled trial. Lancet Diabetes Endocrinol 2015;3: 767–777.https://doi.org/10.1016/s2213-8587(15)00227-2.<br>14. Quinlivan JA, Lam LT, Fisher J. A randomised trial of a four-step multidisciplinary approach to the antenatal care of obese pregnant women. Aust N Z J Obstet Gynaecol 2011;51: 141–146.https://doi.org/10.1111/j.1479-828X.2010.01268.x.<br>15. Renault KM, Norgaard K, Nilas Let al. The Treatment of Obese Pregnant Women (TOP) study: a randomized controlled trial of the effect of physical activity intervention assessed by pedometer with or without dietary intervention in obese pregnant women. AmJ Obstet Gynecol 2014;210: 134e131–134e139.https://doi.org/10.1016/j.ajog.2013.09.029.<br>16. Rhodes ET, Pawlak DB, Takoudes TCet al. Effects of a low-glycemic load diet in overweight and obese pregnant women: a pilot randomized controlled trial. Am J Clin Nutr 2010;92: 1306–1315.https://doi.org/10.3945/ajcn.2010.30130.<br>17. Seneviratne SN, Jiang Y, Derraik Jet al. Effects of antenatal exercise in overweight and obese pregnant women on maternal and perinatal outcomes: a randomised controlled trial.BJOG2016;123: 588–597.https://doi.org/10.1111/1471-0528.13738.<br>18. Simmons D, Jelsma JG, Galjaard Set al. Results from a European multicentre randomized trial of physical activity and/or healthy eating to reduce the risk of gestational diabetes mellitus: the DALI lifestyle pilot. Diabetes Care 2015;38: 1650–1656.https://doi.org/10.2337/dc15-0360.<br>19. Szmeja MA, Cramp C, Grivell RM, Deussen AR, Yelland LN,Dodd JM. Use of a DVD to provide dietary and lifestyle information to pregnant women who are overweight or obese: a nested randomised trial. BMC Pregnancy Childbirth 2014;14: 409.https://doi.org/10.1186/s12884-014-0409-8.<br>20. Thornton YS, Smarkola C, Kopacz SM, Ishoof SB. Perinatal outcomes in nutritionally monitored obese pregnant women: a randomized clinical trial. J Natl Med Assoc 2009;101: 569–577.https://doi.org/10.1016/S0027-9684(15)30942-1.<br>21. Vesco KK, Karanja N, King JCet al. Efficacy of a group-based dietary intervention for limiting gestational weight gain among obese women: a randomized trial. Obesity 2014;22: 1989–1996.https://doi.org/10.1002/oby.20831.<br>22. Vinter CA, Jensen DM, Ovesen P, Beck-Nielsen H, Jorgensen JS. The LiP (Lifestyle in Pregnancy) study: a randomized controlled trial of lifestyle intervention in 360 obese pregnant women.DiabetesCare2011;34: 2502–2507.https://doi.org/10.2337/dc11-1150.<br>23. Wolff S, Legarth J, Vangsgaard K, Toubro S, Astrup A. A randomized trial of the effects of dietary counseling on gestational weight gainand glucose metabolism in obese pregnant women. Int J Obes (Lond)2008;32: 495–501.https://doi.org/10.1038/sj.ijo.0803710.
Bennett *et al.* 2017 [65]	1. Brownfoot FC, Davey MA, Kornman L. Routine weighing to reduce excessive antenatal weight gain: a randomised controlled trial. BJOG 2016;123(2):254–61.<br>2. Herring SJ, Cruice JF, Bennett GG, Rose MZ, Davey A, Foster GD. Preventing excessive gestational weight gain among African American women: a randomized clinical trial. Obesity 2016;24(1):30–6.<br>3. Jeffries K, Shub A, Walker SP, Hiscock R, Permezel M. Reducing excessive weight gain in pregnancy: a randomised controlled trial. Med J Aust 2009;191(8):429–33.<br>4. Quinlivan JA, Lam LT, Fisher J. A randomised trial of a four-step multidisciplinary approach to the antenatal care of obese pregnant women. Aust N Z J Obstet Gynaecol 2011;51(2):141–6.<br>5. Syngelaki A, Nicolaides KH, Balani J, Hyer S, Akolekar R, Kotecha R, et al. Metformin versus placebo in obese pregnant women without diabetes mellitus. N Engl J Med 2016;374(5):434–43.<br>6. Moses RG, Casey SA, Quinn EG, Cleary JM, Tapsell LC, Milosavljevic M, et al. Pregnancy and Glycemic Index Outcomes study: effects of low glycemic index compared with conventional dietary advice on selected pregnancy outcomes. Am J Clin Nutr 2014;99(3):517–23.<br>7. Simmons D, Jelsma JGM, Galjaard S, Devlieger R, van Assche A, Jans G, et al. Results from a European multicenter randomized trial of physical activity and/or healthy eating to reduce the risk of gestational diabetes mellitus: the DALI lifestyle pilot. Diabetes Care 2015;38(9):1650–6.<br>8. Markovic TP, Muirhead R, Overs S, Ross GP, Louie JCY, Kizirian N, et al. Randomized controlled trial investigating the effects of a low-glycemic index diet on pregnancy outcomes in women at high risk of gestational diabetes mellitus: the GI baby 3 study. Diabetes Care 2016;39(1):31–8.<br>9. Bogaerts A, Devlieger R, Nuyts E, Witters I, Gyselaers W, Vanden Bergh B. Effects of lifestyle intervention in obese pregnant women on gestational weight gain and mental health: a randomized controlled trial. Int J Obes 2013;37(6):814–21.<br>10. Hui AL, Back L, Ludwig S, Gardiner P, Sevenhuysen G, Dean HJ, et al. Effects of lifestyle intervention on dietary intake, physical activity level, and gestational weight gain in pregnant women with different pre-pregnancy Body Mass Index in a randomized control trial. BMC Pregnancy and Childbirth 2014;14:331<br>11. Kong KL, Campbell CG, Foster RC, Peterson AD, Lanningham-Foster L. A pilot walking program promotes moderate-intensity physical activity during pregnancy. Med Sci Sports Exerc 2014;46(3):462–71.[27]Phelan S, Phipps MG, Abrams B, Darroch F, Schaffner A, Wing RR. Randomized trial of a behavioral intervention to prevent excessive gestational weight gain: the Fit for Delivery Study. Am J Clin Nutr 2011;93(4):772–9.<br>12. Polley BA, Wing RR, Sims CJ. Randomized controlled trial to prevent excessive weight gain in pregnant women. Int J Obes 2002;26(11):1494–502.<br>13. Ruiz JR, Perales M, Pelaez M, Lopez C, Lucia A, Barakat R. Supervised exercise-based intervention to prevent excessive gestational weight gain: a randomized controlled trial. Mayo Clin Proc 2013;88(12):1388–97.

Review	References of included behavioural interventions delivered in pregnancy
	14. Renault KM, Nørgaard K, Nilas L, Carlsen EM, Cortes D, Pryds O, et al. The Treatment of Obese Pregnant Women (TOP)study: a randomized controlled trial of the effect of physical activity intervention assessed by pedometer with or without dietary intervention in obese pregnant women. Am J ObstetGynecol 2014;210(2):134. e1-9.<br>15. Dodd JM, Turnbull D, McPhee AJ, Deussen AR, Grivell RM, Yelland LN, et al. Antenatal lifestyle advice for women who are overweight or obese: LIMIT randomised trial. BMJ 2014;348:g1285.<br>16. Harrison CL, Lombard CB, Strauss BJ, Teede HJ. Optimizing healthy gestational weight gain in women at high risk of gestational diabetes: a randomized controlled trial. Obesity 2013;21(5):904–9.[33]Hawkins M, Hosker M, Marcus BH, Rosal MC, Braun B, StanekEJ, et al. A pregnancy lifestyle intervention to prevent gestational diabetes risk factors in overweight Hispanic women: a feasibility randomized controlled trial. Diabetic Med 2015;32(1):108–15.<br>17. Hui A, Back L, Ludwig S, Gardiner P, Sevenhuysen G, Dean H, et al. Lifestyle intervention on diet and exercise reduced excessive gestational weight gain in pregnant women under a randomised controlled trial. BJOG 2012;119(1):70–7.<br>18. Jiang YSL. Influence of appropriate nutrition and gymnastic training on pregnancy outcomes. Chin J Women Child Health 2015;6(5):47–8.<br>19. Jing W, Huang Y, Liu X, Luo B, Yang Y, Liao S. The effect of a personalized intervention on weight gain and physical activity among pregnant women in China. Int J Gynaecol Obstet 2015;129(2):138–41.<br>20. Korpi-Hyövälti E, Schwab U, Laaksonen DE, Linjama H, Heinonen S, Niskanen L. Effect of intensive counselling on the quality of dietary fats in pregnant women at high risk of gestational diabetes mellitus. Br J Nutr 2012;108(5):910–7.<br>21. Liao Q, Zhu S, Pang Y, Wei Z. The effect of individual nutrition instruction on nutritional status and birth outcome ofpregnant women. Matern Child Healthcare Chin 2012;27(28):4388–90.<br>22. Luoto RM, Kinnunen TI, Aittasalo M, Ojala K, Mansikkamaki K, Toropainen E, et al. Prevention of gestational diabetes: design of a cluster-randomized controlled trial and one-year follow-up. BMC Pregn Childbirth 2010;10(39).<br>23. McCarthy EA, Walker SP, Ugoni A, Lappas M, Leong O, Shub A. Self-weighing and simple dietary advice for overweight and obese pregnant women to reduce obstetric complications without impact on quality of life: a randomised controlled trial. BJOG 2016;123(6):965–73.<br>24. Petrella E, Malavolti M, Bertarini V, Pignatti L, Neri I, Battistini NC, et al. Gestational weight gain in overweight and obese women enrolled in a healthy lifestyle and eating habits program. J Matern Fetal Neonatal Med 2014;27(13):1348–52.<br>25. Poston L, Briley AL, Barr S, Bell R, Croker H, Coxon K, et al. Developing a complex intervention for diet and activity behaviour change in obese pregnant women (the UPBEAT trial); assessment of behavioural change and process evaluation in a pilot randomised controlled trial. BMC Pregn Childbirth 2013;13(148).<br>26. Sagedal LR, Øverby NC, Bere E, Torstveit MK, Lohne-Seiler H, Småstuen M, et al. Lifestyle intervention to limit gestational weight gain: the Norwegian Fit for Delivery randomised controlled trial. BJOG 2016;124(1):97–109.<br>27. Thornton YS, Smarkola C, Kopacz SM, Ishoof SB, et al. Perinatal outcomes in nutritionally monitored obese pregnant women: a randomised clinical trial. J Natl Med Assoc 2009:101:6 569-77<br>28. Walsh JM, McGowan CA, Mahony R, Foley ME, McAuliffe FM. Low glycaemic index diet in pregnancy to prevent macrosomia (ROLO study): randomised control trial. BMJ 2012;345:e5605.<br>29. Wolff S, Legarth J, Vangsgaard K, Toubro S, Astrup A. A randomized trial of the effects of dietary counselling on gestational weight gain and glucose metabolism in obese pregnant women. Int J Obes 2008;32(3):495–501.<br>30. Xiao R, Liu W, Li Y, Ding J, Yin J. Effects of using DOHad nutritional monitoring on the grading of nutrition management during pregnancy. Med J Chin People's Armed Police Force 2015;26(5):493–5.<br>31. Ye L, Xu H, Ye W. Follow-up application analysis of individualized prenatal nutrition education in the community. Med Innov Chin 2016;13(2):106–10.<br>32. Zhang H, Yang Z. Effect analysis of personal medical nutrition therapy combined with exercise therapy for gestational diabetes mellitus. Jinlin Med J 2012;33(16):3369–72.<br>33. Barakat R, Lucia A, Ruiz JR. Resistance exercise training during pregnancy and newborn's birth size: a randomised controlled trial. Int J Obes 2009;33(9):1048–57.<br>34. Barakat R, Pelaez M, Cordero Y, Perales M, Lopez C, Coteron J,et al. Exercise during pregnancy protects against hypertension and macrosomia: randomized clinical trial. AmJ Obs Gyn 2016;214(5):e649–51.<br>35. Barakat R, Pelaez M, Lopez C, Lucia A, Ruiz JR. Exercise during pregnancy and gestational diabetes-related adverse effects: a randomised controlled trial. Br J Sports Med 2013;47(10):630–6.<br>36. Barakat R, Pelaez M, Montejo R, Luaces M, Zakynthinaki M. Exercise during pregnancy improves maternal health perception: a randomized controlled trial. Am J Obs Gyn 2011;204(5):402. e1-7.<br>37. Barakat R, Pelaez M, Montejo R, Refoyo I, Coteron J. Exercise throughout pregnancy does not cause preterm delivery: a randomized, controlled trial. J Phys Act Health 2014;11(5):1012–7.<br>38. Bisson M, Alméras N, Dufresne SS, Robitaille J, Rhéaume C,Bujold E, et al. A 12-week exercise program for pregnant women with obesity to improve physical activity levels: an open randomised preliminary study. PLoS ONE 2015;10(9):e0137742.<br>39. Oostdam N, Van Poppel MNM, Wouters MGAJ, Eekhoff EMW, Bekedam DJ, Kuchenbecker WKH, et al. No effect of theFitFor2 exercise programme on blood glucose, insulin sensitivity, and birthweight in pregnant women who were overweight and at risk for gestational diabetes: results of a randomised controlled trial. BJOG 2012;119(9):1098–107.<br>40. Vesco KK, Karanja N, King JC, Gillman MW, Leo MC, Perrin N, et al. Efficacy of a group-based dietary intervention for limiting gestational weight gain among obese women: a randomized trial. Obesity 2014;22(9):1989–96.<br>41. Vinter CA, Jensen DM, Ovesen PG, Beck-Nielsen H, Jørgensen JS. Lifestyle and pregnancy (LIP) study: a randomized controlled trial of lifestyle intervention in 360 obese pregnant women. Diabetes Care 2011;34:2502–7.<br>42. Wang Y, Li A, Yang H, Wu C, Li Y, Wei Z, et al. Effect of individualised diet intervention on the incidence of caesarean section on pregnant women with a normal BMI. Chin JPrevent Med 2016;19(3):22–228.<br>43. Zhang L, Zhao M, Li G. Effects of a lifestyle intervention on gestational weight gain and birth outcome in obese pregnant women. Beijing Med J 2015;37(10):1001–2.

S7d: Unique publications included in diet and/or physical activity reviews

Diet and/or physical activity unique publications

1. Aaltonen J, Ojala T, Laitinen K, Piirainen TJ, Poussa TA, Isolauri E. Evidence of infant blood pressure programming by maternal nutrition during pregnancy: a prospective randomized controlled intervention study. J Pediatr 2008;152:79–84.
2. Abdel-Aziz S., Hegazy I., Mohamed D., Abu El Kasem M., Hagag S. Effect of dietary counseling on preventing excessive weight gain during pregnancy. Public Health. 2018;154:172–181. doi: 10.1016/j.puhe.2017.10.014.
3. Abirami P, Judie A. Integrated approach of yoga therapy on maternal and fetal outcome in gestational diabetes mellitus. International Journal of Pharmaceutical and Clinical Research 2015;7:377–82.
4. Abirami P, Judie A. Reduction of risk on newly detected gestational diabetes mellitus by multi model intervention - A hospital based study. 2014.
5. Aittasalo M, Raitanen J, Kinnunen TI, Ojala K, Kolu P, Luoto R. Is intensive counseling in maternity care feasible and effective in promoting physical activity among women at risk for gestational diabetes? Secondary analysis of a cluster randomized NELLI study in Finland. International Journal of Behavioral Nutrition and Physical Activity 2012;9: 104.?ISRCTN33885819. Primary prevention of gestational diabetes among women at risk: a cluster-randomized controlled trial. isrctn.com/ISRCTN33885819 (first received 12 September 2007).?
6. Albright CL, Steffen AD, Wilkens LR et al. Effectiveness of a 12-month randomized clinical trial to increase physical activity in multiethnic postpartum women: results from Hawaii's Na Mikimiki Project. Prev Med 2014; 69: 214–223.
7. Althuizen E, van der Wijden C, van Mechelen W, Seidell J, van Poppel M. The effect of a counselling intervention on weight changes during and after pregnancy: a randomised trial. BJOG : an International Journal of Obstetrics and Gynaecology 2013;120(1):92–9.
8. Althuizen E, Van Poppel MN, Seidell JC, Van der Wijden C, Van Mechelen W. Design of the new life(style) study: a randomised controlled trial to optimise maternal weight development during pregnancy. BMC Public Health 2006;6:168.
9. Angel MD, De Haene J, Perez M, Hernandez G, Castaneda D, King JC. Dietary patterns associated with gestational weight gain and fat mass gain in overweight and obese pregnant women. FASEB Journal 2011;25:783.15.
10. Anjana RM, Sudha V, Lakshmipriya N, et al. Physical activity patterns and gestational diabetes outcomes - The wings project. Diabetes Research & Clinical Practice. 2016.
11. Artal R, Catanzaro RB, Gavard JA, Mostello DJ, Friganza JC. A lifestyle intervention of weight-gain restriction: diet and exercise in obese women with gestational diabetes mellitus. Appl Physiol Nutr Metab 2007;32:596–601.
12. Artal R, Platt LD, Sperling M, Kammula RK, Jilek J, Nakamura R. I. Maternal cardiovascular and metabolic responses in normal pregnancy. 1981.
13. Artal R, Rutherford S, Romem Y, Kammula RK, Dorey FJ, Wiswell RA. Fetal heart rate responses to maternal exercise. American Journal of Obstetrics & Gynecology. 1986.
14. Artal R, Wiswell R, Romem Y. Hormonal responses to exercise in diabetic and nondiabetic pregnant patients. 1985.
15. Asbee S M, Jenkins T R, Butler J R, White J, Elliot M, Rutledge A.Preventing excessive weight gain during pregnancy through dietary and lifestyle counseling: a randomized controlled trial Obstet Gynecol 2009113(2 Pt 1):305–312. [PubMed] [Google Scholar]
16. Asbee SM, Jenkins TR, Butler JR, White J, Elliot M, Rutledge A. Dietary counseling prevents excessive weight gain during pregnancy, a randomized controlled trial. Obstetrics and Gynecology 2008;111(4 Suppl):6S.?
17. Aşcı Ö, Rathfisch G. Effect of lifestyle interventions of pregnant women on their dietary habits, lifestyle behaviors, and weight gain: a randomized controlled trial. J Health Popul Nutr. 2016;35:7. [PMC free article] [PubMed] [Google Scholar]
18. Asgharian H, Homayouni-Rad A, Mirghafourvand M, Mohammad-Alizadeh-Charandabi S. Effect of probiotic yoghurt on plasma glucose in overweight and obese pregnant women: a randomized controlled clinical trial. Eur J Nutr 2019.
19. Avery MD, Leon AS, Kopher RA. Effects of a partially home-based exercise program for women with gestational diabetes. Obstet Gynecol 1997;89:10–5.21.
20. Avery MD, Walker AJ. Acute effect of exercise on blood glucose and insulin levels in women with gestational diabetes. 2001.
21. Bacchi M., Mottola M., Perales M., Refoyo I., Barakat R. Aquatic activities during pregnancy prevent excessive maternal weight gain and preserve birth weight: A randomized clinical trial. Am. J. Health Promot. 2018;32:729–735. doi: 10.1177/0890117117697520.
22. Baciuk EP, Pereira RI, Cecatti JG, Braga AF, Cavalcante SR. Water aerobics in pregnancy: cardiovascular response, labor and neonatal outcomes. Reprod Health. 2008; https://doi.org/10.1186/1742-4755-5-10.
23. Badon SE, Wander PL, Qiu C, Miller RS, Williams MA, Enquobahrie DA. Maternal Leisure Time Physical Activity and Infant Birth Size. Epidemiology. 2016;27(1):74-81.
24. Badrawi H, Hassanein MK, Badraoui MHH, Wafa YA, Shawky HA, Badrawi N. Pregnancy outcome in obese pregnant mothers. J Perinat Med 1992;20:203.
25. Banerjee A, V. Mahalakshmi V, R. Baranitharan R. Effect of Antenatal Exercise Program with and without Abdominal Strengthening Exercises on Diastasis Rectus Abdominis - A Post Partum Follow Up. Indian Journal of Physiotherapy & Occupational Therapy. 2013;7(4):123-6.

Diet and/or physical activity unique publications
26. Barakat R, Alonso G, Rojo JJ. Physical exercise during pregnancy and its relationship with the duration of stages of labor. Prog Obstet Ginecol. 2005;48(2):61-8.
27. Barakat R, Cordero Y, Coteron J, et al. Exercise during pregnancy improves maternal glucose screen at 24-28 weeks: a randomised controlled trial. Br J Sports Med 2012;46:656–61.
28. Barakat R, Lucia A, Ruiz J. Resistance exercise training during pregnancy and newborn's birth size: a randomised controlled trial. Int J Obes V 33 20052009;1048–57.
29. Barakat R, Palaez M, Montejo R, Refoyo I, Coteron J. Exercise throughout pregnancy does not cause preterm delivery: a randomized, controlled trial. J Phys Act Health. 2014;11(5):1012.
30. Barakat R, Pelaez M, Cordero Y, et al. Exercise during pregnancy protects against hypertension and macrosomia: randomized clinical trial. Am J Obstet Gynecol 2016;214(5):649.e1–649.e8.
31. Barakat R, Pelaez M, Lopez C, Lucia A, Ruiz JR (2013) Exercise during pregnancy and gestational diabetes-related adverse effects: a randomised controlled trial. Br J Sports Med 47:630–636
32. Barakat R, Pelaez M, Lopez C, Montejo R, Coteron J. Exercise during pregnancy reduces the rate of cesarean and instrumental deliveries: results of a randomized controlled trial. J Matern Fetal Neonatal Med 2012;25:2372-6.
33. Barakat R, Pelaez M, Montejo R, et al. Exercise during pregnancy improves maternal health perception: a randomized controlled trial. Am J Obstet Gynecol 2011;204:402. e1–7.
34. Barakat R, Perales M, Bacchi M, Coteron J, Refoyo I. A program of exercise throughout pregnancy. Is it safe to mother and newborn? Am J Health Promot 2014;29:2-8.
35. Barakat R, Ruiz JR, Stirling JR, et al. Type of delivery is not affected by light resistance and toning exercise training during pregnancy: a randomized controlled trial. Am J Obstet Gynecol 2009;201:590.e1–590.e6.
36. Barakat R, Stirling JR, Lucia A. Does exercise training during pregnancy affect gestational age? A randomised controlled trial. Br J Sports Med 2008;42:674-8.
37. Barakat R, Cordero Y, Coteron J, Luaces M, Montejo R. Exercise during pregnancy improves maternal glucose screen at 24–28 weeks: a randomised controlled trial. Br J Sports Med. 2012; 46: 656– 61
38. Barakat R, Pelaez M, López C, Montejo R, Coteron J. Exercise during pregnancy reduces the rate of cesarean and instrumental deliveries: results of a randomized controlled trial. J Matern Fetal Neonatal Med. 2012; 25: 2372– 6.
39. Barakat R., Franco E., Perales M., Lopez C., Mottola M. Exercise during pregnancy is associated with shorter duration of labor: A randomized clinical trial. Eur. J. Obstet. Gynecol. Reprod. Biol. 2018;224:33–40. doi: 10.1016/j.ejogrb.2018.03.009
40. Battle CL, Uebelacker LA, Magee SR, Sutton KA, Miller IW. Potential for prenatal yoga to serve as an intervention to treat depression during pregnancy. Womens Health Issues. 2015;25(2):134.
41. Bech BH, Obel C, Henriksen TB, Olsen J. Effect of reducing caffeine intake on birth weight and length of gestation: randomised controlled trial. BMJ 2007;334:409–12.
42. Bechtel-Blackwell DA. Computer-assisted self-interview and nutrition education in pregnant teens. Clin Nurs Res 2002;11:450-62.
43. Beckmann CR, Beckmann CA. Effect of a structured antepartum exercise program on pregnancy and labor outcome in primiparas. J Reprod Med. 1990;35(7):704-9.
44. Beddoe AE, Yang CPP, Kennedy HP, Weiss SJ, Lee KA. The effects of mindfulness-based yoga during pregnancy on maternal psychological and physical distress. Journal of obstetric, gynecologic, and neonatal nursing : JOGNN. 2009;38(3):310.
45. Begum MR, Khanam NN, Quadir E, et al. Prevention of gestational diabetes mellitus by continuing metformin therapy throughout pregnancy in women with polycystic ovary syndrome. J Obstet Gynaecol Res 2009;35:282–286.
46. Bell RJ, Palma SM, Lumley JM. The effect of vigorous exercise during pregnancy on birthweight. Aust N Z J Obstet Gynaecol 1995;35:46–51.
47. Bell RJ, Palma SM. Antenatal exercise and birth-weight. Aust N Z J Obstet Gynaecol 2000;40:70-3.
48. Berkowitz GS, Kelsey JL, Holford TR, Berkowitz RL. Physical activity and the risk of spontaneous preterm delivery. J Reprod Med 1983;28:581–8
49. Bershadsky S, Trumpfheller L, Kimble HB, Pipaloff D, Yim IS. The effect of prenatal Hatha yoga on affect, cortisol and depressive symptoms. Complement Ther Clin Pract. 2014;20(2):106.
50. Bertz F, Brekke HK, Ellega˚rd L, Rasmussen KM, Wennergren M, Winkvist A. Diet and exercise weight-loss trial in lactating overweight and obese women. Am J Clin Nutr 2012;96(4): 698–705.
51. Bertz F, Winkvist A, Brekke HK. Sustainable weight loss among overweight and obese lactating women is achieved with an energy-reduced diet in line with dietary recommendations: results from the LEVA randomized controlled trial. J Acad Nutr Diet 2015; 115: 78–8
52. Bessinger RC, McMurray RG, Hackney AC. Substrate utilization and hormonal responses to moderate intensity exercise during pregnancy and after delivery. 2002.
53. Bevier WC, Fischer R, Jovanovic L. Treatment of women with an abnormal glucose challenge test (but a normal oral glucose tolerance test) decreases the prevalence of macrosomia. Am J Perinatol 1999;16:269–275.
54. Beyaz EA, Ozcan E, Ketenci A, Beyaz MM. The effectiveness of pregnancy rehabilitation: Effects on low back pain and calf cramps during pregnancy and pregnancy outcome. Nobel Medicus. 2011;7(2):67-74.
55. Bisson M, Almeras N, Dufresne S, Rheaume C, Bujold E, Robitailee J, et al. Exercise improving fitness in obese women during pregnancy: a difference for the mother and child?. Pediatric Academic Societies and Asian Society for Pediatric Research Joint Meeting; 2014 May 3-6;Vancouver, Canada. 2014:Abstract no: 2946.618.
56. Bisson M, Alme´ras N, Dufresne SS, Robitaille J, Rhe´aume C, Bujold E, et al. A 12-week exercise program for pregnant women with obesity to improve physical activity levels: An open randomised preliminary study. PLoS ONE 2015;10:1–17. Dekker [45] Some concerns
57. Bo K, Haakstad LAH. Is pelvic floor muscle training effective when taught in a general fitness class in pregnancy? A randomised controlled trial. Physiotherapy 2011;97(3):190–5.

Diet and/or physical activity unique publications
58. Bo K, Fleten C, Nystad W. Effect of antenatal pelvic floor muscle training on labor and birth. Obstet Gynecol. 2009;113(6):1279-84.
59. Bo K, Hilde G, Staer-Jensen J, Siafarikas F, Tennfjord MK, Engh ME. Does general exercise training before and during pregnancy influence the pelvic floor "opening" and delivery outcome? A 3D/4D ultrasound study following nulliparous pregnant women from mid-pregnancy to childbirth. Br J Sports Med. 2015;49:196-9.
60. Bo S, Rosato R, Ciccone G, Canil S, Gambino R, Poala CB, et al. Simple lifestyle recommendations and the outcomes of gestational diabetes. A 2 x 2 factorial randomized trial. Diabetes Obes Metab. 2014;16(10):1032-5.
61. Bogaerts A. Effect of psycho-education on gestational weight gain and anxiety/depression in obese pregnant women. ClinicalTrials.gov(http://clinicaltrials.gov/) [accessed 21 May 2013] 2011.
62. Bogaerts A F, Devlieger R, Nuyts E, Witters I, Gyselaers W, Van den Bergh B RH. Effects of lifestyle intervention in obese pregnant women on gestational weight gain and mental health: a randomized controlled trial. Int J Obes. 2013;37(06):814–821. [PubMed] [Google Scholar]
63. Bonen A, Campagna P, Gilchrist L, Young DC, Beresford P. Substrate and endocrine responses during exercise at selected stages of pregnancy. 1992.
64. Bonomo M, Corica D, Mion E, Goncalves D, Motta G, Merati R, Ragusa A, Morabito A. Evaluating the therapeutic approach in pregnancies complicated by borderline glucose intolerance: a randomized clinical trial. Diabet Med 2005;22:1536–41.
65. Borberg C, Gillmer MD, Brunner EJ, Gunn PJ, Oakley NW, Beard RW. Obesity in pregnancy:the effect of dietary advice. Diabetes Care 1980;3:476–81.
66. Bosaeus M., Hussain A., Karlsson T., Andersson L., Hulthen L., Svelander C., Sandberg A.S., Larsson I., Ellegard L., Holmang A. A randomized longitudinal dietary intervention study during pregnancy: Effects on fish intake, phospholipids, and body composition. Nutrition. 2015;14:1. doi: 10.1186/1475-2891-14-1.
67. Botkin C, Driscoll CE. Maternal aerobic exercise: newborn effects. Fam Pract Res J. 1991;11(4):387-93.
68. Bowen A, Stewart N, Baetz M, Muhajarine N. Antenatal depression in socially high-risk women in Canada. Journal of Epidemiology & Community Health 2009;63(5):414-6.
69. Brand-Miller J. A pregnancy intervention to reduce postprandial glucose excursions in the primary prevention of paediatric obesity. Current Controlled Trials (www.controlled-trials.com/) [accessed 31 October 2010].
70. Brankston GN, Mitchell BF, Ryan EA, Okun NB. Resistance exercise decreases the need for insulin in overweight women with gestational diabetes mellitus. 2004.
71. Brekke HK, Bertz F, Rasmussen KM, Bosaeus I, Ellegard L, Winkvist A. Diet and exercise interventions among overweight and obese lactating women: Randomized trial of effects on cardiovascular risk factors. PloS One 2014;9(2):e88250.
72. Brenner IK, Wolfe LA, Monga M, McGrath MJ. Physical conditioning effects on fetal heart rate responses to graded maternal exercise. Medicine & Science in Sports & Exercise. 1999;31(6):792-9.
73. Briley A, Seed P, Singh C, Patel N, Poston L. Gestational weight gain in obese pregnant women, the impact of a lifestyle intervention and implications for guidelines (UPBEAT trial). BJOG: an international journal of obstetrics and gynaecology 2016;123(Suppl 1):55–6.?
74. Briley A, Seed PT, Singh C, Patel N, Poston L. Gestational weight gain, BMI and pregnancy outcomes in obese pregnant women in the UPBEAT behavioural intervention RCT. Reproductive Sciences 2016;23(1 Suppl 1):277A.
75. Briley AL, Barr S, Badger S, Bell R, Croker H, Godfrey KM, et al. A complex intervention to improve pregnancy outcome in obese women; the UPBEAT randomised controlled trial. BMC Pregnancy and Childbirth 2014;14 (1):74.
76. Briley C., Flanagan N.L. & Lewis N. (2002) In-home prenatal nutrition intervention increased dietary iron intakes and reduced low birthweight in low-income African-American women. Journal of the American Dietetic Association 102 (7), 984–987.
77. Brink HSM, Alkemade AJ, van der Lely J, van der L. Metformin in women at high risk of gestational diabetes mellitus Introduction. Diabetes Metab 2018: 8–10.
78. Broekhuizen K, Althuizen E, van Poppel MNM, Donker M, van Mechelen W. From theory to practice: intervention fidelity in a randomized controlled trial aiming to optimize weight development during pregnancy. Health Promotion Practice 2012;13(6):816–25.
79. Brownfoot F., Davey M., Kornman L., Brownfoot F. Routine weighing to reduce excessive antenatal weight gain: A randomised controlled trial. BJOG. 2016;123:254–261. doi: 10.1111/1471-0528.13735.
80. Bruno, R., Petrella, E., Bertarini, V., Pedrielli, G., Neri, I., and Facchinetti, F. (2017) Adherence to a lifestyle programme in overweight/obese pregnant women and effect on gestational diabetes mellitus: a randomized controlled trial. Maternal & Child Nutrition, 13: e12333. doi: 10.1111/mcn.12333.
81. Bung P, Artal R, Khodiguian N, Kjos S. Exercise in gestational diabetes. An optional therapeutic approach? Diabetes 1991;40(suppl 2):182-5.
82. Bungum TJ, Peaslee DL, Jackson AW, Perez MA. Exercise during pregnancy and type of delivery in nulliparae. J Obstet Gynecol Neonatal Nurs 2000;29:258–64.
83. Byrne N M, Groves A M, McIntyre H D, Callaway L K; BAMBINO group.Changes in resting and walking energy expenditure and walking speed during pregnancy in obese women Am J Clin Nutr 20119403819–830. [PubMed] [Google Scholar]
84. Cahill A., Haire-Joshu D., Cade W., Stein R., Woolfolk C., Moley K., Mathur A., Schechtman K., Klein S. Weight control program and gestational weight gain in disadvantaged women with overweight or obesity: A randomized clinical trial. Obesity. 2018;26:485–491. doi: 10.1002/oby.22070.

Diet and/or physical activity unique publications

85. Callaway L, McIntyre D, Colditz P, Byrne N, Foxcroft K, O'Connor B. Exercise in obese pregnant women: a randomized study to assess feasibility. Hypertension in Pregnancy 2008;27(4):549. ∗
86. Callaway L. A randomized controlled trial using exercise to reduce gestational diabetes and other adverse maternal and neonatal outcomes in obese pregnant women - the pilot study. Australian Clinical Trials Registry (www.actr.org.au) (accessed 21 June 2007).
87. Callaway LK, Colditz PB, Byrne NM, et al. Prevention of gestational diabetes: feasibility issues for an exercise intervention in obese pregnant women. Diabetes Care 2010; 33: 1457–1459.
88. Callaway LK, McIntyre HD, Barrett HL, Foxcroft K, Tremellen A, Lingwood BE, et al. Probiotics for the prevention of gestational diabetes mellitus in overweight and obese women: Findings from the SPRING double-blind randomized controlled trial. Diabetes Care 2019;42:364–71.
89. Calvalcante SR, Cecatti JG, Pereira RI, Baciuk EP, Bernardo AL, Silveira C. Water aerobics II: maternal body composition and perinatal outcomes after a program for low risk pregnant women. Reprod Health 2009;6:1.
90. Campbell DM, MacGillivray I. The effect of a low calorie diet or a thiazide diuretic on the incidence of pre-eclampsia and on birth weight. Br J Obstet Gynaecol 1975;82:572–7.
91. Campbell DM. Dietary restriction in obesity and its effect on neonatal outcome. In Campbell DM, Gillmer MDG, editors. Nutrition in pregnancy. Proceedings of the 10th Study Group of the RCOG. London: 1983. pp. 243–50.
92. Carlo C, Iannotti G, Sparice S, Chiacchio MP, Greco E, Tommaselli GA, et al. The role of a personalized dietary intervention in managing gestational weight gain: A prospective, controlled study in a low-risk antenatal population. Arch Gynecol Obstet 2014;289(4):765–70.
93. Carpenter MW, Sady SP, Sady MA, Haydon BB, Coustah DR, Thompson PD. Effects of exercise training in midpregnancy: a randomized controlled trial. Presented at the 37th annual meeting of the Society for Gynecologic Investigation, St Louis. Missouri, March 21-24, 1990;Abstr.497, p. 345.
94. Carpenter RE, Emery SJ, Uzun O, Rassi D, Lewis MJ. Influence of antenatal physical exercise on heart rate variability and QT variability. Journal of Maternal-Fetal & Neonatal Medicine. 2017;30(1):79.
95. Carpenter RE, Emery SJ, Uzun O, Silva LAD, Lewis MJ. Influence of antenatal physical exercise on haemodynamics in pregnant women: A flexible randomisation approach. BMC Pregnancy and Childbirth. 2015;15:156.
96. Carral F, Ayala Mdel C, Fernández JJ, González C, Piñero A, García G, et al. Web-based telemedicine system is useful for monitoring glucose control in pregnant women with diabetes. Diabetes Technol Ther 2015 May;17(5):349-354. [doi: 10.1089/dia.2014.0223] [Medline: 25629547]
97. Casanueva E, Legarreta D, Diaz-Barriga M, Soberanis Y, Cardenas T, Iturriaga A, et al. Weight gain during pregnancy in adolescents: evaluation of a non-nutritional intervention. Rev Invest Clin 1994;46:157–61.
98. Cavalcante S., Cecatti J., Pereira R., Baciuk E., Bernardo A., Silveira C. Water aerobics II: Maternal body composition and perinatal outcomes after a program for low risk pregnant women. Reprod. Health. 2009;6:1. doi: 10.1186/1742-4755-6-1.
99. Cavalcante SR, Cecatti JG, Pereira RI, Baciuk EP, Bernardo AL, Silveira C. Water aerobics II: maternal body composition and perinatal outcomes after a program for low risk pregnant women. Reprod Health 2009;6:1.
100. Ceesay SM, Prentice AM, Cole TJ, Foord F, Weaver LT, Poskitt EM, Whitehead RG. Effects on birth weight and perinatal mortality of maternal dietary supplements in rural Gambia: 5 year randomised controlled trial. BMJ 1997;315:786–90.
101. Chan G.M., McElligott K., McNaught T. & Gill G. (2006) Effects of dietary calcium intervention on adolescent mothers and newborns: a randomized controlled trial. Obstetrics and Gynecology 108, 565–571.
102. Chasan-Taber L, Schmidt MD, Pekow P, Sternfeld B, Manson JE, Solomon CG, et al. Physical activity and gestational diabetes mellitus among Hispanic women. J Womens Health (Larchmt). 2008;17(6):999-1008.
103. Chasan-Taber L, Silveira M, Lynch KE, et al. Physical activity and gestational weight gain in Hispanic women. Obesity. 2014;22(3):909–18. doi:10.1002/oby.20549.
104. Chasan-Taber L, Silveira M, Pekow P, Braun B, Manson JE, Solomon CG, et al. Physical activity, sedentary behavior and risk of hypertensive disorders of pregnancy in Hispanic women. Hypertens Pregnancy. 2015;34(1):1-16.
105. Chávez VG, Sánchez, M. D. P V, Rasch JRK. Effect of pelvic floor exercise during pregnancy and puerperium on prevention of urinary stress incontinence. Ginecol Obstet Mex. 2004;72(628-636).
106. Chiarello CM, Falzone LA, McCaslin KE, Patel MN, Ulery KR. The effects of an exercise program on diastasis recti abdominis in pregnant women [corrected] [published erratum appears in J WOMENS HEALTH PHYS THER 2005;29(3):76]. 2005.
107. Chiswick C, Reynolds RM, Denison F, Drake AJ, Forbes S, Newby DE, et al. Effect of metformin on maternal and fetal outcomes in obese pregnant women (EMPOWaR): A randomised, double-blind, placebo-controlled trial. Lancet Diabetes Endocrinol 2015;3:778–86.
108. Choi J, Lee J, Vittinghoff E, Fukuoka Y. mHealth Physical Activity Intervention: a randomized pilot study in physically inactive pregnant women. Matern Child Health J 2016; 20: 1091–1101.
109. Chuntharapat S, Petpichetchian W, Hatthakit U. Yoga during pregnancy: effects on maternal comfort, labor pain and birth outcomes. Complement Ther Clin Pract. 2008;14(2):105-15.

Diet and/or physical activity unique publications

| 110. Claesson I, Sydsjö G, Brynhildsen J, Blomberg M, Jeppsson A, Sydsjö A, Josefsson A. Weight after childbirth: A 2year follow-up of obese women in a weight-gain restriction program. Acta Obstetricia et Gynecologica Scandinavica. 2011;90(1):103–110. [PubMed] |
| 111. Claesson I-M, Brynhildsen J, Cedergren M, Jeppsson A, Sydsjo A, Josefsson A. Weight gain restriction during pregnancy is safe for both the mother and neonate. Acta Obstet Gynecol Scand 2009;88:1158–62. |
| 112. Claesson IM, Klein S, Sydsjo G, Josefsson A. Physical activity and psychological well-being in obese pregnant and postpartum women attending a weight-gain restriction programme. Midwifery. 2014;30(1):11. |
| 113. Claesson IM, Sydsjo G, Brynhildsen J et al. Weight gain restriction for obese pregnant women: a case-control intervention study. Br J Obstet Gynaecol 2008; 115: 44-50. |
| 114. Clapp III JF. Maternal carbohydrate intake and pregnancy outcome. Proceedings of the Nutrition Society 2002; 61(1):45–50. |
| 115. Clapp J., Kim H., Burciu B., Lopez B. Beginning regular exercise in early pregnancy: Effect on fetoplacental growth. Am. J. Obstet. Gynecol. 2000;183:1484–1488. doi: 10.1067/mob.2000.107096. |
| 116. Clapp J., Kim H., Burciu B., Schmidt S., Petry K., Lopez B. Continuing regular exercise during pregnancy: Effect of exercise volume on fetoplacental growth. Am. J. Obstet. Gynecol. 2002;186:142–147. doi: 10.1067/mob.2002.119109. |
| 117. Clapp JF (1997) Diet, exercise, and feto-placental growth. Arch Gynecol Obstet 260:101–108 |
| 118. Clapp JF III, Capeless EL. Neonatal morphometrics after endurance exercise during pregnancy. Am J Obstet Gynecol 1990;163:1805–11. |
| 119. Clapp JF III, Dickstein S. Endurance exercise and pregnancy outcome. Med Sci Sports Exerc 1984;16:556–62. |
| 120. Clapp JF III. Effect of dietary carbohydrate on the glucose and insulin response to mixed caloric intake and exercise in both nonpregnant and pregnant women. Diabetes Care 1998;21(Suppl 2):B107–B112. Clapp JF III. Maternal carbohydrate intake and pregnancy outcome. Proceedings of the Nutrition Society 2002;61: 45–50 |
| 121. Clapp JF III. The course of labor after endurance exercise during pregnancy. Am J Obstet Gynecol 1990;163:1799–805. |
| 122. Clapp JF, 3rd. Long-term outcome after exercising throughout pregnancy: fitness and cardiovascular risk. Am J Obstet Gynecol. 2008;199(5):489 e1-6. |
| 123. Clapp JF, Little KD. Effect of recreational exercise on pregnancy weight gain and subcutaneous fat deposition. Med Sci Sports Exerc 1995;27:170–7. |
| 124. Clapp JF, Wesley M, Sleamaker RH. Thermoregulatory and metabolic responses to jogging prior to and during pregnancy. Medicine & Science in Sports & Exercise. 1987;19(2):124???30. |
| 125. Clapp JF. Influence of endurance exercise and diet on human placental development and fetal growth. Placenta 2006;27(6-7):527–34 |
| 126. Clapp JF. The changing thermal response to endurance exercise during pregnancy. American Journal of Obstetrics and Gynecology. 1991;165(6):1684-9. |
| 127. Cogswell ME, Scanlon KS, Fein SB, Schieve LA. Medically advised, mother's personal target, and actual weight gain during pregnancy. Obstet Gynecol 1999;94:616–22. |
| 128. Colleran HL, Lovelady CA. Use of MyPyramid Menu Planner for Moms in a weight-loss intervention during lactation. J Acad Nutr Diet 2012;112(4):553–8. 66. Colleran HL, Wideman L, Lovelady CA. Effects of energy restriction and exercise on bone mineral density during lactation. Med Sci Sport Ex 2012;44(8):1570–9 |
| 129. Collings CA, Curet LB, Mullin JP. Maternal and fetal responses to a maternal aerobic exercise program. Am J Obstet Gynecol 1983;145: 702–7. |
| 130. Conway R, Reddy S, Davies J. Dietary restraint and weight gain during pregnancy. Eur J Clin Nutr 1999;53:849–53. |
| 131. Corcoy R, Mendoza LC, Simmons D, Desoye G, Adelantado JM, Chico A, et al. The DALI vitamin D randomized controlled trial for gestational diabetes mellitus prevention: No major benefit shown besides vitamin D sufficiency. Clin Nutr 2019:1–9. |
| 132. Cordero FY, Mottola FM, Vargas FJ, Blanco FM, Barakat FR. Exercise is associated with a reduction in gestational diabetes mellitus. Med Sci Sports Exerc. 2015;47(7):1328–33. https://doi.org/10.1249/MSS.0000000000000547. |
| 133. Cordero Y, Peláez M, De Miguel M, Perales M, Barakat R. Can moderate physical exercise during pregnancy act as a factor in preventing Gestational Diabetes? Revista Internacional de Ciencias del Deporte. 2012;27(8):3-19. |
| 134. Costa DD, Rippen N, Dritsa M, Ring A. Self-reported leisure-time physical activity during pregnancy and relationship to psychological well-being. J Psychosom Obstet Gynaecol. 2003;24(2):111-9. |
| 135. Courville AB, Harel O, Lammi-Keefe C. Consumption of a DHAcontaining functional food during pregnancy is associated with lower infant ponderal index and cord plasma insulin concentration. Br J Nutr 2011;106:208–12. |
| 136. Cowett RM, Carpenter MW, Carr S, et al. Glucose and lactate kinetics during a short exercise bout in pregnancy. 1996. |
| 137. Craigie AM, Macleod M, Barton KL, Treweek S, Anderson AS. Supporting postpartum weight loss in women living in deprived communities: design implications for a randomised control trial. Eur J Clin Nutr 2011;65(8):952–8. |
| 138. Cramp CS, Moran LJ, Deussen AR, Yelland LN, Dodd JM. Evaluation of printed nutrition education material in overweight and obese women during pregnancy-findings from the LIMIT randomised trial. Journal of Paediatrics and Child Health 2013;49 Suppl 2:118.? |
| 139. Crowther CA, Hiller JE, Moss JR, McPhee AJ, Jeffries WS, Robinson JS. Effect of treatment of gestational diabetes mellitus on pregnancy outcomes. N Engl J Med 2005;352:2477–86. |
| 140. Currie LM, Woolcott CG, Fell DB, Armson BA, Dodds L. The association between physical activity and maternal and neonatal outcomes: a prospective cohort. Matern Child Health J. 2014;18(8):1823-30. |

Diet and/or physical activity unique publications

141. D'Almeida A, Carter JP, Anatol A, Prost C. Effects of a combination of evening primrose oil (gamma linolenic acid) and fish oil (eicosapentaenoic + docahexaenoic acid) versus magnesium, and versus placebo in preventing preeclampsia. Women Health. 1992;19:117–31.
142. da Silva S., Hallal P., Domingues M., Bertoldi A., Silveira M., Bassani D., da Silva I.C., da Sliva B.C., Coll C.D., Evenson K. A randomized controlled trial of exercise during pregnancy on maternal and neonatal outcomes: Results from the PAMELA study. Int. J. Behav. Nutr. Phys. Act. 2017;14 doi: 10.1186/s12966-017-0632-6.
143. Dale E, Mullinax KM, Bryan DH. Exercise during pregnancy: effects on the fetus. Can J Appl Sport Sci 1982;7:98–103.
144. Daley A., Jolly K., Jebb S., Lewis A., Clifford S., Roalfe A., Kenyon S., Aveyard P. Feasibility and acceptability of regular weighing, setting weight gain limits and providing feedback by community midwives to prevent excess weight gain during pregnancy: Randomised controlled trial and qualitative study. BMC Obes. 2015;2:35. doi: 10.1186/s40608-015-0061-5.
145. Daly N, Farren M, McKeating A, O'Kelly R, Stapleton M, Turner MJ. A medically supervised pregnancy exercise intervention in obese women: A randomized controlled trial. Obstet Gynecol 2017;130:1001–10.
146. Daly N, Farren M, McKeating A, O'Higgins A, Mullaney L, Turner MJ. Effect of a randomized controlled trial of an intensive medically supervised exercise program designed to improve maternal glucose control on gestational weight gain. Am J Obstet Gynecol. 2017;216(1 Supplement 1):S24.
147. Daniel JA, Dikki CE, Ibrahim W. Aerobic dance exercise improves blood glucose level in pregnant women with gestational diabetes mellitus. 2014.
148. D'Anna R, Scilipoti A, Giordano D, Caruso C, Cannata ML, et al. (2013) Myo-inositol supplementation and onset of gestational diabetes mellitus in pregnant women with a family history of type 2 diabetes: A prospective, randomized, placebo-controlled study. Diabetes Care 36(4):854–7. doi: 10.2337/dc12-1371 PMID: 23340885.
149. Davenport MH, Girouox I, Sopper MM, Mottola MF. Postpartum Exercise Regardless of Intensity Improves Chronic Disease Risk Factors. Med Sci Sports Exerc 2011;43(6):951–8.
150. Davenport MH, Mottola MF, McManus R, Gratton R. A walking intervention improves capillary glucose control in women with gestational diabetes mellitus: a pilot study. Appl Physiol Nutr Metab. 2008;33(3):511-7.
151. Davis K, Goodman SH, Leiferman J, Taylor M, Dimidjian S. A randomized controlled trial of yoga for pregnant women with symptoms of depression and anxiety. Complement Ther Clin Pract. 2015;21(3):166-72.
152. de Barros MC, Lopes MA, Francisco RP, Sapienza AD, Zugaib M. Resistance exercise and glycemic control in women with gestational diabetes mellitus. Am J Obstet Gynecol. 2010;203(6):556 e1-6.
153. de Groot RHM, Hornstra G, van Houwelingen AC, Roumen F. Effect of alpha-linolenic acid supplementation during pregnancy on maternal and neonatal polyunsaturated fatty acid status and pregnancy outcome. Am J Clin Nutr 2004;79:251–60.
154. De Oliveria Melo AS, Silva JLP, Tavares JS, Barros VO, Leite DFB, Amorim MMR. Effect of a physical exercise program during pregnancy on uteroplacental and fetal blood flow and fetal growth: a randomized controlled trial. Obstet Gynecol. 2012;120(2 PART 1):302–10.
155. de Rooij SR, Painter RC, Holleman F, Bossuyt PM, Roseboom TJ. The metabolic syndrome in adults prenatally exposed to the Dutch famine. Am J Clin Nutr 2007;86:1219–24.
156. de Wit L, Jelsma JG, van Poppel MN, Bogaerts A, Simmons D, Desoye G, et al. Physical activity, depressed mood and pregnancy worries in European obese pregnant women: results from the DALI study. BMC Pregnancy Childbirth. 2015;15:158.
157. Dekker Nitert, M., Barrett, H.L., Denny, K.J., McIntyre, H.D., Callaway, L.K. and (2015), Exercise in pregnancy does not alter gestational weight gain, MCP-1 or leptin in obese women. Aust N Z J Obstet Gynaecol, 55: 27-33. doi:10.1111/ajo.12300
158. Demissie Z, Siega-Riz AM, Evenson KR, Herring AH, Dole N, Gaynes BN. Physical activity and depressive symptoms among pregnant women: the PIN3 study. Arch Womens Ment Health 2011;14(12):145-57.
159. Dempsey JC, Butler CL, Sorensen TK, Lee IM, Thompson ML, Miller RS, et al. A case– control study of maternal recreational physical activity and risk of gestational diabetes mellitus. Diabetes Res Clin Pract 2004;66:203–15.
160. Dempsey JC, Sorensen TK, Williams MA, Lee IM, Miller RS, Dashow EE, et al. Prospective study of gestational diabetes mellitus risk in relation to maternal recreational physical activity before and during pregnancy. Am J Epidemiol 2004;159:663–70.
161. Denda OS, Dalkiranis AX, Denda MS, et al. The effect of some dietary components on body weight and fat deposition in pregnant women in combination with exercise during pregnancy and postpartum period. Studies in Physical Culture and Tourism 2005;12.
162. Deveer R., Deveer M., Akbaba E., Engin-Üstün Y., Aydoğan P., Çelikkaya H., Danisman N., Mollamahmutoglu L. The effect of diet on pregnancy outcomes among pregnants with abnormal glucose challenge test. Eur. Rev. Med. Pharmacol. Sci. 2013;17:1258–1261.
163. Di Carlo C., Iannotti G., Sparice S., Chiacchio M., Greco E., Tommaselli G., Nappi C. The role of a personalized dietary intervention in managing gestational weight gain: A prospective, controlled study in a low-risk antenatal population. Arch. Gynecol. Obstet. 2014;289:765–770. doi: 10.1007/s00404-013-3054-y.
164. Dias LA, Driusso P, Aita DL, Quintana SM, Bo K, Ferreira CH. Effect of pelvic floor muscle training on labour and newborn outcomes: a randomized controlled trial. Rev Bras Fisioter. 2011;15(6):487-93.

Diet and/or physical activity unique publications

165. Dodd 2014 Limiting weight gain in overweight and obese women during pregnancy to improve health outcomes: a randomised trial. anzctr.org.au/Trial/ Registration/TrialReview.aspx?id=81642 (first received 12 October 2006).?
166. Dodd J, Grivell R, Owens J. Antenatal Dietary and Lifestyle Interventions for Women Who are Overweight or Obese: Outcomes from the LIMIT Randomized Trial. Current Nutrition Reports. 2014;3(4):392-9.
167. Dodd J. Obesity in pregnancy-the LIMIT randomised trial. Journal of Paediatrics and Child Health 2013;49 Suppl 2:4. Dodd JM. Dietary and lifestyle advice for pregnant women who are overweight or obese: the LIMIT randomized trial. Annals of Nutrition and Metabolism 2014;64(3-4):197–202.
168. Dodd J., Turnbull D., McPhee A., Deussen A., Grivell R., Yelland L., Crowther C.A., Wittert G., Owens J.A., Robinson J.S. Antenatal lifestyle advice for women who are overweight or obese: LIMIT randomised trial. BMJ. 2014;348:1285. doi: 10.1136/bmj.g1285.
169. Dodd JM, Cramp C, Sui Z, Yelland LN, Deussen AR, Grivell RM, et al. The effects of antenatal dietary and lifestyle advice for women who are overweight or obese on maternal diet and physical activity: the LIMIT randomised trial. BMC Medicine 2014;12(1):161.?
170. Dodd JM, Deussen AR, Mohamad I, Rifas-Shiman SL, Yelland LN, Louise J, et al. The effect of antenatal lifestyle advice for women who are overweight or obese on secondary measures of neonatal body composition: the LIMIT randomised trial. BJOG: an international journal of obstetrics and gynaecology 2016;123(2):244–53.
171. Dodd JM, Kannieappan LM, Grivell RM, Deussen AR, Moran LJ, Yelland LN, et al. Effects of an antenatal dietary intervention on maternal anthropometric measures in pregnant women with obesity. Obesity 2015;23(8):1555–62.
172. Dodd JM, Louise J, Deussen AR, Grivell RM, Dekker G, McPhee AJ, et al. Effect of metformin in addition to dietary and lifestyle advice for pregnant women who are overweight or obese: the GRoW randomised, double-blind, placebo controlled trial. Lancet Diabetes Endocrinol 2019;7:15–24.
173. Dodd JM, McPhee AJ, Turnbull D, Yelland LN, Deussen AR, Grivell RM, et al. The effects of antenatal dietary and lifestyle advice for women who are overweight or obese on neonatal health outcomes: the LIMIT randomised trial. BMC Medicine 2014;12(1):163.
174. Dodd JM, Newman A, Moran LJ, Deussen AR, Grivell RM, Yelland LN, et al. The effect of antenatal dietary and lifestyle advice for women who are overweight or obese on emotional well-being: the LIMIT randomized trial. Acta Obstet Gynecol Scand. 2016;95(3):309-18.
175. Dodd JM, O'Brien CM, Grivell RM. Modifying diet and physical activity to support pregnant women who are overweight or obese. Current Opinion in Clinical Nutrition and Metabolic Care 2015;18(3):318–23.
176. Dodd JM, Turnbull DA, McPhee AJ, Wittert G, Crowther CA, Robinson JS. Limiting weight gain in overweight and obese women during pregnancy to improve health outcomes: the LIMIT randomised controlled trial. BMC Pregnancy and Childbirth 2011;11:79.
177. Domingues MR, Barros AJ, Matijasevich A. Leisure time physical activity during pregnancy and preterm birth in Brazil. Int J Gynaecol Obstet. 2008;103:9–15.
178. Donnelly J, Horan M, Walsh J, McGowan C, Byrne J, Molloy EJ, et al. Impact of a low GI diet on neonatal body composition (ROLO Kids). Pediatric Academic Societies Annual Meeting; 2013 May 4-7; Washington DC, USA. 2013
179. Donnelly JM, Lindsay KL, Walsh JM, Horan M, Molloy EJ, McAuliffe FM. Fetal metabolic influences of neonatal anthropometry and adiposity. BMC Pediatrics 2015;15(1): 175
180. Donnelly JM, Walsh JM, Byrne J, Molloy E, McAuliffe FM. Altered neonatal anthropometric measurements following maternal low GI diet in pregnancy (ROLO study). Acta Obstetricia et Gynecologica Scandinavica 2013;92(s160):13
181. Donnelly JM, Walsh JM, Byrne J, Molloy EJ, McAuliffe FM. Impact of maternal diet on neonatal anthropometry: A randomized controlled trial. Pediatric Obesity 2015;10(1): 52–6
182. Donnelly JMT, Lindsay KL, Walsh JM, Horan M, Molloy EJ, McAuliffe F. Impact of maternal and fetal inflammatory markers on neonatal and infant adiposity. Archives of Disease in Childhood 2015;100:A133–4
183. Doustan M, Seifourian M, Zarghami M, et al. Relationship between physical activity of mothers before and during pregnancy with the newborn health and pregnancy outcome. J Phys Educ Sport. 2012;12(2):222–9.
184. Downs DS, DiNallo JM, Kirner TL. Determinants of pregnancy and postpartum depression: prospective influences of depressive symptoms, body image satisfaction, and exercise behavior. Ann Behav Med. 2008;36(1):54-63.
185. Downs DS, Hausenblas HA. Pregnant women's third trimester exercise behaviors, body mass index, and pregnancy outcomes. Psychol Health. 2007;22(5):545–59.
186. Dumas GA, Reid JG, Wolfe LA, et al. Exercise, posture, and back pain during pregnancy. Clin Biomech 1995;10:98–103.
187. Dumith SC, Domingues MR, Mendoza-Sassi RA, et al. Physical activity during pregnancy and its association with maternal and child health indicators. Rev Saude Publica 2012;46:327–33.
188. Duncombe D, Skouteris H, Wertheim EH, et al. Vigorous exercise and birth outcomes in a sample of recreational exercisers: a prospective study across pregnancy. Aust N Z J Obstet Gynaecol. 2006;46(4):288–92.
189. Dwarkanath P, Muthayya S, Vaz M, et al. The relationship between maternal physical activity during pregnancy and birth weight. Asia Pac J Clin Nutr. 2007;16(4):704–10.

Diet and/or physical activity unique publications

190. Dyck RF, Sheppard SM, Klomp H, Tan L, Chad K, van Vliet SH. Using exercise to prevent gestational diabetes among aboriginal women -- hypothesis and results of a pilot/feasibility project in Saskatchewan. Canadian Journal of Diabetes Care. 1999;23(3):32-8.
191. Dye TD, Knox KL, Artal R, Aubry RH, Wojtowycz MA. Physical activity, obesity, and diabetes in pregnancy. Am J Epidemiol 1997;146:961–5.
192. Eames AJ, Grivell RM, Dodd JM, Deussen A. The effect of limited gestational weight gain in overweight and obese
193. Eggen MH, Stuge B, Mowinckel P, Jensen KS, Hagen KB. Can supervised group exercises including ergonomic advice reduce the prevalence and severity of low back pain and pelvic girdle pain in pregnancy? A randomized controlled trial. Phys Ther. 2012;92(6):781-90.
194. Ehrlich SF, Hedderson MM, Feng J, Crites Y, Quesenberry CP, Ferrara A. Lifestyle intervention improves postpartum fasting glucose levels in women with gestational diabetes. Diabetes 2014; 63 (Suppl 1):A95.
195. El Beltagy N, El Deen SS, Mohamed R. Does physical activity and diet control reduce the risk of developing gestational diabetes mellitus in Egypt? A randomized controlled trial. Journal of Perinatal Medicine 2013;41 (Suppl 1):1176.
196. El Hiday MM, Zumrawi FY. The effect of a nutrition education programme on pregnant women attending Khartoum model clinic. Ahfad J 1992;9:23–36.
197. Elden H, Ostgaard HC, Fagevik-Olsen M, Ladfors L, Hagberg H. Treatments of pelvic girdle pain in pregnant women: adverse effects of standard treatment, acupuncture and stabilising exercises on the pregnancy, mother, delivery and the fetus/neonate. BMC Complement Altern Med. 2008;8:34.
198. El-Rafie M, Khafagy GM, Gamal MG. Effect of aerobic exercise during pregnancy on antenatal depression. International Journal of Women's Health. 2016;8:53.
199. Erkkola R, Makela M. Heart volume and physical fitness of parturients. Ann Clin Res 1976;8:15-21.
200. Erkkola R, Makela M. Heart volume and physical fitness of parturients. Ann Clin Res 1976;8:15–21.
201. Erkkola R, Mäkelä M. Heart volume and physical fitness of parturients. Ann Clin Res. 1976;8(1):15–21.
202. Erkkola R. The influence of physical exercise during pregnancy upon physical work capacity and circulatory parameters. Scand J Clin Lab Invest 1976;36:747–59.
203. Ersek JL, Brunner Huber LR. Physical activity prior to and during pregnancy and risk of postpartum depressive symptoms. J Obstet Gynecol Neonatal Nurs. 2009;38(5):556-66.
204. Evenson K, Barakat R, Brown W, et al. Guidelines for physical activity during pregnancy: comparisons from around the world. Am J Lifestyle Med. 2014;8(2):102–21.
205. Evenson KR, Siega-Riz AM, Savitz DA, et al. Vigorous leisure activity and pregnancy outcome. Epidemiology. 2002;13(6):653–9.
206. Facchinetti F. Pregnancy complications in women with BMI > 25kg/m2 enrolled in a healthy lifestyle and eating habits program. ClinicalTrials.gov (accessed 21 May 2013). NCT01783210 2013.
207. Fard NM, Mehrabian F, Sarraf-Zadegan N, Sajadi F. Fat-modified diets during pregnancy and lactation and serum lipids after birth. Indian J Pediatr 2004;71:683–7.
208. Feodor Nilsson S, Andersen PK, Strandberg-Larsen K, et al. Risk factors for miscarriage from a prevention perspective: a nationwide follow-up study. BJOG 2014;121:1375–85.
209. Ferland S, Bujold E, Giguere Y, Girard M, Demers S, Forest JC. Association between physical activity in early pregnancy and markers of placental growth and function. J Obstet Gynaecol Can. 2013;35(9):787-92.
210. Ferrara A. Diet, exercise and breastfeeding intervention program for women with gestational diabetes (DEBI Trial). ClinicalTrials.gov(www.clinicaltrials.gov) [accessed 31 October 2010].
211. Ferrara A, Hedderson MM, Albright CL, Ehrlich SF, Quesenberry CP, Jr., Peng T, et al. A pregnancy and postpartum lifestyle intervention in women with gestational diabetes mellitus reduces diabetes risk factors: a feasibility randomized control trial. Diabetes Care. 2011;34(7):1519-25.
212. Ferrara A, Hedderson MM, Brown SD, Albright CL, Ehrlich SF, Tsai AL, et al. The Comparative Effectiveness of Diabetes Prevention Strategies to Reduce Postpartum Weight Retention in Women With Gestational Diabetes Mellitus: The Gestational Diabetes' Effects on Moms (GEM) Cluster Randomized Controlled Trial. Diabetes Care. 2016;39(1):65-74.
213. Field T, Diego M, Delgado J, Medina L. Tai chi/yoga reduces prenatal depression, anxiety and sleep disturbances. Complement Ther Clin Pract. 2013;19(1):6-10.
214. Field T, Diego M, Delgado J, Medina L. Yoga and social support reduce prenatal depression, anxiety and cortisol. J Bodyw Mov Ther. 2013;17(4):397-403.
215. Field T, Diego M, Hernandez-Reif M, Medina L, Delgado J, Hernandez A. NIHMS326920 PMC3319349; Yoga and massage therapy reduce prenatal depression and prematurity. Journal of Bodywork & Movement Therapies. 2012;16(2):204.
216. Fjeldsoe BS, Miller YD, Marshall AL. MobileMums: a randomized controlled trial of an SMS-based physical activity intervention. Ann Behav Med 2010; 39: 101–111.
217. Fleten C, Stigum H, Magnus P, et al. Exercise during pregnancy,maternal prepregnancy body mass index, and birth weight.Obstet Gynecol. 2010;115(2 Pt 1):331–7. doi:10.1097/AOG.0b013e3181ca4414.
218. Fortner RT, Pekow PS, Whitcomb BW, et al. Physical activity and hypertensive disorders of pregnancy among Hispanic women. Med Sci Sports Exerc. 2011;43(4):639–46. doi:10.1249/MSS.0b013e3181f58d3e.
219. Foxcroft KF, Rowlands IJ, Byrne NM, McIntyre HD, Callaway LK, for the BAMBINO group. Exercise in obese pregnant women: the role of social factors, lifestyle and pregnancy symptoms. BMC Pregnancy and Childbirth 2011;11:4.
220. Fraser RB, Ford FA, Milner RD. A controlled trial of a high dietary fibre intake in pregnancy—Effects on plasma glucose and insulin levels. Diabetologia 1983;25:238–241.

Diet and/or physical activity unique publications

221. Fraser RB. High fibre diets in pregnancy. Nutrition in Pregnancy. Proceedings of 10th Study Group of the Royal College of Obstetricians and Gynaecologists; 1982 September. London: RCOG, 1983:269–80
222. Fritel X, de Tayrac R, Bader G, Savary D, Gueye A, Deffieux X, et al. Preventing Urinary Incontinence With Supervised Prenatal Pelvic Floor Exercises: A Randomized Controlled Trial. Obstet Gynecol. 2015;126(2):370-7.
223. Garnæs K K, Mørkved S, Salvesen Ø, Moholdt T. Exercise training and weight gain in obese pregnant women: a randomized controlled trial (ETIP trial) PLoS Med. 2016;13(07):e1002079.
224. Garnaes KK, Morkved S, Salvesen O, Moholdt T. Exercise Med 2016;13(7): e1002079.48.
225. Garnaes KK, Nyrnes SA, Salvesen KA, Salvesen O, Morkved S, Moholdt T. Effect of supervised exercise training during pregnancy on neonatal and maternal outcomes among overweight and obese women. Secondary analyses of the ETIP trial: a randomised controlled trial. PloS One. 2017;12:e0173937.
226. Garshasbi A, Faghih Zadeh S. The effect of exercise on the intensity of low back pain in pregnant women. Int J Gynaecol Obstet 2005;88:271–5.
227. Gaston A, Prapavessis H. Tired, moody and pregnant? Exercise may be the answer. Psychol Health. 2013;28(12):1353-69.
228. Gesell S., Katula J., Strickland C., Vitolins M. Feasibility and initial efficacy evaluation of a community-based cognitive-behavioral lifestyle intervention to prevent excessive weight gain during pregnancy in Latina women. Matern. Child Health J. 2015;19:1842–1852. doi: 10.1007/s10995-015-1698-x.
229. Ghodsi Z, Asltoghiri M. Effects of aerobic exercise training on maternal and neonatal outcome: a randomized controlled trial on pregnant women in Iran. J Pak Med Assoc 2014;64:1053.
230. Giroux I, Inglis SD, Lander S, Gerrie S, Mottola MF. Dietary intake, weight gain, and birth outcomes of physically active pregnant women: a pilot study. Appl Physiol Nutr Metab. 2006;31(5):483-9.
231. Gjestland K, Bo K, Owe KM, Eberhard-Gran M. Do pregnant women follow exercise guidelines? Prevalence data among 3482 women, and prediction of low-back pain, pelvic girdle pain and depression. Brit J Sport Med. 2013;47(8):515.
232. Goletzke J, Buyken AE, Louie JC, Moses RG, BrandMiller JC. Dietary micronutrient intake during pregnancy is a function of carbohydrate quality. American Journal of Clinical Nutrition 2015;102(3):626–32
233. Gollenberg AL, Pekow P, Bertone-Johnson ER, et al. Physical activity and risk of small-for-gestational-age birth among predominantly Puerto Rican women. Matern Child Health J. 2011;15(1):49–59. doi:10.1007/s10995-009-0563-1.
234. Gomez TG, Delgado JG, Agudelo AA, Hurtado H. Diet effects on the perinatal result of obese pregnant patient. [Spanish]. Rev Colomb Obstet Ginecol 1994;45:313-6.
235. Goodwin A, Astbury J, McMeeken J. Body image and psychological well-being in pregnancy. A comparison of exercisers and non-exercisers. Aust N Z J Obstet Gynaecol. 2000;40(4):442-7.
236. Grant SM, Wolever TMS, O'Connor DL, Nisenbaum R, Josse RG (2011) Effect of a low glycaemic index diet on blood glucose in women with gestational hyperglycaemia. Diabetes Res Clin Pract 91:15–22
237. Gray-Donald K, Robinson E, Collier A, David K, Renaud L, Rodrigues S. Intervening to reduce weight gain in pregnancy and gestational diabetes mellitus in Cree communities: an evaluation. Can Med Assoc J 2000;163(10):1247–51.
238. Gregory PB, Rush D. Iatrogenic caloric restriction in pregnancy and birthweight. Am J Perinatol 1987;4:365–71.
239. Grivell R, Yelland L, Earl RA, Staehr CJ, Dodd J. The effect of antenatal dietary and lifestyle advice on fetal body composition in women who are overweight or obese: findings from the LIMIT randomised trial. Ultrasound in Obstetrics and Gynecology 2013;42(Suppl 1):10.
240. Grivell RM, Yelland LN, Deussen A, Crowther CA, Dodd JM. Antenatal dietary and lifestyle advice for women who are overweight or obese and the effect on fetal growth and adiposity: the LIMIT randomised trial. BJOG: an international journal of obstetrics & gynaecology 2016;123 (2):233–43.
241. Guelfi KJ, Ong MJ, Crisp NA, Fournier PA, Wallman KE, Grove JR, et al. Regular Exercise to Prevent the Recurrence of Gestational Diabetes Mellitus: A Randomized Controlled Trial. Obstetrics & Gynecology. 2016;128(4):819-27.
242. Guelinckx I, Devlieger R, Mullie P, et al. Effect of lifestyle intervention on dietary habits, physical activity, and gestational weight gain in obese pregnant women: a randomized controlled trial. Am J Clin Nutr 2010; 91: 373–80.
243. Gustafsson MK, Stafne SN, Romundstad PR, Morkved S, Salvesen K, Helvik AS. The effects of an exercise programme during pregnancy on health-related quality of life in pregnant women: a Norwegian randomised controlled trial. BJOG. 2016;123(7):1152-60.
244. Guszkowska M, Langwald M, Sempolska K. Does physical exercise help maintain mental health during pregnancy? A comparison of changes in mental health in participants of physical exercise classes and childbirth classes. Journal of Physical Activity & Health. 2015;12(1):30.
245. Guszkowska M, Sempolska K, Zaremba A, Langwald M. Exercise or relaxation? Which is more effective in improving the emotional state of pregnant women? 2013.
246. Guszkowska M. PHYSICAL ACTIVITY IN RELATION TO AFFECTIVE STATES AND LABOR ANXIETY IN PREGNANT WOMEN. Med Sport. 2011;15(3):114-8.
247. Haakstad L, Bo K. Exercise during pregnancy-Does it impact offspring birth weight parameters?. Journal of Science and Medicine in Sport 2012;15(Suppl 1):S340.
248. Haakstad L A, Bø K. Effect of regular exercise on prevention of excessive weight gain in pregnancy: a randomised controlled trial. Eur J Contracept Reprod Health Care. 2011;16(02):116–125. [PubMed] [Google Scholar]

Diet and/or physical activity unique publications

249. Haakstad L, Bo K. Effect of supervised aerobic dance exercise in prevention of excessive weight gain in pregnancy: a single blind randomised controlled trial. Int J Gynaecol Obstet 2009;107:S198.
250. Haakstad L, Bo K. Exercise in pregnant women and birth weight: a randomized controlled trial. BMC Preg Childbirth 2011;11:66.
251. Haakstad LA, Torset B, Bo K. What is the effect of regular group exercise on maternal psychological outcomes and common pregnancy complaints? An assessor blinded RCT. Midwifery. 2016;32:81-6.
252. Haakstad LA, Voldner N, Henriksen T, Bo K. Physical activity level and weight gain in a cohort of pregnant Norwegian women. Acta Obstetricia et Gynecologica Scandinavica. 2007;86:559–564. [PubMed]
253. Haby K, Glantz A, Hanas R, et al. Mighty mums - an antenatal health care intervention can reduce gestational weight gain in women with obesity. Midwifery 2015;31:685–92.
254. Haby K., Berg M., Gyllensten H., Hanas R., Premberg A. Mighty Mums—A lifestyle intervention at primary care level reduces gestational weight gain in women with obesity. BMC Obes. 2018;5:16. doi: 10.1186/s40608-018-0194-4.
255. Haelterman E, Marcoux S, Croteau A, Dramaix M. Population-based study on occupational risk factors for preeclampsia and gestational hypertension. Scand J Work Environ Health. 2007;33(4):304-17.
256. Hagobian TA, Phelan S, Gorin AA, Phipps MG, Abrams B, Wing RR. Effects of maternal lifestyle intervention during pregnancy on untreated partner weight: results from fit for delivery study. Obesity 2016;24(1):23–5.?NCT01117961. Promoting healthy weight gain during pregnancy. clinicaltrials.gov/ct2/show/NCT01117961 (first received 15 April 2010).?
257. Hall DC, Kaufmann DA. Effects of aerobic and strength conditioning on pregnancy outcomes. Am J Obstet Gynecol 1987;157:1199–203.
258. Halse RE, Wallman KE, Dimmock JA, Newnham JP, Guelfi KJ. Home-Based Exercise Improves Fitness and Exercise Attitude and Intention in Women with GDM. Med Sci Sports Exerc. 2015;47(8):1698-704.
259. Halse RE, Wallman KE, Newnham JP, Guelfi KJ. Pregnant women exercise at a higher intensity during 30 min of self-paced cycling compared with walking during late gestation: implications for 2 h postprandial glucose levels. 2013.
260. Hanan EM, Adly Ali S, Mohamed Mostafa R. Effect of Antenatal Exercises on Umbilical Blood Flow and Neonate Wellbeing in Diabetic Pregnant Women. Indian Journal of Physiotherapy & Occupational Therapy. 2012.
261. Harden SM, Beauchamp MR, Pitts BH et al. Group-based life-style sessions for gestational weight gain management: a mixed method approach. Am J Health Behav 2014;38(4): 560–569.39.
262. Harizopoulou VC, Kritikos A, Papanikolaou Z, Saranti E, Vavilis D, Klonos E, et al. Maternal physical activity before and during early pregnancy as a risk factor for gestational diabetes mellitus. Acta Diabetol. 2010;47 Suppl 1:83-9.
263. Harris ST, Liu J, Wilcox S, Moran R, Gallagher A. Exercise during pregnancy and its association with gestational weight gain. Matern Child Health J. 2015;19(3):528-37.
264. Harrison 2013 ACTRN12608000233325. Healthier lifestyles: preventing gestational diabetes in high risk pregnancies: a research and education project. anzctr.org.au/Trial/Registration/TrialReview.aspx?ACTRN=12608000233325 (first received 5 May 2008).?
265. Harrison C., Lombard C., Strauss B., Teede H. Optimizing healthy gestational weight gain in women at high risk of gestational diabetes: A randomized controlled trial. Obesity. 2013;21:904–909. doi: 10.1002/oby.20163.
266. Harrison CL, Lombard CB, Gibson-Helm M, Deeks A, Teede HJ (2011) Limiting excess weight gain in high-risk pregnancies: A randomized controlled trial. Endocr Rev 32(3). doi: 10.1210/er.2011-0001 PMID: 21441345.
267. Harrison CL, Teede HJ, Lombard CB. How effective is self- weighing in the setting of a lifestyle intervention to reduce gestational weight gain and postpartum weight retention??. Australian and New Zealand Journal of Obstetrics and Gynaecology 2014;54:382–5.?
268. Harrod CS, Chasan-Taber L, Reynolds RM, et al. Physical activity in pregnancy and neonatal body composition: the healthy start study. Obstet Gynecol. 2014;124(2 Pt 1):257–64. doi:10.1097/AOG.0000000000000373.
269. Hatch M, Levin B. Maternal leisure-time exercise and timely delivery. Am J Public Health. 1998;88(10):1528–33.
270. Hatch MC, Shu XO, McLean DE, et al. Maternal exercise during pregnancy, physical fitness, and fetal growth. Am J Epidemiol.1993;137(10):1105–14.
271. Hauth JC, Gilstrap LC, Widmer K. Fetal heart rate reactivity before and after maternal jogging during the third trimester. American Journal of Obstetrics & Gynecology. 1982.
272. Hawkins M., Hosker M., Marcus B., Rosal M., Braun B., Stanek E., Markenson G., Chasan-Taber L. A pregnancy lifestyle intervention to prevent gestational diabetes risk factors in overweight Hispanic women: A feasibility randomized controlled trial. Diabet. Med. 2015;32:108–115. doi: 10.1111/dme.12601.
273. Hayes L, Bell R, Robson S, Poston L. Association between physical activity in obese pregnant women and pregnancy outcomes: The UPBEAT pilot study. Annals of Nutrition and Metabolism 2014;64(3-4):239–46.?
274. Hayes L, Bell R, Robson S, Poston L. UPBEAT study: Association between physical activity in obese pregnant women and health of the offspring. The Power of Programming 2014: International Conference on Developmental Origins of Adiposity and Long-Term Health; 2014 March 13-15; Munich, Germany. 2014:48. ISRCTN89971375. UK Pregnancies Better Eating and Activity Trial. isrctn.com/ISRCTN89971375 (first received 23 July 2008).

Diet and/or physical activity unique publications

275. Hayes L, McParlin C, Kinnunen TI, Poston L, Robson SC, Bell R. Change in level of physical activity during pregnancy in obese women: findings from the UPBEAT pilot trial. BMC Pregnancy and Childbirth 2015;15:52.?
276. Hegaard HK, Hedegaard M, Damm P, et al. Leisure time physical activity is associated with a reduced risk of preterm delivery. Am J Obstet Gynecol. 2008;198(2):180.e1–5. doi:10.1016/j.ajog.2007.08.038.
277. Hegaard HK, Petersson K, Hedegaard M, et al. Sports and leisure- time physical activity in pregnancy and birth weight: a population-based study. Scand J Med Sci Sports. 2010;20(1): e96–102. doi:10.1111/j.1600-0838.2009.00918.x.
278. Hellenes OM, Vik T, Løhaugen GC, Salvesen K, Stafne SN, Mørkved S, et al. Regular moderate exercise during pregnancy does not have an adverse effect on the neurodevelopment of the child. Acta paediatrica (Oslo, Norway : 1992). 2015; https://doi.org/10.1111/apa.12890.
279. Herrera-Perdigon J., Hopkins E., Marcalle M., Brooten D., Youngblut J., Lizardo M. Weight gain in high-risk pregnant women: Comparison by primary diagnosis and type of care. Clin. Excell. Nurse Pract. 2005;9:195–201.
280. Herring SJ, Cruice J., Bennett G., Rose M., Davey A., Foster G. Preventing excessive gestational weight gain among African American women: A randomized clinical trial. Obesity. 2016;24:30–36. doi: 10.1002/oby.21240.
281. Herring SJ, Cruice JF, Bennett GG, Davey A, Foster GD. Using technology to promote postpartum weight loss in urban, low income mothers: a pilot randomized controlled trial. J Nutr Educ Behav 2014; 46: 610–615.
282. Hillesund ER, Bere E, Sagedal LR, Vistad I, Overby NC. Effect of a diet intervention during pregnancy on dietary behavior in the randomized controlled Norwegian Fit for Delivery study. Journal of Developmental Origins of Health and Disease 2016;7(5):538–47.?NCT01001689. Fit for delivery: a study of the effect of exercise sessions and nutritional counselling on pregnancy outcome (FFF). clinicaltrials.gov/ct2/show/NCT01001689 (first received 26 October 2009).?
283. Hofman P, Hopkins S. Randomised controlled study of the effects of exercise during pregnancy on maternal insulin sensitivity and neonatal outcomes. Australian Clinical Trials Register (http://www.actr.org/actr) (accessed 6 December 2005).
284. Hoirisch-Clapauch 2016 {published and unpublished data} Hoirisch-Clapauch S, Sant'Anna MCW, Moreira ECC, Frankel PP, Valle MP, D'Ippolito MM. A protocol combining daily walking and a low glycemic index diet increases the rate of take-home babies in women with consecutive first-trimester miscarriages. BJOG: an international journal of obstetrics and gynaecology 2016;123 (Suppl 2):74–5.
285. Hollingsworth DR, Moore TR. Postprandial walking exercise in pregnant insulin dependent (type I) diabetic women: reduction of plasma lipid levels but absence of a significant effect on glycemic control. Am J Obstet Gynecol 1987;157:1359–63.
286. Hopkins S, Baldi J, Cutfield W, et al. Effects of exercise training on maternal hormonal changes in pregnancy. Clin Endocrinol (Oxf) 2011;74:495–500.
287. Hopkins SA, Baldi JC, Cutfield WS, McCowan L, Hofman PL. Exercise training in pregnancy reduces offspring size without changes in maternal insulin sensitivity. J Clin Endocrinol Metab 2010;95: 2080–8.
288. Hoppu U, Isolauri E, Koskinen P, Laitinen K. Diet and blood lipids in 1-4 year-old children. Nutrition Metabolism and Cardiovascular Diseases 2013;23(10):980–6
289. Hoppu U, Isolauri E, Laakso P, Matomaki J, Laitinen K. Probiotics and dietary counselling targeting maternal dietary fat intake modifies breast milk fatty acids and cytokines. European Journal of Nutrition 2012;51(2):211–9
290. Horan M K, McGowan C A, Gibney E R, Byrne J, Donnelly J M, McAuliffe F M. Maternal nutrition and glycaemic index during pregnancy impacts on offspring adiposity at 6 months of age analysis from the ROLO randomised controlled trial. Nutrients. 2016;8(01):13–15. [PMC free article] [PubMed] [Google Scholar]
291. Horan M, Donnelly J, McGowan C, Gibney E, McAuliffe F. The association between maternal nutrition and lifestyle during pregnancy and 2-year-old offspring adiposity: analysis from the ROLO study. J Public Health. 2016;24(5):427-436.
292. Horan M, McGowan C, Donnelly J, Gibney E, McAuliffe F. Maternal diet and weight at 3 months partum following a pregnancy intervention with a low glycaemic index diet: Results from the ROLO randomised control trial. Archives of Disease in Childhood. Fetal and Neonatal Edition 2014;99 (Suppl 1):A129-A130, Abstract no: PMM.20
293. Horan MK, McGowan CA, Doyle O, McAuliffe FM. Well-being in pregnancy: An examination of the effect of socioeconomic, dietary and lifestyle factors including impact of a low glycaemic index dietary intervention. European Journal of Clinical Nutrition 2014;68(1):19–24
294. Horan MK, McGowan CA, Gibney ER, Donnelly JM, McAuliffe FM. Maternal low glycaemic index diet, fat intake and postprandial glucose influences neonatal adiposity - secondary analysis from the ROLO study. Nutrition Journal 2014;13(1):78. ISRCTN54392969. A randomised controlled trial of low glycaemic index carbohydrate diet versus no dietary intervention in the prevention of recurrence of foetal macrosomia. controlled-trials.com/ISRCTN54392969 Date first received: 10 August 2009
295. Horns PN, Ratcliffe LP, Leggett JC, Swanson MS. Pregnancy outcomes among active and sedentary primiparous women. J Obstet Gynecol Neonatal Nurs 1996;25:49–54.
296. Huang T T, Yeh C Y, Tsai Y C. A diet and physical activity intervention for preventing weight retention among Taiwanese childbearing women: a randomised controlled trial. Midwifery. 2011;27(02):257–264. [PubMed] [Google Scholar]

Diet and/or physical activity unique publications

297. Hui A L, Back L, Ludwig S et al.Effects of lifestyle intervention on dietary intake, physical activity level, and gestational weight gain in pregnant women with different pre-pregnancy body mass index in a randomized control trial. BMC Pregnancy Childbirth. 2014;14:331. [PMC free article] [PubMed] [Google Scholar]
298. Hui A, Back L, Ludwig S et al. Lifestyle intervention on diet and exercise reduced excessive gestational weight gain in pregnant women under a randomised controlled trial. Br J Obstet Gynaecol 2012; 119: 70-77.
299. Hui A, Back L, Ludwig S, Gardiner P, Sevenhuysen G, Dean H, et al. Exercise and dietary intervention increases physical activity, promotes healthy diet and reduces excessive gestational weight gain in pregnant women: A randomized controlled trial in Urban community. Diabetes 2011;60: A351.?
300. Hui AL, Back L, Reid A, Sevenhuysen G, Ludwig S, Dean H, et al. Effects of physical activity and dietary intakes on weight gain of pregnant women with normal and above normal prepregnancy weight. Canadian Journal of Diabetes 2012;36(5 Suppl 1):S8.?
301. Hui AL, Ludwig S, Gardiner P, et al. Community-based exercise and dietary intervention during pregnancy: a pilot study. Can J Diabetes 2006;30:1–7.
302. Hui AL, Ludwig S, Gardiner P, Sevenhuysen G, Dean H, Sellers E, et al. Exercise and dietary intervention during pregnancy results in reduced excessive gestational weight gain. Diabetes 2010;59(Suppl 1):A509.?
303. Huseinovic E, Winkvist A, Bertz F, Berte´us Forslund H, Brekke HK. Eating frequency, energy intake and body weight during a successful weight loss trial in overweight and obese postpartum women. Eur J Clin Nut 2014;68(1):71–6.
304. Ickovics JR, Earnshaw V, Lewis JB, et al. Cluster randomized controlled trial of group prenatal care: perinatal outcomes among adolescents in New York City health centers. Am J Public Health. 2016;106(2):359-365.
305. Ilmonen J, Isolauri E, Poussa T, Laitinen K (2011) Impact of dietary counselling and probiotic intervention on maternal anthropometric measurements during and after pregnancy: A randomized placebo-controlled trial. Clinical Nutrition. 2011; 30(2): 156–164.
306. International Association of Diabetes and Pregnancy Study Groups Consensus Panel, Metzger BE, Gabbe SG, Persson B, Buchanan TA et al. International Association of diabetes and Pregnancy Study Groups recommendations on the diagnosis and classification of hyperglycemia in pregnancy. Diabetes Care 2010;33: 676–82 PubMed PMID: 20190296. PubMed Central PMCID:2827530.
307. Iqbal R, Rafique G, Badruddin S, Qureshi R, Cue R, Gray-Donald K. Increased body fat percentage and physical inactivity are independent predictors of gestational diabetes mellitus in South Asian women. Eur J Clin Nutr. 2007;61(6):736-42.
308. Jaakkola J, Isolauri E, Poussa T, Laitinen K. Benefits of repeated individual dietary counselling in long-term weight control in women after delivery. Maternal & Child Nutrition 2015;11:1041–8.
309. Jackson MR, Gott P, Lye SJ, Ritchie JW, Clapp JF. The effects of maternal aerobic exercise on human placental development: placental volumetric composition and surface areas. Placenta 1995;16:179–91.
310. Jackson R., Stotland N., Caughey A., Gerbert B. Improving diet and exercise in pregnancy with Video Doctor counseling: A randomized trial. Patient Educ. Couns. 2011;83:203–209. doi: 10.1016/j.pec.2010.05.019.
311. Jefferies K, Shub A, Walker S, Hiscock R, Permezel M. Reducing excessive weight gain in pregnancy: a randomised controlled trial. Med J Aust. 2009;191: 429–33.
312. Jennifer G, Swathy S, Jenn L. Antenatal physical activity: Investigating the effects on postpartum depression. 2012.
313. Jennifer M, Tiffany F, Miguel D, Debra B, Rae N, Martha P. Yoga reduces prenatal depression symptoms. Complement Ther Clin Pract. 2012;19(1):6.
314. Jiang H, Qian X, Li M, et al. Can physical activity reduce excessive gestational weight gain? Findings from a Chinese urban pregnant women cohort study. Int J Behav Nutr Phys Act. 2012;9(9):12. doi:10.1186/1479-5868-9-12.
315. Jiang YSL. Influence of appropriate nutrition and gymnastic training on pregnancy outcomes. Chin J Women Child Health 2015;6(5):47–8.
316. Jing W, Huang Y, Liu X, et al. The effect of a personalized intervention on weight gain and physical activity among pregnant women in China. Int J Gynaecol Obstet 2015;129:138–41.
317. Jovanovic L, Kessler A, Peterson CM. Human maternal and fetal response to graded exercise. J Appl Physiol (1985). 1985;58(5):1719-22.
318. Jovanovic-Peterson L, Durak EP, Peterson CM. Randomized trial of diet versus diet plus cardiovascular conditioning on glucose levels in gestational diabetes. Am J Obstet Gynecol 1989;161:415–9
319. Juhl M, Andersen PK, Olsen J, et al. Physical exercise during pregnancy and the risk of preterm birth: a study within the Danish national birth cohort. Am J Epidemiol. 2008;167(7): 859–66. doi:10.1093/aje/kwm364.
320. Juhl M, Kogevinas M, Andersen PK, Andersen AM, Olsen J. Is swimming during pregnancy a safe exercise? Epidemiology. 2010;21(2):253-8.
321. Juhl M, Olsen J, Andersen PK, et al. Physical exercise during pregnancy and fetal growth measures: a study within the Danish National Birth Cohort. Am J Obstet Gynecol. 2010;202(1): e1–8. doi:10.1016/j.ajog.2009.07.033.
322. Jukic AMZ, Evenson KR, Daniels JL, et al. Prospective study of the association between vigorous physical activity during pregnancy and length of gestation and birthweight. Matern Child Health J. 2012;16(5):1031–44. doi:10.1007/s10995-011-0831-8.
323. Kafatos A., Vlachonikolis I. & Codrington C. (1989) Nutrition during pregnancy: the effects of an educational intervention program in Greece. The American Journal of Clinical Nutrition 50, 970–979.

Diet and/or physical activity unique publications

324. Kalayeh SMO, Nikbakht R, Seddigi S. The effect of exercise during pregnancy on mother's depression in Zahedan. International Journal of Pharmacy and Technology. 2016.
325. Kannieappan LM, Deussen AR, Moran LJ, Grivell RM, Yelland LN, Dodd JM. The effect of antenatal dietary advice on maternal body composition in women who are overweight or obese - findings from the LIMIT randomised trial. Journal of Paediatrics and Child Health 2013;49 Suppl 2:94.
326. Kardel KR, Kase T. Training in pregnant women: effects on fetal development and birth. Am J Obstet Gynecol 1998;178:280–6.
327. Kariminia A, Chamberlain ME, Keogh J, Shea A. Randomised controlled trial of effect of hands and knees posturing on incidence of occiput posterior position at birth. BMJ. 2004;328(7438):490.
328. Kasawara KT, Burgos CS, do Nascimento SL, Ferreira NO, Surita FG, Pinto E Silva JL. Maternal and perinatal outcomes of exercise in pregnant women with chronic hypertension and/or previous preeclampsia: a randomized controlled trial. ISRN Obstet Gynecol 2013;2013:857047.
329. Khaledan A, Mirdar S, Motahari Tabari N, Ahmad SM. Effect of an aerobic exercise program on fetal growth in pregnant women. J HAYAT 2010;16:55–64.
330. Khoury J, Henriksen T, Christophersen B, Tonstad S. Effect of a cholesterol-lowering diet on maternal, cord, and neonatal lipids, and pregnancy outcome: a randomised clinical trial. Am J Obstet Gynecol 2005;193:1292–301.
331. Kieffer EC, Welmerink DB, Sinco BR, Welch KB, Rees Clayton EM, Schumann CY, et al. Dietary outcomes in a Spanish-language randomized controlled diabetes prevention trial with pregnant Latinas. American Journal of Public Health 2014;104(3):526–33.
332. Kihlstrand M, Stenman B, Nilsson S, Axelsson O. Water-gymnastics reduced the intensity of back/low back pain in pregnant women. Acta Obstet Gynecol Scand 1999;78:180–5.
333. Kinnunen TI, Pasanen M, Aittasalo M, Fogelholm M, Hilakivi-Clarke L, Weiderpass E, Luoto R: Preventing excessive weight gain during pregnancy - a controlled trial in primary health care. Eur J Clin Nutr 2007, 61:884-891.
334. Kinnunen TI, Puhkala J, Raitanen J, Ahonen S, Aittasalo M, Virtanen SM, et al. Effects of dietary counselling on food habits and dietary intake of Finnish pregnant women at increased risk for gestational diabetes - a secondary analysis of a cluster-randomized controlled trial. Maternal and Child Nutrition 2014;10(2):184–97.?
335. Kizirian N, Garnett S, Markovic T, Ross G, Louie J, Muirhead R, et al. Effects of a low-glycaemic index diet during pregnancy on offspring body composition: a pilot study. Obesity Research and Clinical Practice 2013;7(Suppl 2):e103
336. Kizirian N, Garnett S, Markovic T, Ross G, Muirhead R, Brodie S, et al. Maternal diet and infant body composition in women at risk of gestational diabetes mellitus. Obesity Research and Clinical Practice 2014;8(Suppl 1):55
337. Kluge J, Hall D, Louw Q, et al. Specific exercises to treat pregnancy-related low back pain in a South African population. Int J Gynaecol Obstet 2011;113:187–91.
338. Knudsen VK, Orozova-Bekkevold IM, Mikkelsen TB, Wolff S, Olsen SF. Major dietary patterns in pregnancy and fetal growth. Eur J Clin Nutr 2008;62:463–70.
339. Knuist M, Bonsel GJ, Zondervan HA, Treffers PE. Low sodium diet and pregnancy-induced hypertension: a multi-centre randomised controlled trial. Br J Obstet Gynaecol 1998;105:430–4.
340. Ko CW, Napolitano PG, Lee SP, Schulte SD, Ciol MA, Beresford SA. Physical activity, maternal metabolic measures, and the incidence of gallbladder sludge or stones during pregnancy: a randomized trial. Am J Perinatol. 2014;31(1):39-48.
341. Ko PC, Liang CC, Chang SD, Lee JT, Chao AS, Cheng PJ. A randomized controlled trial of antenatal pelvic floor exercises to prevent and treat urinary incontinence. Int Urogynecol J. 2011;22(1):17-22.
342. Kobe H, Nakai A, Koshino T, Araki T. Effect of regular maternal exercise on lipid peroxidation levels and antioxidant enzymatic activities before and after delivery. J Nippon Med Sch. 2002;69(6):5428.
343. Koivusalo 2016 {published data only} Grotenfelt NE, Wasenius NS, Rono K, Laivuori H, Stach-Lempinen B, Orho-Melander M, et al. Interaction between rs10830963 polymorphism in mtnr1b and lifestyle intervention on occurrence of gestational diabetes. Diabetologia 2016;59(8):1655–8.?
344. Koivusalo SB, Rono K, Klemetti MM et al. Gestational diabetes mellitus can be prevented by lifestyle intervention: the Finnish gestational diabetes prevention study (RADIEL): a randomized controlled trial. Diabetes Care 2016; 39: 24–30 PubMed PMID: 26223239.
345. Kolu P, Raitanen J, Luoto R. Physical activity and health- related quality of life during pregnancy: a secondary analysis of a cluster-randomised trial. Maternal and Child Health Journal 2014;18(9):2098–105.?
346. Kolu P, Raitanen J, Rissanen P, Luoto R. Cost-effectiveness of lifestyle counselling as primary prevention of gestational diabetes mellitus: findings from a cluster-randomised trial. PLOS One 2013;8(2):e56392.
347. Kolu P, Raitanen J, Rissanen P, Luoto R. Health care costs associated with gestational diabetes mellitus among high-risk women - results from a randomised trial. BMC Pregnancy and Childbirth 2012;12:71.?
348. Kong K L, Campbell C G, Foster R C, Peterson A D, Lanningham-Foster L. A pilot walking program promotes moderate-intensity physical activity during pregnancy. Med Sci Sports Exerc. 2014;46(03):462–471. [PubMed] [Google Scholar]
349. Kordi R, Abolhasani M, Rostami M, Hantoushzadeh S, Mansournia MA, Vasheghani-Farahani F. Comparison between the effect of lumbopelvic belt and home based pelvic stabilizing exercise on pregnant women with pelvic girdle pain; a randomized controlled trial. J Back Musculoskelet Rehabil. 2013;26(2):133-9.

Diet and/or physical activity unique publications

350. Korpi-Hyövälti E A, Laaksonen D E, Schwab U S et al.Feasibility of a lifestyle intervention in early pregnancy to prevent deterioration of glucose tolerance. BMC Public Health. 2011;11:179. [PMC free article] [PubMed] [Google Scholar]
351. Korpi-Hyovalti E., Schwab U., Laaksonen D.E., Linjama H., Heinonen S., Niskanen L. Effect of intensive counselling on the quality of dietary fats in pregnant women at high risk of gestational diabetes mellitus. Br. J. Nutr. 2012;108:910–917. doi: 10.1017/S0007114511006118.
352. Kraschnewski JL, Chuang CH, Downs DS, et al. Association of prenatal physical activity and gestational weight gain: results from the first baby study. Womens Health Issues. 2013;23(4): e233–8. doi:10.1016/j.whi.2013.04.004.
353. Kulpa PJ, White BM, Visscher R. Aerobic exercise in pregnancy. Am J Obstet Gynecol 1987;156:1395-403.
354. Kusin JA, Kardjati S, Houtkooper JM, Renqvist UH. Energy supplementation during pregnancy and postnatal growth. Lancet 1992;340:623–6.
355. Labonte-Lemoyne E, Curnier D, Ellemberg D. Exercise during pregnancy enhances cerebral maturation in the newborn: a randomized controlled trial. J Clin Exp Neuropsychol 2017;39:347–54.
356. Laitinen K, Ilmonen J, Isolauri E. Dietary counselling and probiotic intervention during pregnancy modify postpartum adiposity. Annals of Nutrition and Metabolism 2011;58(Suppl 3):87
357. Laitinen K, Poussa T, Isolauri E, Nutrition AMI. Intestinal microbiota G. Probiotics and dietary counselling contribute to glucose regulation during and after pregnancy: a randomised controlled trial. Br J Nutr. 2009;101:1679–87.
358. Laitinen K, Poussa T, Isolauri E, Nutrition, Allergy, Mucosal Immunology and Intestinal Microbiota Group. Probiotics and dietary counselling contribute to glucose regulation during and after pregnancy: a randomised controlled trial. British Journal of Nutrition 2009;101(11): 1679–87
359. Laitinen K, Poussa T, Isolauri E. Probiotics and dietary counselling contribute to glucose regulation during and after pregnancy: A randomised controlled trial. Br J Nutr 2009; 101:1679–1687.
360. Landon M, Spong C, Thom E, Carpenter MW, Ramins S, Casey B, et al. A multicenter, randomised trial of treatment for mild gestational diabetes. N Engl J Med. 2009;361:1339–48.
361. Lawani MM, Alihonou E, Akplogan B, et al. [Effect of antenatal gymnastics on childbirth: a study on 50 sedentary women in the Republic of Benin during the second and third quarters of pregnancy]. Sante 2003;13:235–41.
362. Lee G, Challenger S, McNabb M, Sheridan M. Exercise in pregnancy. Mod Midwife 1996;6:28–33.
363. Leermakers EA, Anglin K, Wing RR. Reducing postpartum weight retention through a correspondence intervention. Int J Obes Relat Metab Disord 1998;22(11):1103–9
364. Lenders CM, Hediger ML, Scholl TO, Khoo CS, Slap GB, Stallings VA. Effect of high sugar intake by low-income pregnant adolescents on infant birth weight. J Adolesc Health 1994;15:596–602.
365. Lenders CM, Hediger ML, Scholl TO, Khoo CS, Slap GB, Stallings VA. Gestational age and infant size at birth are associated with dietary sugar intake among pregnant adolescents. J Nutr 1997;127:1113–17.
366. Leng J, Liu G, Zhang C, Xin S, Chen F, Li B, et al. Physical activity, sedentary behaviors and risk of gestational diabetes mellitus: a population-based cross-sectional study in Tianjin, China. Eur J Endocrinol. 2016;174(6):763-73.
367. Leppanen M, Aittasalo M, Raitanen J, Kinnunen TI, Kujala UM, Luoto R. Physical activity during pregnancy: predictors of change, perceived support and barriers among women at increased risk of gestational diabetes. Maternal and Child Health Journal 2014;18(9):2158–66.?
368. Lesser KB, Gruppuso PA, Terry RB, Carpenter MW. Exercise fails to improve postprandial glycemic excursion in women with gestational diabetes. J Matern Fetal Med. 1996;5(4):211-7.
369. Li Q, Xiong R, Wang L, Cui J, Shi L, Liu Y, et al. Associations of dietary habits, physical activity and cognitive views with gestational diabetes mellitus among Chinese women. Public Health Nutrition. 2014;17(8):1850-7.
370. Liao Q, Zhu S, Pang Y, Wei Z. The effect of individual nutrition instruction on nutritional status and birth outcome of pregnant women. Matern Child Healthcare Chin 2012;27(28):4388–90.
371. Lindholm ES, Norman M, Kilander CP, Altman D. Weight control program for obese pregnant women. Acta Obstet Gynecol Scand 2010; 89: 840-43.
372. Lindqvist M, Lindkvist M, Eurenius E, Persson M, Ivarsson A, Mogren I. Leisure time physical activity among pregnant women and its associations with maternal characteristics and pregnancy outcomes. Sex Reprod Healthc. 2016;9:14-20.
373. Lindsay K, Kennelly M, Smith T, Maguire O, Shanahan F, Brennan L, et al. PPO.19 Probiotics in obese pregnancy to reduce maternal fasting glucose: A randomised controlled trial. Arch Dis Child - Fetal Neonatal Ed 2014; 99: A156.1-A156.
374. Lisa AU, Cynthia LB, Kaeli AS, Susanna RM, Ivan WM. A pilot randomized controlled trial comparing prenatal yoga to perinatal health education for antenatal depression. Archives of women's mental health. 2015;19(3):543.
375. Liu J, Wilcox S, Whitaker K, Blake C, Addy C. Preventing excessive weight gain during pregnancy and promoting postpartum weight loss: a pilot lifestyle intervention for overweight and obese African American women. Matern Child Health J. 2015;19(4):840-9.
376. Lombard C, Harrison C, Teede H. A randomized controlled trial investigating self-weighing and the prevention of excess weight gain in early pregnancy. Endocrine Reviews 2011;32 (3 Meeting Abstracts):P2–768.?
377. Lombardi W, Wilson S, Peniston PB. Wellness intervention with pregnant soldiers. Mil Med 1999;164:22–9.
378. Loprinzi PD, Loprinzi KL, Cardinal BJ. The relationship between physical activity and sleep among pregnant women. 2012.

Diet and/or physical activity unique publications
379. Lotgering FK, Spinnewijn WE, Struijk PC, Boomsma F, Wallenburg HC. Respiratory and metabolic responses to endurance cycle exercise in pregnant and postpartum women. Int J Sports Med. 1998;19(3):193-8.
380. Louie JCY, Markovic TP, Perera N, Foote D, Petocz P, Ross GP, Brand-Miller JC (2011) A randomized controlled trial investigating the effects of a low-glycemic index diet on pregnancy outcomes in gestational diabetes mellitus. Diabetes Care 34:2341–2346
381. Lovelady CA, Garner KE, Moreno KL, Williams JP. The effect of weight loss in overweight, lactating women on the growth of their infants. New Engl J Med 2000;342(7):449–53.
382. Lumey LH, Stein AD, Kahn HS, Romijn JA. Lipid profiles in middle-aged men and women after famine exposure during gestation: the Dutch Hunger Winter Families Study. Am J Clin Nutr 2009;89:1737–43.
383. Luo X-D, Dong X, Zhou J. Effects of nutritional management intervention on gestational weight gain and perinatal outcome. Saudi Med J. 2014;35(10):1267–1270. [PMC free article] [PubMed] [Google Scholar]
384. Luoto R, Kinnunen T I, Aittasalo M et al.Primary prevention of gestational diabetes mellitus and large-for-gestational-age newborns by lifestyle counseling: a cluster-randomized controlled trial. PLoS Med. 2011;8(05):e1001036. [PMC free article] [PubMed] [Google Scholar]
385. Luoto R, Kinnunen T, Aittasalo M, Kolu P, Raitanen J, Ojala K, Mansikkama¨ki K, Lamberg S, Vasankari T, Komulainen T, et al. Primary prevention of gestational diabetes mellitus and large-for-gestational age newborns by lifestyle counseling: a cluster-randomized controlled trial. PLoS Med 2011;8:e1001036.
386. Luoto R, Laitinen K, Nermes M, Isolauri E, Luoto R, et al. (2010) Impact of maternal probiotic-supplemented dietary counselling on pregnancy outcome and prenatal and postnatal growth: a double-blind,placebo-controlled study. Br J Nutr 103(12):1792–9. doi: 10.1017/S0007114509993898 PMID: 20128938.
387. Luoto R., Kinnunen T., Aittasalo M., Ojala K., Mansikkamaki K., Toropainen E., Kolu P., Vasankari T. Prevention of gestational diabetes: Design of a cluster-randomized controlled trial and one-year follow-up. BMC Pregnancy Childbirth. 2010;10:39. doi: 10.1186/1471-2393-10-39.
388. Lynch AM, Goodman C, Choy PL, Dawson B, Newnham JP, McDonald S, et al. Maternal physiological responses to swimming training during the second trimester of pregnancy. Res Sports Med. 2007;15(1):33-45.
389. Ma WJ, Huang ZH, Huang BX, Qi BH, Zhang YJ, Xiao BX, Li YH, Chen L, Zhu HL (2015) Intensive low-glycaemic-load dietary intervention for the management of glycaemia and serum lipids among women with gestational diabetes: a randomized control trial. Public Health Nutr 18:1506–1513
390. Maconochie N, Doyle P, Prior S, et al. Risk factors for first trimester miscarriage-- results from a UK-population-based case-control study. BJOG 2007;114:170–86.
391. Madsen M, Jørgensen T, Jensen ML, et al. Leisure time physical exercise during pregnancy and the risk of miscarriage: a study within the Danish National Birth Cohort. BJOG 2007;114:1419–26.
392. Magann EF, Evans SF, Weitz B, et al. Antepartum, intrapartum, and neonatal significance of exercise on healthy low-risk pregnant working women. Obstet Gynecol 2002;99:466–72.
393. Magnus P, Trogstad L, Owe KM, Olsen SF, Nystad W. Recreational physical activity and the risk of preeclampsia: a prospective cohort of Norwegian women. Am J Epidemiol. 2008;168(8):952-7.
394. Mahony R, Byrne J, Curran S, O'Herlihy C, McAuliffe F. A pilot study of the feasibility of a randomised trial of low glycaemic diet versus normal diet from early pregnancy in euglycaemic women. Archives of Disease in Childhood. Fetal and Neonatal Edition 2008;93(Suppl 1):Fa38
395. Maitland RA, Barr S, Briley A, Seed P, Poston L. Incidence of gestational diabetes in an obese population using the International Association of Diabetes and Pregnancy Study Groups (IADPSG) criteria in the UK Pregnancies Better Eating and Activity Trial (UPBEAT) pilot study. Diabetic Medicine 2012;29(Suppl 1):152.?
396. Makela J, Lagstrom H, Kaljonen A, Simell O, Niinikoski H. Hyperglycemia and lower diet quality in pregnant overweight women and increased infant size at birth and at 13 months of age--STEPS study. 2013.
397. Marcoux S, Brisson J, Fabia J. The effect of leisure time physical activity on the risk of pre-eclampsia and gestational hypertension. J Epidemiol Community Health. 1989;43(2):147-52.
398. Mardones-Santander F, Rosso P, Stekel A, Ahumada E, Llaguno S, Pizarro F, Salinas J, Vial I, Walter T. Effect of a milk-based food supplement on maternal nutritional status and fetal growth in underweight Chilean women. Am J Clin Nutr 1988;47:413–9.
399. Markovic 2016 ACTRN12610000681055. A randomized, two-arm parallel dietary intervention study to compare the effects of consuming a low glycemic diet or wholegrain high fibre diet on infant birth weight and body composition, complications related to Gestational Diabetes Mellitus (GDM) and progression to GDM diagnosis in women at high-risk of GDM. anzctr.org.au/Trial/Registration/ TrialReview.aspx?id=335632 Date first received: 18 August 2010
400. Markovic T., Muirhead R., Overs S., Ross G., Louie J., Kizirian N., et al. Randomized Controlled Trial Investigating the Effects of a Low-Glycemic Index Diet on Pregnancy Outcomes in Women at High Risk of Gestational Diabetes Mellitus: The GI Baby 3 Study. Diabetes Care. 2016;39:31–38. doi: 10.2337/dc15-0572.
401. Markovic TP, Muirhead R, Overs S, Kizirian N, Louie J, Sweeting A, et al. Predictors of birthweight in women at high risk of gestational diabetes mellitus. Obesity Research and Clinical Practice 2013;7(2):e3–4
402. Marquez-Sterling S, Perry AC, Kaplan TA, et al. Physical and psychological changes with vigorous exercise in sedentary primigravidae. Med Sci Sports Exerc 2000;32:58–62.
403. Martin CL, Brunner Huber LR. Physical activity and hypertensive complications during pregnancy: findings from 2004 to 2006 North Carolina Pregnancy Risk Assessment Monitoring System. Birth. 2010;37(3):202-10.

Diet and/or physical activity unique publications

404. Martin J, MacDonald-Wicks L, Hure A, Smith R, Collins CE. Reducing postpartum weight retention and improving breastfeeding outcomes in overweight women: A pilot randomised controlled trial. Nutrients 2015;7(3):1465–79

405. Mason L, Roe B, Wong H, Davies J, Bamber J. The role of antenatal pelvic floor muscle exercises in prevention of postpartum stress incontinence: a randomised controlled trial. J Clin Nurs. 2010;19(1920):2777-86.

406. McAuley SE, Jensen D, McGrath MJ, Wolfe LA. Effects of human pregnancy and aerobic conditioning on alveolar gas exchange during exercise. Can J Physiol Pharmacol. 2005;83(7):625-33.

407. McAuliffe F. A randomised controlled trial of low glycaemic index carbohydrate diet versus no dietary intervention in the prevention of recurrence of foetal macrosomia. CurrentControlled Trials (www.controlled-trials.com/) (accessed 12.05.2010).

408. McAuliffe F. Maternal nutrition and fetal health. The Power of Programming 2014: International Conference on Developmental Origins of Adiposity and Long-Term Health; 2014 March 13-15; Munich, Germany. 2014

409. McCarthy E, Walker S, Ugoni A, Lappas M, Leong O, Shub A. Self-weighing and simple dietary advice for overweight and obese pregnant women to reduce obstetric complications without impact on quality of life: a randomised controlled trial. BJOG 2016;123:965–973.

410. McCullough LE, Mendez MA, Miller EE, et al. Associations between prenatal physical activity, birth weight, and DNA methylation at genomically imprinted domains in a multi-ethnic newborn cohort. Epigenetics. 2015;10(7):597–606. doi:10.1080/15592294.2015.1045181.

411. McDonald EC, Pollitt E, Mueller W. The bacon chow study: maternal nutritional supplementation and birth weight of offspring. Am J Clin Nutr 1981;34:2133–44.

412. McEachan RRC, Santorelli G, Bryant M, et al. The HAPPY (Healthy and Active Parenting Programmme for early Years) feasibility randomised control trial: acceptability and feasibility of an intervention to reduce infant obesity. BMC Public Health. 2016;16(1):211-211.

413. McGiveron A, Foster S, Pearce J, Taylor MA, McMullen S, Langley-Evans SC. Limiting antenatal weight gain improves maternal health outcomes in severely obese pregnant women: findings of a pragmatic evaluation of a midwife-led intervention. J Hum Nutr Diet. 2015;28 Suppl 1:29-37.

414. McGowan C A, Walsh J M, Byrne J, Curran S, McAuliffe F M. The influence of a low glycemic index dietary intervention on maternal dietary intake, glycemic index and gestational weight gain during pregnancy: a randomized controlled trial. Nutr J. 2013;12(01):140. [PMC free article] [PubMed] [Google Scholar]

415. McMurray RG, Hackney AC, Guion WK, Katz VL. Metabolic and hormonal responses to low-impact aerobic dance during pregnancy. Med Sci Sports Exerc. 1996;28(1):41-6.

416. Medek H, Halldorsson T, Gunnarsdottir I, Geirsson RT. Physical activity of relatively high intensity in mid-pregnancy predicts lower glucose tolerance levels. Acta Obstet Gynecol Scand. 2016;95(9):1055-62.

417. Melo A. Exercise and pregnancy: randomised clinical trial. Current Controlled Trials (www.controlled-trials.com/)[accessed 31 October 2010].

418. Melzer K, Schutz Y, Soehnchen N, et al. Effects of recommended levels of physical activity on pregnancy outcomes. Am J Obstet Gynecol. 2010;202(3):266.e1–6. doi:10.1016/j.ajog. 2009.10.876.

419. Metcoff J, Costiloe P, Crosby WM, Dutta S, Sandstead HH, Milne D, Bodwell CE, Majors SH. Effect of food supplementation (WIC) during pregnancy on birth weight. Am J Clin Nutr 1985;41:933–47.

420. Miquelutti MA, Cecatti JG, Makuch MY. Evaluation of a birth preparation program on lumbopelvic pain, urinary incontinence, anxiety and exercise: a randomized controlled trial. BMC Pregnancy Childbirth. 2013 Jul 29;13:154. doi: 10.1186/1471-2393-13-154. PubMed PMID: 23895188; PubMed Central PMCID: PMC3750492

421. Misra DP, Strobino DM, Stashinko EE, et al. Effects of physical activity on preterm birth. Am J Epidemiol. 1998;147(7):628–35.

422. Mohammadi F, Malakooti J, Babapoor J, Mohammad-Alizadeh-Charandabi S. The effect of a home-based exercise intervention on postnatal depression and fatigue: A randomized controlled trial. Int J Nurs Pract. 2015;21(5):478-85.

423. Momeni Javid F, Simbar M, Dolatian M, Alavi Majd H. Comparison of lifestyles of women with gestational diabetes and healthy pregnant women. Glob J Health Sci. 2015;7(2):162-9.

424. Monika G. PHYSICAL ACTIVITY IN RELATION TO AFFECTIVE STATES AND LABOR ANXIETY IN PREGNANT WOMEN. Medicina Sportiva 2011;15(3):114-8.

425. Mora JO, Clement J, Christiansen N, Suescun J, Wagner M, Herrera MG. Nutritonal supplementation and the outcome of pregnancy. III. Perinatal and neonatal mortality. Nutr Rep Int 1978;18:167–75.

426. Morgan KL, Rahman MA, Hill RA, et al. Physical activity and excess weight in pregnancy have independent and unique effects on delivery and perinatal outcomes. PLoS One. 2014;9(4): e94532. doi:10.1371/journal.pone.0094532.

427. Morkrid K, Jenum AK, Berntsen S, et al. Objectively recorded physical activity and the association with gestational diabetes. Scand J Med Sci Sports. 2014;24(5):e389–97. doi:10.1111/sms.12183.

428. Morkved S, Bo K, Schei B, Salvesen KA. Pelvic floor muscle training during pregnancy to prevent urinary incontinence: a single-blind randomized controlled trial. Obstet Gynecol. 2003;101(2):313-9.

429. Morkved S. Effects of regular exercise during pregnancy. ClinicalTrials.gov (http://clinicaltrials.gov/) (accessed 20 February 2008). ∗ Stafne SN, Salvesen KA, Romundstad PR, Eggebo TM, Carlsen SM, Morkved S. Regular exercise during pregnancy to prevent gestational diabetes: a randomized controlled trial. Obstetrics and Gynecology 2012;119(1):29–36.

430. Moses R., Casey S., Quinn E., Cleary J., Tapsell L., Milosavljevic M., Petocz P., Brand-Miller J.C. Pregnancy and Glycemic Index Outcomes study: Effects of low glycemic index compared with conventional dietary advice on selected pregnancy outcomes. Am. J. Clin. Nutr. 2014;99:517–523. doi: 10.3945/ajcn.113.074138.

Diet and/or physical activity unique publications

431. Moses RG, Barker M, Winter M, Petocz P, Brand-Miller JC (2009) Can a low-glycemic index diet reduce the need for insulin in gestational diabetes mellitus? A randomized trial. Diabetes Care 32:996–1000
432. Moses RG, Casey S, Cleary J, Milosavljevic M, Quinn E, Tapsell L, et al. Effect of low glycaemic index dietary advice in normal pregnancy: The PREGGIO study. Obesity Research and Clinical Practice 2013;7:e34–5
433. Moses RG, Luebke M, Petocz P, Brand-Miller JC. Maternal diet and infant size 2 y after the completion of a study of a low-glycemic-index diet in pregnancy. American Journal of Clinical Nutrition 2007;86(6):1806
434. Mottola MF, Giroux I, Gratton R et al. Nutrition and exercise prevent excess weight gain in overweight pregnant women. Med Sci Sports Exerc 2010; 42: 265-72.
435. Mottola MF, Inglis S, Brun CR, Hammond JA. Physiological and metabolic responses of late pregnant women to 40 min of steady-state exercise followed by an oral glucose tolerance perturbation. J Appl Physiol (1985). 2013;115(5):597-604.
436. Mudd LM, Pivarnik J, Holzman CB, et al. Leisure-time physical activity in pregnancy and the birth weight distribution: where is the effect? J Phys Act Health. 2012;9(8):1168–77.
437. Mujsindi W, Habash D, Childs G. Impact of nutrition education on gestational weight gain in obese pregnant women. American Journal of Obstetrics and Gynecology 2014;210(1 Suppl):S188.
438. Murtezani A, Pac¸arada M, Ibraim Z, et al. The impact of exercise during pregnancy on neonatal outcomes: a randomized controlled trial. J Sports Med Phys Fitness. 2014;54(6):802–8.
439. Mustila T, Raitanen J, Keskinen P, Saari A, Luoto R. Lifestyle counseling during pregnancy and offspring weight development until four years of age: follow-up study of a controlled trial. J Negat Results Biomed. 2012;11:11. [PMC free article] [PubMed] [Google Scholar]
440. Narendran S, Nagarathna R, Narendran V, Gunasheela S, Nagendra HR. Efficacy of yoga on pregnancy outcome. J Altern Complement Med 2005;11:237–44.
441. Nascimento KLK, Surita SLN, Parpinelli FGS, Kasawara MAP. Type of delivery and neonatal outcome in overweight and obese pregnant women with excessive weight gain. Journal of Maternal-Fetal and Neonatal Medicine 2012;25(S2):73–4.
442. Nascimento S L, Surita F G, Parpinelli MÂ, Siani S, Pinto e Silva J L. The effect of an antenatal physical exercise programme on maternal/perinatal outcomes and quality of life in overweight and obese pregnant women: a randomised clinical trial. BJOG. 2011;118(12):1455–1463. [PubMed] [Google Scholar]
443. Nasiri-Amiri F, Bakhtiari A, Faramarzi M, Adib Rad H, Pasha H. The Association Between Physical Activity During Pregnancy and Gestational Diabetes Mellitus: A Case-Control Study. Int J Endocrinol Metab. 2016;14(3):e37123.
444. Neugebauer R, Hoek HW, Susser E. Prenatal exposure to wartime famine and development of antisocial personality disorder in early adulthood. JAMA 1999;282:455–62.
445. Newham JJ, Wittkowski A, Hurley J, Aplin JD, Westwood M. Effects of antenatal yoga on maternal anxiety and depression: a randomized controlled trial. Depress Anxiety. 2014;31(8):631-40.
446. Newman AK, Deussen AR, Moran LJ, Grivell RM, Yelland LN, Turnbull D, et al. The effect of antenatal dietary and lifestyle advice on maternal psychological health in women who are overweight or obese-findings from the LIMIT randomised trial. Journal of Paediatrics and Child Health 2013;49 Suppl 2:119.
447. Ney D, Hollingsworth DR, Cousins L. Decreased insulin requirement and improved control of diabetes in pregnant women given a high-carbohydrate, high-fiber, low-fat diet. Diabetes Care 1982;5:529-33.
448. Nielsen CA, Sigsgaard I, Olsen M, Tolstrup M, Danneskiold-Samsoee B, Bock JE. Trainability of the pelvic floor. A prospective study during pregnancy and after delivery. Acta Obstet Gynecol Scand. 1988;67(5):437-40.
449. Nieuwenhuijsen MJ, Northstone K, Golding J. Swimming and birth weight. Epidemiology. 2002;13(6):725–8.
450. Niinivirta K, Isolauri E, Laakso P, Linderborg K, Laitinen K. Dietary counseling to improve fat quality during pregnancy alters maternal fat intake and infant essential fatty acid status. Journal of Nutrition 2011;141(7):1281–5
451. Niinivirta K, Laakso P, Linderborg K, Poussa T, Isolauri E, Laitinen K. Maternal dietary counseling during pregnancy and infant fatty acid profiles. International Journal of Food Sciences and Nutrition 2014;65(3):268–72
452. Nilsson K. Weight gain during pregnancy - a randomized controlled trial of intervention to prevent excessive gestational weight gain. ClinicalTrials.gov (http://clinicaltrials.gov/) [accessed 5 September 2014] 2009.
453. Nobles C, Marcus BH, Stanek EJ 3rd et al. Effect of an exercise intervention on gestational diabetes mellitus: a randomized controlled trial. Obstet Gynecol 2015; 125: 1195–204 PubMed PMID: 25932848. PubMed Central PMCID: 4418021.
454. O'Toole ML, Sawicki MA, Artal R. Structured diet and physical activity prevent postpartum weight retention. J Womens Health 2003;12(10):991–8.
455. O'Brien, C.M., R.M. Grivell, and J.M. Dodd, Systematic review of antenatal dietary and lifestyle interventions in women with a normal body mass index. Acta Obstet Gynecol Scand, 2016. 95(3): p. 259-69.
456. O'Connor PJ, Poudevigne MS, Cress ME, Motl RW, Clapp JF, 3rd. Safety and efficacy of supervised strength training adopted in pregnancy. J Phys Act Health. 2011;8(3):309-20.

Diet and/or physical activity unique publications

457. Oken E, Ning Y, Rifas-Shiman SL, Radesky JS, Rich-Edwards JW, Gillman MW. Associations of physical activity and inactivity before and during pregnancy with glucose tolerance. Obstet Gynecol. 2006;108(5):1200-7.
458. Okesene-Gafa KAM, Li M, McKinlay CJD, Taylor RS, Rush EC, Wall CR, et al. Effect of antenatal dietary interventions in maternal obesity on pregnancy weight-gain and birthweight: Healthy Mums and Babies (HUMBA) randomized trial. Am JObstet Gynecol 2019.
459. Okido MM, Valeri FL, Martins WP, Ferreira CH, Duarte G, Cavalli RC. Assessment of foetal wellbeing in pregnant women subjected to pelvic floor muscle training: a controlled randomised study. Int Urogynecol J. 2015;26(10):1475-81.
460. Olsen C., Groth S., Graham M., Reschke J., Strawderman M., Fernandez D. The effectiveness of an online intervention in preventing excessive gestational weight gain: The e-moms roc randomized controlled trial. BMC Pregnancy Childbirth. 2018;18:148. doi: 10.1186/s12884-018-1767-4.
461. Olsen SF, Secher NJ, Tabor A, Weber T, Walker JJ, Gluud C. Randomised clinical trials of fish oil supplementation in high risk pregnancies. BJOG. 2000;107:382–95.
462. Olson CM, Strawderman MS, Reed RG. Efficacy of an intervention to prevent excessive gestational weight gain. Am J Obstet Gynecol 2004; 191: 530-36.
463. Olson CM: A call for intervention in pregnancy to prevent maternal and child obesity. Am J Prev Med 2007, 33:435-436.
464. Ong MJ, Guelfi KJ, Hunter T, et al. Supervised home-based exercise may attenuate the decline of glucose tolerance in obese pregnant women. Diabetes Metab 2009; 35: 418–421.
465. Onwude JL, Lilford RJ, Hjartardottir H, Staines A, Tuffnell D. A randomised double blind placebo controlled trial of fish oil in high risk pregnancy. BJOG. 1995;102:95–100.
466. Oostdam N, Bosmans J, Wouters MGAJ, Eekhoff EMW, van Mechelen W, van Poppel MNM. Cost-effectiveness of an exercise program during pregnancy to prevent gestational diabetes: Results of an economic evaluation alongside a randomised controlled trial. BMC Pregnancy and Childbirth 2012;12:64.
467. Oostdam N, Van Poppel MN, Eekhoff EM, Wouters MG, Van Mechelen W. Design of FitFor2 study: the effects of an exercise program on insulin sensitivity and plasma glucose levels in pregnant women at high risk for gestational diabetes. BMC Pregnancy and Childbirth 2009;9:1.
468. Oostdam N, Poppel MN, Wouters MG, Eekhoff EM, Bekedam DJ, Kuchenbecker WK, et al. No effect of the FitFor2 exercise programme on blood glucose, insulin sensitivity, and birthweight in pregnant women who were overweight and at risk for gestational diabetes: results of a randomised controlled trial. BJOG : an international journal of obstetrics and gynaecology. 2012; https://doi.org/10.1111/j.1471-0528.2012.03366.x.
469. Orawan L, Preecha W. Effect of pelvic floor muscle training (PFMT) during pregnancy on bladder neck descend and delivery. Journal of the Medical Association of Thailand. 2014;97(Suppl 8):S156-63.
470. Orr ST, James SA, Garry J, Prince CB, Newton ER. Exercise and pregnancy outcome among urban, low-income, black women. EthnDis. 2006;16(4):933-7.
471. Ostbye T, Krause KM, Lovelady CA, Morey MC, Bastian LA, Peterson BL, et al. Active Mothers Postpartum. A Randomized Controlled Weight-Loss Intervention Trial. Am J Prev Med 2009;37(3):173–80.
472. Osterdal ML, Strom M, Klemmensen AK, et al. Does leisure time physical activity in early pregnancy protect against preeclampsia? Prospective cohort in Danish women. BJOG. 2009;116(1):98–107. doi:10.1111/j.1471-0528.2008.02001.x.
473. Owe KM, Nystad W, Bø K. Association between regular exercise and excessive newborn birth weight. Obstet Gynecol. 2009;114(4):770–6. doi:10.1097/AOG.0b013e3181b6c105.
474. Owe KM, Nystad W, Skjaerven R, et al. Exercise during pregnancy and the gestational age distribution: a cohort study. Med Sci Sports Exerc. 2012;44(6):1067–74. doi:10.1249/MSS.0b013 e3182442fc9.
475. Patel N, Godfrey KM, Pasupathy D, Levin J, Flynn AC, Hayes L, et al. Infant adiposity following a randomised controlled trial of a behavioural intervention in obese pregnancy. International Journal of Obesity 2017 Mar 21 [Epub ahead of print]. [DOI: 10.1038/ijo.2017.44
476. Patel NR, Pasupathy D, Flynn AC, Hayes L, Levin JG, Singh C, et al. The UPBEAT behavioural intervention in obese pregnant women - maternal and infant follow-up 6 months postpartum. Reproductive Sciences 2016;23(Suppl 1):71A.
477. Pawalia A., Kulandaivelan S., Savant S., Yadav V. Exercise in pregnancy: Effect on obesity parameters in indian women—A randomized controlled trial. Rom. J. Diabetes Nutr. Metab. Dis. 2017;24:315–323. doi: 10.1515/rjdnmd-2017-0037.
478. Pawlak DB. Glycemic load and infant birth weight in pregnant overweight/obese women. ClinicalTrials.gov (http://clinicaltrials.gov/) (accessed 31 October 2010).
479. Peaceman AM, Kwasny MJ, Gernhofer N, et al. MOMFIT: A randomized clinical trial of an intervention to prevent excess gestational weight gain in overweight and obese women. Am J Obstet Gynecol. 2017;216(1):S2–SS3.
480. Peccei A., Blake-Lamb T., Rahilly D., Hatoum I., Bryant A. Intensive prenatal nutrition counseling in a community health setting: A randomized controlled trial. Obstet. Gynecol. 2017;130:423–432. doi: 10.1097/AOG.0000000000002134.
481. Pelaez M, Gonzalez-Cerron S, Montejo R, Barakat R. Pelvic floor muscle training included in a pregnancy exercise program is effective in primary prevention of urinary incontinence: a randomized controlled trial. Neurourol Urodyn. 2014;33(1):67-71.

Diet and/or physical activity unique publications

482. Pellonpera¨ O, Mokkala K, Houttu N, Vahlberg T, Koivuniemi E, Tertti K, et al. Efficacy of fish oil and/or probiotic intervention on the incidence of gestational diabetes mellitus in an at-risk group of overweight and obese women: a randomized, placebo-controlled, double-blind clinical trial. Diabetes Care 2019;42:1009–17.
483. Perales M, Calabria I, Lopez C, Franco E, Coteron J, Barakat R. Regular exercise throughout pregnancy is associated with a shorter first stage of labor. Am J Health Promot. 2017; https://doi.org/10.4278/ajhp.140221- QUAN-79.
484. Perales M, Cordero Y, Vargas M, et al. Exercise and depression in overweight and obese pregnant women: a randomised controlled trial. Arch Medicina Deporte 2015;32(3):156–63.
485. Perales M, Mateos S, Vargas M, Sanz I, Lucia A, Barakat R. Fetal and maternal heart rate responses to exercise in pregnant women. A randomized controlled trial. Archivos de Medicina del Deporte. 2015;32(6):361-7.
486. Perales M, Refoyo I, Coteron J, Bacchi M, Barakat R. Exercise during pregnancy attenuates prenatal depression: a randomized controlled trial. Eval Health Prof 2015;38:59-72.
487. Perales M, Santos-Lozano A, Sanchis-Gomar F, Luaces M, Pareja-Galeano H, Garatachea N, et al. Maternal Cardiac Adaptations to a Physical Exercise Program during Pregnancy. Med Sci Sports Exerc. 2016;48(5):896-906.
488. Pérez-Ferre N, Galindo M, Fernández MD, Velasco V, Runkle I, de la Cruz MJ, et al. The outcomes of gestational diabetes mellitus after a telecare approach are not inferior to traditional outpatient clinic visits. Int J Endocrinol 2010;2010:386941 [FREE Full text] [doi: 10.1155/2010/386941] [Medline: 20628517]
489. Perichart PO, Balas NM, Parra CA, Rodriguez CA, Ramirez TA, Ortega GC, et al. A medical nutrition therapy programme improves perinatal outcomes in Mexican pregnant women with gestational diabetes and type 2 diabetes mellitus. Diabetes Educ 2009;35:1004–13.
490. Perichart-Perera O, Balas-Nakash M, Rodriguez-Cano A, Legorreta-Legorreta J, Parra-Covarrubias A, Vadillo-Ortega F (2012) Low glycemic index carbohydrates versus all types of carbohydrates for treating diabetes in pregnancy: a randomized clinical trial to evaluate the effect of glycemic control. Int J Endocrinol 2012:296017
491. Perkins CC, Pivarnik JM, Paneth N, et al. Physical activity and fetal growth during pregnancy. Obstet Gynecol. 2007;109(1): 81–7.
492. Peterson CD, Haas M, Gregory WT. A pilot randomized controlled trial comparing the efficacy of exercise, spinal manipulation, and neuro emotional technique for the treatment of pregnancy-related low back pain. Chiropr Man Therap. 2012;20(1):18.
493. Petrella E, Facchinetti F, Bertarini V, Pignatti L, Neri I, Battistini NC. Occurrence of pregnancy complications in women with BMI > 25 submitted to a healthy lifestyle and eating habits program. American Journal of Obstetrics and Gynecology 2013;208(1 Suppl):S33–4.
494. Petrella E., Malavolti M., Bertarini V., Pignatti L., Neri I., Battistini N.C., Facchinetti F. Gestational weight gain in overweight and obese women enrolled in a healthy lifestyle and eating habits program. J. Matern. Neonatal Med. 2014;27:1348–1352. doi: 10.3109/14767058.2013.858318.
495. Petrov Fieril K, Glantz A, Fagevik Olsen M. The efficacy of moderate-to-vigorous resistance exercise during pregnancy: a randomized controlled trial. Acta Obstet Gynecol Scand 2015;94:35–42.
496. Petrovic D, Perovic M, Lazovic B, Pantic I. Association between walking, dysphoric mood and anxiety in late pregnancy: A cross-sectional study. Psychiatry Res. 2016;246:360-3.
497. Phelan S, Phipps M G, Abrams B, Darroch F, Schaffner A, Wing R R. Randomized trial of a behavioral intervention to prevent excessive gestational weight gain: the Fit for Delivery Study. Am J Clin Nutr. 2011;93(04):772–779. [PMC free article] [PubMed] [Google Scholar]
498. Phelan S, Phipps MG, Abrams B, Darroch F, Grantham?K, Schaffner A, et al. Does behavioral intervention in pregnancy reduce postpartum weight retention? Twelve- month outcomes of the Fit for Delivery randomized trial. American Journal of Clinical Nutrition 2014;99(2):302–12.
499. Phelan S, Phipps MG, Abrams B, Darroch F, Schaffner A, Wing RR. Factors associated with success in the ”fit for delivery“ intervention to reduce excessive gestational weight gain. Obesity 2011;19(Suppl 1):S95.?
500. Phelan S., Wing R., Brannen A., McHugh A., Hagobian T., Schaffner A., Jelalian E., Hart C.N., Scholl T.O., Munoz-Christian K., et al. Randomized controlled clinical trial of behavioral lifestyle intervention with partial meal replacement to reduce excessive gestational weight gain. Am. J. Clin. Nutr. 2018;107:183–194. doi: 10.1093/ajcn/nqx043. [PMC free article]
501. Piirainen T, Isolauri E, Lagstrom H, Laitinen K. Impact of dietary counselling on nutrient intake during pregnancy: a prospective cohort study. British Journal of Nutrition 2006; 96(6):1095–104
502. Pinzón DC, Zamora K, Martínez JH, Floréz-López ME, dePlata AC, Mosquera M, et al. Type of delivery and gestational age is not affected by pregnant Latin-American women engaging in vigorous exercise: a secondary analysis of data from a controlled randomized trial. Rev Salud Publica (Bogota)2012;14:731–43.
503. Piravej K, Saksirinukul R. Survey of patterns, attitudes, and the general effects of exercise during pregnancy in 203 Thai pregnant women at King Chulalongkorn Memorial Hospital. J Med Assoc Thai. 2001;84 Suppl 1:S276-82.
504. Pollak KI, Alexander SC, Bennett G, Lyna P, Coffman CJ, Bilheimer A, et al. Weight-related SMS texts promoting appropriate pregnancy weight gain: a pilot study. Patient Educ Couns 2014 Nov;97(2):256-260 [FREE Full text] [doi: 10.1016/j.pec.2014.07.030] [Medline: 25153313]
505. Polley B, Wing R, Sims C. Randomised controlled trial to prevent excessive weight gain in pregnant women. Int J Obesity. 2002;26:1494–502.
506. Pomerance JJ, Gluck L, Lynch VA. Physical fitness in pregnancy: its effect on pregnancy outcome. American Journal of Obstetrics & Gynecology. 1974;44(3):383-7.
507. Portela SN, Rocha-de-Souza R, Oppermann-Lisboa K, et al. Maternal physical activity, cervical length and its relation to spontaneous vaginal birth at term. Arch Gynecol Obstet. 2014;290(2):257–62. doi:10.1007/s00404-014-3198-4.

Diet and/or physical activity unique publications

508. Poston L, Briley AL, Barr S, Bell R, Croker H, Coxon K, et al. Developing a complex intervention for diet and activity behaviour change in obese pregnant women (the UPBEAT trial); assessment of behavioural change and process evaluation in a pilot randomised controlled trial. BMC Pregnancy and Childbirth 2013;13(1):148.
509. Poston L, Holmes B, Kinnunen T, Croker H, Bell R, Sanders T, et al. A complex intervention to improve outcome in obese pregnancies; the upbeat study. Archives of Disease in Childhood: Fetal and Neonatal Edition 2011;96 (Suppl 1):Fa97.?
510. Poston L. The UPBEAT study: A lifestyle intervention in obese pregnant women. The Power of Programming 2014: International Conference on Developmental Origins of Adiposity and Long-Term Health; 2014 March 13-15; Munich, Germany. 2014:22.?
511. Poston, L., Bell R, Croker H, Flynn AC, Godfrey KM, Goff L, Hayes L, Khazaezadeh N, Nelson SM, Oteng-Ntim E, Pasupathy D, Patel N, Robson SC, Sandall J, Sanders TAB, Sattar N, Seed PT, Wardle J, Whitworth MK, Briley AL on behalf of The UPBEAT Trial Consortium, Effect of a behavioural intervention in obese pregnant women (the UPBEAT study): a multicentre, randomised controlled trial, Lancet Diabetes Endocrinol, 2015, doi: 10.1016/S2213-8587(15)00227-2.
512. Prabhu N. Effect of exercise by pregnant women and birth weight: a randomized controlled trial. Journal of Evolution of Medical and Dental Sciences 2015;04:1509–
513. Prevedel T, Calderon I, De Conti M, Consonni E, Rudge M. Maternal and perinatal effects of hydrotherapy in pregnancy. RBGO 2003; 25:53–9.
514. Price BB, Saeid B, Kappeper K. Exercise in pregnancy: effect on fitness and obstetric outcomes-A randomized trial. Med Sci Sports Exerc 2012;44:2263–9.
515. Puhkala J, Luoto R, Ahotupa M, Raitanen J, Vasankari T. Postpartum weight retention is associated with elevated ratio of oxidized LDL lipids to HDL-cholesterol. Lipids 2013;48(12):1227–35.
516. Puhkala J, Raitanen J, Kolu P, Tuominen P, Husu P, Luoto R. Metabolic syndrome in Finnish women 7 years after a gestational diabetes prevention trial. BMJ Open. 2017;7(3):e014565.
517. Putnam KF, Mueller LA, Magann EF, Thagard A, Johnson AM, Ounpraseuth ST, et al. Evaluating effects of self-reported domestic physical activity on pregnancy and neonatal outcomes in "stay at home" military wives. Mil Med. 2013;178(8):893-8.
518. Quinlivan J A, Lam L T, Fisher J. A randomised trial of a four-step multidisciplinary approach to the antenatal care of obese pregnant women. Aust N Z J Obstet Gynaecol. 2011;51(02):141–146. [PubMed] [Google Scholar]
519. Quinlivan J. A randomised trial of a multidisciplinary teamcare approach involving obstetric, dietary and clinical psychological input in obese pregnant women to reduce the incidence of gestational diabetes. https:// www.anzctr.org.au/Trial/Registration/TrialReview.aspx?id= 821 (accessed 2 November 2015)
520. Rae A, Bond D, Evans S, North F, Roberman B, Walters B. A randomised controlled trial of dietary energy restriction in the management of obese women with gestational diabetes. Aust N Z J Obstet Gynaecol. 2000;40:416–22.
521. Rakhshani A, Nagarathna R, Mhaskar R, Mhaskar A, Thomas A, Gunasheela S. The effects of yoga in prevention of pregnancy complications in high-risk pregnancies: a randomized controlled trial. Prev Med 2012; 55: 333–40 PubMed PMID: 22884667.
522. Ramirez-Velez R, Aguilar de Plata AC, Escudero MM, Echeverry I, Ortega JG, Salazar B, et al. Influence of regular aerobic exercise on endothelium-dependent vasodilation and cardiorespiratory fitness in pregnant women. Journal of Obstetrics and Gynaecology Research 2011;37(11):1601–8.
523. Ramirez-Velez R. Aerobic exercise in pregnant Latina women: Effect on metabolic and body composition outcomes. A randomized clinical trial. FASEB Journal 2014;28(1 Suppl 1):886.6.
524. Ramirez-Velez R. Combined aerobic and resistance exercise on metabolic and body composition outcomes in primigravid Latina women. Obesity Reviews 2014;15(Suppl2):201–2.
525. Ramírez-Vélez R, Aguilar de Plata AC, Mosquera-Escudero M, BS, et al. The effect of aerobic exercise on oxygen consumption in healthy first pregnancy females: a randomized clinical trial. Rev Colomb Obstet Ginecol 2011;62:15–23.
526. Ramos JG, Bgeginski R, Opperman ML, Martins-Costa S, Delevatti R, Schuch R, et al. Effect of aerobic training in pregnant women diagnosed with gestational diabetes: a preliminary report. Pregnancy Hypertension. 2015;5(1):105.
527. Rauh 2013 DRKS00003801. Feasibility of a lifestyle intervention?in pregnancy to optimize maternal weight development. drks-neu.uniklinik-freiburg.de/drks web/navigate.do?navigationId=trial.HTML&TRIAL ID=DRKS00003801 (first received 24 April 2012).?
528. Rauh K, Gabriel E, Kerschbaum E, et al. Safety and efficacy of a lifestyle intervention for pregnant women to prevent excessive maternal weight gain: a cluster randomized controlled trial. BMC Pregnancy Childbirth 2013;13:151.
529. Rauh K, Günther J, Kunath J, et al. Lifestyle intervention to prevent excessive maternal weight gain: mother and infant follow-up at 12 months postpartum. BMC Pregnancy Childbirth 2015;15:265.
530. Reilly ET, Freeman RM, Waterfield MR, Waterfield AE, Steggles P, Pedlar F. Prevention of postpartum stress incontinence in primigravidae with increased bladder neck mobility: a randomised controlled trial of antenatal pelvic floor exercises. Bjog. 2002;109(1):68-76.
531. Renault K., Nørgaard K., Nilas L., Carlsen E., Cortes D., Pryds O., Secher N.J. The Treatment of Obese Pregnant Women (TOP) study: A randomized controlled trial of the effect of physical activity intervention assessed by pedometer with or without dietary intervention in obese pregnant women. Am. J. Obstet. Gynecol. 2014;210:134. doi: 10.1016/j.ajog.2013.09.029.
532. Renault KM, Carlsen EM, Nørgaard K, et al. Intake of sweets, snacks and soft drinks predicts weight gain in obese pregnant women: detailed analysis of the results of a randomised controlled trial. PLoS One 2015;10:e0133041.
533. Restall A, Taylor RS, Thompson JM, et al. Risk factors for excessive gestational weight gain in a healthy, nulliparous cohort. J Obes. 2014;2014:148391. doi:10.1155/2014/148391.

Diet and/or physical activity unique publications

534. Rhodes E., Pawlak D., Takoudes T., Ebbeling C., Feldman H., Lovesky M., Cooke E.A., Leidig M.M., Ludwig D.S. Effects of a low-glycemic load diet in overweight and obese pregnant women: A pilot randomized controlled trial. Am. J. Clin. Nutr. 2010;92:1306–1315. doi: 10.3945/ajcn.2010.30130.
535. Rice PL, Fort IL. The relationship of maternal exercise on labor, delivery and health of the newborn. J Sports Med Phys Fitness. 1991;31(1):95-9.
536. Robledo-Colonia AF, Sandoval-Restrepo N, Mosquera-Valderrama YF, Escobar-Hurtado C, Ramirez-Velez R. Aerobic exercise training during pregnancy reduces depressive symptoms in nulliparous women: a randomised trial. J Physiother. 2012;58(1):9-15.
537. Rodríguez YC, Puente MP, Abad MDM, RBC, et al. Can moderate physical exercise during pregnancy act as a factor in preventing Gestational Diabetes? Rev Int Cienc Deporte 2012;8:3–19.
538. Rodríguez-Blanque R., Carlos Sánchez-García J., Manuel Sánchez-López A., Mur-Villar N., Fernández-Castillo R., José Aguilar-Cordero M. Influence of physical exercise during pregnancy on birthweight: A randomized clinical trial. Nutr. Hosp. 2017;34:834–840. doi: 10.20960/nh.1095.
539. Ronnberg A, Hanson U, Ostlund I, Nilsson K. Effects on postpartum weight retention after antenatal lifestyle intervention—a secondary analysis of a randomized controlled trial. Acta Obstet Gynecol Scand. 2016;95(9):999-1007.
540. Ronnberg A., Ostlund I., Fadl H., Gottvall T., Nilsson K. Intervention during pregnancy to reduce excessive gestational weight gain-a randomised controlled trial. BJOG. 2015;122:537–544. doi: 10.1111/1471-0528.13131.
541. Rono K, Stach-Lempinen B, Klemetti MM, Kaaja RJ, Poyhonen-Alho M, Eriksson JG, et al. Prevention of gestational diabetes through lifestyle intervention: study design and methods of a Finnish randomized controlled multicenter trial (RADIEL). BMC Pregnancy and Childbirth 2014;14:70.
542. Ross SM, Nel E, Naeye RL. Differing effects of low and high bulk maternal dietary supplements during pregnancy. Early Hum Dev 1985;10:295–302.
543. Ruchat S, Davenport MH, Giroux I, Hillier M, Batada A, Sopper MM et al. Nutrition and exercise reduce excessive weight gain in normal-weight pregnant women. Med Sci Sports Exerc. 2012;44:1419-26.
544. Ruchat SM, Davenport MH, Giroux I, Hillier M, Batada A, Sopper MM, et al. Effect of exercise intensity and duration on capillary glucose responses in pregnant women at low and high risk for gestational diabetes. Diabetes Metab Res Rev. 2012;28(8):669-78.
545. Rudra CB, Sorensen TK, Luthy DA, et al. A prospective analysis of recreational physical activity and preeclampsia risk. MedSci Sports Exerc. 2008;40(9):1581–8. doi:10.1249/MSS. 0b013e31817cab1.
546. Ruiz J R, Perales M, Pelaez M, Lopez C, Lucia A, Barakat R. Supervised exercise-based intervention to prevent excessive gestational weight gain: a randomized controlled trial. Mayo Clin Proc. 2013;88(12):1388–1397. [PubMed] [Google Scholar]
547. Rush D, Stein Z, Susser M. A randomized controlled trial of prenatal nutritional supplementation in New York City. Pediatrics 1980;65:683–97.
548. Saftlas AF, Logsden-Sackett N, Wang W, Woolson R, Bracken MB. Work, leisure-time physical activity, and risk of preeclampsia and gestational hypertension. Am J Epidemiol. 2004;160(8):758-65.
549. Sagedal L., Øverby N., Bere E., Torstveit M., Lohne-Seiler H., Småstuen M., Hillesund E.R., Henriksen T., Vistad I. Lifestyle intervention to limit gestational weight gain: The Norwegian Fit for Delivery randomised controlled trial. BJOG. 2016;124:97–109. doi: 10.1111/1471-0528.13862.
550. Sagedal LR, Henriksen T, Overby NC, Lohne-Seiler?H, Torstveit MK, Bere E, et al. The problem of non- participation: Who declined to participate in ”Fit for Delivery“, a randomized, controlled trial of a lifestyle intervention in pregnancy?. The Power of Programming 2014: International Conference on Developmental Origins of Adiposity and Long-Term Health; 2014 March 13-15; Munich, Germany. 2014:63.?
551. Sagedal LR, Overby N, Lohne-Seiler H, Bere E, Torstveit M, Henriksen T, et al. Study protocol: Fit for Delivery - can a lifestyle intervention in pregnancy result in measurable health benefits for mothers and newborns? A randomized controlled trial. BMC Public Health 2013;13:132.?
552. Sagedal LR, Sanda B, Overby NC, Bere E, Torstveit MK, Lohne-Seiler H, et al. The effect of prenatal lifestyle intervention on weight retention 12 months postpartum: results of the Norwegian Fit for Delivery randomised controlled trial. BJOG: an international journal of obstetrics and gynaecology 2017;124(1):111–21.
553. Sagedal LR, Vistad I, Øverby NC, et al. The effect of a prenatal lifestyle intervention on glucose metabolism: results of the Norwegian fit for delivery randomized controlled trial. BMC Pregnancy Childbirth 2017;17:167.
554. Saila B. Koivusalo, Kristiina Rönö, Miira M. Klemetti, Risto P. Roine, Jaana Lindström, et al. Gestational Diabetes Mellitus Can Be Prevented by Lifestyle Intervention: The Finnish Gestational Diabetes Prevention Study (RADIEL) Diabetes Care Jan 2016, 39 (1) 24-30; DOI: 10.2337/dc15-0511
555. Salvesen KA, Stafne SN, Eggebo TM, Morkved S. Does regular exercise in pregnancy influence duration of labor? A secondary analysis of a randomized controlled trial. Acta Obstet Gynecol Scand. 2014;93(1):73-9.
556. Salvig J, Olsen S, Secher N. Effects of fish oil supplementation in late pregnancy on blood pressure: a randomised controlled trial. BJOG. 1996;103:529–33.
557. Santaella MP. The role of a supervised physical exercise program as an alternative on the control of maternal gestational weight gain. ClinicalTrials.gov (accessed 21 May20 13). NCT01790347 2013.
558. Santamaria A., Di Benedetto A., Petrella E., Pintaudi B., Corrado F., D'Anna R., Neri I., Facchinetti F. Myo-inositol may prevent gestational diabetes onset in overweight women: A randomized, controlled trial. J. Matern. Fetal Neonatal Med. 2016;29:3234–3237. doi: 10.3109/14767058.2015.1121478.

Diet and/or physical activity unique publications

559. Santos IA, Stein R, Fuchs SC, Duncan BB, Ribeiro JP, Kroeff LR, et al. Aerobic exercise and submaximal functional capacity in overweight pregnant women: a randomised trial. Obstet Gynecol 2005;106:243–9.
560. Satyapriya M, Nagarathna R, Padmalatha V, Nagendra HR. Effect of integrated yoga on anxiety, depression & well being in normal pregnancy. 2013.
561. Savitha Pramilda C, Raj G, Rani GS. Effect of Antenatal Yoga on Postnatal Depressive Symptoms among the Primipara Women of a Selected Hospital. International Journal of Nursing Education. 2016;8(1):182.
562. Schlaff RA, Holzman C, Maier KS, et al. Associations among gestational weight gain, physical activity, and pre-pregnancy body size with varying estimates of pre-pregnancy weight. Midwifery. 2014;30(11):1124–31. doi:10.1016/j.midw.2014.03.014.
563. Schneeberger C, Flynn A, Barr S, Seed PT, Inskip HM, Poston L. Maternal diet patterns and glycaemic load in obese pregnant women taking part in a pilot trial of a lifestyle intervention (the upbeat trial). Diabetes 2014; Vol. 63:A342.
564. Sealy-Jefferson S, Hegner K, Misra DP. Linking non-traditional physical activity and preterm delivery in urban African-American women. Womens Health Issues. 2014;24(4):e389–95. doi:10.1016/j.whi.2014.04.007.
565. Sedaghati P, Ziaee V, Ardjmand A. The effect of an ergometric training program on pregnants weight gain and low back pain. Gazz Med Ital- Arch Sci Med 2007;166:209–13
566. Seed PT, Briley A, Singh C, Patel N, Poston L. A novel method for devising optimal gestational weight gain in obese pregnant women. Reproductive Sciences 2016; Vol. 23, issue 1 Suppl 1:276A.?
567. Seneviratne S, Jiang Y, Derraik J, McCowan L, Parry G, Biggs J, et al. Effects of antenatal exercise in overweight and obese pregnant women on maternal and perinatal outcomes: a randomised controlled trial. BJOG Int J Obstet Gynaecol. 2016;123(4):588–97.
568. Sexton MB, Flynn HA, Lancaster C, et al. Predictors of recovery from prenatal depressive symptoms from pregnancy through postpartum. 2012.
569. Shim MJ, Lee YS, Oh HE, Kim JS. Effects of a back-pain-reducing program during pregnancy for Korean women: a non-equivalent control-group pretest-posttest study. Int J Nurs Stud. 2007;44(1):19-28.
570. Shirazian T, Monteith S, Friedman F, Rebarber A. Lifestyle modification program decreases pregnancy weight gain in obese women. Am J Perinat 2010; 27: 411–414
571. Shub A. Diet and exercise in pregnancy. Australian Clinical Trials Registry (www.actr.org.au/) [accessed 21 June 2007].
572. Shyam S, Arshad F, Abdul Ghani R, Wahab NA, Safii NS, Nisak MYB, et al. Low glycaemic index diets improve glucose tolerance and body weight in women with previous history of gestational diabetes: a six months randomized trial. Nutr J 2013;12:68-.
573. Simmons D, Devlieger R, Assche Av, Jans G, Galjaard S, Corcoy R, et al. Effect of physical activity and/or healthy eating on GDM risk: The DALI Lifestyle Study. Journal of Clinical Endocrinology & Metabolism. 2016.
574. Simmons D, Jelsma JG, Galjaard S, Devlieger R, van Assche A, Jans G, et al. Results From a European Multicenter Randomized Trial of Physical Activity and/or Healthy Eating to Reduce the Risk of Gestational Diabetes Mellitus: The DALI Lifestyle Pilot. Diabetes Care. 2015;38(9):1650-6.
575. Sklempe Kokic I, Ivanisevic M, Biolo G, Simunic B, Kokic T, Pisot R. Combination of a structured aerobic and resistance exercise improves glycaemic control in pregnant women diagnosed with gestational diabetes mellitus. A randomised controlled trial. Women and birth. 2017; https://doi. org/10.1016/j.wombi.2017.10.004.
576. Skouteris H., McPhie S., Hill B., McCabe M., Milgrom J., Kent B., Bruce L., Herring S., Gale J., Mihalopoulos C., et al. Health coaching to prevent excessive gestational weight gain: A randomized-controlled trial. Br. J. Health Psychol. 2016;21:31–51. doi: 10.1111/bjhp.12154.
577. Smith K, Lanningham-Foster L, Welch A, Campbell C. Web-based behavioral intervention increases maternal exercise but does not prevent excessive gestational weight gain in previously sedentary women. J Phys Act Health 2016 Jun;13(6):587-593. [doi: 10.1123/jpah.2015-0219] [Medline: 26594820]
578. Smith KM. The Blossom Project Online: use of a behaviorally based website to promote physical activity and prevent excessive gestational weight gain in previously sedentary pregnant women. Graduate Theses and Dissertations 2014: 14051
579. Smuts CM, Borod E, Peeples JM, Carlson SE. High-DHA eggs: feasibility as a means to enhance circulating DHA in mother and infant. Lipids 2003;38:407–14.
580. Smuts CM, Huang M, Mundy D, Plasse T, Major S, Carlson SE. A randomised trial of docosahexaenoic acid supplementation during the third trimester of pregnancy. Obstet Gynecol. 2003;101:469–79.
581. Snapp CA, Donaldson SK. Gestational diabetes mellitus: physical exercise and health outcomes. Biol Res Nurs. 2008;10(2):145-55.
582. Soltani H, Duxbury AM, Arden MA, Dearden A, Furness PJ, Garland C. Maternal obesity management using mobile technology: a feasibility study to evaluate a text messaging based complex intervention during pregnancy. J Obes 2015;2015:814830 [FREE Full text] [doi: 10.1155/2015/814830] [Medline: 25960889]
583. Songoygard KM, Stafne SN, Evensen KA, Salvesen KA, Vik T, Morkved S. Does exercise during pregnancy prevent postnatal depression? A randomized controlled trial. Acta Obstet Gynecol Scand. 2012;91(1):62-7.
584. Sorensen TK, Williams MA, Lee IM, Dashow EE, Thompson ML, Luthy DA. Recreational physical activity during pregnancy and risk of preeclampsia. Hypertension 2003;41:1273–80.
585. Soultanakis HN, Artal R, Wiswell RA. Prolonged exercise in pregnancy: glucose homeostasis, ventilatory and cardiovascular responses. Semin Perinatol. 1996;20(4):315-27.

Diet and/or physical activity unique publications

586. Spracklen CN, Ryckman KK, Triche EW, Saftlas AF. Physical Activity During Pregnancy and Subsequent Risk of Preeclampsia and Gestational Hypertension: A Case Control Study. Matern Child Health J. 2016;20(6):1193-202.
587. Stafne SN, Salvesen K A, Romundstad PR, Eggebø TM, Carlsen SM, Mørkved S. Regular exercise during pregnancy to prevent gestational diabetes: a randomized controlled trial. Obstet Gynecol. 2012;119:29–36.
588. Stafne SN, Salvesen KA, Romundstad PR, Torjusen IH, Mørkved S. Does regular exercise including pelvic floor muscle training prevent urinary and anal incontinence during pregnancy? A randomised controlled trial. BJOG 2012;119:1270-80.
589. Stein AD, Kahn HS, Rundle A, Zybert PA, van der Pal-de Bruin, Lumey LH. Anthropometric measures in middle age after exposure to famine during gestation: evidence from the Dutch famine. Am J Clin Nutr 2007;85:869–76.
590. Stendell-Hollis NR, Thompson PA, West JL, Wertheim BC, Thomson CA. A comparison of Mediterranean-style and MyPyramid diets on weight loss and inflammatory biomarkers in postpartum breastfeeding women. J Womens Health 2013;22(1):48–57.
591. Sternfeld B, Quesenberry CP, Eskenazi B, et al. Exercise during pregnancy and pregnancy outcome. Med Sci Sports Exerc. 1995;27(5):634–40.
592. Strom M, Mortensen EL, Halldorson TI, Osterdal ML, Olsen SF. Leisure-time physical activity in pregnancy and risk of postpartum depression: a prospective study in a large national birth cohort. J Clin Psychiatry. 2009;70(12):1707-14.
593. Stuebe AM, Oken E, Gillman MW. Associations of diet and physical activity during pregnancy with risk for excessive gestational weight gain. Am J Obstet Gynecol. 2009;201(1): 58.e1–8. doi:10.1016/j.ajog.2009.02.025.
594. Stutzman SS, Brown CA, Hains SM, Godwin M, Smith GN, Parlow JL, et al. The effects of exercise conditioning in normal and overweight pregnant women on blood pressure and heart rate variability. Biol Res Nurs. 2010;12:137–48.
595. Sui Z, Yelland LN, Turnbull D, Dodd JM. Walking to limit gestational weight gain and keep fit during pregnancy - findings from the walk randomised trial. Journal of Paediatrics and Child Health 2013;49 Suppl 2:120.?
596. Sun Y, Zhao H. The effectiveness of lifestyle intervention in early pregnancy to prevent gestational diabetes mellitus in Chinese overweight and obese women: a quasiexperimental study. Appl Nurs Res 2016;30:125–30.
597. Surita F. Physical exercise influence among overweightand obese pregnant women. ClinicalTrials.gov (http://clinicaltrials.gov/) [accessed 21 May 2013] 2010.
598. Syngelaki A, Nicolaides KH, Balani J, Hyer S, Akolekar R, Kotecha R, et al. Metformin versus placebo in obese pregnant women without diabetes mellitus. N Engl J Med 2016;374(5):434–43.
599. Szmeja M A, Cramp C, Grivell R M, Deussen A R, Yelland L N, Dodd J M. Use of a DVD to provide dietary and lifestyle information to pregnant women who are overweight or obese: a nested randomised trial. BMC Pregnancy Childbirth. 2014;14:409. [PMC free article] [PubMed] [Google Scholar]
600. Szmeja MA, Grivell RM, Deussen AR, Dodd JM. Evaluation of information provision to women who are overweight or obese during pregnancy. Journal of Paediatrics Germany. 2014:22–3.
601. Takito MY, Benicio MH, Latorre MR. Maternal posture and its influence on birthweight. Rev Saude Publica. 2005;39(3): 325–32.
602. Taniguchi C, Sato C. Home-based walking during pregnancy affects mood and birth outcomes among sedentary women: a randomized controlled trial. Int J Nurs Pract 2016;22:420–6.
603. Tanvig M. Lifestyle in pregnancy and offspring - comparison between children born to obese women and children born to normal weight women (LiPO). ClinicalTrials.gov (http:// clinicaltrials.gov/) [accessed 6 February 2014] 2013.
604. Tanvig M. Offspring body size and metabolic profile - Effects of lifestyle intervention in obese pregnant women. Danish Medical Journal 2014;61(7):B4893.
605. Tanvig M, Vinte CA, Jorgensen JS, Wehberg S, Ovesen PG, Lamont RF, et al. Anthropometrics and body composition by dual energy X-ray in children of obese women: a follow-up of a randomized controlled trial (the Lifestyle in Pregnancy and Offspring [LiPO] study). PLOS One 2014;9 (2):e89590.
606. Tanvig M, Vinter CA, Jorgensen JS, Wehberg S, Ovesen PG, Beck-Nielsen H, et al. Effects of lifestyle intervention in pregnancy and anthropometrics at birth on offspring metabolic profile at 2.8 years - results from the Lifestyle in Pregnancy and Offspring (LiPO) study. Journal of Clinical Endocrinology and Metabolism 2015;100(1):175–83.?
607. Tavares JS, Melo AS, Amorim MM, et al. Association between maternal physical activity, gestational weight gain and birth weight in a cohort of 118 pregnant women in Campina Grande, Northeast of Brazil. Rev Assoc Med Bras. 2009;55(3):335–41.
608. Teede HJ, Harrison CL, Gibson-Helm M, Lombard CB. Improving physical activity in high-risk pregnancies: A randomized controlled trial. Endocrine Reviews 2011;32(3 Meeting Abstracts):P1–467.
609. Tendais I, Figueiredo B, Mota J, Conde A. Physical activity, health-related quality of life and depression during pregnancy. Cad Saude Publica. 2011;27(2):219-28.
610. Thomson J., Tussing-Humphreys L., Goodman M., Olender S. Gestational weight gain: Results from the delta healthy sprouts comparative impact trial. J. Pregnancy. 2016;2016:12. doi: 10.1155/2016/5703607.
611. Thornton Y, Smarkola C, Kopacz SM, Ishoof S. Perinatal outcomes in nutritionally monitored obese pregnant women: a randomised clinical trial. J Natl Med Assoc. 2009;101:569–77.
612. Tinloy J, Chuang CH, Zhu J, et al. Exercise during pregnancy and risk of late preterm birth, cesarean delivery, and hospitalizations. Womens Health Issues. 2014;24(1):e99–104. doi:10. 1016/j.whi.2013.11.003.

Diet and/or physical activity unique publications

613. To WW, Wong MW. Bone mineral density changes during pregnancy in actively exercising women as measured by quantitative ultrasound. Arch Gynecol Obstet. 2012;286(2):357-63.
614. Tomic V, Sporis G, Tomic J, et al. The effect of maternal exercise during pregnancy on abnormal fetal growth. Croat Med J. 2013; 54:362-368.
615. Uebelacker LA, Battle CL, Sutton KA, Magee SR, Miller IW. A pilot randomized controlled trial comparing prenatal yoga to perinatal health education for antenatal depression. Arch Womens Ment Health. 2016;19(3):543-7.
616. Umpierrez G. Lifestyle intervention to limit excessive weight gain during pregnancy in minority women. ClinicalTrials.gov (accessed 21 May 2013). NCT01084941 2010.
617. Vahamiko S, Isolauri E, Laitinen K. Weight status and dietary intake determine serum leptin concentrations in pregnant and lactating women and their infants. British Journal of Nutrition 2013;110(6):1098–106
618. Valkama A, Koivusalo S, Lindstrom J, Meinila J, Kautiainen H, Stach-Lempinen B, et al. The effect of dietary counselling on food intakes in pregnant women at risk for gestational diabetes: a secondary analysis of a randomised controlled trial RADIEL. European Journal of Clinical Nutrition 2016;70(8):912–7.
619. Van Poppel M, Oostdam N, Wouters M, Eekhoff M, Van Mechelen W. FitFor2: Effects of an exercise training program on the incidence of gestational diabetes. Journal of Science and Medicine in Sport 2012;15 Suppl 1:S342–S343.
620. Van Buul BJA, Steegers EAP, Van Der Maten GD, Delemarre FMC, Jongsma HW, Oosterbaan HP, De Jong PA. Dietary sodium restriction does not prevent gestational hypertension: a Dutch two-center randomized trial. Hypertens Pregnancy 1997;16:335–46
621. Vanky E, Salvesen KA, Heimstad R, et al. Metformin reduces pregnancy complications without affecting androgen levels in pregnant polycystic ovary syndrome women: results of a randomized study. Hum Reprod 2004; 19: 1734–1740.
622. Vanky E, Solhild S, Hemistad R, et al. Metformin versus placebo from First Trimester to Delivery in Polycystic Ovary Syndrome: a randomized, controlled multicenter study. J Clin Endocrinol Metab 2010; 95: E448–E455.
623. Vesco KK, Karanja N, King JC, Gillman MW, Perrin N, McEvoy C, et al. Healthy Moms, a randomized trial to promote and evaluate weight maintenance among obese pregnant women: study design and rationale. Contemporary Clinical Trials 2012;33(4):777–85.
624. Vesco K K, Karanja N, King J C et al.Efficacy of a group-based dietary intervention for limiting gestational weight gain among obese women: a randomized trial. Obesity (Silver Spring) 2014;22(09):1989–1996. [PMC free article] [PubMed] [Google Scholar]
625. Vesco K, Leo M, Gillman M, King J, McEvoy C, et al. (2013) Impact of a weight management intervention on pregnancy outcomes among obese women: The Healthy Moms Trial. Am J Obstet Gynecol 208(1):S352.
626. Vesco K, Leo M, Karanja N, et al. One-year postpartum outcomes following a weight management intervention in pregnant women with obesity. Obesity. 2016;24(10):2042-2049.
627. Vieira MC, Pasupathy D, Patel NR, White SL, Briley A, Seed PT, et al. Factors associated with uncomplicated pregnancy in obese women from the UPBEAT trial. Reproductive Sciences 2016;23(Suppl 1):109A.?White L, Pasupathy D, Vieira MC, Briley AL, Seed P, Lawlor DA, et al. Prediction of gestational diabetes (GDM) in obese women. Reproductive Sciences 2016;23(Suppl 1): 124A.
628. Vieten C, Astin J. Effects of a mindfulness-based intervention during pregnancy on prenatal stress and mood: results of a pilot study. Arch Womens Ment Health. 2008;11(1):67-74.
629. Vinter A. Lifestyle and pregnancy: the clinical effect of lifestyle intervention during pregnancy in obese women. ClinicalTrials.gov(www.clinicaltrials.gov) [accessed 31 October 2010].
630. Vinter CA, Jensen DM, Ovesen P, Beck-Nielsen H, Jorgensen JS. The LiP (Lifestyle in Pregnancy) study: a randomized controlled trial of lifestyle intervention in 360 obese pregnant women. Diabetes Care 2011;34(12):2502–7.
631. Vinter C, Jensen D, Ovesen P, Beck-Nielsen H, Lamont R, Jorgensen J. Postpartum weight retention and breastfeeding among obese women from the LiP (Lifestyle in Pregnancy) Study. Acta Obstetricia et Gynecologica Scandinavica 2012; 91(Suppl 159):141–2.
632. Vinter C, Jensen DM, Ovesen P, Beck-Nielsen H, Jorgensen JS (2011) The LiP (Lifestyle in Pregnancy) study: a randomized controlled trial of lifestyle intervention in 360 obese pregnant women. Diabetes Care 34(12):2502–7. doi: 10.2337/dc11-1150 PMID: 21972411.
633. Vinter CA, Jensen DM, Ovesen PG, Beck-Nielsen H, Jorgensen JS. Lifestyle and pregnancy (LIP) study: The clinical effect of lifestyle intervention during pregnancy in obese women. Diabetes 2011;60:A348–9.
634. Vinter CA, Jørgensen JS, Ovesen P, Beck-Nielsen H, Skytthe A, Jensen DM. Metabolic effects of lifestyle intervention in obese pregnant women. results from the randomized controlled trial ’lifestyle in pregnancy’ (LiP). Diabetic Medicine 2014;31(11):1323–30.
635. Vitolo MR. Impact of a nutritional intervention program for weight control during pregnancy. ClinicalTrials.gov (www.clinicaltrials.gov) [accessed 31 October 2010].
636. Vítolo M., Bueno M., Gama C. Impact of a dietary counseling program on the gain weight speed of pregnant women attended in a primary care service. Rev. Bras. Ginecol. Obstet. 2011;33:58–59. doi: 10.1590/S0100-72032011000100009.
637. Vollebregt KC, Wolf H, Boer K, van der Wal MF, Vrijkotte TGM, Bonsel GJ. Does physical activity in leisure time early in pregnancy reduce the incidence of preeclampsia or gestational hypertension? Acta Obstetricia Et Gynecologica Scandinavica. 2010;89(2):261-7.

Diet and/or physical activity unique publications

638. Wallace AM, Boyer DB, Dan A, Holm K. Aerobic exercise, maternal self-esteem, and physical discomforts during pregnancy. J Nurse Midwifery. 1986;31(6):255-62.
639. Walsh J M, McAuliffe F M. Impact of maternal nutrition on pregnancy outcome--does it matter what pregnant women eat? Best Pract Res Clin Obstet Gynaecol. 2015;29(01):63–78. [PubMed] [Google Scholar]
640. Walsh J, Mahony R, Foley M, Mc Auliffe F. A randomised control trial of low glycaemic index carbohydrate diet versus no dietary intervention in the prevention of recurrence of macrosomia. BMC Pregnancy and Childbirth 2010;10:16
641. Walsh J, Mahony R, Foley M, McAuliffe F. ROLO study: a randomized control trial of low glycemic index diet to prevent macrosomia in euglycemic women. American Journal of Obstetrics and Gynecology 2012;206(Suppl 1):S4
642. Walsh J, McGowan C, Byrne J, Foley M, Mahony R, McAuliffe F. The influence of a low glycaemic index dietary intervention on maternal glycaemic index, dietary intake and gestational weight gain. American Journal of Obstetrics and Gynecology 2013;208(1 Suppl):S33
643. Walsh J., McGowan C., Mahony R., Foley M., McAuliffe F. Low glycaemic index diet in pregnancy to prevent macrosomia (ROLO study): Randomised control trial. BMJ. 2012;345:e5605. doi: 10.1136/bmj.e5605.
644. Walsh JM, Mahony RM, Canty G, Foley ME, McAuliffe FM. Identification of those most likely to benefit from a low-glycaemic index dietary intervention in pregnancy. British Journal of Nutrition 2014;112:583–9
645. Walsh JM, Mahony RM, Culliton M, Foley ME, McAuliffe FM. Impact of a low glycemic index diet in pregnancy on markers of maternal and fetal metabolism and inflammation. Reproductive Sciences 2014;21(11):1378–81
646. Wang C, Wei Y, Zhang X, et al. A randomized clinical trial of exercise during pregnancy to prevent gestational diabetes mellitus and improve pregnancy outcome in overweight and obese pregnant women. Am J Obstet Gynecol 2017;216:340–51.
647. Wang C, Wei Y, Zhang X, Zhang Y, Xu Q, Su S et al (2016) Effect of regular exercise commenced in early pregnancy on the incidence of gestational diabetes mellitus in overweight and obese pregnant women: a randomized controlled trial. Diabetes Care 39:e163–e164
648. Wang S, Ma JM, Yang HX. Lifestyle intervention for gestational diabetes mellitus prevention: A cluster- randomized controlled study. Chronic Diseases and Translational Medicine 2015;1(3):169–74.
649. Wang X, Li GY, Deng ML. Pelvic floor muscle training as a persistent nursing intervention: Effect on delivery outcome and pelvic floor myodynamia. International Journal of Nursing Sciences. 2014;1(1):48-52.
650. Wang Y, Li A, Yang H, Wu C, Li Y, Wei Z, et al. Effect of individualised diet intervention on the incidence of caesarean section on pregnant women with a normal BMI. Chin JPrevent Med 2016;19(3):22–228.
651. Watson PE, McDonald BW. Activity levels in pregnant New Zealand women: relationship with socioeconomic factors, wellbeing, anthropometric measures, and birth outcome. Appl Physiol Nutr Metab. 2007;32(4):733–42.
652. Weissgerber TL, Davies GA, Roberts JM. Modification of angiogenic factors by regular and acute exercise during pregnancy. J Appl Physiol (1985). 2010;108(5):1217-23.
653. White E, Pivarnik J, Pfeiffer K. Resistance training during pregnancy and perinatal outcomes. J Phys Act Health. 2014;11(6):1141-8.
654. Wilkinson SA, McIntyre HD. Evaluation of the 'healthy start to pregnancy' early antenatal health promotion workshop: a randomized controlled trial. BMC Pregnancy and Childbirth 2012;12:131.
655. Wilkinson SA, Pligt P, Gibbons KS, McIntyre HD. Trial for Reducing Weight Retention in New Mums: a randomised controlled trial evaluating a low intensity, postpartum weight management programme. J Hum Nutr Diet 2015;28(Suppl 1):15–28.
656. Willcox J., Wilkinson S., Lappas M., Ball K., Crawford D., McCarthy E., Fjeldsoe B., Whittaker R., Maddison R., Campbell K. A mobile health intervention promoting healthy gestational weight gain for women entering pregnancy at a high body mass index: The txt4two pilot randomised controlled trial. BJOG. 2017;124:1718–1728. doi: 10.1111/1471-0528.14552
657. Wilson, S. M., et al. (2018). "Contingency Management Versus Psychotherapy for Prenatal Smoking Cessation: A Meta-Analysis of Randomized Controlled Trials." Women's Health Issues: Official Publication Of The Jacobs Institute Of Women's Health 28(6): 514-523.
658. Wiltheiss GA, Lovelady CA, West DG, Brouwer RJ, Krause KM, Østbye T. Diet quality and weight change among overweight and obese postpartum women enrolled in a behavioral intervention program. J Acad Nutr Diet 2013;113(1): 54–62
659. Woldringh C, Wijngaart Mvd, Albers-Heitner P, Lycklama a N, A A, Lagro-Janssen T. Pelvic floor muscle training is not effective in women with UI in pregnancy: a randomised controlled trial. Int Urogynecol J Pelvic Floor Dysfunct. 2007;18(4):383-90.
660. Wolfe LA, Heenan AP, Bonen A. Aerobic conditioning effects on substrate responses during graded cycling in pregnancy. Can J Physiol Pharmacol. 2003;81(7):696-703.
661. Wolff S, Legarth J, Vangsaard K, Toubro S, Astrup A. A randomized trial of effects of dietary counseling on gestational weight gain and glucose metabolism in obese pregnant women. Int J Obes (Lond) 2008;32:495–501
662. Xiao R, Liu W, Li Y, Ding J, Yin J. Effects of using DOHad nutritional monitoring on the grading of nutrition management during pregnancy. Med J Chin People's Armed Police Force 2015;26(5):493–5.

Diet and/or physical activity unique publications
663. Xu G, Wu Y, Yang L, et al. Risk factors for early miscarriage among Chinese: a hospitalbased case-control study. Fertil Steril 2014;101:1663–70<br>664. Ye L, Xu H, Ye W. Follow-up application analysis of individualized prenatal nutrition education in the community. Med Innov Chin 2016;13(2):106–10.<br>665. Yeo S, Davidge S, Ronis DL, Antonakos CL, Hayashi R, O'Leary S. A comparison of walking versus stretching exercises to reduce the incidence of preeclampsia: a randomised clinical trial. Hypertens Pregnancy 2008;27:113–30.<br>666. Yeo S, Neelon V, Weaver M, et al. Regular exercise from 12-22 weeks of pregnancy in women at risk for preeclampsia: A feasibility study. (unpublished).<br>667. Yeo S, Steele NM, Chang MC, Leclaire SM, Ronis DL, Hayashi R. Effect of exercise on blood pressure in pregnant women with a high risk of gestational hypertensive disorders. J Reprod Med 2000;45:293-8<br>668. Yeo S. Adherence to walking or stretching, and risk of preeclampsia in sedentary pregnant women. Res Nurs Health 2009;32: 379–90.<br>669. Yeo S: A randomized comparative trial of the efficacy and safety of exercise during pregnancy: design and methods. Contemp Clin Trials 2006, 27:531-540.<br>670. Young JC, Treadway JL. The effect of prior exercise on oral glucose tolerance in late gestational women. Eur J Appl Physiol Occup Physiol. 1992;64(5):430-3.<br>671. Youngwanichsetha S, Phumdoung S, Ingkathawornwong T. The effects of mindfulness eating and yoga exercise on blood sugar levels of pregnant women with gestational diabetes mellitus. Appl Nurs Res. 2014;27(4):227-30.<br>672. Zhang H, Yang Z. Effect analysis of personal medical nutrition therapy combined with exercise therapy for gestational diabetes mellitus. Jinlin Med J 2012;33(16):3369–72.<br>673. Zhang L, Zhao M, Li G. Effects of a lifestyle intervention on gestational weight gain and birth outcome in obese pregnant women. Beijing Med J 2015;37(10):1001–2.<br>674. Zhang X, Li J, Gu Y, et al. A pilot study on environmental and behavioral factors related to missed abortion. Environ Health Prev Med 2011;16:273–8.<br>675. Zhou S, Yelland L, McPhee A, Quinlivan J, Gibson R, Makrides M. Fish oil supplementation in pregnancy does not reduce the risk of gestational diabetes or preeclampsia. Am J Clin Nutr. 2012;95:1378–84.

S7 References:

1. Chamberlain C, O'Mara-Eves A, Porter J, Coleman T, Perlen SM, Thomas J, et al. Psychosocial interventions for supporting women to stop smoking in pregnancy. The Cochrane database of systematic reviews. 2017;2:Cd001055. Epub 2017/02/15. doi: 10.1002/14651858.CD001055.pub5. PubMed PMID: 28196405; PubMed Central PMCID: PMCPMC6472671.
2. Veisani Y, Jenabi E, Delpisheh A, Khazaei S. Effect of prenatal smoking cessation interventions on birth weight: meta-analysis. The journal of maternal-fetal & neonatal medicine : the official journal of the European Association of Perinatal Medicine, the Federation of Asia and Oceania Perinatal Societies, the International Society of Perinatal Obstet. 2019;32(2):332-8. Epub 2017/09/12. doi: 10.1080/14767058.2017.1378335. PubMed PMID: 28889768.
3. Agha M, Agha RA, Sandall J. Interventions to reduce and prevent obesity in pre-conceptual and pregnant women: a systematic review and meta-analysis. PloS one. 2014;9(5):e95132-e. doi: 10.1371/journal.pone.0095132. PubMed PMID: 24827704.
4. Bain E, Crane M, Tieu J, Han S, Crowther CA, Middleton P. Diet and exercise interventions for preventing gestational diabetes mellitus. The Cochrane database of systematic reviews. 2015;(4):Cd010443. Epub 2015/04/13. doi: 10.1002/14651858.CD010443.pub2. PubMed PMID: 25864059.
5. Allen R, Rogozinska E, Sivarajasingam P, Khan KS, Thangaratinam S. Effect of diet- and lifestyle-based metabolic risk-modifying interventions on preeclampsia: a meta-analysis. Acta Obstetricia et Gynecologica Scandinavica. 2014;93(10):973-85. doi: 10.1111/aogs.12467.
6. Campbell F, Johnson M, Messina J, Guillaume L, Goyder E. Behavioural interventions for weight management in pregnancy: a systematic review of quantitative and qualitative data. BMC Public Health. 2011;11:491. Epub 2011/06/24. doi: 10.1186/1471-2458-11-491. PubMed PMID: 21696589; PubMed Central PMCID: PMCPMC3154865.
7. Choi J, Fukuoka Y, Lee JH. The effects of physical activity and physical activity plus diet interventions on body weight in overweight or obese women who are pregnant or in postpartum: a systematic review and meta-analysis of randomized controlled trials. Prev Med. 2013;56(6):351-64. Epub 2013/02/26. doi: 10.1016/j.ypmed.2013.02.021. PubMed PMID: 23480971.
8. Elliott-Sale KJ, Barnett CT, Sale C. Exercise interventions for weight management during pregnancy and up to 1 year postpartum among normal weight, overweight and obese women: a systematic review and meta-analysis. Br J Sports Med. 2015;49(20):1336-42. Epub 2014/11/20. doi: 10.1136/bjsports-2014-093875. PubMed PMID: 25406335.
9. Gardner B, Wardle J, Poston L, Croker H. Changing diet and physical activity to reduce gestational weight gain: a meta-analysis. Obesity reviews : an official journal of the International Association for the Study of Obesity. 2011;12(7):e602-20. Epub 2011/04/28. doi: 10.1111/j.1467-789X.2011.00884.x. PubMed PMID: 21521451.
10. Gresham E, Bisquera A, Byles JE, Hure AJ. Effects of dietary interventions on pregnancy outcomes: a systematic review and meta-analysis. Matern Child Nutr. 2016;12(1):5-23. Epub 2014/07/23. doi: 10.1111/mcn.12142. PubMed PMID: 25048387; PubMed Central PMCID: PMCPMC6860081.
11. Han S, Middleton P, Crowther CA. Exercise for pregnant women for preventing gestational diabetes mellitus. The Cochrane database of systematic reviews. 2012;(7):Cd009021. Epub 2012/07/13. doi: 10.1002/14651858.CD009021.pub2. PubMed PMID: 22786521.
12. Hill B, Skouteris H, Fuller-Tyszkiewicz M. Interventions designed to limit gestational weight gain: a systematic review of theory and meta-analysis of intervention components. Obesity Reviews. 2013;14(6):435-50. doi: 10.1111/obr.12022.
13. Lamina S, Agbanusi E. Effect of aerobic exercise training on maternal weight gain in pregnancy: a meta-analysis of randomized controlled trials. Ethiop J Health Sci. 2013;23(1):59-64. PubMed PMID: 23559839.
14. Madhuvrata P, Govinden G, Bustani R, Song S, Farrell TA. Prevention of gestational diabetes in pregnant women with risk factors for gestational diabetes: a systematic review and meta-analysis of randomised trials. Obstet Med. 2015;8(2):68-85. Epub 2015/06/01. doi: 10.1177/1753495x15576673. PubMed PMID: 27512459; PubMed Central PMCID: PMCPMC4935009.
15. Muktabhant B, Lawrie TA, Lumbiganon P, Laopaiboon M. Diet or exercise, or both, for preventing excessive weight gain in pregnancy. The Cochrane database of systematic reviews. 2015;(6):Cd007145. Epub 2015/06/13. doi: 10.1002/14651858.CD007145.pub3. PubMed PMID: 26068707.

16. O'Brien CM, Grivell RM, Dodd JM. Systematic review of antenatal dietary and lifestyle interventions in women with a normal body mass index. Acta Obstet Gynecol Scand. 2016;95(3):259-69. Epub 2015/11/27. doi: 10.1111/aogs.12829. PubMed PMID: 26610169.
17. Oostdam N, van Poppel MN, Wouters MG, van Mechelen W. Interventions for preventing gestational diabetes mellitus: a systematic review and meta-analysis. Journal of women's health (2002). 2011;20(10):1551-63. Epub 2011/08/16. doi: 10.1089/jwh.2010.2703. PubMed PMID: 21838525.
18. Oteng-Ntim E, Varma R, Croker H, Poston L, Doyle P. Lifestyle interventions for overweight and obese pregnant women to improve pregnancy outcome: systematic review and meta-analysis. BMC Medicine. 2012;10(1):47. doi: 10.1186/1741-7015-10-47.
19. Quinlivan JA, Julania S, Lam L. Antenatal dietary interventions in obese pregnant women to restrict gestational weight gain to Institute of Medicine recommendations: a meta-analysis. Obstet Gynecol. 2011;118(6):1395-401. Epub 2011/11/23. doi: 10.1097/AOG.0b013e3182396bc6. PubMed PMID: 22105270.
20. Rogozińska E, Chamillard M, Hitman GA, Khan KS, Thangaratinam S. Nutritional manipulation for the primary prevention of gestational diabetes mellitus: a meta-analysis of randomised studies. PloS one. 2015;10(2):e0115526. Epub 2015/02/27. doi: 10.1371/journal.pone.0115526. PubMed PMID: 25719363; PubMed Central PMCID: PMCPMC4342242.
21. Russo LM, Nobles C, Ertel KA, Chasan-Taber L, Whitcomb BW. Physical activity interventions in pregnancy and risk of gestational diabetes mellitus: a systematic review and meta-analysis. Obstet Gynecol. 2015;125(3):576-82. Epub 2015/03/03. doi: 10.1097/aog.0000000000000691. PubMed PMID: 25730218.
22. Sanabria-Martínez G, García-Hermoso A, Poyatos-León R, Álvarez-Bueno C, Sánchez-López M, Martínez-Vizcaíno V. Effectiveness of physical activity interventions on preventing gestational diabetes mellitus and excessive maternal weight gain: a meta-analysis. BJOG : an international journal of obstetrics and gynaecology. 2015;122(9):1167-74. Epub 2015/06/04. doi: 10.1111/1471-0528.13429. PubMed PMID: 26036300.
23. Streuling I, Beyerlein A, Rosenfeld E, Hofmann H, Schulz T, von Kries R. Physical activity and gestational weight gain: a meta-analysis of intervention trials. BJOG : an international journal of obstetrics and gynaecology. 2011;118(3):278-84. Epub 2010/12/08. doi: 10.1111/j.1471-0528.2010.02801.x. PubMed PMID: 21134106.
24. Sui Z, Grivell RM, Dodd JM. Antenatal exercise to improve outcomes in overweight or obese women: A systematic review. Acta Obstet Gynecol Scand. 2012;91(5):538-45. Epub 2012/01/11. doi: 10.1111/j.1600-0412.2012.01357.x. PubMed PMID: 22229625.
25. Tanentsapf I, Heitmann BL, Adegboye ARA. Systematic review of clinical trials on dietary interventions to prevent excessive weight gain during pregnancy among normal weight, overweight and obese women. BMC Pregnancy and Childbirth. 2011;11(1):81. doi: 10.1186/1471-2393-11-81.
26. Thangaratinam S, Rogozińska E, Jolly K, Glinkowski S, Duda W, Borowiack E, et al. Interventions to reduce or prevent obesity in pregnant women: a systematic review. 2012;16:31. doi: 10.3310/hta16310.
27. Thangaratinam S, Rogozińska E, Jolly K, Glinkowski S, Roseboom T, Tomlinson JW, et al. Effects of interventions in pregnancy on maternal weight and obstetric outcomes: meta-analysis of randomised evidence. BMJ : British Medical Journal. 2012;344:e2088. doi: 10.1136/bmj.e2088.
28. da Silva SG, Ricardo LI, Evenson KR, Hallal PC. Leisure-Time Physical Activity in Pregnancy and Maternal-Child Health: A Systematic Review and Meta-Analysis of Randomized Controlled Trials and Cohort Studies. Sports Med. 2017;47(2):295-317. Epub 2016/06/11. doi: 10.1007/s40279-016-0565-2. PubMed PMID: 27282925.
29. Di Mascio D, Magro-Malosso ER, Saccone G, Marhefka GD, Berghella V. Exercise during pregnancy in normal-weight women and risk of preterm birth: a systematic review and meta-analysis of randomized controlled trials. American journal of obstetrics and gynecology. 2016;215(5):561-71. Epub 2016/10/30. doi: 10.1016/j.ajog.2016.06.014. PubMed PMID: 27319364.
30. International Weight Management in Pregnancy (IWiP) collaboration. Effect of diet and physical activity based interventions in pregnancy on gestational weight gain and pregnancy outcomes: meta-analysis of individual participant data from randomised trials. BMJ (Clinical research ed). 2017;358:j3119. doi: 10.1136/bmj.j3119.
31. Lau Y, Klainin-Yobas P, Htun TP, Wong SN, Tan KL, Ho-Lim ST, et al. Electronic-based lifestyle interventions in overweight or obese perinatal women: a systematic review and meta-analysis. Obesity reviews : an official journal of the International Association for the Study of Obesity. 2017;18(9):1071-87. Epub 2017/05/26. doi: 10.1111/obr.12557. PubMed PMID: 28544551.

32. Magro-Malosso ER, Saccone G, Di Tommaso M, Roman A, Berghella V. Exercise during pregnancy and risk of gestational hypertensive disorders: a systematic review and meta-analysis. Acta Obstetricia et Gynecologica Scandinavica. 2017;96(8):921-31. doi: 10.1111/aogs.13151.
33. Magro-Malosso ER, Saccone G, Di Mascio D, Di Tommaso M, Berghella V. Exercise during pregnancy and risk of preterm birth in overweight and obese women: a systematic review and meta-analysis of randomized controlled trials. Acta Obstet Gynecol Scand. 2017;96(3):263-73. Epub 2016/12/29. doi: 10.1111/aogs.13087. PubMed PMID: 28029178.
34. Sanabria-Martínez G, García-Hermoso A, Poyatos-León R, González-García A, Sánchez-López M, Martínez-Vizcaíno V. Effects of Exercise-Based Interventions on Neonatal Outcomes: A Meta-Analysis of Randomized Controlled Trials. Am J Health Promot. 2016;30(4):214-23. Epub 2016/07/13. doi: 10.1177/0890117116639569. PubMed PMID: 27404056.
35. Shepherd E, Gomersall JC, Tieu J, Han S, Crowther CA, Middleton P. Combined diet and exercise interventions for preventing gestational diabetes mellitus. The Cochrane database of systematic reviews. 2017;11:Cd010443. Epub 2017/11/13. doi: 10.1002/14651858.CD010443.pub3. PubMed PMID: 29129039; PubMed Central PMCID: PMCPMC6485974.
36. Sherifali D, Nerenberg KA, Wilson S, Semeniuk K, Ali MU, Redman LM, et al. The Effectiveness of eHealth Technologies on Weight Management in Pregnant and Postpartum Women: Systematic Review and Meta-Analysis. Journal of medical Internet research. 2017;19(10):e337. Epub 2017/10/17. doi: 10.2196/jmir.8006. PubMed PMID: 29030327; PubMed Central PMCID: PMCPMC5660296.
37. Song C, Li J, Leng J, Ma RC, Yang X. Lifestyle intervention can reduce the risk of gestational diabetes: a meta-analysis of randomized controlled trials. Obesity reviews : an official journal of the International Association for the Study of Obesity. 2016;17(10):960-9. Epub 2016/07/16. doi: 10.1111/obr.12442. PubMed PMID: 27417680.
38. Tieu J, Shepherd E, Middleton P, Crowther CA. Dietary advice interventions in pregnancy for preventing gestational diabetes mellitus. The Cochrane database of systematic reviews. 2017;1:Cd006674. Epub 2017/01/04. doi: 10.1002/14651858.CD006674.pub3. PubMed PMID: 28046205; PubMed Central PMCID: PMCPMC6464792.
39. Yeo S, Walker JS, Caughey MC, Ferraro AM, Asafu-Adjei JK. What characteristics of nutrition and physical activity interventions are key to effectively reducing weight gain in obese or overweight pregnant women? A systematic review and meta-analysis. Obesity reviews : an official journal of the International Association for the Study of Obesity. 2017;18(4):385-99. Epub 2017/02/09. doi: 10.1111/obr.12511. PubMed PMID: 28177566.
40. Zhang R, Han S, Chen GC, Li ZN, Silva-Zolezzi I, Parés GV, et al. Effects of low-glycemic-index diets in pregnancy on maternal and newborn outcomes in pregnant women: a meta-analysis of randomized controlled trials. Eur J Nutr. 2018;57(1):167-77. Epub 2016/09/11. doi: 10.1007/s00394-016-1306-x. PubMed PMID: 27612876.
41. Zheng J, Wang H, Ren M. Influence of exercise intervention on gestational diabetes mellitus: a systematic review and meta-analysis. J Endocrinol Invest. 2017;40(10):1027-33. Epub 2017/04/13. doi: 10.1007/s40618-017-0673-3. PubMed PMID: 28401529.
42. Gresham E, Byles JE, Bisquera A, Hure AJ. Effects of dietary interventions on neonatal and infant outcomes: a systematic review and meta-analysis. Am J Clin Nutr. 2014;100(5):1298-321. Epub 2014/10/22. doi: 10.3945/ajcn.113.080655. PubMed PMID: 25332328.
43. Chatzakis C, Goulis DG, Mareti E, Eleftheriades M, Zavlanos A, Dinas K, et al. Prevention of gestational diabetes mellitus in overweight or obese pregnant women: A network meta-analysis. Diabetes Res Clin Pract. 2019;158:107924. Epub 2019/11/19. doi: 10.1016/j.diabres.2019.107924. PubMed PMID: 31738997.
44. Craemer KA, Sampene E, Safdar N, Antony KM, Wautlet CK. Nutrition and Exercise Strategies to Prevent Excessive Pregnancy Weight Gain: A Meta-analysis. AJP Rep. 2019;9(1):e92-e120. Epub 2019/03/19. doi: 10.1055/s-0039-1683377. PubMed PMID: 31041118.
45. Davenport MH, Kathol AJ, Mottola MF, Skow RJ, Meah VL, Poitras VJ, et al. Prenatal exercise is not associated with fetal mortality: a systematic review and meta-analysis. Br J Sports Med. 2019;53(2):108-15. Epub 2018/10/20. doi: 10.1136/bjsports-2018-099773. PubMed PMID: 30337346.
46. Davenport MH, McCurdy AP, Mottola MF, Skow RJ, Meah VL, Poitras VJ, et al. Impact of prenatal exercise on both prenatal and postnatal anxiety and depressive symptoms: a systematic review and meta-analysis. British Journal of Sports Medicine. 2018;52(21):1376. doi: 10.1136/bjsports-2018-099697.

47. Davenport MH, Ruchat SM, Poitras VJ, Jaramillo Garcia A, Gray CE, Barrowman N, et al. Prenatal exercise for the prevention of gestational diabetes mellitus and hypertensive disorders of pregnancy: a systematic review and meta-analysis. Br J Sports Med. 2018;52(21):1367-75. Epub 2018/10/20. doi: 10.1136/bjsports-2018-099355. PubMed PMID: 30337463.
48. Davenport MH, Ruchat SM, Sobierajski F, Poitras VJ, Gray CE, Yoo C, et al. Impact of prenatal exercise on maternal harms, labour and delivery outcomes: a systematic review and meta-analysis. Br J Sports Med. 2019;53(2):99-107. Epub 2018/10/20. doi: 10.1136/bjsports-2018-099821. PubMed PMID: 30337349.
49. Davenport MH, Sobierajski F, Mottola MF, Skow RJ, Meah VL, Poitras VJ, et al. Glucose responses to acute and chronic exercise during pregnancy: a systematic review and meta-analysis. Br J Sports Med. 2018;52(21):1357-66. Epub 2018/10/20. doi: 10.1136/bjsports-2018-099829. PubMed PMID: 30337462.
50. Du MC, Ouyang YQ, Nie XF, Huang Y, Redding SR. Effects of physical exercise during pregnancy on maternal and infant outcomes in overweight and obese pregnant women: A meta-analysis. Birth. 2019;46(2):211-21. Epub 2018/09/22. doi: 10.1111/birt.12396. PubMed PMID: 30240042.
51. Guillemette L, Hay JL, Kehler DS, Hamm NC, Oldfield C, McGavock JM, et al. Exercise in Pregnancy and Children's Cardiometabolic Risk Factors: a Systematic Review and Meta-Analysis. Sports Med Open. 2018;4(1):35-. doi: 10.1186/s40798-018-0148-x. PubMed PMID: 30069801.
52. Michel S, Raab R, Drabsch T, Günther J, Stecher L, Hauner H. Do lifestyle interventions during pregnancy have the potential to reduce long-term postpartum weight retention? A systematic review and meta-analysis. Obesity Reviews. 2019;20(4):527-42. doi: 10.1111/obr.12809.
53. Ming W-K, Ding W, Zhang CJP, Zhong L, Long Y, Li Z, et al. The effect of exercise during pregnancy on gestational diabetes mellitus in normal-weight women: a systematic review and meta-analysis. BMC Pregnancy and Childbirth. 2018;18(1):440. doi: 10.1186/s12884-018-2068-7.
54. Morison PN, Bacardi-Gascon M, Lopez-Corrales M, Jimenez-Cruz A. Combined dietary-exercise intervention for gestational weight gain and birthweight: a meta-analysis. Asia Pac J Clin Nutr. 2018;27(4):860-8. Epub 2018/07/27. doi: 10.6133/apjcn.112017.02. PubMed PMID: 30045432.
55. Nasiri-Amiri F, Sepidarkish M, Shirvani MA, Habibipour P, Tabari NSM. The effect of exercise on the prevention of gestational diabetes in obese and overweight pregnant women: a systematic review and meta-analysis. Diabetology & Metabolic Syndrome. 2019;11(1):72. doi: 10.1186/s13098-019-0470-6.
56. Ruchat S-M, Mottola MF, Skow RJ, Nagpal TS, Meah VL, James M, et al. Effectiveness of exercise interventions in the prevention of excessive gestational weight gain and postpartum weight retention: a systematic review and meta-analysis. British Journal of Sports Medicine. 2018;52(21):1347. doi: 10.1136/bjsports-2018-099399.
57. Syngelaki A, Sequeira Campos M, Roberge S, Andrade W, Nicolaides KH. Diet and exercise for preeclampsia prevention in overweight and obese pregnant women: systematic review and meta-analysis. The journal of maternal-fetal & neonatal medicine : the official journal of the European Association of Perinatal Medicine, the Federation of Asia and Oceania Perinatal Societies, the International Society of Perinatal Obstet. 2019;32(20):3495-501. Epub 2018/05/25. doi: 10.1080/14767058.2018.1481037. PubMed PMID: 29792061.
58. Wang J, Wen D, Liu X, Liu Y. Impact of exercise on maternal gestational weight gain: An updated meta-analysis of randomized controlled trials. Medicine (Baltimore). 2019;98(27):e16199-e. doi: 10.1097/MD.0000000000016199. PubMed PMID: 31277127.
59. Poyatos-León R, García-Hermoso A, Sanabria-Martínez G, Álvarez-Bueno C, Sánchez-López M, Martínez-Vizcaíno V. Effects of exercise during pregnancy on mode of delivery: a meta-analysis. Acta Obstet Gynecol Scand. 2015;94(10):1039-47. Epub 2015/05/13. doi: 10.1111/aogs.12675. PubMed PMID: 25965378.
60. Walker R, Bennett C, Blumfield M, Gwini S, Ma J, Wang F, et al. Attenuating Pregnancy Weight Gain-What Works and Why: A Systematic Review and Meta-Analysis. Nutrients. 2018;10(7):944. doi: 10.3390/nu10070944. PubMed PMID: 30037126.
61. Vincze L, Rollo M, Hutchesson M, Hauck Y, MacDonald-Wicks L, Wood L, et al. Interventions including a nutrition component aimed at managing gestational weight gain or postpartum weight retention: a systematic review and meta-analysis. JBI Evidence Synthesis. 2019;17(3):297-364. doi: 10.11124/jbisrir-2017-003593. PubMed PMID: 01938924-201903000-00007.
62. Wiebe HW, Boulé NG, Chari R, Davenport MH. The Effect of Supervised Prenatal Exercise on Fetal Growth: A Meta-analysis. Obstetrics & Gynecology. 2015;125(5):1185-94. doi: 10.1097/aog.0000000000000801. PubMed PMID: 00006250-201505000-00028.

63. Yin YN, Li XL, Tao TJ, Luo BR, Liao SJ. Physical activity during pregnancy and the risk of gestational diabetes mellitus: a systematic review and meta-analysis of randomised controlled trials. Br J Sports Med. 2014;48(4):290-5. Epub 2013/09/17. doi: 10.1136/bjsports-2013-092596. PubMed PMID: 24037671.
64. Shieh C, Cullen DL, Pike C, Pressler SJ. Intervention strategies for preventing excessive gestational weight gain: systematic review and meta-analysis. Obesity Reviews. 2018;19(8):1093-109. doi: https://doi.org/10.1111/obr.12691.
65. Bennett CJ, Walker RE, Blumfield ML, Gwini S-M, Ma J, Wang F, et al. Interventions designed to reduce excessive gestational weight gain can reduce the incidence of gestational diabetes mellitus: A systematic review and meta-analysis of randomised controlled trials. Diabetes research and clinical practice. 2018;141:69-79. doi: 10.1016/j.diabres.2018.04.010. PubMed PMID: 29698713.

S8 Tables: Meta-analysis for maternal weight-related outcomes reported by the included systematic reviews

Table S8a: Meta-analysis for total gestational weight gain (GWG) reported by the included systematic reviews

Systematic review author, year	Included population or intervention subgroup	N studies	Sample size pooled data	Meta-analysis: total GWG	Statistical significance
			Diet and/or Physical Activity		
**Agha *et al.* 2014 [1]**	**Any BMI**	**14**	**1,771**	**MD -1.66kg (95% CI -3.12, -0.21)**	**Significantly reduced p=0.03**
Bain *et al.* 2015 [2]	Any BMI	8	2,707	MD -0.76kg (95% CI -1.55, 0.03)	Not significant p=0.06
**Gardner *et al.* 2011 [3]**	**Any BMI**	**12**	**1,655**	**WMD -1.19kg (95% CI -1.74, -0.65)**	**Significantly reduced p<0.0001**
**Hill *et al.* 2013 [4]**	**Any BMI**	**22**	**3,710**	**WMD -1.54kg (95% CI -1.86, -1.21)**	**Significantly reduced p<0.001**
**Shepherd *et al.* 2017 [5]**	**Any BMI**	**16**	**5,052**	**MD -0.89kg (95% CI -1.39, -0.40)**	**Significantly reduced**
Sherifali *et al.* 2017 [6]	Any BMI	6	363	MD -1.62kg (95% CI -3.57, 0.33)	Not significant p=0.10
**Craemer *et al.* 2019 [7]**	**Any BMI**	**19**	**5,103**	**SMD -0.37kg (95% CI -0.49, -0.24)**	**Significantly reduced p<0.0001**
**Morison *et al.* 2018 [8]**	**Any BMI**	**10**	**5,611**	**SMD -0.21kg (95% CI -0.34, -0.08)**	**Significantly reduced p=0.002**
**Ruchat *et al.* 2018 [9]**	**Any BMI**	**49**	**11,607**	**MD -0.9kg (95% CI -1.16, -0.65)**	**Significantly reduced**
**Walker *et al.* 2018 [10]**	**Any BMI**	**24**	**7,379**	**WMD -0.84 (95% CI -1.29, -0.39)**	**Significantly reduced p<0.00**
**Vincze *et al.* 2019 [11]**	**Any BMI**	**23**	**5230**	**MD -1.25kg (95% CI -2.1, -0.4)**	**Significantly reduced p=0.004**
**Tanentsapf *et al.* 2011 [12]**	**Recommended BMI, overweight, obese**	**10**	**1,434**	**WMD -1.92kg (95% CI -3.65, -0.19)**	**Significantly reduced p=0.03**
**Thangaratinam *et al.* 2012 [13]**	**Recommended BMI, overweight, obese**	**30**	**4,503**	**MD -0.97 kg (95% CI –1.60, –0.34)**	**Significantly reduced p=0.003**
**Thangaratinam *et al.* 2012b [14]**	**Recommended BMI, overweight, obese**	**34**	**5,481**	**MD -1.42kg (95% CI -1.89, -0.95)**	**Significantly reduced p<0.001**
**IWiP 2017 [15]**	**Recommended BMI, overweight, obese**	**33**	**9,320**	**MD –0.70 kg (95% CI –0.92, –0.48) (IPD MA)**	**Significantly reduced**
**IWiP 2017 [15]**	**Recommended BMI, overweight, obese**	**81**	**17,530**	**MD –1.10 kg (95% CI –1.46, –0.74) (IPD + non-IPD)**	**Significantly reduced**
Campbell *et al*. 2011 [16]	Recommended BMI, overweight, obese	5	390	SMD -0.28kg (95% CI -0.64, 0.09)	Not significant p=0.14
Bain *et al.* 2015 [2]	Recommended BMI	2	241	MD -0.92kg (95% CI -2.12, 0.29)	Not significant
**O'Brien *et al.* 2015 [17]**	**Recommended BMI**	**4**	**446**	**MD -1.25kg (95% CI -2.39, -0.11)**	**Significantly reduced p=0.03**
Campbell *et al*. 2011 [16]	Recommended BMI	5	390	SMD -0.56kg (95% CI -2.84, 1.72)	Not significant
**IWiP 2017 [15]**	**Recommended BMI**	**21**	**3,376**	**MD –0.77kg (95% CI –1.15, –0.39)**	**Significantly reduced**
**IWiP 2017 [15]**	**Overweight**	**28**	**2,574**	**MD –0.75kg (95% CI –1.22, –0.27)**	**Significantly reduced**
Bain *et al.* 2015 [2]	Overweight or obese	3	1,980	MD 0.28kg (95% CI -1.13, 1.69)	Not significant
**Choi *et al.* 2013 [18]**	**Overweight or obese**	**7**	**721**	**MD -0.91kg (95% CI -1.76, -0.06)**	**Significantly reduced p=0.035**
**Gardner *et al.* 2011 [3]**	**Overweight or obese**	**6**	**565**	**WMD -2.26kg (95% CI -3.28, -1.24)**	**Significantly reduced P<0.0001**
**Lau *et al.* 2017 [19]**	**Overweight or obese**	**7**	**1,652**	**MD -0.63kg (95% CI -1.07, -0.20)**	**Significantly reduced p=0.004**
**Oteng-Ntim *et al.* 2012 [20]**	**Overweight or obese**	**10**	**1,228**	**MD -2.21kg (95% CI -2.86, -1.57)**	**Significantly reduced p<0.00001**
**Yeo *et al.* 2017 [21]**	**Overweight or obese**	**32**	**5,857**	**WMD -1.71kg (95% CI -2.55, -0.86)**	**Significantly reduced p<0.001**
**Syngelaki *et al.* 2019 [22]**	**Overweight or obese**	**17**	**5,589**	**MD -1.47kg (95% CI -1.97, -0.97)**	**Significantly reduced p<0.00001**
**Shieh *et al.* 2018 [23]**	**Overweight or obese**	**21**	**6,920**	**MD -1.81 kg (95% CI -3.47, -0.16)**	**Significantly reduced p=0.03**

Systematic review author, year	Included population or intervention subgroup	N studies	Sample size pooled data	Meta-analysis: total GWG	Statistical significance
**Agha *et al.* 2014 [1]**	**Obese**	**4**	**631**	**MD -4.65kg (95% CI -8.14, -0.56)**	**Significantly reduced**
**IWiP 2017 [15]**	**Obese**	**31**	**3,335**	**MD −0.85kg (95% CI −1.41 to −0.29)**	**Significantly reduced**
**Diet only**					
Zhang *et al.* 2018 [24]	Any BMI - Low GI	9	1,796	WMD −0.69kg (95% CI −1.74, 0.36)	Not significant
**Tieu *et al.* 2017 [25]**	**Any BMI**	**5**	**1,336**	**MD -4.70kg (95% CI -8.07, -1.34)**	**Significantly reduced p=0.0062**
**Craemer *et al.* 2019 [7]**	**Any BMI**	**6**	**1,874**	**SMD -1.81kg (95% CI -2.61, -1.02)**	**Significantly reduced p<0.0001**
**Walker *et al.* 2018 [10]**	**Any BMI**	**9**	**2,049**	**WMD -3.27kg (95% CI -4.96, -1.58)**	**Significantly reduced p<0.001**
**Thangaratinam *et al.* 2012 [13]**	**Recommended BMI, overweight, obese**	**9**	**2,436**	**MD -3.36 kg (95% CI -4.73, -1.99)**	**Significantly reduced p<0.00001**
**Thangaratinam *et al.* 2012b [14]**	**Recommended BMI, overweight, obese**	**10**	**2,560**	**MD −3.84kg (95% CI -5.22, -2.45)**	**Significantly reduced p<0.001**
IWiP 2017 [15]	Recommended BMI, overweight, obese	4	1,168	MD −0.72kg (95% CI −1.48, 0.04) (IPD MA)	Not significant
**IWiP 2017 [15]**	**Recommended BMI, overweight, obese**	**12**	**2,017**	**MD -2.84 kg (95%CI −4.77, −0.91) (IPD + non-IPD)**	**Significantly reduced**
**Syngelaki *et al.* 2019 [22]**	**Overweight or obese**	**13**	**4,927**	**MD -1.56kg (95% CI -2.94, -0.99)**	**Significantly reduced p<0.00001**
**Shieh *et al.* 2018 [23]**	**Overweight or obese**	**4**	**719**	**MD -5.77kg (95% CI -9.34, -2.21)**	**Significantly reduced p=0.002**
**Physical activity only**					
**Lamina & Agbanusi 2013 [26]**	**Any BMI**	**11**	**1,177**	**MD -3.1kg (95% CI -3.91, -2.31)**	**Significantly reduced p<0.001**
**Streuling *et al.* 2011 [27]**	**Any BMI**	**12**	**906**	**SMD -0.61kg (95% CI -1.17, -0.06)**	**Significantly reduced p=0.03**
**da Silva *et al.* 2017 [28]**	**Any BMI**	**18**	**3,203**	**MD -1.11kg (95% CI -1.53, -0.69)**	**Significantly reduced**
**Craemer *et al.* 2019 [7]**	**Any BMI**	**7**	**1,661**	**SMD -0.37kg (95% CI -0.66, -0.08)**	**Significantly reduced p<0.0001**
**Ruchat *et al.* 2018 [9]**	**Any BMI**	**28**	**5,819**	**MD -0.9kg (95% CI -1.23, -0.57)**	**Significantly reduced**
**Wang *et al.* 2019 [29]**	**Any BMI**	**23**	**4,462**	**WMD -1.02kg (95% CI -1.35, -0.70)**	**Significantly reduced p<0.004**
**Walker *et al.* 2018 [10]**	**Any BMI**	**24**	**4,901**	**WMD -1.02kg (95% CI -1.56, -0.49)**	**Significantly reduced p<0.001**
**Wiebe *et al.* 2015 [30]**	**Any BMI**	**20**	**3,527**	**WMD -1.06kg (95% CI -1.51, -0.62)**	**Significantly reduced p<0.00001**
**Elliot-Sale *et al.* 2015 [31]**	**Recommended BMI, overweight, obese**	**3**	**214**	**MD -2.22kg (95% CI -3.13, -1.30)**	**Significantly reduced p<0.00001**
Thangaratinam *et al.* 2012 [13]	Recommended BMI, overweight, obese	15	1,165	MD -0.57 kg (-1.60, 0.65)	Not significant p=0.89
**Thangaratinam *et al.* 2012b [14]**	**Recommended BMI, overweight, obese**	**14**	**1,057**	**MD −0.72kg (95% CI -1.20, -0.25)**	**Significantly reduced p=0.003**
**IWiP 2017 [15]**	**Recommended BMI, overweight, obese**	**15**	**2,915**	**MD -0.73 kg (95% CI −1.11, −0.34) (IPD MA)**	**Significantly reduced**
**IWiP 2017 [15]**	**Recommended BMI, overweight, obese**	**37**	**7,355**	**MD −0.72 kg (95% CI −1.04, −0.41) (IPD + non-IPD)**	**Significantly reduced**
**Ming *et al.* 2018 [32]**	**Recommended BMI**	**5**	**1,688**	**MD −1.61kg (95% CI −1.99, −1.22)**	**Significantly reduced p<0.01**
Sui *et al.* 2012 [33]	Overweight	3	106	SMD -0.12kg (95%CI -0.51, 0.26)	Not significant p=0.52
**Sui *et al.* 2012 [33]**	**Overweight or obese**	**5**	**216**	**SMD -0.36kg (95%CI -0.64, -0.09)**	**Significantly reduced p=0.008**
**Chatzakis *et al.* 2019 [34]**	**Overweight or obese**	**11**	**Not reported**	**MD -0.96 kg (95% CI -1.66, -0.27)**	**Significantly reduced**
**Du *et al.* 2019 [35]**	**Overweight or obese**	**12**	**1,172**	**SMD −1.14kg (95% CI −1.67, −0.62)**	**Significantly reduced p<0.001**
Ruchat *et al.* 2018 [9]	Overweight or obese	6	644	MD -0.58kg (95% CI -1.30, 0.13)	Not significant p=0.11
**Syngelaki *et al.* 2019 [22]**	**Overweight or obese**	**4**	**662**	**MD -1.15kg (95% CI -2.25, -0.05)**	**Significantly reduced p=0.04**
Shieh *et al.* 2018 [23]	Overweight or obese	6	348	MD -0.28kg (95% CI -1.50, 0.94)	Not significant p=0.65

Systematic review author, year	Included population or intervention subgroup	N studies	Sample size pooled data	Meta-analysis: total GWG	Statistical significance
Wiebe *et al.* 2015 [30]	Overweight or obese	3	456	WMD -0.3kg (95% CI -1.13, 0.52)	Not significant p=0.47
**Sui *et al.* 2012 [33]**	**Obese**	**3**	**38**	**SMD -0.91kg (95%CI -1.66, -0.66)**	**Significantly reduced p=0.02**
**Sanabria-Martínez *et al.* 2015 [36]**	**Not reported**	**13**	**2,873**	**WMD -1.14kg (95% CI -1.50, -0.78)**	**Significantly reduced p<0.001**

Footnote: BMI – body mass index, N – number, GWG - gestational weight gain, MD - mean difference, WMD – weighted mean difference, SMD – standardised mean difference, CI – confidence interval, IPD – individual participant data, MA – meta-analysis, kg - kilograms. Bold data highlights statistically significant results.

Table S8b: Meta-analysis for weekly gestational weight gain (GWG) reported by the included systematic reviews

Systematic review author, year	Included population or intervention subgroup	N studies	Sample size pooled data	Meta-analysis: Weekly GWG	Statistical significance
**Diet and/or Physical Activity**					
Bain *et al.* 2015 [2]	Any BMI	2	1,971	MD -0.04 kg/week (95% CI -0.11, 0.04)	Not significant
**Shepherd *et al.* 2017 [5]**	**Any BMI**	**4**	**2,772**	**MD -0.03kg/week (95% CI -0.06, -0.00)**	**Significantly reduced**
**Ruchat *et al.* 2018 [9]**	**Any BMI**	**4**	**1,046**	**MD -0.06kg/week (95% CI -0.08, -0.03)**	**Significantly reduced**
**Tanentsapf *et al.* 2011 [12]**	**Recommended BMI, overweight, obese**	**2**	**253**	**WMD -0.26kg/week (95% CI -0.42, -0.09)**	**Significantly reduced**

Footnote: BMI – body mass index, N – number, GWG - gestational weight gain, MD - mean difference, WMD – weighted mean difference, CI – confidence interval, kg - kilograms. Bold data highlights statistically significant results.

Table S8c: Meta-analysis for excess gestational weight gain (GWG) above the Institute of Medicine (IOM) recommendations reported by the included systematic reviews

Systematic review author, year	Included population or intervention subgroup	N studies	Sample size pooled data	Meta-analysis: Excess GWG (above IOM recommendations)	Statistical significance
**Diet and/or Physical Activity**					
Bain *et al.* 2015 [2]	Any BMI	2	1,817	RR 0.87 (95% CI 0.57, 1.32)	Not significant
**Muktabhant *et al.* 2015 [37]**	**Any BMI**	**24**	**7,096**	**RR 0.80 (95% CI 0.73, 0.87)**	**Significantly reduced**
**Shepherd *et al.* 2017 [5]**	**Any BMI**	**11**	**4,556**	**RR 0.87 (95% CI 0.79, 0.96)**	**Significantly reduced**
Sherifali *et al.* 2017 [6]	Any BMI	2	99	OR 0.76 (95% CI 0.13, 4.59)	Not significant p=0.76
**Ruchat *et al.* 2018 [9]**	**Any BMI**	**35**	**3,858**	**OR 0.68 (95% CI 0.59, 0.78)**	**Significantly reduced p<0.00001**
Tanentsapf *et al.* 2011 [12]	Recommended BMI, overweight, obese	4	629	RR 0.90 (95% CI 0.77, 1.05)	Not significant
Thangaratinam *et al.* 2012 [13]	Recommended BMI, overweight, obese	3	420	RR 0.77 (95% CI 0.42, 1.42)	Not significant p=0.41
Thangaratinam *et al.* 2012b [14]	Recommended BMI, overweight, obese	5	873	RR 0.85 (95% CI 0.66, 1.11)	Not significant p=0.21
**O'Brien *et al.* 2015 [17]**	**Recommended BMI**	**5**	**714**	**RR 0.72 (95% CI 0.60, 0.86)**	**Significantly reduced p=0.0003**
**Diet only**					
**Muktabhant *et al.* 2015 [37]**	**Any BMI (Low-GI)**	**2**	**835**	**RR 0.77 (95% CI 0.66, 0.91)**	**Significantly reduced**
**Physical activity only**					
**Ruchat *et al.* 2018 [9]**	**Any BMI**	**15**	**1,305**	**OR 0.68 (95% CI 0.57, 0.80)**	**Significantly reduced p<0.00001**
Ruchat *et al.* 2018 [9]	Overweight or obese	8	928	OR 0.77 (95% CI 0.56, 1.06)	Not significant p=0.11

Footnote: IOM – institute of medicine, BMI – body mass index, GWG - gestational weight gain, N – number, RR – risk ratio, OR – odds ratio, CI – confidence interval, low-GI – low glycaemic index diet. Bold data highlights statistically significant results. IOM 2009 GWG recommendations: underweight 12.5-18 kg; recommended BMI 11.5-16kg; overweight 7-11.5kg; obese 5-9kg. Bold data highlights statistically significant results.

Table S8d: Meta-analysis for inadequate gestational weight gain (GWG) below Institute of Medicine (IOM) recommendations reported by the included systematic reviews

Systematic review author, year	Included population or intervention subgroup	N studies	Sample size pooled data	Meta-analysis: Inadequate GWG (below IOM recommendations)	Statistical significance
**Diet and/or Physical Activity**					
Bain *et al.* 2015 [2]	Any BMI	2	1,817	RR 1.00 (95% CI 0.86, 1.18)	Not significant
**Muktabhant *et al.* 2015 [37]**	**Any BMI**	**11**	**4,422**	**RR 1.14 (95% CI 1.02, 1.27)**	**Significantly increased**
Shepherd *et al.* 2017 [5]	Any BMI	7	3,499	RR 1.10 (95% CI 0.98, 1.24)	Not significant
**Ruchat *et al.* 2018 [9]**	**Any BMI**	**13**	**1,111**	**OR 1.22 (95% CI 1.03, 1.45)**	**Significantly increased p=0.02**
O'Brien *et al.* 2015 [17]	Recommended BMI	2	243	RR 1.33 (95% CI 0.74, 2.37)	Not significant
**Physical activity only**					
**Ruchat *et al.* 2018 [9]**	**Any BMI**	**5**	**392**	**OR 1.32 (95% CI 1.04, 1.67)**	**Significantly increased p=0.02**
Ruchat *et al.* 2018 [9]	Overweight or obese	3	504	OR 1.09 (95% CI 0.63, 1.88)	Not significant p=0.77

Footnote: IOM – institute of medicine, BMI – body mass index, GWG - gestational weight gain, N – number, RR – risk ratio, OR – odds ratio, CI – confidence interval. Bold data highlights statistically significant results. IOM 2009 GWG recommendations: underweight 12.5-18 kg; recommended BMI 11.5-16kg; overweight 7-11.5kg; obese 5-9kg. Bold data highlights statistically significant results.

Table S8e: Meta-analysis for adequate gestational weight gain (GWG) within Institute of Medicine (IOM) recommendations reported by the included systematic reviews

Systematic review author, year	Included population or intervention subgroup	N studies	Sample size pooled data	Meta-analysis result: Adequate GWG (within IOM recommendations)	Statistical significance
**Diet and/or Physical Activity**					
Shepherd *et al.* 2017 [5]	Any BMI	9	3,730	RR 1.02 (95% CI 0.93, 1.11)	Not significant
**Ruchat *et al.* 2018 [9]**	**Any BMI**	**16**	**5,497**	**OR 1.39 (95% CI 1.16 to 1.67)**	**Significantly increased**
O'Brien *et al.* 2015 [17]	Recommended BMI	2	243	RR 1.32 (95% CI 0.96, 1.83)	Not significant
**Physical activity only**					
**Ruchat *et al.* 2018 [9]**	**Any BMI**	**8**	**2,038**	**OR 1.54 (95% CI 1.17 to 2.01)**	**Significantly increased**
**Ruchat *et al.* 2018 [9]**	**Overweight or obese**	**4**	**610**	**OR 1.69 (95% CI 1.19, 2.42)**	**Significantly increased p=0.004**

Footnote: IOM – institute of medicine, BMI – body mass index, GWG - gestational weight gain, N – number, RR – risk ratio, OR – odds ratio, CI – confidence interval. Bold data highlights statistically significant results. IOM 2009 GWG recommendations: underweight 12.5-18 kg; recommended BMI 11.5-16kg; overweight 7-11.5kg; obese 5-9kg. Bold data highlights statistically significant results.

Table S8f: Meta-analysis for postnatal weight retention reported by the included systematic reviews

Systematic review author, year	Included population or intervention subgroup	N studies	Sample size pooled data	Meta-analysis: postnatal weight retention	Statistical significance
**Diet and/or Physical Activity**					
Agha *et al.* 2014 [1]	Any BMI	6	839	MD -0.99kg (95% CI -2.25, 0.26)	Not significant
Bain *et al.* 2015 [2]	Any BMI	3	450	MD -0.72kg (95% CI -1.96, 0.51)	Not significant
Muktabhant *et al.* 2015 [37]	Any BMI	7	818	MD -1.12kg (95% CI -2.49, 0.25)	Not significant
**Muktabhant *et al.* 2015 [37]**	**Any BMI**	**5**	**902**	**RR 0.78 (95% CI 0.63, 0.97)**	**Significantly reduced**
**Ruchat *et al.* 2018 [9]**	**Any BMI**	**11**	**4,196**	**MD –0.85kg (95% CI-1.46, -0.25)**	**Significantly reduced p=0.006**
**Shepherd *et al.* 2017 [5]**	**Any BMI**	**6**	**1,673**	**MD -0.94 kg (95% CI -1.52, -0.37)**	**Significantly reduced**
**Michel S *et al.* 2019 [38]**	**Any BMI**	**14**	**3,661**	**WMD -0.73kg (95% CI -1.32, -0.14)**	**Significantly reduced p=0.015**
**Tanentsapf *et al.* 2011 [12]**	**Recommended BMI, overweight, obese**	**2**	**306**	**WMD 0.58kg (95% CI 0.13, 1.03) (6 weeks)**	**Significantly increased**
**Michel S *et al.* 2019 [38]**	**Any BMI**	**8**	**2,446**	**WMD -1.32kg (95% CI -2.11, -0.53) (4-6 months)**	**Significantly reduced p=0.001**
**Tanentsapf *et al.* 2011 [12]**	**Recommended BMI, overweight, obese**	**3**	**443**	**WMD -1.90kg (95% CI -1.69, -1.12) (6 months)**	**Significantly reduced**
**Michel S *et al.* 2019 [38]**	**Any BMI**	**9**	**2,237**	**WMD -0.68kg (95% CI -1.28, -0.09) (12 months)**	**Significantly reduced p=0.023**
Michel S *et al.* 2019 [38]	Any BMI	2	448	WMD -0.47kg (95% CI -1.72, 0.78) (>12 months)	Not significant p=0.462
**Bain *et al.* 2015 [2]**	**Recommended BMI**	**3**	**263**	**MD -1.31kg (95% CI -2.40, -0.23)**	**Significantly reduced**
Bain *et al.* 2015 [2]	Overweight or obese	2	187	MD 1.05kg (95% CI -2.73, 4.83)	Not significant
Michel S *et al.* 2019 [38]	Overweight or obese	5	1,206	WMD -0.38kg (95% CI -1.12, 0.35)	Not significant
**Physical activity only**					
**Ruchat *et al.* 2018 [9]**	**Any BMI**	**3**	**420**	**MD -0.92kg (95% CI -1.84, 0.00)**	**Significantly reduced p=0.05**

Footnote: BMI – body mass index, N – number, MD - mean difference, WMD – weighted mean difference, CI – confidence interval, kg - kilograms. Bold data highlights statistically significant results.

Table S8g: Meta-analysis for additional weight-related outcomes reported by single systematic reviews

Systematic review author, year	Included population or intervention subgroup	Outcome reported	N studies	Sample size pooled data	Meta-analysis	Statistical significance
**Diet and/or Physical Activity**						
Thangaratinam *et al.* 2012 [13]	Recommended BMI, overweight, obese	BMI at delivery	3	1,085	MD -0.23 kg/m² (95% CI -1.40, 0.94)	Not significant p=0.70
Agha *et al.* 2014 [1]	Any BMI	Postnatal weight loss	5	669	MD 0.21kg (95% CI -0.58, 1.01)	Not significant
**Shepherd *et al.* 2017 [5]**	**Any BMI**	**Postnatal return to pre-pregnancy BMI**	**3**	**960**	**RR 1.25 (95% CI 1.08, 1.45)**	**Significantly increased**
Shepherd *et al.* 2017 [5]	Any BMI	Postnatal BMI	2	902	MD -0.15 (95% CI -0.85, 0.55)	Not significant

Footnote: BMI – body mass index, N – number, RR – risk ratio, MD - mean difference, CI – confidence interval, kg - kilograms. Bold data highlights statistically significant results.

S8 References

1. Agha M, Agha RA, Sandall J. Interventions to reduce and prevent obesity in pre-conceptual and pregnant women: a systematic review and meta-analysis. PloS one. 2014;9(5):e95132-e. doi: 10.1371/journal.pone.0095132. PubMed PMID: 24827704.
2. Bain E, Crane M, Tieu J, Han S, Crowther CA, Middleton P. Diet and exercise interventions for preventing gestational diabetes mellitus. The Cochrane database of systematic reviews. 2015;(4):Cd010443. Epub 2015/04/13. doi: 10.1002/14651858.CD010443.pub2. PubMed PMID: 25864059.
3. Gardner B, Wardle J, Poston L, Croker H. Changing diet and physical activity to reduce gestational weight gain: a meta-analysis. Obesity reviews : an official journal of the International Association for the Study of Obesity. 2011;12(7):e602-20. Epub 2011/04/28. doi: 10.1111/j.1467-789X.2011.00884.x. PubMed PMID: 21521451.
4. Hill B, Skouteris H, Fuller-Tyszkiewicz M. Interventions designed to limit gestational weight gain: a systematic review of theory and meta-analysis of intervention components. Obesity Reviews. 2013;14(6):435-50. doi: 10.1111/obr.12022.
5. Shepherd E, Gomersall JC, Tieu J, Han S, Crowther CA, Middleton P. Combined diet and exercise interventions for preventing gestational diabetes mellitus. The Cochrane database of systematic reviews. 2017;11:Cd010443. Epub 2017/11/13. doi: 10.1002/14651858.CD010443.pub3. PubMed PMID: 29129039; PubMed Central PMCID: PMCPMC6485974.
6. Sherifali D, Nerenberg KA, Wilson S, Semeniuk K, Ali MU, Redman LM, et al. The Effectiveness of eHealth Technologies on Weight Management in Pregnant and Postpartum Women: Systematic Review and Meta-Analysis. Journal of medical Internet research. 2017;19(10):e337. Epub 2017/10/17. doi: 10.2196/jmir.8006. PubMed PMID: 29030327; PubMed Central PMCID: PMCPMC5660296.
7. Craemer KA, Sampene E, Safdar N, Antony KM, Wautlet CK. Nutrition and Exercise Strategies to Prevent Excessive Pregnancy Weight Gain: A Meta-analysis. AJP Rep. 2019;9(1):e92-e120. Epub 2019/03/19. doi: 10.1055/s-0039-1683377. PubMed PMID: 31041118.
8. Morison PN, Bacardi-Gascon M, Lopez-Corrales M, Jimenez-Cruz A. Combined dietary-exercise intervention for gestational weight gain and birthweight: a meta-analysis. Asia Pac J Clin Nutr. 2018;27(4):860-8. Epub 2018/07/27. doi: 10.6133/apjcn.112017.02. PubMed PMID: 30045432.
9. Ruchat S-M, Mottola MF, Skow RJ, Nagpal TS, Meah VL, James M, et al. Effectiveness of exercise interventions in the prevention of excessive gestational weight gain and postpartum weight retention: a systematic review and meta-analysis. British Journal of Sports Medicine. 2018;52(21):1347. doi: 10.1136/bjsports-2018-099399.

10. Walker R, Bennett C, Blumfield M, Gwini S, Ma J, Wang F, et al. Attenuating Pregnancy Weight Gain-What Works and Why: A Systematic Review and Meta-Analysis. Nutrients. 2018;10(7):944. doi: 10.3390/nu10070944. PubMed PMID: 30037126.
11. Vincze L, Rollo M, Hutchesson M, Hauck Y, MacDonald-Wicks L, Wood L, et al. Interventions including a nutrition component aimed at managing gestational weight gain or postpartum weight retention: a systematic review and meta-analysis. JBI Evidence Synthesis. 2019;17(3):297-364. doi: 10.11124/jbisrir-2017-003593. PubMed PMID: 01938924-201903000-00007.
12. Tanentsapf I, Heitmann BL, Adegboye ARA. Systematic review of clinical trials on dietary interventions to prevent excessive weight gain during pregnancy among normal weight, overweight and obese women. BMC Pregnancy and Childbirth. 2011;11(1):81. doi: 10.1186/1471-2393-11-81.
13. Thangaratinam S, Rogozińska E, Jolly K, Glinkowski S, Duda W, Borowiack E, et al. Interventions to reduce or prevent obesity in pregnant women: a systematic review. 2012;16:31. doi: 10.3310/hta16310.
14. Thangaratinam S, Rogozińska E, Jolly K, Glinkowski S, Roseboom T, Tomlinson JW, et al. Effects of interventions in pregnancy on maternal weight and obstetric outcomes: meta-analysis of randomised evidence. BMJ : British Medical Journal. 2012;344:e2088. doi: 10.1136/bmj.e2088.
15. International Weight Management in Pregnancy (IWiP) collaboration. Effect of diet and physical activity based interventions in pregnancy on gestational weight gain and pregnancy outcomes: meta-analysis of individual participant data from randomised trials. BMJ (Clinical research ed). 2017;358:j3119. doi: 10.1136/bmj.j3119.
16. Campbell F, Johnson M, Messina J, Guillaume L, Goyder E. Behavioural interventions for weight management in pregnancy: a systematic review of quantitative and qualitative data. BMC Public Health. 2011;11:491. Epub 2011/06/24. doi: 10.1186/1471-2458-11-491. PubMed PMID: 21696589; PubMed Central PMCID: PMCPMC3154865.
17. O'Brien CM, Grivell RM, Dodd JM. Systematic review of antenatal dietary and lifestyle interventions in women with a normal body mass index. Acta Obstet Gynecol Scand. 2016;95(3):259-69. Epub 2015/11/27. doi: 10.1111/aogs.12829. PubMed PMID: 26610169.
18. Choi J, Fukuoka Y, Lee JH. The effects of physical activity and physical activity plus diet interventions on body weight in overweight or obese women who are pregnant or in postpartum: a systematic review and meta-analysis of randomized controlled trials. Prev Med. 2013;56(6):351-64. Epub 2013/02/26. doi: 10.1016/j.ypmed.2013.02.021. PubMed PMID: 23480971.
19. Lau Y, Klainin-Yobas P, Htun TP, Wong SN, Tan KL, Ho-Lim ST, et al. Electronic-based lifestyle interventions in overweight or obese perinatal women: a systematic review and meta-analysis. Obesity reviews : an official journal of the International Association for the Study of Obesity. 2017;18(9):1071-87. Epub 2017/05/26. doi: 10.1111/obr.12557. PubMed PMID: 28544551.
20. Oteng-Ntim E, Varma R, Croker H, Poston L, Doyle P. Lifestyle interventions for overweight and obese pregnant women to improve pregnancy outcome: systematic review and meta-analysis. BMC Medicine. 2012;10(1):47. doi: 10.1186/1741-7015-10-47.
21. Yeo S, Walker JS, Caughey MC, Ferraro AM, Asafu-Adjei JK. What characteristics of nutrition and physical activity interventions are key to effectively reducing weight gain in obese or overweight pregnant women? A systematic review and meta-analysis. Obesity reviews : an official journal of the International Association for the Study of Obesity. 2017;18(4):385-99. Epub 2017/02/09. doi: 10.1111/obr.12511. PubMed PMID: 28177566.
22. Syngelaki A, Sequeira Campos M, Roberge S, Andrade W, Nicolaides KH. Diet and exercise for preeclampsia prevention in overweight and obese pregnant women: systematic review and meta-analysis. The journal of maternal-fetal & neonatal medicine : the official journal of the European Association of Perinatal Medicine, the Federation of Asia and Oceania Perinatal Societies, the International Society of Perinatal Obstet. 2019;32(20):3495-501. Epub 2018/05/25. doi: 10.1080/14767058.2018.1481037. PubMed PMID: 29792061.

23. Shieh C, Cullen DL, Pike C, Pressler SJ. Intervention strategies for preventing excessive gestational weight gain: systematic review and meta-analysis. Obesity Reviews. 2018;19(8):1093-109. doi: https://doi.org/10.1111/obr.12691.
24. Zhang R, Han S, Chen GC, Li ZN, Silva-Zolezzi I, Parés GV, et al. Effects of low-glycemic-index diets in pregnancy on maternal and newborn outcomes in pregnant women: a meta-analysis of randomized controlled trials. Eur J Nutr. 2018;57(1):167-77. Epub 2016/09/11. doi: 10.1007/s00394-016-1306-x. PubMed PMID: 27612876.
25. Tieu J, Shepherd E, Middleton P, Crowther CA. Dietary advice interventions in pregnancy for preventing gestational diabetes mellitus. The Cochrane database of systematic reviews. 2017;1:Cd006674. Epub 2017/01/04. doi: 10.1002/14651858.CD006674.pub3. PubMed PMID: 28046205; PubMed Central PMCID: PMCPMC6464792.
26. Lamina S, Agbanusi E. Effect of aerobic exercise training on maternal weight gain in pregnancy: a meta-analysis of randomized controlled trials. Ethiop J Health Sci. 2013;23(1):59-64. PubMed PMID: 23559839.
27. Streuling I, Beyerlein A, Rosenfeld E, Hofmann H, Schulz T, von Kries R. Physical activity and gestational weight gain: a meta-analysis of intervention trials. BJOG : an international journal of obstetrics and gynaecology. 2011;118(3):278-84. Epub 2010/12/08. doi: 10.1111/j.1471-0528.2010.02801.x. PubMed PMID: 21134106.
28. da Silva SG, Ricardo LI, Evenson KR, Hallal PC. Leisure-Time Physical Activity in Pregnancy and Maternal-Child Health: A Systematic Review and Meta-Analysis of Randomized Controlled Trials and Cohort Studies. Sports Med. 2017;47(2):295-317. Epub 2016/06/11. doi: 10.1007/s40279-016-0565-2. PubMed PMID: 27282925.
29. Wang J, Wen D, Liu X, Liu Y. Impact of exercise on maternal gestational weight gain: An updated meta-analysis of randomized controlled trials. Medicine (Baltimore). 2019;98(27):e16199-e. doi: 10.1097/MD.0000000000016199. PubMed PMID: 31277127.
30. Wiebe HW, Boulé NG, Chari R, Davenport MH. The Effect of Supervised Prenatal Exercise on Fetal Growth: A Meta-analysis. Obstetrics & Gynecology. 2015;125(5):1185-94. doi: 10.1097/aog.0000000000000801. PubMed PMID: 00006250-201505000-00028.
31. Elliott-Sale KJ, Barnett CT, Sale C. Exercise interventions for weight management during pregnancy and up to 1 year postpartum among normal weight, overweight and obese women: a systematic review and meta-analysis. Br J Sports Med. 2015;49(20):1336-42. Epub 2014/11/20. doi: 10.1136/bjsports-2014-093875. PubMed PMID: 25406335.
32. Ming W-K, Ding W, Zhang CJP, Zhong L, Long Y, Li Z, et al. The effect of exercise during pregnancy on gestational diabetes mellitus in normal-weight women: a systematic review and meta-analysis. BMC Pregnancy and Childbirth. 2018;18(1):440. doi: 10.1186/s12884-018-2068-7.
33. Sui Z, Grivell RM, Dodd JM. Antenatal exercise to improve outcomes in overweight or obese women: A systematic review. Acta Obstet Gynecol Scand. 2012;91(5):538-45. Epub 2012/01/11. doi: 10.1111/j.1600-0412.2012.01357.x. PubMed PMID: 22229625.
34. Chatzakis C, Goulis DG, Mareti E, Eleftheriades M, Zavlanos A, Dinas K, et al. Prevention of gestational diabetes mellitus in overweight or obese pregnant women: A network meta-analysis. Diabetes Res Clin Pract. 2019;158:107924. Epub 2019/11/19. doi: 10.1016/j.diabres.2019.107924. PubMed PMID: 31738997.
35. Du MC, Ouyang YQ, Nie XF, Huang Y, Redding SR. Effects of physical exercise during pregnancy on maternal and infant outcomes in overweight and obese pregnant women: A meta-analysis. Birth. 2019;46(2):211-21. Epub 2018/09/22. doi: 10.1111/birt.12396. PubMed PMID: 30240042.
36. Sanabria-Martínez G, García-Hermoso A, Poyatos-León R, Álvarez-Bueno C, Sánchez-López M, Martínez-Vizcaíno V. Effectiveness of physical activity interventions on preventing gestational diabetes mellitus and excessive maternal weight gain: a meta-analysis. BJOG : an international journal of obstetrics and gynaecology. 2015;122(9):1167-74. Epub 2015/06/04. doi: 10.1111/1471-0528.13429. PubMed PMID: 26036300.
37. Muktabhant B, Lawrie TA, Lumbiganon P, Laopaiboon M. Diet or exercise, or both, for preventing excessive weight gain in pregnancy. The Cochrane database of systematic reviews. 2015;(6):Cd007145. Epub 2015/06/13. doi: 10.1002/14651858.CD007145.pub3. PubMed PMID: 26068707.

38. Michel S, Raab R, Drabsch T, Günther J, Stecher L, Hauner H. Do lifestyle interventions during pregnancy have the potential to reduce long-term postpartum weight retention? A systematic review and meta-analysis. Obesity Reviews. 2019;20(4):527-42. doi: 10.1111/obr.12809.

S9 Tables: Meta-analysis for gestational diabetes-related outcomes reported by the included systematic reviews

Table S9a: Meta-analysis for gestational diabetes mellitus (GDM) reported by the included systematic reviews

Systematic review author, year	Included population or intervention subgroup	N studies	Sample size pooled data	Meta-analysis: GDM	Statistical significance
**Diet and/or Physical Activity**					
Bain *et al.* 2015 [1]	Any BMI	11	3,744	RR 0.93 (95% CI 0.71, 1.23)	Not significant
Madhuvrata *et al.* 2015 [2]	Any BMI	6	1,124	OR 1.44 (95% CI 0.96, 2.14)	Not significant p=0.07
Rogozińska *et al.* 2015 [3]	Any BMI	13	4,745	RR 0.95 (95% CI 0.76, 1.18)	Not significant p=0.65
Shepherd *et al.* 2017 [4]	Any BMI	19	6,633	RR 0.85 (95% CI 0.71, 1.01)	Not significant p=0.069
**Song *et al.* 2016 [5]**	**Any BMI**	**29**	**11,487**	**RR 0.82 (95% CI 0.70, 0.95)**	**Significantly reduced p=0.0091**
**Davenport *et al.* 2018b [6]**	**Any BMI**	**45**	**14,766**	**OR 0.76 (95% CI 0.65, 0.88)**	**Significantly reduced p=0.0004**
Bennett *et al.* 2017 [7]	Any BMI	19	7,178	RR 0.90 (95% CI 0.77, 1.05)	Not significant p=0.187
Tanentsapf *et al.* 2011 [8]	Recommended BMI, overweight, obese	6	886	RR 0.74 (95% CI 0.52, 1.06)	Not significant
Thangaratinam *et al.* 2012 [9]	Recommended BMI, overweight, obese	5	675	RR 0.71 (95% CI 0.44, 1.13)	Not significant p=0.55
Thangaratinam *et al.* 2012b [10]	Recommended BMI, overweight, obese	9	1,642	RR 0.78 (95% CI 0.57, 1.08)	Not significant p=0.13
IWiP 2017 [11]	Recommended BMI, overweight, obese	27	9,427	OR 0.89 (95% CI 0.72, 1.10) (IPD MA)	Not significant
**IWiP 2017 [11]**	**Recommended BMI, overweight, obese**	**59**	**16,885**	**OR 0.76 (95% CI 0.65, 0.89) (IPD + non-IPD)**	**Significantly reduced**
Bain *et al.* 2015 [1]	Recommended BMI	2	243	RR 0.91 (95% CI 0.19, 4.24)	Not significant
O'Brien *et al.* 2015 [12]	Recommended BMI	2	243	RR 1.02 (95% CI 0.41, 2.57)	Not significant
Song *et al.* 2016 [5]	Recommended BMI	8	3,962	RR 0.82 (95% CI 0.62, 1.10)	Not significant p=0.1983
Shepherd *et al.* 2017 [4]	Recommended BMI	3	300	RR 0.91 (95% CI 0.19, 4.24)	Not significant
Bennett *et al.* 2017 [7]	Recommended BMI	4	572	RR 0.79 (95% CI 0.51, 1.22)	Not significant
Bain *et al.* 2015 [1]	Overweight or obese	7	3,116	RR 0.90 (95% CI 0.63, 1.29)	Not significant
Oteng-Ntim *et al.* 2012 [13]	Overweight or obese	6	1,017	OR 0.80 (95% CI 0.58, 1.10)	Not significant p=0.17
Rogozińska *et al.* 2015 [3]	Overweight or obese	9	3,714	RR 1.02 (95% CI 0.86, 1.20)	Not significant p=0.44
Lau *et al.* 2017 [14]	Overweight or obese	2	1,447	RR 0.99 (95% CI 0.83, 1.19)	Not significant p=0.91
**Magro-Malosso *et al* 2017b [15]**	**Overweight or obese**	**7**	**1,350**	**RR 0.61 (95% CI 0.41, 0.90)**	**Significantly reduced**
Shepherd *et al.* 2017 [4]	Overweight or obese	8	2,901	RR 0.77 (95% CI 0.50, 1.20)	Not significant
**Song *et al.* 2016 [5]**	**Overweight or obese**	**18**	**7,040**	**RR 0.83 (95% CI 0.69, 1.00)**	**Significantly reduced p=0.05**
Bennett *et al.* 2017 [7]	Overweight or obese	14	5,404	RR 0.96 (95% CI 0.82, 1.14)	Not significant
Shepherd *et al.* 2017 [4]	Obese	3	1,738	RR 0.96 (95% CI 0.81, 1.13)	Not significant
**Diet only**					
Gresham *et al.* 2016 [16]	Any BMI	6	1,206	SMD −0.27 (95% CI −0.72, 0.17)	Not significant p=0.23
**Madhuvrata *et al.* 2015 [2]**	**Any BMI**	**3**	**409**	**OR 0.33 (95% CI 0.14, 0.76)**	**Significantly reduced p=0.009**
**Oostdam *et al.* 2011 [17]**	**Any BMI (counselling)**	**7**	**813**	**MD -0.05 (95% CI -0.10, -0.01)**	**Significantly reduced p=0.03**
Rogozińska *et al.* 2015 [3]	Any BMI	6	1,479	RR 0.67 (95% CI 0.39, 1.15)	Not significant p=0.15

Systematic review author, year	Included population or intervention subgroup	N studies	Sample size pooled data	Meta-analysis: GDM	Statistical significance
Song *et al.* 2016 [5]	Any BMI	5	1,279	RR 0.80 (95% CI 0.58, 1.10)	Not significant p=0.1658
Tieu *et al.* 2017 [18]	Any BMI	5	1,279	RR 0.60 (95% CI 0.35, 1.04)	Not significant p=0.07
**Tieu *et al.* 2017 [18]**	**Any BMI**	**3**	**409**	**RR 0.39 (95% CI 0.19, 0.79)**	**Significantly reduced**
**Bennett *et al.* 2017 [7]**	**Any BMI**	**9**	**2,805**	**RR 0.56 (95% CI 0.36, 0.87)**	**Significantly reduced p=0.009**
Thangaratinam *et al.* 2012 [9]	Recommended BMI, overweight, obese	2	285	RR 0.52 (95% CI 0.27, 1.03)	Not significant p=0.06
**Thangaratinam *et al.* 2012b [10]**	**Recommended BMI, overweight, obese**	**3**	**409**	**RR 0.39 (95% CI 0.23, 0.69)**	**Significantly reduced p=0.001**
IWiP 2017 [11]	Recommended BMI, overweight, obese	4	490	OR 1.03 (95% CI 0.30, 3.61) (IPD MA)	Not significant
IWiP 2017 [11]	Recommended BMI, overweight, obese	8	1,106	OR 0.79 (95% CI 0.37, 1.69) (IPD + non-IPD)	Not significant
**Rogozińska *et al.* 2015 [3]**	**Overweight or obese**	**3**	**455**	**RR 0.40 (95% CI 0.18, 0.86)**	**Significantly reduced p=0.04**
Bennett *et al.* 2017 [7]	Overweight or obese	2	282	RR 0.54 (95% CI 0.27, 1.07)	Not significant
**Physical activity only**					
Han *et al.* 2012 [19]	Any BMI	3	826	RR 1.10 (95% CI 0.66, 1.84)	Not significant p=0.71
Madhuvrata *et al.* 2015 [2]	Any BMI	3	152	OR 0.77 (95% CI 0.33, 1.79)	Not significant p=0.54
Oostdam *et al.* 2011 [17]	Any BMI	3	238	MD -0.05 (95% CI -0.20, 0.10)	Not significant p=0.51
**Russo *et al.* 2015 [20]**	**Any BMI**	**10**	**1,715**	**RR 0.74 (95% CI 0.57, 0.97)**	**Significantly reduced p=0.027**
**da Silva *et al.* 2017 [21]**	**Any BMI**	**10**	**3,790**	**RR 0.67 (95% CI 0.49, 0.92)**	**Significantly reduced**
Song *et al.* 2016 [5]	Any BMI	10	4,161	RR 0.77 (95% CI 0.54, 1.09)	Not significant p=0.1456
**Zheng *et al.* 2017 [22]**	**Any BMI**	**4**	**1,113**	**OR 0.62 (95% CI 0.43, 0.89)**	**Significantly reduced p=0.010**
**Davenport *et al.* 2018b [6]**	**Any BMI**	**25**	**6,934**	**OR 0.62 (95% CI 0.52, 0.75)**	**Significantly reduced p<0.00001**
Yin *et al.* 2013 [23]	Any BMI	5	947	RR 0.91 (95% CI 0.57, 1.44)	Not significant p=0.68
**Bennett *et al.* 2017 [7]**	**Any BMI**	**10**	**3,115**	**RR 0.62 (95% CI 0.50, 0.78)**	**Significantly reduced p<0.001**
**IWiP 2017 [11]**	**Recommended BMI, overweight, obese**	**10**	**2,700**	**OR 0.67 (95% CI 0.46, 0.99) (IPD MA)**	**Significantly reduced**
**IWiP 2017 [11]**	**Recommended BMI, overweight, obese**	**27**	**6,755**	**OR 0.66 (95% CI 0.53, 0.83) (IPD + non-IPD)**	**Significantly reduced**
**Di Mascio *et al.* 2016 [24]**	**Recommended BMI**	**4**	**1,686**	**RR 0.51 (95% CI 0.31, 0.82)**	**Significantly reduced**
**Ming *et al.* 2018 [25]**	**Recommended BMI**	**8**	**2,981**	**RR 0.58 (95% CI 0.37, 0.90)**	**Significantly reduced p=0.01**
Chatzakis *et al*. 2019 [26]	Overweight or obese	10	1,164	RR 0.80 (95% CI 0.60, 1.07)	Not significant
**Du *et al*. 2019 [27]**	**Overweight or obese**	**10**	**1,120**	**RR 0.71 (95% CI 0.57, 0.89)**	**Significantly reduced p=0.004**
Nasiri-Amiri *et al.* 2019 [28]	Overweight or obese	8	1,441	RR 0.76 (95% CI 0.56, 1.03)	Not significant p=0.07
Bennett *et al.* 2017 [7]	Overweight or obese	5	720	RR 0.62 (95% CI 0.37, 1.02)	Not significant
**Sanabria-Martínez *et al.* 2015 [29]**	**Not reported**	**8**	**2,501**	**RR 0.69 (95% CI 0.52, 0.91)**	**Significantly reduced p=0.009**

Footnote: GDM – gestational diabetes mellitus, BMI – body mass index, N – number, RR – risk ratio, OR – odds ratio, CI – confidence interval, IPD – individual participant data, MA – meta-analysis, MD – mean difference, SMD – standardised mean difference. Bold data highlights statistically significant results.

Table S9b: Meta-analysis for other outcomes related to gestational diabetes mellitus (GDM) reported by the included systematic reviews

Systematic review author, year	Type of intervention included in the reviews	Included population or intervention subgroup	Health outcome	N studies	Sample size pooled data	Meta-analysis: other GDM-related measures	Statistical significance
Oostdam *et al.* 2011 [17]	Diet only	Any BMI - Counselling	Fasting blood glucose	3	98	MD -0.21 (95% CI -0.45, 0.02)	Not significant p=0.07
Oostdam *et al.* 2011 [17]	Diet only	Any BMI - Low GI	Fasting blood glucose	3	114	MD -0.13 (95% CI -0.30, 0.04)	Not significant p=0.13
**Zhang *et al.* 2018 [30]**	**Diet only**	**Any BMI - Low GI**	**Fasting blood glucose**	**8**	**1,700**	**WMD −0.18 mmol/L (95% CI −0.33, −0.02)**	**Significantly reduced**
**Zhang *et al.* 2018 [30]**	**Diet only**	**Any BMI - Low GI**	**2-hour blood glucose**	**4**	**Not reported**	**WMD −0.33 mmol/L (95% CI −0.54, −0.12)**	**Significantly reduced**
**Davenport *et al.* 2018c [31]**	**Diet and/or Physical Activity**	**Any BMI**	**Blood glucose**	**13**	**3,688**	**MD -0.20mmol/L (95% CI -0.32, -0.07)**	**Significantly reduced**
**Davenport *et al.* 2018c [31]**	**Physical activity only**	**Any BMI**	**Blood glucose**	**12**	**2,244**	**MD -0.48mmol/L (95% CI -0.76, -0.19)**	**Significantly reduced**
Zheng *et al.* 2017 [22]	Physical activity only	Any BMI	Glucose 2-hour post OGTT	3	1,136	MD -1.02mmol/L (95% CI -2.75, 0.71)	Not significant p=0.25
Madhuvrata *et al.* 2015 [2]	Physical activity only	Any BMI	Fasting blood glucose	2	140	MD -0.12 (95% CI -0.41, 0.17)	Not significant p=0.43
Oostdam *et al.* 2011 [17]	Physical activity only	Any BMI	Fasting blood glucose	2	96	MD 0.07 (95% CI -0.08, 0.22)	Not significant p=0.35
Nasiri-Amiri *et al.* 2019 [28]	Physical activity only	Overweight or obese	Fasting plasma glucose	6	819	SMD 0.01 (95% CI -0.34, 0.36)	Not significant
Nasiri-Amiri *et al.* 2019 [28]	Physical activity only	Overweight or obese	Fasting plasma insulin	3	235	SMD -0.28 (95% CI -0.65, 0.08)	Not significant p=0.13
Sherifali *et al.* 2017 [32]	Diet and/or Physical Activity	Any BMI	HbA1C	2	201	MD 0.10 % (95% CI −0.08, 0.28)	Not significant p=0.29
Zhang *et al.* 2018 [30]	Diet only	Any BMI - Low GI	HbA1C	3	Not reported	WMD 0.02 % (95% CI −0.03, 0.08)	Not significant
Zhang *et al.* 2018 [30]	Diet only	Any BMI - Low GI	Insulin use	3	Not reported	RR 1.01 (95% CI 0.77, 1.33)	Not significant

Footnote: GDM – gestational diabetes mellitus, BMI – body mass index, N – number, RR – risk ratio, CI – confidence interval, IPD – individual participant data, MA – meta-analysis, MD – mean difference, SMD – standardised mean difference, WMD – weighted mean difference, OGTT – oral glucose tolerance test, HbA1C - hemoglobin A1C (glycosylated hemoglobin), low GI – low glycaemic index diet, mmol/L - millimoles per litre. Bold data highlights statistically significant results.

S9 References

1. Bain E, Crane M, Tieu J, Han S, Crowther CA, Middleton P. Diet and exercise interventions for preventing gestational diabetes mellitus. The Cochrane database of systematic reviews. 2015;(4):Cd010443. Epub 2015/04/13. doi: 10.1002/14651858.CD010443.pub2. PubMed PMID: 25864059.
2. Madhuvrata P, Govinden G, Bustani R, Song S, Farrell TA. Prevention of gestational diabetes in pregnant women with risk factors for gestational diabetes: a systematic review and meta-analysis of randomised trials. Obstet Med. 2015;8(2):68-85. Epub 2015/06/01. doi: 10.1177/1753495x15576673. PubMed PMID: 27512459; PubMed Central PMCID: PMCPMC4935009.
3. Rogozińska E, Chamillard M, Hitman GA, Khan KS, Thangaratinam S. Nutritional manipulation for the primary prevention of gestational diabetes mellitus: a meta-analysis of randomised studies. PloS one. 2015;10(2):e0115526. Epub 2015/02/27. doi: 10.1371/journal.pone.0115526. PubMed PMID: 25719363; PubMed Central PMCID: PMCPMC4342242.
4. Shepherd E, Gomersall JC, Tieu J, Han S, Crowther CA, Middleton P. Combined diet and exercise interventions for preventing gestational diabetes mellitus. The Cochrane database of systematic reviews. 2017;11:Cd010443. Epub 2017/11/13. doi: 10.1002/14651858.CD010443.pub3. PubMed PMID: 29129039; PubMed Central PMCID: PMCPMC6485974.

5. Song C, Li J, Leng J, Ma RC, Yang X. Lifestyle intervention can reduce the risk of gestational diabetes: a meta-analysis of randomized controlled trials. Obesity reviews : an official journal of the International Association for the Study of Obesity. 2016;17(10):960-9. Epub 2016/07/16. doi: 10.1111/obr.12442. PubMed PMID: 27417680.
6. Davenport MH, Ruchat SM, Poitras VJ, Jaramillo Garcia A, Gray CE, Barrowman N, et al. Prenatal exercise for the prevention of gestational diabetes mellitus and hypertensive disorders of pregnancy: a systematic review and meta-analysis. Br J Sports Med. 2018;52(21):1367-75. Epub 2018/10/20. doi: 10.1136/bjsports-2018-099355. PubMed PMID: 30337463.
7. Bennett CJ, Walker RE, Blumfield ML, Gwini S-M, Ma J, Wang F, et al. Interventions designed to reduce excessive gestational weight gain can reduce the incidence of gestational diabetes mellitus: A systematic review and meta-analysis of randomised controlled trials. Diabetes research and clinical practice. 2018;141:69-79. doi: 10.1016/j.diabres.2018.04.010. PubMed PMID: 29698713.
8. Tanentsapf I, Heitmann BL, Adegboye ARA. Systematic review of clinical trials on dietary interventions to prevent excessive weight gain during pregnancy among normal weight, overweight and obese women. BMC Pregnancy and Childbirth. 2011;11(1):81. doi: 10.1186/1471-2393-11-81.
9. Thangaratinam S, Rogozińska E, Jolly K, Glinkowski S, Duda W, Borowiack E, et al. Interventions to reduce or prevent obesity in pregnant women: a systematic review. 2012;16:31. doi: 10.3310/hta16310.
10. Thangaratinam S, Rogozińska E, Jolly K, Glinkowski S, Roseboom T, Tomlinson JW, et al. Effects of interventions in pregnancy on maternal weight and obstetric outcomes: meta-analysis of randomised evidence. BMJ : British Medical Journal. 2012;344:e2088. doi: 10.1136/bmj.e2088.
11. International Weight Management in Pregnancy (IWiP) collaboration. Effect of diet and physical activity based interventions in pregnancy on gestational weight gain and pregnancy outcomes: meta-analysis of individual participant data from randomised trials. BMJ (Clinical research ed). 2017;358:j3119. doi: 10.1136/bmj.j3119.
12. O'Brien CM, Grivell RM, Dodd JM. Systematic review of antenatal dietary and lifestyle interventions in women with a normal body mass index. Acta Obstet Gynecol Scand. 2016;95(3):259-69. Epub 2015/11/27. doi: 10.1111/aogs.12829. PubMed PMID: 26610169.
13. Oteng-Ntim E, Varma R, Croker H, Poston L, Doyle P. Lifestyle interventions for overweight and obese pregnant women to improve pregnancy outcome: systematic review and meta-analysis. BMC Medicine. 2012;10(1):47. doi: 10.1186/1741-7015-10-47.
14. Lau Y, Klainin-Yobas P, Htun TP, Wong SN, Tan KL, Ho-Lim ST, et al. Electronic-based lifestyle interventions in overweight or obese perinatal women: a systematic review and meta-analysis. Obesity reviews : an official journal of the International Association for the Study of Obesity. 2017;18(9):1071-87. Epub 2017/05/26. doi: 10.1111/obr.12557. PubMed PMID: 28544551.
15. Magro-Malosso ER, Saccone G, Di Mascio D, Di Tommaso M, Berghella V. Exercise during pregnancy and risk of preterm birth in overweight and obese women: a systematic review and meta-analysis of randomized controlled trials. Acta Obstet Gynecol Scand. 2017;96(3):263-73. Epub 2016/12/29. doi: 10.1111/aogs.13087. PubMed PMID: 28029178.
16. Gresham E, Bisquera A, Byles JE, Hure AJ. Effects of dietary interventions on pregnancy outcomes: a systematic review and meta-analysis. Matern Child Nutr. 2016;12(1):5-23. Epub 2014/07/23. doi: 10.1111/mcn.12142. PubMed PMID: 25048387; PubMed Central PMCID: PMCPMC6860081.
17. Oostdam N, van Poppel MN, Wouters MG, van Mechelen W. Interventions for preventing gestational diabetes mellitus: a systematic review and meta-analysis. Journal of women's health (2002). 2011;20(10):1551-63. Epub 2011/08/16. doi: 10.1089/jwh.2010.2703. PubMed PMID: 21838525.
18. Tieu J, Shepherd E, Middleton P, Crowther CA. Dietary advice interventions in pregnancy for preventing gestational diabetes mellitus. The Cochrane database of systematic reviews. 2017;1:Cd006674. Epub 2017/01/04. doi: 10.1002/14651858.CD006674.pub3. PubMed PMID: 28046205; PubMed Central PMCID: PMCPMC6464792.
19. Han S, Middleton P, Crowther CA. Exercise for pregnant women for preventing gestational diabetes mellitus. The Cochrane database of systematic reviews. 2012;(7):Cd009021. Epub 2012/07/13. doi: 10.1002/14651858.CD009021.pub2. PubMed PMID: 22786521.

20. Russo LM, Nobles C, Ertel KA, Chasan-Taber L, Whitcomb BW. Physical activity interventions in pregnancy and risk of gestational diabetes mellitus: a systematic review and meta-analysis. Obstet Gynecol. 2015;125(3):576-82. Epub 2015/03/03. doi: 10.1097/aog.0000000000000691. PubMed PMID: 25730218.
21. da Silva SG, Ricardo LI, Evenson KR, Hallal PC. Leisure-Time Physical Activity in Pregnancy and Maternal-Child Health: A Systematic Review and Meta-Analysis of Randomized Controlled Trials and Cohort Studies. Sports Med. 2017;47(2):295-317. Epub 2016/06/11. doi: 10.1007/s40279-016-0565-2. PubMed PMID: 27282925.
22. Zheng J, Wang H, Ren M. Influence of exercise intervention on gestational diabetes mellitus: a systematic review and meta-analysis. J Endocrinol Invest. 2017;40(10):1027-33. Epub 2017/04/13. doi: 10.1007/s40618-017-0673-3. PubMed PMID: 28401529.
23. Yin YN, Li XL, Tao TJ, Luo BR, Liao SJ. Physical activity during pregnancy and the risk of gestational diabetes mellitus: a systematic review and meta-analysis of randomised controlled trials. Br J Sports Med. 2014;48(4):290-5. Epub 2013/09/17. doi: 10.1136/bjsports-2013-092596. PubMed PMID: 24037671.
24. Di Mascio D, Magro-Malosso ER, Saccone G, Marhefka GD, Berghella V. Exercise during pregnancy in normal-weight women and risk of preterm birth: a systematic review and meta-analysis of randomized controlled trials. American journal of obstetrics and gynecology. 2016;215(5):561-71. Epub 2016/10/30. doi: 10.1016/j.ajog.2016.06.014. PubMed PMID: 27319364.
25. Ming W-K, Ding W, Zhang CJP, Zhong L, Long Y, Li Z, et al. The effect of exercise during pregnancy on gestational diabetes mellitus in normal-weight women: a systematic review and meta-analysis. BMC Pregnancy and Childbirth. 2018;18(1):440. doi: 10.1186/s12884-018-2068-7.
26. Chatzakis C, Goulis DG, Mareti E, Eleftheriades M, Zavlanos A, Dinas K, et al. Prevention of gestational diabetes mellitus in overweight or obese pregnant women: A network meta-analysis. Diabetes Res Clin Pract. 2019;158:107924. Epub 2019/11/19. doi: 10.1016/j.diabres.2019.107924. PubMed PMID: 31738997.
27. Du MC, Ouyang YQ, Nie XF, Huang Y, Redding SR. Effects of physical exercise during pregnancy on maternal and infant outcomes in overweight and obese pregnant women: A meta-analysis. Birth. 2019;46(2):211-21. Epub 2018/09/22. doi: 10.1111/birt.12396. PubMed PMID: 30240042.
28. Nasiri-Amiri F, Sepidarkish M, Shirvani MA, Habibipour P, Tabari NSM. The effect of exercise on the prevention of gestational diabetes in obese and overweight pregnant women: a systematic review and meta-analysis. Diabetology & Metabolic Syndrome. 2019;11(1):72. doi: 10.1186/s13098-019-0470-6.
29. Sanabria-Martínez G, García-Hermoso A, Poyatos-León R, Álvarez-Bueno C, Sánchez-López M, Martínez-Vizcaíno V. Effectiveness of physical activity interventions on preventing gestational diabetes mellitus and excessive maternal weight gain: a meta-analysis. BJOG : an international journal of obstetrics and gynaecology. 2015;122(9):1167-74. Epub 2015/06/04. doi: 10.1111/1471-0528.13429. PubMed PMID: 26036300.
30. Zhang R, Han S, Chen GC, Li ZN, Silva-Zolezzi I, Parés GV, et al. Effects of low-glycemic-index diets in pregnancy on maternal and newborn outcomes in pregnant women: a meta-analysis of randomized controlled trials. Eur J Nutr. 2018;57(1):167-77. Epub 2016/09/11. doi: 10.1007/s00394-016-1306-x. PubMed PMID: 27612876.
31. Davenport MH, Sobierajski F, Mottola MF, Skow RJ, Meah VL, Poitras VJ, et al. Glucose responses to acute and chronic exercise during pregnancy: a systematic review and meta-analysis. Br J Sports Med. 2018;52(21):1357-66. Epub 2018/10/20. doi: 10.1136/bjsports-2018-099829. PubMed PMID: 30337462.
32. Sherifali D, Nerenberg KA, Wilson S, Semeniuk K, Ali MU, Redman LM, et al. The Effectiveness of eHealth Technologies on Weight Management in Pregnant and Postpartum Women: Systematic Review and Meta-Analysis. Journal of medical Internet research. 2017;19(10):e337. Epub 2017/10/17. doi: 10.2196/jmir.8006. PubMed PMID: 29030327; PubMed Central PMCID: PMCPMC5660296.

S10 Tables: Meta-analysis for outcomes related to hypertensive disorders reported by the included systematic reviews

Table S10a: Meta-analysis for preeclampsia reported by the included systematic reviews

Systematic review author, year	Included population or intervention subgroup	N studies	Sample size pooled data	Meta-analysis: Preeclampsia	Statistical significance
**Diet and/or Physical Activity**					
Bain *et al.* 2015 [1]	Any BMI	6	3,070	RR 0.93 (95% CI 0.72, 1.19)	Not significant
Allen *et al.* 2014 [2]	Any BMI	6	1,478	RR 0.93 (95% CI 0.66, 1.32)	Not significant p=0.68
Madhuvrata *et al.* 2015 [3]	Any BMI	5	1,000	OR 1.08 (95% CI 0.72, 1.62)	Not significant p=0.71
Muktabhant *et al.* 2015 [4]	Any BMI	15	5,330	RR 0.95 (95% CI 0.77, 1.16)	Not significant
Rogozińska *et al.* 2015 [5]	Any BMI	7	3,793	RR 0.96 (95% CI 0.75, 1.24)	Not significant p=0.77
Shepherd *et al.* 2017 [6]	Any BMI	8	5,366	RR 0.98 (95% CI 0.79, 1.22)	Not significant p=0.84
Davenport *et al.* 2018b [7]	Any BMI	27	10,177	OR 0.89 (95% CI 0.73, 1.08)	Not significant p=0.25
Tanentsapf *et al.* 2011 [8]	Recommended BMI, overweight, obese	6	1,025	RR 0.78 (95% CI 0.58, 1.06)	Not significant
**Thangaratinam *et al.* 2012 [9]**	**Recommended BMI, overweight, obese**	**10**	**3,072**	**RR 0.74 (95% CI 0.59, 0.92)**	**Significantly reduced p=0.008**
**Thangaratinam *et al.* 2012b [10]**	**Recommended BMI, overweight, obese**	**10**	**3,342**	**RR 0.74 (95% CI 0.60, 0.92)**	**Significantly reduced p=0.006**
O'Brien *et al.* 2015 [11]	Recommended BMI	2	243	RR 0.34 (95% CI 0.10, 1.22)	Not significant
Shepherd *et al.* 2017 [6]	Recommended BMI	2	243	RR 0.34 (95% CI 0.10, 1.22)	Not significant
Shepherd *et al.* 2017 [6]	Overweight or obese	3	2,369	RR 1.12 (95% CI 0.82, 1.54)	Not significant
Syngelaki *et al.* 2019 [12]	Overweight or obese	14	5,410	RR 1.01 (95% CI 0.80, 1.27)	Not significant p=0.96
Shepherd *et al.* 2017 [6]	Obese	2	1,809	RR 0.92 (95% CI 0.64, 1.32)	Not significant
**Diet only**					
**Allen *et al.* 2014 [2]**	**Any BMI**	**6**	**2,695**	**RR 0.67 (95% CI 0.53, 0.85)**	**Significantly reduced p=0.001**
Madhuvrata *et al.* 2015 [3]	Any BMI	2	282	OR 0.59 (95% CI 0.23, 1.51)	Not significant p=0.27
Rogozińska *et al.* 2015 [5]	Any BMI	2	323	RR 0.66 (95% CI 0.27, 1.59)	Not significant p=0.36
Tieu *et al.* 2017 [13]	Any BMI	2	282	RR 0.61 (95% CI 0.25, 1.46)	Not significant p=0.27
**Thangaratinam *et al.* 2012 [9, 10][#]**	**Recommended BMI, overweight, obese**	**6**	**2,624**	**RR 0.67 (95% CI 0.53, 0.85)**	**Significantly reduced p=0.0009**
Syngelaki *et al.* 2019 [12]	Overweight or obese	11	5,023	RR 1.00 (95% CI 0.79, 1.27)	Not significant p=0.99
**Physical activity only**					
**Davenport *et al.* 2018b [7]**	**Any BMI**	**15**	**3,322**	**OR 0.59 (95% CI 0.37, 0.94)**	**Significantly reduced p=0.03**
da Silva *et al.* 2017 [14]	Any BMI	3	1,417	RR 0.93 (95% CI 0.55, 1.57)	Not significant
Zheng *et al.* 2017 [15]	Any BMI	2	1,009	OR 1.05 (95% CI 0.53, 2.07)	Not significant p=0.88
Chatzakis *et al.* 2019 [16]	Overweight or obese	6	726	RR 0.87 (95% CI 0.58, 1.32)	Not significant

Systematic review author, year	Included population or intervention subgroup	N studies	Sample size pooled data	Meta-analysis: Preeclampsia	Statistical significance
Du *et al*. 2019 [17]	Overweight or obese	4	596	RR 1.39 (95% CI 0.66, 2.93)	Not significant p=0.38
Syngelaki *et al.* 2019 [12]	Overweight or obese	3	387	RR 1.13 (95% CI 0.45, 2.86)	Not significant p=0.79

Footnote: BMI – body mass index, N – number, RR – risk ratio, OR – odds ratio, CI – confidence interval. Bold data highlights statistically significant results. #Thangaratinam *et al.* 2012 [9] and Thangaratinam *et al.* 2012b [10] reported the same meta-analysis. Bold data highlights statistically significant results.

Table S10b: Meta-analysis for hypertension reported by the included systematic reviews

Systematic review author, year	Included population or intervention subgroup	N studies	Sample size pooled data	Meta-analysis: Pregnancy induced hypertension	Statistical significance
**Diet and/or physical activity**					
Shepherd *et al.* 2017 [6]	Any BMI	4	810	RR 0.46 (95% CI 0.16, 1.29)	Not significant p=0.14
**Davenport *et al.* 2018b [7]**	**Any BMI**	**32**	**9,648**	**OR 0.81 (95% CI 0.65, 1.00)**	**Significantly reduced p=0.05**
Rogozińska *et al.* 2015 [5]	Any BMI	7	3,496	RR 0.93 (95% CI 0.68, 1.26)	Not significant p=0.63
**Muktabhant *et al.* 2015 [4]**	**Any BMI**	**11**	**5,162**	**RR 0.70 (95% CI 0.51, 0.96)**	**Significantly reduced**
Madhuvrata *et al.* 2015 [3]	Any BMI	3	301	OR 1.52 (95% CI 0.87, 2.66)	Not significant p=0.15
IWiP 2017 [18]	Recommended BMI, overweight, obese	22	9,618	OR 0.95 (95% CI 0.78, 1.16) (IPD MA)	Not significant
**IWiP 2017 [18]**	**Recommended BMI, overweight, obese**	**45**	**14,849**	**OR 0.85 (95% CI 0.71, 1.00) (IPD + non-IPD)**	**Significantly reduced**
Thangaratinam *et al.* 2012b [10]	Recommended BMI, overweight, obese	6	1,061	RR 0.89 (95% CI 0.64, 1.25)	Not significant p=0.51
Thangaratinam *et al.* 2012 [9]	Recommended BMI, overweight, obese	6	791	RR 0.77 (95% CI 0.54, 1.10)	Not significant p=0.15
**O'Brien *et al.* 2015 [11]**	**Recommended BMI**	**2**	**243**	**RR 0.34 (95% CI 0.13, 0.91)**	**Significantly reduced p=0.03**
Shepherd *et al.* 2017 [6]	Overweight or obese	5	2,781	RR 0.82 (95% CI 0.43, 1.58)	Not significant
Syngelaki *et al.* 2019 [12]	Overweight or obese	20	5,533	RR 0.87 (95% CI 0.70, 1.06)	Not significant p=0.17
**Diet only**					
**Tieu *et al.* 2017 [13]**	**Any BMI**	**2**	**282**	**RR 0.30 (95% CI 0.10, 0.88)**	**Significantly reduced p=0.029**
Rogozińska *et al.* 2015 [5]	Any BMI	2	3	RR 0.16 (95% CI 0.02, 1.11)	Not significant p=0.06
**Madhuvrata *et al.* 2015 [3]**	**Any BMI**	**2**	**282**	**OR 0.28 (95% CI 0.09, 0.86)**	**Significantly reduced p=0.03**
Gresham *et al.* 2016 [19]	Any BMI	7	1,602	SMD −0.12 (95% CI −0.30, 0.06)	Not significant p=0.20
IWiP 2017 [18]	Recommended BMI, overweight, obese	3	397	OR 0.59 (95% CI 0.07, 4.65) (IPD MA)	Not significant
IWiP 2017 [18]	Recommended BMI, overweight, obese	5	729	OR 0.57 (95% CI 0.18, 1.79) (IPD + non-IPD)	Not significant
**Thangaratinam *et al.* 2012 [9, 10][#]**	**Recommended BMI, overweight, obese**	**2**	**282**	**RR 0.30 (95% CI 0.10, 0.88)**	**Significantly reduced p=0.03**
Syngelaki *et al.* 2019 [12]	Overweight or obese	5	4,345	RR 0.84 (95% CI 0.63, 1.13)	Not significant p=0.25
**Physical activity only**					
**Davenport *et al.* 2018b [7]**	**Any BMI**	**22**	**5,316**	**OR 0.61 (95% CI 0.43, 0.85)**	**Significantly reduced p=0.003**
IWiP 2017 [18]	Recommended BMI, overweight, obese	7	2,565	OR 0.74 (95% CI 0.42, 1.33) (IPD MA)	Not significant
**IWiP 2017 [18]**	**Recommended BMI, overweight, obese**	**20**	**5,125**	**OR 0.68 (95% CI 0.49, 0.93) (IPD + non-IPD)**	**Significantly reduced**
**Di Mascio *et al.* 2016 [20]**	**Recommended BMI**	**3**	**1,305**	**RR 0.21 (95% CI 0.09, 0.45)**	**Significantly reduced**
Syngelaki *et al.* 2019 [12]	Overweight or obese	14	884	RR 0.80 (95% CI 0.53, 1.20)	Not significant p=0.27
Du *et al.* 2019 [17]	Overweight or obese	5	671	RR 0.63 (95% CI 0.38, 1.05)	Not significant p=0.08

Systematic review author, year	Included population or intervention subgroup	N studies	Sample size pooled data	Meta-analysis: Pregnancy induced hypertension	Statistical significance
Chatzakis *et al*. 2019 [16]	Overweight or obese	5	681	RR 0.63 (95% CI 0.37, 1.06)	Not significant

Footnote: BMI – body mass index, N – number, RR – risk ratio, OR – odds ratio, CI – confidence interval, SMD – standardised mean difference, IPD – individual participant data, MA – meta-analysis. Bold data highlights statistically significant results. [#]Thangaratinam *et al.* 2012 [9] and Thangaratinam *et al.* 2012b [10] reported the same meta-analysis. Bold data highlights statistically significant results.

Table S10c: Meta-analysis for other measures related to hypertensive disorders of pregnancy reported by the included systematic reviews

Systematic review author, year	Included population or intervention subgroup	Health outcome	N studies	Sample size pooled data	Meta-analysis result	Statistical significance
**Diet and/or Physical Activity**						
Shepherd *et al.* 2017 [6]	Any BMI	Severe preeclampsia/ HELLP/ eclampsia	2	2,088	RR 0.72 (95% CI 0.35, 1.46)	Not significant p=0.36
**Magro-Malosso *et al* 2017a [21]**	**Any BMI**	**Preeclampsia/pregnancy induced hypertension**	**7**	**2,517**	**RR 0.70 (95% CI 0.53, 0.93)**	**Significantly reduced p=0.01**
Lau *et al.* 2017 [22]	Overweight or obese	Preeclampsia/pregnancy induced hypertension	2	1,672	RR 1.18 (95% CI 0.78, 1.79)	Not significant p=0.43
**Diet only**						
**Gresham *et al.* 2016 [19]**	**Any BMI**	**Systolic BP**	**3**	**432**	**SMD −0.26 mmHg (95% CI −0.45, −0.07)**	**Significantly reduced p<0.001**
**Gresham *et al.* 2016 [19]**	**Any BMI**	**Diastolic BP**	**3**	**432**	**SMD −0.57 mmHg (95% CI −0.75, −0.38)**	**Significantly reduced p<0.001**

Footnote: BMI – body mass index, BP – blood pressure, N – number, RR – risk ratio, CI – confidence interval, SMD – standardised mean difference, mmHg - millimetres of mercury. Bold data highlights statistically significant results.

S10 References

1. Bain E, Crane M, Tieu J, Han S, Crowther CA, Middleton P. Diet and exercise interventions for preventing gestational diabetes mellitus. The Cochrane database of systematic reviews. 2015;(4):Cd010443. Epub 2015/04/13. doi: 10.1002/14651858.CD010443.pub2. PubMed PMID: 25864059.
2. Allen R, Rogozinska E, Sivarajasingam P, Khan KS, Thangaratinam S. Effect of diet- and lifestyle-based metabolic risk-modifying interventions on preeclampsia: a meta-analysis. Acta Obstetricia et Gynecologica Scandinavica. 2014;93(10):973-85. doi: 10.1111/aogs.12467.
3. Madhuvrata P, Govinden G, Bustani R, Song S, Farrell TA. Prevention of gestational diabetes in pregnant women with risk factors for gestational diabetes: a systematic review and meta-analysis of randomised trials. Obstet Med. 2015;8(2):68-85. Epub 2015/06/01. doi: 10.1177/1753495x15576673. PubMed PMID: 27512459; PubMed Central PMCID: PMCPMC4935009.
4. Muktabhant B, Lawrie TA, Lumbiganon P, Laopaiboon M. Diet or exercise, or both, for preventing excessive weight gain in pregnancy. The Cochrane database of systematic reviews. 2015;(6):Cd007145. Epub 2015/06/13. doi: 10.1002/14651858.CD007145.pub3. PubMed PMID: 26068707.
5. Rogozińska E, Chamillard M, Hitman GA, Khan KS, Thangaratinam S. Nutritional manipulation for the primary prevention of gestational diabetes mellitus: a meta-analysis of randomised studies. PloS one. 2015;10(2):e0115526. Epub 2015/02/27. doi: 10.1371/journal.pone.0115526. PubMed PMID: 25719363; PubMed Central PMCID: PMCPMC4342242.
6. Shepherd E, Gomersall JC, Tieu J, Han S, Crowther CA, Middleton P. Combined diet and exercise interventions for preventing gestational diabetes mellitus. The Cochrane database of systematic reviews. 2017;11:Cd010443. Epub 2017/11/13. doi: 10.1002/14651858.CD010443.pub3. PubMed PMID: 29129039; PubMed Central PMCID: PMCPMC6485974.

7. Davenport MH, Ruchat SM, Poitras VJ, Jaramillo Garcia A, Gray CE, Barrowman N, et al. Prenatal exercise for the prevention of gestational diabetes mellitus and hypertensive disorders of pregnancy: a systematic review and meta-analysis. Br J Sports Med. 2018;52(21):1367-75. Epub 2018/10/20. doi: 10.1136/bjsports-2018-099355. PubMed PMID: 30337463.
8. Tanentsapf I, Heitmann BL, Adegboye ARA. Systematic review of clinical trials on dietary interventions to prevent excessive weight gain during pregnancy among normal weight, overweight and obese women. BMC Pregnancy and Childbirth. 2011;11(1):81. doi: 10.1186/1471-2393-11-81.
9. Thangaratinam S, Rogozińska E, Jolly K, Glinkowski S, Duda W, Borowiack E, et al. Interventions to reduce or prevent obesity in pregnant women: a systematic review. 2012;16:31. doi: 10.3310/hta16310.
10. Thangaratinam S, Rogozińska E, Jolly K, Glinkowski S, Roseboom T, Tomlinson JW, et al. Effects of interventions in pregnancy on maternal weight and obstetric outcomes: meta-analysis of randomised evidence. BMJ : British Medical Journal. 2012;344:e2088. doi: 10.1136/bmj.e2088.
11. O'Brien CM, Grivell RM, Dodd JM. Systematic review of antenatal dietary and lifestyle interventions in women with a normal body mass index. Acta Obstet Gynecol Scand. 2016;95(3):259-69. Epub 2015/11/27. doi: 10.1111/aogs.12829. PubMed PMID: 26610169.
12. Syngelaki A, Sequeira Campos M, Roberge S, Andrade W, Nicolaides KH. Diet and exercise for preeclampsia prevention in overweight and obese pregnant women: systematic review and meta-analysis. The journal of maternal-fetal & neonatal medicine : the official journal of the European Association of Perinatal Medicine, the Federation of Asia and Oceania Perinatal Societies, the International Society of Perinatal Obstet. 2019;32(20):3495-501. Epub 2018/05/25. doi: 10.1080/14767058.2018.1481037. PubMed PMID: 29792061.
13. Tieu J, Shepherd E, Middleton P, Crowther CA. Dietary advice interventions in pregnancy for preventing gestational diabetes mellitus. The Cochrane database of systematic reviews. 2017;1:Cd006674. Epub 2017/01/04. doi: 10.1002/14651858.CD006674.pub3. PubMed PMID: 28046205; PubMed Central PMCID: PMCPMC6464792.
14. da Silva SG, Ricardo LI, Evenson KR, Hallal PC. Leisure-Time Physical Activity in Pregnancy and Maternal-Child Health: A Systematic Review and Meta-Analysis of Randomized Controlled Trials and Cohort Studies. Sports Med. 2017;47(2):295-317. Epub 2016/06/11. doi: 10.1007/s40279-016-0565-2. PubMed PMID: 27282925.
15. Zheng J, Wang H, Ren M. Influence of exercise intervention on gestational diabetes mellitus: a systematic review and meta-analysis. J Endocrinol Invest. 2017;40(10):1027-33. Epub 2017/04/13. doi: 10.1007/s40618-017-0673-3. PubMed PMID: 28401529.
16. Chatzakis C, Goulis DG, Mareti E, Eleftheriades M, Zavlanos A, Dinas K, et al. Prevention of gestational diabetes mellitus in overweight or obese pregnant women: A network meta-analysis. Diabetes Res Clin Pract. 2019;158:107924. Epub 2019/11/19. doi: 10.1016/j.diabres.2019.107924. PubMed PMID: 31738997.
17. Du MC, Ouyang YQ, Nie XF, Huang Y, Redding SR. Effects of physical exercise during pregnancy on maternal and infant outcomes in overweight and obese pregnant women: A meta-analysis. Birth. 2019;46(2):211-21. Epub 2018/09/22. doi: 10.1111/birt.12396. PubMed PMID: 30240042.
18. International Weight Management in Pregnancy (IWiP) collaboration. Effect of diet and physical activity based interventions in pregnancy on gestational weight gain and pregnancy outcomes: meta-analysis of individual participant data from randomised trials. BMJ (Clinical research ed). 2017;358:j3119. doi: 10.1136/bmj.j3119.
19. Gresham E, Bisquera A, Byles JE, Hure AJ. Effects of dietary interventions on pregnancy outcomes: a systematic review and meta-analysis. Matern Child Nutr. 2016;12(1):5-23. Epub 2014/07/23. doi: 10.1111/mcn.12142. PubMed PMID: 25048387; PubMed Central PMCID: PMCPMC6860081.
20. Di Mascio D, Magro-Malosso ER, Saccone G, Marhefka GD, Berghella V. Exercise during pregnancy in normal-weight women and risk of preterm birth: a systematic review and meta-analysis of randomized controlled trials. American journal of obstetrics and gynecology. 2016;215(5):561-71. Epub 2016/10/30. doi: 10.1016/j.ajog.2016.06.014. PubMed PMID: 27319364.
21. Magro-Malosso ER, Saccone G, Di Tommaso M, Roman A, Berghella V. Exercise during pregnancy and risk of gestational hypertensive disorders: a systematic review and meta-analysis. Acta Obstetricia et Gynecologica Scandinavica. 2017;96(8):921-31. doi: 10.1111/aogs.13151.

22. Lau Y, Klainin-Yobas P, Htun TP, Wong SN, Tan KL, Ho-Lim ST, et al. Electronic-based lifestyle interventions in overweight or obese perinatal women: a systematic review and meta-analysis. Obesity reviews : an official journal of the International Association for the Study of Obesity. 2017;18(9):1071-87. Epub 2017/05/26. doi: 10.1111/obr.12557. PubMed PMID: 28544551.

S11 Tables: Meta-analysis for outcomes related to mode of delivery reported by the included systematic reviews

Table S11a: Meta-analysis for caesarean delivery reported by the included systematic reviews

Systematic review author, year	Included population or intervention subgroup	N studies	Sample size pooled data	Meta-analysis: Caesarean delivery	Statistical significance
**Diet and/or Physical Activity**					
Bain *et al.* 2015 [1]	Any BMI	7	3,246	RR 0.92 (95% CI 0.83, 1.01)	Not significant
Madhuvrata *et al.* 2015 [2]	Any BMI	4	605	OR 0.90 (95% CI 0.52, 1.55)	Not significant p=0.70
Muktabhant *et al.* 2015 [3]	Any BMI	78	7,534	RR 0.95 (95% CI 0.88, 1.03)	Not significant
Rogozińska *et al.* 2015 [4]	Any BMI	10	4,194	RR 0.91 (95% CI 0.82, 1.02)	Not significant p=0.10
**Magro-Malosso *et al* 2017a [5]**	**Any BMI**	**14**	**2,224**	**RR 0.84 (95% CI 0.73, 0.98)**	**Significantly reduced**
Shepherd *et al.* 2017 [6]	Any BMI	14	6,089	RR 0.95 (95% CI 0.88, 1.02)	Not significant p=0.15
**Davenport *et al.* 2019 [7]**	**Any BMI**	**68**	**15,888**	**OR 0.87 (95% CI 0.80, 0.96)**	**Significantly reduced p=0.005**
Tanentsapf *et al.* 2011 [8]	Recommended BMI, overweight, obese	6	841	RR 0.82 (0.60, 1.09)	Not significant
Thangaratinam *et al.* 2012 [9]	Recommended BMI, overweight, obese	14	3,312	RR 0.93 (95% CI 0.85, 1.03)	Not significant p=0.15
Thangaratinam *et al.* 2012b [10]	Recommended BMI, overweight, obese	18	4,222	RR 0.93 (95% CI 0.85, 1.01)	Not significant p=0.10
**IWiP 2017 [11]**	**Recommended BMI, overweight, obese**	**32**	**11,410**	**OR 0.91 (95% CI 0.83, 0.99) (IPD MA)**	**Significantly reduced**
**IWiP 2017 [11]**	**Recommended BMI, overweight, obese**	**66**	**18,041**	**OR 0.89 (95% CI 0.83, 0.96) (IPD + non-IPD)**	**Significantly reduced**
Bain *et al.* 2015 [1]	Recommended BMI	2	243	RR 0.92 (95% CI 0.58, 1.45)	Not significant
O'Brien *et al.* 2015 [12]	Recommended BMI	2	243	RR 0.92 (95% CI 0.58, 1.45)	Not significant
Shepherd *et al.* 2017 [6]	Recommended BMI	3	300	RR 0.92 (95% CI 0.58, 1.45)	Not significant
Bain *et al.* 2015 [1]	Overweight or obese	4	2,662	RR 0.93 (95% CI 0.84, 1.04)	Not significant
Oteng-Ntim *et al.* 2012 [13]	Overweight or obese	6	663	OR 0.96 (95% CI 0.68, 1.36)	Not significant p=0.82
Lau *et al.* 2017 [14]	Overweight or obese	2	1,689	RR 0.96 (95% CI 0.85, 1.09)	Not significant p=0.52
Magro-Malosso *et al* 2017b [15]	Overweight or obese	7	1,208	RR 0.93 (95% CI 0.77, 1.10)	Not significant
Shepherd *et al.* 2017 [6]	Overweight or obese	7	2,662	RR 0.91 (95% CI 0.83, 1.01)	Not significant
Shepherd *et al.* 2017 [6]	Obese	2	1,826	RR 0.99 (95% CI 0.87, 1.12)	Not significant
**Diet only**					
Gresham *et al.* 2016 [16]	Any BMI	6	1,347	SMD −0.02 (95% CI −0.17, 0.14)	Not significant p=0.85
Rogozińska *et al.* 2015 [4]	Any BMI	3	494	RR 1.17 (95% CI 0.99, 1.38)	Not significant p=0.06
Tieu *et al.* 2017 [17]	Any BMI	4	1,194	RR 0.98 (95% CI 0.78, 1.24)	Not significant p=0.87
Zhang *et al.* 2018 [18]	Any BMI - Low GI	5	Not reported	RR 1.07 (95% CI 0.75, 1.53)	Not significant
Muktabhant *et al.* 2015 [3]	Any BMI - Low GI	2	133	RR 0.99 (95% CI 0.33, 3.01)	Not significant
Thangaratinam *et al.* 2012 [9] [10][#]	Recommended BMI, overweight, obese	5	2,273	RR 0.93 (95% CI 0.84, 1.04)	Not significant p=0.19

Systematic review author, year	Included population or intervention subgroup	N studies	Sample size pooled data	Meta-analysis: Caesarean delivery	Statistical significance
IWiP 2017 [11]	Recommended BMI, overweight, obese	4	1,340	OR 0.78 (95% CI 0.50, 1.22) (IPD MA)	Not significant
IWiP 2017 [11]	Recommended BMI, overweight, obese	7	1,732	OR 0.88 (95% CI 0.65, 1.17) (IPD + non-IPD)	Not significant
**Physical activity only**					
Han *et al.* 2012 [19]	Any BMI	2	934	RR 1.33 (95% CI 0.97, 1.84)	Not significant p=0.081
Davenport *et al.* 2019 [7]	Any BMI	47	8,000	OR 0.91 (95% CI 0.79, 1.05)	Not significant p=0.19
Poyatos-Leon *et al.* 2015 [20]	Any BMI	10	3,160	RR 0.78 (95% CI 0.58, 1.05)	Not significant p=0.105
**Wiebe *et al.* 2015 [21]**	**Any BMI**	**23**	**4,414**	**OR 0.80 (95% CI 0.69, 0.94)**	**Significantly reduced p=0.006**
Thangaratinam *et al.* 2012 [9]	Recommended BMI, overweight, obese	4	475	RR 0.92 (95% CI 0.68, 1.24)	Not significant p=0.57
Thangaratinam *et al.* 2012b [10]	Recommended BMI, overweight, obese	5	542	RR 0.88 (95% CI 0.66, 1.17)	Not significant p=0.38
IWiP 2017 [11]	Recommended BMI, overweight, obese	13	3,046	OR 0.82 (95% CI 0.67, 1.01) (IPD MA)	Not significant
**IWiP 2017 [11]**	**Recommended BMI, overweight, obese**	**32**	**6,587**	**OR 0.83 (95% CI 0.73, 0.95) (IPD + non-IPD)**	**Significantly reduced**
**Di Mascio *et al.* 2016 [22]**	**Recommended BMI**	**6**	**2,150**	**RR 0.82 (95% CI 0.69, 0.97)**	**Significantly reduced**
Ming *et al.* 2018 [23]	Recommended BMI	7	2,691	OR 0.88 (95% CI 0.72, 1.08)	Not significant p=0.21
Chatzakis *et al*. 2019 [24]	Overweight or obese	9	773	RR 0.99 (95% CI 0.85, 1.17)	Not significant
Du *et al*. 2019 [25]	Overweight or obese	10	982	RR 1.02 (95% CI 0.87, 1.20)	Not significant p=0.79
Wiebe *et al.* 2015 [21]	Overweight or obese	3	417	OR 0.82 (95% CI 0.53, 1.29)	Not significant p=0.40

Footnote: BMI – body mass index, N – number, RR – risk ratio, OR – odds ratio, CI – confidence interval, IPD – individual participant data, MA – meta-analysis, SMD – standardised mean difference. # Thangaratinam *et al.* 2012 [9] and Thangaratinam *et al.* 2012b [10] reported the same meta-analysis data in both systematic reviews. Bold data highlights statistically significant results.

Table S11b: Meta-analysis for induction of labour reported by the included systematic reviews

Systematic review author, year	Included population or intervention subgroup	N studies	Sample size pooled data	Meta-analysis: Induction of labour	Statistical significance
**Diet and/or Physical Activity**					
Bain *et al.* 2015 [1]	Any BMI	2	2,193	RR 1.02 (95% CI 0.91, 1.14)	Not significant
Shepherd *et al.* 2017 [6]	Any BMI	5	3,907	RR 0.92 (95% CI 0.79, 1.06)	Not significant p=0.24
Rogozińska *et al.* 2015 [4]	Any BMI	4	2,689	RR 1.02 (95% CI 0.91, 1.13)	Not significant p=0.78
Muktabhant *et al.* 2015 [3]	Any BMI	8	3,832	RR 1.06 (95% CI 0.94, 1.19)	Not significant
**Thangaratinam *et al.* 2012 [9, 10]**	**Recommended BMI, overweight, obese**	**5**	**2,362**	**RR 1.12 (95% CI 1.00, 1.26)**	**Significantly increased p=0.05**
**Diet only**					
Tieu *et al.* 2017 [17]	Any BMI	2	991	RR 1.10 (95% CI 0.48, 2.51)	Not significant p=0.82
Rogozińska *et al.* 2015 [4]	Any BMI	2	1,057	RR 1.14 (95% CI 0.54, 2.40)	Not significant p=0.74
Thangaratinam *et al.* 2012 [9, 10]	Recommended BMI, overweight, obese	4	2,277	RR 1.12 (95% CI 0.99, 1.27)	Not significant p=0.07

Footnote: BMI – body mass index, N – number, RR – risk ratio, CI – confidence interval. # Thangaratinam *et al.* 2012 [9] and Thangaratinam *et al.* 2012b [10] reported the same meta-analysis data in both systematic reviews. Bold data highlights statistically significant results.

Table S11c: Meta-analysis for instrumental vaginal delivery reported by the included systematic reviews

Systematic review author, year	Included population or intervention subgroup	N studies	Sample size pooled data	Meta-analysis: Instrumental/operative vaginal delivery	Statistical significance
**Diet and/or Physical Activity**					
Davenport *et al.* 2019 [7]	Any BMI	24	7,103	OR 0.90 (95% CI 0.78, 1.03)	Not significant p=0.13
Shepherd *et al.* 2017 [6]	Any BMI	3	2,164	RR 1.07 (95% CI 0.86, 1.34)	Not significant p=0.52
**Physical activity only**					
**Davenport *et al.* 2019 [7]**	**Any BMI**	**19**	**3,819**	**OR 0.76 (95% CI 0.63, 0.92)**	**Significantly reduced p=0.004**
Poyatos-Leon *et al.* 2015 [20]	Any BMI	6	1,6376	RR 0.88 (95% CI 0.68 to 1.15)	Not significant p=0.365
Han *et al.* 2012 [19]	Any BMI	2	934	RR 0.83 (95% CI 0.58, 1.17)	Not significant p=0.29
Di Mascio *et al.* 2016 [22]	Recommended BMI	4	1,393	RR 0.78 (95% CI 0.61, 1.01)	Not significant

Footnote: BMI – body mass index, N – number, RR – risk ratio, OR – odds ratio, CI – confidence interval. Bold data highlights statistically significant results.

Table S11d: Meta-analysis for vaginal delivery reported by the included systematic reviews

Systematic review author, year	Included population or intervention subgroup	N studies	Sample size pooled data	Meta-analysis: Vaginal delivery	Statistical significance
**Diet and/or Physical Activity**					
Thangaratinam *et al.* 2012b [10]	Recommended BMI, overweight, obese	6	994	RR 1.00 (95% CI 0.94, 1.07)	Not significant p=0.91
Thangaratinam *et al.* 2012 [9]	Recommended BMI, overweight, obese	5	927	RR 1.00 (95% CI 0.94, 1.07)	Not significant p=1.00
**Diet only**					
Thangaratinam *et al.* 2012 [9, 10]	Recommended BMI, overweight, obese	2	472	RR 0.97 (95% CI 0.89, 1.07)	Not significant p=0.56
**Physical activity only**					
**Poyatos-Leon *et al.* 2015 [20]**	**Any BMI**	**8**	**1,770**	**RR 1.12 (95% CI 1.01 to 1.24)**	**Significantly increased p=0.041**
Thangaratinam *et al.* 2012 [9]	Recommended BMI, overweight, obese	2	421	RR 1.01 (95% CI 0.92, 1.11)	Not significant p=0.81
Thangaratinam *et al.* 2012b [10]	Recommended BMI, overweight, obese	3	488	RR 1.02 (95% CI 0.93, 1.11)	Not significant p=0.70
**Di Mascio *et al.* 2016 [22]**	**Recommended BMI**	**6**	**2,150**	**RR 1.09 (95% CI 1.04, 1.15)**	**Significantly increased**

Footnote: BMI – body mass index, N – number, RR – risk ratio, CI – confidence interval. # Thangaratinam *et al.* 2012 [9] and Thangaratinam *et al.* 2012b [10] reported the same meta-analysis data in both systematic reviews. Bold data highlights statistically significant results.

S11 References

1. Bain E, Crane M, Tieu J, Han S, Crowther CA, Middleton P. Diet and exercise interventions for preventing gestational diabetes mellitus. The Cochrane database of systematic reviews. 2015;(4):Cd010443. Epub 2015/04/13. doi: 10.1002/14651858.CD010443.pub2. PubMed PMID: 25864059.
2. Madhuvrata P, Govinden G, Bustani R, Song S, Farrell TA. Prevention of gestational diabetes in pregnant women with risk factors for gestational diabetes: a systematic review and meta-analysis of randomised trials. Obstet Med. 2015;8(2):68-85. Epub 2015/06/01. doi: 10.1177/1753495x15576673. PubMed PMID: 27512459; PubMed Central PMCID: PMCPMC4935009.
3. Muktabhant B, Lawrie TA, Lumbiganon P, Laopaiboon M. Diet or exercise, or both, for preventing excessive weight gain in pregnancy. The Cochrane database of systematic reviews. 2015;(6):Cd007145. Epub 2015/06/13. doi: 10.1002/14651858.CD007145.pub3. PubMed PMID: 26068707.
4. Rogozińska E, Chamillard M, Hitman GA, Khan KS, Thangaratinam S. Nutritional manipulation for the primary prevention of gestational diabetes mellitus: a meta-analysis of randomised studies. PloS one. 2015;10(2):e0115526. Epub 2015/02/27. doi: 10.1371/journal.pone.0115526. PubMed PMID: 25719363; PubMed Central PMCID: PMCPMC4342242.
5. Magro-Malosso ER, Saccone G, Di Tommaso M, Roman A, Berghella V. Exercise during pregnancy and risk of gestational hypertensive disorders: a systematic review and meta-analysis. Acta Obstetricia et Gynecologica Scandinavica. 2017;96(8):921-31. doi: 10.1111/aogs.13151.
6. Shepherd E, Gomersall JC, Tieu J, Han S, Crowther CA, Middleton P. Combined diet and exercise interventions for preventing gestational diabetes mellitus. The Cochrane database of systematic reviews. 2017;11:Cd010443. Epub 2017/11/13. doi: 10.1002/14651858.CD010443.pub3. PubMed PMID: 29129039; PubMed Central PMCID: PMCPMC6485974.

7. Davenport MH, Ruchat SM, Sobierajski F, Poitras VJ, Gray CE, Yoo C, et al. Impact of prenatal exercise on maternal harms, labour and delivery outcomes: a systematic review and meta-analysis. Br J Sports Med. 2019;53(2):99-107. Epub 2018/10/20. doi: 10.1136/bjsports-2018-099821. PubMed PMID: 30337349.
8. Tanentsapf I, Heitmann BL, Adegboye ARA. Systematic review of clinical trials on dietary interventions to prevent excessive weight gain during pregnancy among normal weight, overweight and obese women. BMC Pregnancy and Childbirth. 2011;11(1):81. doi: 10.1186/1471-2393-11-81.
9. Thangaratinam S, Rogozińska E, Jolly K, Glinkowski S, Duda W, Borowiack E, et al. Interventions to reduce or prevent obesity in pregnant women: a systematic review. 2012;16:31. doi: 10.3310/hta16310.
10. Thangaratinam S, Rogozińska E, Jolly K, Glinkowski S, Roseboom T, Tomlinson JW, et al. Effects of interventions in pregnancy on maternal weight and obstetric outcomes: meta-analysis of randomised evidence. BMJ : British Medical Journal. 2012;344:e2088. doi: 10.1136/bmj.e2088.
11. International Weight Management in Pregnancy (IWiP) collaboration. Effect of diet and physical activity based interventions in pregnancy on gestational weight gain and pregnancy outcomes: meta-analysis of individual participant data from randomised trials. BMJ (Clinical research ed). 2017;358:j3119. doi: 10.1136/bmj.j3119.
12. O'Brien CM, Grivell RM, Dodd JM. Systematic review of antenatal dietary and lifestyle interventions in women with a normal body mass index. Acta Obstet Gynecol Scand. 2016;95(3):259-69. Epub 2015/11/27. doi: 10.1111/aogs.12829. PubMed PMID: 26610169.
13. Oteng-Ntim E, Varma R, Croker H, Poston L, Doyle P. Lifestyle interventions for overweight and obese pregnant women to improve pregnancy outcome: systematic review and meta-analysis. BMC Medicine. 2012;10(1):47. doi: 10.1186/1741-7015-10-47.
14. Lau Y, Klainin-Yobas P, Htun TP, Wong SN, Tan KL, Ho-Lim ST, et al. Electronic-based lifestyle interventions in overweight or obese perinatal women: a systematic review and meta-analysis. Obesity reviews : an official journal of the International Association for the Study of Obesity. 2017;18(9):1071-87. Epub 2017/05/26. doi: 10.1111/obr.12557. PubMed PMID: 28544551.
15. Magro-Malosso ER, Saccone G, Di Mascio D, Di Tommaso M, Berghella V. Exercise during pregnancy and risk of preterm birth in overweight and obese women: a systematic review and meta-analysis of randomized controlled trials. Acta Obstet Gynecol Scand. 2017;96(3):263-73. Epub 2016/12/29. doi: 10.1111/aogs.13087. PubMed PMID: 28029178.
16. Gresham E, Bisquera A, Byles JE, Hure AJ. Effects of dietary interventions on pregnancy outcomes: a systematic review and meta-analysis. Matern Child Nutr. 2016;12(1):5-23. Epub 2014/07/23. doi: 10.1111/mcn.12142. PubMed PMID: 25048387; PubMed Central PMCID: PMCPMC6860081.
17. Tieu J, Shepherd E, Middleton P, Crowther CA. Dietary advice interventions in pregnancy for preventing gestational diabetes mellitus. The Cochrane database of systematic reviews. 2017;1:Cd006674. Epub 2017/01/04. doi: 10.1002/14651858.CD006674.pub3. PubMed PMID: 28046205; PubMed Central PMCID: PMCPMC6464792.
18. Zhang R, Han S, Chen GC, Li ZN, Silva-Zolezzi I, Parés GV, et al. Effects of low-glycemic-index diets in pregnancy on maternal and newborn outcomes in pregnant women: a meta-analysis of randomized controlled trials. Eur J Nutr. 2018;57(1):167-77. Epub 2016/09/11. doi: 10.1007/s00394-016-1306-x. PubMed PMID: 27612876.
19. Han S, Middleton P, Crowther CA. Exercise for pregnant women for preventing gestational diabetes mellitus. The Cochrane database of systematic reviews. 2012;(7):Cd009021. Epub 2012/07/13. doi: 10.1002/14651858.CD009021.pub2. PubMed PMID: 22786521.
20. Poyatos-León R, García-Hermoso A, Sanabria-Martínez G, Álvarez-Bueno C, Sánchez-López M, Martínez-Vizcaíno V. Effects of exercise during pregnancy on mode of delivery: a meta-analysis. Acta Obstet Gynecol Scand. 2015;94(10):1039-47. Epub 2015/05/13. doi: 10.1111/aogs.12675. PubMed PMID: 25965378.
21. Wiebe HW, Boulé NG, Chari R, Davenport MH. The Effect of Supervised Prenatal Exercise on Fetal Growth: A Meta-analysis. Obstetrics & Gynecology. 2015;125(5):1185-94. doi: 10.1097/aog.0000000000000801. PubMed PMID: 00006250-201505000-00028.
22. Di Mascio D, Magro-Malosso ER, Saccone G, Marhefka GD, Berghella V. Exercise during pregnancy in normal-weight women and risk of preterm birth: a systematic review and meta-analysis of randomized controlled trials. American journal of obstetrics and gynecology. 2016;215(5):561-71. Epub 2016/10/30. doi: 10.1016/j.ajog.2016.06.014. PubMed PMID: 27319364.

23. Ming W-K, Ding W, Zhang CJP, Zhong L, Long Y, Li Z, et al. The effect of exercise during pregnancy on gestational diabetes mellitus in normal-weight women: a systematic review and meta-analysis. BMC Pregnancy and Childbirth. 2018;18(1):440. doi: 10.1186/s12884-018-2068-7.
24. Chatzakis C, Goulis DG, Mareti E, Eleftheriades M, Zavlanos A, Dinas K, et al. Prevention of gestational diabetes mellitus in overweight or obese pregnant women: A network meta-analysis. Diabetes Res Clin Pract. 2019;158:107924. Epub 2019/11/19. doi: 10.1016/j.diabres.2019.107924. PubMed PMID: 31738997.
25. Du MC, Ouyang YQ, Nie XF, Huang Y, Redding SR. Effects of physical exercise during pregnancy on maternal and infant outcomes in overweight and obese pregnant women: A meta-analysis. Birth. 2019;46(2):211-21. Epub 2018/09/22. doi: 10.1111/birt.12396. PubMed PMID: 30240042.

Table S12: Meta-analysis for other measures of maternal health reported by the included systematic reviews

Systematic review author, year	Type of intervention included in the reviews	Included population or intervention subgroup	N studies	Sample size pooled data	Meta-analysis result	Statistical significance
**PPH**						
Muktabhant *et al.* 2015 [1]	Diet and/or Physical Activity	Any BMI	2	2,901	RR 0.94 (95% CI 0.78, 1.14)	Not significant
Shepherd *et al.* 2017 [2]	Diet and/or Physical Activity	Any BMI	3	4,235	RR 1.03 (95% CI 0.89, 1.18)	Not significant
Thangaratinam *et al.* 2012 [3, 4][#]	Diet and/or Physical Activity	Recommended BMI, overweight, obese	2	1,232	RR 0.90 (95% CI 0.57, 1.42)	Not significant p=0.64
Tieu *et al.* 2017 [5]	Diet only	Any BMI	2	991	RR 0.71 (95% CI 0.28, 1.86)	Not significant p=0.49
**Composite maternal outcome: gestational diabetes, hypertensive disorders of pregnancy, preterm delivery, caesarean**						
IWiP 2017 [6]	Diet and/or physical activity	Recommended BMI, overweight, obese	24	8,851	OR 0.90 (95% CI 0.79, 1.03)	Not significant
IWiP 2017 [6]	Diet only	Recommended BMI, overweight, obese	3	397	OR 0.60 (95% CI 0.20, 1.75)	Not significant
IWiP 2017 [6]	Physical activity only	Recommended BMI, overweight, obese	9	6,259	OR 0.81 (95% CI 0.61, 1.09)	Not significant
**Low back pain score**						
Thangaratinam *et al.* 2012 [3]	Diet and/or Physical Activity	Recommended BMI, overweight, obese	2	302	MD 0.16 (95% CI -10.16, 10.48)	Not significant p=0.98
**Perineal trauma**						
Shepherd *et al.* 2017 [2]	Diet and/or Physical Activity	Any BMI	3	2,733	RR 1.27 (95% CI 0.78, 2.05)	Not significant p=0.33
**Prenatal mental health**						
**Davenport *et al.* 2018a [7]**	**Diet and/or Physical Activity**	**Any BMI**	**17**	**3,316**	**SMD -0.23 (95% CI -0.36, -0.09) (depressive symptoms)**	**Significantly reduced p=0.002**
**Davenport *et al.* 2018a [7]**	**Physical activity only**	**Any BMI**	**11**	**1,076**	**SMD -0.38 (95% CI -0.51, -0.25) (depressive symptoms)**	**Significantly reduced p<0.00001**
**Davenport *et al.* 2018a [7]**	**Diet and/or Physical Activity**	**Any BMI**	**7**	**2,481**	**OR 0.55 (95% CI 0.34, 0.90) (depression)**	**Significantly reduced p=0.02**
Lau *et al.* 2017 [8]	Diet and/or Physical Activity	Overweight or obese	2	289	MD in score -0.06 (95% CI -0.29, 0.17) (depression)	Not significant p=0.61
**Davenport *et al.* 2018a [7]**	**Physical activity only**	**Any BMI**	**4**	**683**	**OR 0.33 (95% CI 0.21, 0.53) (depression)**	**Significantly reduced p<0.00001**
Davenport *et al.* 2018a [7]	Diet and/or Physical Activity	Any BMI	7	1,689	SMD 0.06 (95% CI -0.04, 0.15) (state anxiety symptoms)	Not significant
Davenport *et al.* 2018a [7]	Physical activity only	Any BMI	5	276	SMD 0.03 (95% CI -0.21, 0.27) (state anxiety symptoms)	Not significant
Davenport *et al.* 2018a [7]	Diet and/or Physical Activity	Any BMI	2	1,534	OR 1.12 (95% CI 0.85, 1.48) (state anxiety)	Not significant
Davenport *et al.* 2018a [7]	Physical activity only	Any BMI	2	90	SMD -0.21 (95% CI -0.63, 0.20) (trait anxiety symptoms)	Not significant
**Postnatal mental health**						
Davenport *et al.* 2018a [7]	Diet and/or Physical Activity	Any BMI	7	2,795	SMD 0.05 (95% CI -0.02, 0.12) (depressive symptoms)	Not significant p=0.18
Davenport *et al.* 2018a [7]	Physical activity only	Any BMI	4	1,006	SMD -0.01 (95% CI -0.13, 0.12) (depressive symptoms)	Not significant p=0.93
Davenport *et al.* 2018a [7]	Diet and/or Physical Activity	Any BMI	4	2,536	OR 0.91 (95% CI 0.61, 1.36) (depression)	Not significant
Davenport *et al.* 2018a [7]	Physical activity only	Any BMI	2	793	OR 0.48 (95% CI 0.18, 1.22) (depression)	Not significant
Davenport *et al.* 2018a [7]	Diet and/or Physical Activity	Any BMI	2	1,299	SMD 0.01 (95% CI -0.10, 0.12) (state anxiety symptoms)	Not significant

Footnote: BMI – body mass index, N – number, RR – risk ratio, OR – odds ratio, CI – confidence intervalMD – mean difference, SMD – standardised mean difference. Bold data highlights statistically significant results.

[#]Thangaratinam *et al.* 2012 [3] and Thangaratinam *et al.* 2012b [4] reported the same meta-analysis.

S12 References

1. Muktabhant B, Lawrie TA, Lumbiganon P, Laopaiboon M. Diet or exercise, or both, for preventing excessive weight gain in pregnancy. The Cochrane database of systematic reviews. 2015;(6):Cd007145. Epub 2015/06/13. doi: 10.1002/14651858.CD007145.pub3. PubMed PMID: 26068707.
2. Shepherd E, Gomersall JC, Tieu J, Han S, Crowther CA, Middleton P. Combined diet and exercise interventions for preventing gestational diabetes mellitus. The Cochrane database of systematic reviews. 2017;11:Cd010443. Epub 2017/11/13. doi: 10.1002/14651858.CD010443.pub3. PubMed PMID: 29129039; PubMed Central PMCID: PMCPMC6485974.
3. Thangaratinam S, Rogozińska E, Jolly K, Glinkowski S, Duda W, Borowiack E, et al. Interventions to reduce or prevent obesity in pregnant women: a systematic review. 2012;16:31. doi: 10.3310/hta16310.
4. Thangaratinam S, Rogozińska E, Jolly K, Glinkowski S, Roseboom T, Tomlinson JW, et al. Effects of interventions in pregnancy on maternal weight and obstetric outcomes: meta-analysis of randomised evidence. BMJ : British Medical Journal. 2012;344:e2088. doi: 10.1136/bmj.e2088.
5. Tieu J, Shepherd E, Middleton P, Crowther CA. Dietary advice interventions in pregnancy for preventing gestational diabetes mellitus. The Cochrane database of systematic reviews. 2017;1:Cd006674. Epub 2017/01/04. doi: 10.1002/14651858.CD006674.pub3. PubMed PMID: 28046205; PubMed Central PMCID: PMCPMC6464792.
6. International Weight Management in Pregnancy (IWiP) collaboration. Effect of diet and physical activity based interventions in pregnancy on gestational weight gain and pregnancy outcomes: meta-analysis of individual participant data from randomised trials. BMJ (Clinical research ed). 2017;358:j3119. doi: 10.1136/bmj.j3119.
7. Davenport MH, McCurdy AP, Mottola MF, Skow RJ, Meah VL, Poitras VJ, et al. Impact of prenatal exercise on both prenatal and postnatal anxiety and depressive symptoms: a systematic review and meta-analysis. British Journal of Sports Medicine. 2018;52(21):1376. doi: 10.1136/bjsports-2018-099697.
8. Lau Y, Klainin-Yobas P, Htun TP, Wong SN, Tan KL, Ho-Lim ST, et al. Electronic-based lifestyle interventions in overweight or obese perinatal women: a systematic review and meta-analysis. Obesity reviews : an official journal of the International Association for the Study of Obesity. 2017;18(9):1071-87. Epub 2017/05/26. doi: 10.1111/obr.12557. PubMed PMID: 28544551.

S13 Tables: Meta-analysis for fetal growth-related outcomes reported by the included systematic reviews

Table S13a: Meta-analysis for birth weight reported by the included systematic reviews

Systematic review author, year	Included population or intervention subgroup	N studies	Sample size (pooled data)	Meta-analysis: birth weight	Statistical significance / direction
**Smoking**					
**Veisani *et al.* 2017 [1]**	**n/a**	**16**	**6,192**	**MD 0.28kg (95% CI 0.05, 0.50)**	**Significantly increased**
**Chamberlain *et al.* 2017 [2]**	**Counselling vs usual care**	**11**	**4,925**	**MD 40.27g (95% CI 7.87, 72.66)**	**Significantly increased p=0.015**
**Chamberlain *et al.* 2017 [2]**	**Incentives vs usual care**	**5**	**797**	**MD 109.38g (95% CI 60.51, 158.26)**	**Significantly increased p<0.0001**
**Chamberlain *et al.* 2017 [2]**	**Incentives vs alternative intervention**	**3**	**184**	**MD 134.58g (95% CI 76.32, 192.83)**	**Significantly increased p<0.0001**
Chamberlain *et al.* 2017 [2]	Counselling vs less intensive intervention	3	546	MD 56.02g (95% CI -31.46, 143.50)	Not significant
Chamberlain *et al.* 2017 [2]	Feedback vs usual care	2	3,006	MD 79.43g (95% CI -53.05, 211.91)	Not significant
**Diet and/or Physical Activity**					
Agha *et al.* 2014 [3]	Any BMI	9	1,381	MD 17.88g (95% CI -38.93, 74.69)	Not significant
Bain *et al.* 2015 [4]	Any BMI	5	737	MD 28.24g (95% CI -78.26, 134.74)	Not significant
Madhuvrata *et al.* 2015 [5]	Any BMI	4	701	MD 0.02kg (95% CI -0.15, 0.19)	Not significant p=0.82
Muktabhant *et al.* 2015 [6]	Any BMI	29	8,350	MD 12.20g (95% CI -15.26, 39.65)	Not significant
Rogozińska *et al.* 2015 [7]	Any BMI	6	1,088	SMD 0.04kg (95% CI -0.17, 0.24)	Not significant p=0.73
Magro-Malosso *et al* 2017a [8]	Any BMI	18	5,077	MD -57.23g (95% CI -117.45, 26.14)	Not significant
Shepherd *et al.* 2017 [9]	Any BMI	13	5,763	MD -17.67g (95% CI -46.28, 10.94)	Not significant
Tanentsapf *et al.* 2011 [10]	Recommended BMI, overweight, obese	7	1,048	WMD -34.8g (95% CI -162.6, 93)	Not significant
**Thangaratinam *et al.* 2012 [11]**	**Recommended BMI, overweight, obese**	**28**	**4,573**	**MD -0.07kg (95% CI -0.14, -0.01)**	**Significantly reduced p=0.03**
Thangaratinam *et al.* 2012b [12]	Recommended BMI, overweight, obese	31	5,278	MD -50g (95% CI -100, 0)	Not significant p=0.08
O'Brien *et al.* 2015 [13]	Recommended BMI	3	300	MD 21.6g (95% CI -98.8, 141.93)	Not significant
Oteng-Ntim *et al.* 2012 [14]	Overweight or obese	7	1,133	MD -56.64g (95% CI -120.15, 6.88)	Not significant p=0.08
Lau *et al.* 2017 [15]	Overweight or obese	5	1,968	MD -13.12g (95% CI -64.47, 38.23)	Not significant p=0.62
Magro-Malosso *et al* 2017b [16]	Overweight or obese	7	891	MD 16.91g (95% CI -89.33, 123.19)	Not significant
**Diet only**					
Madhuvrata *et al.* 2015 [5]	Any BMI	3	406	MD -0.03kg (95% CI -0.16, 0.09)	Not significant p=0.59
Rogozińska *et al.* 2015 [7]	Any BMI	5	1,219	SMD 0.06 (95% CI -0.13, 0.25)	Not significant p=0.53
Tieu *et al.* 2017 [17]	Any BMI	5	1,324	MD 5.94g (95% CI -51.11, 62.99)	Not significant p=0.84

Systematic review author, year	Included population or intervention subgroup	N studies	Sample size (pooled data)	Meta-analysis: birth weight	Statistical significance / direction
Zhang *et al.* 2018 [18]	Any BMI - Low GI	11	1,970	WMD −0.10kg (95% CI −0.23, 0.03)	Not significant
**Muktabhant *et al.* 2015 [6]**	**Any BMI - Low GI**	**4**	**1447**	**MD -0.84g (95% CI -1.16, -0.52)**	**Significantly reduced**
**Gresham *et al.* 2014 [19]**	**Any BMI**	**28**	**8,990**	**SMD 0.19 (95% CI 0.06, 0.31)**	**Significantly increased p<0.01**
Thangaratinam *et al.* 2012 [11]	Recommended BMI, overweight, obese	9	2,737	MD -0.07kg (95% CI -0.21, 0.07)	Not significant p=0.31
Thangaratinam *et al.* 2012b [12]	Recommended BMI, overweight, obese	10	2,861	MD −60g (95% CI −190, 80)	Not significant p=0.41
Quinlivan *et al.* 2011 [20]	Overweight or obese	4	537	WMD 8.49g (-84.88, 101.86)	Not significant p=0.40
**Physical activity only**					
Han *et al.* 2012 [21]	Any BMI	2	167	MD -102.87g (95% CI -235.34, 29.60)	Not significant p=0.13
da Silva *et al.* 2017 [22]	Any BMI	22	4,909	MD -31.09g (95% CI -69.91, 7.73)	Not significant
**Sanabria-Martínez *et al.* 2016 [23]**	**Any BMI**	**14**	**3,044**	**ES (t-score) -0.10 (95% CI -0.17, -0.13)**	**Significantly reduced p=0.004**
Zheng *et al.* 2017 [24]	Any BMI	5	1,691	SMD -0.10 (95% CI -0.25, 0.04)	Not significant p=0.16
Guillemette *et al.* 2018 [25]	Any BMI	38	6,766	MD -22.12g (95% CI -51.54, 7.29)	Not significant p=0.14
**Wiebe *et al.* 2015 [not yet linked]**	**Any BMI**	**27**	**5,214**	**WMD -31g (95% CI -57 to -4)**	**Significantly reduced p=0.002**
**Thangaratinam *et al.* 2012 [11]**	**Recommended BMI, overweight, obese**	**14**	**1,291**	**MD -0.09kg (95% CI -0.18, -0.00)**	**Significantly reduced p=0.05**
**Thangaratinam *et al.* 2012b [12]**	**Recommended BMI, overweight, obese**	**14**	**1,369**	**MD −60g (95% CI −120, −10)**	**Significantly reduced p=0.02**
Di Mascio *et al.* 2016 [26]	Recommended BMI	8	2,063	MD -10.46g (SD -47.1, 26.21)	Not significant
Ming *et al.* 2018 [27]	Recommended BMI	9	2,981	MD −18.70g (95% CI −52.49, 15.08)	Not significant p=0.28
Guillemette *et al.* 2018 [25]	Recommended or underweight BMI	4	831	MD -69.38g (95% CI -210.25, 71.49)	Not significant p=0.33
Chatzakis *et al*. 2019 [28]	Overweight or obese	10	Not reported	MD -0.05kg (95% CI -0.17, 0.07)	Not significant
Du *et al*. 2019 [29]	Overweight or obese	11	1,212	SMD -0.06 (95% CI -0.17, 0.05)	Not significant p=0.31
Guillemette *et al.* 2018 [25]	Overweight or obese	9	1,032	MD -46.54g (95% CI -107.10, 14.02)	Not significant p=0.13
Wiebe *et al.* 2015 [not yet linked]	Overweight or obese	3	462	WMD 9.76g (95% CI -85.73, 105.25)	Not significant p=0.84
Guillemette *et al.* 2018 [25]	Obese	2	106	MD -151.08g (95% CI -528.90, 226.73)	Not significant p=0.43

Footnote: BMI – body mass index, N – number, MD – mean difference, SMD – standardised mean difference, WMD – weighted mean difference, ES – effect size, CI – confidence interval, g – grams, kg – kilograms, low GI – low glycaemic index diet. Bold data highlights statistically significant results.

Table S13b: Meta-analysis for large for gestational age (LGA) reported by the included systematic reviews

Systematic review author, year	Included population or intervention subgroup	N studies	Sample size (pooled data)	Meta-analysis: Large for gestational age	Statistical significance / direction
**Diet and/or Physical Activity**					
Bain *et al.* 2015 [4]	Any BMI	6	2,950	RR 0.90 (95% CI 0.77, 1.05)	Not significant
Madhuvrata *et al.* 2015 [5]	Any BMI	2	699	OR 0.88 (95% CI 0.38, 2.02)	Not significant p=0.76
Muktabhant *et al.* 2015 [6]	Any BMI	18	4,525	RR 0.92 (95% CI 0.80, 1.05)	Not significant
Shepherd *et al.* 2017 [9]	Any BMI	11	5,353	RR 0.93 (95% CI 0.81, 1.07)	Not significant p=0.30
**Morison *et al.* 2018 [30]**	**Any BMI**	**5**	**3,880**	**OR 0.58 (95% CI 0.36, 0.94)**	**Significantly reduced p=0.03**
**Thangaratinam *et al.* 2012 [11]**	**Recommended BMI, overweight, obese**	**12**	**3,021**	**RR 0.73 (95% CI 0.54, 0.99)**	**Significantly reduced p=0.05**
Thangaratinam *et al.* 2012b [12]	Recommended BMI, overweight, obese	18	4,233	RR 0.85 (95% CI 0.66, 1.09)	Not significant p=0.21
IWiP 2017 [31]	Recommended BMI, overweight, obese	34	12,047	OR 0.90 (95% CI 0.76, 1.07) (IPD MA)	Not significant
IWiP 2017 [31]	Recommended BMI, overweight, obese	45	13,348	OR 0.86 (95% CI 0.71, 1.04) (IPD + non-IPD)	Not significant
Bain *et al.* 2015 [4]	Overweight or obese	3	2,616	RR 0.93 (95% CI 0.79, 1.09)	Not significant
Shepherd *et al.* 2017 [9]	Overweight or obese	4	2,385	RR 0.89 (95% CI 0.76, 1.06)	Not significant
Oteng-Ntim *et al.* 2012 [14]	Overweight or obese	6	1,008	OR 0.91 (95% CI 0.62, 1.32)	Not significant p=0.61
Shepherd *et al.* 2017 [9]	Obese	3	1,986	RR 1.17 (95% CI 0.89, 1.54)	Not significant
**Diet only**					
**Oostdam *et al.* 2011 [32]**	**Any BMI - low GI**	**3**	**127**	**RR 0.14 (95% CI 0.05, 0.41)**	**Significantly reduced p=0.0004**
**Zhang *et al.* 2018 [18]**	**Any BMI - Low GI**	**8**	**1,090**	**RR 0.52 (95% CI 0.31, 0.89)**	**Significantly reduced**
Muktabhant *et al.* 2015 [6]	Any BMI - Low GI	3	200	RR 1.25 (95% CI 0.50, 3.11)	Not significant
Gresham *et al.* 2014 [19]	Any BMI	3	992	SMD -0.21 (95% CI -0.60, 0.17)	Not significant p=0.28
Thangaratinam *et al.* 2012 [11]	Recommended BMI, overweight, obese	5	2,378	RR 0.78 (95% CI 0.51, 1.19)	Not significant p=0.26
Thangaratinam *et al.* 2012b [12]	Recommended BMI, overweight, obese	5	2,378	RR 0.78 (95% CI 0.51, 1.19)	Not significant p=0.26
IWiP 2017 [31]	Recommended BMI, overweight, obese	4	1,408	OR 0.91 (95% CI 0.60, 1.37) (IPD MA)	Not significant
IWiP 2017 [31]	Recommended BMI, overweight, obese	6	1,699	OR 0.82 (95% CI 0.54, 1.22) (IPD + non-IPD)	Not significant
**Physical activity only**					
**da Silva *et al.* 2017 [22]**	**Any BMI**	**3**	**603**	**RR 0.51 (95% CI 0.30, 0.87)**	**Significantly reduced**
Guillemette *et al.* 2018 [25]	Any BMI	7	937	RR 0.85 (95% CI 0.51, 1.44)	Not significant p=0.55
Thangaratinam *et al.* 2012 [11]	Recommended BMI, overweight, obese	2	183	RR 0.37 (95% CI 0.06, 2.30)	Not significant p=0.28

Thangaratinam *et al.* 2012b [12]	Recommended BMI, overweight, obese	4	355	RR 0.52 (95% CI 0.25, 1.09)	Not significant p=0.08
IWiP 2017 [31]	Recommended BMI, overweight, obese	15	3,330	OR 0.96 (95% CI 0.59, 1.54) (IPD MA)	Not significant
IWiP 2017 [31]	Recommended BMI, overweight, obese	21	3,930	OR 0.96 (95% CI 0.67, 1.37) (IPD + non-IPD)	Not significant
Chatzakis *et al*. 2019 [28]	Overweight or obese	7	971	RR 1.0 (95% CI 0.66, 1.49)	Not significant
Du *et al*. 2019 [29]	Overweight or obese	7	961	RR 0.90 (95% CI 0.65, 1.25)	Not significant p=0.52

Footnote: BMI – body mass index, N – number, RR – risk ratio, OR – odds ratio, CI – confidence interval, SMD – standardised mean difference, low GI – low glycaemic index diet, IPD – individual participant data, MA – meta-analysis. Bold data highlights statistically significant results.

Table S13c: Meta-analysis for macrosomia reported by the included systematic reviews

Systematic review author, year	Included population or intervention subgroup	N studies	Sample size (pooled data)	Meta-analysis: macrosomia (>4000g)	Statistical significance / direction
**Diet and/or Physical Activity**					
Bain *et al.* 2015 [4]	Any BMI	6	3,168	RR 0.90 (95% CI 0.77, 1.05)	Not significant
Madhuvrata *et al.* 2015 [5]	Any BMI	5	1,000	OR 0.99 (95% CI 0.72, 1.36)	Not significant p=0.94
Muktabhant *et al.* 2015 [6]	Any BMI	27	8,598	RR 0.93 (95% CI 0.86, 1.02)	Not significant
Shepherd *et al.* 2017 [9]	Any BMI	9	5,368	RR 0.89 (95% CI 0.78, 1.01)	Not significant
Tanentsapf *et al.* 2011 [10]	Recommended BMI, overweight, obese	6	1,023	RR 0.94 (95% CI 0.62, 1.35)	Not significant
O'Brien *et al.* 2015 [13]	Recommended BMI	2	243	RR 2.19 (95% CI 0.63, 7.60)	Not significant
Lau *et al.* 2017 [15]	Overweight or obese	2	238	RR 1.00 (95% CI 0.79, 1.26)	Not significant p=0.97
Magro-Malosso *et al* 2017b [16]	Overweight or obese	5	1,227	RR 0.92 (95% CI 0.72, 1.18)	Not significant
**Shepherd *et al.* 2017 [9]**	**Any BMI**	**4**	**3,061**	**RR 0.63 (95% CI 0.42, 0.94) (>4500g)**	**Significantly reduced**
**Diet only**					
Zhang *et al.* 2018 [18]	Any BMI - Low GI	8	Not reported	RR 0.95 (95% CI 0.83, 1.09)	Not significant
Muktabhant *et al.* 2015 [6]	Any BMI - Low GI	4	1,472	RR 0.96 (95% CI 0.84, 1.10)	Not significant
Oostdam *et al.* 2011 [32]	Any BMI (counselling)	5	383	RD -0.04 (95% CI -0.15, 0.06)	Not significant p=0.40
Gresham *et al.* 2014 [19]	Any BMI	4	1,224	SMD -0.04 (95% CI -0.27, 0.19)	Not significant p=0.75
**Physical activity only**					
**Oostdam *et al.* 2011 [32]**	**Any BMI**	**2**	**236**	**RD 0.36 (95% CI 0.13, 0.99)**	**Significantly reduced p=0.05**
Han *et al.* 2012 [21]	Any BMI	2	934	RR 0.91 (95% CI 0.68, 1.22)	Not significant p=0.52
Du *et al.* 2019 [29]	Overweight or obese	4	595	RR 0.91 (95% CI 0.61, 1.36)	Not significant p=0.64

Footnote: BMI – body mass index, N – number, RR – risk ratio, OR – odds ratio, CI – confidence interval, SMD – standardised mean difference, MD – mean difference, RD – risk difference, g – grams, low GI – low glycaemic index diet. Bold data highlights statistically significant results.

Table S13d: Meta-analysis for small for gestational age (SGA) reported by the included systematic reviews

Systematic review author, year	Included population or intervention subgroup	N studies	Sample size (pooled data)	Meta-analysis: small for gestational age	Statistical significance / direction
**Diet and/or Physical Activity**					
Bain *et al.* 2015 [4]	Any BMI	2	144	RR 1.02 (95% CI 0.18, 5.64)	Not significant
Madhuvrata *et al.* 2015 [5]	Any BMI	3	611	OR 1.33 (95% CI 0.60, 2.98)	Not significant p=0.48
Muktabhant *et al.* 2015 [6]	Any BMI	7	662	RR 1.09 (95%CI 0.61, 1.94)	Not significant
Shepherd *et al.* 2017 [9]	Any BMI	6	2,434	RR 1.20 (95% CI 0.95, 1.52)	Not significant
Thangaratinam *et al.* 2012 [11]	Recommended BMI, overweight, obese	8	2,901	RR 0.99 (95% CI 0.76, 1.29)	Not significant p=0.95
Thangaratinam *et al.* 2012b [12]	Recommended BMI, overweight, obese	11	3,552	RR 1.00 (95% CI 0.78, 1.28)	Not significant p=0.99
IWiP 2017 [31]	Recommended BMI, overweight, obese	33	11,666	OR 1.06 (95% CI 0.94, 1.20) (IPD MA)	Not significant
IWiP 2017 [31]	Recommended BMI, overweight, obese	44	12,937	OR 1.05 (95% CI 0.94 to 1.18) (IPD + non-IPD)	Not significant
**Diet only**					
Zhang *et al.* 2018 [18]	Any BMI - Low GI	6	Not reported	RR 1.33 (95% CI 0.71, 2.50)	Not significant
Muktabhant *et al.* 2015 [6]	Any BMI - Low GI	2	155	RR 1.49 (95% CI 0.47, 4.71)	Not significant
Gresham *et al.* 2014 [19]	Any BMI	5	2,617	SMD 0.12 (95% CI -0.06, 0.30)	Not significant p=0.20
Thangaratinam *et al.* 2012 [11]	Recommended BMI, overweight, obese	3	2,252	RR 1.02 (95% CI 0.75, 1.37)	Not significant p=0.91
Thangaratinam *et al.* 2012b [12]	Recommended BMI, overweight, obese	3	2,252	RR 1.02 (95% CI 0.75, 1.37)	Not significant p=0.91
IWiP 2017 [31]	Recommended BMI, overweight, obese	4	1,337	OR 0.92 (95% CI 0.45 to 1.88) (IPD MA)	Not significant
IWiP 2017 [31]	Recommended BMI, overweight, obese	6	1,628	OR 1.05 (95% CI 0.62 to 1.77) (IPD + non-IPD)	Not significant
**Physical activity only**					
da Silva *et al.* 2017 [22]	Any BMI	4	1,499	RR 1.08 (95% CI 0.66, 1.76)	Not significant
Thangaratinam *et al.* 2012 [11]	Recommended BMI, overweight, obese	3	304	RR 1.31 (95% CI 0.50, 3.42)	Not significant p=0.58
Thangaratinam *et al.* 2012b [12]	Recommended BMI, overweight, obese	4	409	RR 1.28 (95% CI 0.52, 3.15)	Not significant p=0.60
IWiP 2017 [31]	Recommended BMI, overweight, obese	14	3,272	OR 1.05 (95% CI 0.84 to 1.34) (IPD MA)	Not significant
IWiP 2017 [31]	Recommended BMI, overweight, obese	21	3,955	OR 1.01 (95% CI 0.83 to 1.24) (IPD + non-IPD)	Not significant
Du *et al.* 2019 [29]	Overweight or obese	6	863	RR 1.02 (95% CI 0.54, 1.91)	Not significant p=0.96

Footnote: BMI – body mass index, N – number, RR – risk ratio, OR – odds ratio, CI – confidence interval, SMD – standardised mean difference, low GI – low glycaemic index diet, IPD – individual participant data, MA – meta-analysis. Bold data highlights statistically significant results.

Table S13e: Meta-analysis for low birth weight (LBW) reported by the included systematic reviews

Systematic review author, year	Included population or intervention subgroup	N studies	Sample size (pooled data)	Meta-analysis: Low birth weight (<2500g)	Statistical significance / direction
**Smoking**					
**Veisani *et al.* 2017 [1]**	**n/a**	**8**	**2,890**	**OR 0.65 (95% CI 0.42, 0.88)**	**Significantly reduced**
Chamberlain *et al.* 2017 [2]	Counselling vs usual care	6	3,836	RR 0.87 (95% CI 0.70, 1.08)	Not significant
Chamberlain *et al.* 2017 [2]	Counselling vs less intensive intervention	2	503	RR 0.58 (95% CI 0.32, 1.04)	Not significant
Chamberlain *et al.* 2017 [2]	Incentives vs usual care	4	215	RR 0.70 (95% CI 0.40, 1.23)	Not significant
Chamberlain *et al.* 2017 [2]	Incentives vs alternative intervention	3	184	RR 0.60 (95% CI 0.26, 1.40)	Not significant
Chamberlain *et al.* 2017 [2]	Counselling vs usual care	2	1,666	RR 1.27 (95% CI 0.60, 2.71) (<1500g)	Not significant
**Diet and/or Physical Activity**					
Bain *et al.* 2015 [4]	Any BMI	2	459	RR 1.00 (95% CI 0.49, 2.05)	Not significant
Muktabhant *et al.* 2015 [6]	Any BMI	12	4,834	RR 0.88 (95% CI 0.67, 1.14)	Not significant
Tanentsapf *et al.* 2011 [10]	Recommended BMI, overweight, obese	2	531	RR 1.30 (95% CI 0.8, 2.10)	Not significant
O'Brien *et al.* 2015 [13]	Recommended BMI	2	243	RR 1.03 (95% CI 0.40, 2.63)	Not significant
Magro-Malosso *et al* 2017b [16]	Overweight or obese	5	686	RR 0.58 (95% CI 0.25, 1.34)	Not significant
**Diet only**					
**Gresham *et al.* 2014 [19]**	**Any BMI**	**10**	**5,448**	**SMD -0.19 (95% CI -0.32, -0.05)**	**Significantly reduced p<0.01**
**Physical activity only**					
Di Mascio *et al.* 2016 [26]	Recommended BMI	5	1,517	RR 1.11 (95% CI 0.72, 1.73)	Not significant

Footnote: BMI – body mass index, N – number, RR – risk ratio, OR – odds ratio, CI – confidence interval, SMD – standardised mean difference, g - grams. Bold data highlights statistically significant results.

Table S13f: Meta-analysis for other measures of fetal growth reported by the included systematic reviews

Systematic review author, year	Type of intervention	Included population or intervention subgroup	Outcome	N studies	Sample size (pooled data)	Meta-analysis: other measures of growth	Statistical significance/ direction
Thangaratinam *et al.* 2012 [11]	Diet and/or PA	Recommended BMI, overweight, obese	Ponderal index	4	323	MD -0.09 kg/m^3 (95% CI -0.18, 0.00)	Not significant p=0.06
Shepherd *et al.* 2017 [9]	Diet and/or PA	Any BMI	Ponderal index	3	2,826	MD 0.04kg/m^3 (95% CI -0.16, 0.25)	Not significant
Zhang *et al.* 2018 [18]	Diet only - Low GI	Any BMI	Ponderal index	8	Not reported	WMD -0.07kg/m^3 (95% CI -0.71, 0.57)	Not significant
Thangaratinam *et al.* 2012 [11]	Diet and/or PA	Recommended BMI, overweight, obese	Fetal fat mass	4	1,067	MD -0.08kg (95% CI -0.18, 0.03)	Not significant p=0.14
**Thangaratinam *et al.* 2012 [11]**	**Diet only**	**Recommended BMI, overweight, obese**	**Fetal fat mass**	**2**	**970**	**MD -0.04kg (95% CI -0.06, -0.01)**	**Significantly reduced p=0.005**
Thangaratinam *et al.* 2012 [11]	PA only	Recommended BMI, overweight, obese	Fetal fat mass	2	97	MD -0.09kg (-0.30, 0.13)	Not significant p=0.44
Guillemette *et al.* 2018 [25]	PA only	Any BMI	Infant fat mass percentage	2	130	RR 0.19% (95% CI -0.27, 0.65)	Not significant p=0.42
Shepherd *et al.* 2017 [9]	Diet and/or PA	Any BMI	Infant sum of skinfold thickness	2	1,472	MD 0.09mm (95% CI -0.33, 0.50)	Not significant
Thangaratinam *et al.* 2012 [11]	Diet and/or PA	Recommended BMI, overweight, obese	Infant abdominal circumference	2	62	MD -1.26cm (95% CI -3.71, 1.19)	Not significant p=0.31
Shepherd *et al.* 2017 [9]	Diet and/or PA	Any BMI	Infant abdominal circumference	2	1,566	MD -0.01cm (95% CI -0.23, 0.22)	Not significant
Zhang *et al.* 2018 [18]	Diet only - Low GI	Any BMI	Infant abdominal circumference	3	Not reported	WMD -0.65cm (95% CI -2.23, 0.92)	Not significant
Thangaratinam *et al.* 2012 [11]	Diet and/or PA	Recommended BMI, overweight, obese	Infant length (units not specified)	5	323	MD -0.80 (95% CI -1.81, 0.21)	Not significant p=0.12
**Thangaratinam *et al.* 2012 [11]**	**Diet only**	**Recommended BMI, overweight, obese**	**Infant length (units not specified)**	**2**	**62**	**MD -1.84 (95% CI -3.61, -0.08)**	**Significantly reduced p=0.04**
Thangaratinam *et al.* 2012 [11]	Diet and/or PA	Recommended BMI, overweight, obese	Infant crown-heel length	3	181	MD -0.18cm (95% CI -1.80, 1.44)	Not significant p=0.83
Shepherd *et al.* 2017 [9]	Diet and/or PA	Any BMI	Infant length	6	3,303	MD -0.09cm (95% CI -0.26, 0.09)	Not significant
Tieu *et al.* 2017 [17]	Diet only	Any BMI	Infant length	3	968	MD -0.16cm (95% CI -0.28, 0.60)	Not significant
Zhang *et al.* 2018 [18]	Diet only - Low GI	Any BMI	Infant length	6	Not reported	WMD -0.05cm (95% CI -0.66, 0.55)	Not significant
**Gresham *et al.* 2014 [19]**	**Diet only**	**Any BMI**	**Infant length**	**14**	**5,195**	**SMD 0.08 cm (95% CI 0.01, 0.15)**	**Significantly increased p=0.03**
Shepherd *et al.* 2017 [9]	Diet and/or PA	Any BMI	Infant head circumference	4	4,229	MD -0.01cm (95% CI -0.11, 0.10)	Not significant
Tieu *et al.* 2017 [17]	Diet only	Any BMI	Infant head circumference	3	968	MD -0.21cm (95% CI -0.67, 0.25)	Not significant
Zhang *et al.* 2018 [18]	Diet only - Low GI	Any BMI	Infant head circumference	5	Not reported	WMD -0.13cm (95% CI -0.68, 0.41)	Not significant
Gresham *et al.* 2014 [19]	Diet only	Any BMI	Infant head circumference	10	2,785	SMD 0.00cm (95% CI -0.19, 0.19)	Not significant p=1.0
**Wiebe *et al.* 2015 [33]**	**PA only**	**Any BMI**	**Large at birth (either macrosomia or LGA)**	**21**	**3,982**	**OR 0.69 (95% CI 0.55, 0.86)**	**Significantly reduced p=0.0009**
Wiebe *et al.* 2015 [33]	PA only	Overweight or obese	Large at birth (either macrosomia or LGA)	3	438	OR 0.71 (95% CI 0.36, 1.41)	Not significant p=0.33

Wiebe *et al.* 2015 [33]	PA only	Any BMI	Small at birth (either low birth weight or SGA)	12	2,183	OR 1.02 (95% CI 0.72, 1.46)	Not significant p=0.75
Wiebe *et al.* 2015 [33]	PA only	Overweight or obese	Small at birth (either low birth weight or SGA)	2	341	OR 0.90 (95% CI 0.31, 2.63)	Not significant p=0.85

Footnote: BMI – body mass index, N – number, RR – risk ratio, CI – confidence interval, SMD – standardised mean difference, WMD – weighted mean difference, MD – mean difference, , low GI – low glycaemic index diet, kg – kilograms, cm – centimetre, m – metre, mm - millimetre. Bold data highlights statistically significant results.

S13 References

1. Veisani Y, Jenabi E, Delpisheh A, Khazaei S. Effect of prenatal smoking cessation interventions on birth weight: meta-analysis. The journal of maternal-fetal & neonatal medicine : the official journal of the European Association of Perinatal Medicine, the Federation of Asia and Oceania Perinatal Societies, the International Society of Perinatal Obstet. 2019;32(2):332-8. Epub 2017/09/12. doi: 10.1080/14767058.2017.1378335. PubMed PMID: 28889768.
2. Chamberlain C, O'Mara-Eves A, Porter J, Coleman T, Perlen SM, Thomas J, et al. Psychosocial interventions for supporting women to stop smoking in pregnancy. The Cochrane database of systematic reviews. 2017;2:Cd001055. Epub 2017/02/15. doi: 10.1002/14651858.CD001055.pub5. PubMed PMID: 28196405; PubMed Central PMCID: PMCPMC6472671.
3. Agha M, Agha RA, Sandall J. Interventions to reduce and prevent obesity in pre-conceptual and pregnant women: a systematic review and meta-analysis. PloS one. 2014;9(5):e95132-e. doi: 10.1371/journal.pone.0095132. PubMed PMID: 24827704.
4. Bain E, Crane M, Tieu J, Han S, Crowther CA, Middleton P. Diet and exercise interventions for preventing gestational diabetes mellitus. The Cochrane database of systematic reviews. 2015;(4):Cd010443. Epub 2015/04/13. doi: 10.1002/14651858.CD010443.pub2. PubMed PMID: 25864059.
5. Madhuvrata P, Govinden G, Bustani R, Song S, Farrell TA. Prevention of gestational diabetes in pregnant women with risk factors for gestational diabetes: a systematic review and meta-analysis of randomised trials. Obstet Med. 2015;8(2):68-85. Epub 2015/06/01. doi: 10.1177/1753495x15576673. PubMed PMID: 27512459; PubMed Central PMCID: PMCPMC4935009.
6. Muktabhant B, Lawrie TA, Lumbiganon P, Laopaiboon M. Diet or exercise, or both, for preventing excessive weight gain in pregnancy. The Cochrane database of systematic reviews. 2015;(6):Cd007145. Epub 2015/06/13. doi: 10.1002/14651858.CD007145.pub3. PubMed PMID: 26068707.
7. Rogozińska E, Chamillard M, Hitman GA, Khan KS, Thangaratinam S. Nutritional manipulation for the primary prevention of gestational diabetes mellitus: a meta-analysis of randomised studies. PloS one. 2015;10(2):e0115526. Epub 2015/02/27. doi: 10.1371/journal.pone.0115526. PubMed PMID: 25719363; PubMed Central PMCID: PMCPMC4342242.
8. Magro-Malosso ER, Saccone G, Di Tommaso M, Roman A, Berghella V. Exercise during pregnancy and risk of gestational hypertensive disorders: a systematic review and meta-analysis. Acta Obstetricia et Gynecologica Scandinavica. 2017;96(8):921-31. doi: 10.1111/aogs.13151.
9. Shepherd E, Gomersall JC, Tieu J, Han S, Crowther CA, Middleton P. Combined diet and exercise interventions for preventing gestational diabetes mellitus. The Cochrane database of systematic reviews. 2017;11:Cd010443. Epub 2017/11/13. doi: 10.1002/14651858.CD010443.pub3. PubMed PMID: 29129039; PubMed Central PMCID: PMCPMC6485974.

10. Tanentsapf I, Heitmann BL, Adegboye ARA. Systematic review of clinical trials on dietary interventions to prevent excessive weight gain during pregnancy among normal weight, overweight and obese women. BMC Pregnancy and Childbirth. 2011;11(1):81. doi: 10.1186/1471-2393-11-81.
11. Thangaratinam S, Rogozińska E, Jolly K, Glinkowski S, Duda W, Borowiack E, et al. Interventions to reduce or prevent obesity in pregnant women: a systematic review. 2012;16:31. doi: 10.3310/hta16310.
12. Thangaratinam S, Rogozińska E, Jolly K, Glinkowski S, Roseboom T, Tomlinson JW, et al. Effects of interventions in pregnancy on maternal weight and obstetric outcomes: meta-analysis of randomised evidence. BMJ : British Medical Journal. 2012;344:e2088. doi: 10.1136/bmj.e2088.
13. O'Brien CM, Grivell RM, Dodd JM. Systematic review of antenatal dietary and lifestyle interventions in women with a normal body mass index. Acta Obstet Gynecol Scand. 2016;95(3):259-69. Epub 2015/11/27. doi: 10.1111/aogs.12829. PubMed PMID: 26610169.
14. Oteng-Ntim E, Varma R, Croker H, Poston L, Doyle P. Lifestyle interventions for overweight and obese pregnant women to improve pregnancy outcome: systematic review and meta-analysis. BMC Medicine. 2012;10(1):47. doi: 10.1186/1741-7015-10-47.
15. Lau Y, Klainin-Yobas P, Htun TP, Wong SN, Tan KL, Ho-Lim ST, et al. Electronic-based lifestyle interventions in overweight or obese perinatal women: a systematic review and meta-analysis. Obesity reviews : an official journal of the International Association for the Study of Obesity. 2017;18(9):1071-87. Epub 2017/05/26. doi: 10.1111/obr.12557. PubMed PMID: 28544551.
16. Magro-Malosso ER, Saccone G, Di Mascio D, Di Tommaso M, Berghella V. Exercise during pregnancy and risk of preterm birth in overweight and obese women: a systematic review and meta-analysis of randomized controlled trials. Acta Obstet Gynecol Scand. 2017;96(3):263-73. Epub 2016/12/29. doi: 10.1111/aogs.13087. PubMed PMID: 28029178.
17. Tieu J, Shepherd E, Middleton P, Crowther CA. Dietary advice interventions in pregnancy for preventing gestational diabetes mellitus. The Cochrane database of systematic reviews. 2017;1:Cd006674. Epub 2017/01/04. doi: 10.1002/14651858.CD006674.pub3. PubMed PMID: 28046205; PubMed Central PMCID: PMCPMC6464792.
18. Zhang R, Han S, Chen GC, Li ZN, Silva-Zolezzi I, Parés GV, et al. Effects of low-glycemic-index diets in pregnancy on maternal and newborn outcomes in pregnant women: a meta-analysis of randomized controlled trials. Eur J Nutr. 2018;57(1):167-77. Epub 2016/09/11. doi: 10.1007/s00394-016-1306-x. PubMed PMID: 27612876.
19. Gresham E, Byles JE, Bisquera A, Hure AJ. Effects of dietary interventions on neonatal and infant outcomes: a systematic review and meta-analysis. Am J Clin Nutr. 2014;100(5):1298-321. Epub 2014/10/22. doi: 10.3945/ajcn.113.080655. PubMed PMID: 25332328.
20. Quinlivan JA, Julania S, Lam L. Antenatal dietary interventions in obese pregnant women to restrict gestational weight gain to Institute of Medicine recommendations: a meta-analysis. Obstet Gynecol. 2011;118(6):1395-401. Epub 2011/11/23. doi: 10.1097/AOG.0b013e3182396bc6. PubMed PMID: 22105270.
21. Han S, Middleton P, Crowther CA. Exercise for pregnant women for preventing gestational diabetes mellitus. The Cochrane database of systematic reviews. 2012;(7):Cd009021. Epub 2012/07/13. doi: 10.1002/14651858.CD009021.pub2. PubMed PMID: 22786521.
22. da Silva SG, Ricardo LI, Evenson KR, Hallal PC. Leisure-Time Physical Activity in Pregnancy and Maternal-Child Health: A Systematic Review and Meta-Analysis of Randomized Controlled Trials and Cohort Studies. Sports Med. 2017;47(2):295-317. Epub 2016/06/11. doi: 10.1007/s40279-016-0565-2. PubMed PMID: 27282925.
23. Sanabria-Martínez G, García-Hermoso A, Poyatos-León R, González-García A, Sánchez-López M, Martínez-Vizcaíno V. Effects of Exercise-Based Interventions on Neonatal Outcomes: A Meta-Analysis of Randomized Controlled Trials. Am J Health Promot. 2016;30(4):214-23. Epub 2016/07/13. doi: 10.1177/0890117116639569. PubMed PMID: 27404056.
24. Zheng J, Wang H, Ren M. Influence of exercise intervention on gestational diabetes mellitus: a systematic review and meta-analysis. J Endocrinol Invest. 2017;40(10):1027-33. Epub 2017/04/13. doi: 10.1007/s40618-017-0673-3. PubMed PMID: 28401529.

25. Guillemette L, Hay JL, Kehler DS, Hamm NC, Oldfield C, McGavock JM, et al. Exercise in Pregnancy and Children's Cardiometabolic Risk Factors: a Systematic Review and Meta-Analysis. Sports Med Open. 2018;4(1):35-. doi: 10.1186/s40798-018-0148-x. PubMed PMID: 30069801.
26. Di Mascio D, Magro-Malosso ER, Saccone G, Marhefka GD, Berghella V. Exercise during pregnancy in normal-weight women and risk of preterm birth: a systematic review and meta-analysis of randomized controlled trials. American journal of obstetrics and gynecology. 2016;215(5):561-71. Epub 2016/10/30. doi: 10.1016/j.ajog.2016.06.014. PubMed PMID: 27319364.
27. Ming W-K, Ding W, Zhang CJP, Zhong L, Long Y, Li Z, et al. The effect of exercise during pregnancy on gestational diabetes mellitus in normal-weight women: a systematic review and meta-analysis. BMC Pregnancy and Childbirth. 2018;18(1):440. doi: 10.1186/s12884-018-2068-7.
28. Chatzakis C, Goulis DG, Mareti E, Eleftheriades M, Zavlanos A, Dinas K, et al. Prevention of gestational diabetes mellitus in overweight or obese pregnant women: A network meta-analysis. Diabetes Res Clin Pract. 2019;158:107924. Epub 2019/11/19. doi: 10.1016/j.diabres.2019.107924. PubMed PMID: 31738997.
29. Du MC, Ouyang YQ, Nie XF, Huang Y, Redding SR. Effects of physical exercise during pregnancy on maternal and infant outcomes in overweight and obese pregnant women: A meta-analysis. Birth. 2019;46(2):211-21. Epub 2018/09/22. doi: 10.1111/birt.12396. PubMed PMID: 30240042.
30. Morison PN, Bacardi-Gascon M, Lopez-Corrales M, Jimenez-Cruz A. Combined dietary-exercise intervention for gestational weight gain and birthweight: a meta-analysis. Asia Pac J Clin Nutr. 2018;27(4):860-8. Epub 2018/07/27. doi: 10.6133/apjcn.112017.02. PubMed PMID: 30045432.
31. International Weight Management in Pregnancy (IWiP) collaboration. Effect of diet and physical activity based interventions in pregnancy on gestational weight gain and pregnancy outcomes: meta-analysis of individual participant data from randomised trials. BMJ (Clinical research ed). 2017;358:j3119. doi: 10.1136/bmj.j3119.
32. Oostdam N, van Poppel MN, Wouters MG, van Mechelen W. Interventions for preventing gestational diabetes mellitus: a systematic review and meta-analysis. Journal of women's health (2002). 2011;20(10):1551-63. Epub 2011/08/16. doi: 10.1089/jwh.2010.2703. PubMed PMID: 21838525.
33. Wiebe HW, Boulé NG, Chari R, Davenport MH. The Effect of Supervised Prenatal Exercise on Fetal Growth: A Meta-analysis. Obstetrics & Gynecology. 2015;125(5):1185-94. doi: 10.1097/aog.0000000000000801. PubMed PMID: 00006250-201505000-00028.

S14 Tables: Meta-analysis for gestational age at delivery-related outcomes reported by the included systematic reviews

Table S14a: Meta-analysis for gestational age at delivery reported by the included systematic reviews

Systematic review author, year	Included population or intervention subgroup	N studies	Sample size pooled data	Meta-analysis: Gestational age at delivery	Statistical significance / direction
**Diet and/or Physical Activity**					
Agha *et al.* 2014 [1]	Any BMI	8	1,146	MD 0.19 weeks (95% CI -0.03, 0.41)	Not significant
Bain *et al.* 2015 [2]	Any BMI	3	632	MD 0.13 weeks (95% CI -0.24, 0.50)	Not significant
Shepherd *et al.* 2017 [3]	Any BMI	11	5,658	MD 0.05 weeks (95% CI -0.05, 0.15)	Not significant
Magro-Malosso *et al* 2017a [4]	Any BMI	16	4,933	MD 0.03 weeks (95% CI -0.06, 0.13)	Not significant
**Tanentsapf *et al.* 2011 [5]**	**Recommended BMI, overweight, obese**	**7**	**1,167**	**WMD 0.22 weeks (95% CI 0.01, 0.42)**	**Significantly increased**
Thangaratinam *et al.* 2012 [6]	Recommended BMI, overweight, obese	20	4,028	MD -0.03 weeks (95% CI -0.13, 0.07)	Not significant p=0.53
Thangaratinam *et al.* 2012b [7]	Recommended BMI, overweight, obese	23	4,688	MD 0.02 weeks (95% CI -0.08, 0.11)	Not significant p=0.72
Lau *et al.* 2017 [8]	Overweight or obese	5	1,968	MD 0.01 week (95% CI -0.18, 0.20)	Not significant p=0.90
**Diet only**					
Zhang *et al.* 2018 [9]	Any BMI - Low GI	5	Not reported	WMD 0.03 weeks (95% CI −0.14, 0.20)	Not significant
Gresham *et al.* 2016 [10]	Any BMI	12	3,103	SMD 0.06 weeks (95% CI −0.05, 0.16)	Not significant p=0.29
Tieu *et al.* 2017 [11]	Any BMI	4	1,195	MD 0.05 weeks (95% CI -0.31, 0.40)	Not significant
Thangaratinam *et al.* 2012 [6]	Recommended BMI, overweight, obese	6	2,625	MD -0.05 weeks (95% CI -0.18, 0.08)	Not significant p=0.42
Thangaratinam *et al.* 2012b [7]	Recommended BMI, overweight, obese	6	813	MD 0.20 weeks (95% CI −0.02, 0.42)	Not significant p=0.07
**Physical activity only**					
Han *et al.* 2012 [12]	Any BMI	2	167	MD -0.04 weeks (95% CI -0.37, 0.29)	Not significant p=0.81
da Silva *et al.* 2017 [13]	Any BMI	17	4,278	MD -0.07 weeks (95% CI -0.29, 0.16)	Not significant
Sanabria-Martínez *et al.* 2016 [14]	Any BMI	13	2,982	ES (t-score) -0.07 (95% CI -0.19, 0.06)	Not significant p=0.284
Zheng *et al.* 2017 [15]	Any BMI	5	1,691	SMD -0.03 (95% CI -0.12, 0.07)	Not significant p=0.60
Wiebe *et al.* 2015 [16]	Any BMI	24	5,033	WMD 0.00 (95% CI -0.09, 0.09)	Not significant p=0.99
Thangaratinam *et al.* 2012 [6]	Recommended BMI, overweight, obese	10	1,129	MD -0.04 weeks (95% CI -0.22, 0.14)	Not significant p=0.64
Thangaratinam *et al.* 2012b [7]	Recommended BMI, overweight, obese	11	1250	MD 0.03 weeks (95% CI −0.14, 0.20)	Not significant p=0.74
**Di Mascio *et al.* 2016 [17]**	**Recommended BMI**	**7**	**2,008**	**MD 0.05 week (95% CI 0.07, 0.17)**	**Significantly increased**
Ming *et al.* 2018 [18]	Recommended BMI	9	2,981	MD −0.55 days (95% CI −1.57, 0.47)	Not significant p=0.29

Systematic review author, year	Included population or intervention subgroup	N studies	Sample size pooled data	Meta-analysis: Gestational age at delivery	Statistical significance / direction
Wiebe *et al.* 2015 [16]	Overweight or obese	3	462	WMD 0.07 (95% CI -0.24, 0.38)	Not significant p=0.67

Footnote: BMI – body mass index, N – number, MD – mean difference, SMD – standardised mean difference, WMD – weighted mean difference, ES – effect size, CI – confidence interval, low GI – low glycaemic index diet. Bold data highlights statistically significant results.

Table S14b: Meta-analysis for preterm delivery reported by the included systematic reviews

Systematic review author, year	Included population or intervention subgroup	N studies	Sample size pooled data	Meta-analysis: preterm delivery	Statistical significance / direction
**Smoking**					
Chamberlain *et al.* 2017 [19]	Counselling vs usual care	5	2,653	RR 0.90 (95% CI 0.64, 1.27)	Not significant
Chamberlain *et al.* 2017 [19]	Counselling vs less intensive intervention	3	794	RR 0.82 (95% CI 0.47, 1.42)	Not significant
Chamberlain *et al.* 2017 [19]	Feedback vs usual care	2	3,111	RR 0.60 (95% CI 0.28, 1.29)	Not significant
Chamberlain *et al.* 2017 [19]	Incentives vs usual care	5	753	RR 1.15 (95% CI 0.73, 1.82)	Not significant
Chamberlain *et al.* 2017 [19]	Incentives vs alternative intervention	3	184	RR 0.48 (95% CI 0.20, 1.14)	Not significant
**Diet and/or Physical Activity**					
**Bain *et al.* 2015 [2]**	**Any BMI**	**5**	**2,713**	**RR 0.71 (95% CI 0.55, 0.93)**	**Significantly reduced**
Madhuvrata *et al.* 2015 [20]	Any BMI	2	216	OR 1.20 (95% CI 0.45, 3.15)	Not significant p=0.72
Muktabhant *et al.* 2015 [21]	Any BMI	16	5,923	RR 0.91 (95% CI 0.68, 1.22)	Not significant
Rogozińska *et al.* 2015 [22]	Any BMI	8	3,697	RR 0.84 (95% CI 0.55, 1.27)	Not significant p=0.40
**Shepherd *et al.* 2017 [3]**	**Any BMI**	**11**	**5,398**	**RR 0.80 (95% CI 0.65, 0.98)**	**Significantly reduced**
Tanentsapf *et al.* 2011 [5]	Recommended BMI, overweight, obese	4	873	RR 0.83 (95% CI 0.51, 1.34)	Not significant
Thangaratinam *et al.* 2012 [6]	Recommended BMI, overweight, obese	12	2,198	RR 0.76 (95% CI 0.56, 1.02)	Not significant p=0.07
Thangaratinam *et al.* 2012b [7]	Recommended BMI, overweight, obese	13	2,652	RR 0.78 (95% CI 0.60, 1.02)	Not significant p=0.07
IWiP 2017 [23]	Recommended BMI, overweight, obese	32	11,676	OR 0.94 (95% CI 0.78, 1.13) (IPD MA)	Not significant
IWiP 2017 [23]	Recommended BMI, overweight, obese	49	14,339	OR 0.92 (95% CI 0.79, 1.08) (IPD + non-IPD)	Not significant
**Magro-Malosso *et al* 2017b [24]**	**Overweight or obese**	**9**	**1,502**	**RR 0.62 (95% CI 0.41, 0.95)**	**Significantly reduced p=0.03**
**Diet only**					
Zhang *et al.* 2018 [9]	Any BMI - Low GI	5	Not reported	RR 0.70 (95% CI 0.39, 1.28)	Not significant
Muktabhant *et al.* 2015 [21]	Any BMI - Low GI	2	804	RR 0.33 (95% CI 0.11, 1.02)	Not significant
Gresham *et al.* 2016 [10]	Any BMI	7	1,759	SMD −0.25 (95% CI −0.56, 0.05)	Not significant p=0.10
Rogozińska *et al.* 2015 [22]	Any BMI	2	1,057	RR 0.49 (95% CI 0.19, 1.29)	Not significant p=0.15
Tieu *et al.* 2017 [11]	Any BMI	3	1,149	RR 0.51 (95% CI 0.21, 1.25)	Not significant p=0.14
**Thangaratinam *et al.* 2012 [6, 7]**	**Recommended BMI, overweight, obese**	**4**	**1,474**	**RR 0.68 (95% CI 0.48, 0.96)**	**Significantly reduced P=0.03**
**IWiP 2017 [23]**	**Recommended BMI, overweight, obese**	**4**	**1,344**	**OR 0.28 (95% CI 0.08, 0.96) (IPD MA)**	**Significantly reduced**
**IWiP 2017 [23]**	**Recommended BMI, overweight, obese**	**7**	**1,696**	**OR 0.32 (95% CI 0.14, 0.70) (IPD + non-IPD)**	**Significantly reduced**

Systematic review author, year	Included population or intervention subgroup	N studies	Sample size pooled data	Meta-analysis: preterm delivery	Statistical significance / direction
**Physical activity only**					
Zheng *et al.* 2017 [15]	Any BMI	2	408	OR 0.93 (95% CI 0.44, 1.99)	Not significant p=0.86
Thangaratinam *et al.* 2012 [6]	Recommended BMI, overweight, obese	4	345	RR 1.12 (95% CI 0.44, 2.85)	Not significant p=0.82
Thangaratinam *et al.* 2012b [7]	Recommended BMI, overweight, obese	5	450	RR 1.22 (95% CI 0.51, 2.90)	Not significant p=0.65
IWiP 2017 [23]	Recommended BMI, overweight, obese	13	3,249	OR 1.29 (95% CI 0.90, 1.85) (IPD MA)	Not significant
IWiP 2017 [23]	Recommended BMI, overweight, obese	23	5,149	OR 1.09 (95% CI 0.84, 1.41) (IPD + non-IPD)	Not significant
Di Mascio *et al.* 2016 [17]	Recommended BMI	9	2,059	RR 1.01 (95% CI 0.68, 1.50)	Not significant p=0.97
Chatzakis *et al*. 2019 [25]	Overweight or obese	6	773	RR 1.11 (95% CI 0.57, 2.19)	Not significant
Du *et al*. 2019 [26]	Overweight or obese	6	737	RR 1.18 (95% CI 0.59, 2.39)	Not significant p=0.54

Footnote: BMI – body mass index, N – number, RR – risk ratio, OR – odds ratio, CI – confidence interval, SMD – standardised mean difference, low GI – low glycaemic index diet, IPD – individual participant data, MA – meta-analysis. Bold data highlights statistically significant results.

S14 References

1. Agha M, Agha RA, Sandall J. Interventions to reduce and prevent obesity in pre-conceptual and pregnant women: a systematic review and meta-analysis. PloS one. 2014;9(5):e95132-e. doi: 10.1371/journal.pone.0095132. PubMed PMID: 24827704.
2. Bain E, Crane M, Tieu J, Han S, Crowther CA, Middleton P. Diet and exercise interventions for preventing gestational diabetes mellitus. The Cochrane database of systematic reviews. 2015;(4):Cd010443. Epub 2015/04/13. doi: 10.1002/14651858.CD010443.pub2. PubMed PMID: 25864059.
3. Shepherd E, Gomersall JC, Tieu J, Han S, Crowther CA, Middleton P. Combined diet and exercise interventions for preventing gestational diabetes mellitus. The Cochrane database of systematic reviews. 2017;11:Cd010443. Epub 2017/11/13. doi: 10.1002/14651858.CD010443.pub3. PubMed PMID: 29129039; PubMed Central PMCID: PMCPMC6485974.
4. Magro-Malosso ER, Saccone G, Di Tommaso M, Roman A, Berghella V. Exercise during pregnancy and risk of gestational hypertensive disorders: a systematic review and meta-analysis. Acta Obstetricia et Gynecologica Scandinavica. 2017;96(8):921-31. doi: 10.1111/aogs.13151.
5. Tanentsapf I, Heitmann BL, Adegboye ARA. Systematic review of clinical trials on dietary interventions to prevent excessive weight gain during pregnancy among normal weight, overweight and obese women. BMC Pregnancy and Childbirth. 2011;11(1):81. doi: 10.1186/1471-2393-11-81.
6. Thangaratinam S, Rogozińska E, Jolly K, Glinkowski S, Duda W, Borowiack E, et al. Interventions to reduce or prevent obesity in pregnant women: a systematic review. 2012;16:31. doi: 10.3310/hta16310.
7. Thangaratinam S, Rogozińska E, Jolly K, Glinkowski S, Roseboom T, Tomlinson JW, et al. Effects of interventions in pregnancy on maternal weight and obstetric outcomes: meta-analysis of randomised evidence. BMJ : British Medical Journal. 2012;344:e2088. doi: 10.1136/bmj.e2088.

8. Lau Y, Klainin-Yobas P, Htun TP, Wong SN, Tan KL, Ho-Lim ST, et al. Electronic-based lifestyle interventions in overweight or obese perinatal women: a systematic review and meta-analysis. Obesity reviews : an official journal of the International Association for the Study of Obesity. 2017;18(9):1071-87. Epub 2017/05/26. doi: 10.1111/obr.12557. PubMed PMID: 28544551.
9. Zhang R, Han S, Chen GC, Li ZN, Silva-Zolezzi I, Parés GV, et al. Effects of low-glycemic-index diets in pregnancy on maternal and newborn outcomes in pregnant women: a meta-analysis of randomized controlled trials. Eur J Nutr. 2018;57(1):167-77. Epub 2016/09/11. doi: 10.1007/s00394-016-1306-x. PubMed PMID: 27612876.
10. Gresham E, Bisquera A, Byles JE, Hure AJ. Effects of dietary interventions on pregnancy outcomes: a systematic review and meta-analysis. Matern Child Nutr. 2016;12(1):5-23. Epub 2014/07/23. doi: 10.1111/mcn.12142. PubMed PMID: 25048387; PubMed Central PMCID: PMCPMC6860081.
11. Tieu J, Shepherd E, Middleton P, Crowther CA. Dietary advice interventions in pregnancy for preventing gestational diabetes mellitus. The Cochrane database of systematic reviews. 2017;1:Cd006674. Epub 2017/01/04. doi: 10.1002/14651858.CD006674.pub3. PubMed PMID: 28046205; PubMed Central PMCID: PMCPMC6464792.
12. Han S, Middleton P, Crowther CA. Exercise for pregnant women for preventing gestational diabetes mellitus. The Cochrane database of systematic reviews. 2012;(7):Cd009021. Epub 2012/07/13. doi: 10.1002/14651858.CD009021.pub2. PubMed PMID: 22786521.
13. da Silva SG, Ricardo LI, Evenson KR, Hallal PC. Leisure-Time Physical Activity in Pregnancy and Maternal-Child Health: A Systematic Review and Meta-Analysis of Randomized Controlled Trials and Cohort Studies. Sports Med. 2017;47(2):295-317. Epub 2016/06/11. doi: 10.1007/s40279-016-0565-2. PubMed PMID: 27282925.
14. Sanabria-Martínez G, García-Hermoso A, Poyatos-León R, González-García A, Sánchez-López M, Martínez-Vizcaíno V. Effects of Exercise-Based Interventions on Neonatal Outcomes: A Meta-Analysis of Randomized Controlled Trials. Am J Health Promot. 2016;30(4):214-23. Epub 2016/07/13. doi: 10.1177/0890117116639569. PubMed PMID: 27404056.
15. Zheng J, Wang H, Ren M. Influence of exercise intervention on gestational diabetes mellitus: a systematic review and meta-analysis. J Endocrinol Invest. 2017;40(10):1027-33. Epub 2017/04/13. doi: 10.1007/s40618-017-0673-3. PubMed PMID: 28401529.
16. Wiebe HW, Boulé NG, Chari R, Davenport MH. The Effect of Supervised Prenatal Exercise on Fetal Growth: A Meta-analysis. Obstetrics & Gynecology. 2015;125(5):1185-94. doi: 10.1097/aog.0000000000000801. PubMed PMID: 00006250-201505000-00028.
17. Di Mascio D, Magro-Malosso ER, Saccone G, Marhefka GD, Berghella V. Exercise during pregnancy in normal-weight women and risk of preterm birth: a systematic review and meta-analysis of randomized controlled trials. American journal of obstetrics and gynecology. 2016;215(5):561-71. Epub 2016/10/30. doi: 10.1016/j.ajog.2016.06.014. PubMed PMID: 27319364.
18. Ming W-K, Ding W, Zhang CJP, Zhong L, Long Y, Li Z, et al. The effect of exercise during pregnancy on gestational diabetes mellitus in normal-weight women: a systematic review and meta-analysis. BMC Pregnancy and Childbirth. 2018;18(1):440. doi: 10.1186/s12884-018-2068-7.
19. Chamberlain C, O'Mara-Eves A, Porter J, Coleman T, Perlen SM, Thomas J, et al. Psychosocial interventions for supporting women to stop smoking in pregnancy. The Cochrane database of systematic reviews. 2017;2:Cd001055. Epub 2017/02/15. doi: 10.1002/14651858.CD001055.pub5. PubMed PMID: 28196405; PubMed Central PMCID: PMCPMC6472671.
20. Madhuvrata P, Govinden G, Bustani R, Song S, Farrell TA. Prevention of gestational diabetes in pregnant women with risk factors for gestational diabetes: a systematic review and meta-analysis of randomised trials. Obstet Med. 2015;8(2):68-85. Epub 2015/06/01. doi: 10.1177/1753495x15576673. PubMed PMID: 27512459; PubMed Central PMCID: PMCPMC4935009.
21. Muktabhant B, Lawrie TA, Lumbiganon P, Laopaiboon M. Diet or exercise, or both, for preventing excessive weight gain in pregnancy. The Cochrane database of systematic reviews. 2015;(6):Cd007145. Epub 2015/06/13. doi: 10.1002/14651858.CD007145.pub3. PubMed PMID: 26068707.

22. Rogozińska E, Chamillard M, Hitman GA, Khan KS, Thangaratinam S. Nutritional manipulation for the primary prevention of gestational diabetes mellitus: a meta-analysis of randomised studies. PloS one. 2015;10(2):e0115526. Epub 2015/02/27. doi: 10.1371/journal.pone.0115526. PubMed PMID: 25719363; PubMed Central PMCID: PMCPMC4342242.
23. International Weight Management in Pregnancy (IWiP) collaboration. Effect of diet and physical activity based interventions in pregnancy on gestational weight gain and pregnancy outcomes: meta-analysis of individual participant data from randomised trials. BMJ (Clinical research ed). 2017;358:j3119. doi: 10.1136/bmj.j3119.
24. Magro-Malosso ER, Saccone G, Di Mascio D, Di Tommaso M, Berghella V. Exercise during pregnancy and risk of preterm birth in overweight and obese women: a systematic review and meta-analysis of randomized controlled trials. Acta Obstet Gynecol Scand. 2017;96(3):263-73. Epub 2016/12/29. doi: 10.1111/aogs.13087. PubMed PMID: 28029178.
25. Chatzakis C, Goulis DG, Mareti E, Eleftheriades M, Zavlanos A, Dinas K, et al. Prevention of gestational diabetes mellitus in overweight or obese pregnant women: A network meta-analysis. Diabetes Res Clin Pract. 2019;158:107924. Epub 2019/11/19. doi: 10.1016/j.diabres.2019.107924. PubMed PMID: 31738997.
26. Du MC, Ouyang YQ, Nie XF, Huang Y, Redding SR. Effects of physical exercise during pregnancy on maternal and infant outcomes in overweight and obese pregnant women: A meta-analysis. Birth. 2019;46(2):211-21. Epub 2018/09/22. doi: 10.1111/birt.12396. PubMed PMID: 30240042.

Table S15: Meta-analysis for mortality outcomes reported by the included systematic reviews

Systematic review author, year	Type of intervention included in the reviews	Included population or intervention subgroup	N studies	Sample size pooled data	Meta-analysis: Mortality	Statistical significance
**Stillbirth**						
Chamberlain *et al.* 2017 [1]	Smoking	Counselling vs usual care	4	2,212	RR 1.08 (95% CI 0.51, 2.30)	Not significant
Chamberlain *et al.* 2017 [1]	Smoking	Feedback vs usual care	2	2,960	RR 1.28 (95% CI 0.69, 2.39)	Not significant
Shepherd *et al.* 2017 [2]	Diet and/or Physical Activity	Any BMI	5	4,783	RR 0.69 (95% CI 0.35, 1.36)	Not significant
IWiP 2017 [3]	Diet and/or physical activity	Recommended BMI, overweight, obese	2	3,719	OR 0.81 (95% CI <0.01, 256.69) (IPD)	Not significant
IWiP 2017 [3]	Diet and/or physical activity	Recommended BMI, overweight, obese	4	4,534	OR 0.85 (95% CI 0.24, 3.02) (IPD + non-IPD)	Not significant
Magro-Malosso *et al* 2017b [4]	Diet and/or Physical Activity	Overweight or obese	2	693	RR 2.13 (95% CI 0.22, 20.4)	Not significant
Tieu *et al.* 2017 [5]	Diet only	Any BMI	2	959	RR 3.09 (95% CI 0.13, 75.65)	Not significant p=0.49
**Intrauterine death**						
Thangaratinam *et al.* 2012 [6, 7]	Diet and/or Physical Activity	Recommended BMI, overweight, obese	2	1,320	RR 0.15 (95% CI 0.02, 1.20)	Not significant p=0.07
Chatzakis *et al*. 2019 [8]	Physical activity only	Overweight or obese	2	339	RR 0.63 (95% CI 0.08, 5.03)	Not significant
**Neonatal mortality**						
Chamberlain *et al.* 2017 [1]	Smoking	Counselling vs usual care	3	2,095	RR 2.06 (95% CI 0.61, 6.92)	Not significant
Shepherd *et al.* 2017 [2]	Diet and/or Physical Activity	Any BMI	2	3,756	RR 2.31 (95% CI 0.60, 8.90)	Not significant
**Perinatal mortality**						
Shepherd *et al.* 2017 [2]	Diet and/or Physical Activity	Any BMI	2	3,757	RR 0.82 (95% CI 0.42, 1.63)	Not significant p=0.58
Davenport *et al.* 2019a [9]	Diet and/or Physical Activity	Any BMI	13	6,837	OR 0.86 (95% CI 0.49, 1.52)	Not significant p=0.61
Gresham *et al.* 2014 [10]	Diet only	Any BMI	10	6,125	SMD -0.16 (95% CI -0.39, 0.08)	Not significant p=0.19
Davenport *et al.* 2019a [9]	Physical activity only	Any BMI	6	1,651	OR 0.79 (95% CI 0.26, 2.38)	Not significant p=0.68
**Miscarriage**						
Davenport *et al.* 2019a [9]	Diet and/or Physical Activity	Any BMI	23	7,125	OR 0.88 (95% CI 0.63, 1.21)	Not significant p=0.43
Davenport *et al.* 2019a [9]	Physical activity only	Any BMI	10	2,248	OR 0.69 (95% CI 0.40, 1.22)	Not significant p=0.20

Footnote: BMI – body mass index, N – number, RR – risk ratio, OR – odds ratio, CI – confidence interval, IPD – individual participant data, MA – meta-analysis, SMD – standardised mean difference. [#]Thangaratinam *et al.* 2012 [6] and Thangaratinam *et al.* 2012b [7] reported the same meta-analysis.

S15 References

1. Chamberlain C, O'Mara-Eves A, Porter J, Coleman T, Perlen SM, Thomas J, et al. Psychosocial interventions for supporting women to stop smoking in pregnancy. The Cochrane database of systematic reviews. 2017;2:Cd001055. Epub 2017/02/15. doi: 10.1002/14651858.CD001055.pub5. PubMed PMID: 28196405; PubMed Central PMCID: PMCPMC6472671.

2. Shepherd E, Gomersall JC, Tieu J, Han S, Crowther CA, Middleton P. Combined diet and exercise interventions for preventing gestational diabetes mellitus. The Cochrane database of systematic reviews. 2017;11:Cd010443. Epub 2017/11/13. doi: 10.1002/14651858.CD010443.pub3. PubMed PMID: 29129039; PubMed Central PMCID: PMCPMC6485974.
3. International Weight Management in Pregnancy (IWiP) collaboration. Effect of diet and physical activity based interventions in pregnancy on gestational weight gain and pregnancy outcomes: meta-analysis of individual participant data from randomised trials. BMJ (Clinical research ed). 2017;358:j3119. doi: 10.1136/bmj.j3119.
4. Magro-Malosso ER, Saccone G, Di Mascio D, Di Tommaso M, Berghella V. Exercise during pregnancy and risk of preterm birth in overweight and obese women: a systematic review and meta-analysis of randomized controlled trials. Acta Obstet Gynecol Scand. 2017;96(3):263-73. Epub 2016/12/29. doi: 10.1111/aogs.13087. PubMed PMID: 28029178.
5. Tieu J, Shepherd E, Middleton P, Crowther CA. Dietary advice interventions in pregnancy for preventing gestational diabetes mellitus. The Cochrane database of systematic reviews. 2017;1:Cd006674. Epub 2017/01/04. doi: 10.1002/14651858.CD006674.pub3. PubMed PMID: 28046205; PubMed Central PMCID: PMCPMC6464792.
6. Thangaratinam S, Rogozińska E, Jolly K, Glinkowski S, Duda W, Borowiack E, et al. Interventions to reduce or prevent obesity in pregnant women: a systematic review. 2012;16:31. doi: 10.3310/hta16310.
7. Thangaratinam S, Rogozińska E, Jolly K, Glinkowski S, Roseboom T, Tomlinson JW, et al. Effects of interventions in pregnancy on maternal weight and obstetric outcomes: meta-analysis of randomised evidence. BMJ : British Medical Journal. 2012;344:e2088. doi: 10.1136/bmj.e2088.
8. Chatzakis C, Goulis DG, Mareti E, Eleftheriades M, Zavlanos A, Dinas K, et al. Prevention of gestational diabetes mellitus in overweight or obese pregnant women: A network meta-analysis. Diabetes Res Clin Pract. 2019;158:107924. Epub 2019/11/19. doi: 10.1016/j.diabres.2019.107924. PubMed PMID: 31738997.
9. Davenport MH, Kathol AJ, Mottola MF, Skow RJ, Meah VL, Poitras VJ, et al. Prenatal exercise is not associated with fetal mortality: a systematic review and meta-analysis. Br J Sports Med. 2019;53(2):108-15. Epub 2018/10/20. doi: 10.1136/bjsports-2018-099773. PubMed PMID: 30337346.
10. Gresham E, Byles JE, Bisquera A, Hure AJ. Effects of dietary interventions on neonatal and infant outcomes: a systematic review and meta-analysis. Am J Clin Nutr. 2014;100(5):1298-321. Epub 2014/10/22. doi: 10.3945/ajcn.113.080655. PubMed PMID: 25332328.

Table S16: Meta-analysis for neonatal intensive care unit (NICU) admission reported by the included systematic reviews

Systematic review author, year	Included population or intervention subgroup	N studies	Sample size pooled data	Meta-analysis: NICU	Statistical significance
**Smoking**					
Chamberlain *et al.* 2017 [1]	Counselling vs usual care	2	1,140	RR 0.82 (95% CI 0.52, 1.29)	Not significant
Chamberlain *et al.* 2017 [1]	Incentives vs usual care	2	68	RR 0.80 (95% CI 0.51, 1.26)	Not significant
Chamberlain *et al.* 2017 [1]	Incentives vs alternative intervention	3	184	RR 0.64 (95% CI 0.26, 1.55)	Not significant
**Diet and/or Physical Activity**					
Bain *et al.* 2015 [2]	Any BMI	2	2,446	RR 1.01 (95% CI 0.91, 1.13)	Not significant
Rogozińska *et al.* 2015 [3]	Any BMI	2	2,562	RR 1.01 (95% CI 0.91, 1.13)	Not significant p=0.82
Shepherd *et al.* 2017 [4]	Any BMI	4	4,549	RR 1.03 (95% CI 0.93, 1.14)	Not significant
Thangaratinam *et al.* 2012 [5, 6][#]	Recommended BMI, overweight, obese	2	1,962	RR 0.98 (95% CI 0.66, 1.47)	Not significant p=0.93
Thangaratinam *et al.* 2012b [6]	Recommended BMI, overweight, obese	3	2,266	RR 1.00 (95% CI 0.75, 1.33)	Not significant p=1.00
IWiP 2017 [7]	Recommended BMI, overweight, obese	16	8,140	OR 1.01 (95% CI 0.84, 1.23) (IPD MA)	Not significant
IWiP 2017 [7]	Recommended BMI, overweight, obese	21	9,498	OR 0.97 (95% CI 0.82, 1.14) (IPD + non-IPD)	Not significant
**Diet only**					
IWiP 2017 [7]	Recommended BMI, overweight, obese	2	389	OR 0.33 (95% CI <0.01, 47.97) (IPD + non-IPD)	Not significant
**Physical activity only**					
IWiP 2017 [7]	Recommended BMI, overweight, obese	3	1,166	OR 0.77 (95% CI 0.21, 2.81) (IPD MA)	Not significant
IWiP 2017 [7]	Recommended BMI, overweight, obese	4	1,240	OR 0.79 (95% CI 0.35, 1.78) (IPD + non-IPD)	Not significant
Chatzakis *et al*. 2019 [8]	Overweight or obese	2	161	RR 0.69 (95% CI 0.27, 1.73)	Not significant

Footnote: NICU – neonatal intensive care unit, BMI – body mass index, N – number, RR – risk ratio, OR – odds ratio, CI – confidence interval, IPD – individual participant data, MA – meta-analysis. [#] Thangaratinam *et al.* 2012 [5] and Thangaratinam *et al.* 2012b [6] reported the same meta-analysis data in both systematic reviews.

S16 References

1. Chamberlain C, O'Mara-Eves A, Porter J, Coleman T, Perlen SM, Thomas J, et al. Psychosocial interventions for supporting women to stop smoking in pregnancy. The Cochrane database of systematic reviews. 2017;2:Cd001055. Epub 2017/02/15. doi: 10.1002/14651858.CD001055.pub5. PubMed PMID: 28196405; PubMed Central PMCID: PMCPMC6472671.
2. Bain E, Crane M, Tieu J, Han S, Crowther CA, Middleton P. Diet and exercise interventions for preventing gestational diabetes mellitus. The Cochrane database of systematic reviews. 2015;(4):Cd010443. Epub 2015/04/13. doi: 10.1002/14651858.CD010443.pub2. PubMed PMID: 25864059.
3. Rogozińska E, Chamillard M, Hitman GA, Khan KS, Thangaratinam S. Nutritional manipulation for the primary prevention of gestational diabetes mellitus: a meta-analysis of randomised studies. PloS one. 2015;10(2):e0115526. Epub 2015/02/27. doi: 10.1371/journal.pone.0115526. PubMed PMID: 25719363; PubMed Central PMCID: PMCPMC4342242.

4. Shepherd E, Gomersall JC, Tieu J, Han S, Crowther CA, Middleton P. Combined diet and exercise interventions for preventing gestational diabetes mellitus. The Cochrane database of systematic reviews. 2017;11:Cd010443. Epub 2017/11/13. doi: 10.1002/14651858.CD010443.pub3. PubMed PMID: 29129039; PubMed Central PMCID: PMCPMC6485974.
5. Thangaratinam S, Rogozińska E, Jolly K, Glinkowski S, Duda W, Borowiack E, et al. Interventions to reduce or prevent obesity in pregnant women: a systematic review. 2012;16:31. doi: 10.3310/hta16310.
6. Thangaratinam S, Rogozińska E, Jolly K, Glinkowski S, Roseboom T, Tomlinson JW, et al. Effects of interventions in pregnancy on maternal weight and obstetric outcomes: meta-analysis of randomised evidence. BMJ : British Medical Journal. 2012;344:e2088. doi: 10.1136/bmj.e2088.
7. International Weight Management in Pregnancy (IWiP) collaboration. Effect of diet and physical activity based interventions in pregnancy on gestational weight gain and pregnancy outcomes: meta-analysis of individual participant data from randomised trials. BMJ (Clinical research ed). 2017;358:j3119. doi: 10.1136/bmj.j3119.
8. Chatzakis C, Goulis DG, Mareti E, Eleftheriades M, Zavlanos A, Dinas K, et al. Prevention of gestational diabetes mellitus in overweight or obese pregnant women: A network meta-analysis. Diabetes Res Clin Pract. 2019;158:107924. Epub 2019/11/19. doi: 10.1016/j.diabres.2019.107924. PubMed PMID: 31738997.

Table S17: Meta-analysis for Apgar score reported by the included systematic reviews

Systematic review author, year	Type of intervention included in the reviews	Included population or intervention subgroup	N studies	Sample size pooled data	Meta-analysis: Apgar score	Statistical significance
**Apgar score <7 at 5 minutes**						
Shepherd *et al.* 2017 [1]	Diet and/or Physical Activity	Any BMI	3	2,864	RR 0.80 (95% CI 0.48, 1.32)	Not significant
Thangaratinam *et al.* 2012 [2]	Diet and/or Physical Activity	Recommended BMI, overweight, obese	3	1,497	RR 0.64 (95% CI 0.27, 1.49)	Not significant p=0.30
Gresham *et al.* 2014 [3]	Diet only	Any BMI	3	1,698	SMD 0.20 (-0.21, 0.62)	Not significant p=0.34
Zheng *et al.* 2017 [4]	Physical activity only	Any BMI	2	993	OR 0.78 (95% CI 0.21, 2.91)	Not significant p=0.71
Han *et al.* 2012 [5]	Physical activity only	Any BMI	2	919	RR 1.00 (95% CI 0.27, 3.65)	Not significant p=1.0
**Apgar score at 1 minute**						
Magro-Malosso *et al* 2017a [6]	Diet and/or Physical Activity	Any BMI	11	2,530	MD 0.01 (95% CI -0.15, 0.17)	Not significant
Sanabria-Martínez *et al.* 2016 [7]	Physical activity only	Any BMI	9	2,455	ES 0.08 (95% CI 0.00, 0.16)	Not significant p=0.063
**Apgar score at 5 minutes**						
Magro-Malosso *et al* 2017a [6]	Diet and/or Physical Activity	Any BMI	11	2,530	MD 0.01 (95% CI -0.05, 0.07)	Not significant
Thangaratinam *et al.* 2012 [2]	Diet and/or Physical Activity	Recommended BMI, overweight, obese	3	502	MD 0.00 (95% CI -0.05, 0.05)	Not significant p=0.94
Gresham *et al.* 2014 [3]	Diet only	Any BMI	3	518	SMD 0.15 (95% CI -0.31, 0.62)	Not significant p=0.52
Sanabria-Martínez *et al.* 2016 [7]	Physical activity only	Any BMI	9	2,455	ES 0.01 (95% CI -0.07, 0.09)	Not significant p=0.800

Footnote: BMI – body mass index, N – number, RR – risk ratio, OR – odds ratio, CI – confidence interval, MD – mean difference, SMD – standardised mean difference, ES – effect size.

S17 References

1. Shepherd E, Gomersall JC, Tieu J, Han S, Crowther CA, Middleton P. Combined diet and exercise interventions for preventing gestational diabetes mellitus. The Cochrane database of systematic reviews. 2017;11:Cd010443. Epub 2017/11/13. doi: 10.1002/14651858.CD010443.pub3. PubMed PMID: 29129039; PubMed Central PMCID: PMCPMC6485974.
2. Thangaratinam S, Rogozińska E, Jolly K, Glinkowski S, Duda W, Borowiack E, et al. Interventions to reduce or prevent obesity in pregnant women: a systematic review. 2012;16:31. doi: 10.3310/hta16310.
3. Gresham E, Byles JE, Bisquera A, Hure AJ. Effects of dietary interventions on neonatal and infant outcomes: a systematic review and meta-analysis. Am J Clin Nutr. 2014;100(5):1298-321. Epub 2014/10/22. doi: 10.3945/ajcn.113.080655. PubMed PMID: 25332328.
4. Zheng J, Wang H, Ren M. Influence of exercise intervention on gestational diabetes mellitus: a systematic review and meta-analysis. J Endocrinol Invest. 2017;40(10):1027-33. Epub 2017/04/13. doi: 10.1007/s40618-017-0673-3. PubMed PMID: 28401529.
5. Han S, Middleton P, Crowther CA. Exercise for pregnant women for preventing gestational diabetes mellitus. The Cochrane database of systematic reviews. 2012;(7):Cd009021. Epub 2012/07/13. doi: 10.1002/14651858.CD009021.pub2. PubMed PMID: 22786521.
6. Magro-Malosso ER, Saccone G, Di Tommaso M, Roman A, Berghella V. Exercise during pregnancy and risk of gestational hypertensive disorders: a systematic review and meta-analysis. Acta Obstetricia et Gynecologica Scandinavica. 2017;96(8):921-31. doi: 10.1111/aogs.13151.

7. Sanabria-Martínez G, García-Hermoso A, Poyatos-León R, González-García A, Sánchez-López M, Martínez-Vizcaíno V. Effects of Exercise-Based Interventions on Neonatal Outcomes: A Meta-Analysis of Randomized Controlled Trials. Am J Health Promot. 2016;30(4):214-23. Epub 2016/07/13. doi: 10.1177/0890117116639569. PubMed PMID: 27404056.

Table S18: Meta-analysis for other measures of infant health-related outcomes reported by the included systematic reviews

Systematic review author, year	Type of intervention included in the reviews	Included population or intervention subgroup	N studies	Sample size pooled data	Meta-analysis	Statistical significance
**Shoulder dystocia**						
Rogozińska *et al.* 2015 [1]	Diet and/or Physical Activity	Any BMI	2	2,506	RR 1.24 (95% CI 0.81, 1.91)	Not significant p=0.33
Muktabhant *et al.* 2015 [2]	Diet and/or Physical Activity	Any BMI	4	3,253	RR 1.02 (95% CI 0.57, 1.83)	Not significant
Shepherd *et al.* 2017 [3]	Diet and/or Physical Activity	Any BMI	2	2,733	RR 1.20 (95% CI 0.79, 1.83)	Not significant
**Thangaratinam *et al.* 2012 [4, 5] #**	**Diet and/or Physical Activity**	**Recommended BMI, overweight, obese**	**4**	**2,317**	**RR 0.39 (95% CI 0.22, 0.70)**	**Significantly reduced p=0.02**
**Thangaratinam *et al.* 2012 [4, 5] #**	**Diet only**	**Recommended BMI, overweight, obese**	**3**	**2,082**	**RR 0.38 (95% CI 0.21, 0.69)**	**Significantly reduced p=0.001**
**Neonatal / infant hypoglycaemia**						
Muktabhant *et al.* 2015 [2]	Diet and/or Physical Activity	Any BMI	4	2,601	RR 0.95 (95% CI 0.76, 1.18)	Not significant
Shepherd *et al.* 2017 [3]	Diet and/or Physical Activity	Any BMI	2	3,653	RR 1.42 (95% CI 0.67, 2.98)	Not significant
Thangaratinam *et al.* 2012 [4, 5] #	Diet and/or Physical Activity	Recommended BMI, overweight, obese	5	2,146	RR 1.07 (95% CI 0.85, 1.35)	Not significant p=0.55
Thangaratinam *et al.* 2012 [4, 5] #	Diet only	Recommended BMI, overweight, obese	3	1,877	RR 1.05 (95% CI 0.83, 1.33)	Not significant p=0.69
**Respiratory distress syndrome**						
**Muktabhant *et al.* 2015 [2]**	**Diet and/or Physical Activity**	**Any BMI**	**2**	**2,256**	**RR 0.47 (95% CI 0.26, 0.85)**	**Significantly reduced**
**Shepherd *et al.* 2017 [3]**	**Diet and/or Physical Activity**	**Any BMI**	**2**	**2,411**	**RR 0.56 (95% CI 0.33, 0.97)**	**Significantly reduced**
Thangaratinam *et al.* 2012 [4, 5] #	Diet and/or Physical Activity	Recommended BMI, overweight, obese	2	1,962	RR 1.05 (95% CI 0.48, 2.28)	Not significant p=0.91
**Infant hyperbilirubinaemia**						
Muktabhant *et al.* 2015 [2]	Diet and/or Physical Activity	Any BMI	2	2,256	RR 0.83 (95% CI 0.62, 1.10)	Not significant
Thangaratinam *et al.* 2012b [4, 5] #	Diet and/or Physical Activity	Recommended BMI, overweight, obese	2	1,898	RR 0.84 (95% CI 0.64, 1.10)	Not significant p=0.19
**Birth trauma**						
Thangaratinam *et al.* 2012 [4, 5] #	Diet and/or Physical Activity	Recommended BMI, overweight, obese	2	1,961	RR 0.36 (95% CI 0.11, 1.23)	Not significant p=0.10
Muktabhant *et al.* 2015 [2]	Diet and/or Physical Activity	Any BMI	2	2,256	RR 0.89 (95% CI 0.35, 2.30)	Not significant
**Placental weight**						
Gresham *et al.* 2014 [6]	Diet only	Any BMI	6	1,016	SMD 0.04g (95% CI -0.12, 0.20)	Not significant p=0.66
**PROM**						
Davenport *et al.* 2019 [7]	Diet and/or Physical Activity	Any BMI	4	337	OR 1.01 (95% CI 0.38, 2.68)	Not significant p=0.99
**Breastfeeding (at 6 months)**						
Shepherd *et al.* 2017 [3]	Diet and/or Physical Activity	Any BMI	2	921	RR 0.91 (95% CI 0.61, 1.36) (Exclusive)	Not significant
Shepherd *et al.* 2017 [3]	Diet and/or Physical Activity	Any BMI	2	921	RR 0.98 (95% CI 0.82, 1.18) (Partial)	Not significant
**Composite offspring outcomes: stillbirth, SGA, LGA, NICU**						
IWiP 2017 [8]	Diet and/or physical activity	Recommended BMI, overweight, obese	18	7,981	OR 0.94 (95% CI 0.83, 1.08)	Not significant
IWiP 2017 [8]	Diet only	Recommended BMI, overweight, obese	2	346	OR 0.71 (95% CI 0.03, 18.23)	Not significant
IWiP 2017 [8]	Physical activity only	Recommended BMI, overweight, obese	5	1,274	OR 0.99 (95% CI 0.67, 1.46)	Not significant

Footnote: BMI – body mass index, N – number, RR – risk ratio, OR – odds ratio, SMD – standardised mean difference, CI – confidence interval, g - grams. Bold data highlights statistically significant results. #Thangaratinam *et al.* 2012 [4] and Thangaratinam *et al.* 2012b [5] reported the same meta-analysis.

S18 References

1. Rogozińska E, Chamillard M, Hitman GA, Khan KS, Thangaratinam S. Nutritional manipulation for the primary prevention of gestational diabetes mellitus: a meta-analysis of randomised studies. PloS one. 2015;10(2):e0115526. Epub 2015/02/27. doi: 10.1371/journal.pone.0115526. PubMed PMID: 25719363; PubMed Central PMCID: PMCPMC4342242.
2. Muktabhant B, Lawrie TA, Lumbiganon P, Laopaiboon M. Diet or exercise, or both, for preventing excessive weight gain in pregnancy. The Cochrane database of systematic reviews. 2015;(6):Cd007145. Epub 2015/06/13. doi: 10.1002/14651858.CD007145.pub3. PubMed PMID: 26068707.
3. Shepherd E, Gomersall JC, Tieu J, Han S, Crowther CA, Middleton P. Combined diet and exercise interventions for preventing gestational diabetes mellitus. The Cochrane database of systematic reviews. 2017;11:Cd010443. Epub 2017/11/13. doi: 10.1002/14651858.CD010443.pub3. PubMed PMID: 29129039; PubMed Central PMCID: PMCPMC6485974.
4. Thangaratinam S, Rogozińska E, Jolly K, Glinkowski S, Duda W, Borowiack E, et al. Interventions to reduce or prevent obesity in pregnant women: a systematic review. 2012;16:31. doi: 10.3310/hta16310.
5. Thangaratinam S, Rogozińska E, Jolly K, Glinkowski S, Roseboom T, Tomlinson JW, et al. Effects of interventions in pregnancy on maternal weight and obstetric outcomes: meta-analysis of randomised evidence. BMJ : British Medical Journal. 2012;344:e2088. doi: 10.1136/bmj.e2088.
6. Gresham E, Byles JE, Bisquera A, Hure AJ. Effects of dietary interventions on neonatal and infant outcomes: a systematic review and meta-analysis. Am J Clin Nutr. 2014;100(5):1298-321. Epub 2014/10/22. doi: 10.3945/ajcn.113.080655. PubMed PMID: 25332328.
7. Davenport MH, Ruchat SM, Sobierajski F, Poitras VJ, Gray CE, Yoo C, et al. Impact of prenatal exercise on maternal harms, labour and delivery outcomes: a systematic review and meta-analysis. Br J Sports Med. 2019;53(2):99-107. Epub 2018/10/20. doi: 10.1136/bjsports-2018-099821. PubMed PMID: 30337349.
8. International Weight Management in Pregnancy (IWiP) collaboration. Effect of diet and physical activity based interventions in pregnancy on gestational weight gain and pregnancy outcomes: meta-analysis of individual participant data from randomised trials. BMJ (Clinical research ed). 2017;358:j3119. doi: 10.1136/bmj.j3119.

S19 Table: Author reported conflicts of interest for systematic reviews reporting outcomes for each behavioural domain, and overall quality for all included systematic reviews

S19a: Systematic reviews reporting meta-analysis of smoking cessation interventions

Systematic review author, year	Author reported conflict of interest
Chamberlain *et al.* 2017 [1]	Authors declare: "Catherine Chamberlain is receiving an Australian National Health and Medical Research Council Early Career Fellowship (1088813). She was also awarded an NIHR Cochrane Review Incentive Scheme award (15/81/18) to support the preparation of this updated review. Catherine Chamberlain is also an author on the Cochrane Review entitled 'Pharmacological Interventions to promote smoking cessation in pregnancy" (Coleman 2015). Alison O'Mara-Eves: none known. Jessie Porter: none known. Tim Coleman has received awards from NIHR, HTA and NIHR paid to his institution. These awards have been used to run research projects. He has also received a single payment from Pierre Fabre Laboratories, France, for speaking at an educational meeting arranged by Pierre Fabre Laboratories (who are manufacturers of nicotine replacement therapy). PFL are a manufacturer of transdermal nicotine patches - the content of the presentation was not vetted and no attempt was made to influence the content of the presentation. Tim Coleman is also an author and contact person for the Cochrane Review entitled 'Pharmacological Interventions to promote smoking cessation in pregnancy" (Coleman 2015). Susan M Perlen: none known. James Thomas: none known. Joanne E McKenzie: none known."
Veisani *et al.* 2017 [2]	Authors declare: "No potential conflict of interest was reported by the authors."

S19b: Systematic reviews reporting meta-analysis of diet and/or physical activity interventions

Systematic review author, year	Author reported conflict of interest
Agha *et al.* 2014 [3]	Authors declare: "The authors have declared that no competing interests exist"
Bain *et al.* 2015 [4]	Authors declare: "Emily Bain: none known. Morven Crane: none known. Joanna Tieu: none known. Caroline Crowther was an investigator on the LIMIT Trial (Dodd 2014). All tasks relating to this study (assessment of eligibility for inclusion, assessment of risk of bias, data extraction) were carried out by other members of the review team who were not directly involved in the trial"
Allen *et al.* 2014 [5]	Authors declare: "No support from any organization for the submitted work; no financial relationships with any organizations that might have an interest in the submitted work in the previous 3 years; no other relationships or activities that could appear to have influenced the submitted work"
Campbell *et al*. 2011 [6]	Authors declare: "No support from any organization for the submitted work; no financial relationships with any organizations that might have an interest in the submitted work in the previous 3 years; no other relationships or activities that could appear to have influenced the submitted work"
Choi *et al.* 2013 [7]	Authors declare: "There are no conflicts of interest"
Gardner *et al.* 2011 [8]	Authors declare: "BG, JW and HC declare that they have no conflicts of interest. LP has received payment from ILSI Europe as reimbursement of expenses incurred in attending a workshop on obese pregnancy and long-term outcomes, and was paid as a member of the Tate and Lyle Research Advisory Group from 2007 to 2010, prior to submission of this work"
Hill *et al.* 2013 [9]	Authors declare: "The authors declare no conflict of interest"
Madhuvrata *et al.* 2015 [10]	Authors declare: "None known"
Muktabhant *et al.* 2015 [11]	Authors declare: "Declaration of Interest: None known"
O'Brien *et al.* 2015 [12]	Authors declare: "The authors declare no conflict of interest"
Oteng-Ntim *et al.* 2012 [13]	Authors declare: "The authors declare that they have no competing interests"
Rogozińska *et al.* 2015 [14]	Authors declare: "The authors have declared that no competing interests exist"
Tanentsapf *et al.* 2011 [15]	Authors declare: "The authors declare that they have no competing interests"
Thangaratinam *et al.* 2012a [16]	Authors declare: "None declared"
Thangaratinam *et al.* 2012b [17]	Authors declare: "Competing interest: All authors have completed the ICMJE uniform disclosure form at www.icmje.org/coi_disclosure.pdf (available on request from the corresponding author) and declare: no support from any organisation for the submitted work; no financial relationships with any organisations that might have an interest in the submitted work in the previous three years; no other relationships or activities that could appear to have influenced the submitted work."
IWiP 2017 [18]	Authors declare: "No support from any organisation for the submitted work."
Lau *et al.* 2017 [19]	Authors declare: "no conflict of interest"
Magro-Malosso *et al* 2017a [20]	Authors declare: "that there are no conflicts of interest in connection with this article."
Magro-Malosso *et al* 2017b [21]	Authors declare: "that there are no conflicts of interest in connection with this article."
Shepherd *et al.* 2017 [22]	Authors declare: "Emily Shepherd: none known. Judith Gomersall: none known. Joanna Tieu has received funding for work outside of the scope of this review- NHMRC postgraduate scholarship, Ken Muirden fellowship (administered by Arthritis Australia; jointly funded by Australian Rheumatology Association and Roche). Shanshan Han: Shanshan Han was an investigator on one of the excluded trials (Crowther 2012). Assessment of eligibility for inclusion was carried out by other members of the review team who were not directly involved in the trial. Caroline Crowther: Caroline Crowther was an investigator on one of the included trials (Dodd 2014), and one of the excluded trials (Crowther 2012). All tasks relating to these trials (assessment of eligibility for inclusion, and if applicable, data extraction and assessment of risk of bias) were carried out by other members of the review team who were not directly involved in the trials. Philippa Middleton: Philippa Middleton was an investigator on one of the excluded trials (Crowther 2012). Assessment of eligibility for inclusion was carried out by other members of the review team who were not directly involved in the trial."
Sherifali *et al.* 2017 [23]	Authors declare: "None declared"
Song *et al.* 2016 [24]	Authors declare: "All the authors declared no conflicts of interest"
Yeo *et al.* 2017 [25]	Authors declare; "No conflict of interest was declared"
Craemer *et al.* 2019 [26]	Authors declare: "The authors of this study declare no conflict of interest"
Davenport *et al.* 2019a [27]	Authors declare: "None declared"
Davenport *et al.* 2018a [28]	Authors declare: "None declared"
Davenport *et al.* 2018b [29]	Authors declare: "No competing interest"
Davenport *et al.* 2019b [30]	Authors declare: "No competing interest"

Systematic review author, year	Author reported conflict of interest
Davenport *et al.* 2018c [31]	Authors declare: "None declared"
Michel S *et al.* 2019 [32]	Authors declare; "No conflict of interest was declared"
Morison *et al.* 2018 [33]	Authors declare: "No conflict of interest"
Ruchat *et al.* 2018 [34]	Authors declare: "None declared"
Syngelaki *et al.* 2019 [35]	Authors declare "No potential conflict of interest"
Walker *et al.* 2018 [36]	Authors declare: "The authors declared no conflicts of interest"
Vincze *et al.* 2019 [37]	Authors declare: "There is no conflict of interest in this project"
Shieh *et al.* 2018 [38]	Authors declare: "Authors have no conflict of interest to declare"
Bennett *et al.* 2017 [39]	Authors declare: "The authors have no conflicts of interest to declare"

S19c: Systematic reviews reporting meta-analysis of diet only interventions

Systematic review author, year	Author reported conflict of interest
Allen *et al.* 2014 [5]	Authors declare: "No support from any organization for the submitted work; no financial relationships with any organizations that might have an interest in the submitted work in the previous 3 years; no other relationships or activities that could appear to have influenced the submitted work"
Gresham *et al.* 2016 [40]	Authors declare: "The authors declare that they have no conflicts of interest"
Madhuvrata *et al.* 2015 [10]	Authors declare: "None known"
Muktabhant *et al.* 2015 [11]	Authors declare: "Declaration of Interest: None known"
Oostdam *et al.* 2011 [41]	Authors declare: "There are no potential conflicts of interest in connection with this article"
Quinlivan *et al.* 2011 [42]	Authors declare: "The authors did not report any potential conflicts of interest"
Rogozińska *et al.* 2015 [14]	Authors declare: "The authors have declared that no competing interests exist"
Thangaratinam *et al.* 2012a [16]	Authors declare: "None declared"
Thangaratinam *et al.* 2012b [17]	Authors declare: "Competing interest: All authors have completed the ICMJE uniform disclosure form at www.icmje.org/coi_disclosure.pdf (available on request from the corresponding author) and declare: no support from any organisation for the submitted work; no financial relationships with any organisations that might have an interest in the submitted work in the previous three years; no other relationships or activities that could appear to have influenced the submitted work."
IWiP 2017 [18]	Authors declare: "No support from any organisation for the submitted work."
Song *et al.* 2016 [24]	Authors declare: "All the authors declared no conflicts of interest"
Tieu *et al.* 2017 [43]	Authors declare: "Declarations of interest: Joanna Tieu: none known. Emily Shepherd: none known. Philippa Middleton: none known. Caroline A Crowther: none known."
Zhang *et al.* 2018 [44]	Authors declare: "Irma Silva-Zolezzi is employee of Nestlé Research Center, Lausanne. Gerard Vinyes Parés and Yi Wang are employees of Nestlé Research Center Beijing. The authors state that there is no conflict of interest"
Gresham *et al.* 2014 [45]	Authors declare: "None of the authors had a conflict of interest"
Craemer *et al.* 2019 [26]	Authors declare: "There are no conflicts of interest"
Syngelaki *et al.* 2019 [35]	Authors declare "No potential conflict of interest"
Walker *et al.* 2018 [36]	Authors declare: "The authors declared no conflicts of interest"
Shieh *et al.* 2018 [38]	Authors declare: "Authors have no conflict of interest to declare"
Bennett *et al.* 2017 [39]	Authors declare: "The authors have no conflicts of interest to declare"

S19d: Systematic reviews reporting meta-analysis of physical activity only interventions

Systematic review author, year	Author reported conflict of interest
Elliot-Sale *et al.* 2015 [46]	Authors declare: "The authors declare no conflict of interest"
Han *et al.* 2012 [47]	Authors declare: "None known"
Lamina & Agbanusi 2013 [48]	Authors declare: "No any conflict of interest on this review."
Madhuvrata *et al.* 2015 [10]	Authors declare: "None known"
Oostdam *et al.* 2011 [41]	Authors declare: "There are no potential conflicts of interest in connection with this article"
Russo *et al.* 2015 [49]	Authors declare: "The authors did not report any potential conflicts of interest"
Sanabria-Martínez *et al.* 2015 [50]	Authors declare: "None declared"
Streuling *et al.* 2011 [51]	Authors declare: "None of the authors had any conflicts of interest"
Sui *et al.* 2012 [52]	Authors declare: "The authors have stated explicitly that there are no conflicts of interest in connection with this article"
Thangaratinam *et al.* 2012a [16]	Authors declare: "None declared"
Thangaratinam *et al.* 2012b [17]	Authors declare: "Competing interest: All authors have completed the ICMJE uniform disclosure form at www.icmje.org/coi_disclosure.pdf (available on request from the corresponding author) and declare: no support from any organisation for the submitted work; no financial relationships with any organisations that might have an interest in the submitted work in the previous three years; no other relationships or activities that could appear to have influenced the submitted work."
da Silva *et al.* 2017 [53]	Authors declare: "Shana G da Silva, Luiza I Ricardo, Kelly R Evenson, and Pedro C Hallal have no conflicts of interest relevant to the content of this review."
Di Mascio *et al.* 2016 [54]	Authors declare: "The authors report no conflict of interest."
IWiP 2017 [18]	Authors declare: "No support from any organisation for the submitted work."
Sanabria-Martínez *et al.* 2016 [55]	Authors declare: "The author(s) declared no potential conflicts of interest with respect to the research, authorship, and/or publication of this article "
Song *et al.* 2016 [24]	Authors declare: "All the authors declared no conflicts of interest"
Zheng *et al.* 2017 [56]	Authors declare: "The authors declare no conflict of interest"
Chatzakis *et al.* 2019 [57]	Authors declare: "The authors have no conflict of interest to declare"
Craemer *et al.* 2019 [26]	Authors declare: "The authors of this study declare no conflict of interest"
Davenport *et al.* 2019a [27]	Authors declare: "None declared"
Davenport *et al.* 2018a [28]	Authors declare: "None declared"
Davenport *et al.* 2018b [29]	Authors declare: "No competing interest"
Davenport *et al.* 2019b [30]	Authors declare: "No competing interest"

Systematic review author, year	Author reported conflict of interest
Davenport *et al.* 2018c [31]	Authors declare: “None declared”
**Du *et al.* 2019 [58]**	**No statement of conflict of interest included in the published systematic review**
Guillemette *et al.* 2018 [59]	Authors declare: “Laetitia Guillemette, Jacqueline L. Hay, D. Scott Kehler, Naomi C. Hamm, Christopher Oldfield, Jonathan M. McGavock, and Todd A. Duhamel declare that they have no competing interests”
Ming *et al.* 2018 [60]	Authors declare: “No competing interests”
Nasiri-Amiri *et al.* 2019 [61]	Authors declare: “No competing interests”
Ruchat *et al.* 2018 [34]	Authors declare: “None declared”
Syngelaki *et al.* 2019 [35]	Authors declare “No potential conflict of interest”
Wang *et al.* 2019 [62]	Authors declare: “No conflicts of interest to disclose”
Poyatos-Leon *et al.* 2015 [63]	Authors declare: “The authors have stated explicitly that there are no conflicts of interest in connection with this article”
Walker *et al.* 2018 [36]	Authors declare: “The authors declared no conflicts of interest”
Wiebe *et al.* 2015 [64]	Authors declare: “The authors did not report any potential conflicts of interest”
Yin *et al.* 2013 [65]	Authors declare: “Competing interests – none”
Shieh *et al.* 2018 [38]	Authors declare: “Authors have no conflict of interest to declare”
Bennett *et al.* 2017 [39]	Authors declare: “The authors have no conflicts of interest to declare”

Table S19e: All included systematic reviews

Systematic review author, year	Author reported conflict of interest
Agha *et al.* 2014 [3]	Authors declare: “The authors have declared that no competing interests exist”
Bain *et al.* 2015 [4]	Authors declare: “Emily Bain: none known. Morven Crane: none known. Joanna Tieu: none known. Caroline Crowther was an investigator on the LIMIT Trial (Dodd 2014). All tasks relating to this study (assessment of eligibility for inclusion, assessment of risk of bias, data extraction) were carried out by other members of the review team who were not directly involved in the trial”
Allen *et al.* 2014 [5]	Authors declare: “No support from any organization for the submitted work; no financial relationships with any organizations that might have an interest in the submitted work in the previous 3 years; no other relationships or activities that could appear to have influenced the submitted work”
Campbell *et al*. 2011 [6]	Authors declare: “No support from any organization for the submitted work; no financial relationships with any organizations that might have an interest in the submitted work in the previous 3 years; no other relationships or activities that could appear to have influenced the submitted work”
Choi *et al.* 2013 [7]	Authors declare: “There are no conflicts of interest”
Elliot-Sale *et al.* 2015 [46]	Authors declare: “The authors declare no conflict of interest”
Gardner *et al.* 2011 [8]	Authors declare: “BG, JW and HC declare that they have no conflicts of interest. LP has received payment from ILSI Europe as reimbursement of expenses incurred in attending a workshop on obese pregnancy and long-term outcomes, and was paid as a member of the Tate and Lyle Research Advisory Group from 2007 to 2010, prior to submission of this work”
Gresham *et al.* 2016 [40]	Authors declare: “The authors declare that they have no conflicts of interest”
Han *et al.* 2012 [47]	Authors declare: “None known”
Hill *et al.* 2013 [9]	Authors declare: “The authors declare no conflict of interest”
Lamina & Agbanusi 2013 [48]	Authors declare: “No any conflict of interest on this review.”
Madhuvrata *et al.* 2015 [10]	Authors declare: “None known”
Muktabhant *et al.* 2015 [11]	Authors declare: “Declaration of Interest: None known”
O’Brien *et al.* 2015 [12]	Authors declare: “The authors declare no conflict of interest”
Oostdam *et al.* 2011 [41]	Authors declare: “There are no potential conflicts of interest in connection with this article”
Oteng-Ntim *et al.* 2012 [13]	Authors declare: “The authors declare that they have no competing interests”
Quinlivan *et al.* 2011 [42]	Authors declare: “The authors did not report any potential conflicts of interest”
Rogozińska *et al.* 2015 [14]	Authors declare: “The authors have declared that no competing interests exist”
Russo *et al.* 2015 [49]	Authors declare: “The authors did not report any potential conflicts of interest”
Sanabria-Martínez *et al.* 2015 [50]	Authors declare: “None declared”
Streuling *et al.* 2011 [51]	Authors declare: “None of the authors had any conflicts of interest”
Sui *et al.* 2012 [52]	Authors declare: “The authors have stated explicitly that there are no conflicts of interest in connection with this article”
Tanentsapf *et al.* 2011 [15]	Authors declare: “The authors declare that they have no competing interests”
Thangaratinam *et al.* 2012a [16]	Authors declare: “None declared”
Thangaratinam *et al.* 2012b [17]	Authors declare: “Competing interest: All authors have completed the ICMJE uniform disclosure form at www.icmje.org/coi_disclosure.pdf (available on request from the corresponding author) and declare: no support from any organisation for the submitted work; no financial relationships with any organisations that might have an interest in the submitted work in the previous three years; no other relationships or activities that could appear to have influenced the submitted work.”
Chamberlain *et al.* 2017 [1]	Authors declare: “Catherine Chamberlain is receiving an Australian National Health and Medical Research Council Early Career Fellowship (1088813). She was also awarded an NIHR Cochrane Review Incentive Scheme award (15/81/18) to support the preparation of this updated review. Catherine Chamberlain is also an author on the Cochrane Review entitled ’Pharmacological Interventions to promote smoking cessation in pregnancy” (Coleman 2015). Alison O’Mara-Eves: none known. Jessie Porter: none known. Tim Coleman has received awards from NIHR, HTA and NIHR paid to his institution. These awards have been used to run research projects. He has also received a single payment from Pierre Fabre Laboratories, France, for speaking at an educational meeting arranged by Pierre Fabre Laboratories (who are manufacturers of nicotine replacement therapy). PFL are a manufacturer of transdermal nicotine patches - the content of the presentation was not vetted and no attempt was made to influence the content of the presentation. Tim Coleman is also an author and contact person for the Cochrane Review entitled ’Pharmacological Interventions to promote smoking cessation in pregnancy” (Coleman 2015). Susan M Perlen: none known. James Thomas: none known. Joanne E McKenzie: none known.”

Systematic review author, year	Author reported conflict of interest
da Silva *et al.* 2017 [53]	Authors declare: "Shana G da Silva, Luiza I Ricardo, Kelly R Evenson, and Pedro C Hallal have no conflicts of interest relevant to the content of this review."
Di Mascio *et al.* 2016 [54]	Authors declare: "The authors report no conflict of interest."
IWiP 2017 [18]	Authors declare: "No support from any organisation for the submitted work."
Lau *et al.* 2017 [19]	Authors declare: "no conflict of interest"
Magro-Malosso *et al* 2017a [20]	Authors declare: "that there are no conflicts of interest in connection with this article."
Magro-Malosso *et al* 2017b [21]	Authors declare: "that there are no conflicts of interest in connection with this article."
Sanabria-Martínez *et al.* 2016 [55]	Authors declare: "The author(s) declared no potential conflicts of interest with respect to the research, authorship, and/or publication of this article "
Shepherd *et al.* 2017 [22]	Authors declare: "Emily Shepherd: none known. Judith Gomersall: none known. Joanna Tieu has received funding for work outside of the scope of this review- NHMRC postgraduate scholarship, Ken Muirden fellowship (administered by Arthritis Australia; jointly funded by Australian Rheumatology Association and Roche). Shanshan Han: Shanshan Han was an investigator on one of the excluded trials (Crowther 2012). Assessment of eligibility for inclusion was carried out by other members of the review team who were not directly involved in the trial. Caroline Crowther: Caroline Crowther was an investigator on one of the included trials (Dodd 2014), and one of the excluded trials (Crowther 2012). All tasks relating to these trials (assessment of eligibility for inclusion, and if applicable, data extraction and assessment of risk of bias) were carried out by other members of the review team who were not directly involved in the trials. Philippa Middleton: Philippa Middleton was an investigator on one of the excluded trials (Crowther 2012). Assessment of eligibility for inclusion was carried out by other members of the review team who were not directly involved in the trial."
Sherifali *et al.* 2017 [23]	Authors declare: "None declared"
Song *et al.* 2016 [24]	Authors declare: "All the authors declared no conflicts of interest"
Tieu *et al.* 2017 [43]	Authors declare: "Declarations of interest: Joanna Tieu: none known. Emily Shepherd: none known. Philippa Middleton: none known. Caroline A Crowther: none known."
Veisani *et al.* 2017 [2]	Authors declare: "No potential conflict of interest was reported by the authors."
Yeo *et al.* 2017 [25]	Authors declare; "No conflict of interest was declared"
Zhang *et al.* 2018 [44]	Authors declare: "Irma Silva-Zolezzi is employee of Nestlé Research Center, Lausanne. Gerard Vinyes Parés and Yi Wang are employees of Nestlé Research Center Beijing. The authors state that there is no conflict of interest"
Zheng *et al.* 2017 [56]	Authors declare: "The authors declare no conflict of interest"
Gresham *et al.* 2014 [45]	Authors declare: "None of the authors had a conflict of interest"
Chatzakis *et al*. 2019 [57]	Authors declare: "The authors have no conflict of interest to declare"
Craemer *et al.* 2019 [26]	Authors declare: "The authors of this study declare no conflict of interest"
Davenport *et al.* 2019a [27]	Authors declare: "None declared"
Davenport *et al.* 2018a [28]	Authors declare: "None declared"
Davenport *et al.* 2018b [29]	Authors declare: "No competing interest"
Davenport *et al.* 2019b [30]	Authors declare: "No competing interest"
Davenport *et al.* 2018c [31]	Authors declare: "None declared"
**Du *et al*. 2019 [58]**	**No statement of conflict of interest included in the published systematic review**
Guillemette *et al.* 2018 [59]	Authors declare: "Laetitia Guillemette, Jacqueline L. Hay, D. Scott Kehler, Naomi C. Hamm, Christopher Oldfield, Jonathan M. McGavock, and Todd A. Duhamel declare that they have no competing interests"
Michel *et al.* 2019 [32]	Authors declare; "No conflict of interest was declared"
Ming *et al.* 2018 [60]	Authors declare: "No competing interests"
Morison *et al.* 2018 [33]	Authors declare: "No conflict of interest"
Nasiri-Amiri *et al.* 2019 [61]	Authors declare: "No competing interests"
Ruchat *et al.* 2018 [34]	Authors declare: "None declared"
Syngelaki *et al.* 2019 [35]	Authors declare "No potential conflict of interest"
Wang *et al.* 2019 [62]	Authors declare: "No conflicts of interest to disclose"
Poyatos-Leon *et al.* 2015 [63]	Authors declare: "The authors have stated explicitly that there are no conflicts of interest in connection with this article"
Walker *et al.* 2018 [36]	Authors declare: "The authors declared no conflicts of interest"
Vincze *et al.* 2019 [37]	Authors declare: "There is no conflict of interest in this project"
Wiebe *et al.* 2015 [64]	Authors declare: "The authors did not report any potential conflicts of interest"
Yin *et al.* 2013 [65]	Authors declare: "Competing interests – none"
Shieh *et al.* 2018 [38]	Authors declare: "Authors have no conflict of interest to declare"
Bennett *et al.* 2017 [39]	Authors declare: "The authors have no conflicts of interest to declare"

Note: some of the same systematic reviews were included in diet and/or physical activity, diet only and physical activity only conflict of interest summary tables depending on whether they reported data for all or a combination of intervention types, whereas S19e Table includes each systematic review once (out of a total of 66 included reviews).
Bold data represent the included systematic reviews which did not report any conflict-of-interest statement.
Amber highlighted data represent the included systematic reviews which reported potential conflicts of interest.

S19 References:

1. Chamberlain C, O'Mara-Eves A, Porter J, Coleman T, Perlen SM, Thomas J, et al. Psychosocial interventions for supporting women to stop smoking in pregnancy. The Cochrane database of systematic reviews. 2017;2:Cd001055. Epub 2017/02/15. doi: 10.1002/14651858.CD001055.pub5. PubMed PMID: 28196405; PubMed Central PMCID: PMCPMC6472671.
2. Veisani Y, Jenabi E, Delpisheh A, Khazaei S. Effect of prenatal smoking cessation interventions on birth weight: meta-analysis. The journal of maternal-fetal & neonatal medicine : the official journal of the European Association of Perinatal Medicine, the Federation of Asia and Oceania Perinatal Societies, the International Society of Perinatal Obstet. 2019;32(2):332-8. Epub 2017/09/12. doi: 10.1080/14767058.2017.1378335. PubMed PMID: 28889768.

3. Agha M, Agha RA, Sandall J. Interventions to reduce and prevent obesity in pre-conceptual and pregnant women: a systematic review and meta-analysis. PloS one. 2014;9(5):e95132-e. doi: 10.1371/journal.pone.0095132. PubMed PMID: 24827704.
4. Bain E, Crane M, Tieu J, Han S, Crowther CA, Middleton P. Diet and exercise interventions for preventing gestational diabetes mellitus. The Cochrane database of systematic reviews. 2015;(4):Cd010443. Epub 2015/04/13. doi: 10.1002/14651858.CD010443.pub2. PubMed PMID: 25864059.
5. Allen R, Rogozinska E, Sivarajasingam P, Khan KS, Thangaratinam S. Effect of diet- and lifestyle-based metabolic risk-modifying interventions on preeclampsia: a meta-analysis. Acta Obstetricia et Gynecologica Scandinavica. 2014;93(10):973-85. doi: 10.1111/aogs.12467.
6. Campbell F, Johnson M, Messina J, Guillaume L, Goyder E. Behavioural interventions for weight management in pregnancy: a systematic review of quantitative and qualitative data. BMC Public Health. 2011;11:491. Epub 2011/06/24. doi: 10.1186/1471-2458-11-491. PubMed PMID: 21696589; PubMed Central PMCID: PMCPMC3154865.
7. Choi J, Fukuoka Y, Lee JH. The effects of physical activity and physical activity plus diet interventions on body weight in overweight or obese women who are pregnant or in postpartum: a systematic review and meta-analysis of randomized controlled trials. Prev Med. 2013;56(6):351-64. Epub 2013/02/26. doi: 10.1016/j.ypmed.2013.02.021. PubMed PMID: 23480971.
8. Gardner B, Wardle J, Poston L, Croker H. Changing diet and physical activity to reduce gestational weight gain: a meta-analysis. Obesity reviews : an official journal of the International Association for the Study of Obesity. 2011;12(7):e602-20. Epub 2011/04/28. doi: 10.1111/j.1467-789X.2011.00884.x. PubMed PMID: 21521451.
9. Hill B, Skouteris H, Fuller-Tyszkiewicz M. Interventions designed to limit gestational weight gain: a systematic review of theory and meta-analysis of intervention components. Obesity Reviews. 2013;14(6):435-50. doi: 10.1111/obr.12022.
10. Madhuvrata P, Govinden G, Bustani R, Song S, Farrell TA. Prevention of gestational diabetes in pregnant women with risk factors for gestational diabetes: a systematic review and meta-analysis of randomised trials. Obstet Med. 2015;8(2):68-85. Epub 2015/06/01. doi: 10.1177/1753495x15576673. PubMed PMID: 27512459; PubMed Central PMCID: PMCPMC4935009.
11. Muktabhant B, Lawrie TA, Lumbiganon P, Laopaiboon M. Diet or exercise, or both, for preventing excessive weight gain in pregnancy. The Cochrane database of systematic reviews. 2015;(6):Cd007145. Epub 2015/06/13. doi: 10.1002/14651858.CD007145.pub3. PubMed PMID: 26068707.
12. O'Brien CM, Grivell RM, Dodd JM. Systematic review of antenatal dietary and lifestyle interventions in women with a normal body mass index. Acta Obstet Gynecol Scand. 2016;95(3):259-69. Epub 2015/11/27. doi: 10.1111/aogs.12829. PubMed PMID: 26610169.
13. Oteng-Ntim E, Varma R, Croker H, Poston L, Doyle P. Lifestyle interventions for overweight and obese pregnant women to improve pregnancy outcome: systematic review and meta-analysis. BMC Medicine. 2012;10(1):47. doi: 10.1186/1741-7015-10-47.
14. Rogozińska E, Chamillard M, Hitman GA, Khan KS, Thangaratinam S. Nutritional manipulation for the primary prevention of gestational diabetes mellitus: a meta-analysis of randomised studies. PloS one. 2015;10(2):e0115526. Epub 2015/02/27. doi: 10.1371/journal.pone.0115526. PubMed PMID: 25719363; PubMed Central PMCID: PMCPMC4342242.
15. Tanentsapf I, Heitmann BL, Adegboye ARA. Systematic review of clinical trials on dietary interventions to prevent excessive weight gain during pregnancy among normal weight, overweight and obese women. BMC Pregnancy and Childbirth. 2011;11(1):81. doi: 10.1186/1471-2393-11-81.
16. Thangaratinam S, Rogozińska E, Jolly K, Glinkowski S, Duda W, Borowiack E, et al. Interventions to reduce or prevent obesity in pregnant women: a systematic review. 2012;16:31. doi: 10.3310/hta16310.
17. Thangaratinam S, Rogozińska E, Jolly K, Glinkowski S, Roseboom T, Tomlinson JW, et al. Effects of interventions in pregnancy on maternal weight and obstetric outcomes: meta-analysis of randomised evidence. BMJ : British Medical Journal. 2012;344:e2088. doi: 10.1136/bmj.e2088.
18. International Weight Management in Pregnancy (IWiP) collaboration. Effect of diet and physical activity based interventions in pregnancy on gestational weight gain and pregnancy outcomes: meta-analysis of individual participant data from randomised trials. BMJ (Clinical research ed). 2017;358:j3119. doi: 10.1136/bmj.j3119.
19. Lau Y, Klainin-Yobas P, Htun TP, Wong SN, Tan KL, Ho-Lim ST, et al. Electronic-based lifestyle interventions in overweight or obese perinatal women: a systematic review and meta-analysis. Obesity reviews : an official journal of the International Association for the Study of Obesity. 2017;18(9):1071-87. Epub 2017/05/26. doi: 10.1111/obr.12557. PubMed PMID: 28544551.
20. Magro-Malosso ER, Saccone G, Di Tommaso M, Roman A, Berghella V. Exercise during pregnancy and risk of gestational hypertensive disorders: a systematic review and meta-analysis. Acta Obstetricia et Gynecologica Scandinavica. 2017;96(8):921-31. doi: 10.1111/aogs.13151.
21. Magro-Malosso ER, Saccone G, Di Mascio D, Di Tommaso M, Berghella V. Exercise during pregnancy and risk of preterm birth in overweight and obese women: a systematic review and meta-analysis of randomized controlled trials. Acta Obstet Gynecol Scand. 2017;96(3):263-73. Epub 2016/12/29. doi: 10.1111/aogs.13087. PubMed PMID: 28029178.
22. Shepherd E, Gomersall JC, Tieu J, Han S, Crowther CA, Middleton P. Combined diet and exercise interventions for preventing gestational diabetes mellitus. The Cochrane database of systematic reviews. 2017;11:Cd010443. Epub

2017/11/13. doi: 10.1002/14651858.CD010443.pub3. PubMed PMID: 29129039; PubMed Central PMCID: PMCPMC6485974.
23. Sherifali D, Nerenberg KA, Wilson S, Semeniuk K, Ali MU, Redman LM, et al. The Effectiveness of eHealth Technologies on Weight Management in Pregnant and Postpartum Women: Systematic Review and Meta-Analysis. Journal of medical Internet research. 2017;19(10):e337. Epub 2017/10/17. doi: 10.2196/jmir.8006. PubMed PMID: 29030327; PubMed Central PMCID: PMCPMC5660296.
24. Song C, Li J, Leng J, Ma RC, Yang X. Lifestyle intervention can reduce the risk of gestational diabetes: a meta-analysis of randomized controlled trials. Obesity reviews : an official journal of the International Association for the Study of Obesity. 2016;17(10):960-9. Epub 2016/07/16. doi: 10.1111/obr.12442. PubMed PMID: 27417680.
25. Yeo S, Walker JS, Caughey MC, Ferraro AM, Asafu-Adjei JK. What characteristics of nutrition and physical activity interventions are key to effectively reducing weight gain in obese or overweight pregnant women? A systematic review and meta-analysis. Obesity reviews : an official journal of the International Association for the Study of Obesity. 2017;18(4):385-99. Epub 2017/02/09. doi: 10.1111/obr.12511. PubMed PMID: 28177566.
26. Craemer KA, Sampene E, Safdar N, Antony KM, Wautlet CK. Nutrition and Exercise Strategies to Prevent Excessive Pregnancy Weight Gain: A Meta-analysis. AJP Rep. 2019;9(1):e92-e120. Epub 2019/03/19. doi: 10.1055/s-0039-1683377. PubMed PMID: 31041118.
27. Davenport MH, Kathol AJ, Mottola MF, Skow RJ, Meah VL, Poitras VJ, et al. Prenatal exercise is not associated with fetal mortality: a systematic review and meta-analysis. Br J Sports Med. 2019;53(2):108-15. Epub 2018/10/20. doi: 10.1136/bjsports-2018-099773. PubMed PMID: 30337346.
28. Davenport MH, McCurdy AP, Mottola MF, Skow RJ, Meah VL, Poitras VJ, et al. Impact of prenatal exercise on both prenatal and postnatal anxiety and depressive symptoms: a systematic review and meta-analysis. British Journal of Sports Medicine. 2018;52(21):1376. doi: 10.1136/bjsports-2018-099697.
29. Davenport MH, Ruchat SM, Poitras VJ, Jaramillo Garcia A, Gray CE, Barrowman N, et al. Prenatal exercise for the prevention of gestational diabetes mellitus and hypertensive disorders of pregnancy: a systematic review and meta-analysis. Br J Sports Med. 2018;52(21):1367-75. Epub 2018/10/20. doi: 10.1136/bjsports-2018-099355. PubMed PMID: 30337463.
30. Davenport MH, Ruchat SM, Sobierajski F, Poitras VJ, Gray CE, Yoo C, et al. Impact of prenatal exercise on maternal harms, labour and delivery outcomes: a systematic review and meta-analysis. Br J Sports Med. 2019;53(2):99-107. Epub 2018/10/20. doi: 10.1136/bjsports-2018-099821. PubMed PMID: 30337349.
31. Davenport MH, Sobierajski F, Mottola MF, Skow RJ, Meah VL, Poitras VJ, et al. Glucose responses to acute and chronic exercise during pregnancy: a systematic review and meta-analysis. Br J Sports Med. 2018;52(21):1357-66. Epub 2018/10/20. doi: 10.1136/bjsports-2018-099829. PubMed PMID: 30337462.
32. Michel S, Raab R, Drabsch T, Günther J, Stecher L, Hauner H. Do lifestyle interventions during pregnancy have the potential to reduce long-term postpartum weight retention? A systematic review and meta-analysis. Obesity Reviews. 2019;20(4):527-42. doi: 10.1111/obr.12809.
33. Morison PN, Bacardi-Gascon M, Lopez-Corrales M, Jimenez-Cruz A. Combined dietary-exercise intervention for gestational weight gain and birthweight: a meta-analysis. Asia Pac J Clin Nutr. 2018;27(4):860-8. Epub 2018/07/27. doi: 10.6133/apjcn.112017.02. PubMed PMID: 30045432.
34. Ruchat S-M, Mottola MF, Skow RJ, Nagpal TS, Meah VL, James M, et al. Effectiveness of exercise interventions in the prevention of excessive gestational weight gain and postpartum weight retention: a systematic review and meta-analysis. British Journal of Sports Medicine. 2018;52(21):1347. doi: 10.1136/bjsports-2018-099399.
35. Syngelaki A, Sequeira Campos M, Roberge S, Andrade W, Nicolaides KH. Diet and exercise for preeclampsia prevention in overweight and obese pregnant women: systematic review and meta-analysis. The journal of maternal-fetal & neonatal medicine : the official journal of the European Association of Perinatal Medicine, the Federation of Asia and Oceania Perinatal Societies, the International Society of Perinatal Obstet. 2019;32(20):3495-501. Epub 2018/05/25. doi: 10.1080/14767058.2018.1481037. PubMed PMID: 29792061.
36. Walker R, Bennett C, Blumfield M, Gwini S, Ma J, Wang F, et al. Attenuating Pregnancy Weight Gain-What Works and Why: A Systematic Review and Meta-Analysis. Nutrients. 2018;10(7):944. doi: 10.3390/nu10070944. PubMed PMID: 30037126.
37. Vincze L, Rollo M, Hutchesson M, Hauck Y, MacDonald-Wicks L, Wood L, et al. Interventions including a nutrition component aimed at managing gestational weight gain or postpartum weight retention: a systematic review and meta-analysis. JBI Evidence Synthesis. 2019;17(3):297-364. doi: 10.11124/jbisrir-2017-003593. PubMed PMID: 01938924-201903000-00007.
38. Shieh C, Cullen DL, Pike C, Pressler SJ. Intervention strategies for preventing excessive gestational weight gain: systematic review and meta-analysis. Obesity Reviews. 2018;19(8):1093-109. doi: https://doi.org/10.1111/obr.12691.
39. Bennett CJ, Walker RE, Blumfield ML, Gwini S-M, Ma J, Wang F, et al. Interventions designed to reduce excessive gestational weight gain can reduce the incidence of gestational diabetes mellitus: A systematic review and meta-analysis of randomised controlled trials. Diabetes research and clinical practice. 2018;141:69-79. doi: 10.1016/j.diabres.2018.04.010. PubMed PMID: 29698713.

40. Gresham E, Bisquera A, Byles JE, Hure AJ. Effects of dietary interventions on pregnancy outcomes: a systematic review and meta-analysis. Matern Child Nutr. 2016;12(1):5-23. Epub 2014/07/23. doi: 10.1111/mcn.12142. PubMed PMID: 25048387; PubMed Central PMCID: PMCPMC6860081.
41. Oostdam N, van Poppel MN, Wouters MG, van Mechelen W. Interventions for preventing gestational diabetes mellitus: a systematic review and meta-analysis. Journal of women's health (2002). 2011;20(10):1551-63. Epub 2011/08/16. doi: 10.1089/jwh.2010.2703. PubMed PMID: 21838525.
42. Quinlivan JA, Julania S, Lam L. Antenatal dietary interventions in obese pregnant women to restrict gestational weight gain to Institute of Medicine recommendations: a meta-analysis. Obstet Gynecol. 2011;118(6):1395-401. Epub 2011/11/23. doi: 10.1097/AOG.0b013e3182396bc6. PubMed PMID: 22105270.
43. Tieu J, Shepherd E, Middleton P, Crowther CA. Dietary advice interventions in pregnancy for preventing gestational diabetes mellitus. The Cochrane database of systematic reviews. 2017;1:Cd006674. Epub 2017/01/04. doi: 10.1002/14651858.CD006674.pub3. PubMed PMID: 28046205; PubMed Central PMCID: PMCPMC6464792.
44. Zhang R, Han S, Chen GC, Li ZN, Silva-Zolezzi I, Parés GV, et al. Effects of low-glycemic-index diets in pregnancy on maternal and newborn outcomes in pregnant women: a meta-analysis of randomized controlled trials. Eur J Nutr. 2018;57(1):167-77. Epub 2016/09/11. doi: 10.1007/s00394-016-1306-x. PubMed PMID: 27612876.
45. Gresham E, Byles JE, Bisquera A, Hure AJ. Effects of dietary interventions on neonatal and infant outcomes: a systematic review and meta-analysis. Am J Clin Nutr. 2014;100(5):1298-321. Epub 2014/10/22. doi: 10.3945/ajcn.113.080655. PubMed PMID: 25332328.
46. Elliott-Sale KJ, Barnett CT, Sale C. Exercise interventions for weight management during pregnancy and up to 1 year postpartum among normal weight, overweight and obese women: a systematic review and meta-analysis. Br J Sports Med. 2015;49(20):1336-42. Epub 2014/11/20. doi: 10.1136/bjsports-2014-093875. PubMed PMID: 25406335.
47. Han S, Middleton P, Crowther CA. Exercise for pregnant women for preventing gestational diabetes mellitus. The Cochrane database of systematic reviews. 2012;(7):Cd009021. Epub 2012/07/13. doi: 10.1002/14651858.CD009021.pub2. PubMed PMID: 22786521.
48. Lamina S, Agbanusi E. Effect of aerobic exercise training on maternal weight gain in pregnancy: a meta-analysis of randomized controlled trials. Ethiop J Health Sci. 2013;23(1):59-64. PubMed PMID: 23559839.
49. Russo LM, Nobles C, Ertel KA, Chasan-Taber L, Whitcomb BW. Physical activity interventions in pregnancy and risk of gestational diabetes mellitus: a systematic review and meta-analysis. Obstet Gynecol. 2015;125(3):576-82. Epub 2015/03/03. doi: 10.1097/aog.0000000000000691. PubMed PMID: 25730218.
50. Sanabria-Martínez G, García-Hermoso A, Poyatos-León R, Álvarez-Bueno C, Sánchez-López M, Martínez-Vizcaíno V. Effectiveness of physical activity interventions on preventing gestational diabetes mellitus and excessive maternal weight gain: a meta-analysis. BJOG : an international journal of obstetrics and gynaecology. 2015;122(9):1167-74. Epub 2015/06/04. doi: 10.1111/1471-0528.13429. PubMed PMID: 26036300.
51. Streuling I, Beyerlein A, Rosenfeld E, Hofmann H, Schulz T, von Kries R. Physical activity and gestational weight gain: a meta-analysis of intervention trials. BJOG : an international journal of obstetrics and gynaecology. 2011;118(3):278-84. Epub 2010/12/08. doi: 10.1111/j.1471-0528.2010.02801.x. PubMed PMID: 21134106.
52. Sui Z, Grivell RM, Dodd JM. Antenatal exercise to improve outcomes in overweight or obese women: A systematic review. Acta Obstet Gynecol Scand. 2012;91(5):538-45. Epub 2012/01/11. doi: 10.1111/j.1600-0412.2012.01357.x. PubMed PMID: 22229625.
53. da Silva SG, Ricardo LI, Evenson KR, Hallal PC. Leisure-Time Physical Activity in Pregnancy and Maternal-Child Health: A Systematic Review and Meta-Analysis of Randomized Controlled Trials and Cohort Studies. Sports Med. 2017;47(2):295-317. Epub 2016/06/11. doi: 10.1007/s40279-016-0565-2. PubMed PMID: 27282925.
54. Di Mascio D, Magro-Malosso ER, Saccone G, Marhefka GD, Berghella V. Exercise during pregnancy in normal-weight women and risk of preterm birth: a systematic review and meta-analysis of randomized controlled trials. American journal of obstetrics and gynecology. 2016;215(5):561-71. Epub 2016/10/30. doi: 10.1016/j.ajog.2016.06.014. PubMed PMID: 27319364.
55. Sanabria-Martínez G, García-Hermoso A, Poyatos-León R, González-García A, Sánchez-López M, Martínez-Vizcaíno V. Effects of Exercise-Based Interventions on Neonatal Outcomes: A Meta-Analysis of Randomized Controlled Trials. Am J Health Promot. 2016;30(4):214-23. Epub 2016/07/13. doi: 10.1177/0890117116639569. PubMed PMID: 27404056.
56. Zheng J, Wang H, Ren M. Influence of exercise intervention on gestational diabetes mellitus: a systematic review and meta-analysis. J Endocrinol Invest. 2017;40(10):1027-33. Epub 2017/04/13. doi: 10.1007/s40618-017-0673-3. PubMed PMID: 28401529.
57. Chatzakis C, Goulis DG, Mareti E, Eleftheriades M, Zavlanos A, Dinas K, et al. Prevention of gestational diabetes mellitus in overweight or obese pregnant women: A network meta-analysis. Diabetes Res Clin Pract. 2019;158:107924. Epub 2019/11/19. doi: 10.1016/j.diabres.2019.107924. PubMed PMID: 31738997.
58. Du MC, Ouyang YQ, Nie XF, Huang Y, Redding SR. Effects of physical exercise during pregnancy on maternal and infant outcomes in overweight and obese pregnant women: A meta-analysis. Birth. 2019;46(2):211-21. Epub 2018/09/22. doi: 10.1111/birt.12396. PubMed PMID: 30240042.

59. Guillemette L, Hay JL, Kehler DS, Hamm NC, Oldfield C, McGavock JM, et al. Exercise in Pregnancy and Children's Cardiometabolic Risk Factors: a Systematic Review and Meta-Analysis. Sports Med Open. 2018;4(1):35-. doi: 10.1186/s40798-018-0148-x. PubMed PMID: 30069801.
60. Ming W-K, Ding W, Zhang CJP, Zhong L, Long Y, Li Z, et al. The effect of exercise during pregnancy on gestational diabetes mellitus in normal-weight women: a systematic review and meta-analysis. BMC Pregnancy and Childbirth. 2018;18(1):440. doi: 10.1186/s12884-018-2068-7.
61. Nasiri-Amiri F, Sepidarkish M, Shirvani MA, Habibipour P, Tabari NSM. The effect of exercise on the prevention of gestational diabetes in obese and overweight pregnant women: a systematic review and meta-analysis. Diabetology & Metabolic Syndrome. 2019;11(1):72. doi: 10.1186/s13098-019-0470-6.
62. Wang J, Wen D, Liu X, Liu Y. Impact of exercise on maternal gestational weight gain: An updated meta-analysis of randomized controlled trials. Medicine (Baltimore). 2019;98(27):e16199-e. doi: 10.1097/MD.0000000000016199. PubMed PMID: 31277127.
63. Poyatos-León R, García-Hermoso A, Sanabria-Martínez G, Álvarez-Bueno C, Sánchez-López M, Martínez-Vizcaíno V. Effects of exercise during pregnancy on mode of delivery: a meta-analysis. Acta Obstet Gynecol Scand. 2015;94(10):1039-47. Epub 2015/05/13. doi: 10.1111/aogs.12675. PubMed PMID: 25965378.
64. Wiebe HW, Boulé NG, Chari R, Davenport MH. The Effect of Supervised Prenatal Exercise on Fetal Growth: A Meta-analysis. Obstetrics & Gynecology. 2015;125(5):1185-94. doi: 10.1097/aog.0000000000000801. PubMed PMID: 00006250-201505000-00028.
65. Yin YN, Li XL, Tao TJ, Luo BR, Liao SJ. Physical activity during pregnancy and the risk of gestational diabetes mellitus: a systematic review and meta-analysis of randomised controlled trials. Br J Sports Med. 2014;48(4):290-5. Epub 2013/09/17. doi: 10.1136/bjsports-2013-092596. PubMed PMID: 24037671.
